# Health related quality of life measure in systemic pediatric rheumatic diseases and its translation to different languages: an international collaboration

**DOI:** 10.1186/1546-0096-12-49

**Published:** 2014-11-25

**Authors:** Lakshmi Nandini Moorthy, Elizabeth Roy, Vamsi Kurra, Margaret GE Peterson, Afton L Hassett, Thomas JA Lehman, Members of our collaborative group, Christiaan Scott, Dalia El-Ghoneimy, Shereen Saad, Reem El Feky, Sulaiman Al-Mayouf, Pavla Dolezalova, Hana Malcova, Troels Herlin, Susan Nielsen, Nico Wulffraat, Annet van Royen, Stephen D Marks, Alexandre Belot, Jurgen Brunner, Christian Huemer, Ivan Foeldvari, Gerd Horneff, Traudel Saurenman, Silke Schroeder, Polyxeni Pratsidou-Gertsi, Maria Trachana, Yosef Uziel, Amita Aggarwal, Tamas Constantin, Rolando Cimaz, Theresa Giani, Luca Cantarini, Fernanda Falcini, Silvia Magni Manzoni, Angelo Ravelli, Donato Rigante, Fracnceso Zulian, Takako Miyamae, Shumpei Yokota, Juliana Sato, Claudia S Magalhaes, Claudio A Len, Simone Appenzeller, Sheila Oliveira Knupp, Marta Cristine Rodrigues, Flavio Sztajnbok, Rozana Gasparello de Almeida, Adriana Almeida de Jesus, Lucia Maria de Arruda Campos, Clovis Silva, Calin Lazar, Gordana Susic, Tadej Avcin, Ruben Cuttica, Ruben Burgos-Vargas, Enrique Faugier, Jordi Anton, Consuelo Modesto, Liza Vazquez, Lilliana Barillas, Laura Barinstein, Gary Sterba, Irama Maldonado, Seza Ozen, Ozgur Kasapcopur, Erkan Demirkaya, Susa Benseler

**Affiliations:** Robert Wood Johnson Medical School, Rutgers University, New Brunswick, NJ USA; Pediatric Rheumatology, Rutgers University Child Health Institute of New Jersey, 89 French Street, New Brunswick, NJ 08901 USA; Hospital for Special Surgery, New York, NY USA; University of Michigan, Ann Arbor, MI USA; Pediatric Rheumatology, Red Cross War Memorial Children’s Hospital, Cape Town, South Africa; Pediatric Allergy, Immunology and Rheumatology Unit, Ain Shams University, Cairo, Egypt; Pediatric Rheumatology, Pediatric Allergy, Immunology and Rheumatology Unit, Ain Shams University, Cairo, Egypt; King Faisal Specialist Hospital and Research Center, Riyadh, Saudi Arabia; Charles University in Prague and General University Hospital, Prague, Czech Republic; Pediatric Department, University Hospital Motol, Prague, Czech Republic; Pediatric Rheumatology, Aarhus University Hospital Skejby, Aarhus, Denmark; Pediatric Rheumatology, Juliane Marie Centret Rigshospitalet, Copenhagen, Denmark; Department of Pediatric Immunology, University Medical Center, Utrecht, Netherlands; Pediatric Rheumatology, Wilhelmina Children’s Hospital, Utrecht, Netherlands; Consultant Pediatric Nephrologist, Renal Unit, Great Ormond Street Hospital for Children NHS Foundation Trust, London, UK; Pediatric Nephrology, Rheumatology and Dermatology Department, Hospices Civils de Lyon, Lyon University, Lyon, France; Medical University of Innsbruck, Innsbruck, Austria; Pediatric Rheumatology, Prim. University Doz, Bregenz, Austria; Head of the Hamburg Centre for Pediatric and Adolescence Rheumatology, Hamburg, Germany; Pediatric Rheumatology, Asklepios Clinic Sankt, Augustin, Germany; Pediatric Rheumatology, Zurich University Children’s Hospital, Zürich, Switzerland; Pediatrics/Pediatric Rheumatology, Pediatric Immunology and Rheumatology Referral Center, Aristotle University, Thessaloniki, Greece; Kfar-Saba, Pediatric Rheumatology, Israel Meir Hospital, Kfar Saba, Israel; Pediatric Rheumatology, Sanjay Gandhi Postgraduate Institute of Medical Sciences, Lucknow, India; Pediatric Rheumatology, Semmelweis University, Budapest, Hungary; Pediatric Rheumatology, Anna Meyer Hospital, Florence, Italy; Pediatric Rheumatology, Research Center of Systemic Autoimmune and Autoinflammatory Diseases, University of Siena, Siena, Italy; Pediatric Rheumatology, University of Florence, Florence, Italy; Pediatric Rheumatology Unit IRCCS Ospedale Pediatrico Bambino Gesù, Rome, Italy; Pediatric Rheumatology, University of Genoa Pediatria II-Reumatologia, Istituto G. Gaslini EULAR Centre of Excellence in Rheumatology, Genova, Italy; Pediatric Rheumatology, Institute of Pediatrics Università Cattolica Sacro Cuore, Rome, Italy; Pediatric Rheumatology Unit, Department of Pediatrics, University of Padua, Padua, Italy; Yokohama City University School of Medicine, Yokohama, Japan; Pediatric Rheumatology, Universidade Estadual Paulista (UNESP), Botucatu, Brazil; Pediatric Rheumatology Unit, Department of Pediatrics, Universidade Federal de Sao Paulo, Sao Paulo, Brazil; Rheumatology unit, Department of Medicine, State University of Campinas, Campinas, Brazil; Department of Pediatrics, Pediatric Rheumatology, Universidade Federal do Rio de Janeiro, Rio de Janeiro, Brazil; Department of Pediatrics, Universidade Federal do Rio de Janeiro, Rio de Janeiro, Brazil; Pediatric Rheumatology Division, Adolescent Health Care Unit, Universidade do Estado do, Rio de Janeiro, Brazil; Department of Pediatrics, Pediatric Rheumatology Unit, Children’s Institute, Faculdade de Medicina da Universidade de Sao Paulo, Sao Paulo, Brazil; Pediatric Rheumatology Unit, Children’s Institute, Sao Paulo, Brazil; Pediatric Rheumatology, Clinica Pediatrie I, Cluj-Napoca, Romania; Pediatric Rheumatology, Institute of Rheumatology, Belgrade, Serbia; Allergy, Rheumatology and Clinical Immunology University Children’s Hospital, University Medical Center Ljubljana, Ljubljana, Slovenia; Pediatric Rheumatology, Head Rheumatology Hospital Pedro de Elizalde, Buenos Aires, Argentina; Pediatric Rheumatology, Hospital General de México, México City, México; Hospital Infantil de México Federico Gómez, México City, Mexico; Pediatric Rheumatology, Hospital Sant Joan de Déu, Barcelona, Spain; Pediatric Rheumatology, Hospital Universitario Valle de Hebron, Barcelona, Spain; Pediatric Rheumatology, Calle Convento # 252, San Juan, PR USA; Pediatric Rheumatology, 47 New Scotland Ave Suite 197, Albany, NY USA; Pediatric Rheumatology, Mt Sinai Medical Center, New York, NY USA; Rheumatology, Mount Sinai Medical Center, Miami Beach, FL USA; Pediatric Rheumatology, Complejo Hospitalario Universitario Ruiz y Paez, Unidad de Reumatologia, Bolivar, Venezuela; Pediatric Rheumatology, Hacettepe University Department of Pediatrics, Ankara, Turkey; Pediatric Rheumatology, Cerrahpasa Medical School, Istanbul University, Istanbul, Turkey; FMF Arthritis Vasculitis and Orphan disease Research Center, Institute of Health Sciences, Gata, Ankara Turkey; Department of Pediatrics, Alberta Children’s Hospital Research Institute Faculty of Medicine, University of Calgary, Calgary, AB Canada

## Abstract

**Background:**

Rheumatic diseases in children are associated with significant morbidity and poor health-related quality of life (HRQOL). There is no health-related quality of life (HRQOL) scale available specifically for children with less common rheumatic diseases. These diseases share several features with systemic lupus erythematosus (SLE) such as their chronic episodic nature, multi-systemic involvement, and the need for immunosuppressive medications. HRQOL scale developed for pediatric SLE will likely be applicable to children with systemic inflammatory diseases.

**Findings:**

We adapted Simple Measure of Impact of Lupus Erythematosus in Youngsters (SMILEY©) to Simple Measure of Impact of Illness in Youngsters (SMILY©-Illness) and had it reviewed by pediatric rheumatologists for its appropriateness and cultural suitability. We tested SMILY©-Illness in patients with inflammatory rheumatic diseases and then translated it into 28 languages.

Nineteen children (79% female, n=15) and 17 parents participated. The mean age was 12±4 years, with median disease duration of 21 months (1-172 months). We translated SMILY©-Illness into the following 28 languages: Danish, Dutch, French (France), English (UK), German (Germany), German (Austria), German (Switzerland), Hebrew, Italian, Portuguese (Brazil), Slovene, Spanish (USA and Puerto Rico), Spanish (Spain), Spanish (Argentina), Spanish (Mexico), Spanish (Venezuela), Turkish, Afrikaans, Arabic (Saudi Arabia), Arabic (Egypt), Czech, Greek, Hindi, Hungarian, Japanese, Romanian, Serbian and Xhosa.

**Conclusion:**

SMILY©-Illness is a brief, easy to administer and score HRQOL scale for children with systemic rheumatic diseases. It is suitable for use across different age groups and literacy levels. SMILY©-Illness with its available translations may be used as useful adjuncts to clinical practice and research.

**Electronic supplementary material:**

The online version of this article (doi:10.1186/1546-0096-12-49) contains supplementary material, which is available to authorized users.

## Findings

### Introduction

In children, chronic rheumatic diseases are associated with significant disease- and treatment-related morbidity, thus impacting their health-related quality of life (HRQOL). There are generic scales available to assess HRQOL in children with rheumatic diseases such as the Pediatric Quality of Life Inventory (PedsQL)-Rheumatology module [1]. But there is no specific health-related quality of life (HRQOL) scale that addresses the impact of the less common rheumatic diseases such as mixed connective tissue disease (MCTD), juvenile dermatomyositis (JDM), systemic sclerosis (SS), Sjögren’s syndrome, vasculitides, Behçets, sarcoidosis or systemic arthritis (SJIA).

Simple Measure of the Impact of Lupus Erythematosus in Youngsters©" (SMILEY) is valid in US-English [2] and in Portuguese-for Brazil [3]. SMILEY-US English was validated through a multicenter study in the US [2]. Subjects with SLE completed other gold standards and SLE status measures and psychometric properties were determined [2]. Relationship of HRQOL and changes in disease activity were measured over time [4]. SMILEY US English was further translated and adapted into several languages [2, 5, 6].

### Hypothesis

Systemic Lupus Erythematosus (SLE) and systemic inflammatory diseases share several features such as their chronic episodic nature, multi-systemic involvement, and the need for immunosuppressive medications. HRQOL scale developed for pediatric SLE will be applicable to children with systemic inflammatory diseases. We decided to adapt a tool that is valid in SLE, titled, SMILEY [2]. We will report the: (i) adaptation of SMILEY© to Simple Measure of Impact of Illness in Youngsters (SMILY©-Illness) for use in children with systemic inflammatory diseases such as MCTD, JDM, SS, Sjögren’s syndrome, vasculitis and SJIA and preliminary testing in patients; and (ii) translation into different languages. We think this is very important since the systemic rheumatic diseases mentioned above can lead to significant disability which impact HRQOL.

## Methods used

### Overview in brief

(i) We adapted SMILEY to SMILY**©-**Illness and had it reviewed by pediatric rheumatologists from two centers (RWJMS, HSS) for its appropriateness and cultural suitability, and tested SMILY**©-**Illness in as small sample. We examined time taken to complete questionnaire, feasibility, and also collected demographic and disease-related data. We subsequently translated it into the following 28 languages using professional translators: Danish, Dutch, French (France), English (UK), German (Germany), German (Austria), German (Switzerland), Hebrew, Italian, Portuguese(Brazil), Slovene, Spanish (USA and Puerto Rico), Spanish (Spain), Spanish (Argentina), Spanish (Mexico), Spanish (Venezuela ), Turkish, Afrikaans, Arabic (Saudi Arabia), Arabic (Egypt), Czech, Greek, Hindi, Hungarian, Japanese, Romanian, Serbian and Xhosa. Each translation was reviewed by the pediatric rheumatologist(s) from that country for its applicability and cultural suitability in order to be approved.

### Subjects and settings

Children ≤18 years of age diagnosed with the following systemic chronic rheumatic diseases were included: MCTD, JDM, Sjögren’s syndrome, Systemic sclerosis/CREST, Behçets, sarcoidosis and SJIA. The patients who had to have been followed for at least one month, and able to participate in the study as determined by the pediatric rheumatologist, and their parents (or guardians) were recruited from two US pediatric rheumatology practices^a^. Children were excluded if they were unable to complete the questionnaires, or had a significant co-morbid condition likely to impact HRQOL exclusive of their rheumatic disease (such as an infectious, endocrine, psychiatric, congenital, genetic, neurodegenerative or an oncological process).

### Measures used

The 26-item SMILY©-Illness for children <19 years features parallel child self reports and parent reports with responses in the form of a five-step scale with different facial expressions with 5^th^ grade reading level. The four domains are similar to that of SMILEY and are: Effect on self (5 items), Limitations (8 items), Social (4 items) and Burden of Illness (7 items). Scoring is also similar to SMILEY, where each item score ranges from 1 to 5 and the total score is transformed to a 1 to 100 scale. Higher scores indicate better HRQOL. If >12 questions are not answered, the SMILY©-Illness cannot be scored. The first two items on current illness status and HRQOL assessment are not included in the domains or calculating the final score. The remaining questions refer the respondent to the previous month.

### Additional data

We examined self-esteem using the Piers Harris Self concept scale (SCS) [7, 8], entitled, “The Way I Feel About Myself.” Average scores usually range from 46–60 with higher scores corresponding to better self-concept. We collected data on demographics, ethnicity, co-morbidity, insurance, education; and impact of disease using the PedsQL-Family information form. We recorded the date of disease onset, and the current/prior use of all medication(s). The Hollingshead Socioeconomic scale (SES) score, which takes into account the educational and occupational status of the family members, was calculated using the educational and occupational status of the parents [9]. The scores range from 8 to 66, with higher scores indicating a higher socioeconomic status [9].

### Procedure

Appropriate Institutional Review Board approval was obtained at both sites. Potential subjects were identified at each center through the clinic appointment schedule or during in-patient admissions. Children and parents completed corresponding versions of the 26-item SMILY**©**-Illness and the SCS. The investigator was available at all times to respond to queries posed by study respondents.

### Methods and statistical analysis

Using the SPSS statistical package for Windows (SPSS Inc, Chicago, Illinois versions 20), we performed descriptive analyses on all variables and examined data distribution, and examined instrument scores for ceiling and floor effects. Minimal missing data were handled in accordance with rules for scoring each questionnaire. Feasibility was determined from the percentage of missing values for each item and the distribution of item responses [10]. Spearman’s rho correlation was used.

## Results

Nineteen children (79% female, n=15) and 17 parents (16 mothers) participated in the study. The mean age was 12±4 years (3–18 years) with median disease duration of 21 months (1–172 months), and mean self -concept of 50±8 (36–69). Hollingshead socioeconomic score was 47±11 (26–61). Subjects had the following diagnosis: SJIA (n=5, 26%), dermatomyositis (n=4, 21%), systemic sclerosis/CREST syndrome (n=5, 26%), mixed connective tissue disease (n=2, 11%), Behçet’s disease (n=1, 5%), sarcoidosis (n=1, 5%), and Sjögren’s syndrome (n=1, 5%). Seventeen patients used the English translation and two patients used the Spanish translation. They were of the following ethnicities: White (n=9, 47%), Black (n=3, 16%), Mexican/Latino (n=6, 32%), and Asian (n=1, 5%). The following had major life events (injury/illness-2, change of job-1, unable to pay bills-1, >/=2 events −4). Six children were in preschool-5^th^ grade, 11 from 6^th^ grade –11^th^ grade and 1 was in college. They had the following insurance to cover their standard clinical care: private (n=12, 63%), Medicaid (n=5, 26%), and other (n=1). The subjects were either currently using the following medications or discontinued them: corticosteroids (14/19, 74%), mycophoenolate mofetil (2/19, 11%), cyclosporine (5/19, 26%), cyclophosphamide and/or rituximab (3/19, 16%), hydroxychloroquine (9/19, 47%), azathioprine (1/19), methotrexate (4/19, 21%), and thalidomide (1/19).

Seventeen parents stated that their child had a health condition. Fourteen patients had an emergency room/urgent care visit in the last year. Parents reported a mean of 2.5 ±4, median 2.5 missed work-days in the past 30 days. Parents perceived the impact of child’s illness on daily routine at work (sometimes, often or almost always) in 10/14 cases, and ability to concentrate at work (sometimes, often or almost always) in 12/14 cases). The conditions (other than rheumatic diseases) mentioned by the subjects were: neurocardiogenic syncope (n=1), and celiac disease (n=1).

Child and parent SMILY scores were highly correlated (Spearman rho 0.7, p <0.05, n=17). Child SMILY score correlated with duration illness (Spearman rho =0.4, NS). We examined the HRQOL scores of patients who had ever used disease modifying anti-rheumatic drugs (DMARDS) versus those had never used DMARDS. The mean SMILY scores were 71**±**49 (child, n=15) and 66**±**15 (parent, n=12) for those had had ever used DMARDS. The mean SMILY scores were 49**±**13 (child, n=2) and 53**±**20 (parent) for those had had ever used DMARDS.

### Feasibility

17 child and 15 parent subjects completed the corresponding reports of SMILY©-illness. Subjects completed SMILY©-Illness in ≤10 minutes and scoring each questionnaire took ≤ 10 minutes. For the child report of SMILY©-Illness, 5 items were omitted out of a total of 442 items (26 items × 17 children) with mean number of items omitted =0.3±0.7 (range 0–2). Two children did not complete any forms. For the parent report of SMILY©-Illness, 19 items were omitted out of a total of 390 items (26 items × 15 parents who completed the scale). Mean number of items omitted =1.3±2.4 (range 0–8). Maximum number omitted was 8 items by one parent.

### Means, standard deviations and response range of SMILY©-Illness and other questionnaires

Scores and distribution of SMILY©-Illness, are provided in Table 1. All the reviewers of SMILY©-Illness approved the content, found it to be valid and relevant, easy to understand and especially liked the responses in the form of faces. The questionnaire has face and content validity (Table 2). Due to small sample size we did not perform calculation for psychometric properties.

**Table 1:** **Scale descriptives for child and parent reports of measures of SMILY-Illness**

Questionnaire	Child report	Parent report
SMILY**©-**illness total	69 ± 17 (40–100) (17)	64 ± 16 (40–100) (15)
Effect on self	68 ± 19 (40–100) (17)	64 ± 16 (40–100) (15)
Limitations	67 ± 17 (40–100) (17)	60 ± 17 (40–100) (15)
Social	81 ± 21 (35–100) (17)	77 ± 21 (40–100) (14)
Burden of illness	64 ± 20 (31–100) (17)	61 ± 18 (30–100) (15)
Global HRQOL	80 ± 22 (40–100) (17)	70 ± 20 (40–100) (14)
Global illness status	71 ± 25 (40–100) (17)	70 ± 22 (40–100) (35)

Mean ± SD (range) (number of subjects) is listed above for child and parent reports. SMILY©-Illness scores range between 0–100; *Abbreviations used:* Simple Measure of Impact of Illness in Youngsters©-illness (SMILY©-Illness); SD (standard deviation).

**Table 2:** **Translation and adaptation for cultural suitability of US English SMILY-Illness**

	Language SMILY © was adapted into	Modified by professional translation company and collaborators who made more edits	Number of reviewers for accuracy and cultural suitability and have finally approved the translation
1	Afrikans	Prof trans (1 Peds Rheum)	1Prof trans (1 Peds Rheum)
2	Arabic-Egypt	Prof trans, 3 Peds Rheum	3 (3 Peds Rheum)
3	Arabic-Saudi Arabia	Prof trans, 1 Peds Rheum	1 (1 Peds Rheum)
4	Czech	Prof trans, 1 Peds Rheum	2 (2 Peds Rheum.)
5	Danish	Prof trans, 2 Peds Rheum	2 (2 Peds Rheum.)
6	Dutch	Prof trans	1 (1 Peds Rheum)
7	English-United Kingdom	Adaptation by Peds Nephrologist	1 (Peds Nephrologist)
8	French	Prof trans, 1 Peds Rheum	2 (2 Peds Rheum)
9	German-Austria	Prof trans	2 (2 Peds Rheum)
10	German-Germany	Prof trans 1 Peds Rheum	2 (2 Peds Rheum)
11	German-Swiss	Prof Trans 1 Peds Rheum	2 (2 Peds Rheum)
12	Greek	Prof trans, 1 Peds Rheum	2 (2 Peds Rheum)
13	Hebrew	Prof trans	1 (Peds Rheum)
14	Hindi	Prof trans,	1 (1 Peds Rheum)
15	Hungarian	Prof trans,	1 (1 Peds Rheum)
16	Italian	Prof trans, 1 Peds Rheum	8 (8 Peds Rheum)
17	Japanese	Prof trans,	2 (2 Peds Rheum)
18	Portuguese	Prof trans 6 Peds Rheum	10 (10 Peds Rheum)
19	Romanian	Prof trans, 1 peds Rheum	1 (1 Peds Rheum)
20	Serbia	Prof trans, 1 Peds Rheum	1 (1 Peds Rheum)
21	Slovenia	Prof trans,	1 (1 Peds Rheum)
22	Spanish-Argentina	Prof trans, 1 Peds Rheum	2 (1 Peds Rheum)*
23	Spanish-Mexican	Prof trans, 2 Peds Rheum	2 (2 Peds Rheum)
24	Spanish-Spain	Prof trans, 2 Peds Rheum	2 (2 Peds Rheum)
25	Spanish –US& Puertorico	Prof trans	3 (3 Peds Rheum)*
26	Spanish-Venezuela	Prof trans, 2 Peds Rheum	2 (1 Adult Rheum, 1 Peds Rheum)
27	Turkish	Prof trans, 1 Peds Rheum	3 (3 Peds Rheum)
28	Xhosa	Prof Trans, 1 Peds Rheum nurse	2 (1 Prof trans, 1 Peds Rheum nurse)

*A physician of Argentinian origin, now living in USA, was involved in both versions.

*Abbreviations used* **:** Prof trans-Professional translation company. AP- Assistant Professor of Pediatric Rheumatology, Peds- Pediatrician, Peds Rheum- Pediatric Rheumatologist.

The enclosed translations are in the same order as above.

### Translation process

We had already described the rigorous translation process of SMILEY in previous manuscripts [5, 6]. All the SMILEY translations were adapted to SMILY**©**-Illness using a professional translation company. Collaborative relationships with the different centers across the world were already set up. The review process was similar to the process we followed for SMILEY translations [5, 6]. From each country, pediatric rheumatologists reviewed the translation and approved them for content and cultural appropriateness for their population. Table 2 details the entire adaptation process of 28 languages and all the translations are enclosed at the end of this brief report as Additional file 1.

## Conclusion

SMILY©-Illness is a brief, easy to administer and score HRQOL scale for children with systemic rheumatic diseases. SMILY©-Illness is suitable for use across different age groups and literacy levels. SMILY**©**-Illness has good face and content validity based on its process of adaptation, review by multiple pediatric rheumatologists and initial testing. However, further validation in each country is required for the translated and adapted versions. In our population, a significant percentage of children were on immunosuppressive/immunomodulatory medications. Parents appeared to feel the impact of their child’s illness on a daily basis. The children’s self-concept was only average. The mean total SMILY illness scores were similar in the range of what we found for SMILEY scores in patients with SLE [2]. The lowest scores were found in the domain of “burden of illness” and the highest score indicating better HRQOL was found in the social domain as reported in other studies [2]. As found in the literature, children had higher scores compared to parents [2].

The number of subjects is very small and it would be ideal if the disease types were well distributed. Unfortunately in this sample they are not due to referral bias at the time of the study. Due to the small sample size, we cannot make any definitive conclusions regarding the correlations. Another limitation is that we do not have information regarding the duration of disease prior to diagnosis.

The availability of translations will make recruitment for validation easier since these diseases are rare. SMILY©-Illness with its available translations may be used as useful adjuncts to clinical practice and research, providing valuable insight to the impact of disease on the overall HRQOL of the child.

## Endnote

^a^Robert Wood Johnson Medical School, New Brunswick, NJ; and Hospital for Special Surgery, New York, NY.

## Electronic supplementary material

Additional file 1: **Please see below all the translations of Smily-Illness.** (PDF 3 MB)
