# Supplementary material for: Health related quality of life measure in systemic pediatric rheumatic diseases and its translation to different languages: an international collaboration
Source: Pediatr Rheumatol Online J. 2014 Nov 25;12:49. doi: 10.1186/1546-0096-12-49 (PMC4334772; doi:10.1186/1546-0096-12-49)
Supplement: Supplementary file 1 — Additional file 1: Please see below all the translations of Smily-Illness. (PDF 3 MB) [file 12969_2014_2180_MOESM1_ESM.pdf]

EENVOUDIGE METING VAN UITWERKING VAN SIEKTE IN JONG MENSE (SMILY-Illness) – Ouerverslag

Ondersoeknr. _____ __ Datum: _____/_____/20___

Vir die doeleindes van hierdie vraelys het ons die onderstaande 5 gesiggies van die Wong-Baker FACES pynbeoordelingskaal gebruik, wat met die toestemming van D.L. Wong aangepas is. Hierdie 5 GESIGGIES verskyn onder elke vraag.

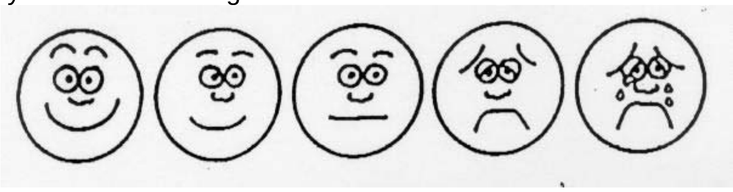

Van Wong DL, Hockenberry-Eaton M, Wilson D, Winklestein ML, Schwartz P.: Wong's Essentials of Pediatric Nursing, ed. 6, St. Louis, 2001, Mosby, p 1301. Kopiereg Mosby. Met toestemming herdruk.

Die SMILY-Illness is onder kopiereg van L. Nandini Moorthy, MD MS, RWJMS-UMDNJ, New Brunswick, NJ. Op hierdie stadium mag hierdie vraelys slegs deur L. Nandini Moorthy, MD MS en haar toegewese navorsingspersoneel gebruik word.

VRAE

In hierdie vraelys stel ons baie vrae oor u kind se siekte en sy/haar gevoelens daaroor. Daar is geen verkeerde antwoorde nie. Vra asb. ons hulp indien daar enigiets is wat u nie verstaan nie. Indien u enige kommentaar wil neerskryf, doen dit asseblief op die keersy van bladsye 5 en 6.

Verskeie goed affekteer u kind se lewe en maak u kind gelukkig of ongelukkig. Daar is sekere goed wat vir u kind belangrik is, en dit sluit dinge in wat u kind kan doen (of nie kan doen nie). Saam vorm al hierdie dinge u kind se lewensgehalte.

1. Hoe is u kind se lewensgehalte? Trek asseblief 'n kring om die gesiggie wat u kind se lewensgehalte die beste uitbeeld.

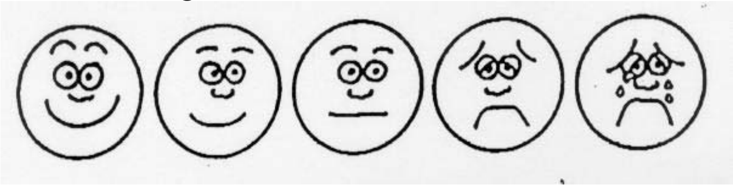

2. Hoe is die toestand van u kind se siekte op die oomblik? Trek asseblief 'n kring om die gesiggie wat die huidige toestand van u kind se *siekte* die beste uitbeeld.

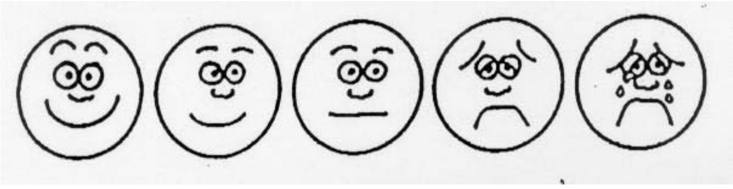

Vrae 3-19 handel oor hoe u kind die afgelope maand gevoel het weens sy/haar *siekte*. Trek asseblief'n kring om die gesiggie wat u kind se gevoelens die beste uitbeeld.

3. Hoe voel u kind daaroor dat hy/sy 'n siekte het?

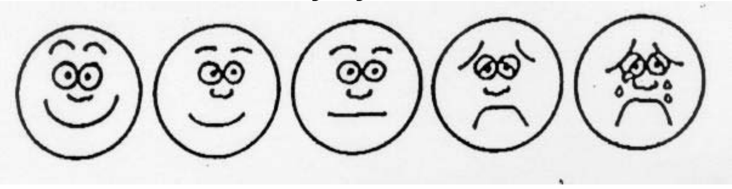

4. Hoe laat 'n siekte u kind voel oor skool-toe gaan?

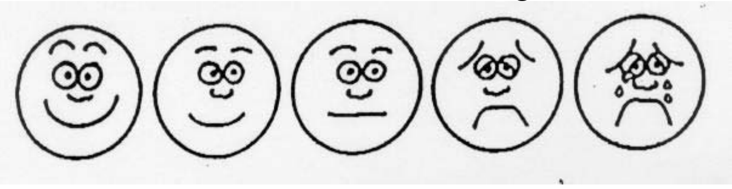

5. Hoe laat 'n siekte u kind voel oor elke dag se klere-aantrek?

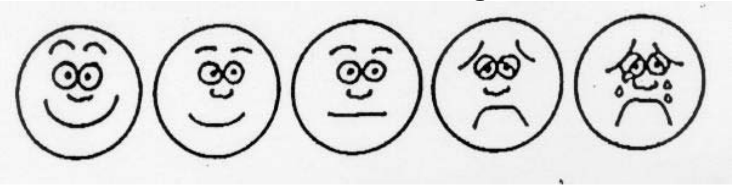

6. Hoe laat 'n siekte u kind voel oor sy/haar voorkoms (hoe hy/sy lyk)?

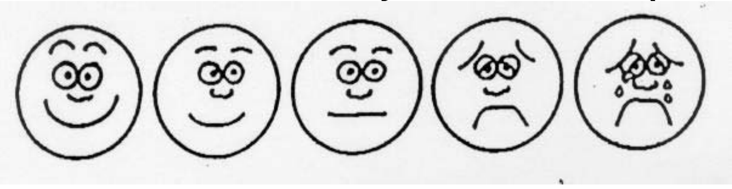

7. Hoe laat 'n siekte u kind oor homself/haarself voel?

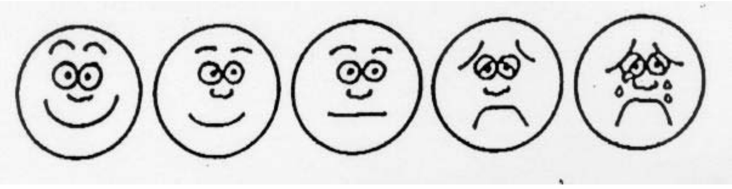

8. Hoe voel u kind wanneer hy/sy oor sy/haar siekte moet praat?

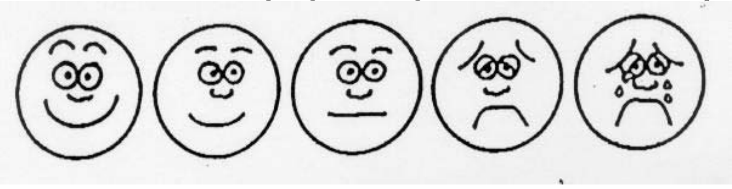

9. Hoe voel u kind daaroor as hy/sy weens sy/haar siekte dokter toe moet gaan?

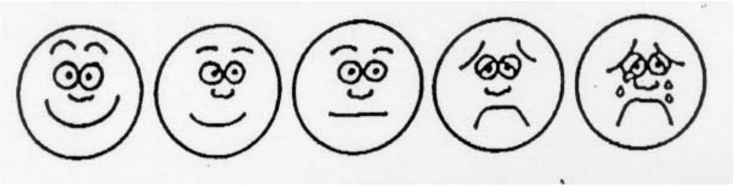

10. Hoe voel u kind daaroor as hy/sy weens sy/haar siekte hospitaal toe moet gaan?

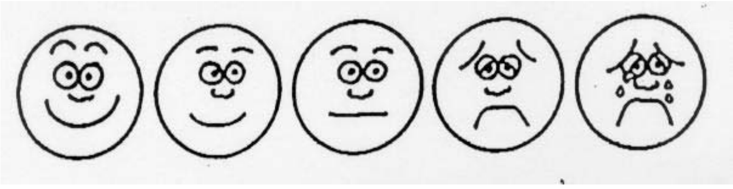

11. Hoe laat ’n siekte u kind voel oor sy/haar toekoms?

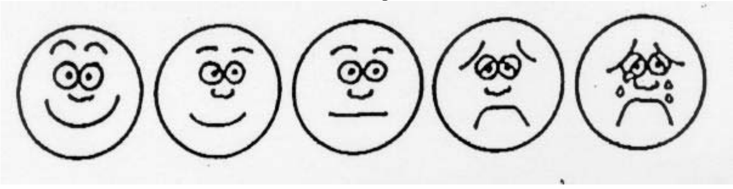

12. Hoe voel u kind daaroor dat hy/sy medikasie vir sy/haar siekte moet neem?

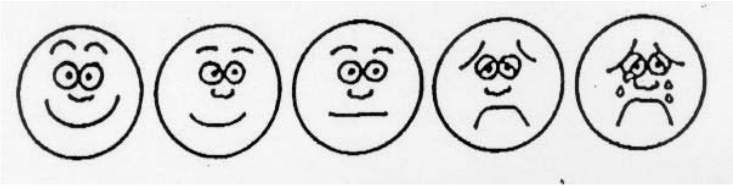

13. Hoe bekommerd is u kind oor die newe-effekte van die medikasie wat hy/sy vir sy/haar siekte moet neem?

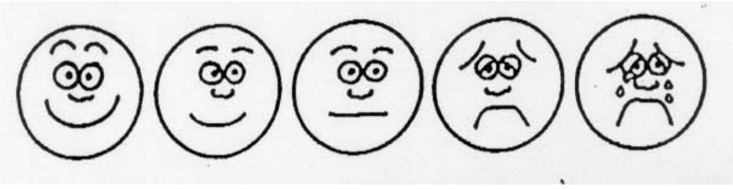

14. Hoe bekommerd is u kind dat sy/haar siekte kan vererger?

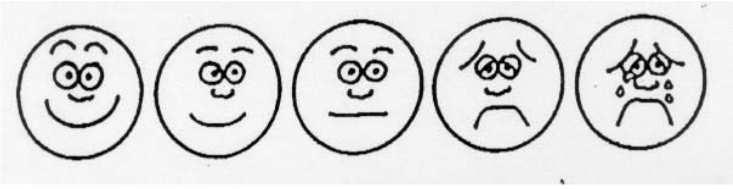

15. Hoe eensaam voel u kind as gevolg van sy/haar siekte ?

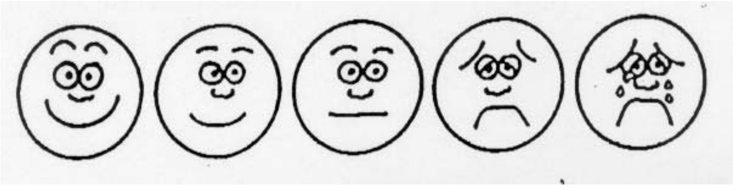

16. Hoe laat u kind se gesin (familie) hom/haar voel?

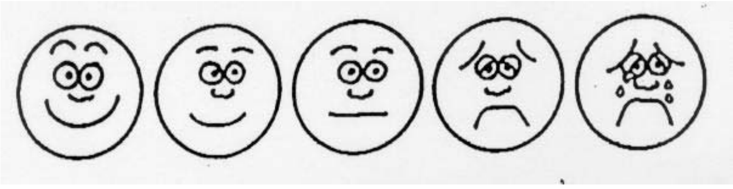

17. Hoe laat die pyn wat met sy/haar siekte gepaard gaan, u kind voel?

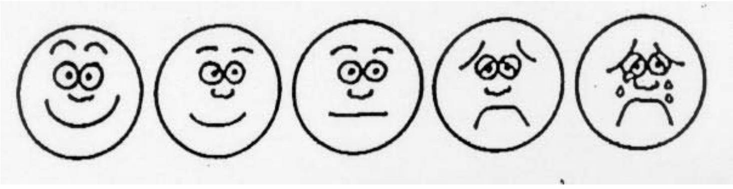

18. Hoe voel u kind daaroor dat hy/sy weens sy/haar siekte nie buite in die son mag wees nie?

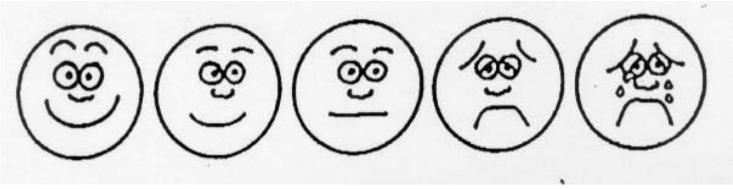

19. Hoe moeg laat sy/haar siekte u kind voel?

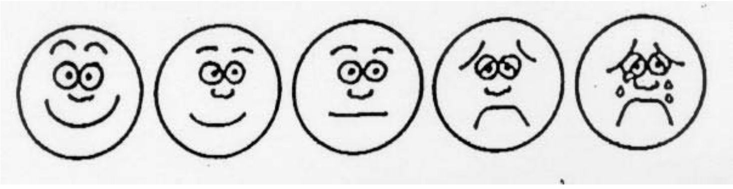

Beantwoord asb. vrae 20 A – 26 A met "ja" of "nee". Volg dan die instruksies wat volg.

20 A. U kind se vriende het as gevolg van sy/haar siekte verander. Trek asb. 'n kring om "ja" of "nee." Ja Nee

Indien u "ja" geantwoord het, beantwoord asb. ook vraag 20 B.

20 B. Hoe laat sy/haar *siekte* u kind oor sy/haar vriende voel? Trek asb. 'n kring om die gesiggie wat aandui hoe u kind voel.

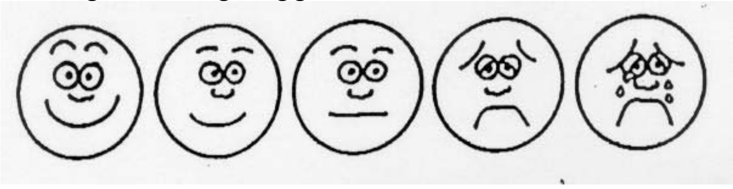

21 A. U kind se vriende verstaan dat hy/sy 'n siekte het. Ja Nee

Indien u "nee" geantwoord het, beantwoord asb. ook vraag 21 B.

21 B. Hoe voel u kind daaroor dat sy/haar vriende nie verstaan dat hy/sy 'n siekte het nie? Trek asb. 'n kring om die gesiggie wat aandui hoe u kind voel.

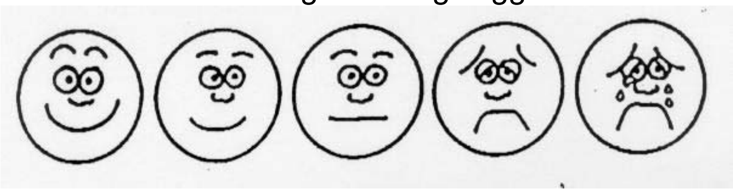

22 A. 'n Siekte affekteer u kind se skoolwerk. Ja Nee

Indien u "ja" geantwoord het, beantwoord asb. ook vraag 22 B.

22 B. Hoe laat u kind se *siekte* hom/haar voel oor sy/haar skoolwerk? Trek asb. 'n kring om die gesiggie wat aandui hoe u kind voel.

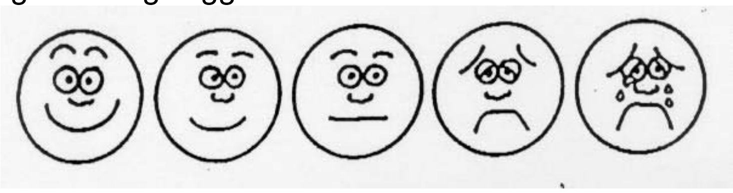

23 A. Weens sy/haar siekte kan u kind soms nie skool-toe gaan nie. Ja Nee

Indien u "ja" geantwoord het, beantwoord asb. ook 23 B.

23 B. Hoe voel u kind as hy/sy weens sy/haar siekte nie skool-toe kan gaan nie?
Trek asb. ’n kring om die gesiggie wat aandui hoe u kind voel.

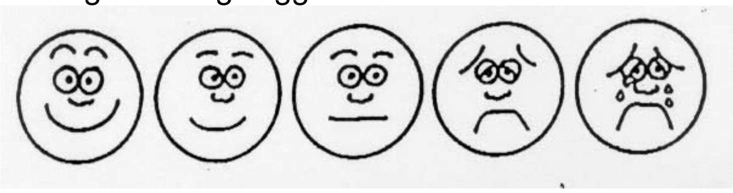

24 A. Die siekte affekteer u kind se náskoolse bedrywighede. Ja Nee

Indien u “ja” geantwoord het, beantwoord asb. ook vraag 24 B.

24 B. Hoe voel u kind daaroor dat sy/haar siekte sy/haar náskoolse bedrywighede affekteer? Trek asb. ’n kring om die gesiggie wat aandui hoe u kind voel.

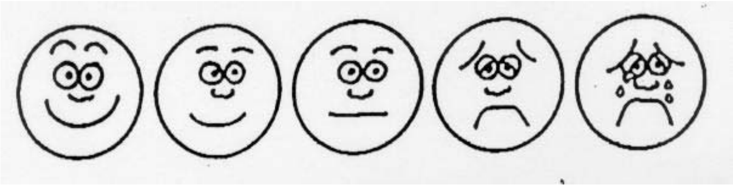

25 A. Die siekte affekteer die pret wat u kind met sy/haar vriende het. Ja Nee

Indien u “ja” geantwoord het, beantwoord asb. ook vraag 25 B.

25 B. Hoe voel u kind daaroor dat sy/haar siekte die pret wat hy/sy met sy/haar vriende het, affekteer? Trek asb. ’n kring om die gesiggie wat aandui hoe u kind voel.

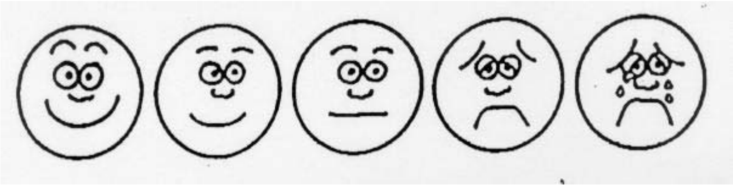

26 A. Die siekte affekteer u kind se deelname aan liggaamlike bedrywighede (soos liggaamsoefeninge by die skool, hardloop, stap, ’n gunsteling-speletjie in die buitelug). Ja Nee

Indien u “ja” geantwoord het, beantwoord asb. ook vraag 26 B.

26 B. Hoe voel u kind daaroor dat sy/haar siekte sy/haar deelname aan fisiese aktiwiteite affekteer (soos liggaamsoefeninge by die skool, hardloop, stap, gunsteling- speletjies in die buitelug)? Trek asb. ’n kring om die gesiggie wat aandui hoe u kind voel.

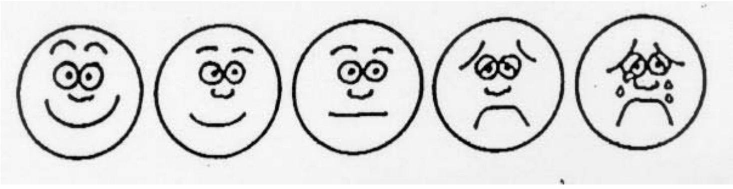

EENVOUDIGE METING VAN UITWERKING VAN SIEKTE IN JONG MENSE (SMILY-Illness) – Kinderverslag

Ondersoeknr. ________ Datum: _____/_____/20_____

Vir die doeleindes van hierdie vraelys het ons die onderstaande 5 gesiggies van die Wong-Baker FACES pynbeoordelingskaal gebruik, wat met die toestemming van D.L. Wong aangepas is. Hierdie 5 GESIGGIES sal onder elke vraag verskyn.

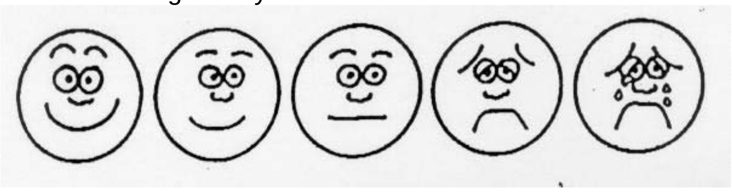

Van Wong DL, Hockenberry-Eaton M, Wilson D, Winklestein ML, Schwartz P.: Wong's Essentials of Pediatric Nursing, ed. 6, St. Louis, 2001, Mosby, p 1301. Kopiereg Mosby. Met toestemming herdruk.

Die SMILY-illness is onder kopiereg van L. Nandini Moorthy, MD MS, RWJMS-UMDNJ, New Brunswick, NJ. Op hierdie stadium mag hierdie vraelys slegs deur L. Nandini Moorthy, MD MS en haar toegewese navorsingspersoneel gebruik word.

VRAE

In hierdie vraelys stel ons baie vrae oor jou siekte en hoe jy daaroor voel. Daar is geen verkeerde antwoorde nie. Vra asb. ons hulp as daar enigiets is wat jy nie mooi verstaan nie. As jy enige kommentaar wil neerskryf, doen dit asb. op die keersy van bladsye 5 en 6.

Verskeie dinge affekteer jou lewe en maak jou gelukkig of ongelukkig. Daar is sekere dinge wat vir jou belangrik is en dit sluit dinge in wat jy kan doen (of wat jy nie kan doen nie). Saam maak al hierdie verskillende dinge jou lewensgehalte uit.

1. **Hoe is jou lewensgehalte? Trek asb. 'n kring om die gesiggie wat jou lewensgehalte die beste wys.**

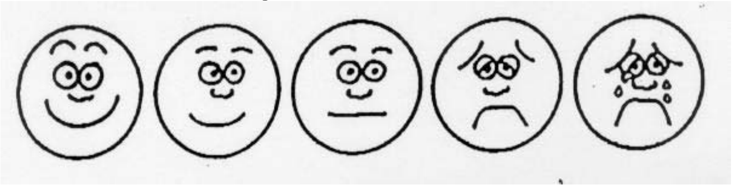

2. **Hoe gaan dit met jou siekte op hierdie oomblik? Trek asb. 'n kring om die gesiggie wat nou jou siekte die beste wys.**

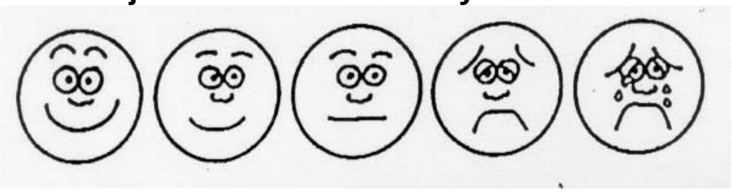

Kopiereg © 2007 Lakshmi Nandini Moorthy. Alle regte voorbehou.

Vrae 3-19 handel oor hoe jy die afgelope maand gevoel het weens jou *siekte*. Trek asb. 'n kring om die gesiggie wat die beste wys hoe jy voel.

3. Hoe laat dit jou voel omdat jy 'n siekte het?

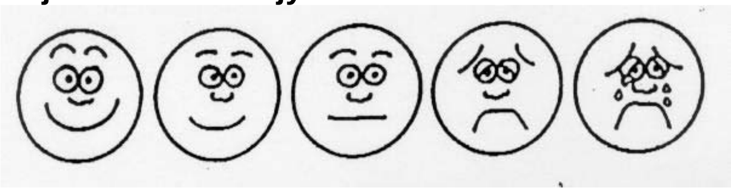

4. Hoe laat 'n siekte jou voel oor skool-toe gaan?

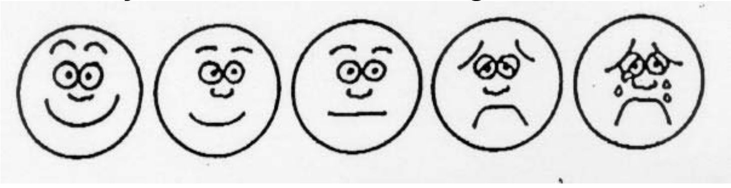

5. Hoe laat 'n siekte jou voel oor jy elke dag klere moet aantrek?

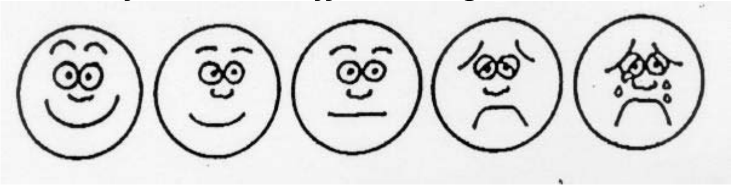

6. Hoe laat 'n siekte jou voel oor hoe jy lyk?

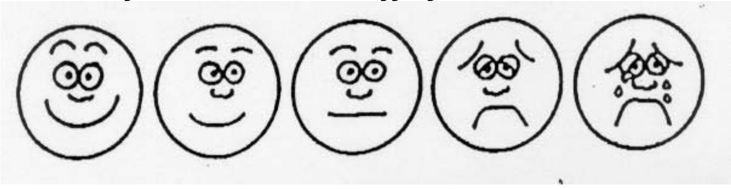

7. Hoe laat 'n siekte jou oor jouself voel?

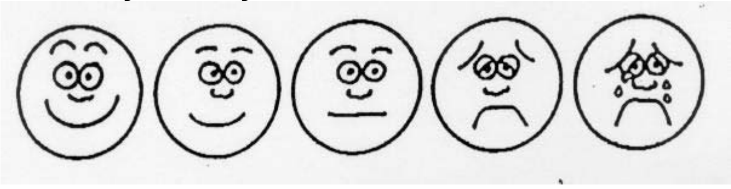

8. Hoe voel jy daaroor as jy oor jou siekte moet praat?

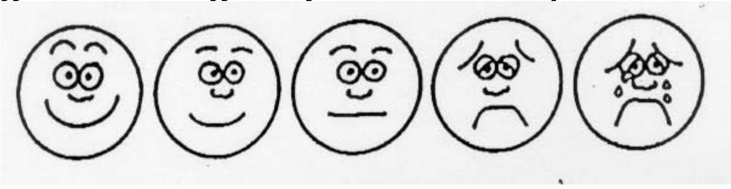

Kopiereg © 2007 Lakshmi Nandini Moorthy. Alle regte voorbehou.

9. Hoe voel jy daaroor as jy weens 'n siekte dokter toe moet gaan?

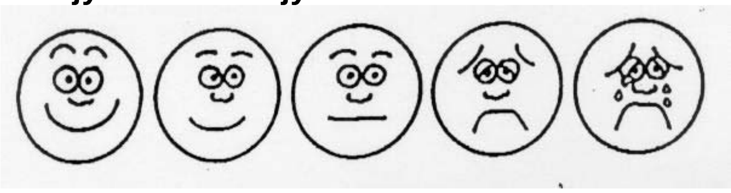

10. Hoe voel jy daaroor as jy weens 'n siekte hospitaal toe moet gaan?

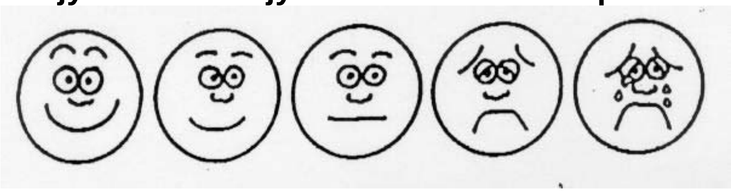

11. Hoe laat 'n siekte jou oor jou toekoms voel?

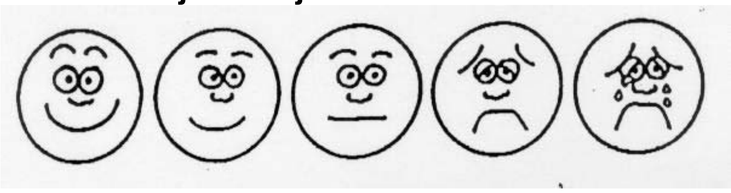

12. Hoe voel jy omdat jy medikasie vir jou siekte moet neem?

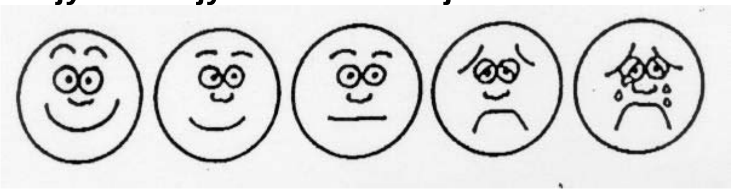

13. Hoe bekommerd is jy oor die newe-effekte van die medikasie wat jy vir jou siekte neem?

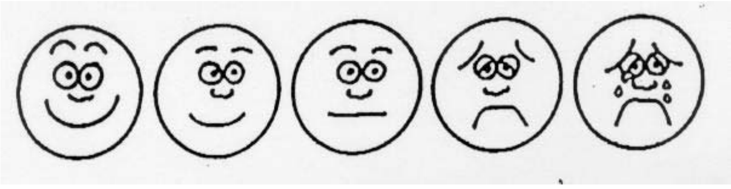

Kopiereg © 2007 Lakshmi Nandini Moorthy. Alle regte voorbehou.

14. **Hoe bekommerd is jy dat jou siekte kan vererger?**

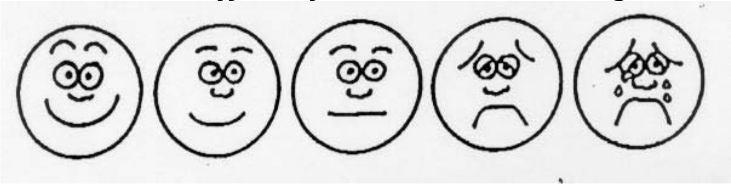

15. **Hoe eensaam voel jy omdat jy jou siekte het?**

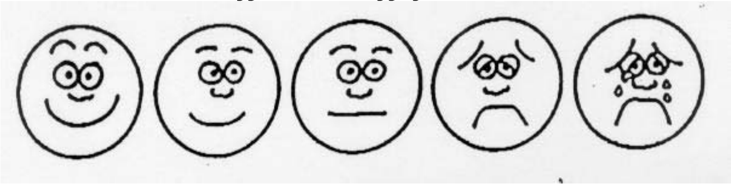

16. **Hoe laat jou gesin (familie) jou voel?**

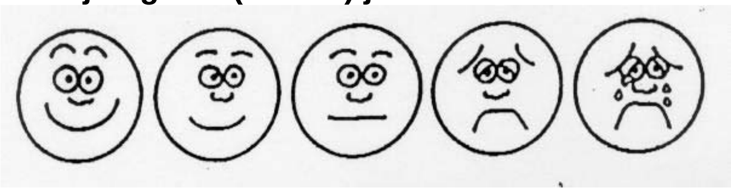

17. **Hoe laat die pyn van jou siekte jou voel?**

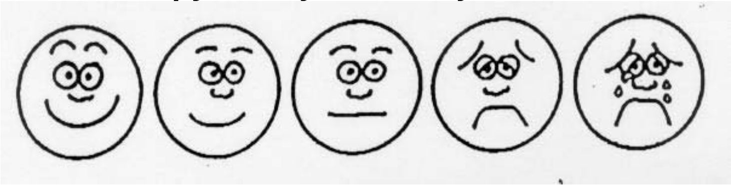

18. **Hoe voel jy daaroor dat jy weens jou siekte nie buite in die son kan wees nie?**

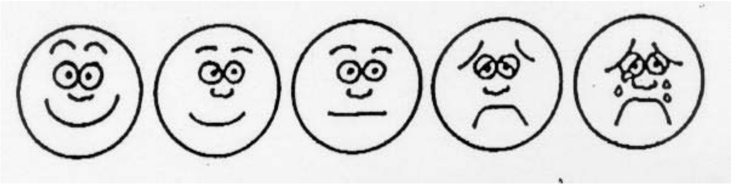

19. **Hoe moeg laat jou siekte jou voel?**

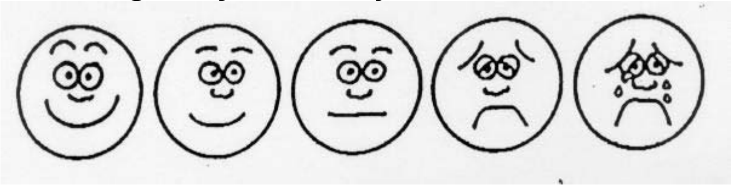

Kopiereg © 2007 Lakshmi Nandini Moorthy. Alle regte voorbehou.

Antwoord "ja" of "nee" op vrae 20A – 26A. Volg dan asb. die instruksies wat volg.

20 A. My vriende het verander as gevolg van my siekte. Trek asb'n kring om "ja" of "nee". Ja Nee

As jy "ja" geantwoord het, gaan na vraag 20B.

20 B. Hoe laat jou siekte jou oor jou vriende voel? Trek asb'n kring om die gesiggie wat jou gevoelens wys.

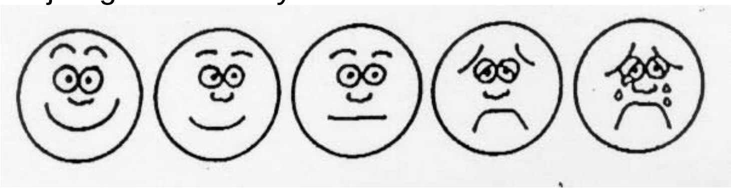

21 A. My vriende verstaan dat ek 'n siekte het. Ja Nee

As jy "nee" geantwoord het, gaan na vraag 21B.

21 B. Hoe voel jy daaroor dat jou vriende nie verstaan dat jy'n siekte het nie? Trek asb. 'n kring om die gesiggie wat wys hoe jy voel.

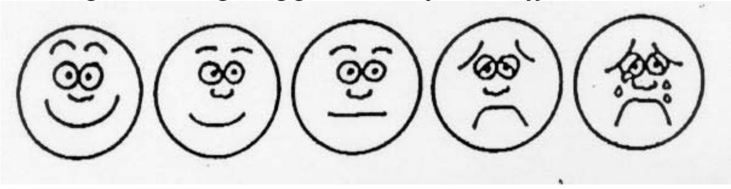

22 A. My siekte affekteer my skoolwerk. Ja Nee

As jy "ja" geantwoord het, gaan na vraag 22B.

22 B. Hoe laat 'n siekte jou voel oor jou skoolwerk? Trek asb.'n kring om die gesiggie wat wys hoe jy voel.

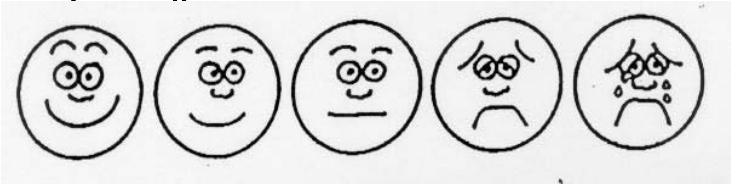

23 A. Weens my siekte kan ek nie skool-toe gaan nie. Ja Nee

As jy "ja" geantwoord het, gaan na vraag 23B.

23 B. Hoe voel jy as jy weens jou siekte nie skool-toe kan gaan nie? Trek asb. 'n kring om die gesiggie wat wys hoe jy voel.

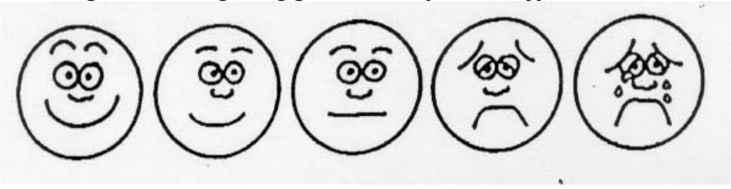

Kopiereg © 2007 Lakshmi Nandini Moorthy. Alle regte voorbehou.

24 A. My siekte affekteer my aktiwiteite wat ek buite die skool doen.

Ja Nee

As jy "ja" geantwoord het, gaan na vraag 24B.

24 B. Hoe voel jy daaroor dat jou siekte jou aktiwiteite buite die skool affekteer? Trek asb. 'n kring om die gesiggie wat wys hoe jy voel.

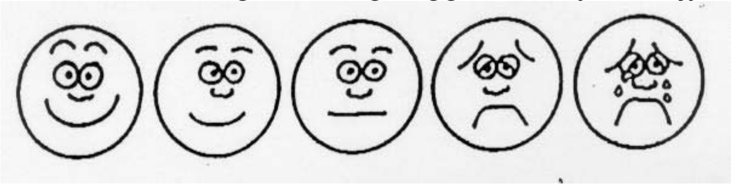

25 A. My siekte affekteer die pret wat ek saam met my vriende het.

Ja Nee

As jy "ja" geantwoord het, gaan na vraag 25B.

25 B. Hoe voel jy daaroor dat jou siekte die pret affekteer wat jy saam met jou vriende het? Trek asb. 'n kring om die gesiggie wat wys hoe jy voel.

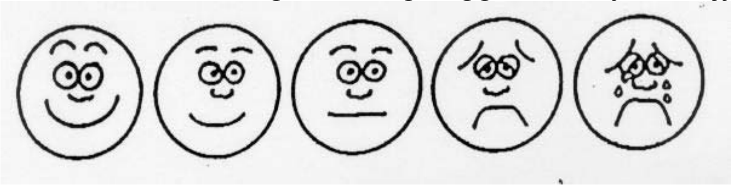

26 A. My siekte affekteer my deelname aan fisiese aktiwiteite (soos gim-aktiwiteite by die skool, hardloop, stap,'n gunsteling -speletjie in die buitelug speel). Ja Nee

As jy "ja" geantwoord het, gaan na vraag 26B.

26 B. Hoe voel jy daaroor dat jou siekte jou deelname aan fisiese aktiwiteite (soos gim-aktiwiteite by die skool, hardloop, stap,'n gunsteling -speletjie in die buitelug speel) affekteer? Trek asb.'n kring om die gesiggie wat wys hoe jy voel.

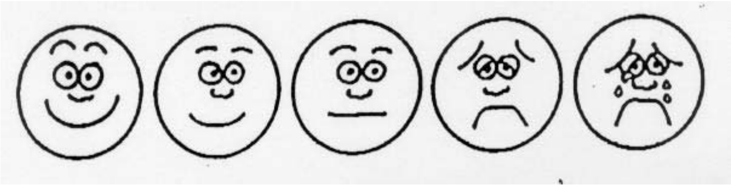

Kopiereg © 2007 Lakshmi Nandini Moorthy. Alle regte voorbehou.

المقياس البسيط لتقييم أثر مرض الأطفال
(SMILY-Illness) – نسخة الأبوين (Parent Survey)

حالة رقم __ تاريخ __/__/20_

من اجل هذا الاستبيان أخذنا الصور الخمسة الآتية من أوجه وونغ -بيكر لتقييم الألم، وقمنا بإدخال تغييرات عليها بموافقة د. ل. وونغ. سوف تظهر هذه الأشكال الخمسة تحت كل سؤال.

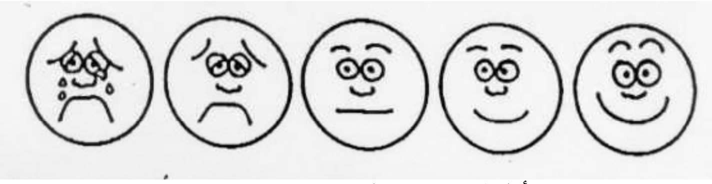

من المؤلف وونغ د.ل ـ الأساسيات في مرض الأطفال. نشر بمطابع موسبي عام 2001. صفحة 1301. حقوق الطبع محفوظة

حقوق نشر SMILY-illness محفوظة للدكتورة نانديني مورثي، طبيبة وحائزة على ماجستير جراحة، مدرسة طب روبرت وود جونسون، جامعة الطب وطب الأسنان في نيوجيرسي، . نيوبرونسويك. ولاية نيوجرسي، يمكن استخدام الاستبيان في الوقت الحاضر فقط من خلال الدكتورة الأخصائية نانديني مورثي وفريق الباحثين العامل معها.

الأسئلة

هذا الاستبيان يحتوي على عدة أسئلة عن مرض طفلك وشعوره نحوها. اختر ما تراه مناسب فلا توجد أجوبة خاطئة. إذا كان لديك أي سؤال فلا تتردد بطرحه علينا وان كان لديك أي تعليق فيمكنك تدوينه على ظهر الصفحتين 5 و 6.

هناك أمور تؤثر في حيات طفلك وتجعله يشعر بالسرور أو الحزن وتوجد بعض الأمور المهمة التي بمقدوره القيام بها (وربما لا يتمكن من إتمامها). هذه الأمور مجتمعه تحدد نوعية وجودة حياة طفلك.

السؤال الأول كيف تقيم جودة حياة طفلك؟ من فضلك ضع دائرة حول الوجه الذي يعكس جودة حياته.

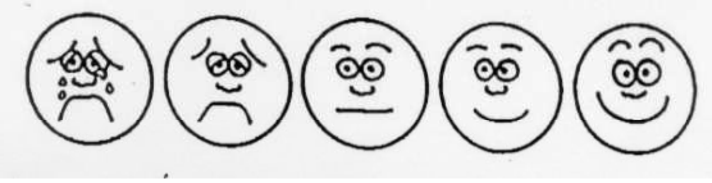

السؤال الثاني ما هي حالة طفلك الآن مع المرض؟ من فضلك ضع دائرة حول الوجه الذي يظهر وصف حالة طفلك ألان

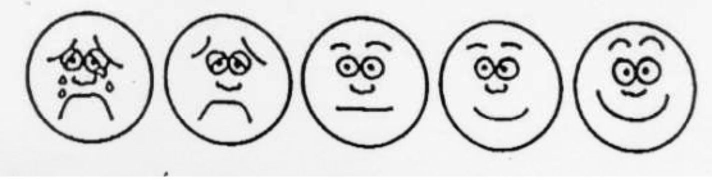

حقوق النشر 2007 © محفوظة للدكتورة نانديني مورثي. جميع الحقوق محفوظة.

الأسئلة 3-19 عن كيفية شعور طفلك خلال الشهر الماضي بسبب المرض. من فضلك ضع دائرة حول الوجه الذي يصف شعور طفلك

السؤال الثالث **ما هو شعور طفلك تجاه كونه مصاب بهذا المرض؟**

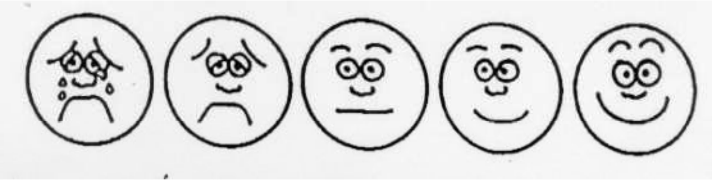

السؤال الرابع **كيف يؤثر المرض على شعور طفلك تجاه ذهابه إلى المدرسة؟**

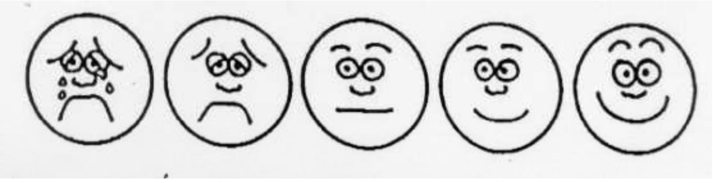

السؤال الخامس **كيف يؤثر المرض على شعور طفلك تجاه ارتدائه ملابسه كل يوم؟**

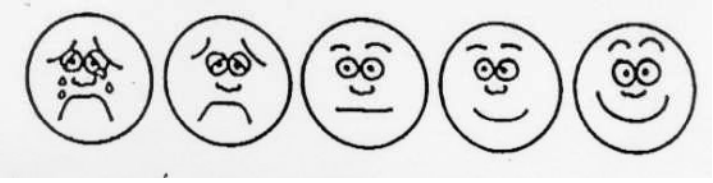

السؤال السادس **كيف يؤثر المرض على شعور طفلك تجاه مظهره؟**

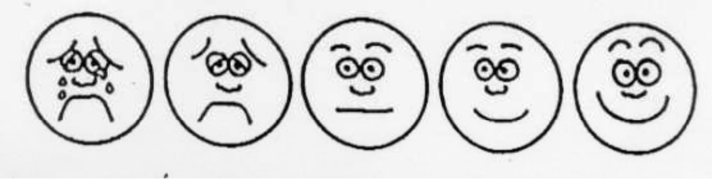

السؤال السابع **كيف يجعل المرض طفلك يشعر تجاه نفسه؟**

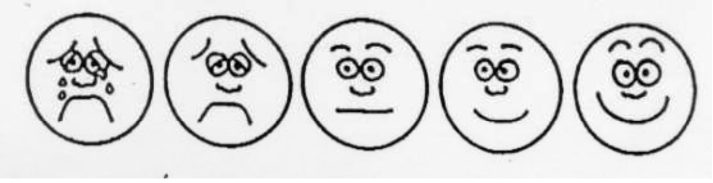

السؤال الثامن **كيف يشعر طفلك عندما يريد التحدث عن المرض؟**

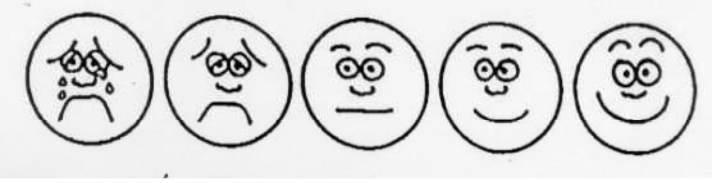

حقوق النشر 2007 © محفوظة للدكتورة نانديني مورثي. جميع الحقوق محفوظة.

السؤال التاسع **كيف يشعر طفلك تجاه الذهاب إلى الطبيب بسبب هذا المرض؟**

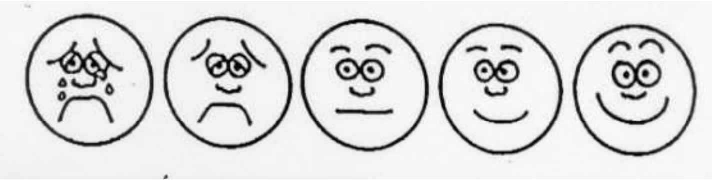

السؤال العاشر **كيف يشعر طفلك تجاه الذهاب إلى المستشفى بسبب هذا المرض؟**

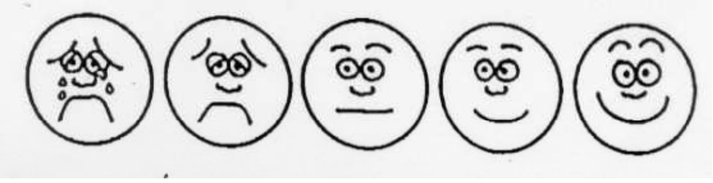

السؤال الحادي عشر **كيف يؤثر المرض على شعور طفلك تجاه مستقبله؟**

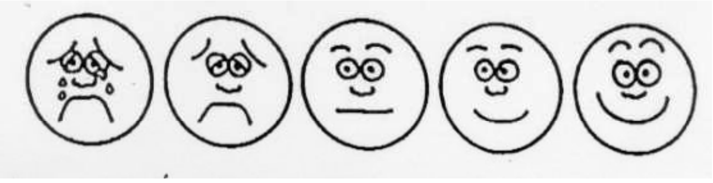

السؤال الثاني عشر **كيف يشعر طفلك تجاه أخذ الأدوية الخاصة بالمرض؟**

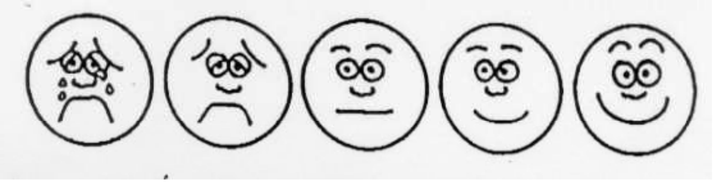

السؤال الثالث عشر **ما مدى قلق طفلك بسبب الآثار الجانبية لأدوية المرض؟**

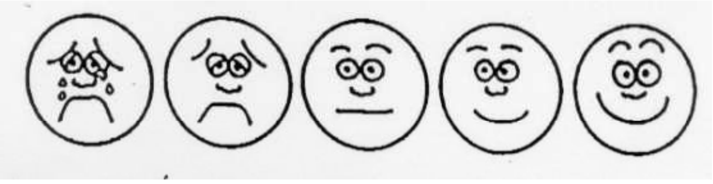

السؤال الرابع عشر **ما مدى قلق طفلك خشية أن تسوء حالته المرضية؟**

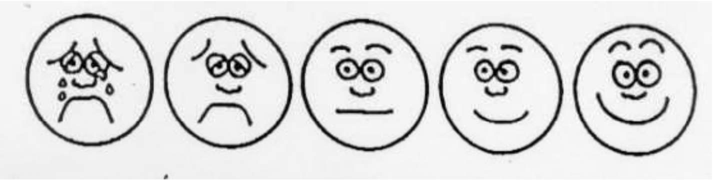

حقوق النشر 2007 © محفوظة للدكتورة نانديني مورثي. جميع الحقوق محفوظة.

السؤال الخامس عشر   ما مدى شعور طفلك بالوحدة بسبب المرض؟

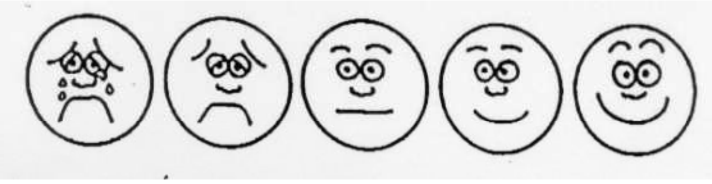

السؤال السادس عشر   ما هو تأثير العائلة على شعور الطفل؟

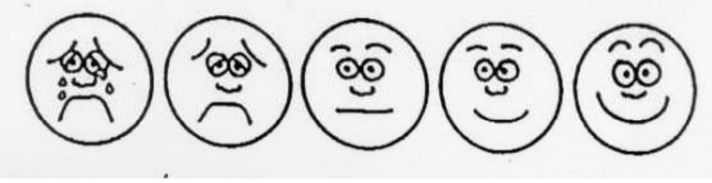

السؤال السابع عشر   ما هو تأثير ألم المرض على شعور طفلك؟

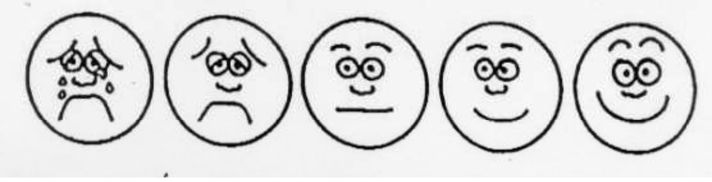

السؤال الثامن عشر   كيف يشعر طفلك تجاه عدم قدرته الخروج في الشمس بسبب المرض؟

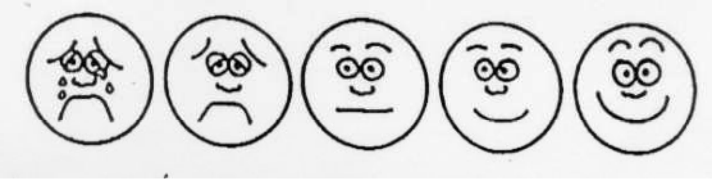

السؤال التاسع عشر   إلى أي مدى يشعر طفلك بالتعب والإجهاد بسبب المرض؟

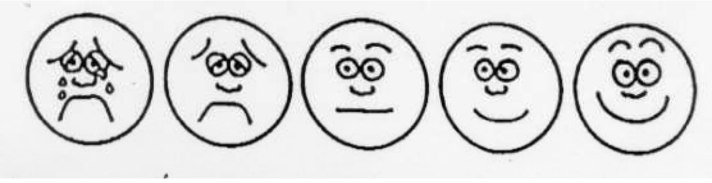

حقوق النشر 2007 © محفوظة للدكتورة نانديني مورثي. جميع الحقوق محفوظة.

من السؤال العشرين أ إلى السؤال السادس والعشرين أ، أجب بـ " نعم" أو "لا"، ثم اتبع التعليمات التالية

السؤال العشرون أ **تغيرت علاقة أصدقائه معه بسبب مرضه.** **"نعم"** **"لا"**

إذا كان جوابك " نعم"، " اذهب إلى سؤال 20ب.

"20ب" كيف يجعل المرض طفلك يشعر تجاه أصدقائه

من فضلك ضع دائرة حول الوجه المناسب.

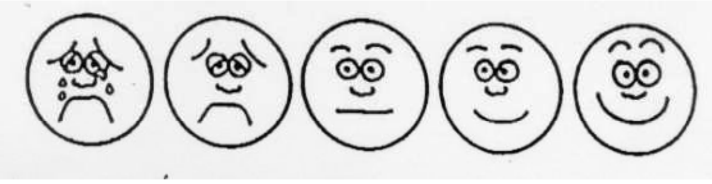

السؤال الحادي والعشرون أ **يتفهم أصدقاء طفلك مرضه** **"نعم"** **"لا"**

إذا أجبت بـ "لا" اذهب إلى سؤال 21ب.

21ب: كيف يشعر طفلك تجاه عدم تفهم أصدقائه مرضه؟

من فضلك ضع دائرة حول الوجه الذي يصف شعور طفلك.

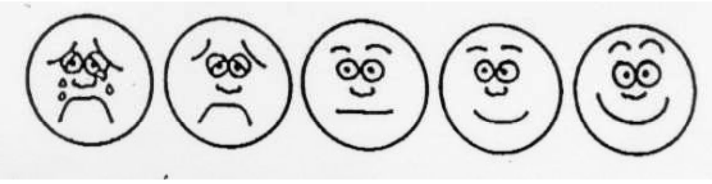

السؤال الثاني وعشرون أ **هل يؤثر المرض علي أداء طفلك بالمدرسة ؟** **"نعم"** **أم "لا"**

إذا أجبت بـ "نعم" اذهب إلى سؤال 22ب

22ب:. كيف يؤثر المرض على شعور طفلك تجاه أدائه بالمدرسة؟

من فضلك ضع دائرة حول الوجه الذي يصف شعور طفلك.

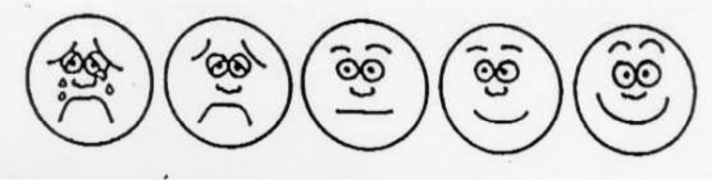

السؤال الثالث وعشرون أ **يغيب طفلك عن المدرسة بسبب مرضه** **"نعم"** **أم "لا"**

إذا أجبت بـ "نعم" اذهب إلى سؤال 23ب.

23ب:.كيف يشعر طفلك تجاه تغيبه عن المدرسة بسبب المرض؟

من فضلك ضع دائرة حول الوجه الذي يصف شعور طفلك.

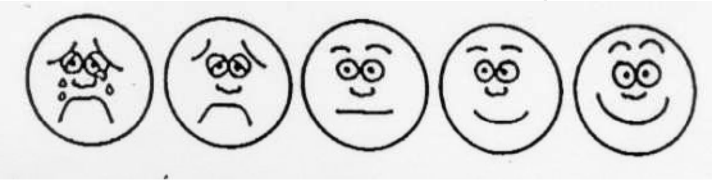

حقوق النشر 2007 © محفوظة للدكتورة ناديني مورثي. جميع الحقوق محفوظة.

السؤال الرابع وعشرون أ **يؤثر المرض علي نشاطات طفلك خارج المدرسة** **"نعم** **أم "لا"**

إذا أجبت "نعم" اذهب إلى سؤال ب24.

24ب كيف يشعر طفلك تجاه تأثير المرض على نشاطاته خارج المدرسة؟

من فضلك ضع دائرة حول الوجه الذي يصف شعور طفلك

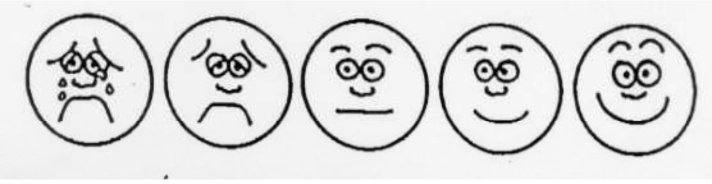

السؤال الخامس وعشرون أ **يؤثر المرض على وقت المرح الذي يقضيه طفلك مع أصدقائه؟** **"نعم"** **أم "لا"**

إذا أجبت بـ "نعم" اذهب إلى سؤال 25ب

25ب كيف يشعر طفلك تجاه تأثير المرض على أوقات المرح الذي يقضيه مع أصدقائه

من فضلك ضع دائرة حول الوجه الذي يصف شعور طفلك

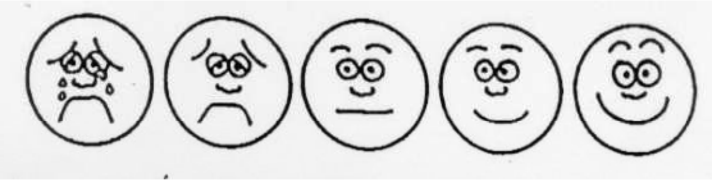

السؤال السادس وعشرون أ **يؤثر المرض علي اشتراك طفلك في النشاطات البدنية (مثل نشاطات النادي الرياضي في المدرسة والجري، والمشي، ولعب أي من الألعاب الخارجية المفضلة)**

"نعم" **"لا"**

إذا أجبت بـ "نعم" اذهب إلى سؤال 26ب

26ب. كيف يشعر طفلك تجاه تأثير المرض على اشتراكه في النشاطات البدنية (مثل نشاطات النادي الرياضي في المدرسة و الجري، المشي، لعب أي من الألعاب الخارجية المفضلة)؟

من فضلك ضع دائرة حول الوجه الذي يصف شعور طفلك.

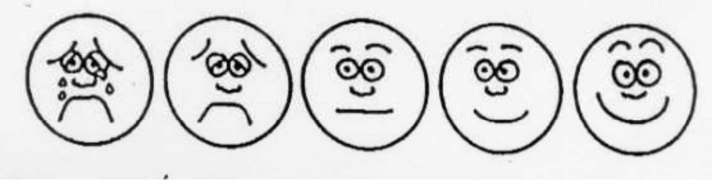

أعدت الترجمة إلى العربية مع المحافظة على بنية السؤال بطابعه الانكليزي. دعاء عبد الرحيم

قام بالمراجعة والتصحيح د.سليمان المعيوف. مستشفى الملك فيصل التخصصي ومركز الأبحاث بالرياض. المملكة العربية السعودية

حقوق النشر 2007 © محفوظة للدكتورة نانديني مورثي. جميع الحقوق محفوظة.

معيار بسيط لتقييم أثر المرض لدى الأطفال (SMILY-illness)
نسخة الطفل (Child Survey)

حالة رقم __ تاريخ __/__/_20

من اجل هذا الاستبيان أخذنا الصور الخمسة الآتية من أوجه "وونغ بيكر" لتقييم الألم، وقمنا بإدخال تغييرات عليها بموافقة د. ل. وونغ. سوف تظهر هذه الأشكال الخمسة تحت كل سؤال

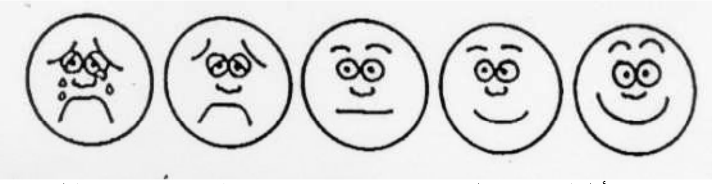

من المؤلف وونغ د.ل ـ الأساسيات في مرض الأطفال. نشر بمطابع موسبي عام 2001. صفحة 1301. حقوق الطبع محفوظة

حقوق نشر SMILY-illness محفوظة للدكتورة نانديني مورثي، طبيبة وحائزة على ماجستير جراحة، مدرسة طب روبرت وود جونسون، جامعة الطب وطب الأسنان في نيوجيرسي، . نيوبرونسويك. ولاية نيوجرسي، يمكن استخدام الاستبيان في الوقت الحاضر فقط من خلال الدكتورة الأخصائية نانديني مورثي وفريق الباحثين العامل معها

الأسئلة

هذا الاستبيان يحتوي على عدة أسئلة عن مرضك وشعورك نحوها. اختر ما تراه مناسب فلا توجد أجوبة خاطئة. إذا كان لديك أي سؤال فلا تتردد بطرحه علينا وان كان لديك أي تعليق فيمكنك تدوينه على ظهر الصفحتين 5 و 6.

هناك أمور تؤثر في حياتك وتجعلك تشعر بالسرور أو الحزن
وتوجد بعض الأمور المهمة التي بمقدورك القيام بها (وربما لا تتمكن من إتمامها).
هذه الأمور مجتمعه تحدد نوعية وجودة حياتك.

السؤال الأول **كيف تقيم نوعية حياتك؟ من فضلك ضع دائرة حول الوجه الذي يظهر درجة نوعية حياتك**

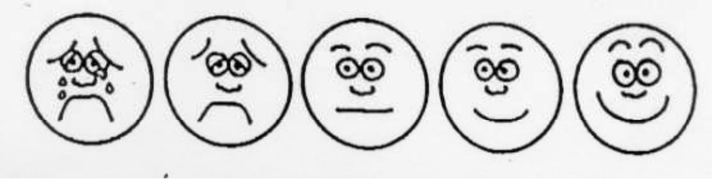

السؤال الثاني **كيف هي حالة المرض الآن؟ من فضلك ضع دائرة حول الوجه الذي يظهر وصف لحالتك الآن**

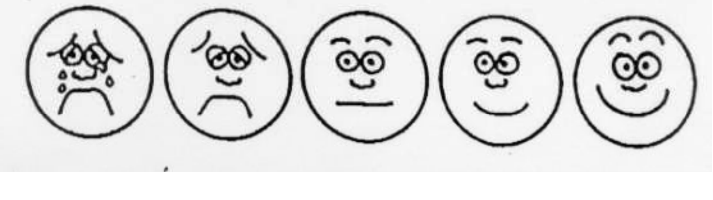

حقوق النشر 2007 © محفوظة للدكتورة نانديني مورثي. جميع الحقوق محفوظة.

تعبر الأسئلة 3-19 عن كيفية شعورك خلال الشهر الماضي بسبب المرض. من فضلك ضع دائرة حول الوجه الذي يعبّر بشكل أفضل عن شعورك.

السؤال الثالث **كيف يؤثر المرض على شعورك؟**

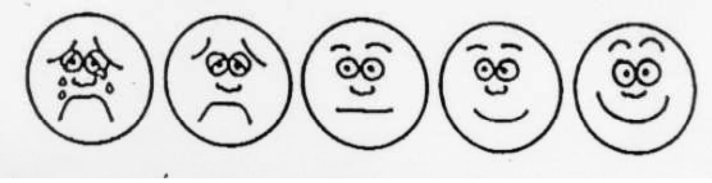

السؤال الرابع **كيف يؤثر المرض على شعورك تجاه ذهابك إلي المدرسة؟**

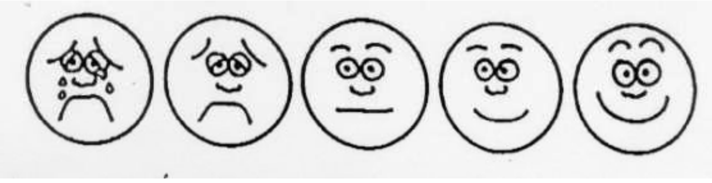

السؤال الخامس **كيف يؤثر المرض على شعورك تجاه ارتدائك ملابسك كل يوم؟**

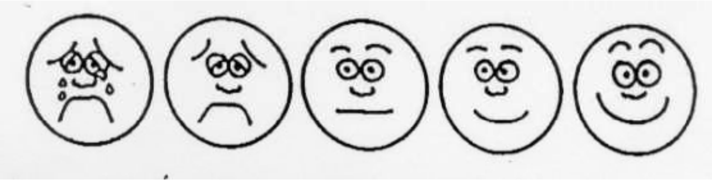

السؤال السادس **كيف يجعلك المرض تشعر تجاه مظهرك؟**

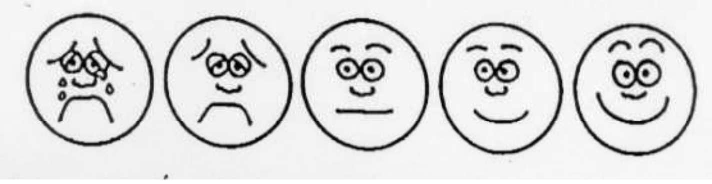

السؤال السابع **كيف يجعلك المرض تشعر تجاه نفسك؟**

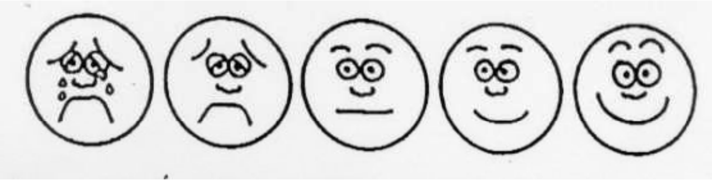

السؤال الثامن **كيف تشعر عندما تريد التحدث عن المرض؟**

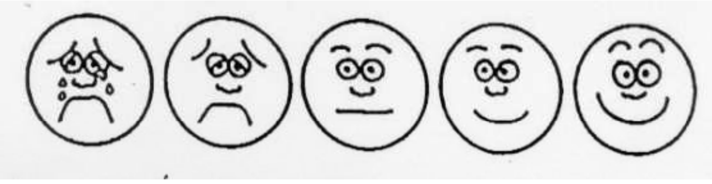

حقوق النشر 2007 © محفوظة للدكتورة ناديني مورثي. جميع الحقوق محفوظة.

السؤال التاسع **ما هو شعورك عندما يجب عليك الذهاب إلى الطبيب المعالج بسبب المرض؟**

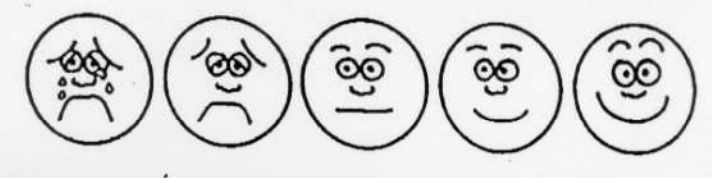

السؤال العاشر **كيف تشعر تجاه الذهاب إلى المستشفى بسبب المرض؟**

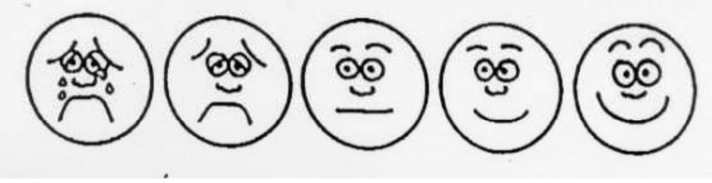

السؤال الحادي عشر **كيف يجعلك المرض تشعر تجاه مستقبلك؟**

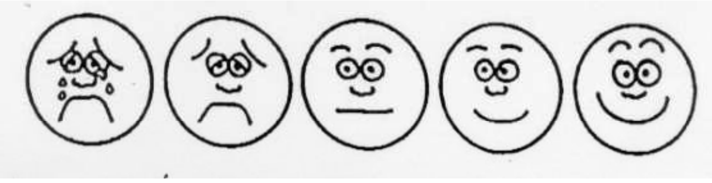

السؤال الثاني عشر **كيف تشعر تجاه تناول الأدوية الخاصة يالمرض؟**

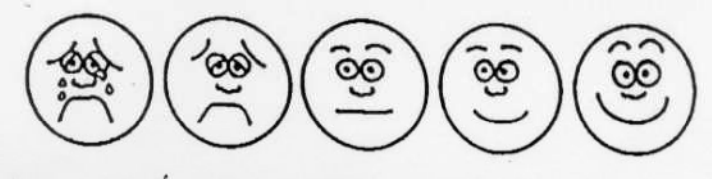

السؤال الثالث عشر **كم أنت قلق بسبب الآثار الجانبية لأدوية المرض؟**

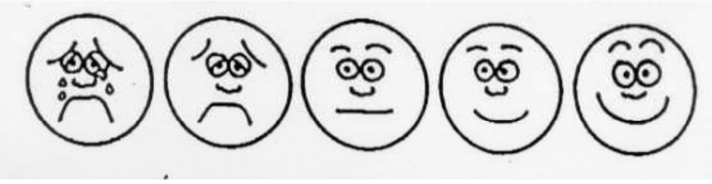

السؤال الرابع عشر **كم أنت قلق خشية أن تسوء حالتك المرضية؟**

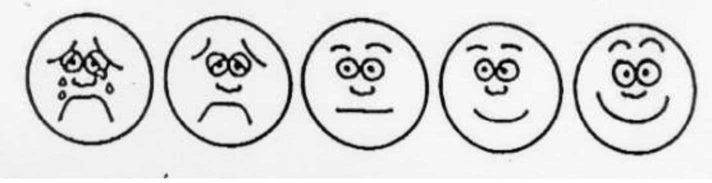

السؤال الخامس عشر **كم هو مقدار شعورك بالوحدة بسبب المرض؟**

حقوق النشر 2007 © محفوظة للدكتورة ناديني مورثي. جميع الحقوق محفوظة.

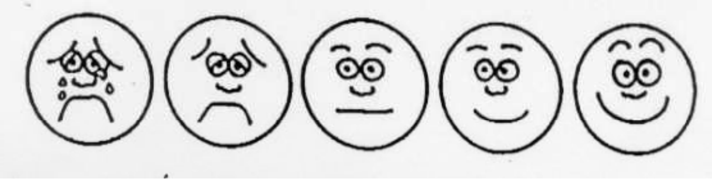

السؤال السادس عشر كيف تجعلك أسرتك تشعر؟

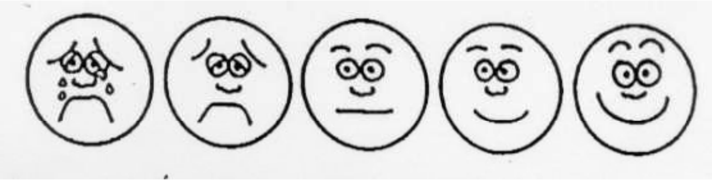

السؤال السابع عشر كيف يؤثر ألم المرض على شعورك؟

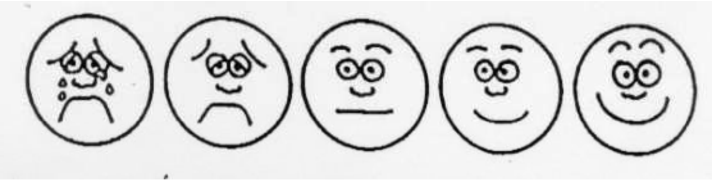

السؤال الثامن عشر كيف تشعر تجاه عدم قدرتك على الخروج في الشمس بسبب المرض؟

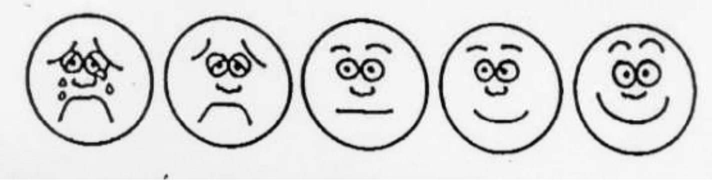

السؤال التاسع عشر إلى أي مدى يؤدى المرض إلى شعورك بالإجهاد؟

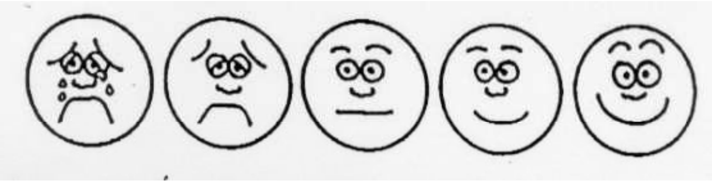

حقوق النشر 2007 © محفوظة للدكتورة ناديني مورثي. جميع الحقوق محفوظة.

من السؤال العشرون "أ" إلى السؤال السادس والعشرون "أ"، أجب بـ"نعم" أو "لا"، ثم اتبع التعليمات التالية:

السؤال العشرون أ **لقد تغير أصدقائي بسبب المرض . "نعم" أم "لا "من فضلك ضع دائرة حول "نعم" أو "لا"،**

إذا أجبت "نعم" اذهب إلى سؤال رقم 20ب

20ب: كيف يجعلك المرض تشعر تجاه أصدقائك؟

من فضلك ضع دائرة حول الوجه الذي يصف شعورك.

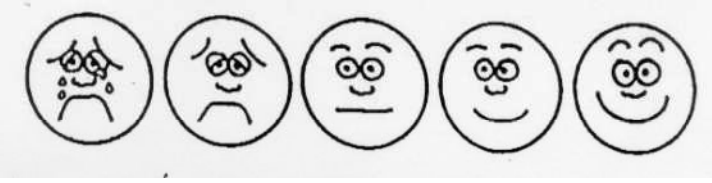

السؤال الحادي والعشرون أ **يتفهم أصدقائي المرض.** **"نعم" أم "لا"**

إذا أجبت ب "لا" اذهب إلى سؤال رقم 21ب

21ب: كيف تشعر تجاه عدم تفهم أصدقائك للمرض؟

من فضلك ضع دائرة حول الوجه الذي يصف شعورك.

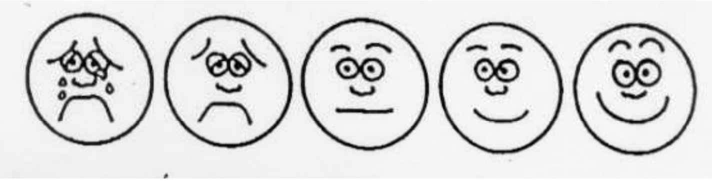

السؤال الثاني والعشرون أ **يؤثر المرض علي أدائي بالمدرسة .** **"نعم" أم "لا"**

إذا أجبت بـ "نعم" اذهب إلى سؤال 22ب

22ب:. كيف يجعلك المرض تشعر تجاه أدائك بالمدرسة؟

من فضلك ضع دائرة حول الوجه الذي يصف شعورك.

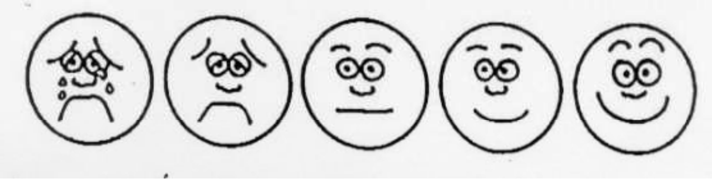

السؤال الثالث والعشرون أ **لقد تغيبت عن المدرسة بسبب المرض** **"نعم" أم "لا"**

إذا أجبت بـ "نعم" اذهب إلى سؤال 23ب

23ب. كيف تشعر تجاه تغيبك عن المدرسة بسبب المرض؟

من فضلك ضع دائرة حول الوجه الذي يصف شعورك.

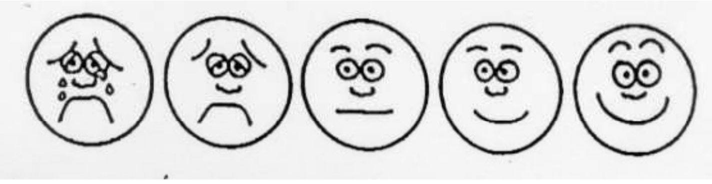

حقوق النشر 2007 © محفوظة للدكتورة نانديني مورثي. جميع الحقوق محفوظة.

السؤال الرابع والعشرون أ **يؤثر المرض على نشاطاتي خارج المدرسة .** **"نعم"** **أم "لا"**

إذا أجبت ب "نعم" اذهب إلي سؤال ب24

24ب: كيف تشعر تجاه التأثير الذي يسببه المرض على نشاطاتك خارج المدرسة؟

من فضلك ضع دائرة حول الوجه الذي يصف شعورك

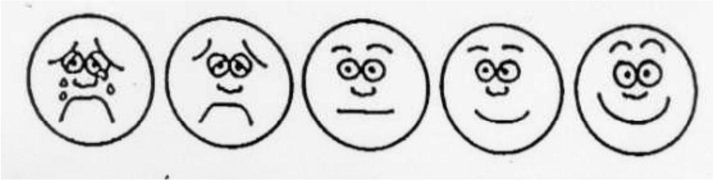

السؤال الخامس والعشرون أ **يؤثر المرض على وقت المرح الذي أقضيه مع أصدقائي.** **" نعم"** **أم "لا"**

إذا أجبت بـ "نعم" اذهب إلى سؤال 25ب

25ب:ما هو شعورك تجاه تأثير المرض على وقت المرح الذي تقضيه مع أصدقائك؟

من فضلك ضع دائرة حول الوجه الذي يصف شعورك.

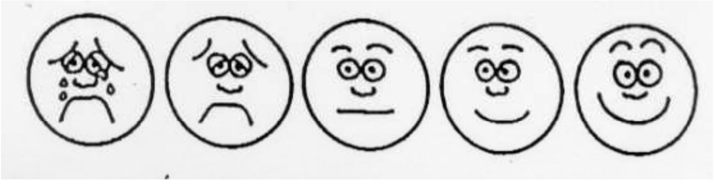

السؤال السادس والعشرون أ **يؤثر المرض على اشتراكي في النشاطات البدنية (مثل الألعاب الرياضية في المدرسة، الجري، المشي، أو حتى اللعب خارج المنزل).**

أجب بـ"نعم" **أو "لا"**

إذا أجبت بـ "نعم" اذهب إلى سؤال 26ب.

26ب:. كيف تشعرتجاه تاثير المرض على اشتراكك في النشاطات البدنية كالجري،المشي، واللعب خارج المنزل.

من فضلك ضع دائرة حول الوجه الذي يعكس شعورك؟

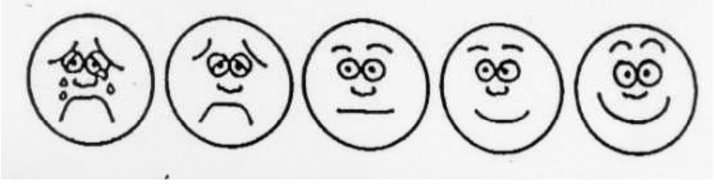

أعدت الترجمة إلى العربية مع المحافظة على بنية السؤال بطابعه الانكليزي. دعاء عبد الرحيم

قام بالمراجعة والتصحيح د.سليمان المعيوف. مستشفى الملك فيصل التخصصي ومركز الأبحاث بالرياض. المملكة العربية السعودية

حقوق النشر 2007 © محفوظة للدكتورة ناديني مورثي. جميع الحقوق محفوظة.

Arabic-Saudi Arabia

المقياس البسيط لتقييم أثر مرض الأطفال
(SMILY-Illness) – نسخة الأبوين

رقم الدراسة__ التاريخ__/___/___20

من أجل هذا الاستبيان أخذنا الصور الخمسة الآتية من أوجه وونغ - بيكر لتقييم الألم، وقمنا بإدخال تغييرات عليها بموافقة د. إل. وونغ. سوف تظهر هذه الأشكال الخمسة تحت كل سؤال.

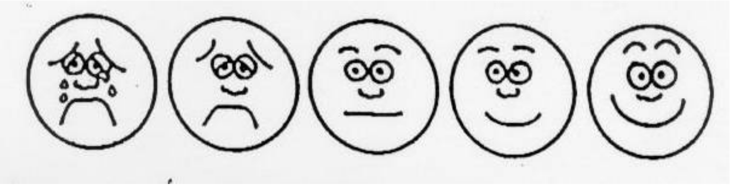

من المؤلف ونغ د.ل هوكينبيري - إيتون إم ويلسون، دي وينكليستاين إم إل، شوارتز بي: أساسيات التمريض في طب الأطفال لونغ، الإصدار 6، سانت لويس، 2001، موسبي، صفحة 1301، حقوق النشر لشركة موسبي. أُعيدت الطباعة بتصريح.

SMILY-illness محمي بحقوق النشر الخاصة بالدكتورة نانديني مورثي، "MD MS، RWJMS-UMDNJ"، نيوبرونسويك، بولاية نيوجرسي. ويمكن استخدام الاستبيان في الوقت الحاضر فقط عن طريق الدكتورة نانديني مورثي وفريق الباحثين العامل معها.

الأسئلة

هذا الاستبيان يحتوي على عدة أسئلة عن "مرض الأطفال" وشعورك نحوه. اختر ما تراه مناسب ويرجى العلم أنه لا توجد أجوبة خاطئة .إذا كان لديك أي سؤال فلا تتردد بطرحه علينا وان كان لديك أي تعليق فيمكنك تدوينه على ظهر الصفحتين 5 و 6.

هناك أمور مختلفة تؤثر في حياتك وتجعلك تشعر بالسرور أو الحزن. وهناك أمور تعتقد أنها مهمة بالنسبة لك، والتي بمقدورك القيام بها (أو ربما لا يكون بمقدورك القيام بها). هذه الأمور مجتمعه تحدد نوعية وجودة حياتك.

1. **كيف تقيم جودة حياة طفلك؟ من فضلك ضع دائرة حول الوجه الذي يعكس جودة حياته.**

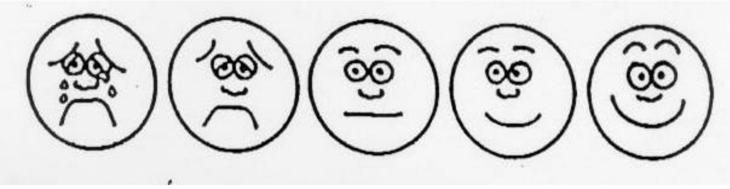

2. **كيف هي حالة طفلك الآن مع المرض؟ من فضلك ضع دائرة حول الوجه الذي يظهر وصف حالة طفلك الآن**

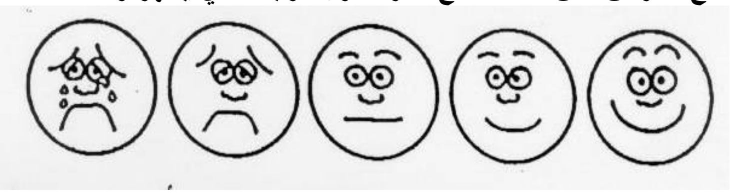

حقوق النشر © لسنة 2007 .Lakshmi Nandini Moorthy. جميع الحقوق محفوظة.

الأسئلة من 3 إلى 19 عن كيفية شعور طفلك خلال الشهر الماضي بسبب المرض. من فضلك ضع دائرة حول الوجه الذي يصف شعور طفلك.

.3 ما هو شعور طفلك كونه مصاب بالمرض؟

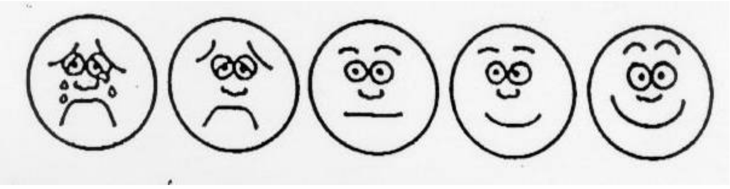

.4 كيف يوثر المرض على شعور طفلك تجاه ذهابه إلى المدرسة؟

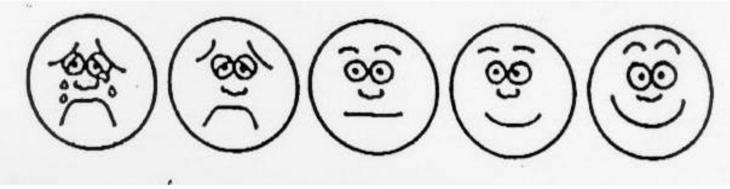

.5 كيف يوثر المرض على شعور طفلك تجاه ارتدائه ملابسه كل يوم؟

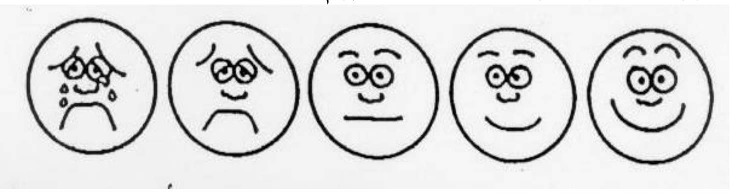

.6 كيف يوثر المرض على شعور طفلك تجاه مظهره؟

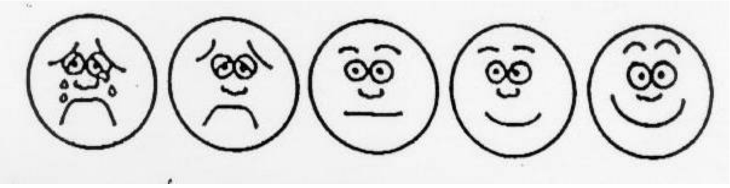

.7 كيف يجعلك المرض طفلك يشعر تجاه نفسه؟

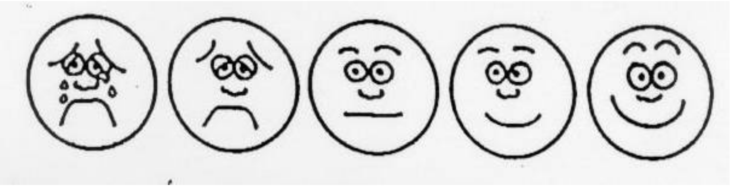

.8 كيف يشعر طفلك عندما يريد التحدث عن مرضه؟

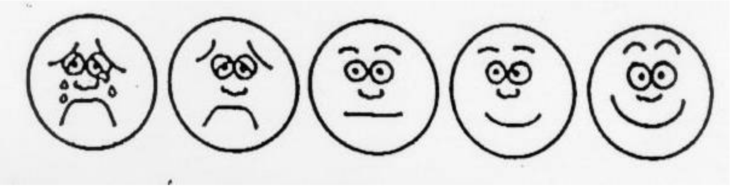

حقوق النشر © لسنة 2007 .Lakshmi Nandini Moorthy. جميع الحقوق محفوظة.

9. كيف يشعر طفلك تجاه الذهاب إلى الطبيب بسبب المرض؟

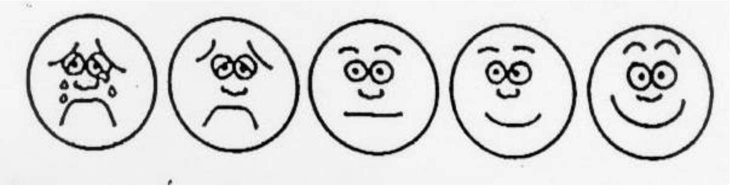

10. كيف يشعر طفلك تجاه الذهاب إلى المستشفى بسبب المرض؟

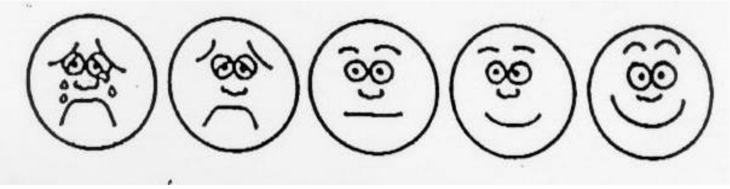

11. كيف يوثر المرض على شعور طفلك تجاه مستقبله؟

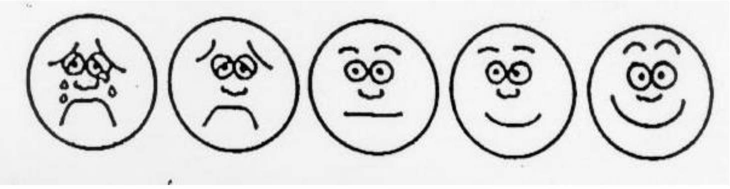

12. كيف يشعر طفلك تجاه اخذ الأدوية الخاصة بالمرض؟

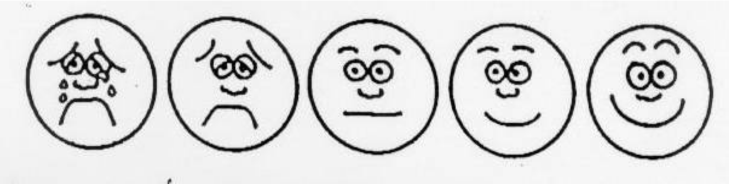

13. ما مدى قلق طفلك بسبب الآثار الجانبية لأدوية المرض؟

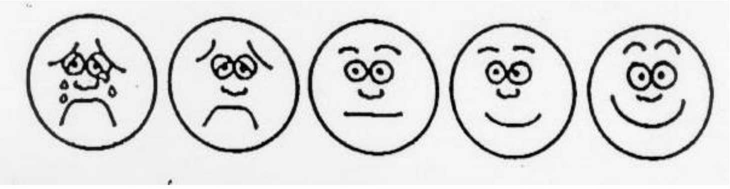

14. ما مدى قلق طفلك خشية أن تسوء حالته المرضية؟

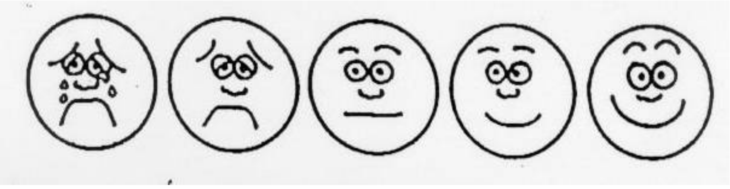

حقوق النشر © لسنة 2007 .Lakshmi Nandini Moorthy. جميع الحقوق محفوظة.

15. كيف يشعر طفلك بالوحدة بسبب المرض؟

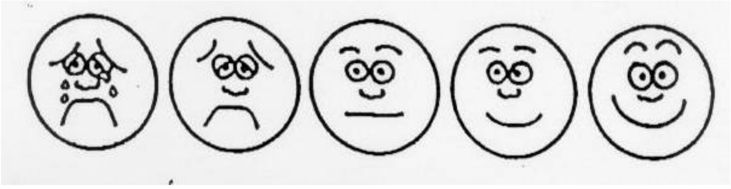

16. ما هو تأثير العائلة على شعور الطفل؟

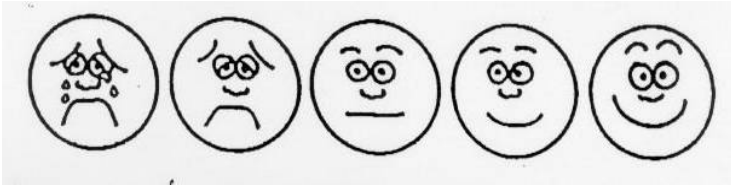

17. ما هو تأثير ألم المرض على شعور طفلك؟

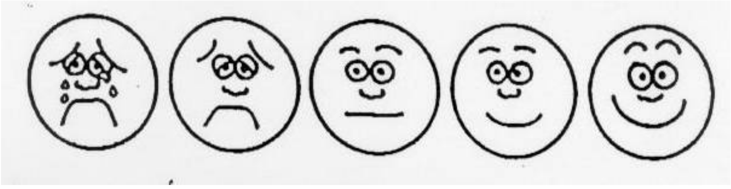

18. كيف يشعر طفلك تجاه عدم قدرته على الخروج في الشمس بسبب المرض؟

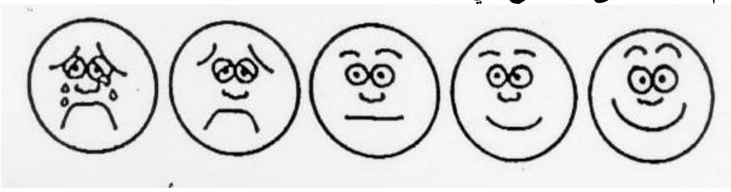

19. إلى أي مدى يشعر طفلك بالتعب والإجهاد؟

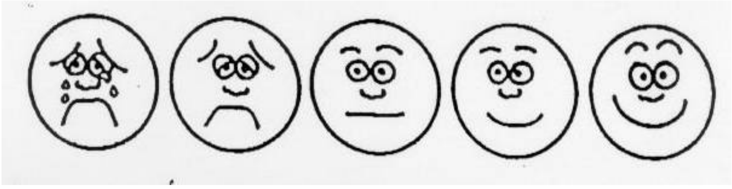

حقوق النشر © لسنة 2007 .Lakshmi Nandini Moorthy. جميع الحقوق محفوظة.

من السؤال 20 أ إلى السؤال 26 أ، أجب " نعم" أو "لا"، ثم اتبع التعليمات التالية

20 أ هل تغيرت علاقة أصدقائه معه بسبب مرضه. **"نعم"** **"لا"**

إذا كانت الإجابة" نعم"، " اذهب إلى سؤال 20ب .

"20ب" كيف جعلت الإصابة بالمرض شعور طفلك نحو أصدقائه؟

من فضلك ضع دائرة حول الوجه المناسب.

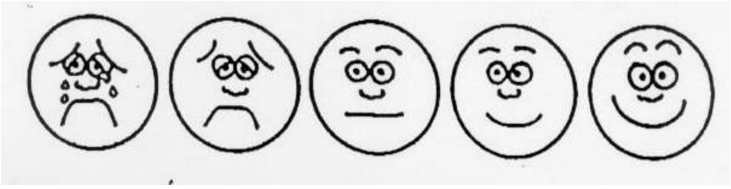

21 أ هل يتفهم أصدقاء طفلك بمرضه **"نعم"** **"لا"**

إذا كانت الإجابة "لا"، اذهب إلى سؤال 21ب.

21ب. كيف يشعر طفلك تجاه عدم تفهم أصدقائه مرضه؟

من فضلك ضع دائرة حول الوجه الذي يصف شعور طفلك.

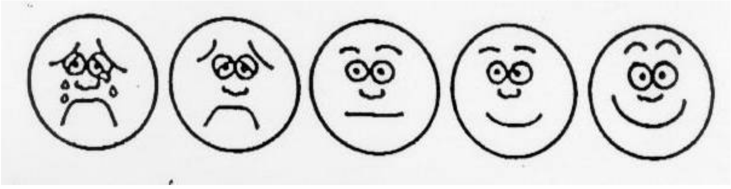

22 أ هل يؤثر المرض علي أداء طفلك بالمدرسة **"نعم"** **"لا"**

إذا كانت الإجابة "نعم" اذهب إلى سؤال 22ب

22ب. كيف يؤثر المرض على شعور طفلك تجاه أدائه بالمدرسة؟

من فضلك ضع دائرة حول الوجه الذي يصف شعور طفلك

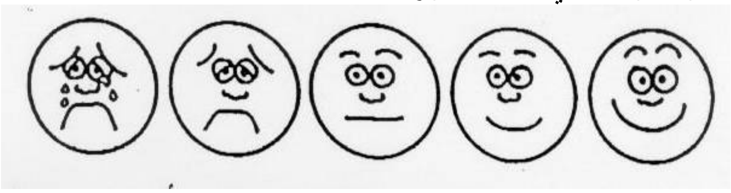

23 أ هل يغيب طفلك عن المدرسة بسبب بمرضه **"نعم"** **"لا"**

إذا كانت الإجابة "نعم" اذهب إلى سؤال 23ب.

23ب. كيف يشعر طفلك تجاه تغيبه عن المدرسة بسبب مرضه؟

من فضلك ضع دائرة حول الوجه الذي يصف شعور طفلك

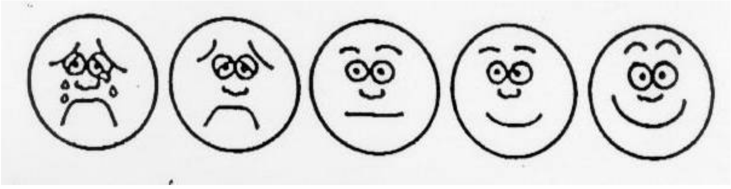

حقوق النشر © لسنة 2007 .Lakshmi Nandini Moorthy. جميع الحقوق محفوظة.

24 أ هل يؤثر المرض علي نشاطات طفلك خارج المدرسة "نعم" "لا"

إذا كانت الإجابة "نعم" اذهب إلى سؤال ب24.

24ب. كيف يشعر طفلك تجاه التأثير الذي يسببه مرضه على نشاطاته خارج المدرسة؟
من فضلك ضع دائرة حول الوجه الذي يصف شعور طفلك

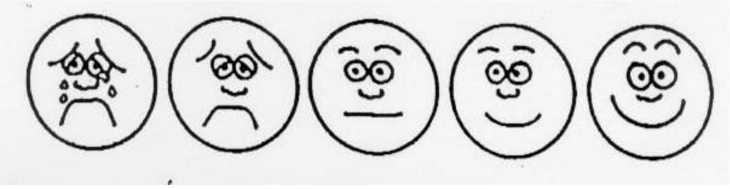

25 أ هل يؤثر المرض على الوقت المرح الذي يقضيه طفلك مع أصدقائه؟ "نعم" "لا"

إذا كانت الإجابة "نعم" اذهب إلى سؤال 25ب

25ب. كيف هو شعور طفلك تجاه التأثير الذي يسببه المرض على وقت المرح الذي يقضيه مع أصدقائه؟
من فضلك ضع دائرة حول الوجه الذي يصف شعور طفلك

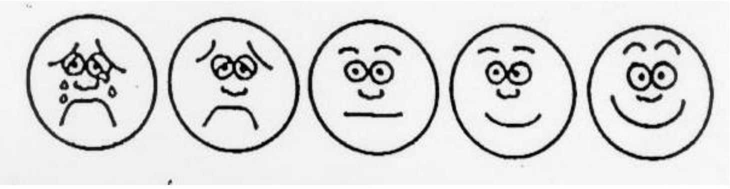

26 أ هل يؤثر المرض علي اشتراك طفلك في النشاطات البدنية (مثل نشاطات النادي الرياضي في المدرسة والجري، والمشي، ولعب أي من الألعاب الخارجية المفضلة) "نعم" "لا"

إذا كانت الإجابة "نعم"، اذهب إلى سؤال 26ب.

26ب. كيف يشعر طفلك تجاه التأثير الذي يسببه مرضه على مشاركته في النشاطات البدنية (مثل نشاطات النادي الرياضي في المدرسة و الجري، المشي، لعب أي من الألعاب الخارجية المفضلة)؟
من فضلك ضع دائرة حول الوجه الذي يصف شعور طفلك.

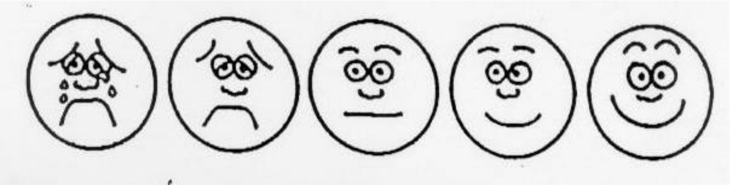

أعدت الترجمة إلى العربية مع المحافظة على بنية السؤال بطابعه الانكليزي. دعاء عبد الرحيم

قام بالمراجعة والتصحيح د.سليمان المعيوف. مستشفى الملك فيصل التخصصي ومركز الأبحاث بالرياض. المملكة العربية السعودية

حقوق النشر © لسنة 2007 .Lakshmi Nandini Moorthy. جميع الحقوق محفوظة.

المقياس البسيط لتقييم أثر مرض الأطفال
(SMILEY) – تقرير الطفل

رقم الدراسة__ التاريخ__/___/___20

من أجل هذا الاستبيان أخذنا الصور الخمسة الآتية من أوجه "وونغ بيكر" لتقييم الألم، وقمنا بإدخال تغييرات عليها بموافقة د. ل. وونغ. سوف تظهر هذه الأشكال الخمسة تحت كل سؤال.

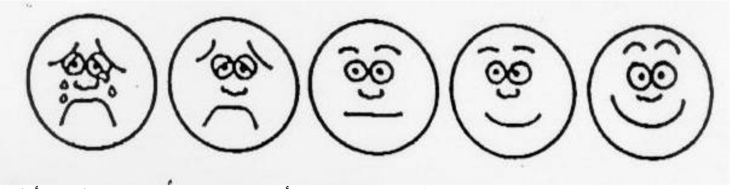

من المؤلف ونغ د.ل هوكينبيري - إيتون إم ويلسون، دي وينكليستاين إم إل، شوارتز بي: أساسيات التمريض في طب الأطفال لونغ، الإصدار 6، سانت لويس، 2001، موسبي، صفحة 1301، حقوق النشر لشركة موسبي. أعيدت الطباعة بتصريح.

SMILEY محمي بحقوق النشر للدكتورة نانديني مورثي "*MD MS, RWJMS-UMDNJ*"، نيوبرونسويك. ولاية نيوجرسي، يمكن استخدام الاستبيان في الوقت الحاضر فقط عن طريق الدكتورة نانديني مورثي وفريق الباحثين العامل معها.

الأسئلة

هذا الاستبيان يحتوي على عدة أسئلة عن "مرض الأطفال" وشعورك نحوها. اختر ما تراه مناسب ويرجى العلم أنه لا توجد أجوبة خاطئة .إذا كان لديك أي سؤال فلا تتردد بطرحه علينا وان كان لديك أي تعليق فيمكنك تدوينه على ظهر الصفحتين 5 و 6.

هناك أمور مختلفة تؤثر في حياتك وتجعلك تشعر بالسرور أو الحزن.
وهناك أمور تعتقد أنها مهمة بالنسبة لك، والتي بمقدورك القيام بها (أو ربما لا يكون بمقدورك القيام بها).
هذه الأمور مجتمعه تحدد نوعية وجودة حياتك.

1. كيف تقيم نوعية حياتك؟ من فضلك ضع دائرة حول الوجه الذي يظهر درجة نوعية حياتك.

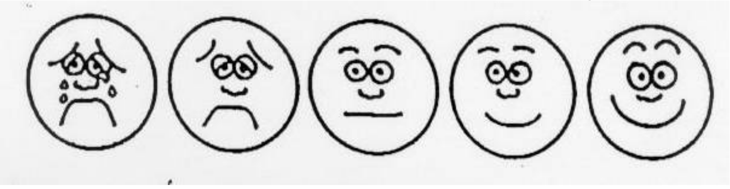

2. كيف هي حالتك الصحية الآن؟ من فضلك ضع دائرة حول الوجه الذي يظهر وصفاً لحالتك الآن.

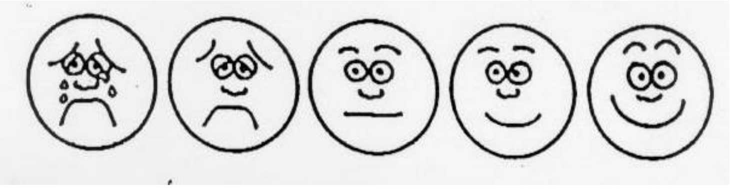

حقوق النشر © لسنة 2007 .Lakshmi Nandini Moorthy. جميع الحقوق محفوظة.

الأسئلة من 3 إلى 19 عن كيفية شعورك خلال الشهر الماضي بسبب المرض من فضلك ضع دائرة حول الوجه الذي يعبّر بشكل أفضل عن شعورك.

3. كيف يؤثر مرضك على شعور طفلك؟

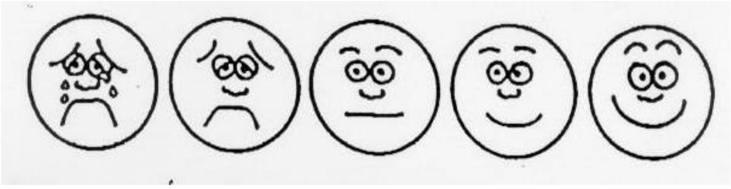

4. كيف يؤثر المرض على شعورك تجاه ذهابك إلي لمدرسه؟

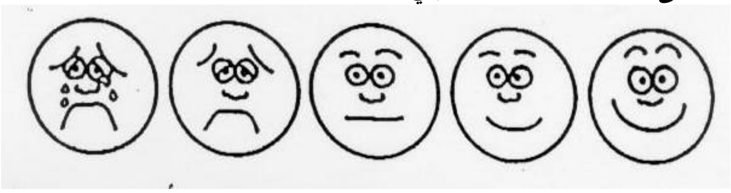

5. كيف يؤثر المرض على شعورك تجاه ارتدائك ملابسك كل يوم؟

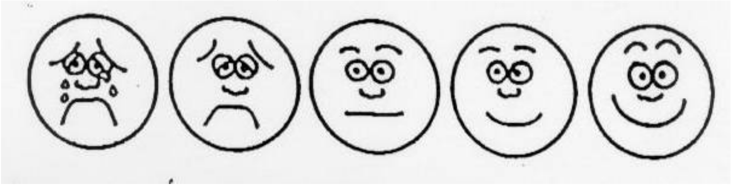

6. كيف يجعلك المرض تشعر تجاه مظهرك؟

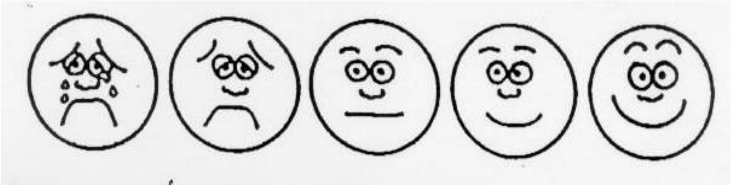

7. كيف يجعلك المرض تشعر تجاه نفسك؟

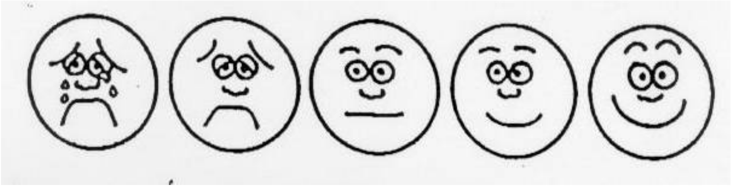

8. كيف تشعر عندما تريد التحدث عن المرض الذي تعاني منه؟

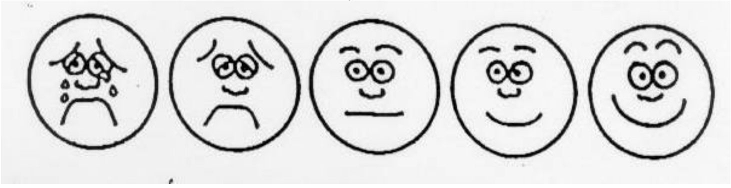

حقوق النشر © لسنة 2007 .Lakshmi Nandini Moorthy. جميع الحقوق محفوظة.

9. ما هو شعورك عندما يحين موعد ذهابك إلى الطبيب المعالج لالمرض؟

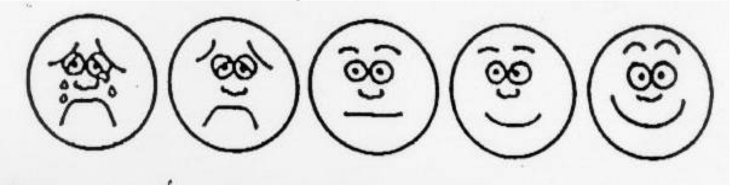

10.كيف تشعر تجاه الذهاب إلى المستشفى بسبب المرض؟

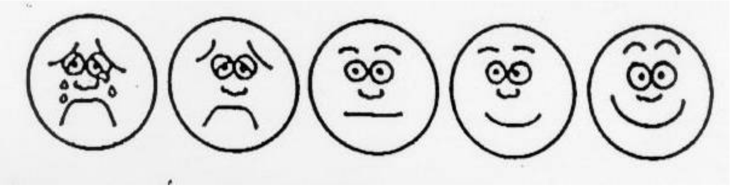

11.كيف يجعلك المرض تشعر تجاه مستقبلك؟

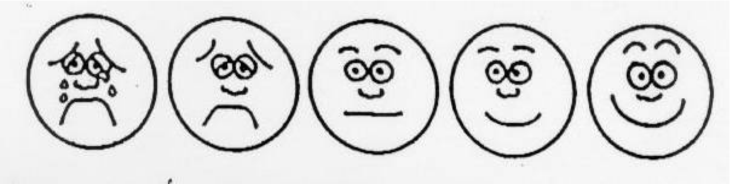

12.كيف تشعر تجاه تناول الأدوية الخاصة بالمرض؟

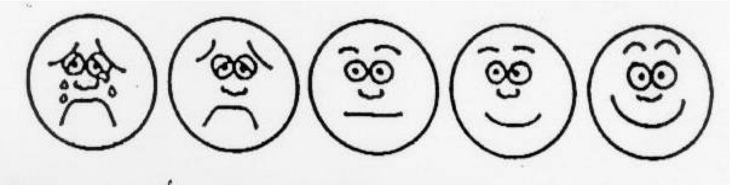

13.كم أنت قلق بسبب الآثار الجانبية لأدوية المرض؟

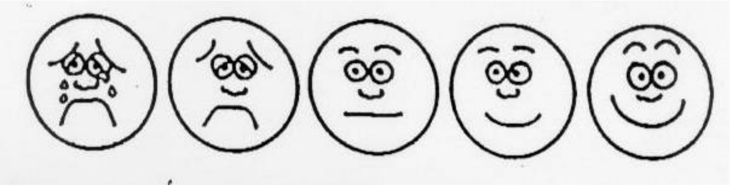

14. كم أنت قلق خشية أن تسوء حالتك المرضية؟

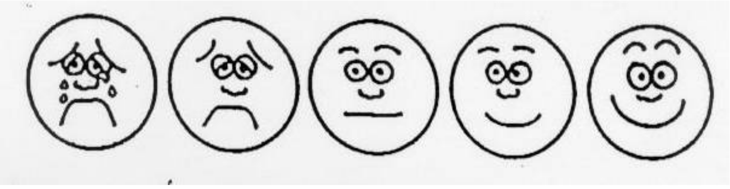

حقوق النشر © لسنة 2007 Lakshmi Nandini Moorthy. جميع الحقوق محفوظة.

15.كم هو مقدار شعورك بالوحدة بسبب المرض؟

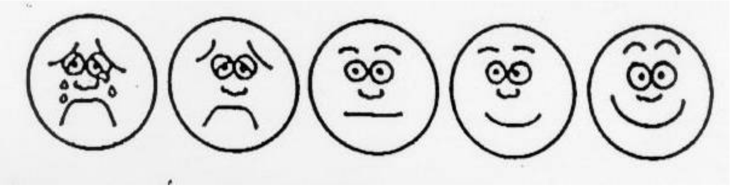

16.كيف تؤثر عائلتك على شعورك؟

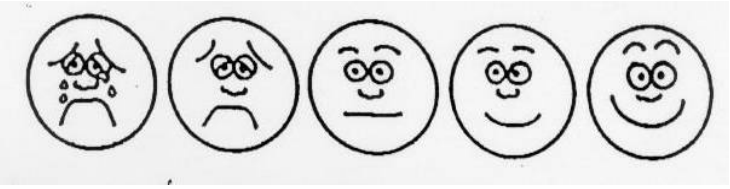

17.كيف يجعلك ألم المرض تشعر؟

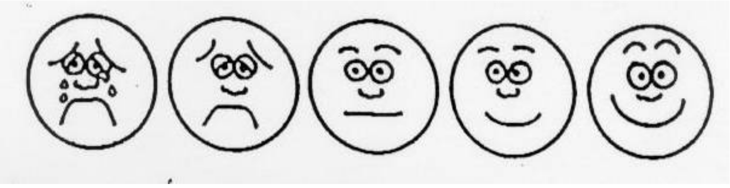

18.كيف تشعر تجاه عدم القدرة على الخروج في الشمس بسبب المرض؟

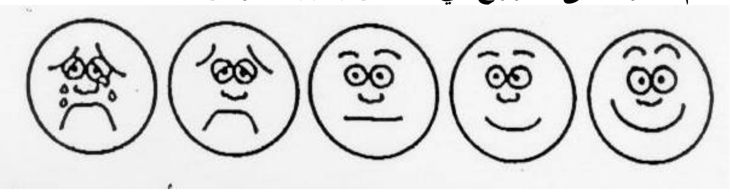

19.إلى أي مدى يؤثر المرض على شعورك بالإجهاد؟

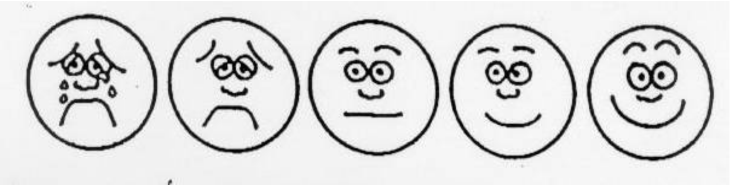

حقوق النشر © لسنة 2007 .Lakshmi Nandini Moorthy. جميع الحقوق محفوظة.

**من السؤال 20 أ إلى السؤال 26 أ، أجب بـ "نعم" أو "لا"، ثم اتبع التعليمات التالية**

20 أ لقد تغير أصدقائي بسبب مرضي من فضلك ضع دائرة حول "نعم" أو "لا"

"نعم" **"لا"**

، إذا كانت الإجابة "نعم" اذهب إلى السؤال 20 ب

20ب. كيف يجعلك المرض تشعر تجاه أصدقائك؟

من فضلك ضع دائرة حول الوجه الذي يصف شعورك.

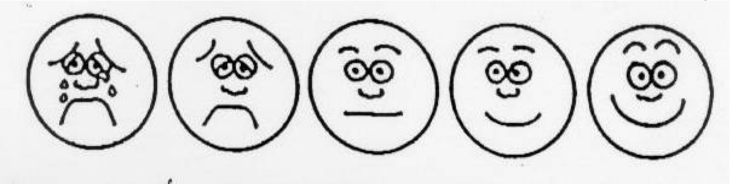

21 أ يتفهم أصدقائي مرضي **"نعم"** **"لا"**

إذا كانت الإجابة "لا" اذهب إلى سؤال 21ب.

21ب. كيف تشعر تجاه عدم تفهم أصدقائك للمرض؟

من فضلك ضع دائرة حول الوجه الذي يصف شعورك.

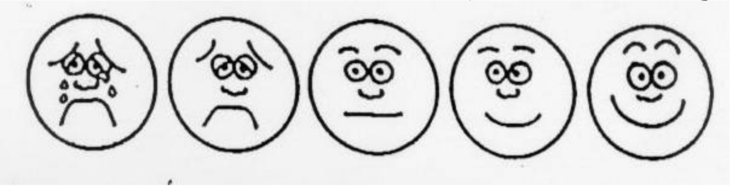

22 أ هل يؤثر المرض علي أدائك بالمدرسة **"نعم"** **"لا"**

إذا كانت الإجابة "نعم" اذهب إلى سؤال 22ب

22ب. كيف يجعلك المرض تشعر تجاه أدائك بالمدرسة؟

من فضلك ضع دائرة حول الوجه الذي يصف شعورك.

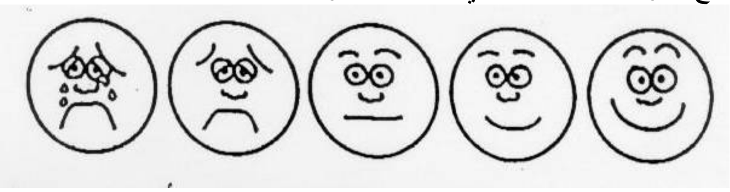

23 أ لقد تغيبت عن المدرسة بسبب المرض

إذا كانت الإجابة "نعم" اذهب إلى سؤال 23ب.

23ب. كيف تشعر تجاه تغيبك عن المدرسة بسبب المرض؟

من فضلك ضع دائرة حول الوجه الذي يصف شعورك.

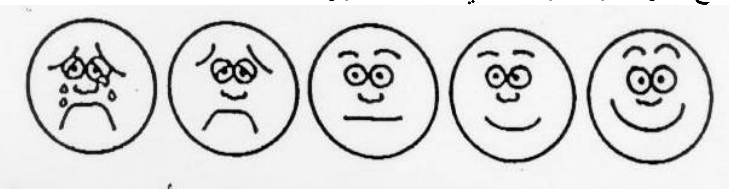

حقوق النشر © لسنة 2007. Lakshmi Nandini Moorthy. جميع الحقوق محفوظة.

24 أ هل يؤثر المرض على نشاطك خارج المدرسة "نعم" "لا"

إذا كانت الإجابة ب "نعم" اذهب إلي سؤال ب24

24ب. كيف شعر الطفل تجاه التأثير الذي يسببه مرضه على أنشطتك خارج المدرسة؟

من فضلك ضع دائرة حول الوجه الذي يصف شعورك

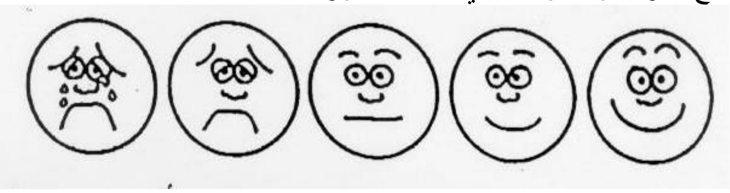

25 أ يؤثر المرض على وقت المرح الذي اقضيه مع أصدقائي "نعم" "لا"

إذا كانت الإجابة "نعم" اذهب إلى سؤال 25ب

25ب. كيف هو شعورك تجاه التأثير الذي يسببه المرض على الوقت المرح الذي أقضيه مع أصدقائي؟

من فضلك ضع دائرة حول الوجه الذي يصف شعورك.

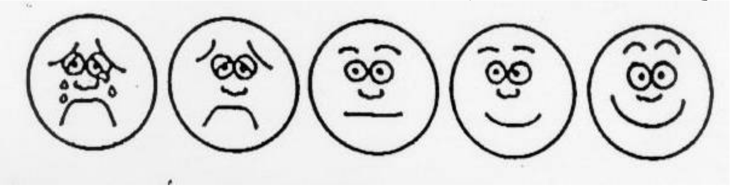

26 أ يؤثر المرض على اشتراكي في النشاطات البدنية (مثل الألعاب الرياضية في المدرسة، الجري، المشي، أو حتى اللعب خارج المنزل).

"نعم أو"لا"

إذا كانت الإجابة "نعم" ، تابع إلى السؤال 26ب.

26ب. هل تشعر أن المرض قد أثر على اشتراكك في النشاطات البدنية كالجري، والمشي، واللعب خارج المنزل.

من فضلك ضع دائرة حول الوجه الذي يعكس شعورك؟

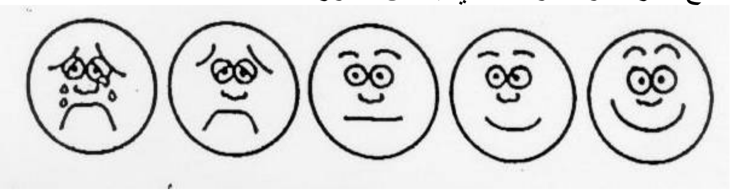

أعدت الترجمة إلى العربية مع المحافظة على بنية السؤال بطابعه الإنكليزي. دعاء عبد الرحيم

قام بالمراجعة والتصحيح د.سليمان المعيوف. مستشفى الملك فيصل التخصصي ومركز الأبحاث بالرياض. المملكة العربية السعودية

حقوق النشر © لسنة 2007. Lakshmi Nandini Moorthy. جميع الحقوق محفوظة.

JEDNODUCHÉ HODNOCENÍ VLIVU ONEMOCNĚNÍ NA DĚTI A DOSPÍVAJÍCÍ (SMILY-Illness) – verze pro rodiče

Czech

Číslo studie __ Datum __/__/20__(Czech)

V tomto dotazníku používáme následujících pět obličejů převzatých ze škály hodnocení bolesti od Wongové a Bakerové, které jsme upravili se svolením paní D. L. Wongové. Těchto pět obličejů se bude objevovat pod každou otázkou.

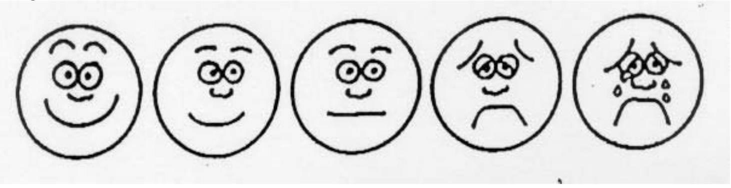

Z knihy Wong D. L., Hockenberry-Eaton M., Wilson D.,Winklestein M. L., Schwartz P.: Wong's Essentials of Pediatric Nursing (Wongové základy zdravotnické péče o děti), ed. 6, St. Louis, 2001, Mosby, st. 1301. Copyright Mosby. Přetisknuto se svolením.

SMILY-illness je koncept chráněný autorským právem doktorky L. Nandini Moorthyové, MD MS, RWJMS-UMDNJ, New Brunswick, NJ. V daném okamžiku může dotazník používat pouze Dr. L. Nandini Moorthyová a jí pověření výzkumní pracovníci.

OTÁZKY

V tomto dotazníku vám položíme různé otázky o nemoci vašeho dítěte a o tom, jak se cítí. Neexistují žádné chybné odpovědi. Prosíme vás, abyste se zeptali na jakékoliv nejasnosti. Pokud chcete uvést nějaké komentáře, prosím napište je za stránky 5 a 6.

Na život vašeho dítěte mají vliv různé okolnosti, které ho činí smutným nebo šťastným. Některé věci, které jsou pro vaše dítě důležité, je schopné dělat a jiné ne. Všechny tyto okolnosti v životě vašeho dítěte dohromady určují kvalitu jeho života.

1. Jaká je kvalita života vašeho dítěte? Prosím zaškrtněte (√) obličej, který nejlépe znázorňuje, jaká je kvalita života vašeho dítěte.

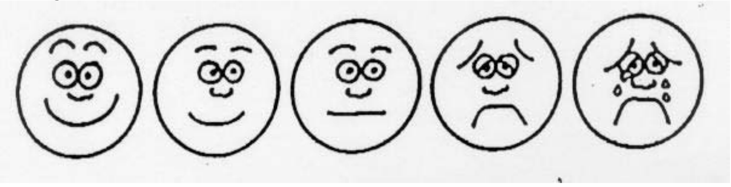

2. Jak je na tom právě teď nemoc vašeho dítěte? Prosím zaškrtněte (√) obličej, který nejlépe znázorňuje, v jakém stavu je nemoc vašeho dítěte.

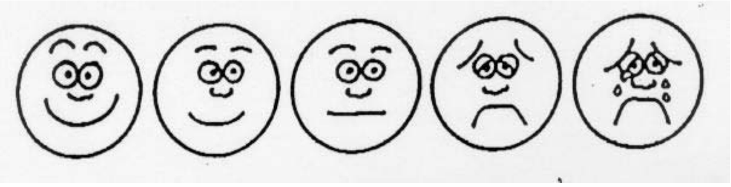

Copyright © 2007 Lakshmi Nandini Moorthy. Všechna práva vyhrazena.

Otázky 3-19 se týkají toho, jak se kvůli nemoci vaše dítě cítilo během uplynulého měsíce. Prosím zaškrtněte (√) obličej, který nejlépe znázorňuje vaši odpověď.

3. Jak se vaše dítě kvůli své nemoci cítí?

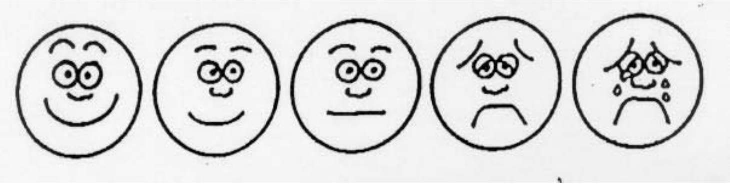

4. Jak se vaše dítě kvůli své nemoci cítí, když chodí do školy?

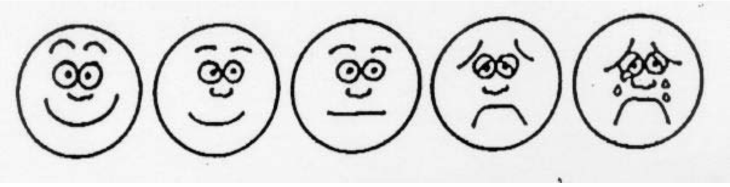

5. Jak se vaše dítě kvůli své nemoci cítí, když se každý den obléká?

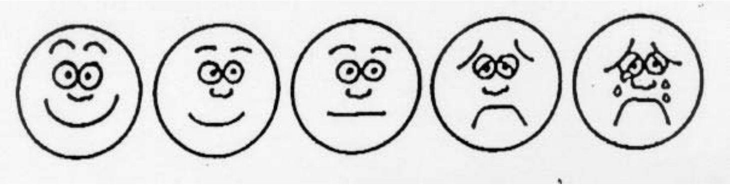

6. Jaký má vaše dítě kvůli své nemoci pocit z toho, jak vypadá?

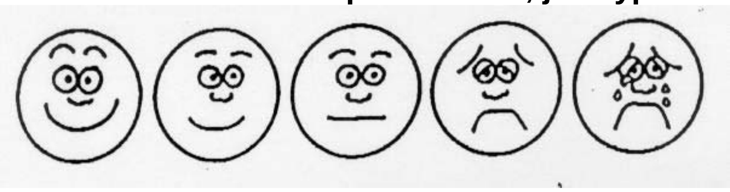

7. Jaký má vaše dítě kvůli své nemoci pocit samo ze sebe?

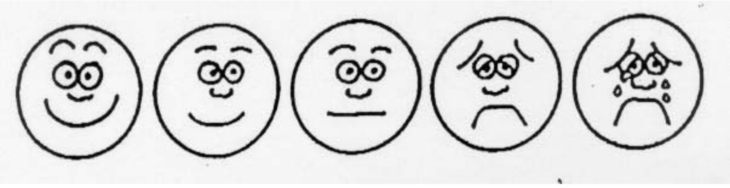

8. Jak se vaše dítě cítí, když potřebuje mluvit o své nemoci?

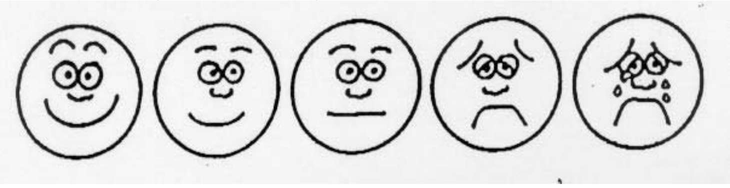

Copyright © 2007 Lakshmi Nandini Moorthy. Všechna práva vyhrazena.

9. **Jak se vaše dítě cítí kvůli tomu, že se svou nemocí chodí k lékaři?**

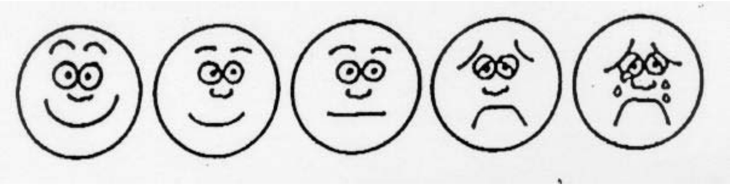

10. **Jak se vaše dítě cítí kvůli tomu, že se svou nemocí chodí do nemocnice?**

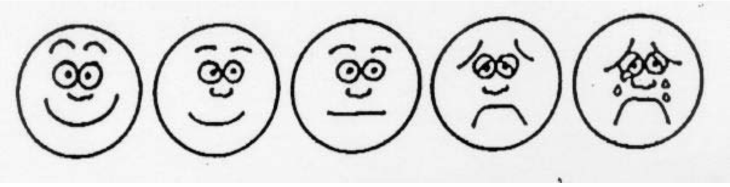

11. **Jaké má vaše dítě kvůli nemoci pocity ohledně své budoucnosti?**

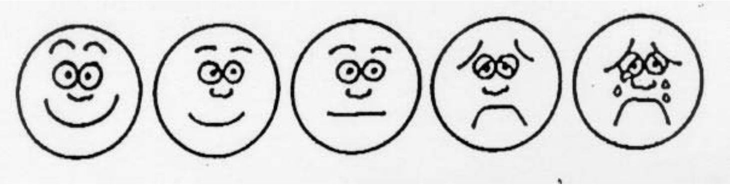

12. **Jak se vaše dítě cítí kvůli tomu, že na svoji nemoc bere léky?**

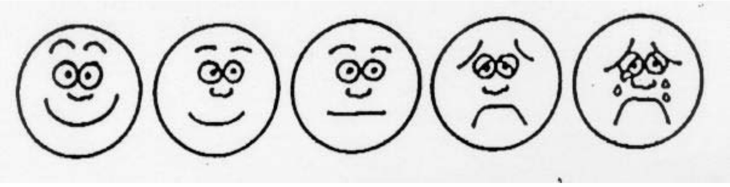

13. **Jak moc se vaše dítě obává vedlejších účinků léků, které užívá na nemoc?**

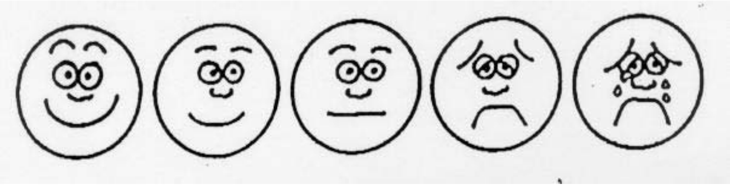

14. **Jak moc se vaše dítě obává toho, že se mu jeho nemoc zhorší?**

Copyright © 2007 Lakshmi Nandini Moorthy. Všechna práva vyhrazena.

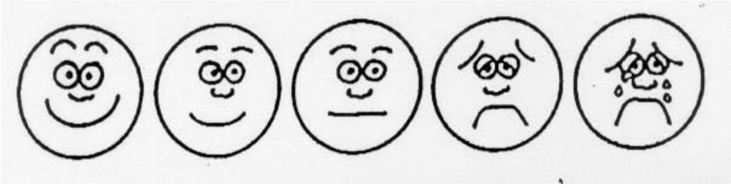

15. **Do jaké míry se vaše dítě cítí kvůli své nemoci osaměle?**

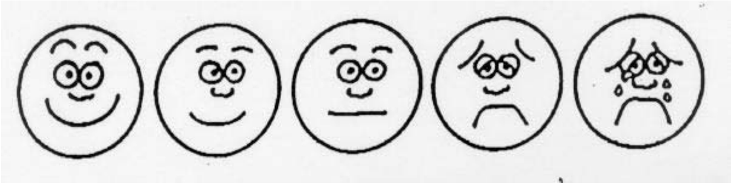

16. **Jak se vaše dítě cítí ve své rodině?**

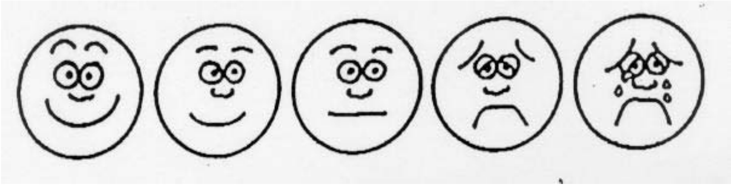

17. **Jak se vaše dítě cítí kvůli bolesti způsobené nemocí?**

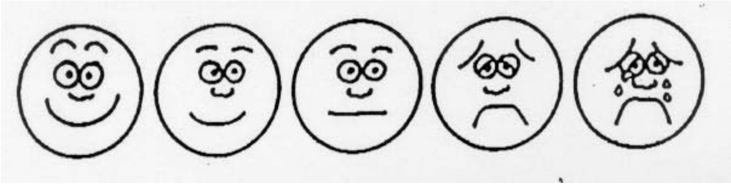

18. **Jaký má vaše dítě pocit z toho, že kvůli nemoci nesmí chodit na sluníčko?**

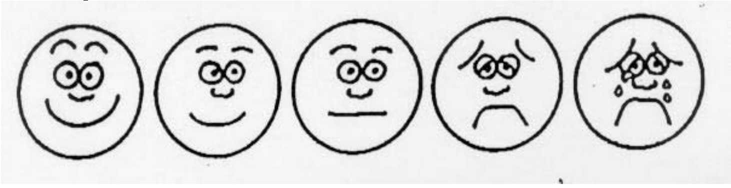

19. **Jak moc unavené se cítí vaše dítě kvůli své nemoci?**

Copyright © 2007 Lakshmi Nandini Moorthy. Všechna práva vyhrazena.

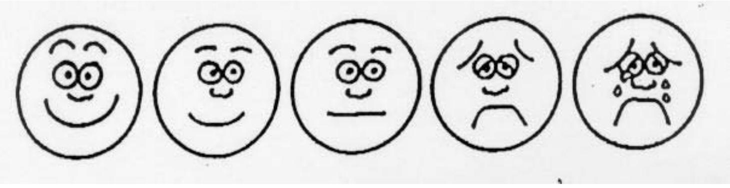

U otázek 20 A-26 A odpovězte „ano“, nebo „ne“. Poté se prosím řiďte pokyny.

20 A. Přátelé vašeho dítěte se k němu kvůli jeho nemoci začali chovat jinak. Prosím zaškrtněte „ano“, nebo „ne“. **Ano** **Ne**

Pokud „ano“, přejděte na otázku 20 B.

20 B. Jaký má vaše dítě kvůli **své nemoci** pocit ze svých přátel?
Prosím zaškrtněte obličej, který nejlépe znázorňuje, jak se vaše dítě cítí.

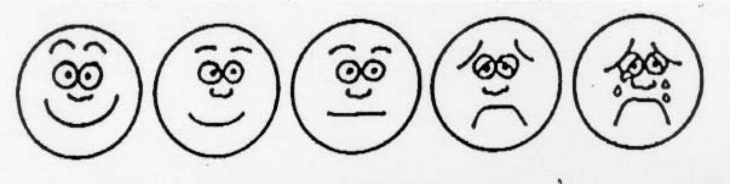

21 A. Přátelé vašeho dítěte chápou, že je nemocné. **Ano** **Ne**

Pokud jste odpověděl/a „ne“, přejděte na otázku 21 B.

21 B. Jak se vaše dítě cítí ohledně toho, že jeho přátelé nechápou, že je nemocné?
Prosím zaškrtněte obličej, který nejlépe znázorňuje, jak se vaše dítě cítí.

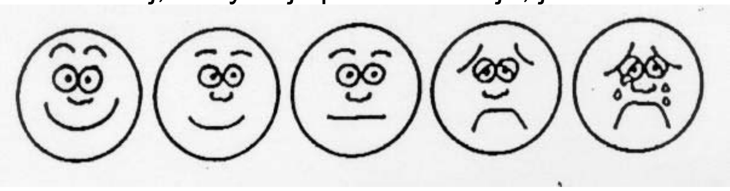

22 A. Nemoc zhoršuje výkon vašeho dítěte ve škole. **Ano** **Ne**

Pokud „ano“, přejděte na otázku 22 B.

22 B. Jaký má vaše dítě kvůli své nemoci pocit ze své práce ve škole?
Prosím zaškrtněte obličej, který nejlépe znázorňuje, jak se vaše dítě cítí.

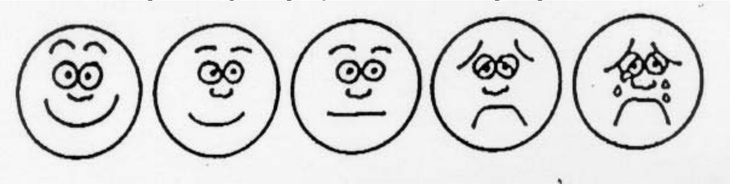

Copyright © 2007 Lakshmi Nandini Moorthy. Všechna práva vyhrazena.

23 A. Vaše dítě ve škole kvůli nemoci hodně zameškává. **Ano** **Ne**

Pokud „ano“, přejděte na otázku 23 B.

23 B. Jaký má vaše dítě pocit z toho, že kvůli nemoci zameškává školu? Prosím zaškrtněte obličej, který nejlépe znázorňuje, jak se vaše dítě cítí.

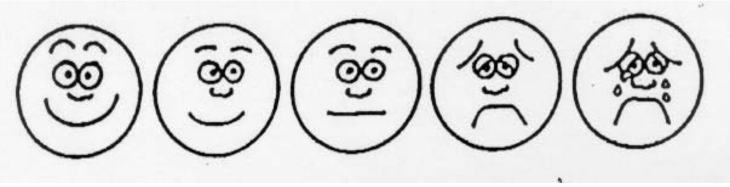

24 A. Nemoc omezuje mimoškolní aktivity vašeho dítěte.

Ano **Ne**

Pokud jste odpověděl/a „ano“, přejděte na otázku 24 B.

24 B. Jaký má vaše dítě pocit z toho, že nemoc omezuje jeho mimoškolní aktivity? Prosím zaškrtněte obličej, který nejlépe znázorňuje, jak se vaše dítě cítí.

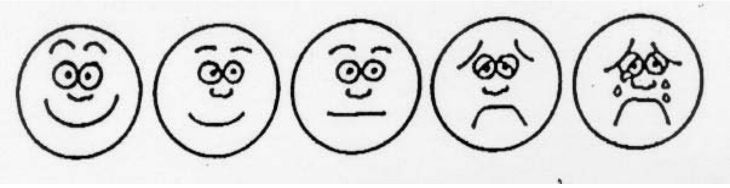

25 A. Kvůli nemoci si vaše dítě užije méně zábavy se svými přáteli.

Ano **Ne**

Pokud jste odpověděl/a „ano“, přejděte na otázku 25 B.

25 B. Jak se vaše dítě cítí ohledně toho, že si kvůli nemoci užije méně zábavy se svými přáteli? Prosím zaškrtněte obličej, který nejlépe znázorňuje, jak se vaše dítě cítí.

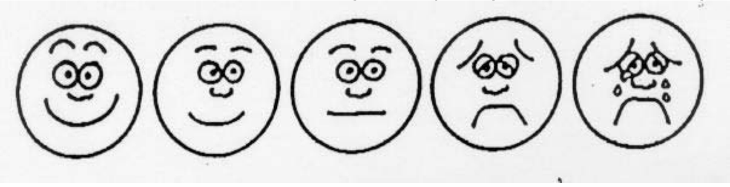

26 A. Kvůli nemoci je pro vaše dítě obtížnější se zúčastnit pohybových činností (jako je například tělocvik ve škole, běhání, chůze, oblíbená hra venku).

Ano **Ne**

Pokud jste odpověděl/a „ano“, přejděte na otázku 26 B.

26 B. Jak se vaše dítě cítí, že kvůli nemoci je pro ně obtížnější se zúčastnit pohybových činností (jako je například tělocvik ve škole, běhání, chůze, oblíbená hra venku)? Prosím zaškrtněte obličej, který nejlépe znázorňuje, jak se vaše dítě cítí.

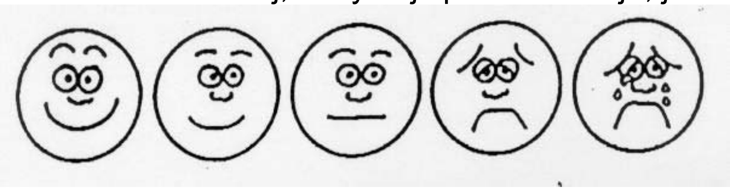

Copyright © 2007 Lakshmi Nandini Moorthy. Všechna práva vyhrazena.

Copyright © 2007 Lakshmi Nandini Moorthy. Všechna práva vyhrazena.

JEDNODUCHÉ HODNOCENÍ VLIVU ONEMOCNĚNÍ NA DĚTI A DOSPÍVAJÍCÍ (SMILY - Illness) – verze pro pacienty

Číslo studie __ Datum __/__/20__

V tomto dotazníku používáme následujících pět obličejů převzatých ze škály hodnocení bolesti od Wongové a Bakerové, které jsme upravili se svolením paní D. L. Wongové. Těchto pět obličejů se bude objevovat pod každou otázkou.

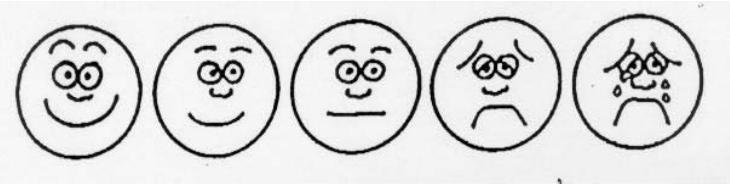

Z knihy Wong D. L., Hockenberry-Eaton M., Wilson D.,Winklestein M. L., Schwartz P.: Wong's Essentials of Pediatric Nursing (Wongové základy zdravotnické péče o děti), ed. 6, St. Louis, 2001, Mosby, st. 1301. Copyright Mosby. Přetisknuto se svolením.

SMILY-illness je koncept chráněný autorským právem doktorky L. Nandini Moorthyové, MD MS, RWJMS-UMDNJ, New Brunswick, NJ. V daném okamžiku může dotazník používat pouze Dr. L. Nandini Moorthyová a jí pověření výzkumní pracovníci.

OTÁZKY

V tomto dotazníku ti položíme pár otázek o tvé nemoci a o tom, jak se cítíš. Nejsou zde žádné chybné odpovědi. V případě, že ti něco nebude jasné, prosím, zeptej se. Pokud budeš chtít něco připsat, prosím, abys to napsal/a za stránky 5 a 6.

Na tvůj život působí mnoho věcí, ze kterých jsi smutná/ý nebo šťastná/ý. Některé věci, které jsou pro tebe důležité, můžeš dělat, jiné ne. Všechny tyto různé okolnosti ve tvém životě se dohromady podílejí na tom, jaký tvůj život je.

1. Jaký je tvůj život? Prosím zaškrtni (√) obličej, který nejlépe znázorňuje, jaký je tvůj život.

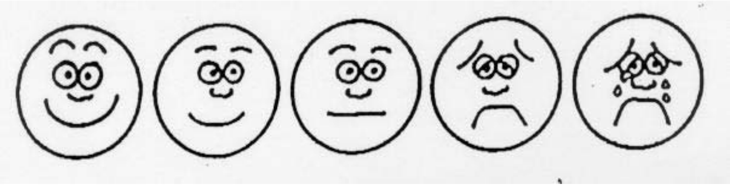

2. Jak je na tom právě teď tvá nemoc? Prosím zaškrtni (√) obličej, který nejlépe znázorňuje, jak je na tom tvá nemoc.

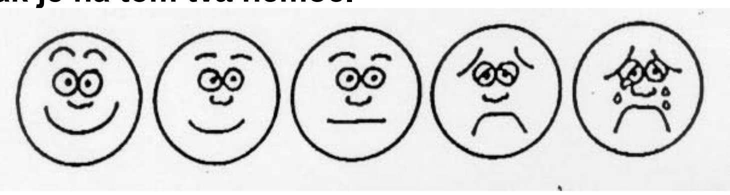

Copyright © 2007 Lakshmi Nandini Moorthy. Všechna práva vyhrazena.

Otázky 3-19 se týkají toho, jak ses kvůli své nemoci cítil/a během uplynulého měsíce. Prosím zaškrtni (√) obličej, který nejlépe znázorňuje, jak ses cítil/a.

3. Jak se cítíš při svém onemocnění?

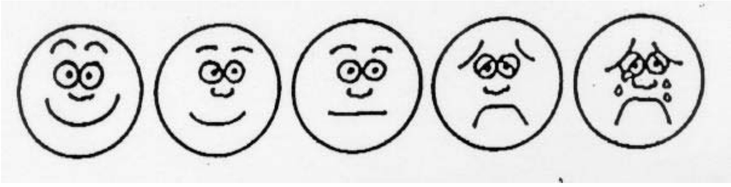

4. Jak se při své nemoci cítíš, když chodíš do školy?

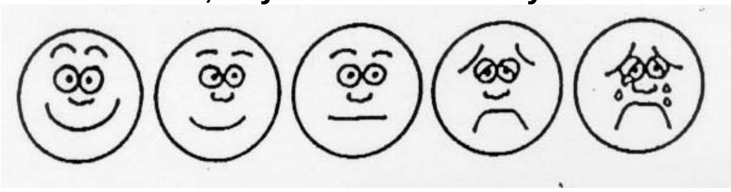

5. Jak se při své nemoci cítíš, když se každý den oblékáš?

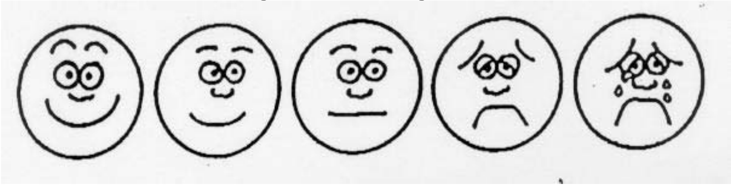

6. Jaký máš při své nemoci pocit z toho, jak vypadáš?

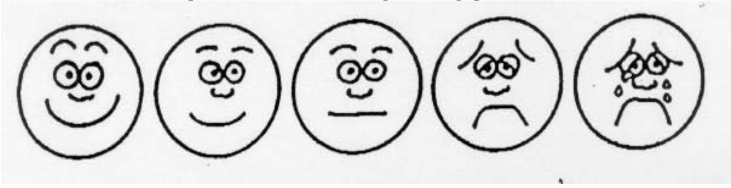

7. Jaký máš kvůli nemoci pocit sama/sám ze sebe?

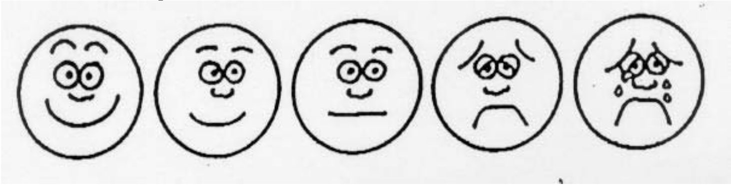

8. Jak se cítíš, když potřebuješ o své nemoci mluvit?

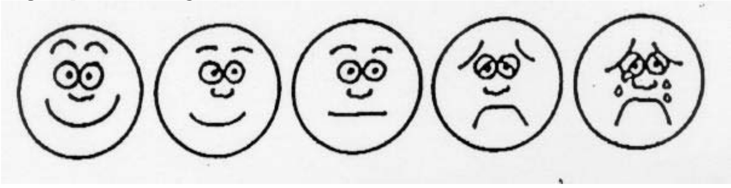

Copyright © 2007 Lakshmi Nandini Moorthy. Všechna práva vyhrazena.

9. Jak se cítíš, když jdeš kvůli nemoci k lékaři?

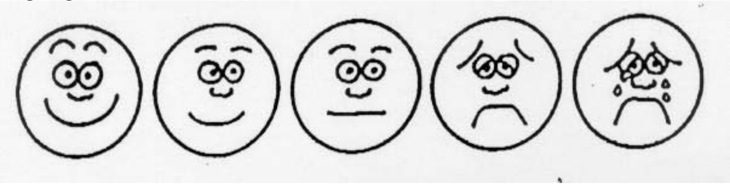

10. Jak se cítíš kvůli tomu, že se svou nemocí chodíš do nemocnice?

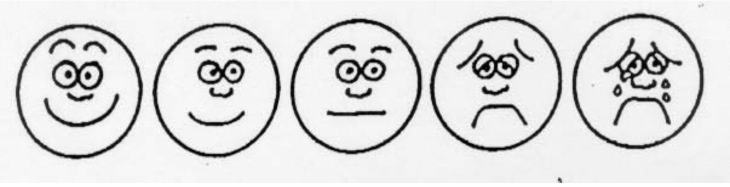

11. Jaké máš kvůli nemoci pocity ohledně své budoucnosti?

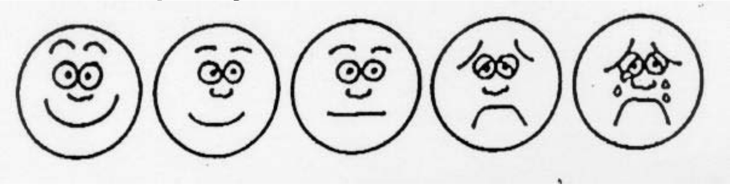

12. Jak se cítíš kvůli tomu, že na nemoc bereš léky?

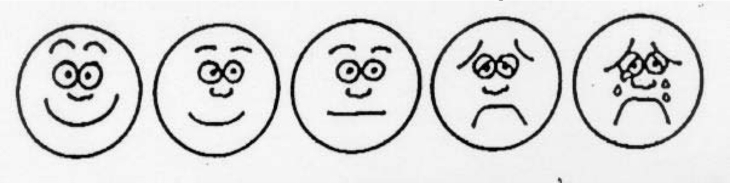

13. Jak moc se obáváš vedlejších účinků léků, které bereš?

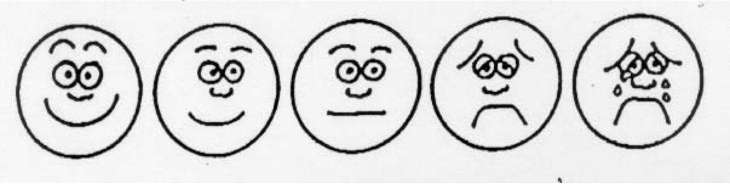

14. Jak moc se obáváš toho, že se tvoje nemoc zhorší?

Copyright © 2007 Lakshmi Nandini Moorthy. Všechna práva vyhrazena.

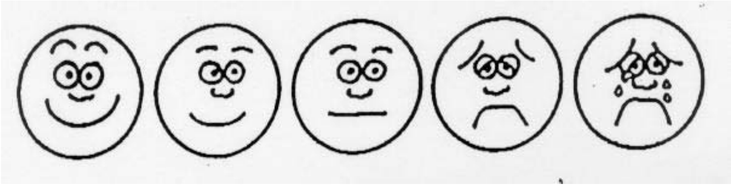

15. Jak moc se kvůli nemoci cítíš osaměle?

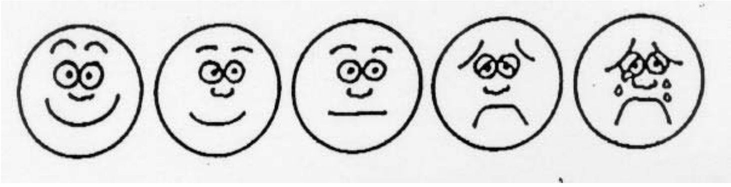

16. Jaké pocity u tebe vyvolává tvoje rodina?

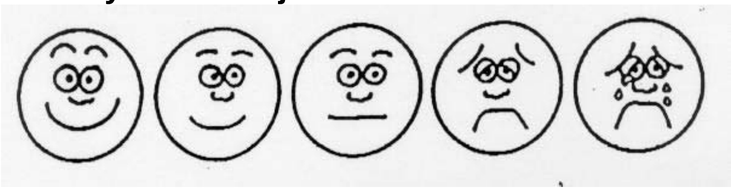

17. Jaký pocit v tobě vyvolává bolest způsobená nemocí?

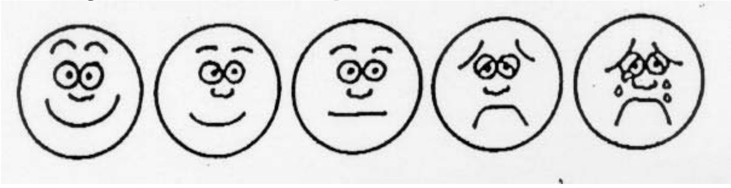

18. Jaké to je, když kvůli nemoci nesmíš na sluníčko?

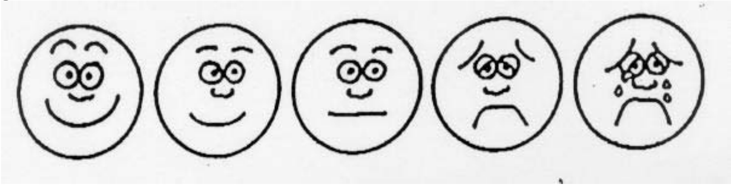

19. Jak moc se cítíš při své nemoci unavená/unavený?

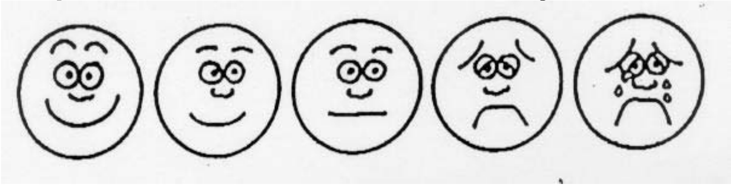

Copyright © 2007 Lakshmi Nandini Moorthy. Všechna práva vyhrazena.

U otázek 20 A-26 A odpověz „ano“, nebo „ne“. Poté se prosím řiď pokyny.

20 A. Moji kamarádi se kvůli mé nemoci chovají jinak. Prosím zaškrtni „ano“, nebo „ne“.

Ano Ne

Pokud „ano“, přejdi na otázku 20 B.

20 B. Jaký máš kvůli své nemoci pocit ze svých přátel?

Prosím zaškrtni obličej, který nejlépe znázorňuje, jak se cítíš.

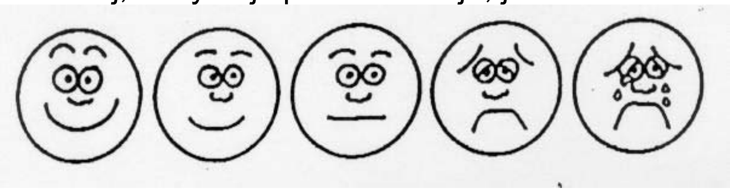

21 A. Moji kamarádi chápou, že jsem nemocný. Ano Ne

Pokud jsi odpověděl/a „ne“, přejdi na otázku 21 B.

21 B. Jak se cítíš kvůli tomu, že tvoji přátelé nechápou, že máš nemoc? Prosím zaškrtni obličej, který nejlépe znázorňuje, jak se cítíš.

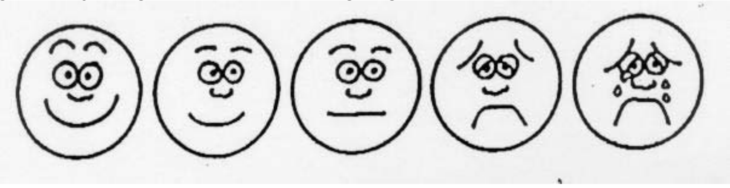

22 A. Nemoc zhoršuje moji práci ve škole. Ano Ne

Pokud „ano“, přejdi na otázku 22 B.

22 B. Jaký máš kvůli své nemoci pocit ze své práce ve škole? Prosím zaškrtni obličej, který nejlépe znázorňuje, jak se cítíš.

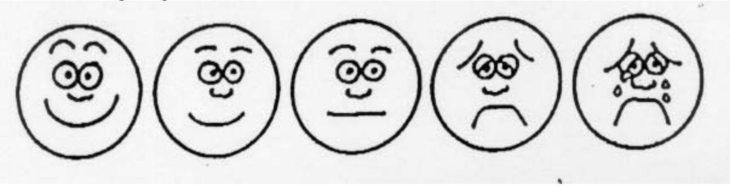

23 A. Kvůli nemoci vynechávám školu. Ano Ne

Pokud „ano“, přejdi na otázku 23 B.

23 B. Jaký máš pocit z toho, že kvůli nemoci vynecháváš školu?

Copyright © 2007 Lakshmi Nandini Moorthy. Všechna práva vyhrazena.

Prosím zaškrtni obličej, který nejlépe znázorňuje, jak se cítíš.

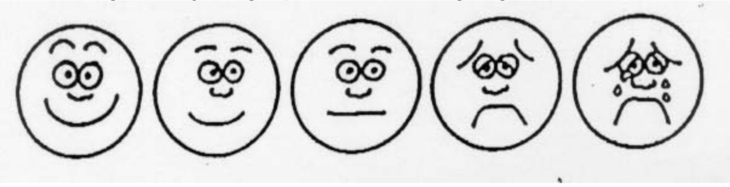

24 A. Nemoc omezuje moje mimoškolní činnosti. Ano Ne

Pokud jsi odpověděl/a „ano", přejdi na otázku 24 B.

24 B. Jaký máš pocit z toho, že nemoc omezuje tvé mimoškolní činnosti? Prosím zaškrtni obličej, který nejlépe znázorňuje, jak se cítíš.

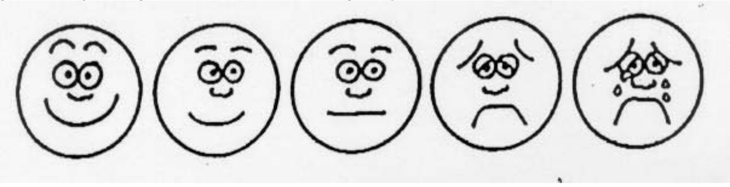

25 A. Kvůli nemoci si užiji méně zábavy se svými kamarády a kamarádkami.

Ano Ne

Pokud jsi odpověděl/a „ano", přejdi na otázku 25 B.

25 B. Jaký máš pocit z toho, že si kvůli nemoci užiješ méně zábavy se svými kamarády a kamarádkami? Prosím zaškrtni obličej, který nejlépe znázorňuje, jak se cítíš.

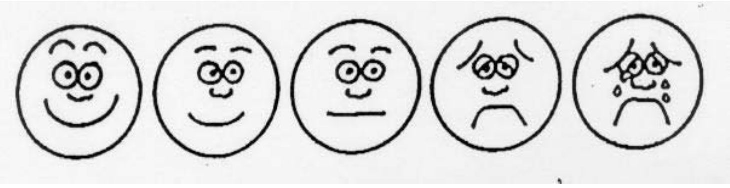

26 A. Kvůli nemoci je pro mě obtížnější se zúčastnit pohybových činností (jako je například tělocvik ve škole, běhání, chůze, oblíbená hra venku).

Ano Ne

Pokud jsi odpověděl/a „ano", přejdi na otázku 26 B.

26 B. Jak se cítíš, že kvůli nemoci je pro tebe obtížnější se zúčastnit pohybových činností (jako je například tělocvik ve škole, běhání, chůze, oblíbená hra venku)? Prosím zaškrtni obličej, který nejlépe znázorňuje, jak se cítíš.

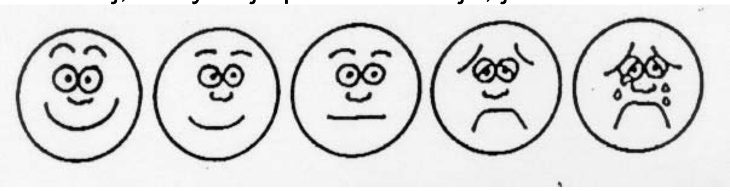

Copyright © 2007 Lakshmi Nandini Moorthy. Všechna práva vyhrazena.

Copyright © 2007 Lakshmi Nandini Moorthy. Všechna práva vyhrazena.

SIMPLE MEASURE OF IMPACT OF ILLNESS IN YOUNGSTERS (SMILY-Illness) – Børnerapport

Danish

Nummer __ Dato __/__/20

I dette spørgeskema har vi brugt de 5 ansigter nedenfor fra ”Wong-Baker FACES Pain Rating Scale”. Disse er blevet modificeret med tilladelse af D.L.Wong og de 5 ansigter vil være tilstede under hvert spørgsmål.

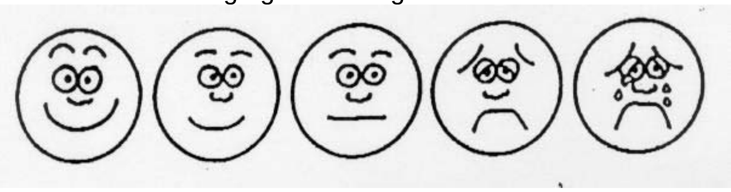

From Wong DL, Hockenberry-Eaton M, Wilson D,Winklestein ML, Schwartz P.: Wong's Essentials of Pediatric Nursing, ed. 6, St. Louis, 2001, Mosby, p 1301. Copyright Mosby. Reprinted with Permission.

SMILY-illness er copyrightbeskyttet af L. Nandini Moorthy, MD MS, RWJMS-UMDNJ, New Brunswick, NJ. Spørgeskemaet må på nuværende tidspunkt kun anvendes af L. Nandini Moorthy, MD MS og dennes forskningspersonale.

QUESTIONS

I dette spørgeskema vil vi stille dig mange spørgsmål om din sygdom og dine følelser. Der er ingen forkerte svar. Du skal endelig spørge os, hvis der er noget i skemaet, som du ikke forstår. Hvis du ønsker at tilføje kommentarer, kan du gøre dette på bagsiden af s. 5 og 6.

Forskellige ting påvirker dit liv og gør dig glad eller ked af det. Der er nogle ting, som du synes er vigtige for dig, som du er i stand til at gøre (eller ikke er i stand til at gøre). Alle disse forskellige ting i dit liv udgør tilsammen din livskvalitet.

1. Hvordan er din livskvalitet? Markér venligst det ansigt, som bedst viser din livskvalitet.

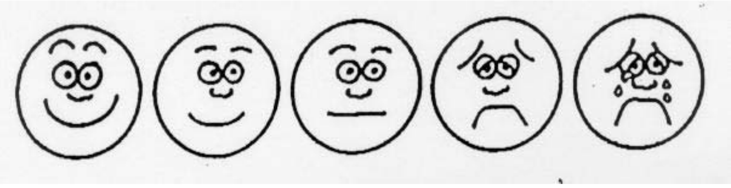

2. Hvordan er din sygdom lige nu? Markér venligst det ansigt, som bedst viser, hvordan din sygdom er lige nu.

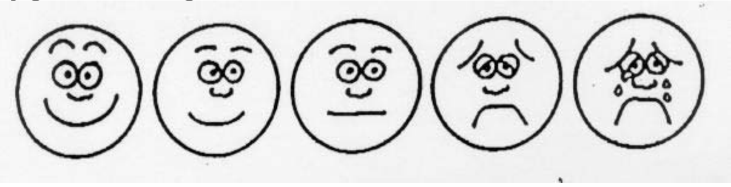

Copyright © 2007 Lakshmi Nandini Moorthy. Alle rettigheder forbeholdt.

Spørgsmål 3-19 handler om, hvordan du har haft det den sidste måned pga. din sygdom. Markér venligst det ansigt som bedst viser, hvordan du føler.

3. Hvordan har du det med at have en sygdom?

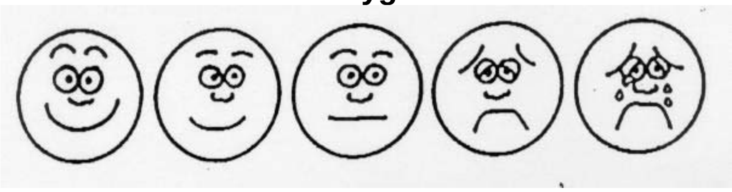

4. Hvordan påvirker din sygdom dig i forhold til at gå i skole?

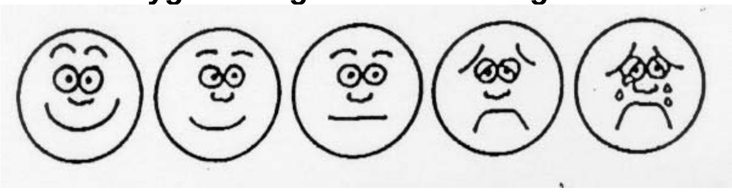

5. Hvordan påvirker din sygdom dig i forhold til at tage tøj på hver dag?

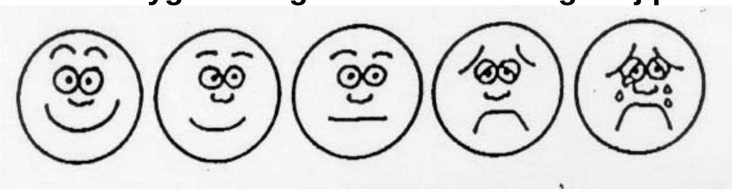

6. Hvordan påvirker din sygdom dig i forhold til dit udseende?

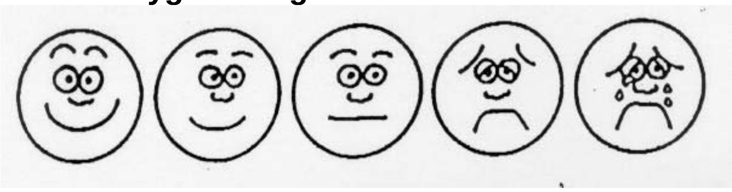

7. Hvordan påvirker din sygdom dit syn på dig selv?

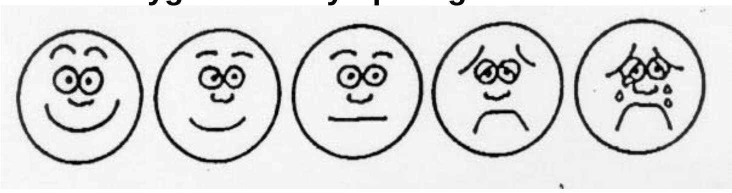

8. Hvordan føles det når du er nødt til at tale om din sygdom?

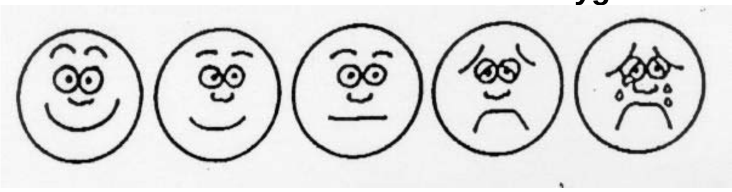

Copyright © 2007 Lakshmi Nandini Moorthy. Alle rettigheder forbeholdt.

9. Hvordan føles det når du skal til lægen pga. din sygdom?

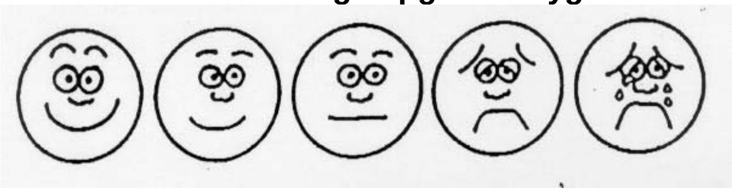

10. Hvordan føles det, når du skal på hospitalet pga. din sygdom?

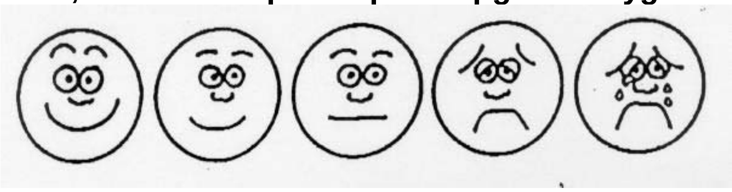

11. Hvordan får det at have en sygdom dig til at tænke på din fremtid?

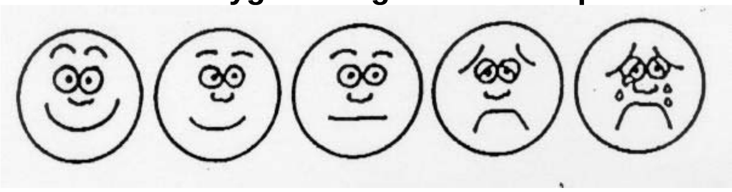

12. Hvordan har du det med at tage medicin imod din sygdom?

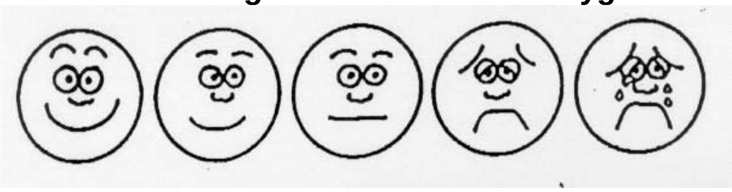

13. Hvor bekymret er du for medicinens bivirkninger?

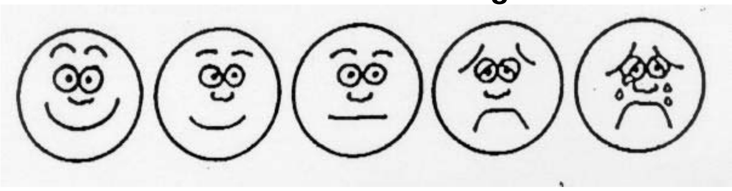

14. Hvor bekymret er du for, at din sygdom bliver værre?

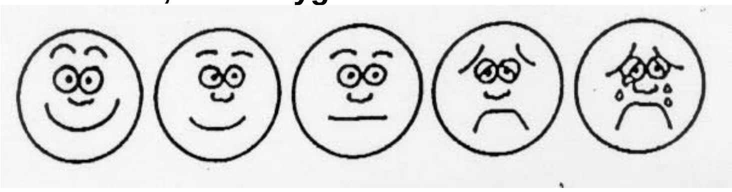

Copyright © 2007 Lakshmi Nandini Moorthy. Alle rettigheder forbeholdt.

15. Hvor ensom føler du dig pga. din sygdom?

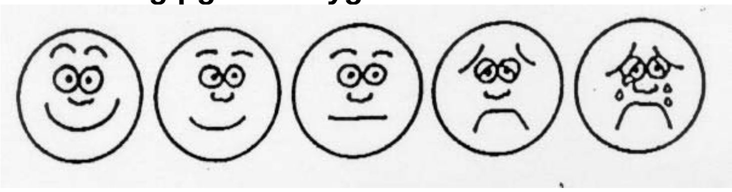

16. Hvordan påvirkes du af din familie?

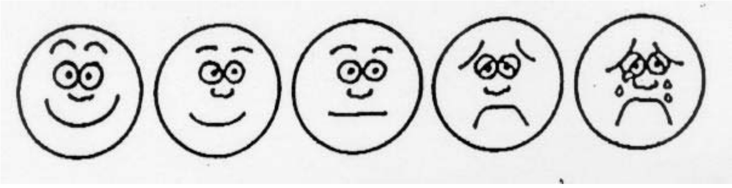

17. Hvordan påvirker smerterne fra din sygdom dig?

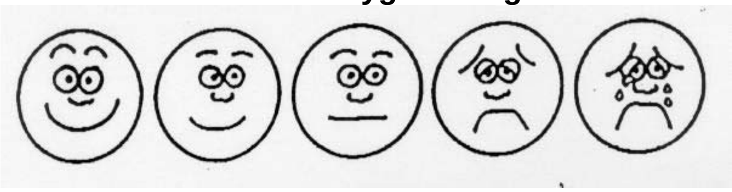

19. Hvor meget træthed mærker du pga. din sygdom?

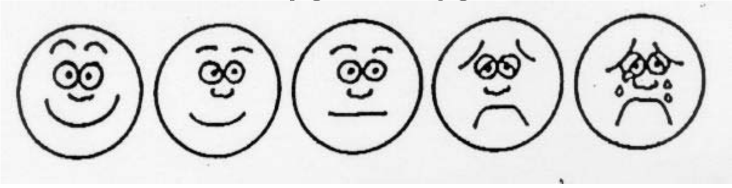

I spørgsmål 20 A – 26 A skal du svare ”ja” eller ”nej”. Derefter skal du følge instruktionerne.

Copyright © 2007 Lakshmi Nandini Moorthy. Alle rettigheder forbeholdt.

20 A. Mine venner har ændret sig pga. min sygdom. Vær venlig at sætte ring om "ja" eller "nej". Ja Nej

Hvis "ja" gå til spørgsmål 20 B.

20 B. Hvordan påvirker det at have en sygdom dit forhold til dine venner? Vær venlig at sætte ring om det ansigt, som viser, hvordan du føler.

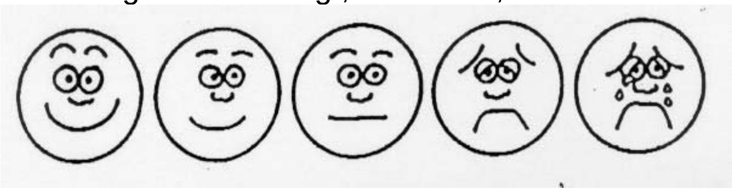

21 A. Mine venner forstår, at jeg har en sygdom. Ja Nej

Hvis du svarede "nej" gå til spørgsmål 21 B.

21 B. Hvordan føles det, at dine venner ikke forstår du har en sygdom? Vær venlig at sætte ring om det ansigt, som viser, hvordan du føler.

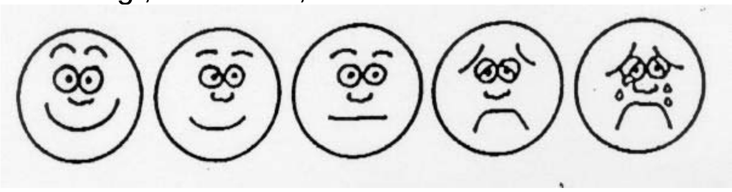

22 A. Sygdommen påvirker mit skolearbejde. Ja Nej

Hvis "ja" gå til spørgsmål 22 B.

22 B. Hvordan påvirker det at have en sygdom dit skolearbejde? Vær venlig at sætte ring om det ansigt, som viser, hvordan du føler.

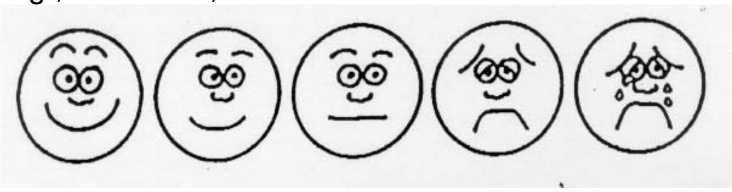

23 A. Jeg savner skolen pga. min sygdom. Ja Nej

Hvis "ja" gå til spørgsmål 23 B.

23 B. Hvordan får det at savne skolen pga. sygdommen dig til at føle? Vær venlig at sætte ring om det ansigt, som viser, hvordan du føler.

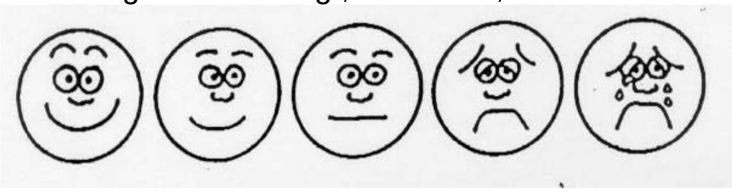

24 A. Sygdommen påvirker min aktivitet uden for skolen. Ja Nej

Hvis du har svaret "ja" gå til spørgsmål 24 B.

24 B. Hvordan føler du, at sygdommen påvirker din aktivitet uden for skolen? Vær venlig at sætte ring om det ansigt, som viser, hvordan du føler.

Copyright © 2007 Lakshmi Nandini Moorthy. Alle rettigheder forbeholdt.

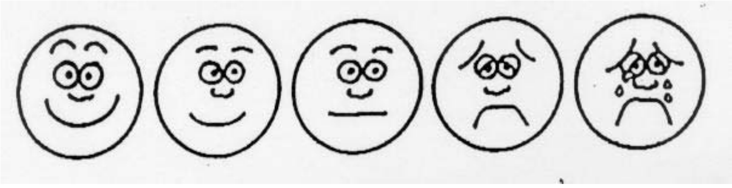

25 A. Sygdommen påvirker det sjove som jeg har med mine venner. Ja Nej

Hvis du har svaret "ja" gå til spørgsmål 25 B.

25 B. Hvordan føler du, at sygdommen påvirker det sjov, som du har med dine venner? Vær venlig at sætte ring om det ansigt, som viser, hvordan du føler.

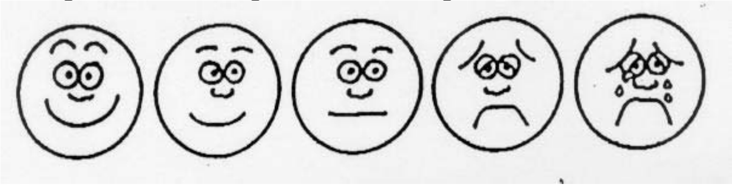

26 A. Sygdommen påvirker min deltagelse i fysiske aktiviteter (Så som gymnastik i skolen, at løbe, at gå, spille et yndlings udendørs spil).

Ja Nej

Hvis du har svaret "ja" gå til spørgsmål 26 B.

26 B. Hvordan føler du, at sygdommen påvirker din deltagelse i fysiske aktiviteter (Så som gymnastik i skolen, at løbe, at gå, spille et yndlings udendørs spil)? Vær venlig at sætte ring om det ansigt, som viser, hvordan du føler.

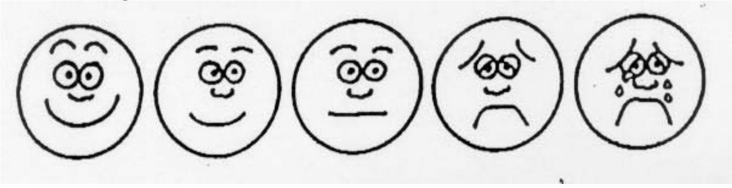

Copyright © 2007 Lakshmi Nandini Moorthy. Alle rettigheder forbeholdt.

SIMPLE MEASURE OF IMPACT OF ILLNESS IN YOUNGSTERS (SMILY-Illness) – Forældreversion

Nummer __ Dato __/__/20

I dette spørgeskema har vi brugt de 5 ansigter nedenfor fra "Wong-Baker FACES Pain Rating Scale". Disse er blevet modificeret med tilladelse af D.L.Wong og de 5 ansigter vil være tilstede under hvert spørgsmål.

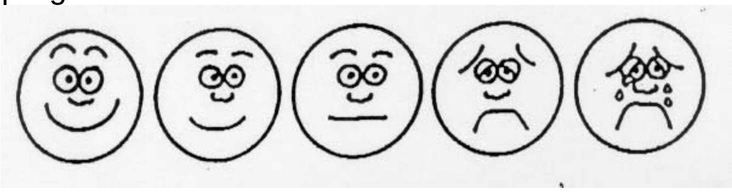

From Wong DL, Hockenberry-Eaton M, Wilson D,Winklestein ML, Schwartz P.: Wong's Essentials of Pediatric Nursing, ed. 6, St. Louis, 2001, Mosby, p 1301. Copyright Mosby. Reprinted with Permission.

SMILY-illness er copyrightbeskyttet af L. Nandini Moorthy, MD MS, RWJMS-UMDNJ, New Brunswick, NJ. Spørgeskemaet må på nuværende tidspunkt kun anvendes af L. Nandini Moorthy, MD MS og dennes forskningspersonale.

QUESTIONS

I dette spørgeskema vil vi stille dig mange spørgsmål om dit barns sygdom og følelser. Der er ingen forkerte svar. Du skal endelig spørge os, hvis der er noget i skemaet, som du ikke forstår. Hvis du ønsker at skrive nogle kommentarer, kan du gøre dette på bagsiden af s. 5 og 6.

Forskellige ting påvirker dit barns liv og gør ham/hende glad eller ked af det. Der er nogle ting, som dit barn synes er vigtige for ham/hende, som dit barn er i stand til at gøre (eller ikke er i stand til at gøre). Alle disse forskellige ting i dit barns liv udgør tilsammen dit barns livskvalitet.

1. Hvordan er dit barns livskvalitet? Markér venligst det ansigt, som bedst viser dit barns livskvalitet.

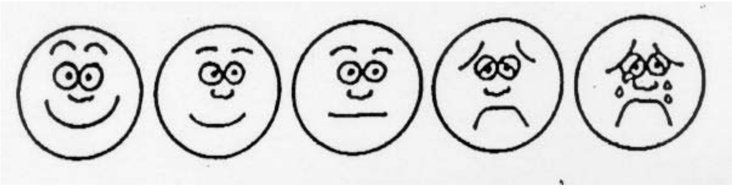

2. Hvordan er dit barns sygdom generelt lige nu? Markér venligst det ansigt, som bedst viser, hvordan dit barns sygdom er.

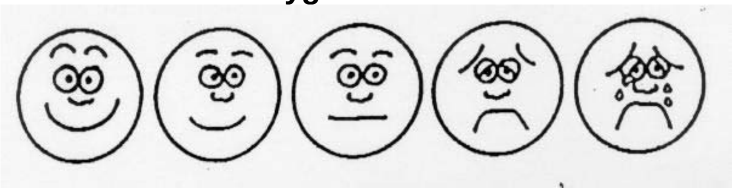

Copyright © 2007 Lakshmi Nandini Moorthy. Alle rettigheder forbeholdt.

Spørgsmål 3-19 handler om, hvordan dit barn har haft det den sidste måned pga. sygdommen. Markér venligst det ansigt, som bedst viser din respons.

3. Hvordan har dit barn det med sin sygdom?

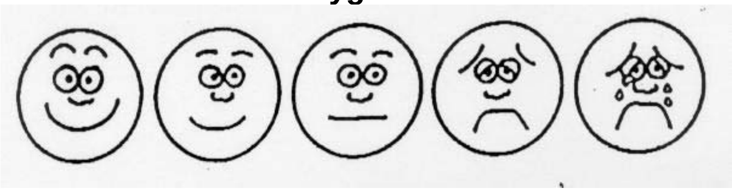

4. Hvordan påvirker sygdommen dit barns skolegang?

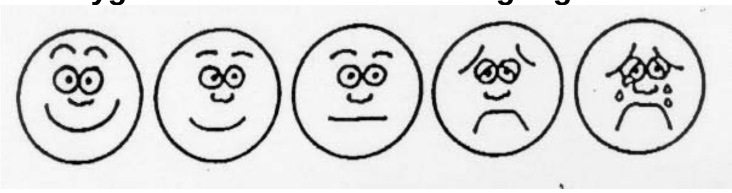

5. Hvordan påvirker sygdommen dit barns evne til at tage tøj på?

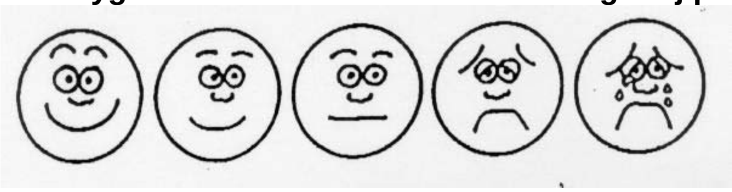

6. Hvordan påvirker sygdommen dit barns syn på sit udseende?

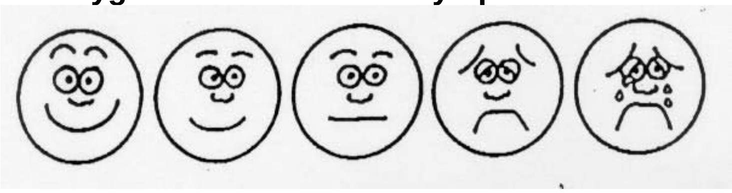

7. Hvordan påvirker sygdommen dit barns selvværd?

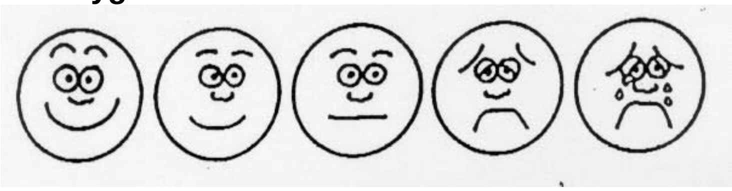

8. Hvordan har dit barn det, når han/hun skal tale om sin sygdom?

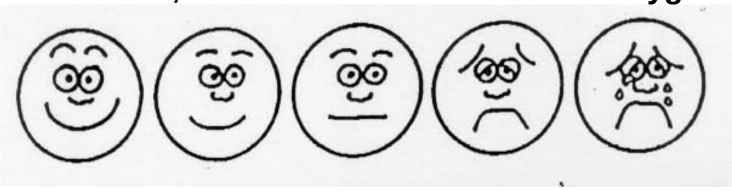

Copyright © 2007 Lakshmi Nandini Moorthy. Alle rettigheder forbeholdt.

9. Hvordan har dit barn det, når han/hun skal til lægen pga. sin sygdom?

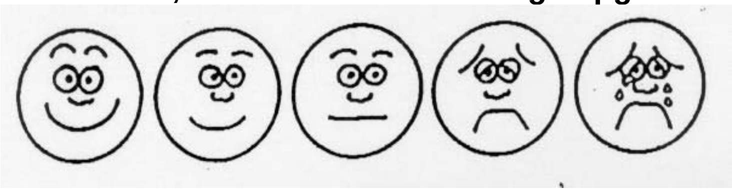

10. Hvordan har dit barn det, når han/hun skal på hospitalet pga. sin sygdom?

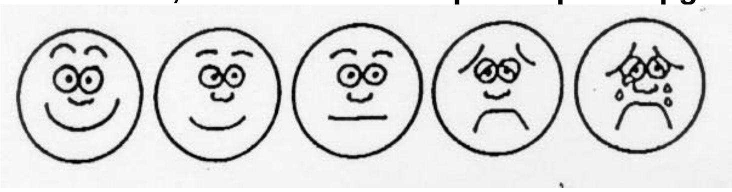

11. Hvordan får sygdommen dit barn til at føle/tænke omkring sin fremtid?

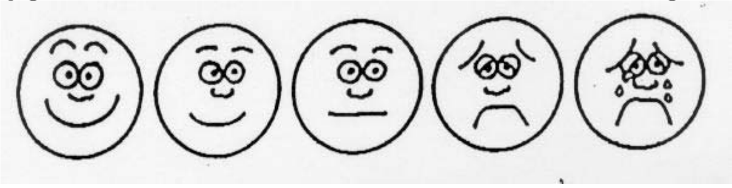

12. Hvordan har dit barn det med at tage medicin imod sygdommen?

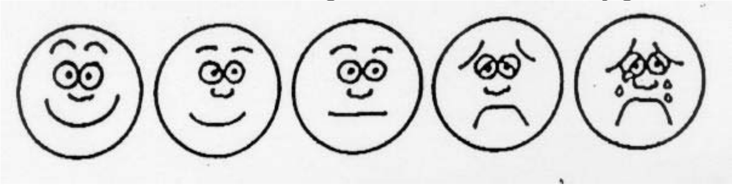

13. Hvor bekymret er dit barn for medicinens bivirkninger?

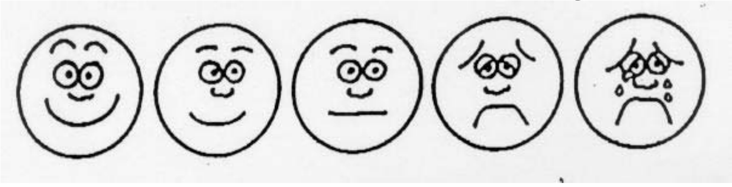

14. Hvor bekymret er dit barn for, at han/hendes sygdom bliver værre?

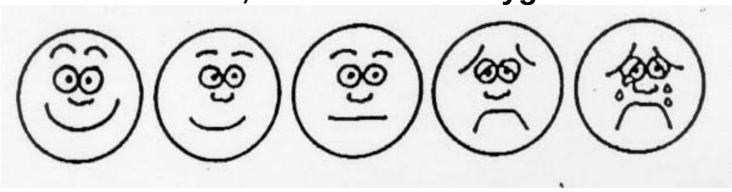

Copyright © 2007 Lakshmi Nandini Moorthy. Alle rettigheder forbeholdt.

15. Hvor ensom føler dit barn sig pga. sygdommen?

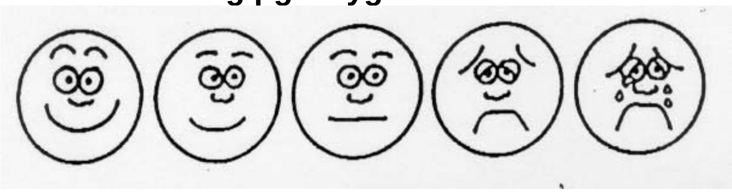

16. Hvordan påvirker den nære familie dit barns følelser?

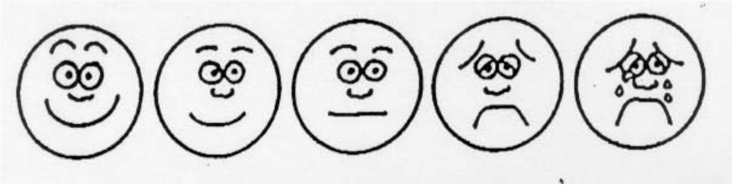

17. Hvordan påvirker smerterne fra sygdommen dit barn?

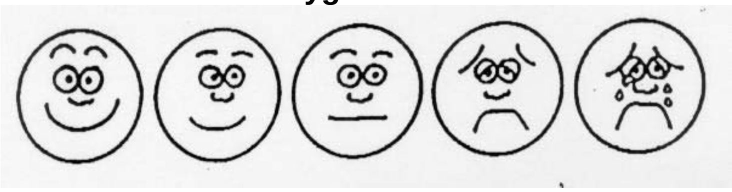

19. Hvor træt får sygdommen dit barn til at føle sig?

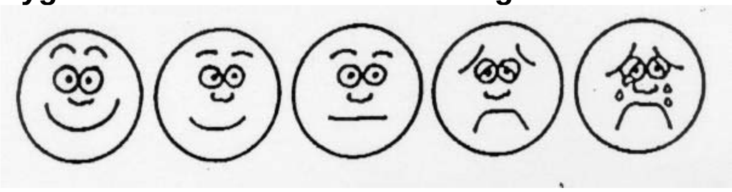

I spørgsmål 20 A – 26 A skal du svare "ja" eller "nej". Derefter skal du følge instruktionerne.

20 A. Dit barns venner har ændret sig pga. dit barns sygdom. Vær venlig at sætte ring om "ja" eller "nej". Ja Nej

Hvis "ja" gå til spørgsmål 20 B.

20 B. Hvordan har dit barn det med sine venner pga. sygdommen?

Copyright © 2007 Lakshmi Nandini Moorthy. Alle rettigheder forbeholdt.

Vær venlig at sætte ring om det ansigt, som viser, hvordan dit barn føler.

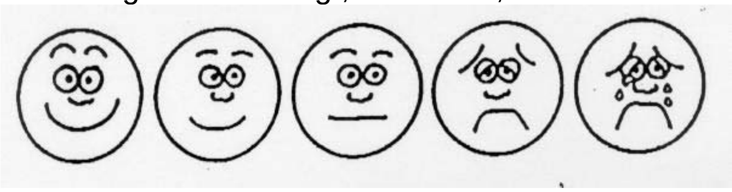

21 A. Dit barns venner forstår, at han/hun har en sygdom. Ja Nej

Hvis du svarede "nej" gå til spørgsmål 21 B.

21 B. Hvordan har dit barn det med, at vennerne ikke forstår, at han/hun har en sygdom? Vær venlig at sætte ring om det ansigt, som viser, hvordan dit barn føler.

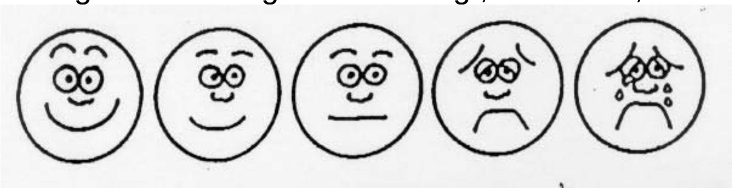

22 A. Sygdommen påvirker dit barns skolearbejde. Ja Nej

Hvis "ja" gå til spørgsmål 22 B.

22 B. Hvordan får sygdommen dit barn til at synes om sit skolearbejde? Vær venlig at sætte ring om det ansigt, som viser, hvordan dit barn føler.

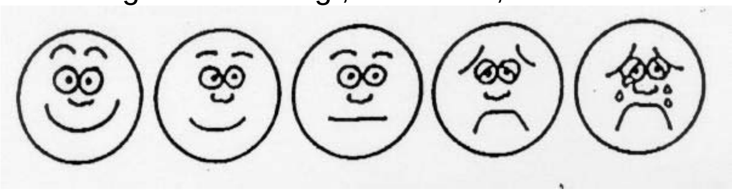

23 A. Dit barn savner skolen pga. sin sygdom. Ja Nej

Hvis "ja" gå til spørgsmål 23 B.

23 B. Hvordan har dit barn det med at savne skolen pga. sin sygdom? Vær venlig at sætte ring om det ansigt, som viser, hvordan dit barn føler.

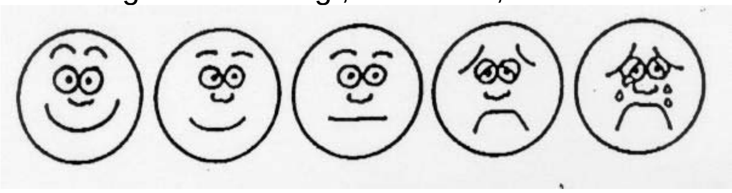

24 A. Sygdommen påvirker dit barns aktiviteter uden for skolen. Ja Nej

Hvis du har svaret "ja" gå til spørgsmål 24 B.

24 B. Hvordan påvirker sygdommen hans/hendes aktivitet uden for skolen? Vær venlig at sætte ring om det ansigt, som viser, hvordan dit barn føler.

Copyright © 2007 Lakshmi Nandini Moorthy. Alle rettigheder forbeholdt.

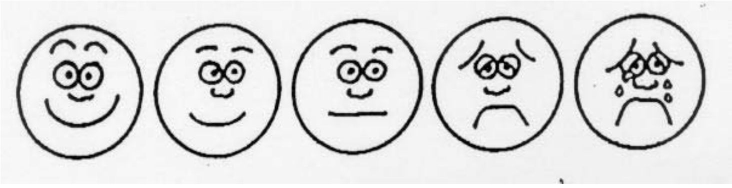

25 A. Sygdommen påvirker de sjove aktiviteter som dit barn har med sine venner. Ja Nej

Hvis du har svaret "ja" gå til spørgsmål 25 B.

25 B. På hvilken måde føler dit barn, at sygdommen påvirker de sjove aktiviteter, som han/hun har med sine venner? Vær venlig at sætte ring om det ansigt, som viser, hvordan du føler.

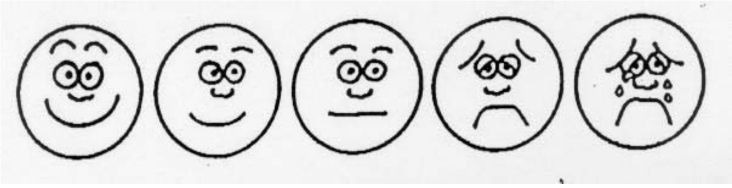

26 A. Sygdommen påvirker dit barns deltagelse i fysiske aktiviteter (Fx gymnastik i skolen, at løbe, at gå, at spille et yndlings udendørs spil).

Ja Nej

Hvis du har svaret "ja" gå til spørgsmål 26 B.

26 B. Hvordan føler dit barn at sygdommen påvirker hans/hendes deltagelse i fysiske aktiviteter (Fx gymnastik i skolen, at løbe, at gå, spille et yndlings udendørs spil)? Vær venlig at sætte ring om det ansigt, som viser, hvordan dit barn føler.

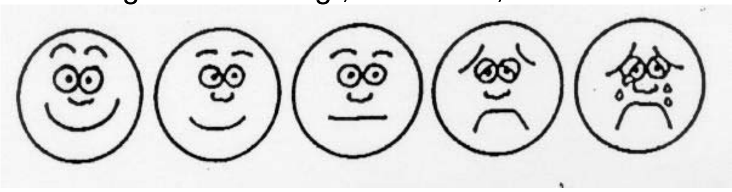

Copyright © 2007 Lakshmi Nandini Moorthy. Alle rettigheder forbeholdt.

SIMPLE MEASURE OF IMPACT OF ILLNESS IN YOUNGSTERS
Dutch **(SMILY-Illness) – Kinderformulier**

Onderzoeksnummer __ Datum __/__/20

Voor deze vragenlijst maken wij gebruik van de 5 onderstaande smileys (gezichtjes) uit de Wong-Baker FACES Pain Rating Scale, die we met toestemming van D.L. Wong hebben aangepast. Deze 5 gezichtjes verschijnen onder iedere vraag.

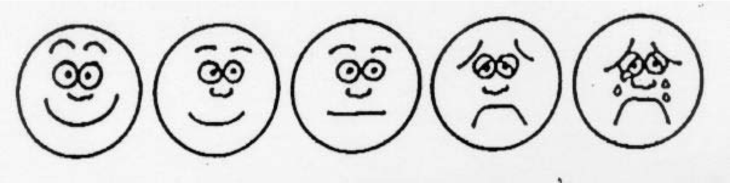

Uit: Wong DL, Hockenberry-Eaton M, Wilson D,Winklestein ML, Schwartz P.: Wong's Essentials of Pediatric Nursing, ed. 6, St. Louis, 2001, Mosby, p 1301. Copyright Mosby. Herdrukt met toestemming.

De SMILY-illness valt onder het copyright van L. Nandini Moorthy, MD MS, RWJMS-UMDNJ, New Brunswick, NJ, VS. Op dit moment mag de vragenlijst alleen worden gebruikt door L. Nandini Moorthy, MD MS en haar aangewezen onderzoeksmedewerkers.

VRAGEN

In deze vragenlijst stellen we je veel vragen over je ziekte en over je gevoelens. Er zijn geen verkeerde antwoorden. Vraag het ons gerust als je iets niet begrijpt. Als je opmerkingen wilt toevoegen, doe dit dan op de achterkant van pagina 5 en 6.

Verschillende dingen hebben invloed op je leven en maken je blij of verdrietig. Een aantal dingen die voor jou belangrijk zijn kun je wel of niet doen. Al deze verschillende dingen in je leven bepalen je kwaliteit van leven.

1. Hoe is jouw kwaliteit van leven? Omcirkel het gezichtje dat het beste jouw levenskwaliteit aangeeft.

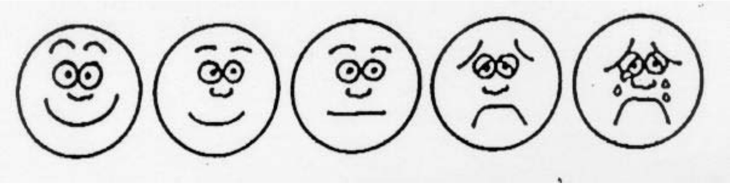

2. Hoe gaat het op dit moment met je ziekte? Omcirkel het gezichtje dat het beste aangeeft hoe het met je ziekte gaat.

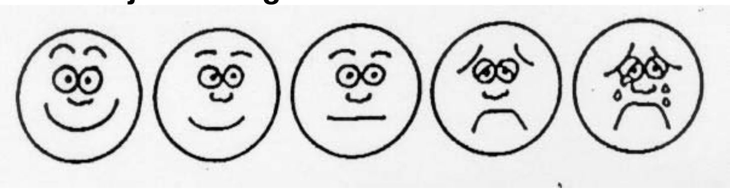

Copyright © 2007 Lakshmi Nandini Moorthy. Alle rechten voorbehouden.

Vragen 3-19 gaan over hoe je je de afgelopen maand hebt gevoeld vanwege je ziekte. Omcirkel het gezichtje dat het beste aangeeft hoe jij je voelt.

3. Hoe voelt het om een ziekte te hebben?

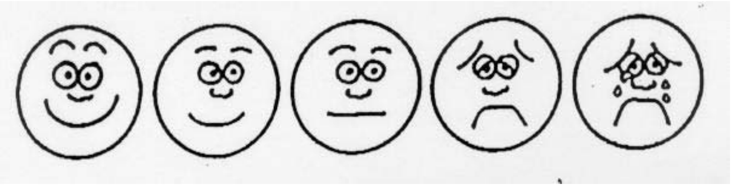

4. Hoe voelt het om naar school te gaan nu je een ziekte hebt?

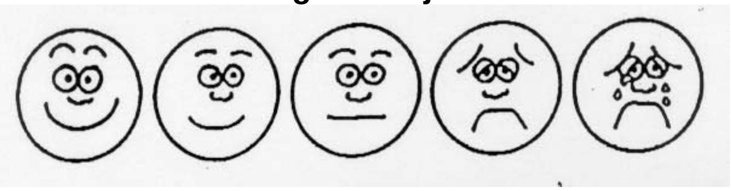

5. Hoe voelt het om je dagelijkse aan te kleden door je ziekte?

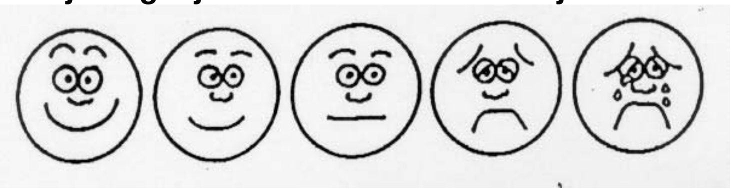

6. Hoe voel je je over je uiterlijk vanwege je ziekte?

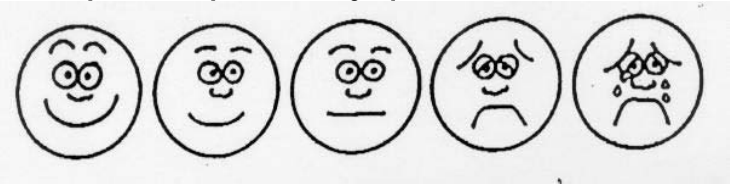

7. Hoe voel je je over jezelf vanwege je ziekte?

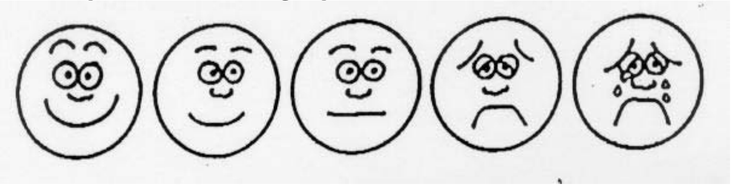

8. Hoe vind je het om over je ziekte te moeten praten?

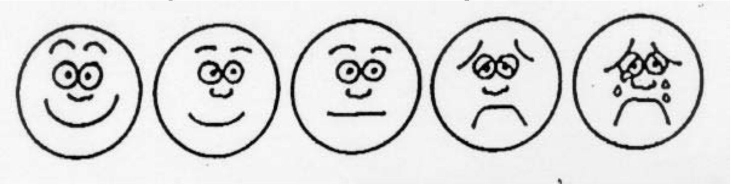

Copyright © 2007 Lakshmi Nandini Moorthy. Alle rechten voorbehouden.

9. Hoe vind je het om naar de dokter te gaan vanwege een ziekte?

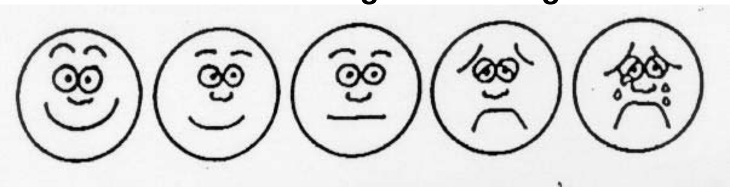

10. Hoe vind je het om naar het ziekenhuis te gaan vanwege een ziekte?

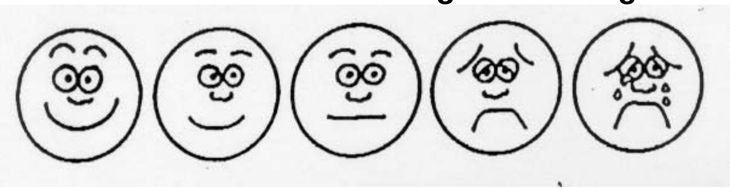

11. Hoe voel je je over je toekomst vanwege je ziekte?

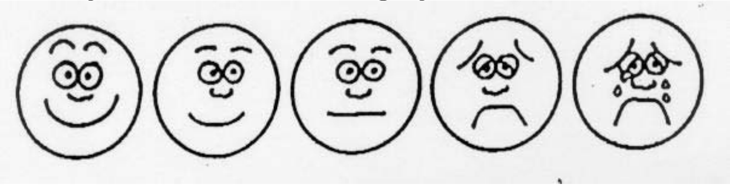

12. Hoe vind je het om medicijnen in te nemen tegen je ziekte?

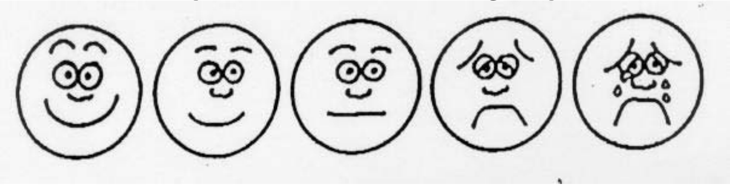

13. Hoe bezorgd ben je over de bijwerkingen van de medicijnen die je tegen je ziekte inneemt?

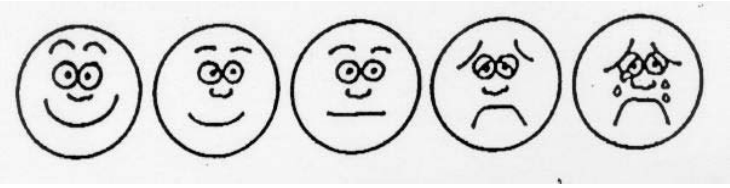

14. Hoe bezorgd ben je dat je ziekte erger wordt?

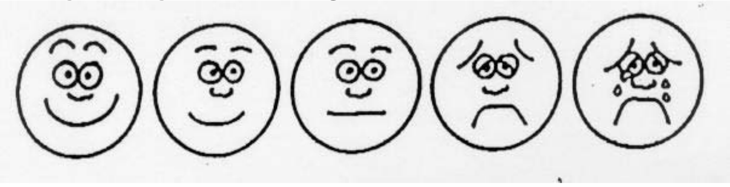

Copyright © 2007 Lakshmi Nandini Moorthy. Alle rechten voorbehouden.

15. Hoe eenzaam voel je je door je ziekte?

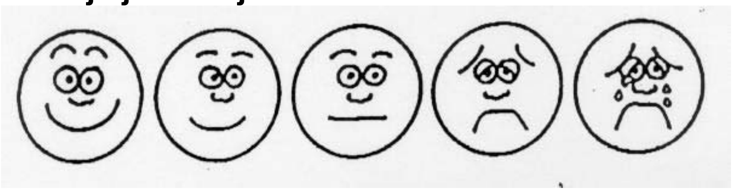

16. Welk gevoel geeft je familie je?

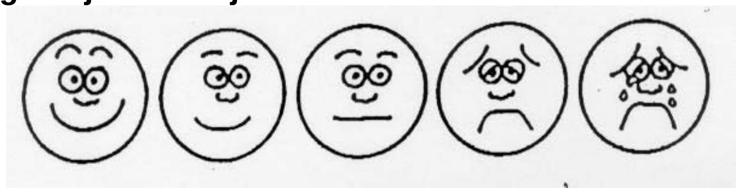

17. Hoe voel je je door de pijn van je ziekte?

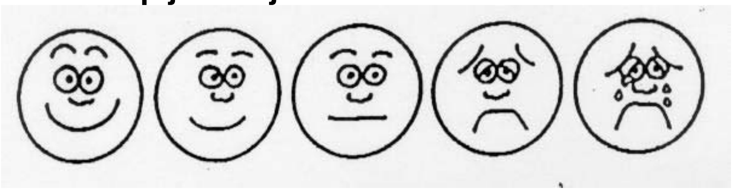

18. Wat vind je ervan dat je niet in de zon kan gaan vanwege je ziekte?

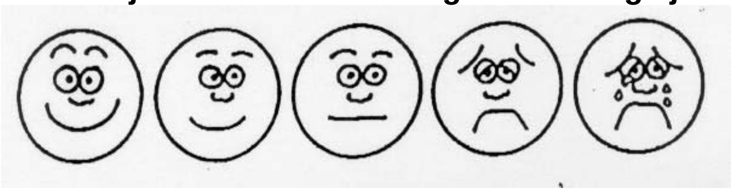

19. Hoe moe voel je je door je ziekte?

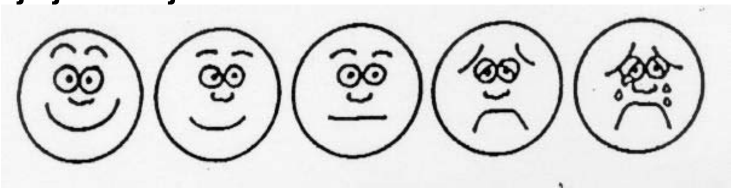

Copyright © 2007 Lakshmi Nandini Moorthy. Alle rechten voorbehouden.

Beantwoord vraag 20 A- 26 A met "ja" of "nee". Volg daarna de aanwijzingen.

20 A. Het gedrag van mijn vrienden/vriendinnen is veranderd door mijn ziekte. Omcirkel "ja" of "nee". **Ja** **Nee**

Zo "ja", ga dan naar 20 B.

20 B. Hoe voel je je over je vrienden/vriendinnen door het feit dat je een ziekte hebt? Omcirkel het gezichtje dat aangeeft hoe je je voelt.

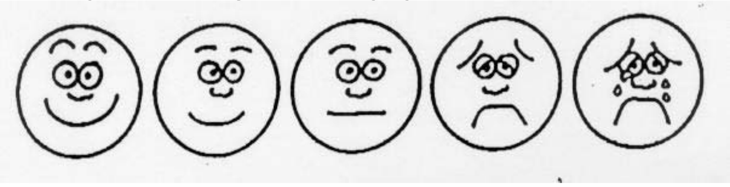

21 A. Mijn vrienden/vriendinnen begrijpen dat ik een ziekte heb. **Ja** **Nee**

Als je "nee" hebt geantwoord, ga dan naar vraag 21 B.

21 B. Wat vind je ervan dat je vrienden/vriendinnen niet begrijpen dat je een ziekte hebt? Omcirkel het gezichtje dat aangeeft hoe je je voelt.

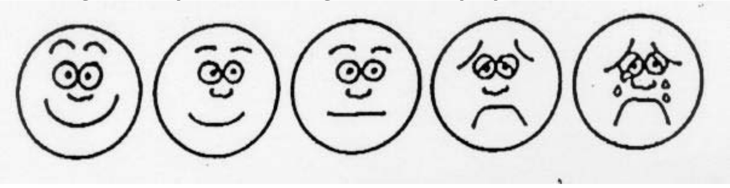

22 A. Mijn ziekre beïnvloedt mijn schoolprestaties. **Ja** **Nee**

Zo "ja", ga dan naar 22 B.

22 B. Hoe voel je je over je schoolprestaties vanwege je ziekte? Omcirkel het gezichtje dat aangeeft hoe je je voelt.

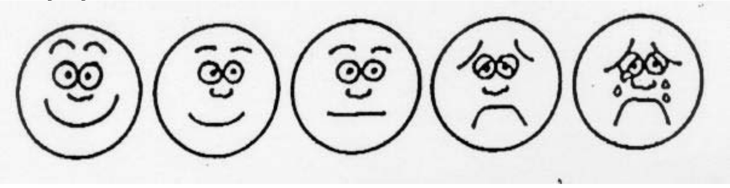

23 A. Ik verzuim school vanwege mijn ziekte. **Ja** **Nee**

Zo "ja", ga dan naar vraag 23 B.

23 B. Hoe vind het dat je school verzuimt vanwege je ziekte? Omcirkel het gezichtje dat aangeeft hoe je je voelt.

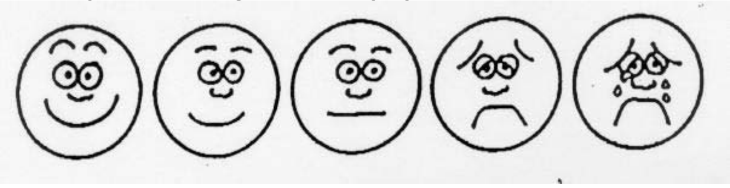

Copyright © 2007 Lakshmi Nandini Moorthy. Alle rechten voorbehouden.

24 A. Mijn ziekte beïnvloedt mijn buitenschoolse activiteiten. **Ja** **Nee**

Als je "ja" hebt geantwoord, ga dan naar vraag 24 B.

24 B. Wat vind je ervan dat je ziekte je buitenschoolse activiteiten beïnvloedt? Omcirkel het gezichtje dat aangeeft hoe je je voelt.

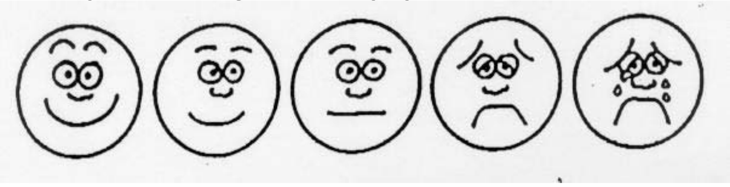

25 A. Mijn ziekte beïnvloedt het plezier dat ik met mijn vrienden/vriendinnen heb.

Ja **Nee**

Als je "ja" hebt geantwoord, ga dan naar vraag 25 B.

25 B. Wat vind je ervan dat je ziekte het plezier dat je met je vrienden/vriendinnen hebt, beïnvloedt? Omcirkel het gezichtje dat aangeeft hoe je je voelt.

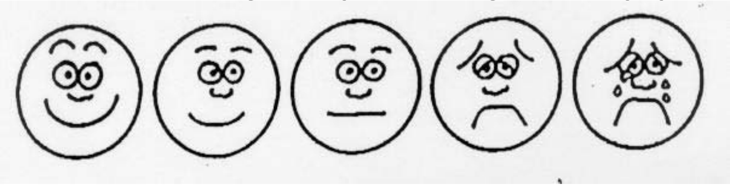

26 A. Mijn ziekte beïnvloedt mijn deelname aan lichamelijke activiteiten (zoals gymnastiek op school, rennen, lopen, een favoriete buitensport beoefenen).

Ja **Nee**

Als je "ja" hebt geantwoord, ga dan naar vraag 26 B.

26 B Wat vind je ervan dat je ziekte je deelname aan lichamelijke activiteiten (zoals gymnastiek op school, rennen, lopen, een favoriete buitensport beoefenen) beïnvloedt? Omcirkel het gezichtje dat aangeeft hoe je je voelt.

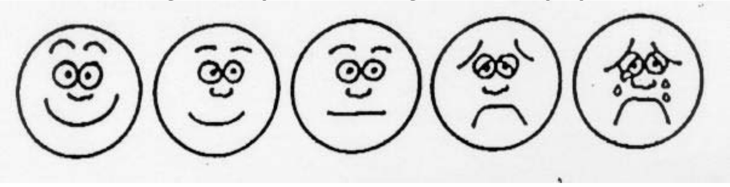

Copyright © 2007 Lakshmi Nandini Moorthy. Alle rechten voorbehouden.

SIMPLE MEASURE OF IMPACT OF ILLNESS IN YOUNGSTERS (SMILY-Illness) – Ouderversie

Onderzoeksnummer __ Datum __/__/20

Voor deze vragenlijst maken wij gebruik van de 5 onderstaande smileys (gezichtjes) uit de Wong-Baker FACES Pain Rating Scale, die we met toestemming van D.L. Wong hebben aangepast. Deze 5 gezichtjes verschijnen onder iedere vraag.

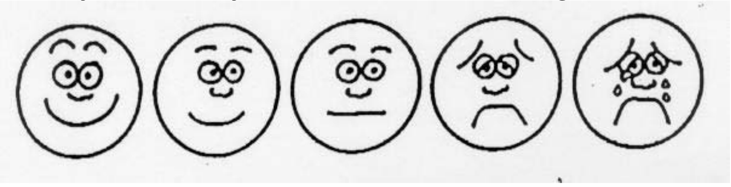

Uit: Wong DL, Hockenberry-Eaton M, Wilson D,Winklestein ML, Schwartz P.: Wong's Essentials of Pediatric Nursing, ed. 6, St. Louis, 2001, Mosby, p 1301. Copyright Mosby. Herdrukt met toestemming.

De SMILY-illness valt onder het copyright van L. Nandini Moorthy, MD MS, RWJMS-UMDNJ, New Brunswick, NJ, VS. Op dit moment mag de vragenlijst alleen worden gebruikt door L. Nandini Moorthy, MD MS en haar aangewezen onderzoeksmedewerkers.

VRAGEN

In deze vragenlijst stellen we u veel vragen over de ziekte van uw kind en de gevoelens die hij/zij daarbij heeft. Er zijn geen verkeerde antwoorden. Vraag het ons gerust als u iets niet begrijpt. Als u opmerkingen wilt toevoegen, kunt u dit doen op de achterkant van pagina 5 en 6.

Verschillende dingen hebben invloed op het leven van uw kind en maken hem/haar blij of verdrietig. Er zijn een aantal dingen die uw kind belangrijk vindt, dingen die uw kind wel of niet kan doen. Al deze verschillende dingen in het leven van uw kind bepalen de kwaliteit van leven van uw kind.

1. Hoe is de kwaliteit van leven van uw kind? Omcirkel het gezichtje dat het beste de kwaliteit van leven van uw kind aangeeft.

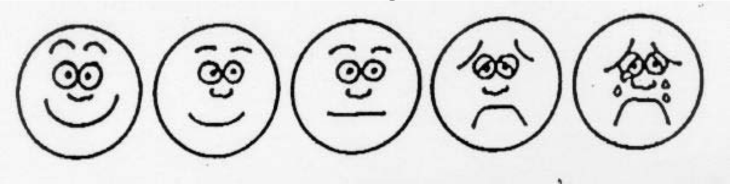

2. Hoe gaat het op dit moment met de ziekte van uw kind? Omcirkel het gezichtje dat het beste toont hoe de ziekte van uw kind is.

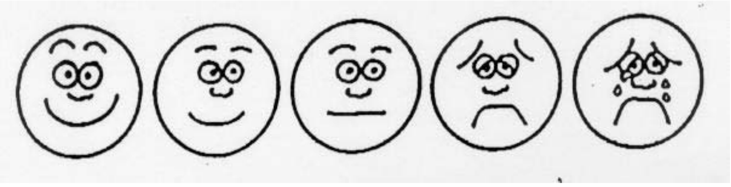

Copyright © 2007 Lakshmi Nandini Moorthy. Alle rechten voorbehouden.

Vragen 3-19 gaan over hoe uw kind zich de afgelopen maand heeft gevoeld vanwege de ziekte. Omcirkel het gezichtje dat uw antwoord het beste weergeeft.

3. Hoe voelt het voor uw kind om een ziekte te hebben?

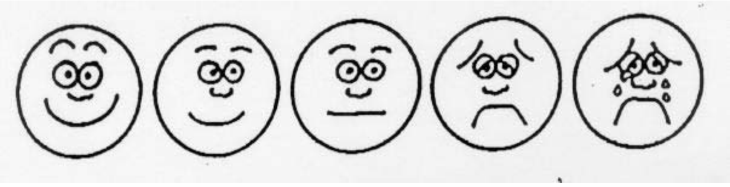

4. Hoe voelt het voor uw kind om naar school te gaan nu hij/zij een ziekte heeft?

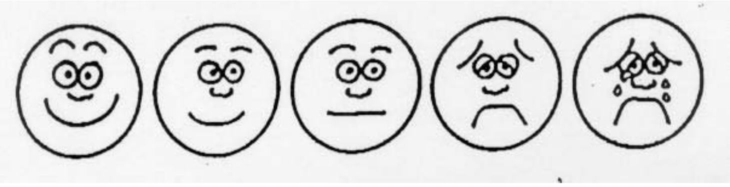

5. Hoe voelt het voor uw kind om zich dagelijks aan te kleden door het feit dat hij/zij een ziekte heeft?

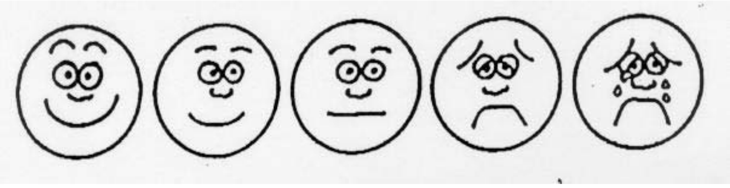

6. Hoe voelt uw kind zich over zijn/haar uiterlijk door het feit dat hij/zij een ziekte heeft?

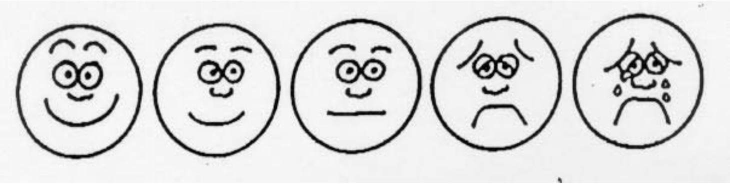

7. Hoe voelt uw kind zich over zichzelf door het feit dat hij/zij een ziekte heeft?

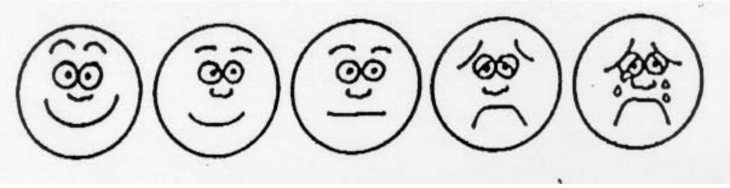

8. Hoe vindt uw kind het om over zijn/haar ziekte te moeten praten?

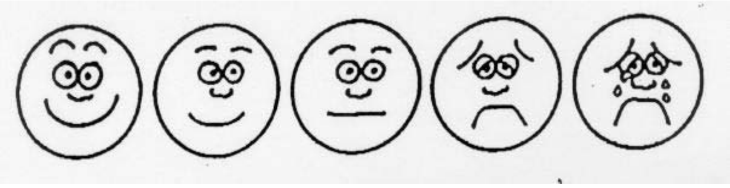

Copyright © 2007 Lakshmi Nandini Moorthy. Alle rechten voorbehouden.

9. Hoe vindt uw kind het om naar de dokter te gaan vanwege zijn/haar ziekte?

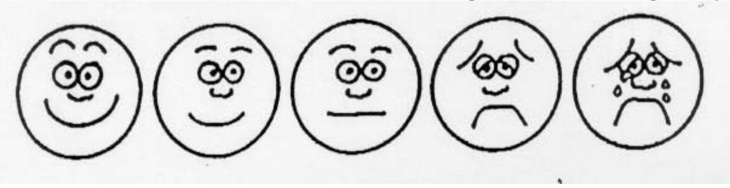

10. Hoe vindt uw kind het om naar het ziekenhuis te gaan vanwege zijn/haar ziekte?

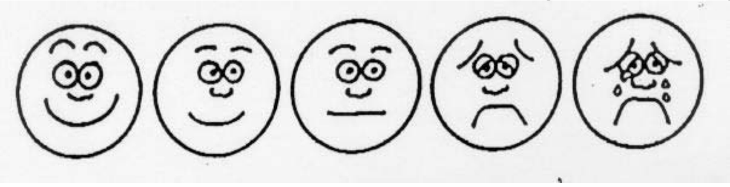

11. Hoe voelt uw kind zich met betrekking tot zijn/haar toekomst door het feit dat hij/zij een ziekte heeft?

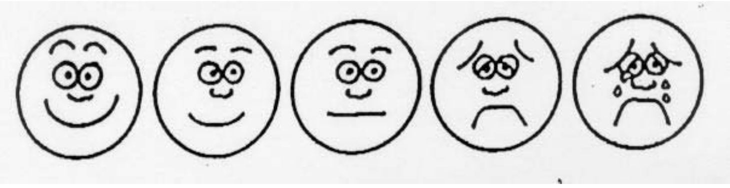

12. Hoe vindt uw kind het om medicijnen in te nemen tegen zijn/haar ziekte?

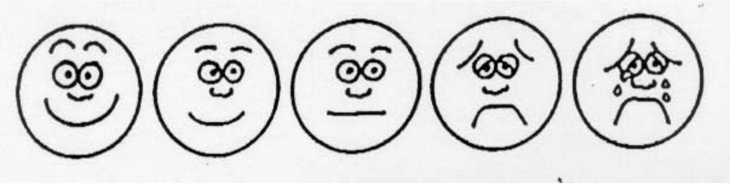

13. Hoe bezorgd is uw kind over de bijwerkingen van de medicijnen die hij/zij tegen zijn/haar ziekte inneemt?

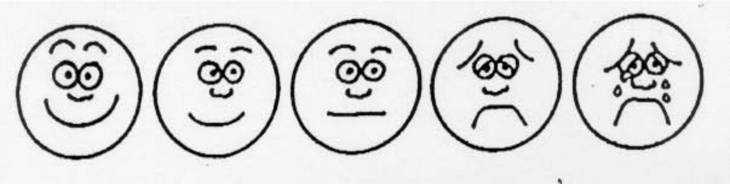

Copyright © 2007 Lakshmi Nandini Moorthy. Alle rechten voorbehouden.

14. Hoe bezorgd is uw kind dat zijn/haar ziekte erger wordt?

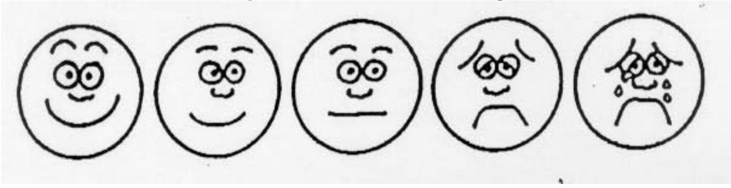

15. Hoe eenzaam voelt uw kind zich door zijn/haar ziekte?

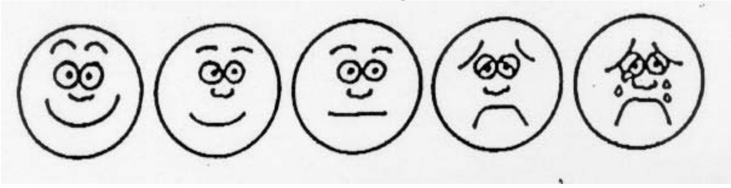

16. Welk gevoel geeft de familie uw kind?

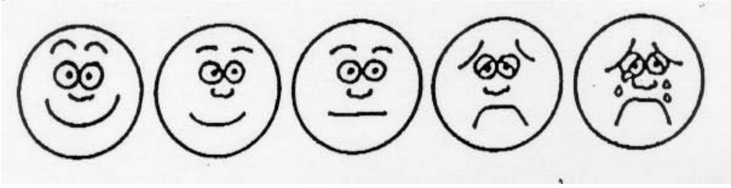

17. Hoe voelt uw kind zich door de pijn van zijn/haar ziekte?

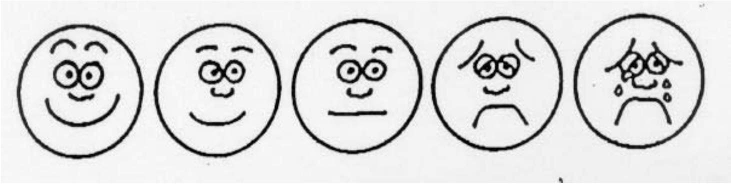

18. Wat vindt uw kind ervan dat hij/zij niet in de zon kan gaan vanwege zijn/haar ziekte?

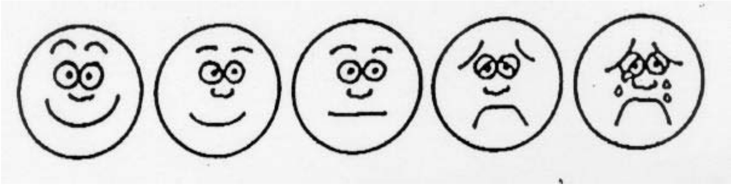

19. Hoe moe voelt uw kind zich door zijn/haar ziekte?

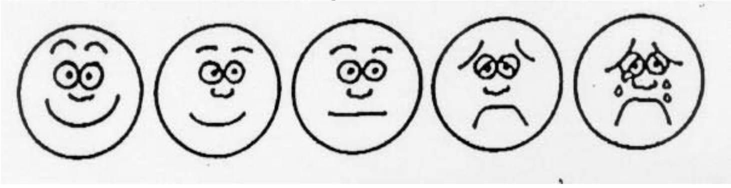

Copyright © 2007 Lakshmi Nandini Moorthy. Alle rechten voorbehouden.

Beantwoord vraag 20 A- 26 A met "ja" of "nee". Volg daarna de aanwijzingen.

20 A. Het gedrag van de vrienden/vriendinnen van uw kind is door zijn/haar ziekte veranderd. Omcirkel "Ja" of "Nee". Ja Nee

Zo "ja", ga dan naar 20 B.

20 B. Hoe voelt uw kind zich over zijn/haar vrienden/vriendinnen door het feit dat hij/zij een ziekte heeft?

Omcirkel het gezichtje dat aangeeft hoe uw kind zich voelt.

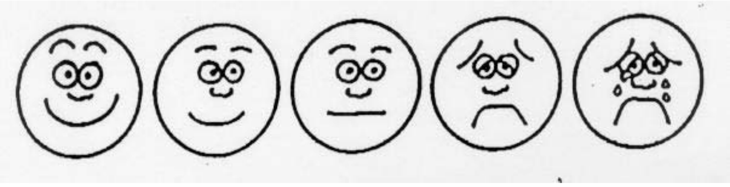

21 A. De vrienden/vriendinnen van uw kind begrijpen dat hij/zij een ziekte heeft.

Ja Nee

Als u "nee" hebt geantwoord, ga dan naar vraag 21 B.

21 B. Hoe voelt het voor uw kind dat zijn/haar vrienden niet begrijpen dat hij/zij een ziekte heeft? Omcirkel het gezichtje dat aangeeft hoe uw kind zich voelt.

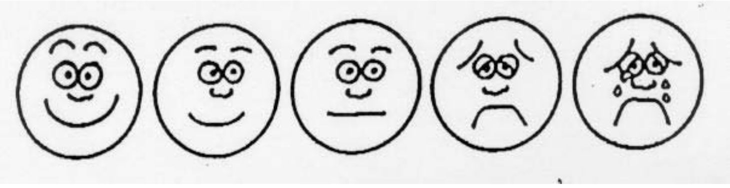

22 A. Een ziekte beïnvloedt de schoolprestaties van uw kind. Ja Nee

Zo "ja", ga dan naar 22 B.

22 B. Hoe voelt uw kind zich over zijn/haar schoolprestaties door het feit dat hij/zij een ziekte heeft?

Omcirkel het gezichtje dat aangeeft hoe uw kind zich voelt.

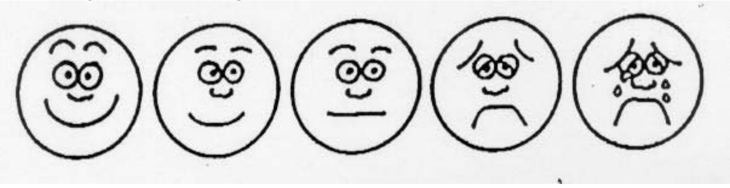

23 A. Uw kind verzuimt school vanwege zijn/haar ziekte. Ja Nee

Zo "ja", ga dan naar vraag 23 B.

23 B. Hoe vindt uw kind het dat hij/zij school verzuimt vanwege zijn/haar ziekte?

Omcirkel het gezichtje dat aangeeft hoe uw kind zich voelt.

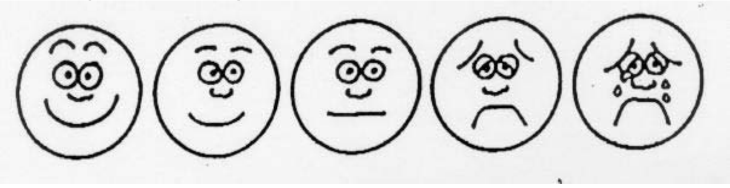

Copyright © 2007 Lakshmi Nandini Moorthy. Alle rechten voorbehouden.

24 A. De ziekte beïnvloedt de buitenschoolse activiteiten van uw kind. Ja Nee

Als u "ja" hebt geantwoord, ga dan naar vraag 24 B.

24 B. Hoe vindt uw kind het dat zijn/haar ziekte zijn/haar buitenschoolse activiteiten beïnvloedt? Omcirkel het gezichtje dat aangeeft hoe uw kind zich voelt.

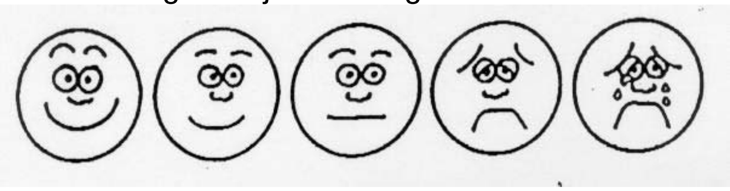

25 A. De ziekte beïnvloedt het plezier dat uw kind met zijn/haar vrienden/vriendinnen heeft.

Ja Nee

Als u "ja" hebt geantwoord, ga dan naar vraag 25 B.

25 B. Hoe vindt uw kind het dat zijn/haar ziekte het plezier dat hij/zij met zijn/haar vrienden/vriendinnen heeft, beïnvloedt? Omcirkel het gezichtje dat aangeeft hoe uw kind zich voelt.

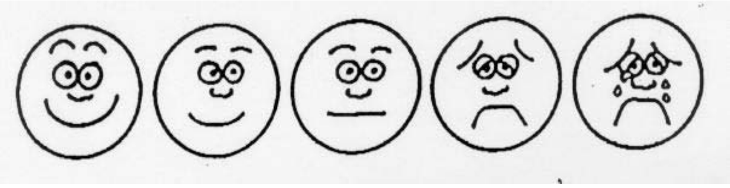

26 A. De ziekte beïnvloedt de deelname van uw kind aan lichamelijke activiteiten (zoals gymnastiek op school, rennen, lopen, een favoriete buitensport beoefenen).

Ja Nee

Als u "ja" hebt geantwoord, ga dan naar vraag 26 B.

26 B. Hoe vindt uw kind het dat zijn/haar ziekte zijn/haar deelname aan lichamelijke activiteiten (zoals gymnastiek op school, rennen, lopen, een favoriete buitensport beoefenen) beïnvloedt? Omcirkel het gezichtje dat aangeeft hoe uw kind zich voelt.

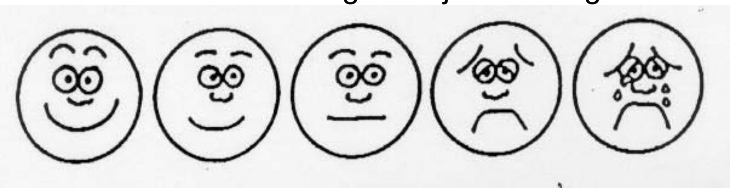

Copyright © 2007 Lakshmi Nandini Moorthy. Alle rechten voorbehouden.

SIMPLE MEASURE OF IMPACT OF ILLNESS IN YOUNGSTERS (SMILY-Illness) – Parent version

Study Number __ Date __/__/20 (UK VERSION)

For this questionnaire, we have taken the below 5 faces from the Wong-Baker FACES Pain Rating Scale, which have been modified with permission of D.L. Wong. These 5 faces will appear under every question.

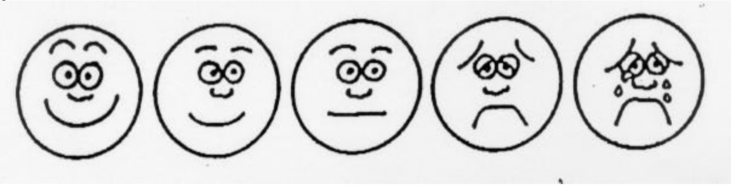

From Wong DL, Hockenberry-Eaton M, Wilson D,Winklestein ML, Schwartz P.: Wong's Essentials of Pediatric Nursing, ed. 6, St. Louis, 2001, Mosby, p 1301. Copyright Mosby. Reprinted with Permission.

The SMILY-illness is copyrighted by L. Nandini Moorthy, MD MS, RWJMS-UMDNJ, New Brunswick, NJ. At this point, the questionnaire may only be used by L. Nandini Moorthy, MD MS and her designated research personnel.

QUESTIONS

In this questionnaire, we will ask you many questions about your child's illness and feelings. There are no wrong answers. Please ask us anything that you do not understand. If you wish to write any comments, please do so behind pages 5 and 6.

Different things affect your child's life and make him/her happy or sad. There are some things your child thinks is/are important for him/her, which your child is able to do (or not able to do). All these different things in your child's life together make up your child's quality of life.

1. How is your child's quality of life? Please circle the face that best shows your child's quality of life.

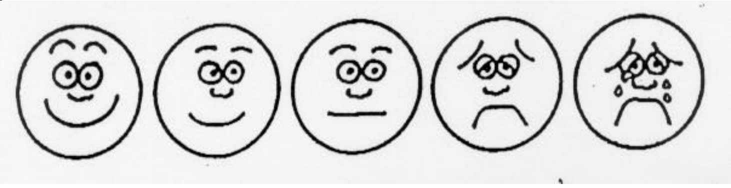

2. How is your child's illness right now? Please circle the face that best shows how your child's illness is.

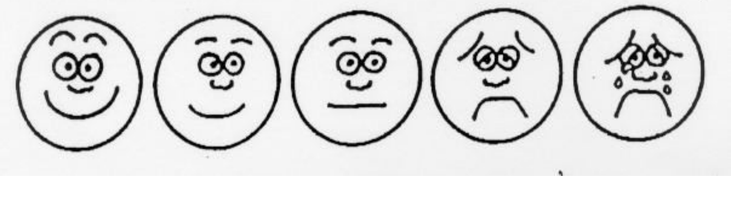

Copyright © 2007 Lakshmi Nandini Moorthy. All rights reserved.

Questions 3-19 are about how your child has been feeling over the last month because of lupus. Please circle the face that best shows your response.

3. How does having an illness make your child feel?

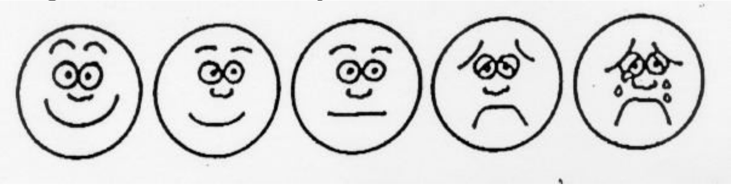

4. How does having an illness make your child feel about going to school?

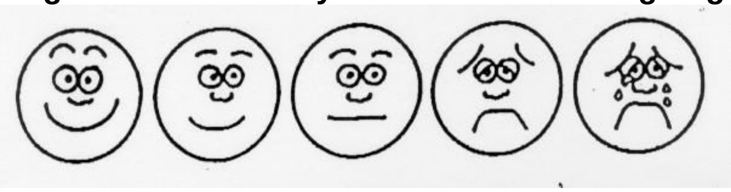

5. How does having an illness make your child feel about getting dressed everyday?

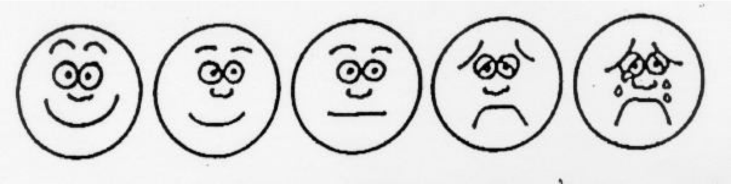

6. How does having an illness make your child feel about the way he/she looks?

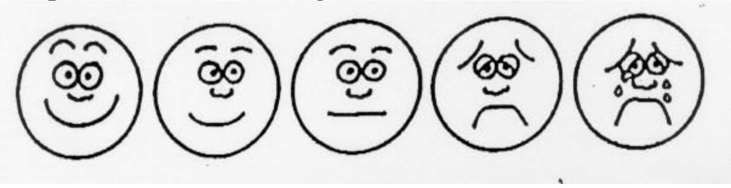

7. How does having an illness make your child feel about himself/herself?

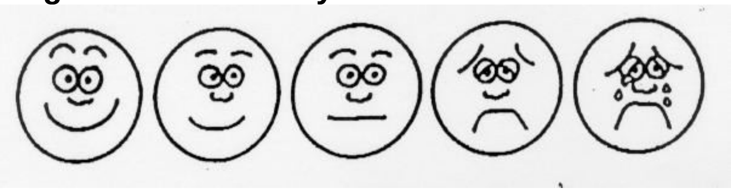

8. How does your child feel when he/she needs to talk about his/her illness?

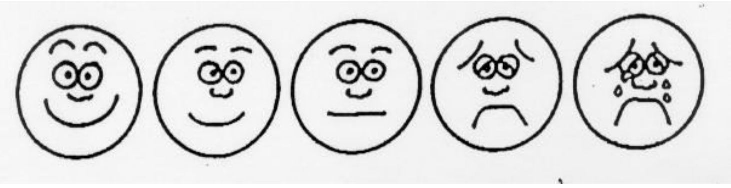

Copyright © 2007 Lakshmi Nandini Moorthy. All rights reserved.

9. How does your child feel about going to the doctor because of his/her illness?

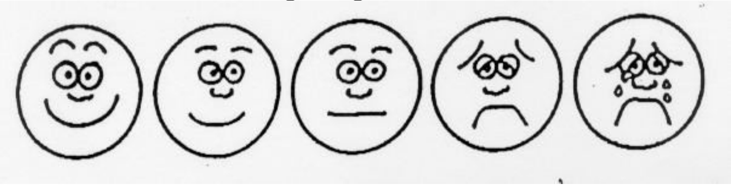

10. How does your child feel about going to the hospital because of his/her illness?

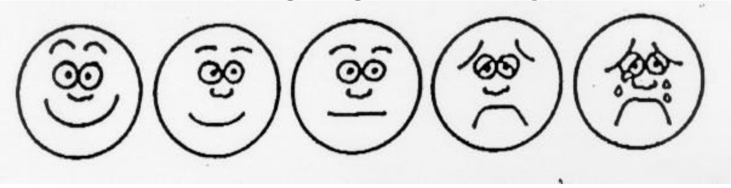

11. How does having an illness make your child feel about his/her future?

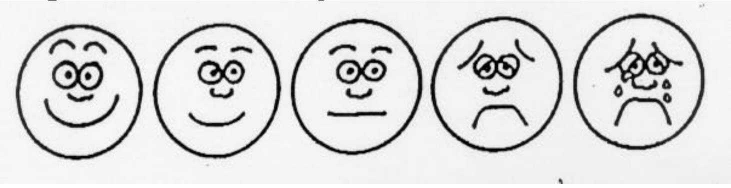

12. How does your child feel about taking medication for his/her illness?

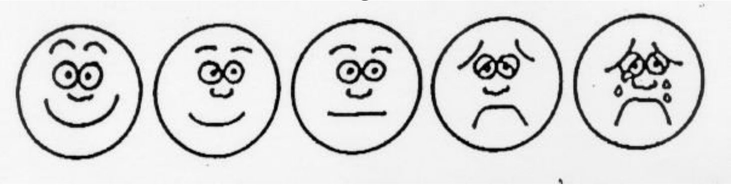

13. How worried is your child about the side effects of the medication he/she takes for his/her illness?

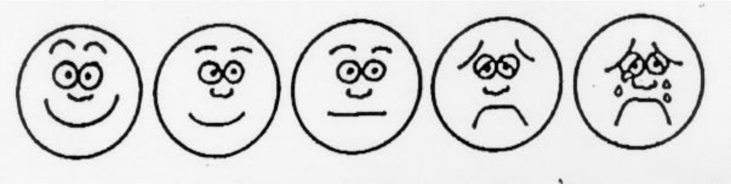

14. How worried is your child about his/her illness getting worse?

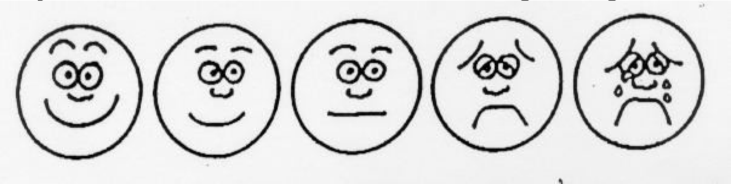

Copyright © 2007 Lakshmi Nandini Moorthy. All rights reserved.

15. How lonely does your child feel because of his/her illness?

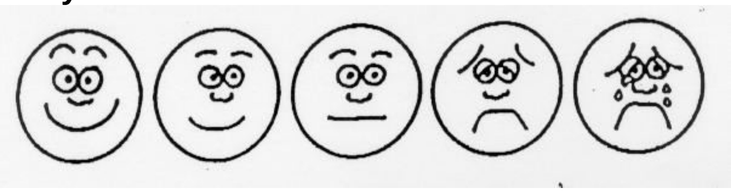

16. How does your child's family make your child feel?

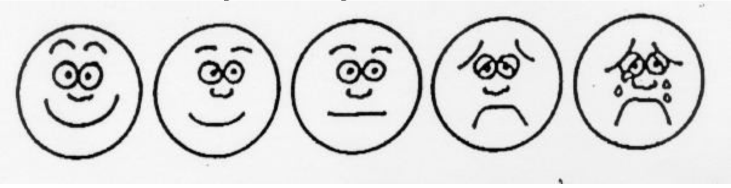

17. How does the pain of his/her illness make your child feel?

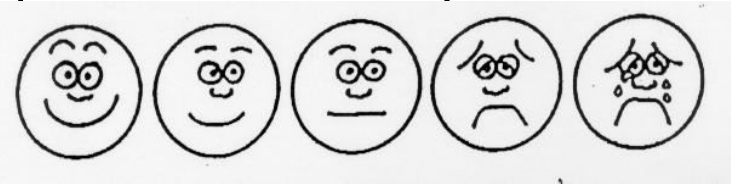

18. How does your child feel about not being able to go out in the sun because of his/her illness?

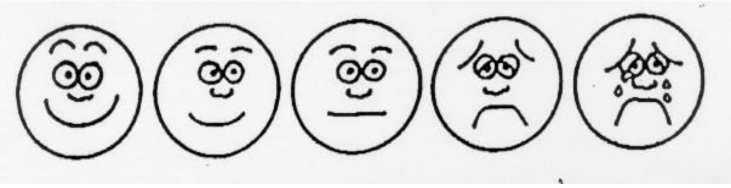

19. How tired does his/her illness make your child feel?

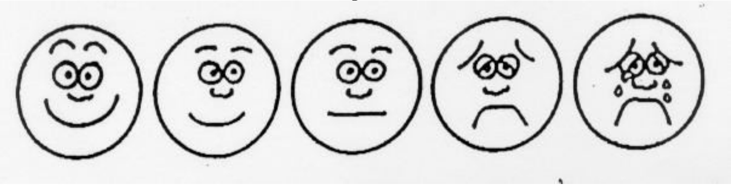

Copyright © 2007 Lakshmi Nandini Moorthy. All rights reserved.

For questions 20 A- 26 A, answer “yes” or “no.” Then, please follow instructions.

20 A. Your child’s friends have changed because of your child’s illness. Please circle “yes” or “no.” **Yes** **No**

If “yes”, go to 20 B.

20 B. How does having an illness make your child feel about his/her friends? Please circle the face that shows how your child feels.

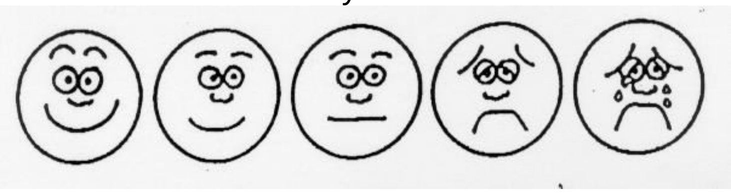

21 A. Your child’s friends understand that he/she has an illness. **Yes** **No**

If you have answered “no”, go to question 21 B.

21 B. How does your child feel about his/her friends not understanding that he/she has an illness? Please circle the face that shows how your child feels.

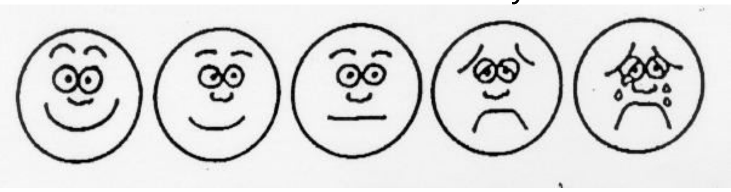

22 A. An illness affects your child’s schoolwork. **Yes** **No**

If “yes” go to 22 B

22 B. How does having an illness make your child feel about his/her schoolwork? Please circle the face that shows how your child feels.

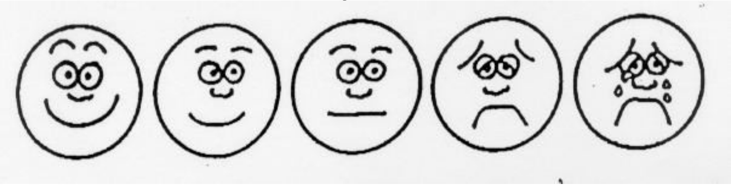

23 A. Your child misses school because of his/her illness. **Yes** **No**

If “yes”, go to question 23 B.

23 B. How does your child feel about missing school because of his/her illness? Please circle the face that shows how your child feels.

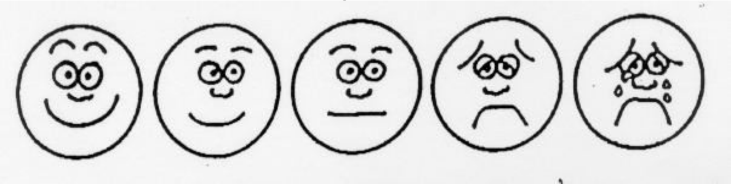

Copyright © 2007 Lakshmi Nandini Moorthy. All rights reserved.

24 A. The illness affects your child's activities outside of school. Yes No

If you have answered "yes" go to question 24 B.

24 B. How does your child feel about his/her illness affecting his/her activities outside of school? Please circle the face that shows how your child feels.

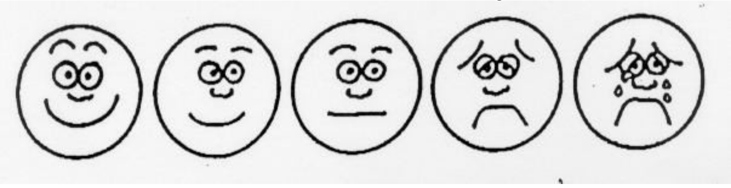

25 A. The illness affects the fun your child has with his/her friends. Yes No

If you have answered "yes" go to question 25 B.

25 B. How does your child feel about his/her illness affecting the fun he/she has with his/her friends? Please circle the face that shows how your child feels.

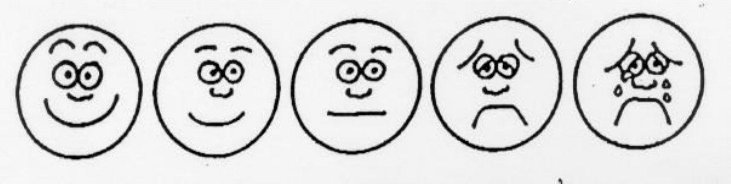

26 A. The illness affects your child's participation in physical activities (like gym activities in school, running, walking, playing a favourite outdoor game).

Yes No

If you have answered "yes" go to question 26B.

26 B. How does your child feel about his/her illness affecting his/her participation in physical activities (like gym activities in school, running, walking, playing a favourite outdoor game)? Please circle the face that shows how your child feels.

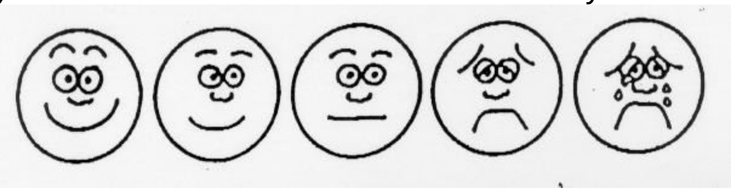

Copyright © 2007 Lakshmi Nandini Moorthy. All rights reserved.

SIMPLE MEASURE OF IMPACT OF ILLNESS IN YOUNGSTERS (SMILY-Illness) – Child report

Study Number __ Date __/__/200_

For this questionnaire, we have taken the below 5 faces from the Wong-Baker FACES Pain Rating Scale, which have been modified with permission of D.L. Wong. These 5 faces will appear under every question.

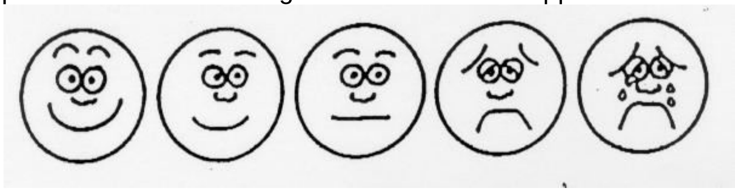

From Wong DL, Hockenberry-Eaton M, Wilson D,Winklestein ML, Schwartz P.: Wong's Essentials of Pediatric Nursing, ed. 6, St. Louis, 2001, Mosby, p 1301. Copyright Mosby. Reprinted with Permission.

The SMILY-illness is copyrighted by L. Nandini Moorthy, MD MS, RWJMS-UMDNJ, New Brunswick, NJ. At this point, the questionnaire may only be used by L. Nandini Moorthy, MD MS and her designated research personnel.

QUESTIONS

In this questionnaire, we will ask you many questions about your illness and your feelings. There are no wrong answers. Please ask us anything that you do not understand. If you wish to write any comments, please do so behind pages 5 and 6.

Different things affect your life and make you happy or sad. There are some things you think are important for you, which you are able to do (or not able to do). All these different things in your life together make up your quality of your life.

1. How is your quality of life? Please circle the face that best shows your quality of life.

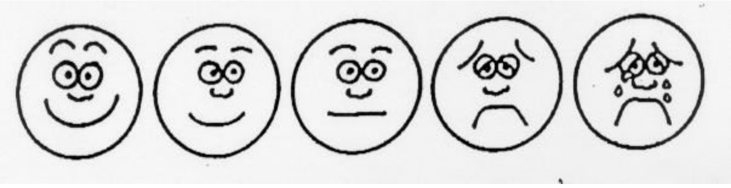

2. How is your illness right now? Please circle the face that best shows how your illness is.

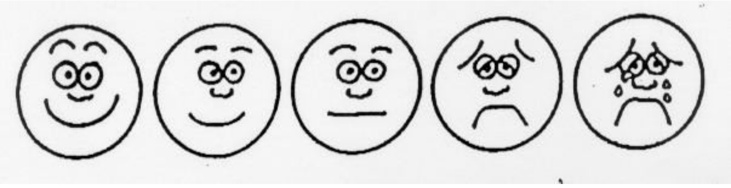

Copyright © 2007 Lakshmi Nandini Moorthy. All rights reserved.

Questions 3-19 are about how you have been feeling over the last month because of lupus. Please circle the face that best shows how you feel.

3. How does having an illness make you feel?

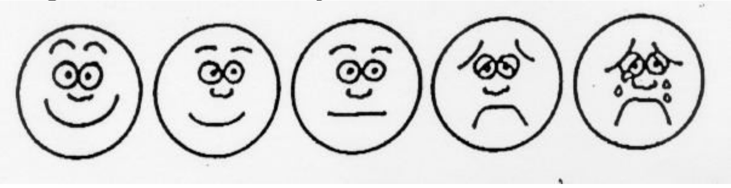

4. How does having an illness make you feel about going to school?

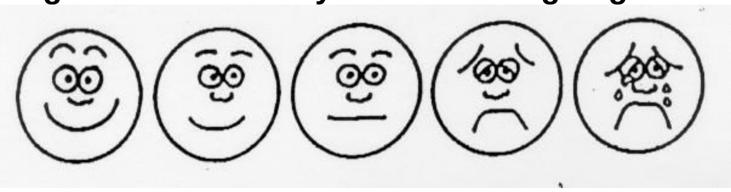

5. How does having an illness make you feel about getting dressed everyday?

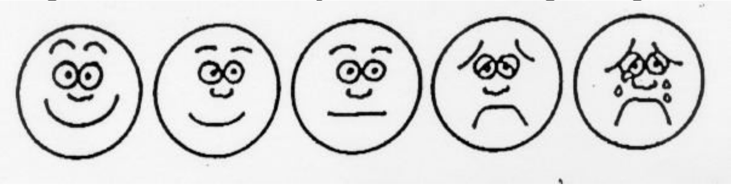

6. How does having an illness make you feel about the way you look?

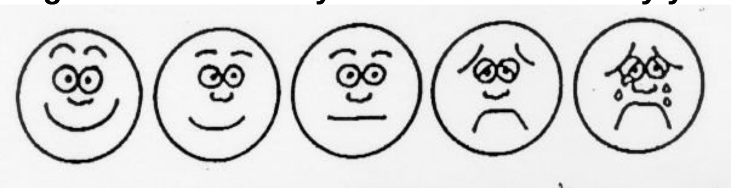

7. How does having an illness make you feel about yourself?

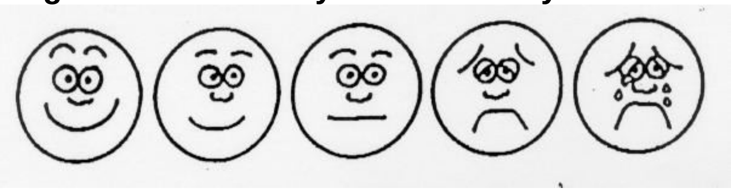

8. How do you feel when you need to talk about your illness?

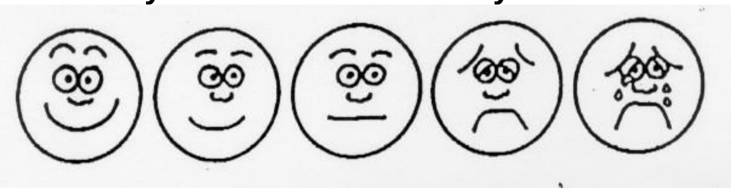

Copyright © 2007 Lakshmi Nandini Moorthy. All rights reserved.

9. How do you feel about going to the doctor because of an illness?

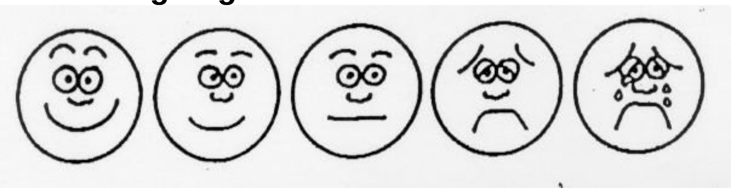

10. How do you feel about going to the hospital because of an illness?

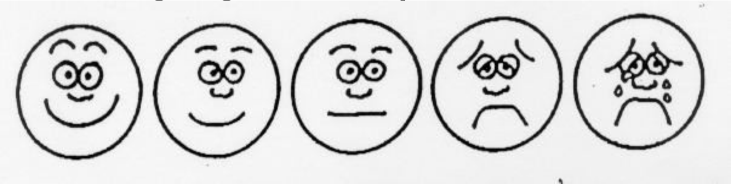

11. How does having an illness make you feel about your future?

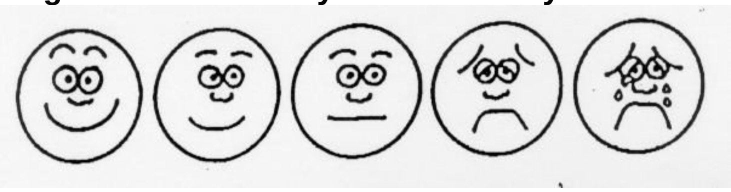

12. How do you feel about taking medications for your illness?

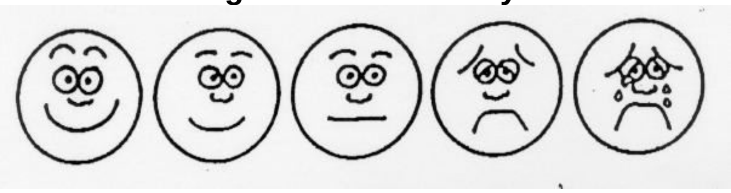

13. How worried are you about the side effects of the medications you take for your illness?

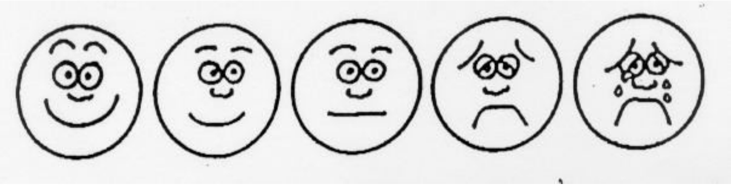

14. How worried are you about your illness pus getting worse?

Copyright © 2007 Lakshmi Nandini Moorthy. All rights reserved.

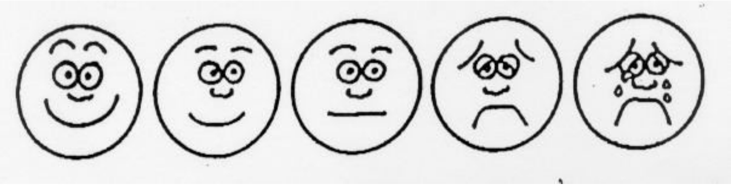

15. How lonely do you feel because of your illness?

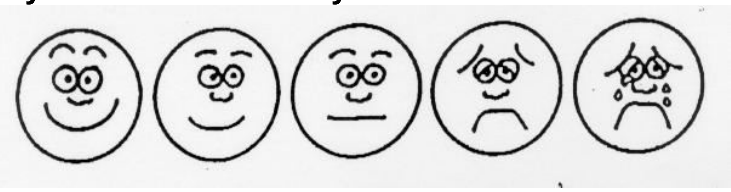

16. How does your family make you feel?

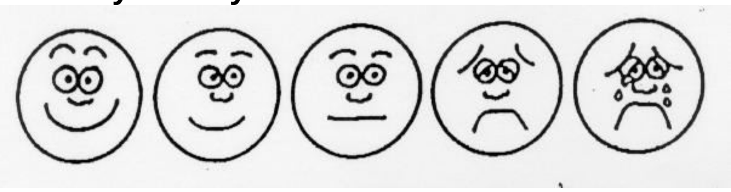

17. How does the pain of your illness make you feel?

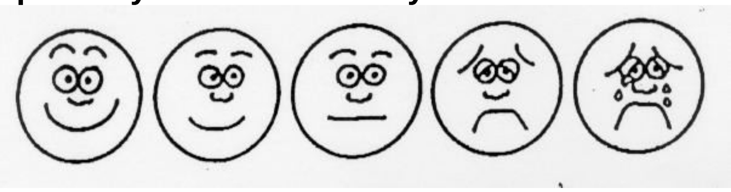

18. How do you feel about not being able to go out in the sun because of your illness?

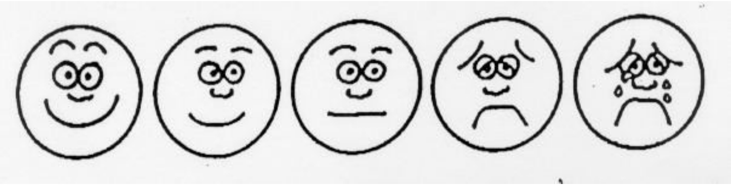

19. How tired does your illness make you feel?

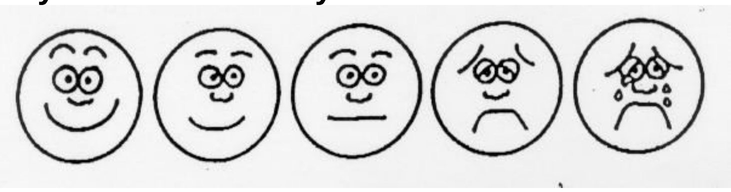

Copyright © 2007 Lakshmi Nandini Moorthy. All rights reserved.

For questions 20 A- 26 A, answer “yes” or “no.” Then, please follow instructions.

20 A. My friends have changed because of my illness. Please circle “yes” or “no.” **Yes** **No**

If “yes”, go to 20 B.

20 B. How does having lupus make you feel about your friends?
Please circle the face that shows how you feel.

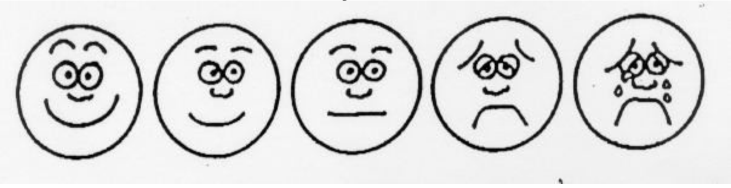

21 A. My friends understand that I have an illness **Yes** **No**

If you have answered “no”, go to question 21 B.

21 B. How do you feel about your friends not understanding that you have an illness?
Please circle the face that shows how you feel.

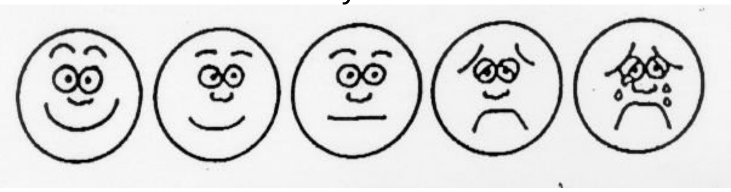

22 A. My illness affects my schoolwork. **Yes** **No**

If “yes” go to 22 B

22 B. How does having an illness make you feel about your schoolwork? Please circle the face that shows how you feel.

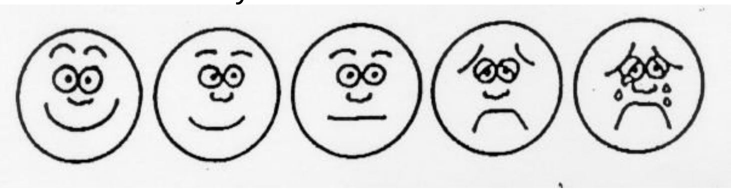

23 A. I miss school because of my illness. **Yes** **No**

If “yes”, go to question 23 B.

23 B. How do you feel about missing school because of your illness?
Please circle the face that shows how you feel.

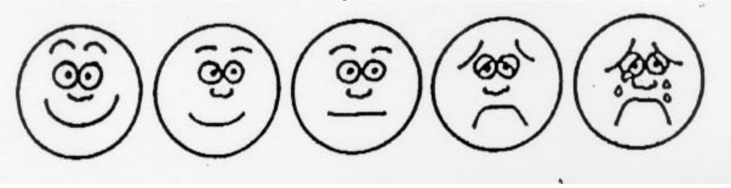

Copyright © 2007 Lakshmi Nandini Moorthy. All rights reserved.

24 A. My illness affects my activities outside of school. Yes No

If you have answered "yes" go to question 24 B.

24 B. How do you feel about your illness affecting your activities outside of school? Please circle the face that shows how you feel.

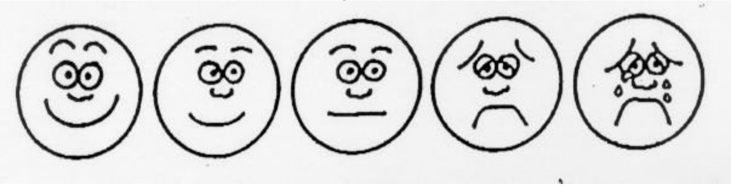

25 A. My illness affects the fun I have with my friends. Yes No

If you have answered "yes" go to question 25 B.

25 B. How do you feel about your illness affecting the fun you have with your friends? Please circle the face that shows how you feel.

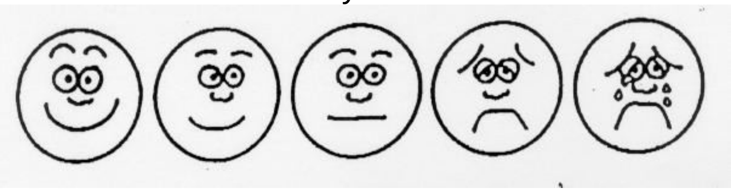

26 A. My illness affects my participation in physical activities (like gym activities in school, running, walking, playing a favourite outdoor game). Yes No

If you have answered "yes" go to question 26B.

26 B. How do you feel about your illness affecting your participation in physical activities (like gym activities in school, running, walking, playing a favourite outdoor game)? Please circle the face that shows how you feel.

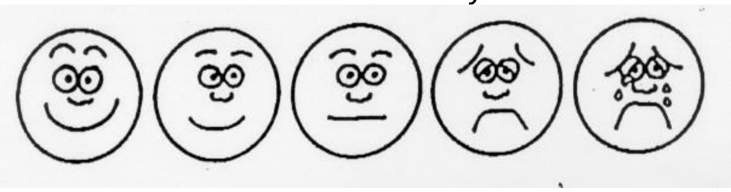

Copyright © 2007 Lakshmi Nandini Moorthy. All rights reserved.

MESURE SIMPLE DU RETENTISSEMENT DE LA MALADIE CHEZ LES JEUNES (SMILY) – Questionnaire enfant

French

Étude numéro __ Date __/__/20__

Pour ce questionnaire, nous avons pris les 5 visages de l'échelle d'évaluation de la douleur des VISAGES (FACES) de Wong-Baker, qui ont été modifiés avec la permission du Dr Wong.
Ces 5 visages apparaîtront sous chaque question.

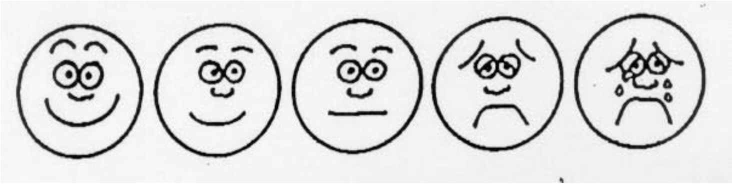

Wong DL, Hockenberry-Eaton M, Wilson D, Winklestein ML, Schwartz P.: Wong's Essentials of Pediatric Nursing, ed. 6, St. Louis, 2001, Mosby, p 1301. Copyright Mosby. Reproduit avec autorisation

The SMILY-Illness est protégé pour les droits d'auteur au nom de L. Nandini Moorthy, MD MS, RWJMS-UMDNJ, New Brunswick, NJ. Le questionnaire ne peut actuellement être utilisé que par L. Nandini Moorthy, MD MS et les personnes autorisées dans le cadre du protocole d'étude.

QUESTIONS

Dans ce questionnaire, nous te poserons beaucoup de questions sur ta maladie et comment tu la ressens Il n'y a pas de mauvaises réponses. N'hésite pas à nous demander si tu ne comprends pas quelque chose. Si tu souhaites ajouter des commentaires, tu peux le faire derrière les pages 5 et 6.

Différentes choses t'affectent dans ta vie et peuvent te rendre heureux ou triste. Il y a des choses que tu juges importantes pour toi, que tu es capable de faire (ou que tu es incapable de faire).Toutes ces différentes choses contribuent à ta qualité de vie.

1. Comment est ta qualité de vie?
Entoure le visage qui exprime le mieux ta qualité de vie.

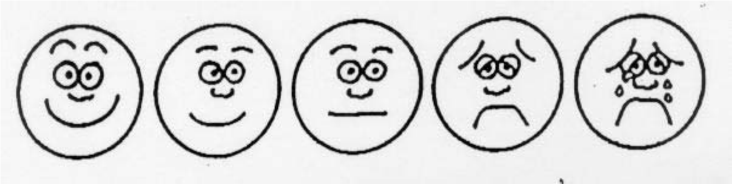

2. Comment est ta maladie en ce moment ?
Entoure le visage qui exprime le mieux comment est ta maladie.

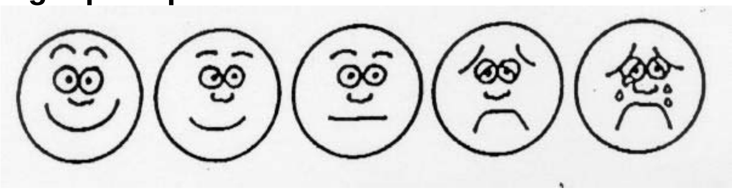

Copyright © 2007 Lakshmi Nandini Moorthy. Tous droits réservés.

Les questions 3-19 évaluent comment tu t'es senti au cours du dernier mois à cause de la maladie. Entoure le visage qui exprime le mieux comment tu te sentais.

3. Comment t'es tu senti avec la maladie?

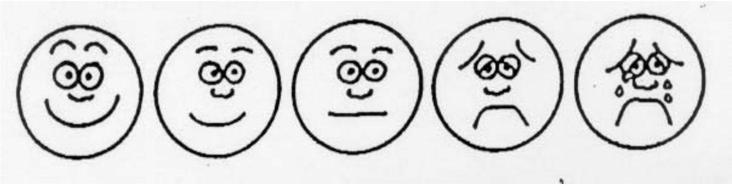

4. Comment t'es tu senti, avec la maladie, pour aller à l'école?

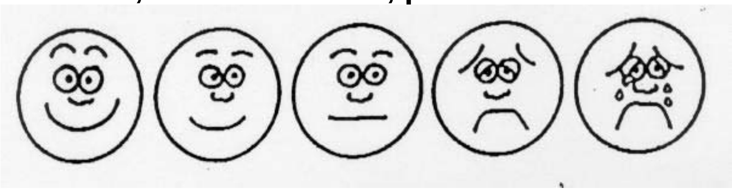

5. Comment t'es tu senti, avec la maladie, pour t´habiller chaque jour ?

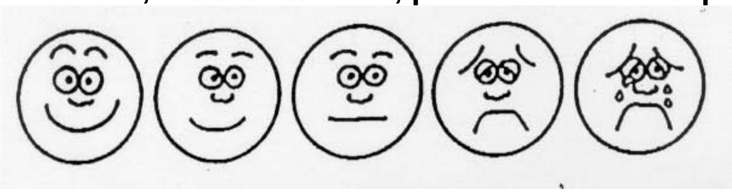

6. Comment t'es tu senti, avec la maladie, vis à vis de ton apparence (ton look) ?

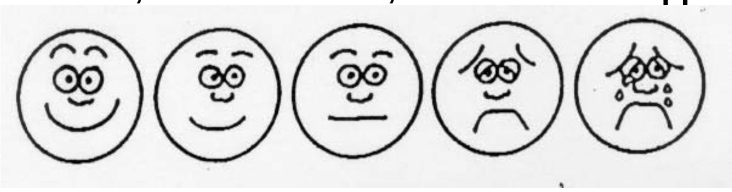

7. Comment t'es tu senti, avec la maladie, vis à vis de toi même (ton moral) ?

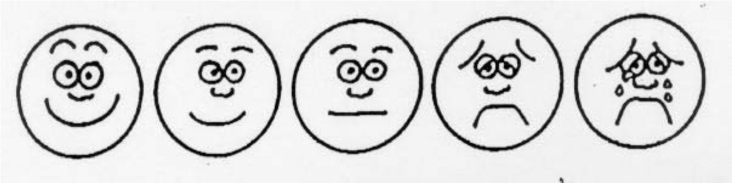

8. Comment te sens-tu quand tu as besoin de parler de ta maladie?

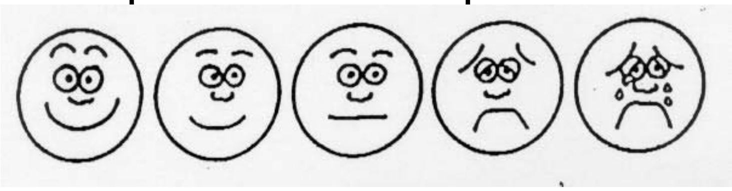

Copyright © 2007 Lakshmi Nandini Moorthy. Tous droits réservés.

9. Comment te sens-tu quand tu dois aller chez le docteur à cause de ta maladie?

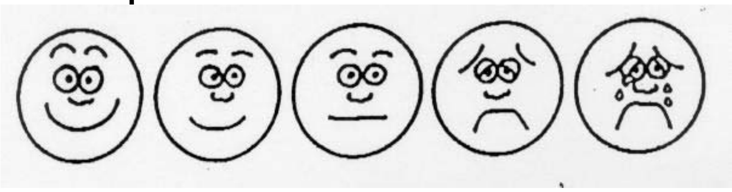

10. Comment te sens-tu quand tu dois aller à l´hôpital à cause de ta maladie?

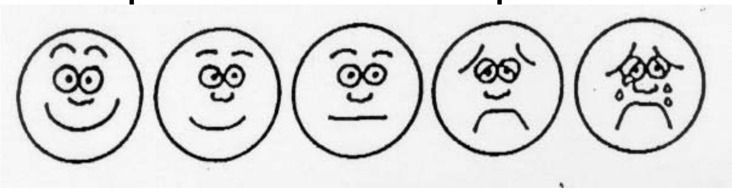

11. Comment te sens-tu avec la maladie quand tu envisages ton avenir?

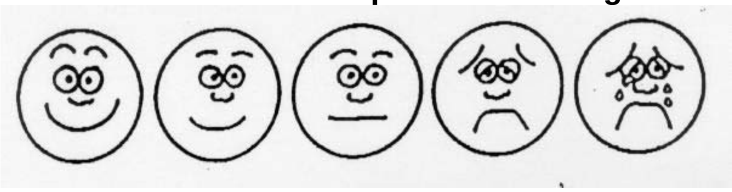

12. Comment te sens-tu vis à vis de la prise de médicaments pour la maladie ?

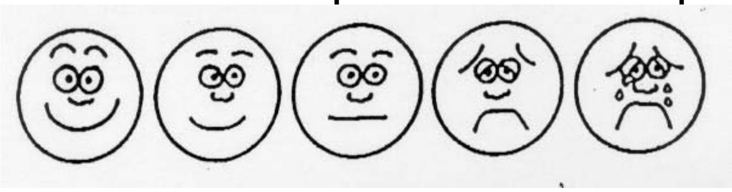

13. Est ce que tu es inquiet à propos des effets secondaires des médicaments que tu prends pour la maladie ?

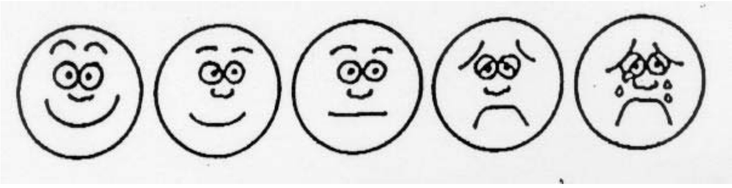

14. Est ce que tu es inquiet par rapport au risque d'aggravation de la maladie ?

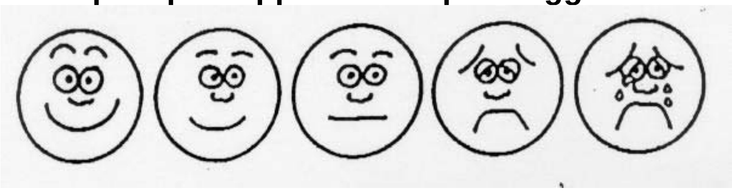

Copyright © 2007 Lakshmi Nandini Moorthy. Tous droits réservés.

15. Te sens-tu seul à cause de la maladie ?

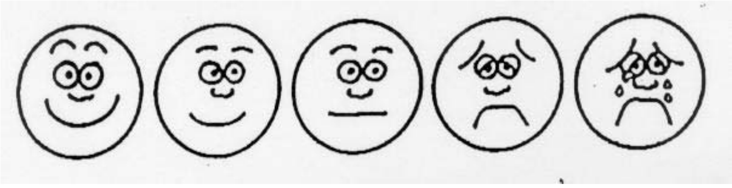

16. Comment te sens tu avec ta famille ?

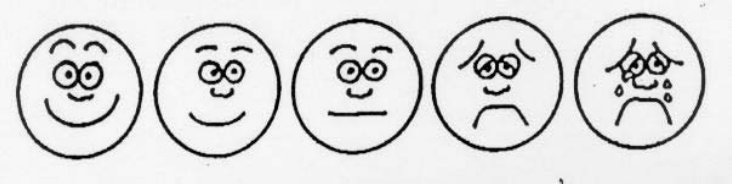

17. Comment te sens tu par rapport à la douleur causée par la maladie ?

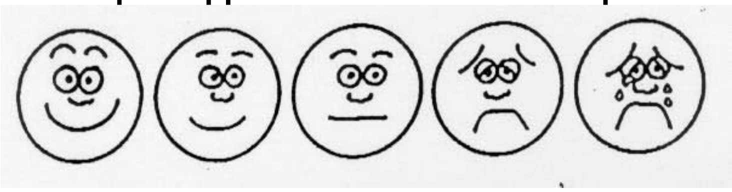

18. Comment vis tu le fait de ne pas pouvoir aller au soleil en raison de la maladie ?

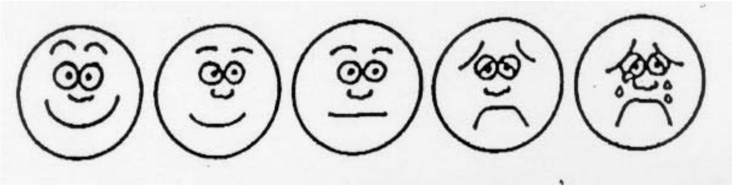

19. Comment évalues tu la fatigue liée à la maladie ?

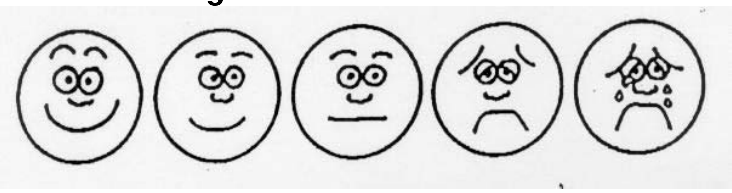

Copyright © 2007 Lakshmi Nandini Moorthy. Tous droits réservés.

Pour les questions 20 A - 26 A, répond «oui» ou «non» Puis, suis bien les instructions.

20 A. Mes amis ont changé en raison de ma maladie. Répond «oui» ou «non»
Oui **Non**

Si «oui» répond à la question 20 B.

20 B. Comment te sens-tu avec tes amis à cause de la maladie ?
Tu dois encercler le visage qui montre comment tu te sens.

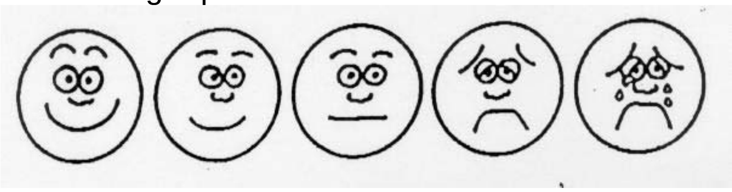

21 A. Mes amis comprennent que j´ai la maladie **Oui** **Non**

Si tu as répondu «non» passe à la question 21 B.

21 B. Comment te sens-tu si tes amis ne comprennent pas que tu as une maladie?
Encercle le visage qui montre comment tu te sens.

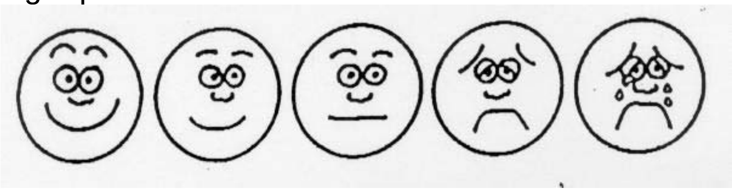

22 A. La maladie affecte mon travail scolaire. **Oui** **Non**

Si «oui» passe à la question *22 B*

22 B. Comment te sens tu pour ton travail scolaire à cause de la maladie ? Tu dois encercler le visage qui montre comment tu te sens.

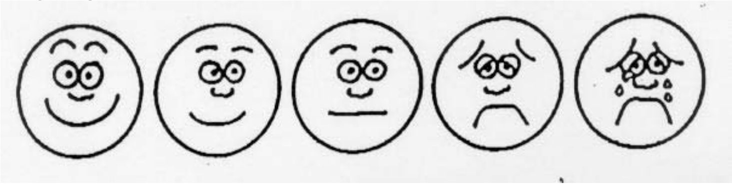

23 A. Je m´absente de l´école à cause de la maladie. **Oui** **Non**

Si «oui» passe à la question 23 B.

23 B. Comment te sens tu lorsque tu t´absentes de l´école à cause de la maladie?
Tu dois encercler le visage qui montre comment tu te sens.

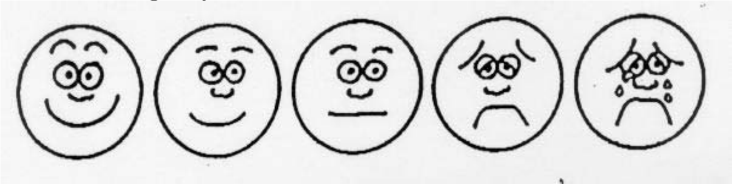

Copyright © 2007 Lakshmi Nandini Moorthy. Tous droits réservés.

24 A. La maladie modifie mes activités extra scolaires. Oui Non

Si tu as répondu «oui» passe à la question 24 B.

24 B. Comment te sens tu lorsque la maladie affecte tes activités extra scolaires? Tu dois encercler le visage qui montre comment tu te sens.

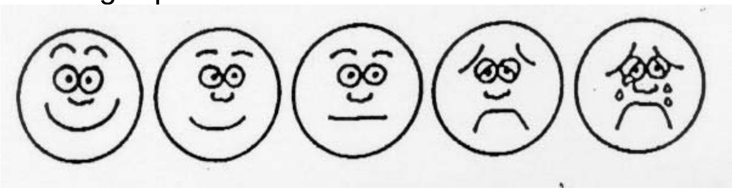

25 A. La maladie modifie le plaisir que tu prends avec tes amis. Oui Non

Si tu as répondu «oui» passe à la question 25 B.

25 B. Comment te sens tu lorsque la maladie affecte les loisirs que tu as avec tes amis? Tu dois encercler le visage qui montre comment tu te sens.

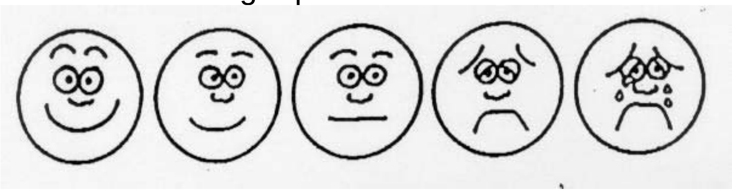

26 A. La maladie modifie ta participation aux activités physiques (comme faire de la gymnastique à l´école, courir, marcher, jouer à ton jeu de plein air préféré)
Oui Non

Si tu as répondu «oui» passe à la question 26B.

26 B. Comment ressens tu le fait que la maladie affecte ta participation aux activités physiques (comme participer à la gymnastique à l´école, courir, marcher, jouer à ton jeu de plein air préféré)? Tu dois encercler le visage qui montre comment tu te sens.

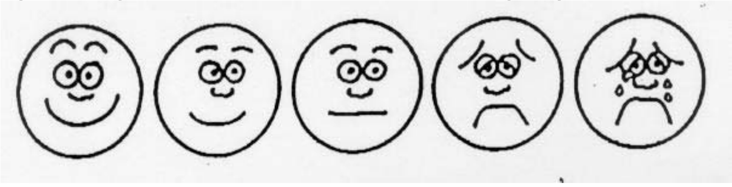

Copyright © 2007 Lakshmi Nandini Moorthy. Tous droits réservés.

MESURE SIMPLE DE L'IMPACT DE LA MALADIE CHEZ LES JEUNES (SMILY-Illness) – Questionnaire parent

Étude numéro __ Date __/__/20

Pour ce questionnaire, nous avons pris les 5 visages de l'échelle d'évaluation de la douleur des VISAGES (FACES) de Wong-Baker, qui ont été modifiés avec la permission du Dr Wong. Ces 5 visages apparaîtront sous chaque question.

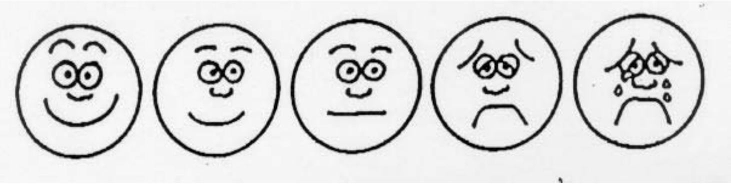

De Wong DL, Hockenberry-Eaton M, Wilson D,Winklestein ML, Schwartz P.: Wong's Essentials of Pediatric Nursing, éd. 6, St. Louis, 2001, Mosby, p. 1301. Copyright Mosby. Reproduit avec autorisation.

The SMILY-Illness est protégé pour les droits d'auteur au nom de L. Nandini Moorthy, MD MS, RWJMS-UMDNJ, New Brunswick, NJ. Le questionnaire ne peut être utilisé que par L. Nandini Moorthy, MD MS et les personnes autorisées dans le cadre du protocole d'étude.

QUESTIONS

Dans ce questionnaire, on vous posera plusieurs questions sur la maladie et le ressenti de votre enfant. Il n'y a aucune mauvaise réponse. N'hésitez pas à nous demander toutes les clarifications nécessaires si il y a des choses que vous ne comprenez pas. Si vous désirez nous faire part de vos commentaires écrits, vous pouvez le faire au verso des pages 5 et 6.

Différents choses affectent la vie de votre enfant et peuvent le/la rendre heureux ou triste. Il y a certaines choses qu'il/elle est capable (ou incapable) de faire, qui sont importantes pour lui/elle et qui contribuent à sa qualité de vie.

1. Comment est la qualité de vie de votre enfant?
Entourez le visage qui illustre le mieux la qualité de vie de votre enfant.

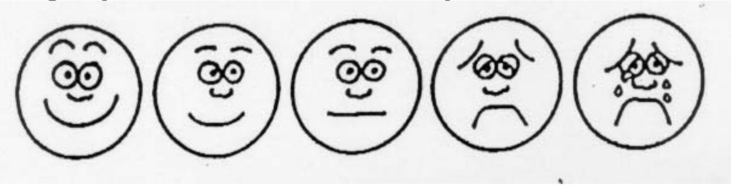

2. Comment est la maladie de votre enfant en ce moment?
Entourez le visage qui illustre le mieux la maladie de votre enfant.

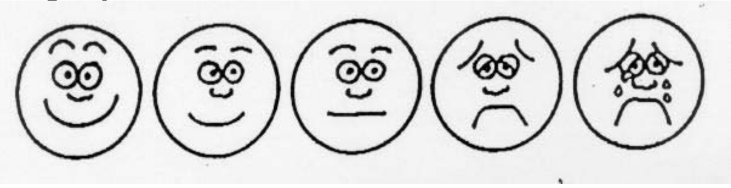

Copyright © 2007 Lakshmi Nandini Moorthy. Tous droits réservés.

Les questions 3 à 19 portent sur le ressenti de la maladie que votre enfant a éprouvé au cours du dernier mois, à cause de la maladie. Entourez le visage qui illustre le mieux votre réponse.

3. Comment votre enfant s'est il senti vis à vis de la maladie?

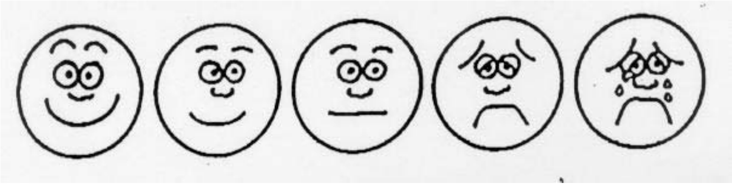

4. Comment votre enfant s'est il senti, avec la maladie, pour aller à l'école ?

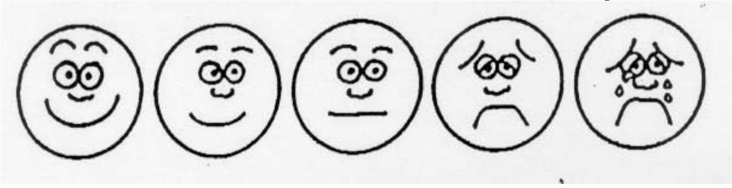

5. Comment votre enfant s'est il senti, avec la maladie, pour s'habiller chaque jour?

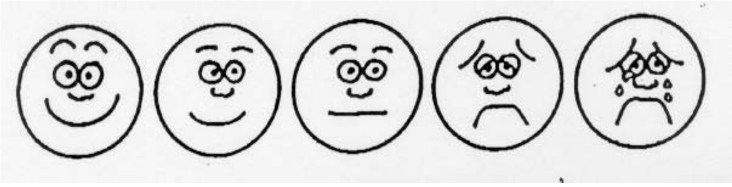

6. Comment votre enfant s'est il senti, avec la maladie ; vis à vis de son apparence (look) ?

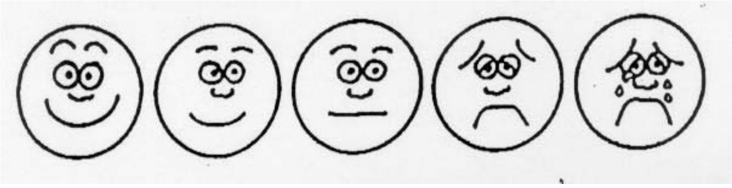

7. Comment votre enfant s'est il senti vis à vis de son moral/estime de soi ?

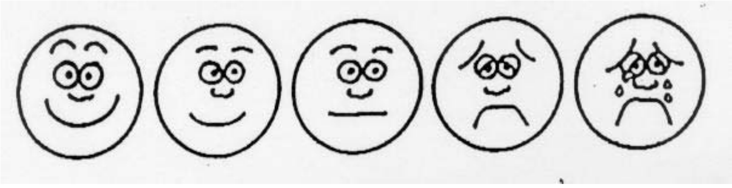

8. Comment votre enfant se sent il lorsqu'il doit parler de la maladie?

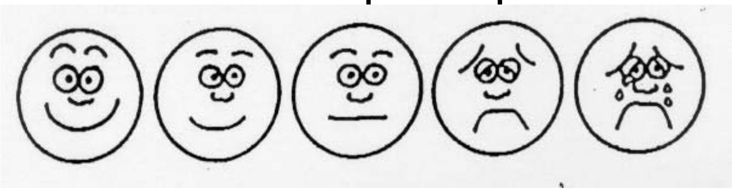

Copyright © 2007 Lakshmi Nandini Moorthy. Tous droits réservés.

9. Comment votre enfant se sent il/elle quand il/elle doit voir le médecin à cause de la maladie?

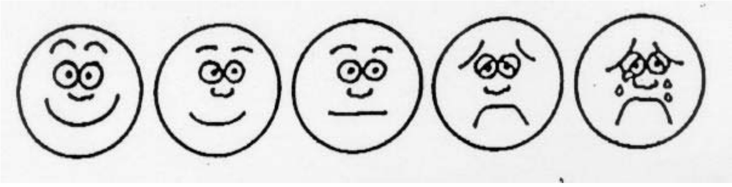

10. Comment votre enfant se sent il/elle quand il/elle doit aller à l'hôpital à cause de la maladie?

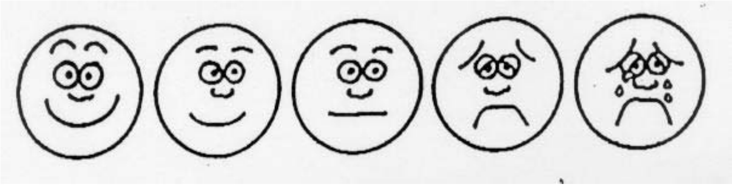

11. Comment votre enfant se sent il/elle vis à vis de son avenir par rapport à la maladie?

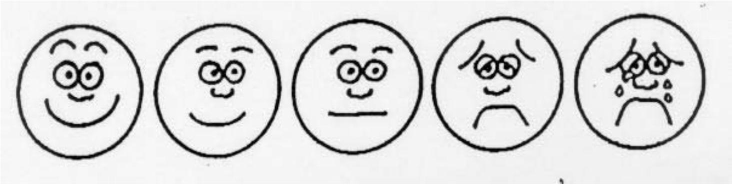

12. Qu'éprouve votre enfant à l'idée de prendre des médicaments pour soigner la maladie?

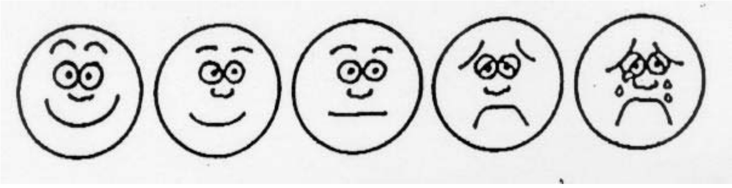

13. Quel degré d'inquiétude votre enfant ressent-il/elle face aux effets secondaires des médicaments qu'il/elle prend pour soigner la maladie?

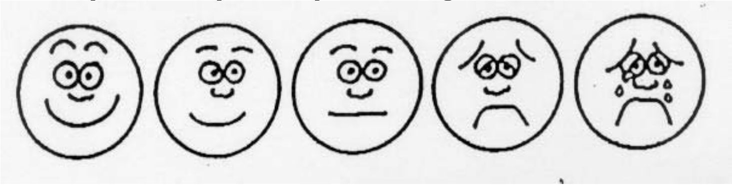

14. Quel degré d'inquiétude votre enfant ressent-il/elle par rapport au risque d'aggravation de sa maladie?

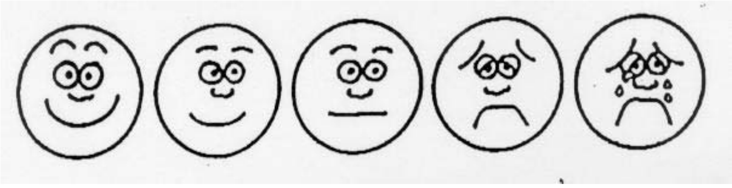

Copyright © 2007 Lakshmi Nandini Moorthy. Tous droits réservés.

15. Votre enfant se sent-il/elle seul(e) en raison de sa maladie?

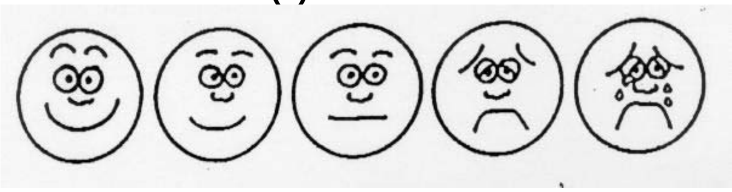

16. Comment votre enfant se sent il dans sa famille à cause de la maladie ?

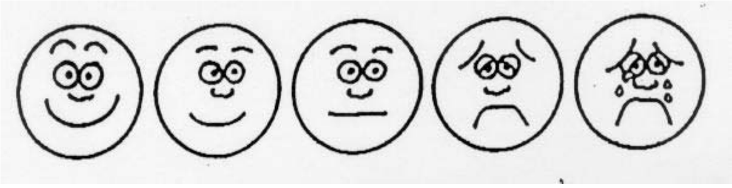

17. Comment votre enfant se sent il/elle par rapport à la douleur liée à la maladie ?

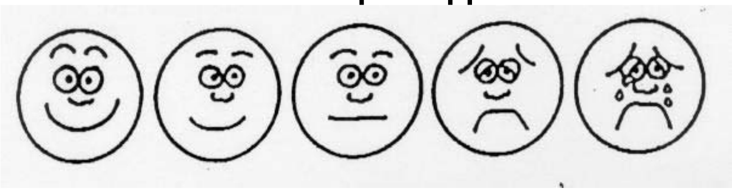

18. Comment votre enfant se sent il/elle à l'idée de ne pas pouvoir aller au soleil à cause de la maladie?

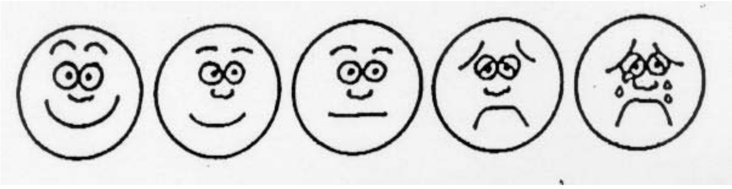

19. Quel degré de fatigue la maladie fait-elle éprouver à votre enfant?

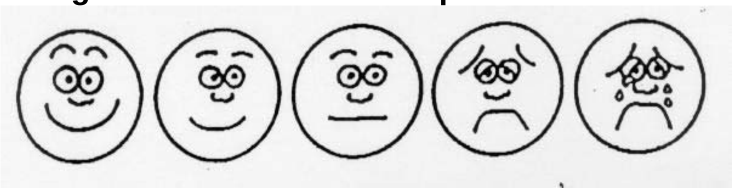

Copyright © 2007 Lakshmi Nandini Moorthy. Tous droits réservés.

Pour les questions 20 A à 26 A, veuillez répondre « oui » ou « non ». Ensuite, veuillez suivre les indications.

20 A. Les amis de votre enfant ont changé à cause de sa maladie.
Veuillez répondre « oui » ou « non ». Oui Non

Si « oui », passez à la question 20 B.

20 B. Quels sentiments la maladie fait-elle éprouver à votre enfant face à ses amis? Veuillez encercler le visage qui illustre le mieux les sentiments de votre enfant.

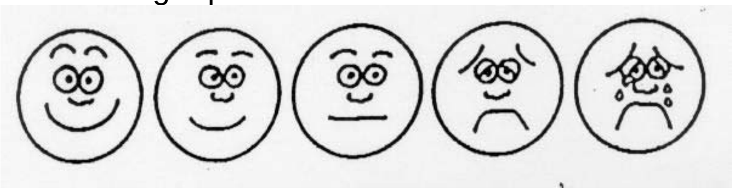

21 A. Les amis de votre enfant comprennent qu'il/elle a une maladie. Oui Non

Si vous avez répondu « non », passez à la question 21 B.

21 B. Quels sentiments votre enfant éprouve t-il/elle face au fait que ses amis ne comprennent pas qu'il/elle a une maladie? Veuillez encercler le visage qui illustre le mieux les sentiments de votre enfant.

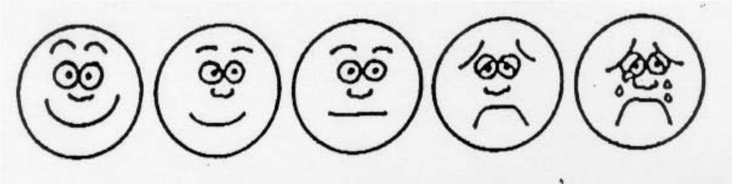

22 A. La maladie affecte les études de votre enfant. Oui Non

Si « oui », passez à la question 22 B.

22 B. Quels sentiments la maladie fait-elle éprouver à votre enfant face à ses études? Veuillez encercler le visage qui illustre le mieux les sentiments de votre enfant.

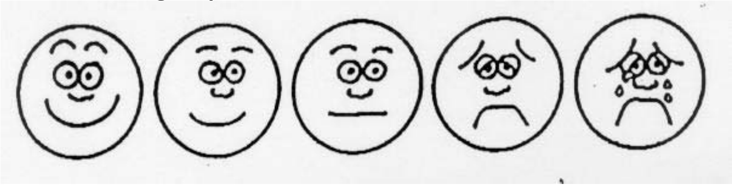

23 A. Votre enfant ne va pas à l'école à cause de la maladie. Oui Non

Si « oui », passez à la question 23 B.

23 B. Quels sentiments votre enfant éprouve t-il/elle à l'idée de ne pas aller à l'école à cause de la maladie?
Veuillez encercler le visage qui illustre le mieux les sentiments de votre enfant.

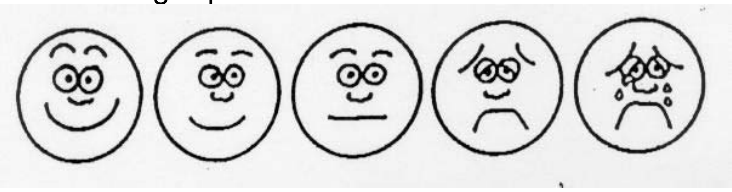

Copyright © 2007 Lakshmi Nandini Moorthy. Tous droits réservés.

24 A. La maladie affecte les activités extrascolaires de votre enfant. Oui Non

Si vous avez répondu « oui », passez à la question 24 B.

24 B. Quels sentiments votre enfant éprouve t-il/elle lorsque la maladie affecte ses activités parascolaires? Veuillez encercler le visage qui illustre le mieux les sentiments de votre enfant.

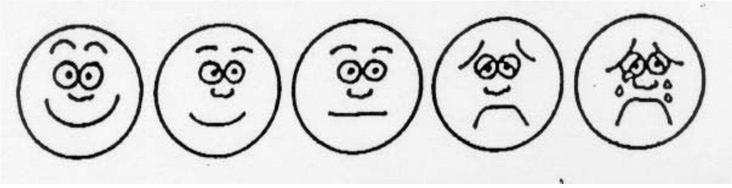

25 A. La maladie affecte le plaisir qu'a votre enfant d'être avec ses amis. Oui Non

Si vous avez répondu « oui », passez à la question 25 B.

25 B. Quels sentiments votre enfant éprouve t-il/elle lorsque la maladie affecte le plaisir qu'il/elle a d'être avec ses amis? Veuillez encercler le visage qui illustre le mieux les sentiments de votre enfant.

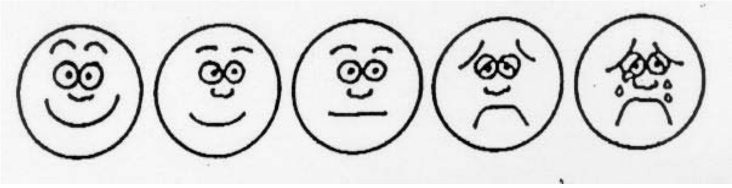

26 A. La maladie affecte la participation de votre enfant à des activités physiques (comme participer à la gymnastique à l´école, courir, marcher, jouer à son jeu de plein air préféré)**.**

Oui Non

Si vous avez répondu « oui », passez à la question 26 B.

26 B. Quels sentiments votre enfant éprouve t-il/elle le fait que la maladie affecte sa participation à des activités physiques (comme participer à la gymnastique à l´école, courir, marcher, jouer à son jeu de plein air préféré) ? Veuillez encercler le visage qui illustre le mieux les sentiments de votre enfant.

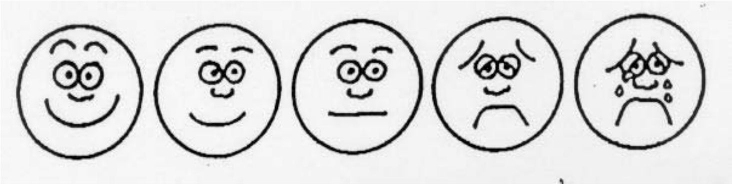

Copyright © 2007 Lakshmi Nandini Moorthy. Tous droits réservés.

EINFACHE MESSUNG DER AUSWIRKUNGEN VON KRANKHEIT BEI KINDERN UND JUGENDLICHEN (SMILY-Illness) – Elternfragebogen (German for Austria)

German austria

Nr. der Studie __ Datum __/__/20_

Für diesen Fragebogen haben wir die 5 Gesichter aus der Wong-Baker GESICHTER-Skala zur Einstufung von Schmerzempfinden ausgewählt, und mit Erlaubnis von D.L. Wong modifiziert. Diese 5 Gesichter werden unter jeder Frage erscheinen.

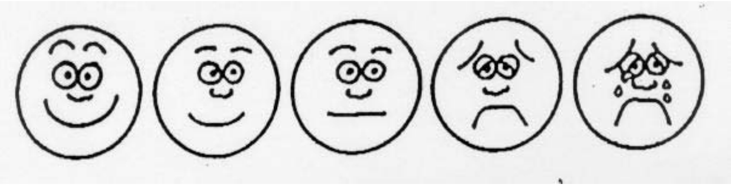

Aus der Publikation von Wong DL, Hockenberry-Eaton M, Wilson D,Winklestein ML, Schwartz P.: *Wong's Essentials of Pediatric Nursing*, 6. Aufl., St. Louis, 2001, Verlag Mosby, S. 1301. Urheberrechtlich geschützt, Verlag Mosby. Nachdruck mit Erlaubnis des Berechtigten.

Inhaberin des Urheberrechts an SMILY-Illness ist L. Nandini Moorthy, MD MS, RWJMS-UMDNJ, New Brunswick, NJ. Derzeit darf der Fragebogen ausschließlich von L. Nandini Moorthy, MD MS und durch von ihr ermächtigtes Studienpersonal verwendet werden.

FRAGEN

In diesem Fragebogen stellen wir Ihnen viele Fragen über die Krankheit Ihres Kindes und über sein Befinden und seine Gefühle. Es gibt keine falschen Antworten. Bitte fragen Sie uns, wenn Sie etwas nicht verstehen. Wenn Sie etwas anmerken möchten, schreiben Sie Ihre Anmerkungen bitte im Anschluss an die Seiten 5 und 6 auf.

Verschiedene Dinge beeinflussen das Leben Ihres Kindes und stimmen es glücklich oder traurig. Es gibt wichtige Dinge im Leben Ihres Kindes, die es tun kann (oder nicht tun kann). Alle diese verschiedenen Dinge im Leben Ihres Kindes machen insgesamt seine Lebensqualität aus.

1. Wie ist die Lebensqualität Ihres Kindes? Bitte markieren Sie das Gesicht, das seine Lebensqualität am besten wiedergibt.

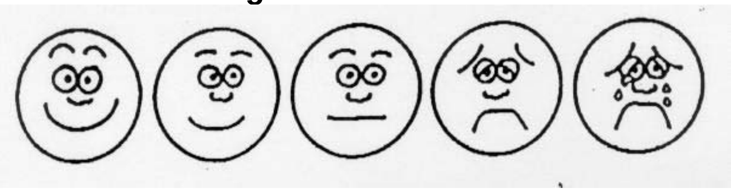

2. Wie ist die Krankheit Ihres Kindes derzeit? Bitte markieren Sie das Gesicht, das am besten wiedergibt, wie die Krankheit Ihres Kindes ist.

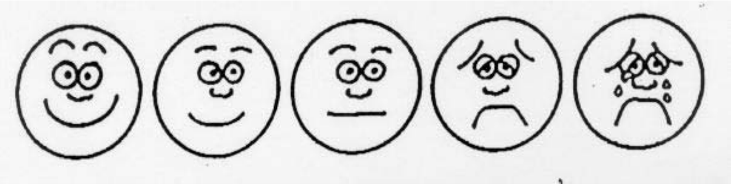

Copyright © 2007 Lakshmi Nandini Moorthy. Alle Rechte vorbehalten.

Fragen 3-19 betreffen das durch die Krankheit verursachte Empfinden Ihres Kindes während der letzten Monate. Bitte markieren Sie das Gesicht, das Ihre Antwort am besten wiedergibt.

3. Welche Gefühle löst die Krankheit bei Ihrem Kind aus?

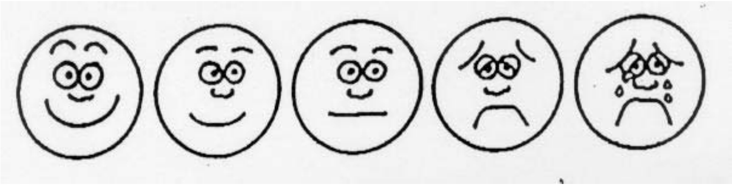

4. Wie ist es für Ihr Kind, mit der Krankheit die Schule zu besuchen?

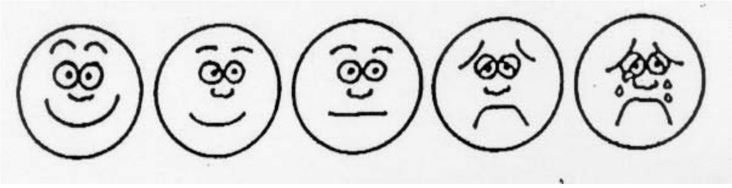

5. Wie ist es für Ihr Kind, sich mit der Krankheit jeden Tag anzuziehen?

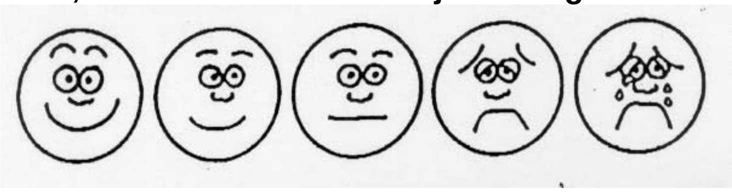

6. Welche Gefühle löst die Krankheit bei Ihrem Kind im Hinblick auf sein Aussehen aus?

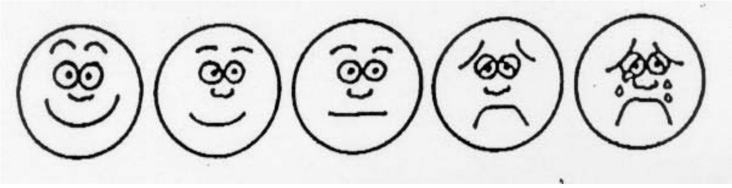

7. Wie beeinflusst die Tatsache der Krankheit das Selbstempfinden Ihres Kindes?

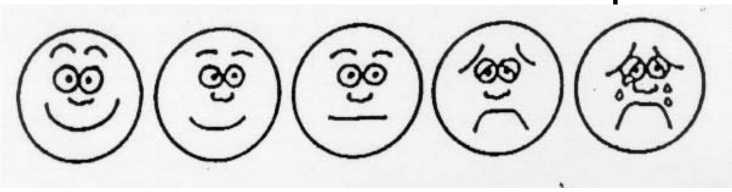

8. Wie fühlt sich Ihr Kind, wenn es über seine Krankheit reden muss?

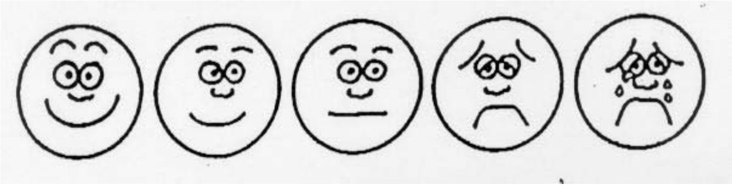

9. Wie empfindet es Ihr Kind, wegen seiner Krankheit zum Arzt gehen zu müssen?

Copyright © 2007 Lakshmi Nandini Moorthy. Alle Rechte vorbehalten.

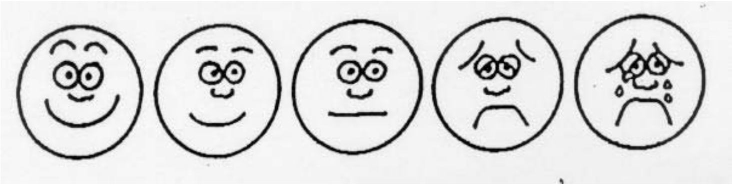

10. Wie empfindet es Ihr Kind, wegen seiner Krankheit das Krankenhaus aufsuchen zu müssen?

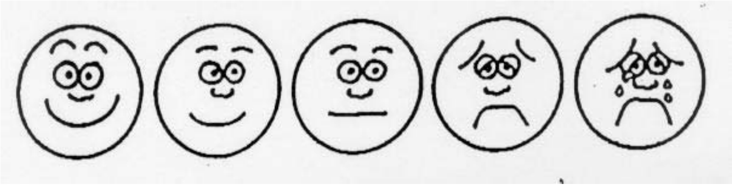

11. Welche Gefühle löst die Krankheit bei Ihrem Kind im Hinblick auf seine Zukunft aus?

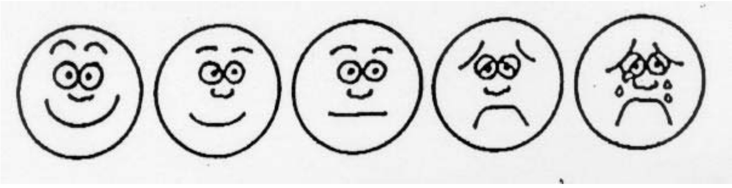

12. Wie fühlt sich Ihr Kind damit, wegen seiner Krankheit Medikamente nehmen zu müssen?

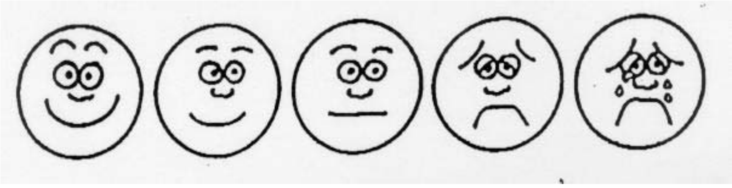

13. Wie besorgt ist Ihr Kind über die Nebenwirkungen der Medikamente, die es gegen seine Krankheit einnehmen muss?

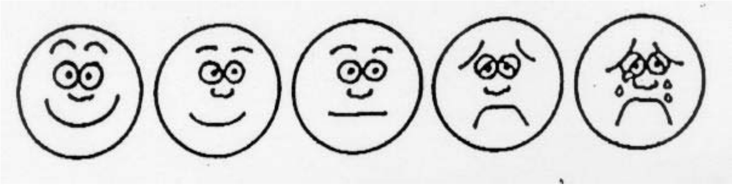

14. Wie besorgt ist Ihr Kind darüber, dass die sich die Krankheit verschlechtern könnte?

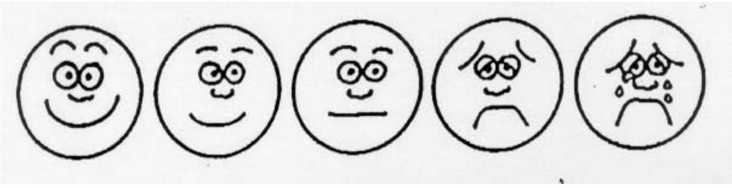

Copyright © 2007 Lakshmi Nandini Moorthy. Alle Rechte vorbehalten.

15. Wie einsam fühlt sich Ihr Kind wegen seiner Krankheit?

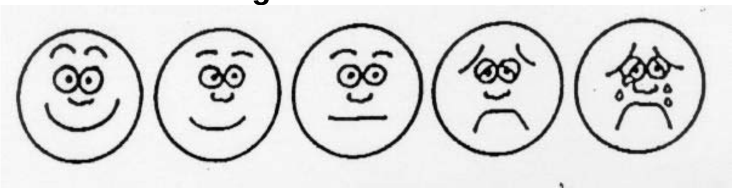

16. Welches Gefühl wird Ihrem Kind durch seine Familie vermittelt?

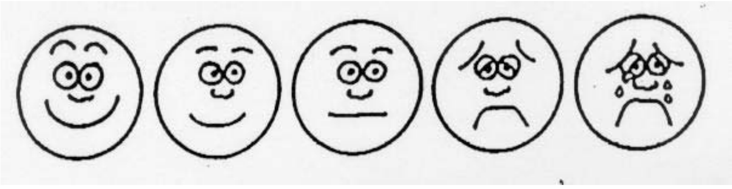

17. Welches Gefühl lösen die Schmerzen der Krankheit bei Ihrem Kind aus?

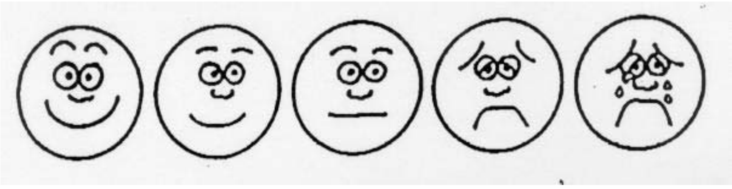

18. Was empfindet Ihr Kind dabei, wegen seiner Krankheit nicht in die Sonne gehen zu können?

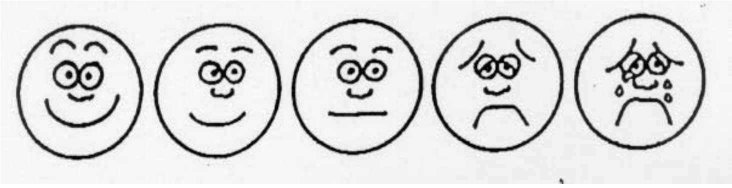

19. Wie müde macht die Krankheit Ihr Kind?

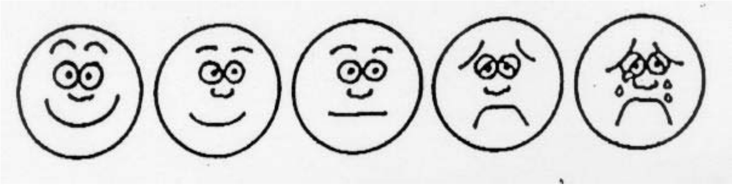

Copyright © 2007 Lakshmi Nandini Moorthy. Alle Rechte vorbehalten.

Beantworten Sie die Fragen 20 A- 26 A mit „Ja” oder „Nein.” Bitte folgen Sie dann den Anweisungen.

20 A. Die Freunde Ihres Kindes haben sich wegen seiner Krankheit verändert. Bitte markieren Sie „Ja” oder „Nein.”

Ja **Nein**

Bei „Ja”, gehen Sie zu Frage 20 B.

20 B. Welches Gefühl gibt die Krankheit Ihrem Kind bezüglich seiner Freunde? Bitte markieren Sie das Gesicht, das zeigt, wie sich Ihr Kind fühlt.

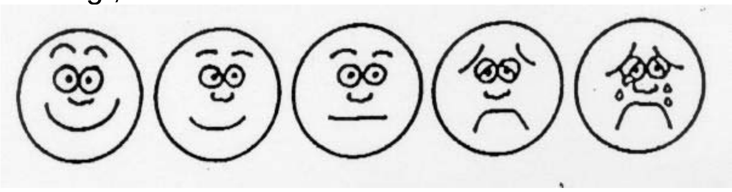

21 A. Die Freunde Ihres Kindes haben Verständnis dafür, dass es krank ist.

Ja **Nein**

Bei „Ja”, gehen Sie zu Frage 21 B.

21 B. Was empfindet Ihr Kind dabei, dass seine Freunde kein Verständnis dafür haben, dass es krank ist? Bitte markieren Sie das Gesicht, das zeigt, wie sich Ihr Kind fühlt.

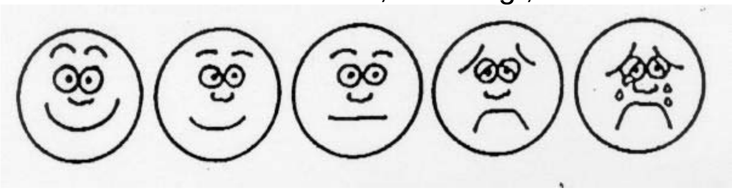

22 A. Die Krankheit beeinträchtigt die schulischen Leistungen Ihres Kindes.

Ja **Nein**

Bei „Ja”, gehen Sie zu Frage *22 B*

22 B. Wie empfindet Ihr Kind seine Krankheit bezüglich seiner schulischen Leistungen? Bitte markieren Sie das Gesicht, das zeigt, wie sich Ihr Kind fühlt.

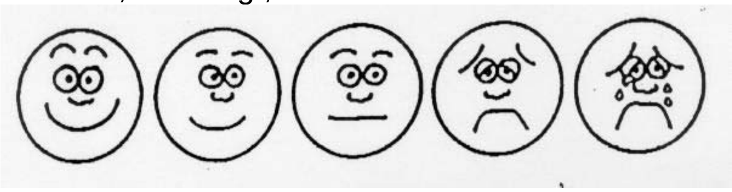

23 A. Ihr Kind versäumt wegen der Krankheit die Teilnahme an der Schule.

Ja **Nein**

Bei „Ja”, gehen Sie zu Frage 23 B.

23 B. Was empfindet Ihr Kind dabei, wegen seiner Krankheit die Teilnahme an der Schule zu versäumen? Bitte markieren Sie das Gesicht, das zeigt, wie sich Ihr Kind fühlt.

Copyright © 2007 Lakshmi Nandini Moorthy. Alle Rechte vorbehalten.

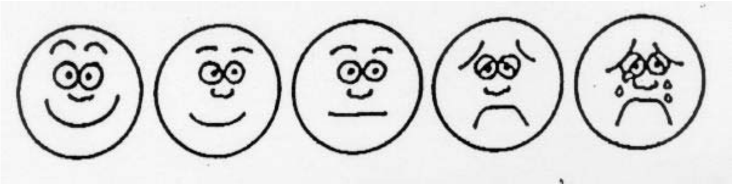

24 A. Die Krankheit beeinträchtigt die außerschulischen Aktivitäten Ihres Kindes.

Ja **Nein**

Bei „Ja", gehen Sie zu Frage 24 B.

24 B. Was empfindet Ihr Kind dabei, dass die Krankheit seine außerschulischen Aktivitäten beeinträchtigt? Bitte markieren Sie das Gesicht, das zeigt, wie sich Ihr Kind fühlt.

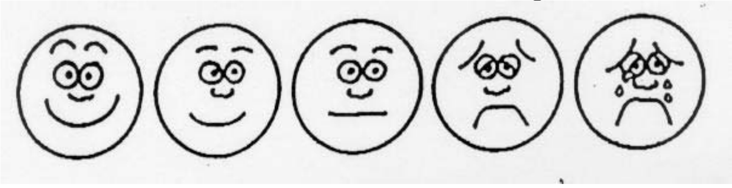

25 A. Die Krankheit beeinträchtigt Ihr Kind darin, Spaß mit seinen Freunden zu haben.

Ja **Nein**

Bei „Ja", gehen Sie zu Frage 25 B.

25 B. Was empfindet Ihr Kind dabei, dass die Krankheit es darin beeinträchtigt, Spaß mit seinen Freunden zu haben? Bitte markieren Sie das Gesicht, das zeigt, wie sich Ihr Kind fühlt.

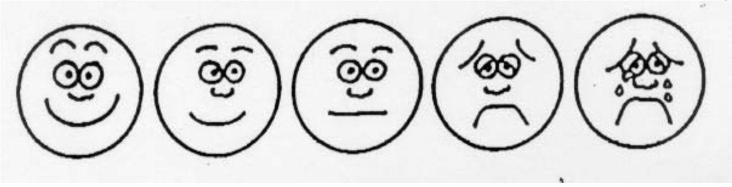

26 A. Die Krankheit beeinträchtigt Ihr Kind in seinen körperlichen Betätigungen (wie zum Beispiel beim Sport in der Schule, beim Laufen, Gehen oder beim Spielen eines Lieblingsspiels draußen).

Ja **Nein**

Bei „Ja", gehen Sie zu Frage 26B.

26 B. Was empfindet Ihr Kind dabei, dass die Krankheit es in seinen körperlichen Betätigungen (wie zum Beispiel beim Sport in der Schule, beim Laufen, Gehen oder beim Spielen eines Lieblingsspiels draußen) beeinträchtigt? Bitte markieren Sie das Gesicht, das zeigt, wie sich Ihr Kind fühlt.

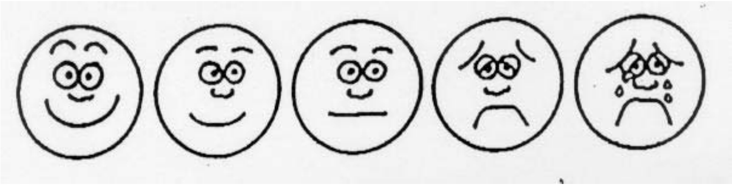

Copyright © 2007 Lakshmi Nandini Moorthy. Alle Rechte vorbehalten.

EINFACHE MESSUNG DER AUSWIRKUNGEN VON KRANKHEIT BEI KINDERN UND JUGENDLICHEN (SMILY-Illness) – Kinderfragebogen

Nr. der Studie __ Datum __/__/20_

Für diesen Fragebogen haben wir die 5 Gesichter aus der Wong-Baker GESICHTER-Skala zur Einstufung von Schmerzempfinden ausgewählt, und mit Erlaubnis von D.L. Wong modifiziert. Diese 5 Gesichter stehen unter jeder Frage.

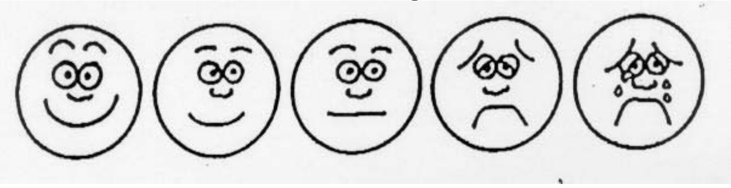

Aus der Publikation von Wong DL, Hockenberry-Eaton M, Wilson D,Winklestein ML, Schwartz P.: *Wong's Essentials of Pediatric Nursing*, 6. Aufl., St. Louis, 2001, Verlag Mosby, S. 1301. Urheberrechtlich geschützt, Verlag Mosby. Nachdruck mit Erlaubnis des Berechtigten.

Inhaberin des Urheberrechts an SMILY-illness ist L. Nandini Moorthy, MD MS, RWJMS-UMDNJ, New Brunswick, NJ. Derzeit darf der Fragebogen ausschließlich von L. Nandini Moorthy, MD MS und durch von ihr ermächtigtes Studienpersonal verwendet werden.

FRAGEN

In diesem Fragebogen stellen wir Dir viele Fragen über Deine Krankeit und über Dein Befinden und Deine Gefühle. Es gibt keine falschen Antworten. Bitte frage uns, wenn Du etwas nicht verstehst. Wenn Du etwas anmerken möchtest, schreibe Deine Anmerkungen bitte im Anschluss an die Seiten 5 und 6 auf.

Verschiedene Dinge beeinflussen Dein Leben und machen Dich glücklich oder traurig. Es gibt Dinge in Deinem Leben, die für Dich wichtig sind und die Du tun kannst (oder nicht tun kannst). Alle diese verschiedenen Dinge in Deinem Leben machen insgesamt Deine Lebensqualität aus.

1. Wie ist Deine Lebensqualität? Bitte markiere das Gesicht, das Deine Lebensqualität am besten beschreibt.

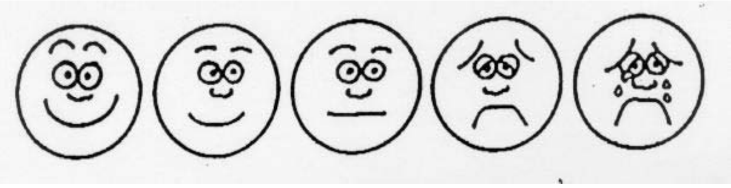

2. Wie ist Deine Krankeit derzeit? Bitte markiere das Gesicht, das Deine Krankheit am besten beschreibt.

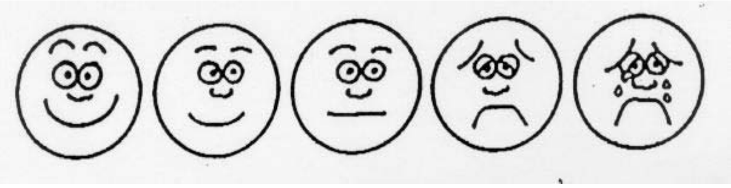

Copyright © 2007 Lakshmi Nandini Moorthy. Alle Rechte vorbehalten.

In den Fragen 3 bis 19 wirst Du darüber befragt, wie Du Dich in den vergangenen Monaten durch Deine Krankheit gefühlt hast.
Bitte markiere das Gesicht, das am besten beschreibt, wie Du Dich fühlst.

3. Wie ist es für Dich, krank zu sein?

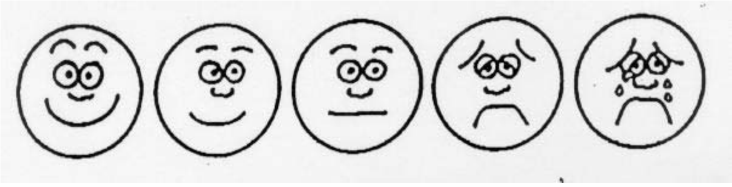

4. Wie ist es für Dich, mit der Krankheit zur Schule zu gehen?

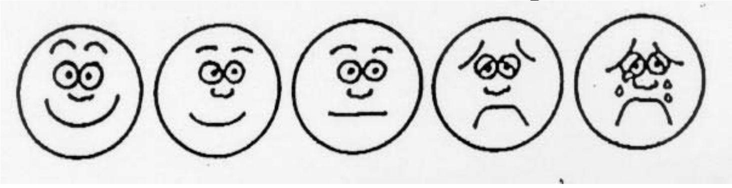

5. Wie ist es für Dich, sich mit der Krankheit jeden Tag anzuziehen?

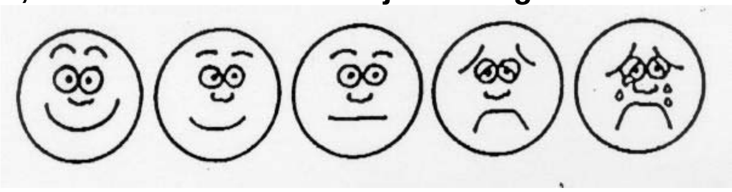

6. Wie empfindest Du Dein durch die Krankheit verursachtes Aussehen?

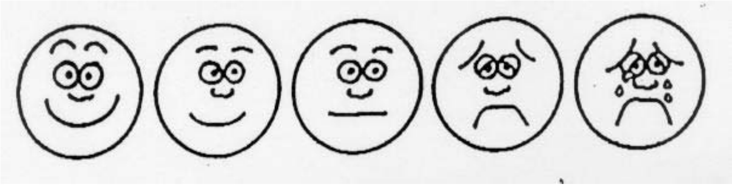

7. Wie beeinflusst die Krankheit Dein Selbstempfinden?

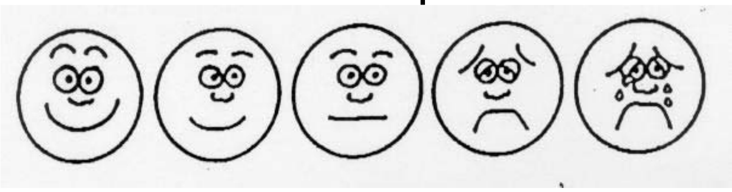

8. Wie fühlst Du Dich, wenn Du über Deine Krankheit reden musst?

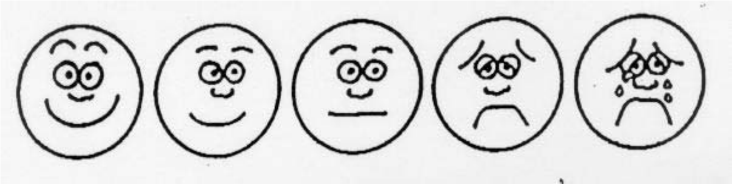

9. Wie empfindest Du es, wegen Deiner Krankheit zum Arzt gehen zu müssen?

Copyright © 2007 Lakshmi Nandini Moorthy. Alle Rechte vorbehalten.

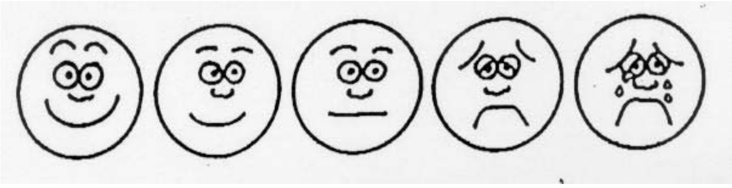

10. Wie empfindest Du es, wegen Deiner Krankheit ins Krankenhaus gehen zu müssen?

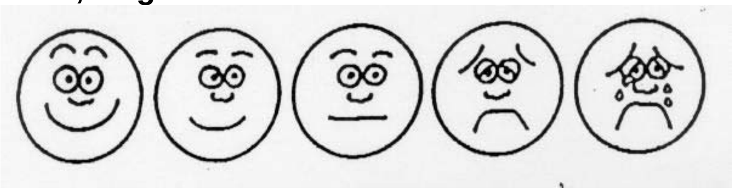

13. Wie fühlst Du Dich wegen Deiner Krankheit, wenn Du an Deine Zukunft denkst?

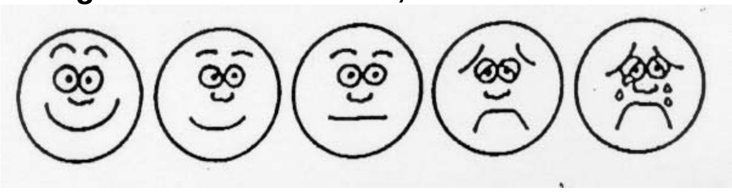

12. Wie empfindest Du es, wegen Deiner Krankheit Medikamente nehmen zu müssen?

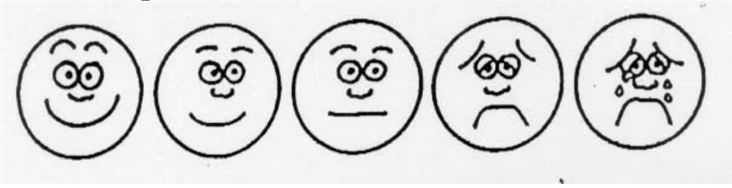

13. Wie besorgt bist Du über die Nebenwirkungen der Medikamente, die Du gegen Deine Krankheit nimmst?

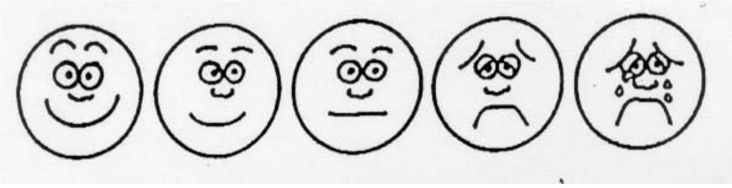

14. Wie besorgt bist Du darüber, dass sich die Krankheit verschlechtern könnte?

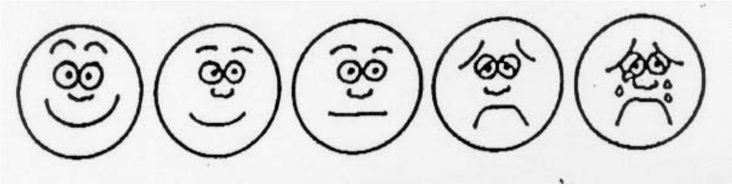

15. Wie einsam fühlst Du Dich wegen Deiner Krankheit?

Copyright © 2007 Lakshmi Nandini Moorthy. Alle Rechte vorbehalten.

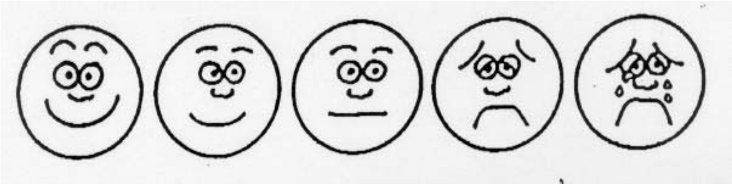

16. Welches Gefühl gibt Dir Deine Familie?

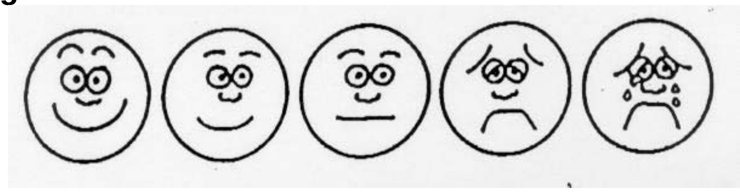

17. Welches Gefühl lösen die Schmerzen der Krankheit bei Dir aus?

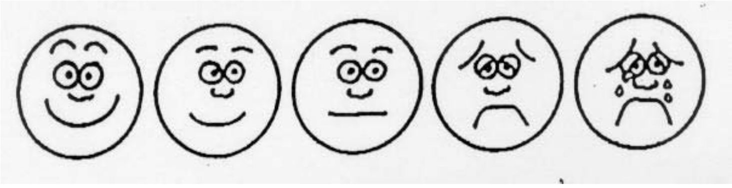

18. Wie findest Du es, dass Du wegen der Krankheit nicht in die Sonne gehen kannst?

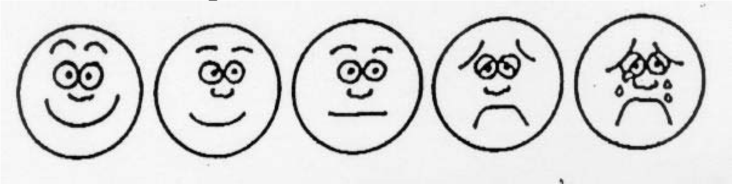

19. Wie müde macht Dich die Krankheit?

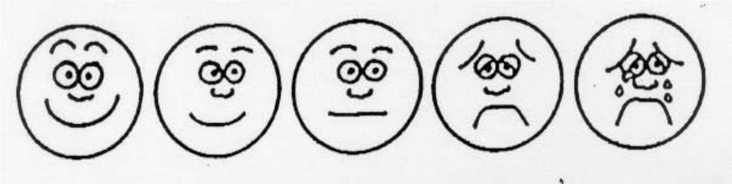

Copyright © 2007 Lakshmi Nandini Moorthy. Alle Rechte vorbehalten.

Beantworte die Fragen 20 A- 26 A mit „Ja" oder „Nein." Bitte folge dann den Anweisungen.

20 A. Meine Freunde haben sich wegen meiner Krankheit verändert. Bitte markiere „Ja" oder „Nein." Ja Nein

Wenn Du „Ja" gewählt hast, gehe zu Frage 20 B.

20 B. Welches Gefühl gibt Dir Deine Krankheit bezogen auf Deine Freunde? Bitte markiere das Gesicht, das zeigt, wie Du Dich fühlst.

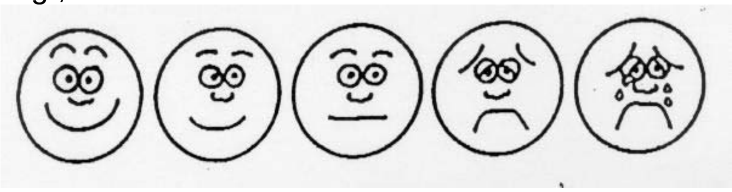

21 A. Meine Freunde haben Verständnis dafür, dass ich krank bin.

Ja Nein

Wenn Du „Ja" gewählt hast, gehe zu Frage 21 B.

21 B. Wie findest Du es, wenn Deine Freunde kein Verständnis haben, dass Du krank bist? Bitte markiere das Gesicht, das zeigt, wie Du Dich fühlst.

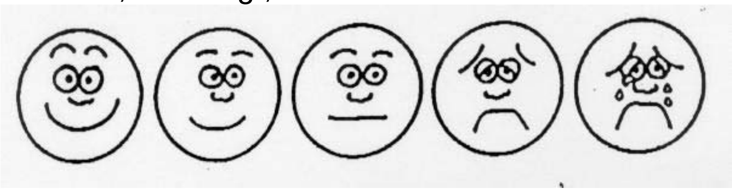

22 A. Die Krankheit beeinträchtigt meine schulischen Leistungen. Ja Nein

Wenn Du „Ja" gewählt hast, gehe zu *Frage 22 B*

22 B. Welches Gefühl gibt Dir Deine Krankheit bezogen auf Deine schulischen Leistungen? Bitte markiere das Gesicht, das zeigt, wie Du Dich fühlst.

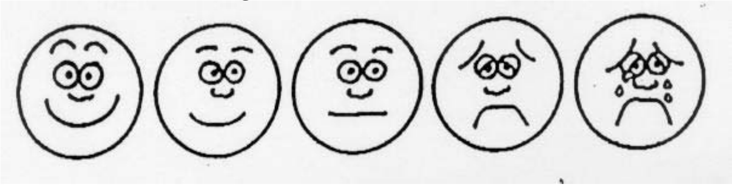

23 A. Ich versäume wegen meiner Krankheit viele Schulstunden.

Ja Nein

Wenn Du „Ja" gewählt hast, gehe zu Frage 23 B.

23 B. Wie findest Du es, dass Du wegen Deiner Krankheit viele Schulstunden versäumst? Bitte markiere das Gesicht, das zeigt, wie Du Dich fühlst.

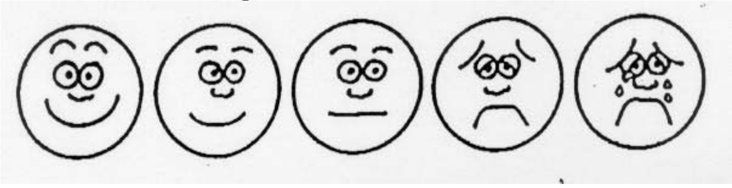

Copyright © 2007 Lakshmi Nandini Moorthy. Alle Rechte vorbehalten.

24 A. Die Krankheit beeinträchtigt meine außerschulischen Aktivitäten.

Ja **Nein**

Wenn Du „Ja” gewählt hast, gehe zu Frage 24 B.

24 B. Wie findest Du es, dass die Krankheit Deine außerschulischen Aktivitäten beeinträchtigt? Bitte markiere das Gesicht, das zeigt, wie Du Dich fühlst.

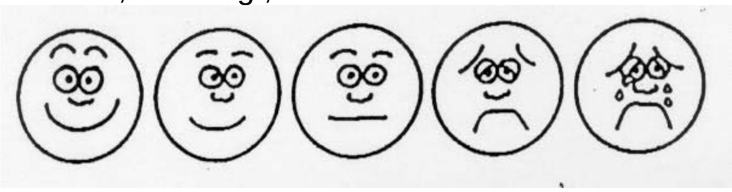

25 A. Die Krankheit beeinträchtigt mich dabei, Spaß mit meinen Freunden zu haben.

Ja **Nein**

Wenn Du „Ja” gewählt hast, gehe zu Frage 25 B.

25 B. Wie findest Du es, dass Dich die Krankheit dabei beeinträchtigt, Spaß mit Deinen Freunden zu haben? Bitte markiere das Gesicht, das zeigt, wie Du Dich fühlst.

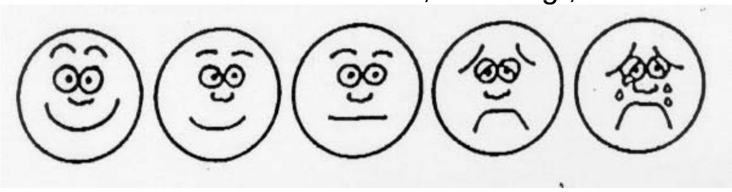

26 A. Die Krankheit beeinträchtigt mich bei körperlichen Betätigungen (wie zum Beispiel beim Sport in der Schule, beim Laufen, Gehen oder beim Spielen eines meiner Lieblingsspiele draußen).

Ja **Nein**

Wenn Du „Ja” gewählt hast, gehe zu Frage 26B.

26 B. Wie findest Du es, dass Dich die Krankheit bei Deinen körperlichen Betätigungen (wie zum Beispiel beim Sport in der Schule, beim Laufen, Gehen oder beim Spielen eines Deiner Lieblingsspiele draußen) beeinträchtigt? Bitte markiere das Gesicht, das zeigt, wie Du Dich fühlst.

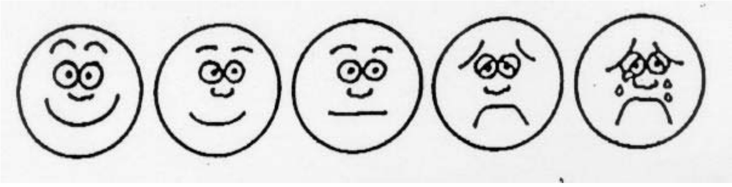

Copyright © 2007 Lakshmi Nandini Moorthy. Alle Rechte vorbehalten.

EINFACHE MESSUNG DER AUSWIRKUNGEN DER KRANKHEIT BEI KINDERN UND JUGENDLICHEN (SMILY-Krankheit) – Kinderfragebogen

German Germany **Nr. der Studie __ Datum __/__/20**

Für diesen Fragebogen haben wir die 5 Gesichter aus der Wong-Baker GESICHTER-Skala zur Einstufung des Schmerzempfindens ausgewählt und mit Erlaubnis von D.L. Wong modifiziert. Diese 5 Gesichter werden unter jeder Frage erscheinen.

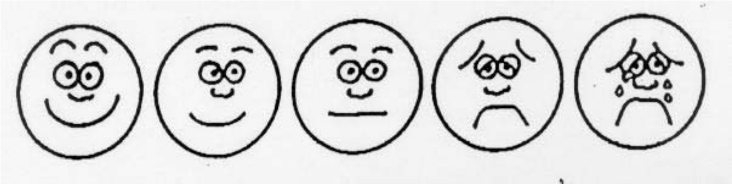

Aus der Publikation von Wong DL, Hockenberry-Eaton M, Wilson D,Winklestein ML, Schwartz P.: *Wong's Essentials of Pediatric Nursing*, 6. Aufl., St. Louis, 2001, Verlag Mosby, S. 1301. Urheberrechtlich geschützt, Verlag Mosby. Nachdruck mit Erlaubnis des Berechtigten.

Inhaberin des Urheberrechts an SMILY ist L. Nandini Moorthy, MD MS, RWJMS-UMDNJ, New Brunswick, NJ. Derzeit darf der Fragebogen ausschließlich von L. Nandini Moorthy, MD MS und durch von ihr ermächtigtes Studienpersonal verwendet werden.

FRAGEN

In diesem Fragebogen stellen wir Dir viele Fragen über Deine Krankheit, über Dein Befinden und Deine Gefühle. Es gibt keine falschen Antworten. Bitte frage uns, wenn Du etwas nicht verstehst. Wenn Du etwas anmerken möchtest, schreibe Deine Anmerkungen bitte im Anschluss an die Seiten 5 und 6 auf.

Verschiedene Dinge beeinflussen Dein Leben und stimmen Dich glücklich oder traurig. Es gibt Dinge in Deinem Leben, die für Dich wichtig sind und die Du machen kannst (oder nicht machen kannst). Alle diese verschiedenen Dinge in Deinem Leben machen insgesamt Deine Lebensqualität aus.

1. Wie ist Deine Lebensqualität? Bitte markiere das Gesicht, das am besten Deine Lebensqualität wiedergibt.

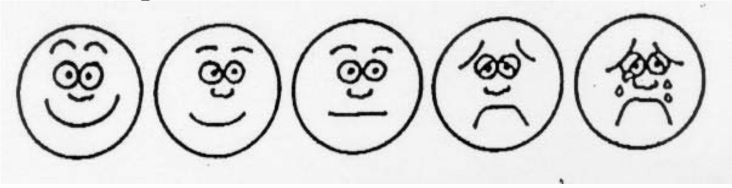

2. Wie ist Deine Krankheit derzeit? Bitte markiere das Gesicht, das am besten Deine Krankheit wiedergibt.

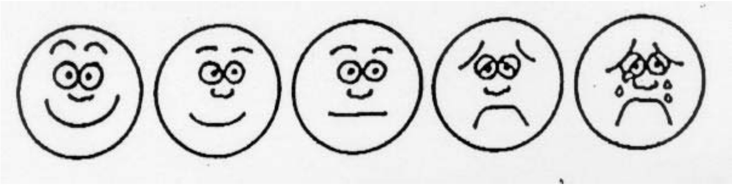

Copyright © 2007 Lakshmi Nandini Moorthy. Alle Rechte vorbehalten.

In den Fragen 3 bis 19 wirst Du darüber befragt, wie Du Dich im vergangenen Monat durch Deine Erkrankung gefühlt hast.
Bitte markiere das Gesicht, das am besten wiedergibt, wie Du Dich fühlst.

3. Wie ist es für Dich eine Krankheit zu haben?

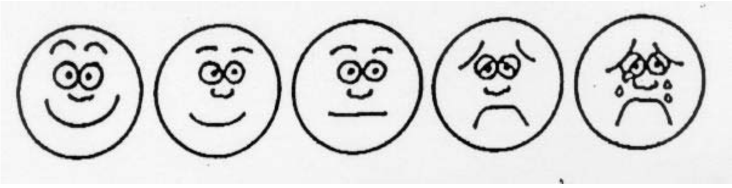

4. Wie ist es für Dich, mit einer Krankheit die Schule zu besuchen?

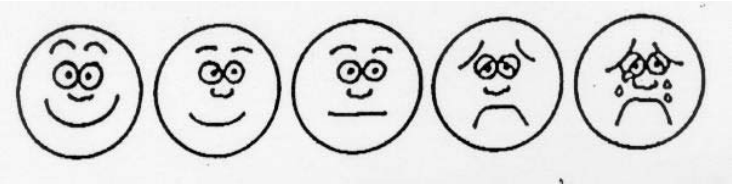

5. Wie ist es für Dich durch Deine Krankheit Dich jeden Tag anzuziehen?

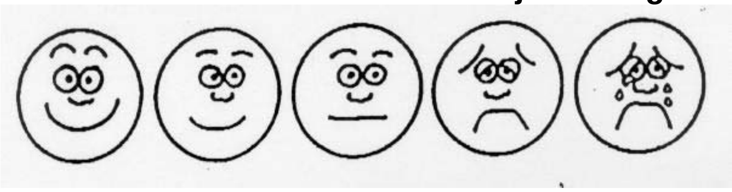

6. Wie empfindest Du Dein durch die Krankheit verursachtes Aussehen?

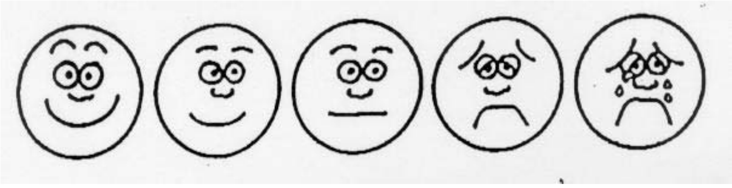

7. Wie beeinflusst die Krankheit Dein Selbstempfinden?

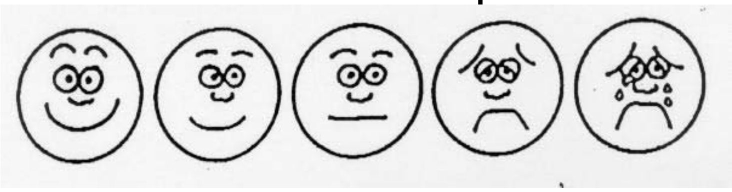

8. Wie fühlst Du Dich, wenn Du über Deine Krankheit reden musst?

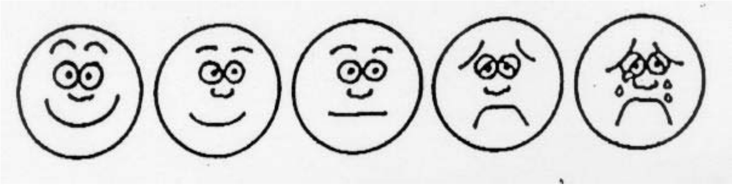

Copyright © 2007 Lakshmi Nandini Moorthy. Alle Rechte vorbehalten.

9. Wie empfindest Du es, wegen Deiner Krankheit zum Arzt zu gehen?

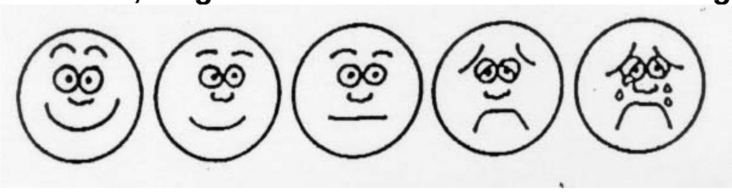

10. Wie empfindest Du es, wegen Deiner Krankheit ins Krankenhaus zu gehen?

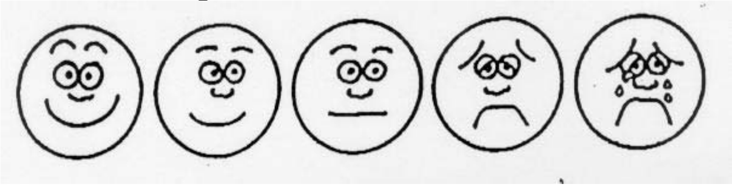

11. Was lässt Dich die Tatsache Deiner Krankheit mit Blick auf Deine Zukunft empfinden?

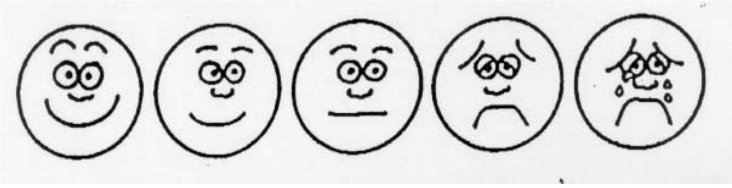

12. Wie empfindest Du es, wegen Deiner Krankheit Medikamente zu nehmen?

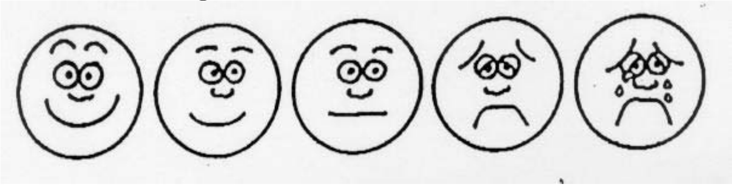

13. Wie besorgt bist Du über Nebenwirkungen der Medikamente, die Du gegen die Krankheit nimmst?

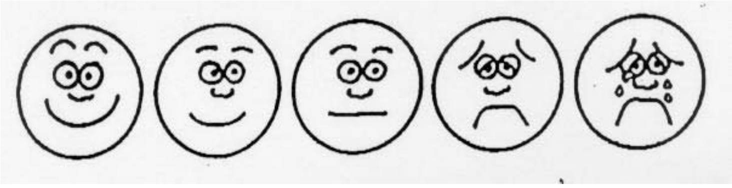

Copyright © 2007 Lakshmi Nandini Moorthy. Alle Rechte vorbehalten.

14. Wie besorgt bist Du darüber, dass die Krankheit sich verschlechtern könnte?

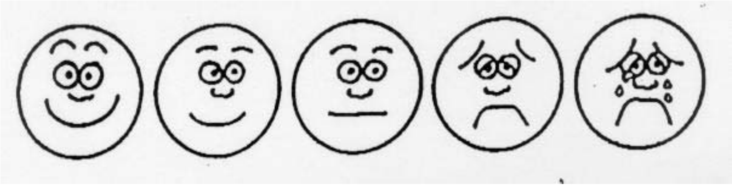

15. Wie einsam fühlst Du Dich wegen Deiner Krankheit?

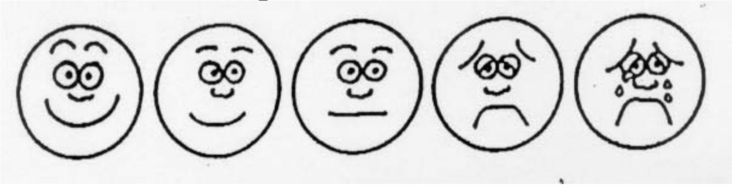

16. Welches Gefühl gibt Dir Deine Familie?

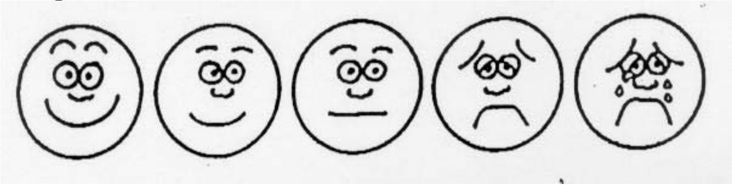

17. Welches Gefühl lösen die krankheitsbedingten Schmerzen bei Dir aus?

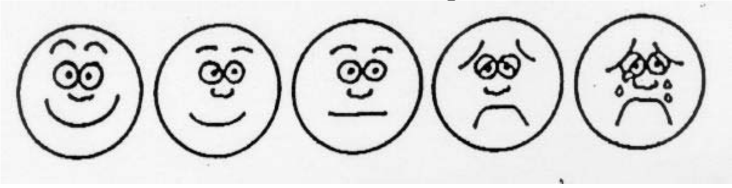

18. Wie findest Du es, wegen deiner Krankheit nicht in die Sonne gehen zu können?

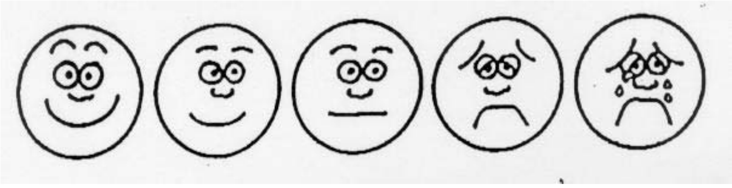

Copyright © 2007 Lakshmi Nandini Moorthy. Alle Rechte vorbehalten.

19. Wie müde macht Dich Deine Krankheit?

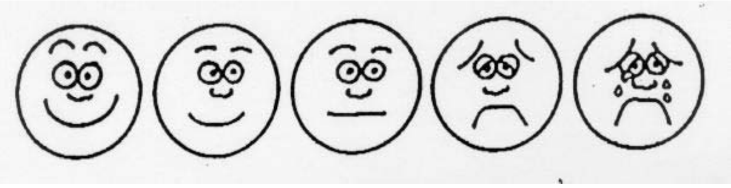

Beantworte die Fragen 20 A- 26 A mit "ja" oder "nein." Bitte folge dann den Anweisungen.

20 A. Mein Freundeskreis hat sich wegen meiner Krankheit verändert. Bitte markiere "ja" oder "nein." Ja Nein

Wenn Du "ja" gewählt hast, gehe zu Frage 20 B.
20 B. Welches Gefühl gibt Dir die Tatsache Deiner Erkrankung mit Blick auf Deine Freunde? Bitte markiere das Gesicht, das zeigt, wie Du Dich fühlst.

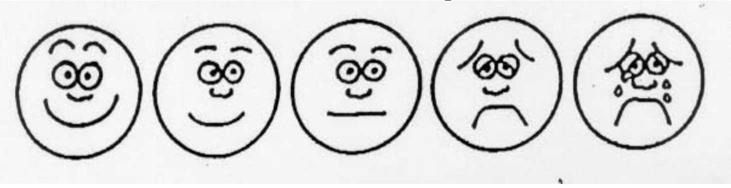

21 A. Meine Freunde haben Verständnis dafür, dass ich eine Krankheit habe.

Ja Nein

Wenn Du "Nein" gewählt hast, gehe zu Frage 21 B.
21 B. Wie findest Du es, wenn Deine Freunde nicht verstehen, dass Du eine Krankheit hast? Bitte markiere das Gesicht, das zeigt, wie Du Dich fühlst.

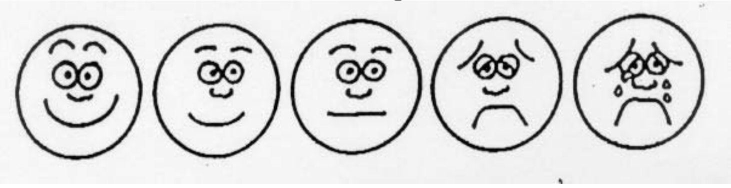

22 A. Meine Krankheit beeinträchtigt meine schulischen Leistungen. Ja Nein
Wenn Du "ja" gewählt hast, gehe zu *Frage 22 B*
22 B. Welches Gefühl gibt Dir Deine Erkrankung bezogen auf Deine schulischen Leistungen? Bitte markiere das Gesicht, das zeigt, wie Du Dich fühlst.

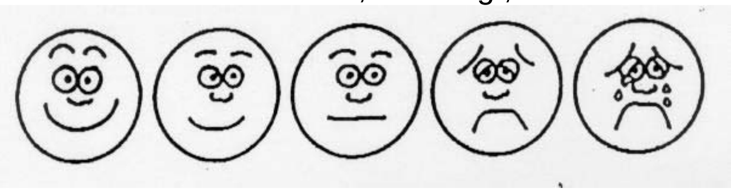

Copyright © 2007 Lakshmi Nandini Moorthy. Alle Rechte vorbehalten.

23 A. Ich versäume wegen meiner Krankheit die Teilnahme an der Schule.

Ja **Nein**

Wenn Du "ja" gewählt hast, gehe zu Frage 23 B.

23 B. Wie findest Du es, wegen deiner Erkrankung die Teilnahme an der Schule zu versäumen?

Bitte markiere das Gesicht, das zeigt, wie Du Dich fühlst.

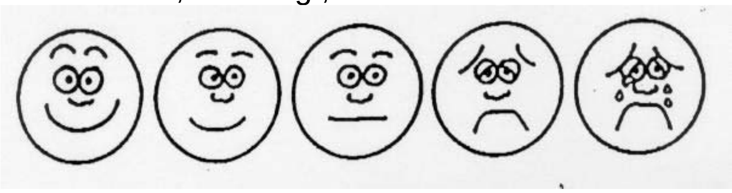

24 A. Die Krankheit beeinträchtigt meine außerschulischen Aktivitäten.

Ja **Nein**

Wenn Du "ja" gewählt hast, gehe zu Frage 24 B.

24 B. Wie findest Du es, dass Deine Erkrankung Deine außerschulischen Aktivitäten beeinträchtigt? Bitte markiere das Gesicht, das zeigt, wie Du Dich fühlst.

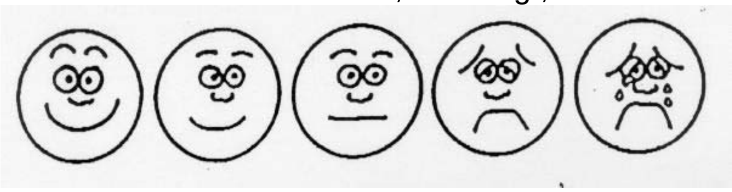

25 A. Die Krankheit beeinträchtigt mich dabei, Spaß mit meinen Freunden zu haben.

Ja **Nein**

Wenn Du "ja" gewählt hast, gehe zu Frage 25 B.

25 B. Wie findest Du es, dass deine Erkrankung Dich dabei beeinträchtigt, Spaß mit Deinen Freunden zu haben? Bitte markiere das Gesicht, das zeigt, wie Du Dich fühlst.

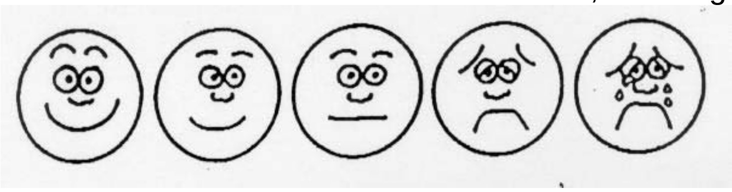

Copyright © 2007 Lakshmi Nandini Moorthy. Alle Rechte vorbehalten.

26 A. Die Krankheit beeinträchtigt mich bei körperlichen Betätigungen (wie zum Beispiel beim Sport in der Schule, beim Laufen, Gehen oder beim Spielen eines Lieblingsspiels draußen).

Ja **Nein**

Wenn Du "ja" gewählt hast, gehe zu Frage 26B.

26 B. Wie findest Du es, dass deine Erkrankung Dich bei Deinen körperlichen Betätigungen (wie zum Beispiel beim Sport in der Schule, beim Laufen, Gehen oder beim Spielen eines Lieblingsspiels draußen) beeinträchtigt? Bitte markiere das Gesicht, das zeigt, wie Du Dich fühlst.

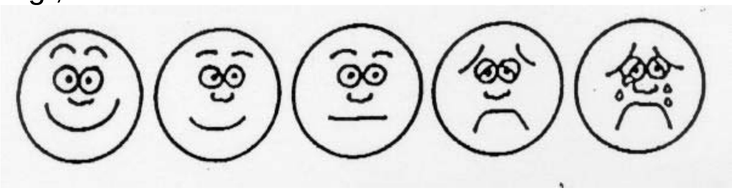

Copyright © 2007 Lakshmi Nandini Moorthy. Alle Rechte vorbehalten.

EINFACHE MESSUNG DER AUSWIRKUNGEN DER KRANKHEIT BEI KINDERN UND JUGENDLICHEN (SMILY-Krankheit) – Elternfragebogen

Nr. der Studie __ Datum __/__/20

Für diesen Fragebogen haben wir die 5 Gesichter aus der Wong-Baker GESICHTER-Skala zur Einstufung des Schmerzempfindens ausgewählt und sie mit Erlaubnis von D.L. Wong modifiziert. Diese 5 Gesichter werden unter jeder Frage erscheinen.

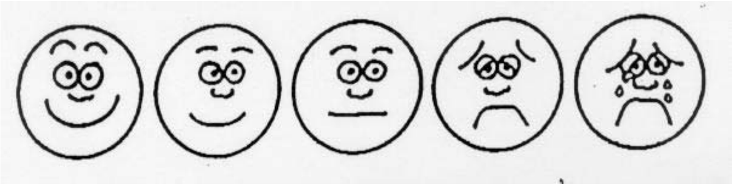

Aus der Publikation von Wong DL, Hockenberry-Eaton M, Wilson D,Winklestein ML, Schwartz P.: *Wong's Essentials of Pediatric Nursing*, 6. Aufl., St. Louis, 2001, Verlag Mosby, S. 1301. Urheberrechtlich geschützt, Verlag Mosby. Nachdruck mit Erlaubnis des Berechtigten.

Inhaberin des Urheberrechts an SMILY ist L. Nandini Moorthy, MD MS, RWJMS-UMDNJ, New Brunswick, NJ. Derzeit darf der Fragebogen ausschließlich von L. Nandini Moorthy, MD MS und durch von ihr ermächtigtes Studienpersonal verwendet werden.

FRAGEN

In diesem Fragebogen stellen wir Ihnen viele Fragen über die Erkrankung Ihres Kindes und über sein Befinden und seine Gefühle. Es gibt keine falschen Antworten. Bitte fragen Sie uns, wenn Sie etwas nicht verstehen. Wenn Sie etwas anmerken möchten, schreiben Sie Ihre Anmerkungen bitte im Anschluss an die Seiten 5 und 6 auf.

Verschiedene Dinge beeinflussen das Leben Ihres Kindes und stimmen es glücklich oder traurig. Es gibt einige Dinge im Leben Ihres Kindes, die es für wichtig hält und die es tun kann (oder nicht tun kann). Alle diese verschiedenen Dinge im Leben Ihres Kindes machen insgesamt seine Lebensqualität aus.

1. **Wie ist die Lebensqualität Ihres Kindes? Bitte markieren Sie das Gesicht, das am besten seine Lebensqualität wiedergibt.**

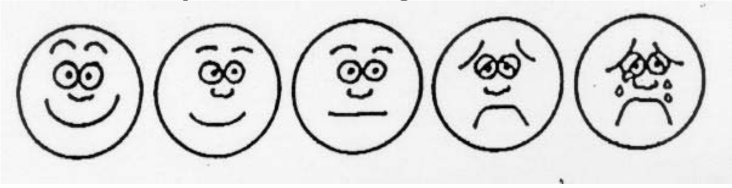

2. **Wie ist die Krankheit Ihres Kindes derzeit? Bitte markieren Sie das Gesicht, das am besten wiedergibt, wie die Krankheit Ihres Kindes ist.**

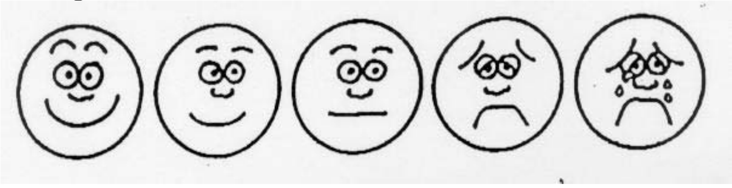

Copyright © 2007 Lakshmi Nandini Moorthy. Alle Rechte vorbehalten.

Fragen 3-19 betreffen das durch die Krankheit verursachte Empfinden Ihres Kindes während des letzten Monats. Bitte markieren Sie das Gesicht, das Ihre Antwort am besten wiedergibt.

3. Welche Gefühle löst die Tatsache der Erkrankung bei Ihrem Kind aus?

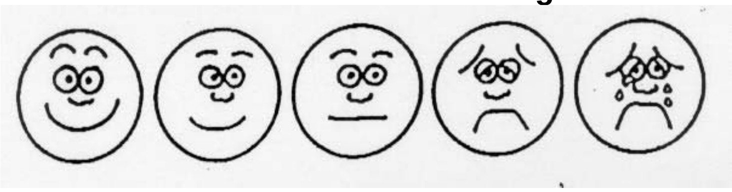

4. Wie ist es für Ihr Kind, mit der Erkrankung die Schule zu besuchen?

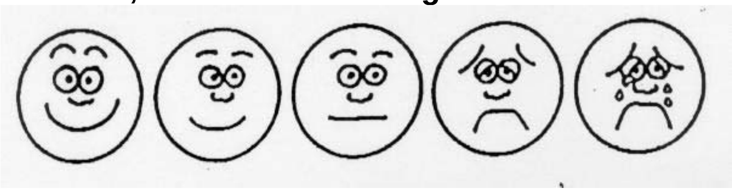

5. Wie ist es für Ihr Kind, sich mit der Erkrankung jeden Tag anzuziehen?

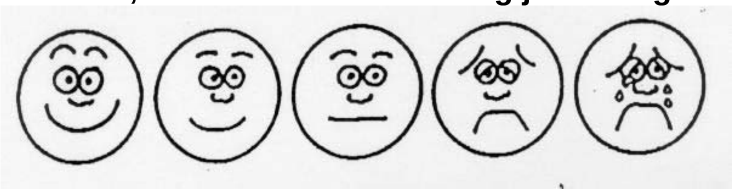

6. Welche Gefühle löst die Tatsache der Erkrankung bei Ihrem Kind mit Blick auf sein/ihr Aussehen aus?

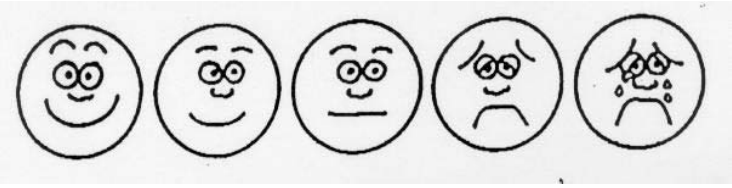

7. Wie beeinflusst die Tatsache der Erkrankung das Selbstempfinden Ihres Kindes?

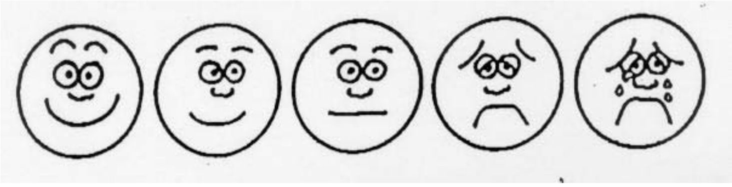

Copyright © 2007 Lakshmi Nandini Moorthy. Alle Rechte vorbehalten.

8. Wie fühlt sich Ihr Kind, wenn es über seine/ihre Erkrankung reden muss?

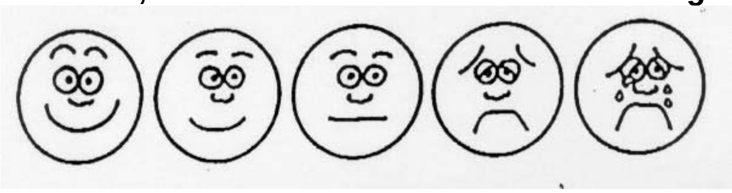

9. Wie empfindet es Ihr Kind, wegen der Erkrankung zum Arzt zu gehen?

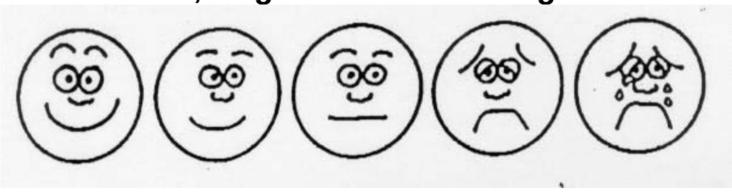

10. Wie empfindet es Ihr Kind, wegen der Erkrankung das Krankenhaus aufzusuchen?

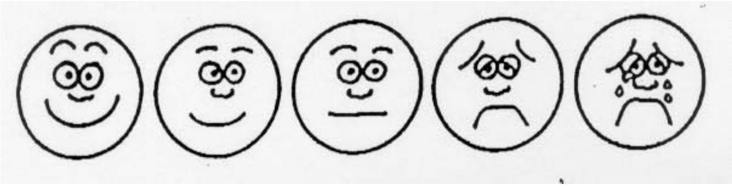

11. Welche Gefühle löst die Tatsache der Erkrankung bei Ihrem Kind mit Blick auf seine/ihre Zukunft aus?

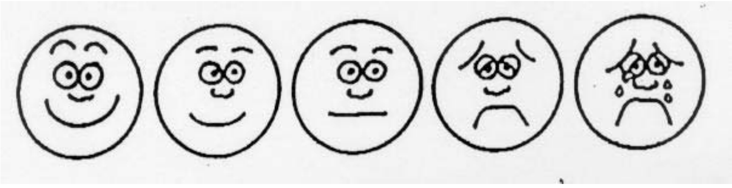

12. Wie fühlt sich Ihr Kind damit, wegen der Erkrankung Medikamente zu nehmen?

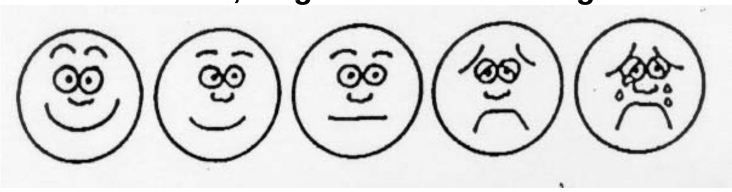

13. Wie besorgt ist Ihr Kind über Nebenwirkungen der Medikamente, die es gegen die Krankheit nimmt?

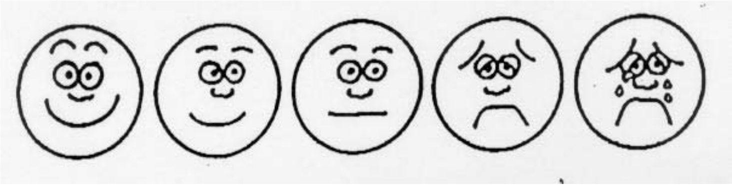

Copyright © 2007 Lakshmi Nandini Moorthy. Alle Rechte vorbehalten.

14. Wie besorgt ist Ihr Kind darüber, dass die Erkrankung sich verschlechtern könnte?

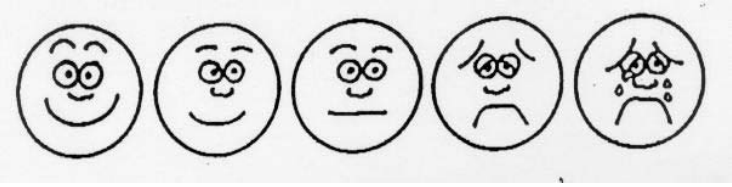

15. Wie einsam fühlt sich Ihr Kind wegen der Krankheit?

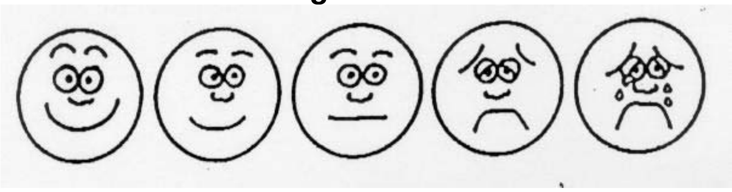

16. Welches Gefühl wird Ihrem Kind durch seine/ihre Familie vermittelt?

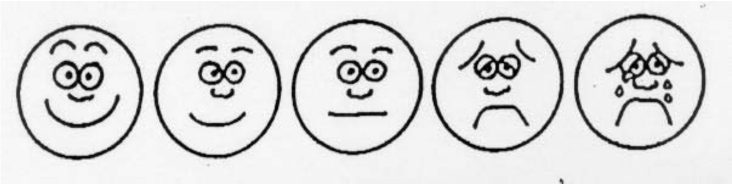

17. Welches Gefühl lösen die Krankheits-Schmerzen bei Ihrem Kind aus?

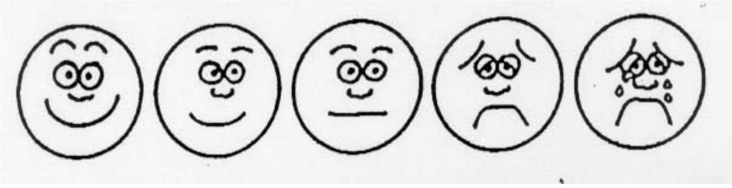

18. Was empfindet Ihr Kind dabei, wegen der Krankheit nicht in die Sonne gehen zu können?

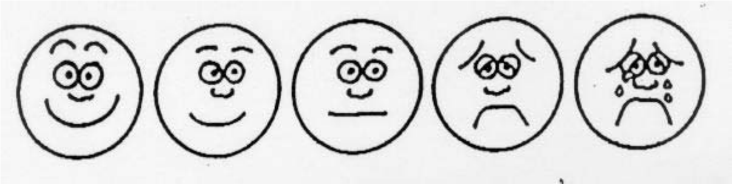

19. Wie müde macht die Erkrankung Ihr Kind?

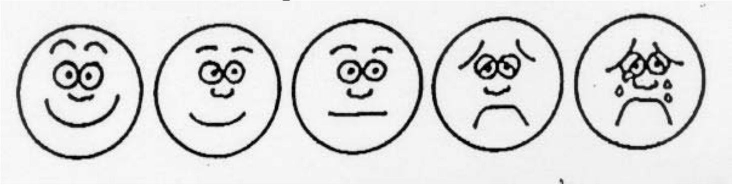

Copyright © 2007 Lakshmi Nandini Moorthy. Alle Rechte vorbehalten.

Beantworten Sie die Fragen 20 A- 26 A mit "ja" oder "nein." Bitte folgen Sie dann den Anweisungen.

20 A. Der Freundeskreis Ihres Kindes hat sich wegen seiner/ihrer Erkrankung verändert. Bitte markieren Sie "ja" oder "nein." Ja Nein

Bei "ja", gehen Sie zu Frage 20 B.

20 B. Welches Gefühl gibt die Tatsache der Erkrankung Ihrem Kind mit Blick auf seine/ihre Freunde?

Bitte markieren Sie das Gesicht, das zeigt, wie sich Ihr Kind fühlt.

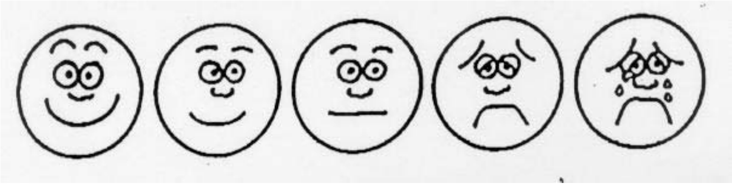

21 A. Die Freunde Ihres Kindes haben Verständnis dafür, dass es erkrankt ist.

Bei "nein", gehen Sie zu Frage 21 B **Ja Nein**

21 B. Was empfindet Ihr Kind dabei, wenn seine/ihre Freunde kein Verständnis dafür haben, dass es eine Krankheit hat? Bitte markieren Sie das Gesicht, das zeigt, wie sich Ihr Kind fühlt.

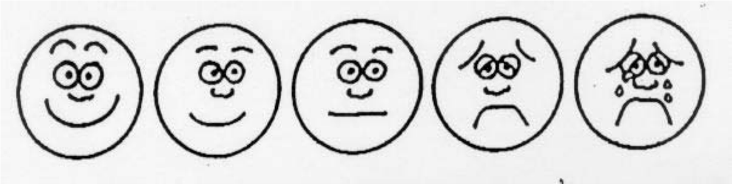

22 A. Die Krankheit beeinträchtigt die schulischen Leistungen Ihres Kindes.

Bei "ja", gehen Sie zu Frage *22 B* **Ja Nein**

22 B. Wie empfindet Ihr Kind die Tatsache seiner/ihrer Erkrankung bezogen auf seine/ihre Mitarbeit in der Schule?

Bitte markieren Sie das Gesicht, das zeigt, wie sich Ihr Kind fühlt.

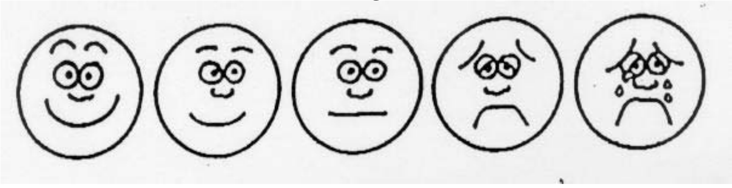

Copyright © 2007 Lakshmi Nandini Moorthy. Alle Rechte vorbehalten.

23 A. Ihr Kind versäumt wegen der Erkrankung die Teilnahme an der Schule.

Bei “ja”, gehen Sie zu Frage 23 B. **Ja** **Nein**

23 B. Was empfindet Ihr Kind dabei, wegen der Erkrankung die Teilnahme an der Schule zu versäumen?

Bitte markieren Sie das Gesicht, das zeigt, wie sich Ihr Kind fühlt.

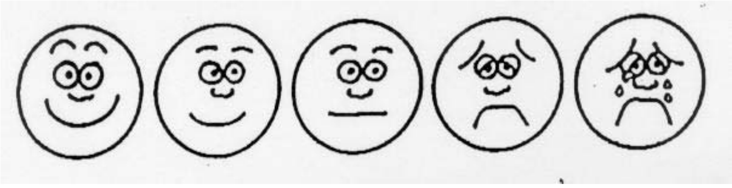

24 A. Die Erkrankung beeinträchtigt die außerschulischen Aktivitäten Ihres Kindes.

Ja **Nein**

Bei “ja”, gehen Sie zu Frage 24 B.

24 B. Was empfindet Ihr Kind dabei, dass die Erkrankung seine/ihre außerschulischen Aktivitäten beeinträchtigt? Bitte markieren Sie das Gesicht, das zeigt, wie sich Ihr Kind fühlt.

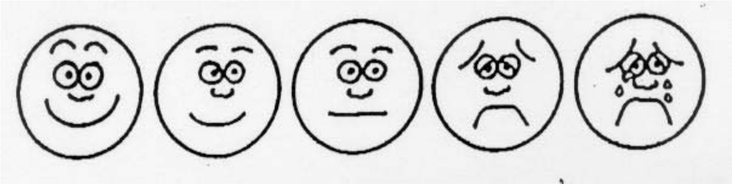

25 A. Die Erkrankung beeinträchtigt Ihr Kind darin, Spaß mit seinen Freunden zu haben.

Ja **Nein**

Bei “ja”, gehen Sie zu Frage 25 B.

25 B. Was empfindet Ihr Kind dabei, dass die Erkrankung ihn/sie darin beeinträchtigt, Spaß mit seinen/ihren Freunden zu haben? Bitte markieren Sie das Gesicht, das zeigt, wie sich Ihr Kind fühlt.

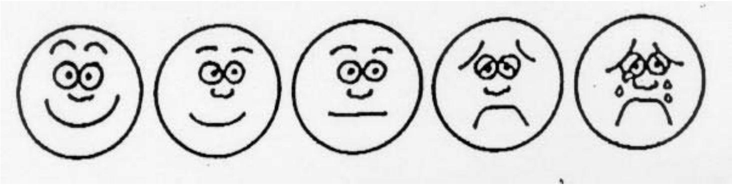

Copyright © 2007 Lakshmi Nandini Moorthy. Alle Rechte vorbehalten.

26 A. Die Erkrankung beeinträchtigt Ihr Kind in seinen/ihren körperlichen Betätigungen (wie zum Beispiel beim Sport in der Schule, beim Laufen, Gehen oder beim Spielen eines Lieblingsspiels draußen).

Ja **Nein**

Bei "ja", gehen Sie zu Frage 26B.

26 B. Was empfindet Ihr Kind dabei, dass die Erkrankung ihn/sie in seinen/ihren körperlichen Betätigungen (wie zum Beispiel beim Sport in der Schule, beim Laufen, Gehen oder beim Spielen eines Lieblingsspiels draußen) beeinträchtigt? Bitte markieren Sie das Gesicht, das zeigt, wie sich Ihr Kind fühlt.

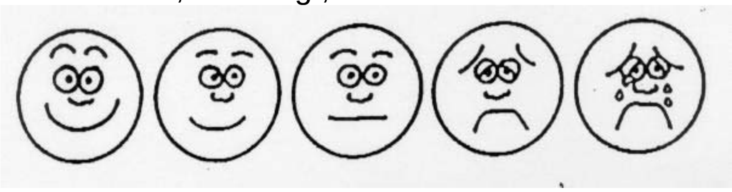

Copyright © 2007 Lakshmi Nandini Moorthy. Alle Rechte vorbehalten.

EINFACHE MESSUNG DER KRANKHEITSAUSWIRKUNGEN AUF KINDER UND JUGENDLICHE (SMILY-Krankheitsfragebogen) – Elternfragebogen (GERMAN FOR SWITZERLAND)

German Swiss

Nr. der Studie __ Datum __/__/20__

Für diesen Fragebogen haben wir die 5 Gesichter aus der Wong-Baker-GESICHTER-Skala zur Einstufung von Schmerzempfinden ausgewählt und mit Erlaubnis von D.L. Wong modifiziert. Diese 5 Gesichter werden unter jeder Frage erscheinen.

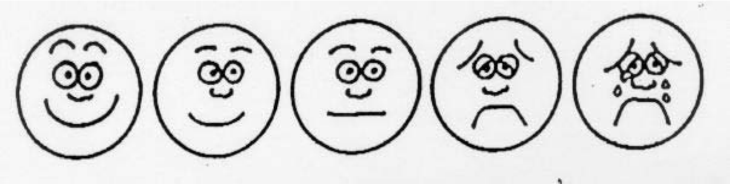

Aus der Publikation von Wong DL, Hockenberry-Eaton M, Wilson D,Winklestein ML, Schwartz P.: *Wong's Essentials of Pediatric Nursing*, 6. Aufl., St. Louis, 2001, Verlag Mosby, S. 1301. Urheberrechtlich geschützt, Verlag Mosby. Autorisierter Nachdruck.

Inhaberin des Urheberrechts am SMILY-Fragebogen ist L. Nandini Moorthy, MD MS, RWJMS-UMDNJ, New Brunswick im US-Bundesstaat New Jersey. Derzeit darf der Fragebogen ausschliesslich von L. Nandini Moorthy (MD MS) und durch von ihr dazu ermächtigtes Studienpersonal verwendet werden.

FRAGEN

In diesem Fragebogen stellen wir Ihnen viele Fragen über die Krankheit Ihres Kindes und über sein Befinden und seine Gefühle. Es gibt keine falschen Antworten. Bitte fragen Sie uns, wenn Sie etwas nicht verstehen. Wenn Sie etwas anmerken möchten, schreiben Sie Ihre Anmerkungen bitte im Anschluss an die Seiten 5 und 6 auf.

Verschiedene Dinge beeinflussen das Leben Ihres Kindes und stimmen es glücklich oder traurig. Es gibt einige Dinge im Leben Ihres Kindes, die es für wichtig hält und die es tun kann (oder nicht tun kann). All diese verschiedenen Dinge im Leben Ihres Kindes machen insgesamt seine Lebensqualität aus.

1. Wie ist die Lebensqualität Ihres Kindes? Bitte markieren Sie das Gesicht, das am besten seine Lebensqualität wiedergibt.

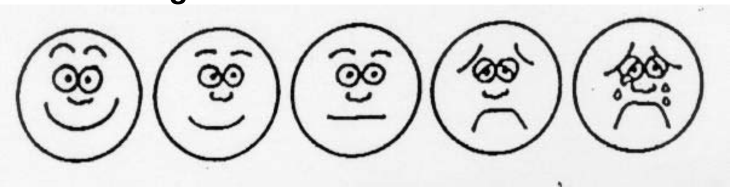

2. Wie ist der Krankheitszustand Ihres Kindes derzeit? Bitte markieren Sie das Gesicht, das am besten wiedergibt, wie der Krankheitszustand Ihres Kindes ist.

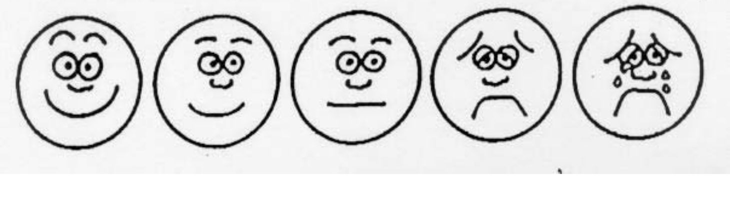

Copyright © 2007 Lakshmi Nandini Moorthy. Alle Rechte vorbehalten.

Fragen 3-19 betreffen das durch die Krankheit verursachte Empfinden Ihres Kindes im letzten Monat. Bitte markieren Sie das Gesicht, das Ihre Antwort am besten wiedergibt.

3. Welche Gefühle löst die Tatsache, dass es eine Krankheit hat, bei Ihrem Kind aus?

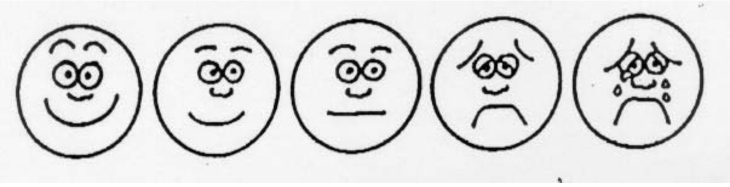

4. Wie ist es für Ihr Kind, mit einer Krankheit die Schule zu besuchen?

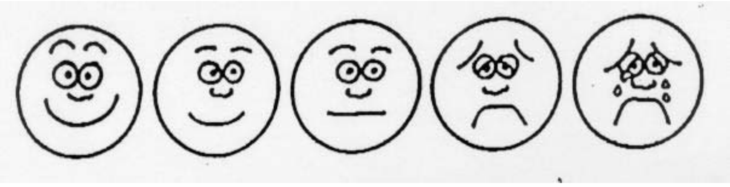

5. Wie ist es für Ihr Kind, sich mit einer Krankheit jeden Tag anzuziehen?

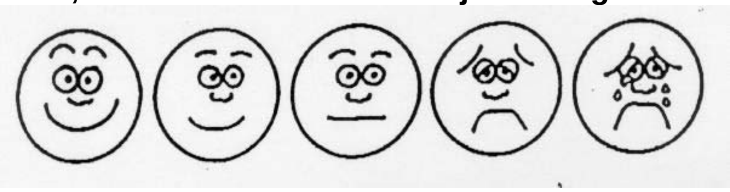

6. Welche Gefühle löst die Tatsache, dass es eine Krankheit hat, bei Ihrem Kind mit Blick auf sein Aussehen aus?

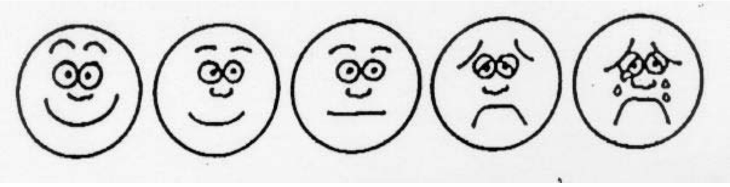

7. Wie beeinflusst die Tatsache, dass es eine Krankheit hat, das Selbstempfinden Ihres Kindes?

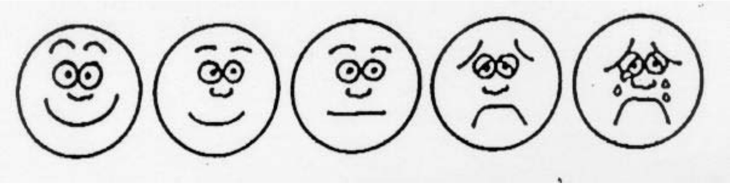

8. Wie fühlt sich Ihr Kind, wenn es über seine Krankheit reden muss?

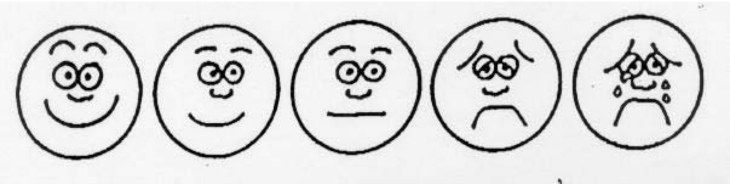

Copyright © 2007 Lakshmi Nandini Moorthy. Alle Rechte vorbehalten.

9. Wie empfindet es Ihr Kind, wegen der Krankheit zum Arzt zu gehen?

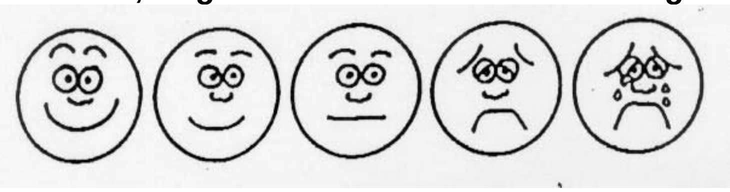

10. Wie empfindet es Ihr Kind, wegen der Krankheit das Spital aufzusuchen?

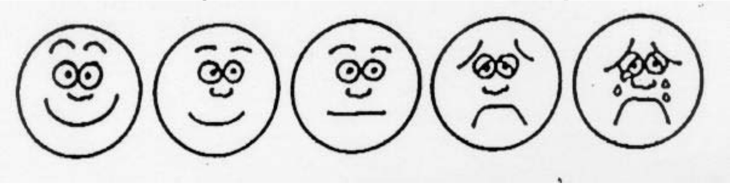

11. Welche Gefühle löst die Tatsache, eine Krankheit zu haben, bei Ihrem Kind mit Blick auf seine Zukunft aus?

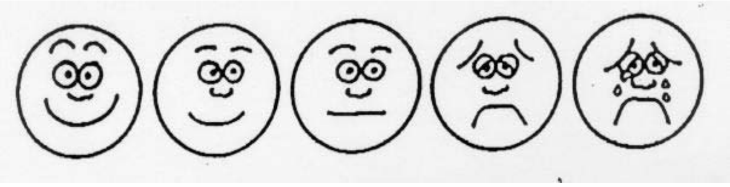

12. Wie fühlt sich Ihr Kind damit, wegen seiner Krankheit Medikamente zu nehmen?

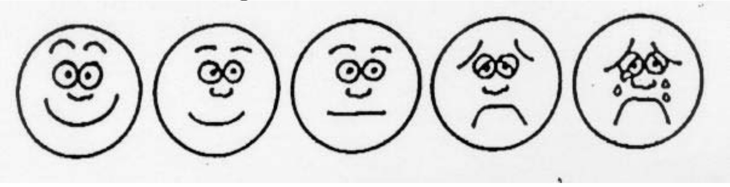

13. Wie besorgt ist Ihr Kind über Nebenwirkungen der Medikamente, die es gegen seine Krankheit nimmt?

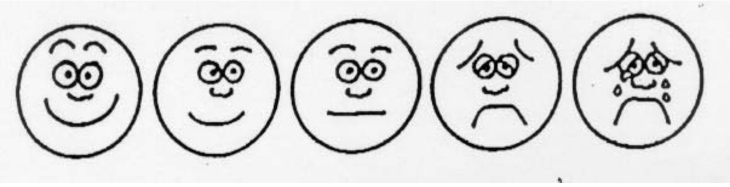

14. Wie besorgt ist Ihr Kind darüber, dass sich die Krankheit verschlimmern könnte?

Copyright © 2007 Lakshmi Nandini Moorthy. Alle Rechte vorbehalten.

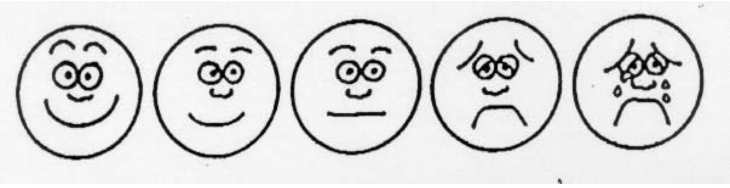

15. Wie einsam fühlt sich Ihr Kind wegen der Krankheit?

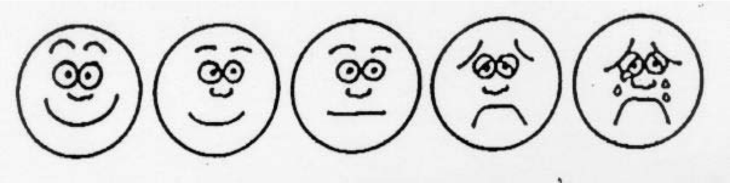

16. Welches Gefühl wird Ihrem Kind durch seine Familie vermittelt?

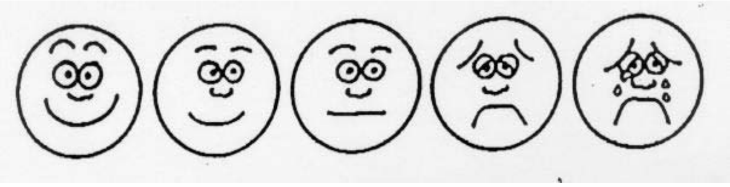

17. Welches Gefühl lösen die durch die Krankheit hervorgerufenen Schmerzen bei Ihrem Kind aus?

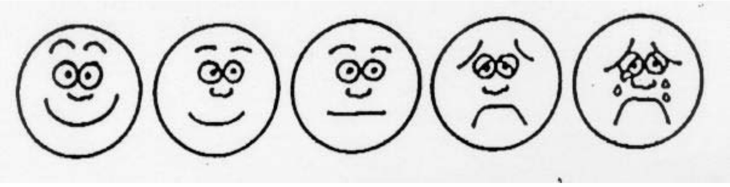

18. Was empfindet Ihr Kind dabei, wegen der Krankheit nicht in die Sonne gehen zu können?

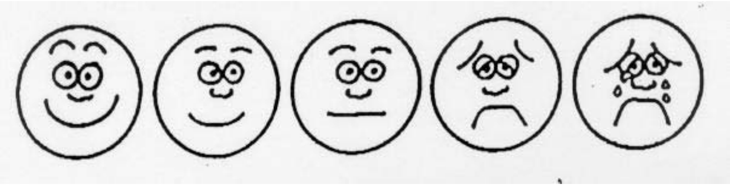

19. Wie müde macht die Krankheit Ihr Kind?

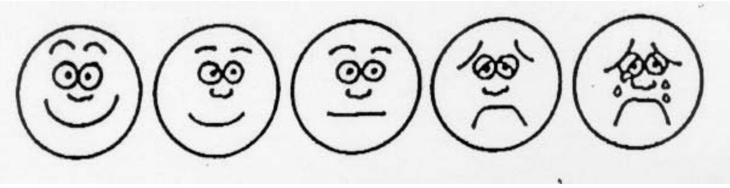

Copyright © 2007 Lakshmi Nandini Moorthy. Alle Rechte vorbehalten.

Beantworten Sie die Fragen 20 A bis 26 A mit „ja" oder „nein". Bitte folgen Sie dann den Anweisungen.

20 A. Der Freundeskreis Ihres Kindes hat sich wegen seiner Krankheit verändert. Bitte markieren Sie „ja" oder „nein".

Ja **Nein**

Bei „ja" gehen Sie zu Frage 20 B.

20 B. Welches Gefühl gibt die Tatsache, dass es eine Krankheit hat, Ihrem Kind mit Blick auf seine Freunde? Bitte markieren Sie das Gesicht, das zeigt, wie sich Ihr Kind fühlt.

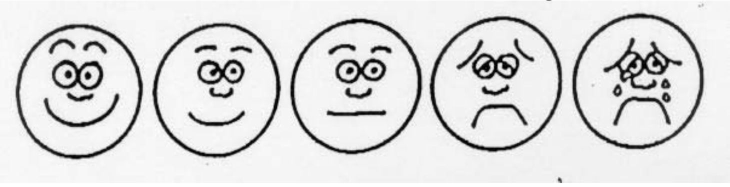

21 A. Die Freunde Ihres Kindes haben Verständnis dafür, dass es an einer Krankheit leidet.

Ja **Nein**

Bei „nein" gehen Sie zu Frage 21 B.

21 B. Was empfindet Ihr Kind dabei, wenn seine Freunde kein Verständnis dafür haben, dass es eine Krankheit hat? Bitte markieren Sie das Gesicht, das zeigt, wie sich Ihr Kind fühlt.

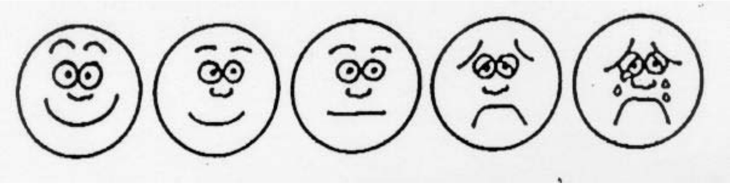

22 A. Die Krankheit beeinträchtigt die schulischen Leistungen Ihres Kindes.

Ja **Nein**

Bei „ja" gehen Sie zu Frage *22 B*

22 B. Wie empfindet Ihr Kind die Tatsache, dass es an einer Krankheit leidet, bezogen auf seine Mitarbeit in der Schule? Bitte markieren Sie das Gesicht, das zeigt, wie sich Ihr Kind fühlt.

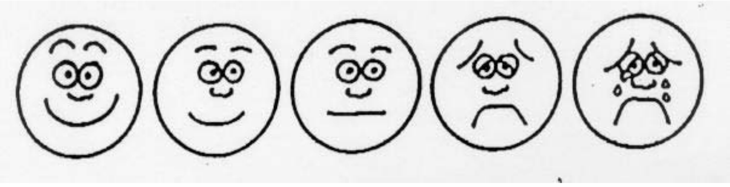

Copyright © 2007 Lakshmi Nandini Moorthy. Alle Rechte vorbehalten.

23 A. Ihr Kind versäumt wegen der Krankheit die Teilnahme an der Schule.

Ja **Nein**

Bei „ja” gehen Sie zu Frage 23 B.

23 B. Was empfindet Ihr Kind dabei, wegen der Krankheit die Teilnahme an der Schule zu versäumen? Bitte markieren Sie das Gesicht, das zeigt, wie sich Ihr Kind fühlt.

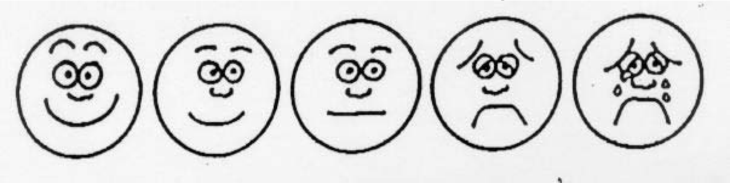

24 A. Die Krankheit beeinträchtigt die Aktivitäten in der Freizeit Ihres Kindes.

Ja **Nein**

Bei „ja”, gehen Sie zu Frage 24 B.

24 B. Was empfindet Ihr Kind dabei, dass die Krankheit seine Freizeitaktivitäten beeinträchtigt? Bitte markieren Sie das Gesicht, das zeigt, wie sich Ihr Kind fühlt.

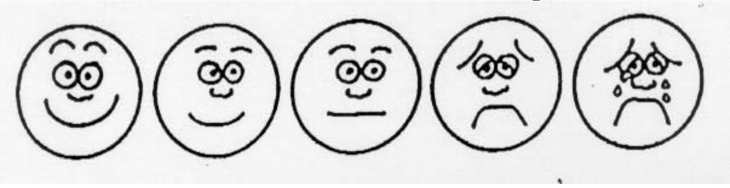

25 A. Die Krankheit beeinträchtigt Ihr Kind darin, Spass mit seinen Freunden zu haben.

Ja **Nein**

Bei „ja” gehen Sie zu Frage 25 B.

25 B. Was empfindet Ihr Kind dabei, dass die Krankheit es darin beeinträchtigt, Spass mit seinen Freunden zu haben? Bitte markieren Sie das Gesicht, das zeigt, wie sich Ihr Kind fühlt.

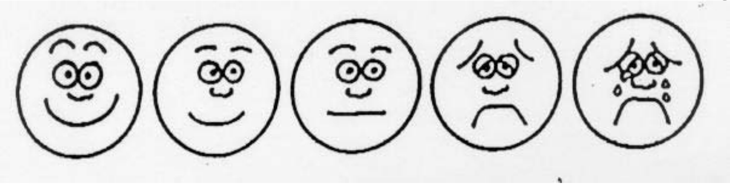

Copyright © 2007 Lakshmi Nandini Moorthy. Alle Rechte vorbehalten.

26 A. Die Krankheit beeinträchtigt Ihr Kind in seiner körperlichen Betätigung (wie zum Beispiel beim Sport in der Schule, beim Laufen, Gehen oder beim Spielen eines Lieblingsspiels draussen).

Ja **Nein**

Bei „ja" gehen Sie zu Frage 26B..

26 B. Was empfindet Ihr Kind dabei, dass die Krankheit es in seiner körperlichen Betätigung (wie zum Beispiel beim Sport in der Schule, beim Laufen, Gehen oder beim Spielen eines Lieblingsspiels draussen) beeinträchtigt? Bitte markieren Sie das Gesicht, das zeigt, wie sich Ihr Kind fühlt.

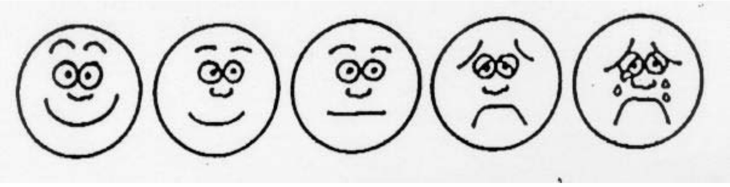

Copyright © 2007 Lakshmi Nandini Moorthy. Alle Rechte vorbehalten.

EINFACHE MESSUNG DER KRANKHEITSAUSWIRKUNGEN AUF KINDER UND JUGENDLICHE (SMILY-Krankheitsfragebogen) – Kinderfragebogen

Nr. der Studie __ Datum __/__/20__

Für diesen Fragebogen haben wir die 5 Gesichter aus der Wong-Baker-GESICHTER-Skala zur Einstufung von Schmerzempfinden ausgewählt und mit Erlaubnis von D.L. Wong angepasst. Diese 5 Gesichter werden unter jeder Frage erscheinen.

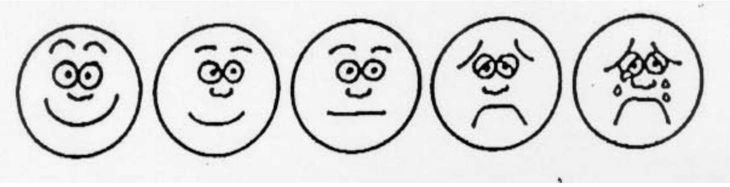

Aus der Publikation von Wong DL, Hockenberry-Eaton M, Wilson D,Winklestein ML, Schwartz P.: *Wong's Essentials of Pediatric Nursing*, 6. Aufl., St. Louis, 2001, Verlag Mosby, S. 1301. Urheberrechtlich geschützt, Verlag Mosby. Autorisierter Nachdruck.

Inhaberin des Urheberrechts am SMILY-Krankheitsfragebogen ist L. Nandini Moorthy, MD MS, RWJMS-UMDNJ, New Brunswick im US-Bundesstaat New Jersey. Derzeit darf der Fragebogen ausschliesslich von L. Nandini Moorthy (MD MS) und durch von ihr ermächtigtes Studienpersonal verwendet werden.

FRAGEN

In diesem Fragebogen stellen wir dir viele Fragen über deine Krankheit und über dein Befinden und deine Gefühle. Es gibt keine falschen Antworten. Bitte frage uns, wenn du etwas nicht verstehst. Wenn du etwas anmerken möchtest, schreibe deine Anmerkungen bitte im Anschluss an die Seiten 5 und 6 auf.

Verschiedene Dinge beeinflussen dein Leben und machen dich glücklich oder traurig. Es gibt Dinge in deinem Leben, die für dich wichtig sind und die du machen kannst (oder nicht machen kannst). Alle diese verschiedenen Dinge in deinem Leben machen insgesamt deine Lebensqualität aus.

1. Wie ist deine Lebensqualität? Bitte markiere das Gesicht, das am besten deine Lebensqualität wiedergibt.

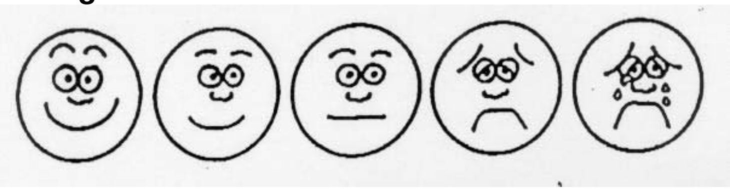

2. Wie ist deine Krankheit im Moment? Bitte markiere das Gesicht, das am besten deineen Krankheitszustand wiedergibt.

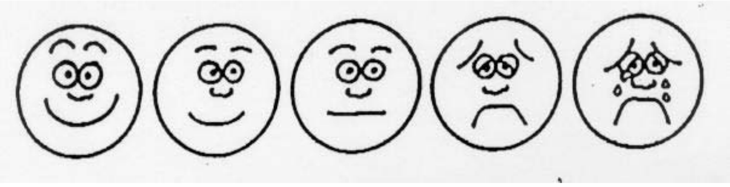

Copyright © 2007 Lakshmi Nandini Moorthy. Alle Rechte vorbehalten.

In den Fragen 3 bis 19 wirst du darüber befragt, wie du dich im letzten Monat wegen deiner Krankheit gefühlt hast.
Bitte markiere das Gesicht, das am besten wiedergibt, wie du dich fühlst.

3. Wie ist es für dich, eine Krankheit zu haben?

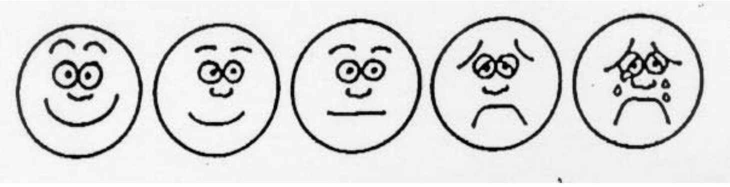

4. Wie ist es für dich, mit einer Krankheit in die Schule zu gehen?

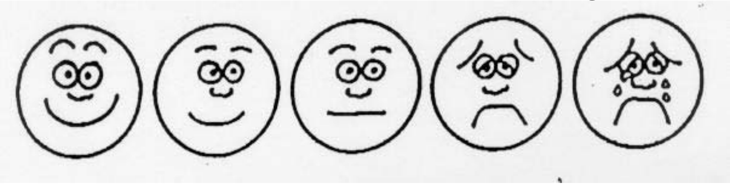

5. Wie fühlst du dich wegen der Krankheit, wenn du dich jeden Tag anziehst?

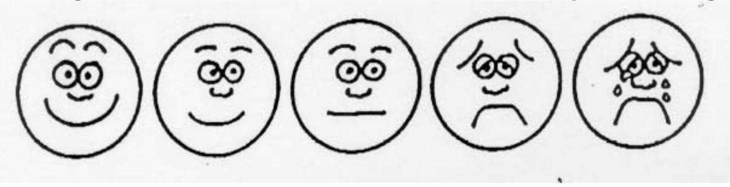

6. Wie findest du dein Aussehen wegen der Krankheit?

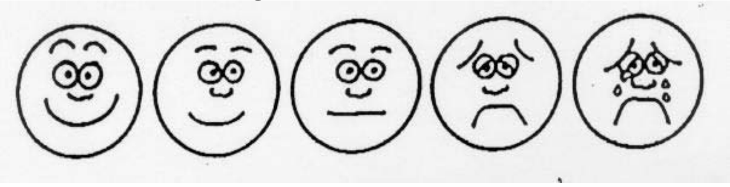

7. Wie beeinflusst die Krankheit dein Selbstempfinden?

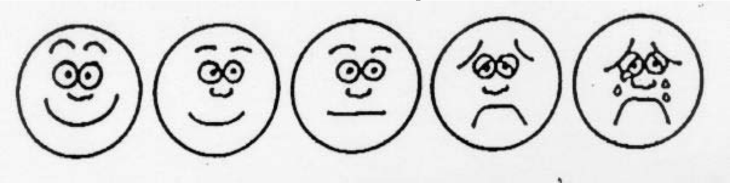

8. Wie fühlst du dich, wenn du über deine Krankheit reden musst?

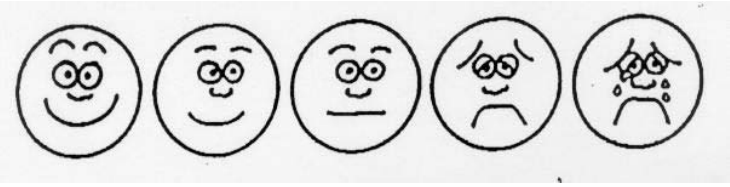

Copyright © 2007 Lakshmi Nandini Moorthy. Alle Rechte vorbehalten.

9. Wie findest du es, wegen deiner Krankheit zum Arzt zu gehen?

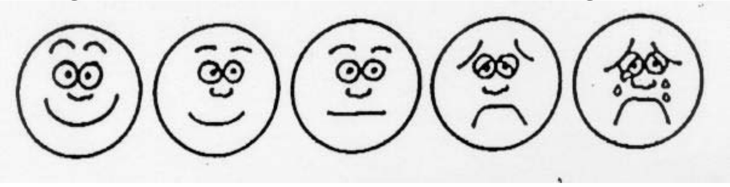

10. Wie findest du es, wegen deiner Krankheit ins Spital zu gehen?

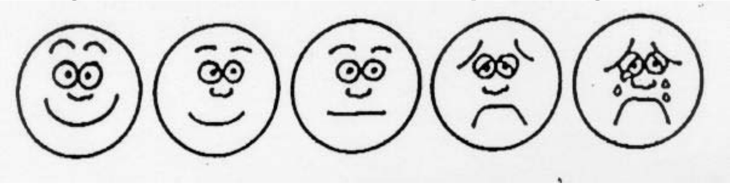

12. Wie fühlst du dich, wenn du an deine Zukunft denkst und die Tatsache, dass du eine Krankheit hast?

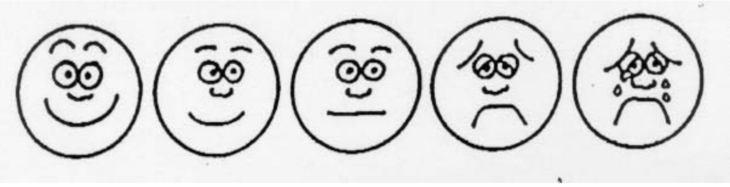

12. Wie findest du es, wegen deiner Krankheit Medikamente zu nehmen?

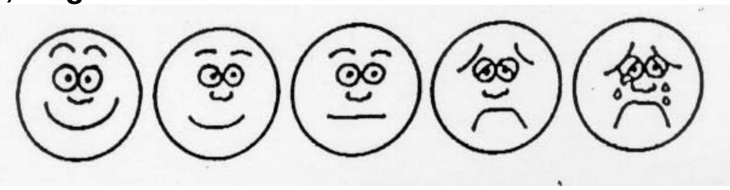

13. Wie besorgt bist du wegen der Nebenwirkungen der Medikamente, die du gegen deine Krankheit nimmst?

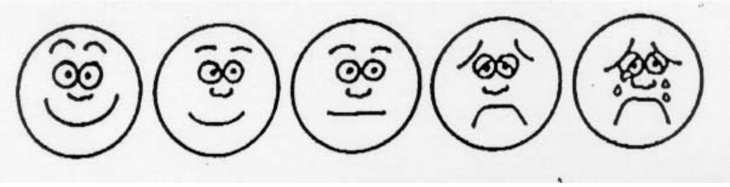

14. Wie besorgt bist du darüber, dass die Krankheit sich verschlimmern könnte?

Copyright © 2007 Lakshmi Nandini Moorthy. Alle Rechte vorbehalten.

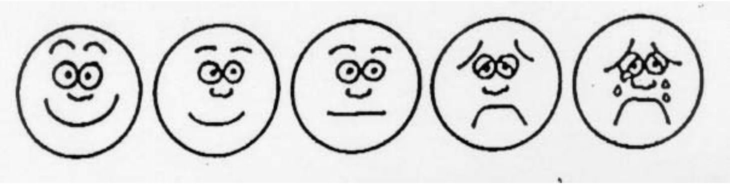

15. **Wie einsam fühlst du dich wegen deiner Krankheit?**

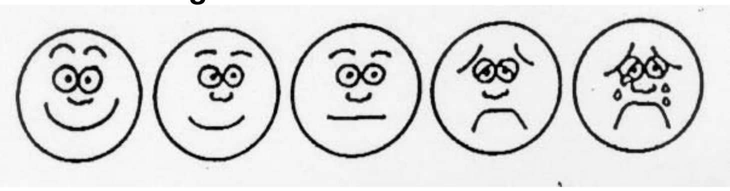

16. **Welches Gefühl gibt dir deine Familie?**

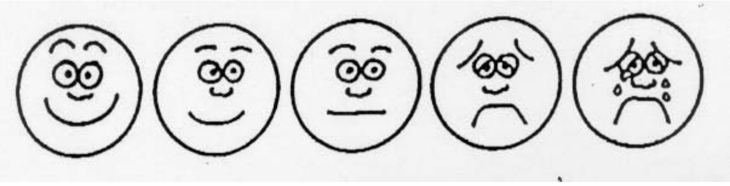

17. **Welches Gefühl lösen die Schmerzen wegen deiner Krankheit bei dir aus?**

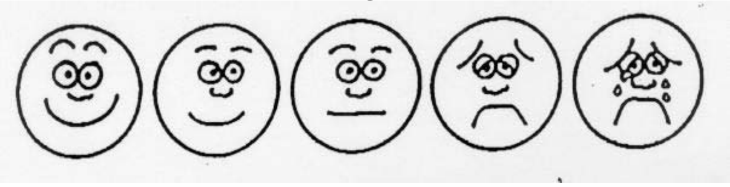

18. **Wie findest du es, wegen deiner Krankheit nicht in die Sonne gehen zu können?**

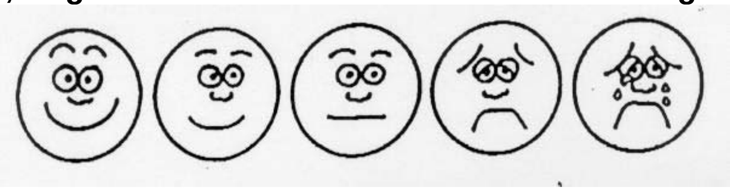

19. **Wie müde macht dich die Krankheit?**

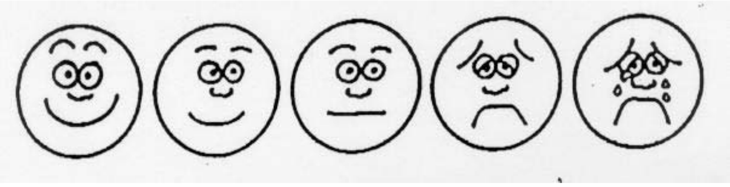

Copyright © 2007 Lakshmi Nandini Moorthy. Alle Rechte vorbehalten.

Beantworte die Fragen 20 A - 26 A mit „ja” oder „nein”. Bitte folge dann den Anweisungen.

20 A. Mein Freundeskreis hat sich verändert wegen meiner Krankheit. Bitte markiere „ja” oder „nein”. Ja Nein

Wenn du „ja” gewählt hast, gehe zu Frage 20 B.

20 B. Welches Gefühl hast du, wenn du an deine Freunde denkst und die Tatsache, dass du eine Krankheit hast? Bitte markiere das Gesicht, das zeigt, wie du dich fühlst.

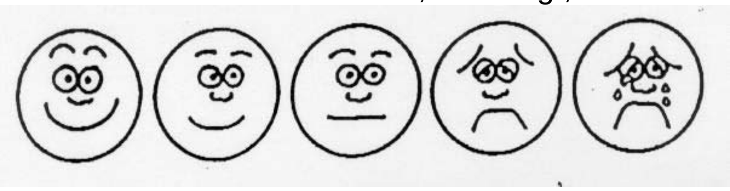

21 A. Meine Freunde verstehen, dass ich eine Krankheit habe.

Ja Nein

Wenn du „nein” gewählt hast, gehe zu Frage 21 B.

21 B. Wie findest du es, wenn deine Freunde nicht verstehen, dass du eine Krankheit hast? Bitte markiere das Gesicht, das zeigt, wie du dich fühlst.

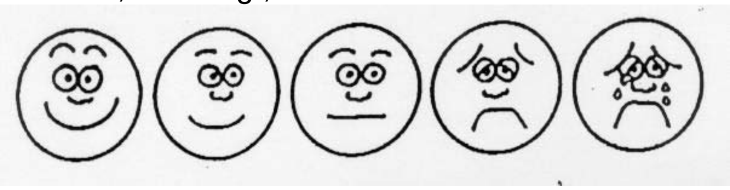

22 A. Die Krankheit beeinflusst meine schulischen Leistungen. Ja Nein

Wenn du „ja” gewählt hast, gehe zu *Frage 22 B*

22 B. Welches Gefühl gibt dir die Krankheit bezogen auf deine Schulleistungen? Bitte markiere das Gesicht, das zeigt, wie du dich fühlst.

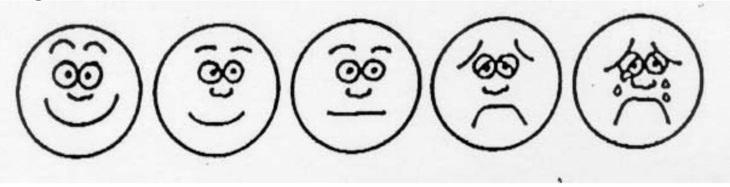

23 A. Ich verpasse wegen der Krankheit die Schule.

Ja Nein

Wenn du „ja” gewählt hast, gehe zu Frage 23 B.

23 B. Wie findest du es, wegen der Krankheit die Schule zu verpassen?

Copyright © 2007 Lakshmi Nandini Moorthy. Alle Rechte vorbehalten.

Bitte markiere das Gesicht, das zeigt, wie du dich fühlst.

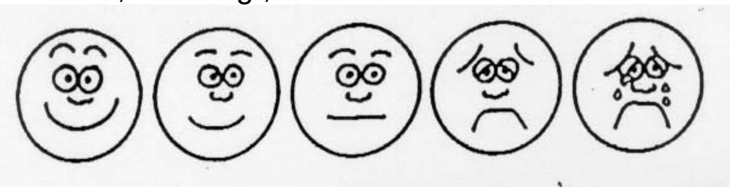

24 A. Die Krakheit beeinflusst meine Freizeitaktivitäten.

Ja **Nein**

Wenn du „ja” gewählt hast, gehe zu Frage 24 B.

24 B. Wie findest du es, dass die Krankheit deine Freizeitaktivitäten beeinflusst? Bitte markiere das Gesicht, das zeigt, wie du dich fühlst.

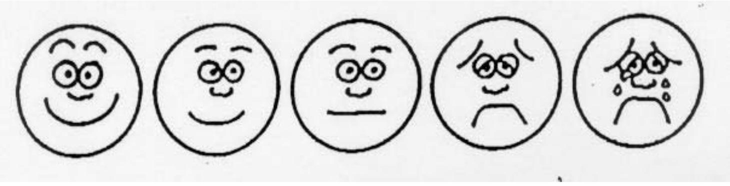

25 A. Die Krankheit beeinflusst, wie ich mit meinen Freunden Spass habe.

Ja **Nein**

Wenn du „ja” gewählt hast, gehe zu Frage 25 B.

25 B. Wie findest du es, dass die Krankheit beeinflusst, wie du mit deinen Freunden Spass hast? Bitte markiere das Gesicht, das zeigt, wie du dich fühlst.

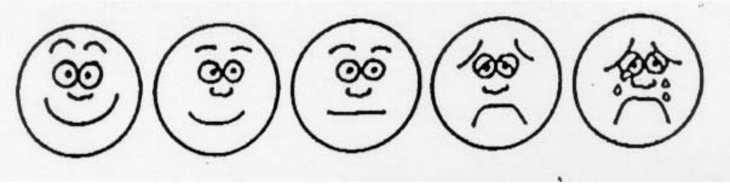

26 A. Die Krankheit beeinträchtigt mich bei körperlichen Aktivitäten (wie zum Beispiel beim Sport in der Schule, beim Laufen, Gehen oder beim Spielen eines Lieblingsspiels draussen).

Ja **Nein**

Wenn du „ja” gewählt hast, gehe zu Frage 26B.

26 B. Wie findest du es, dass die Krankheit dich bei deinen körperlichen Aktivitäten (wie zum Beispiel beim Sport in der Schule, beim Laufen, Gehen oder beim Spielen eines Lieblingsspiels draussen) beeinträchtigt? Bitte markiere das Gesicht, das zeigt, wie du dich fühlst.

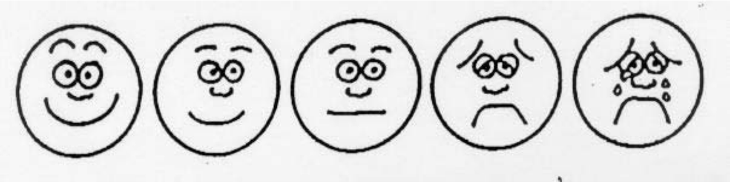

Copyright © 2007 Lakshmi Nandini Moorthy. Alle Rechte vorbehalten.

ΑΠΟΤΙΜΗΣΗ ΜΕ ΑΠΛΟ ΤΡΟΠΟ ΤΗΣ ΕΠΙΔΡΑΣΗΣ ΤΗΣ ΑΣΘΕΝΕΙΑΣ ΣΕ ΝΕΑΡΑ ΑΤΟΜΑ (SMILY-Illness) – Αξιολόγηση του Γονέα – Parent Version

Greek

Αριθμός Μελέτης __ Ημερομηνία __/__/20__

Στο ερωτηματολόγιο αυτό έχουμε χρησιμοποιήσει τα παρακάτω 5 προσωπάκια (φατσούλες) από την Κλίμακα Εκτίμησης του Πόνου με βάση την έκφραση του προσώπου των Wong-Baker (FACES Pain Rating Scale), η οποία τροποποιήθηκε με άδεια του D.L. Wong. Τα 5 αυτά προσωπάκια θα εμφανίζονται μετά από κάθε ερώτηση.

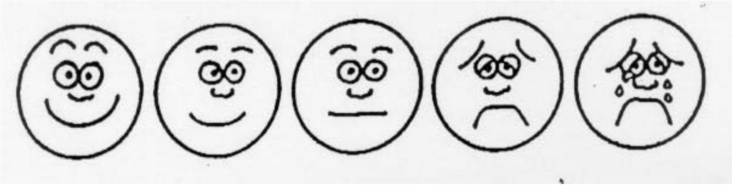

Από το Βιβλίο: Βασική Παιδιατρική Νοσηλευτική του Wong, με συγγραφείς τους Wong DL, Hockenberry-Eaton M, Wilson D, Winklestein ML και Schwartz P. 6η έκδοση: Louis, έτος 2001, εκδοτικός οίκος Mosby, σελίδα 1301. Copyright Mosby. Ανατύπωση μετά από άδεια των συγγραφέων.

Το ερωτηματολόγιο SMILY-illness είναι πνευματικά κατοχυρωμένο στην L. Nandini Moorthy, MD MS, RWJMS-UMDNJ, New Brunswick, NJ. Στη φάση αυτή της μελέτης, το ερωτηματολόγιο επιτρέπεται να χρησιμοποιηθεί μόνο από την L. Nandini Moorthy, MD, MS και τους επίσημα αναγνωρισμένους συνεργάτες της.

ΕΡΩΤΗΣΕΙΣ

Στο ερωτηματολόγιο αυτό θα σας κάνουμε πολλές ερωτήσεις σχετικά με την ασθένεια του παιδιού σας και το πώς αισθάνεται σε σχέση με αυτήν. Δεν υπάρχουν λάθος απαντήσεις. Μπορείτε να μας ρωτήσετε οτιδήποτε δεν καταλαβαίνετε. Αν θέλετε να γράψετε κάποια σχόλια, παρακαλούμε να τα καταγράψετε στο πίσω μέρος των σελίδων 5 και 6.

Διάφορα πράγματα επηρεάζουν τη ζωή του παιδιού σας και το κάνουν να αισθάνεται χαρούμενο ή λυπημένο. Υπάρχουν κάποια πράγματα που νομίζει πως είναι σημαντικά για κείνο και που μπορεί να τα κάνει (ή που δεν μπορεί να τα κάνει). Όλα αυτά μαζί διαμορφώνουν την ποιότητα ζωής του παιδιού σας.

1. Ποιά είναι η ποιότητα ζωής του παιδιού σας; Παρακαλώ, κάντε έναν κύκλο γύρω από το προσωπάκι που δείχνει καλύτερα την ποιότητα ζωής του παιδιού σας.

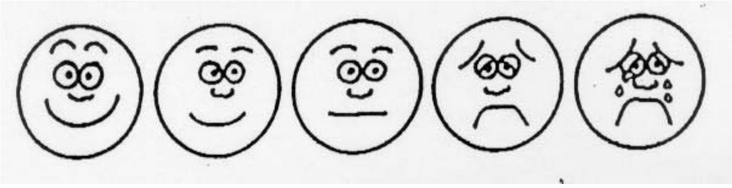

2. Πώς είναι η ασθένεια του παιδιού σας αυτήν τη στιγμή; Παρακαλώ, κάντε έναν κύκλο γύρω από το προσωπάκι που δείχνει καλύτερα πώς είναι η ασθένεια του παιδιού σας.

Copyright © 2007 Lakshmi Nandini Moorthy. Με την επιφύλαξη παντός δικαιώματος.

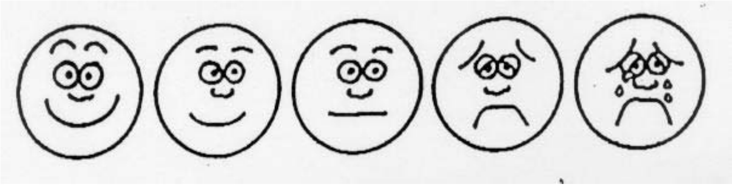

Οι ερωτήσεις 3-19 είναι σχετικές με το πώς ένοιωθε το παιδί σας τον τελευταίο μήνα εξαιτίας αυτής της ασθένειας. Παρακαλώ, κάντε έναν κύκλο γύρω από το προσωπάκι που φανερώνει καλύτερα την απάντησή σας.

3. Πώς αισθάνεται το παιδί σας λόγω της ασθένειάς του;

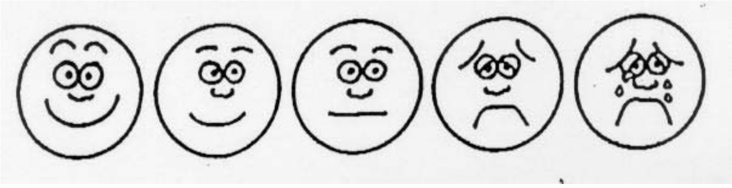

4. Το γεγονός ότι έχει αυτήν την ασθένεια, πώς κάνει το παιδί σας να αισθάνεται σε σχέση με τη φοίτησή του στο σχολείο;

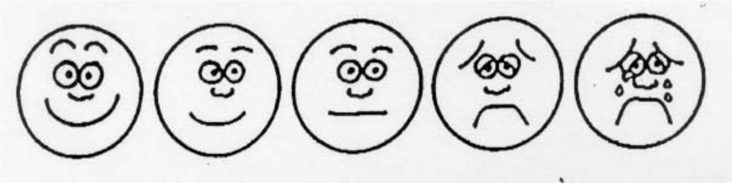

5. Το γεγονός ότι έχει αυτήν την ασθένεια, πώς κάνει το παιδί σας να αισθάνεται σε σχέση με το καθημερινό του ντύσιμο;

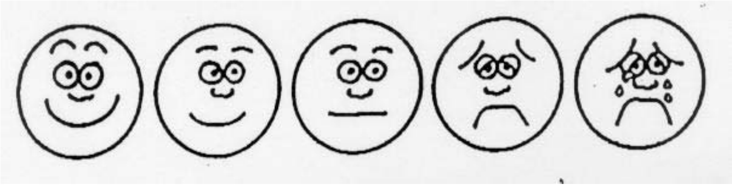

6. Το γεγονός ότι έχει αυτήν την ασθένεια, πώς κάνει το παιδί σας να αισθάνεται σε σχέση με την εμφάνισή του;

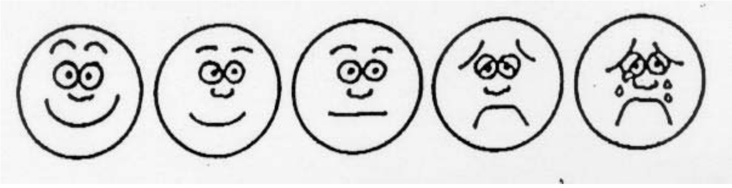

7. Το γεγονός ότι έχει αυτήν την ασθένεια, πώς κάνει το παιδί σας να αισθάνεται σε σχέση με τον εαυτό του;

Copyright © 2007 Lakshmi Nandini Moorthy. Με την επιφύλαξη παντός δικαιώματος.

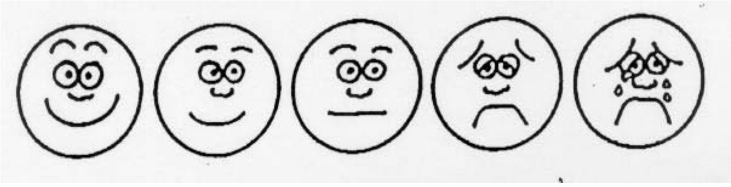

8. Πώς αισθάνεται το παιδί σας όταν χρειάζεται να μιλήσει για την ασθένειά του;

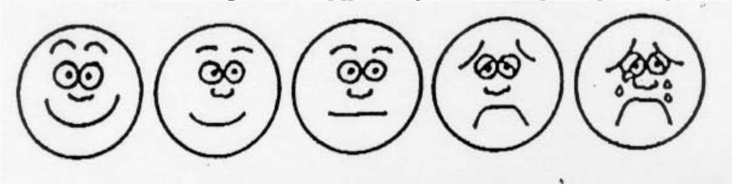

9. Πώς αισθάνεται το παιδί σας με το ότι πρέπει να πηγαίνει στο γιατρό λόγω της ασθένειάς του;

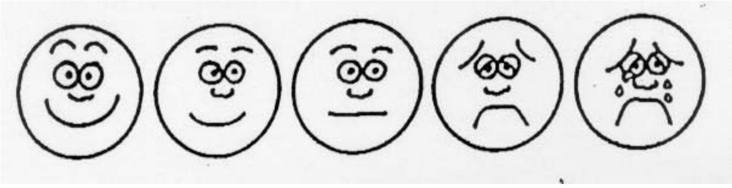

10. Πώς αισθάνεται το παιδί σας με το ότι πρέπει να πηγαίνει στο νοσοκομείο εξαιτίας της ασθένειάς του;

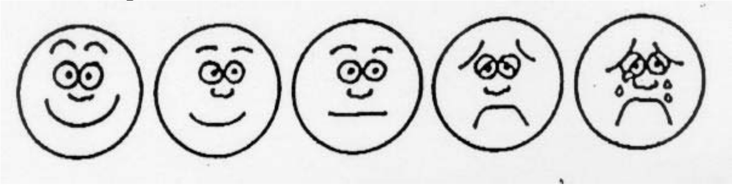

11. Πώς αισθάνεται το παιδί σας για το μέλλον του, λόγω της ασθένειάς του;

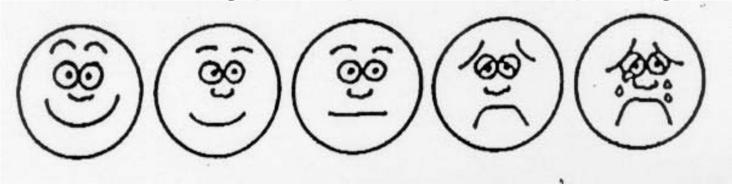

12. Πώς αισθάνεται το παιδί σας σχετικά με το ότι πρέπει να παίρνει φάρμακα για την ασθένειά του;

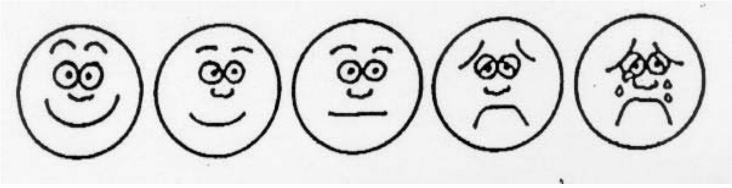

Copyright © 2007 Lakshmi Nandini Moorthy. Με την επιφύλαξη παντός δικαιώματος.

13. Πόσο ανησυχεί το παιδί σας για τις ανεπιθύμητες ενέργειες των φαρμάκων που παίρνει για την ασθένειά του;

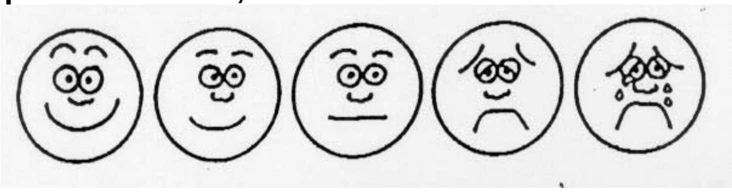

14. Πόσο ανησυχεί το παιδί σας για το αν θα χειροτερεύσει η ασθένειά του;

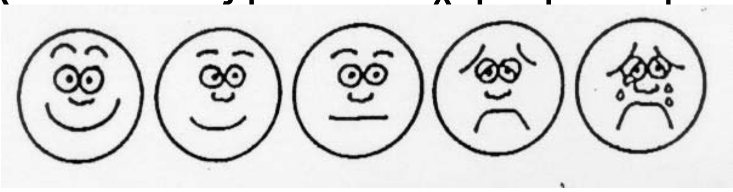

15. Πόσο μόνο αισθάνεται το παιδί σας λόγω της ασθένειάς του;

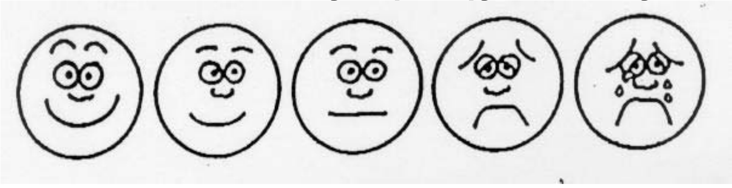

16. Το οικογενειακό περιβάλλον, πώς κάνει το παιδί σας να αισθάνεται ;

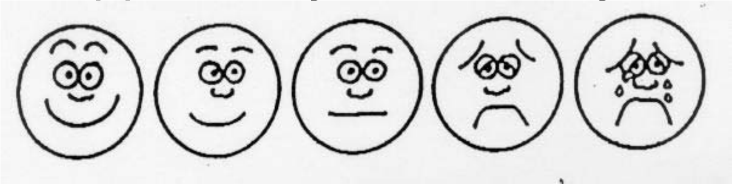

17. Πώς αισθάνεται το παιδί σας όταν πονάει εξαιτίας της ασθένειάς του;

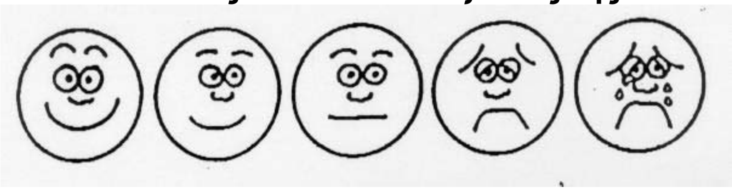

18. Πώς αισθάνεται το παιδί σας που πιθανώς δεν μπορεί να βγαίνει στον ήλιο λόγω της ασθένειάς του;

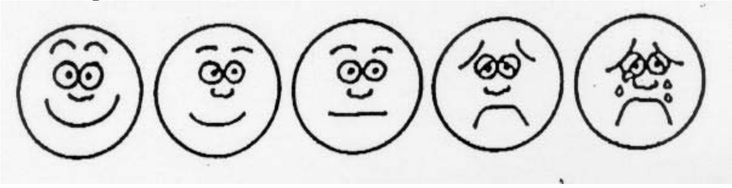

Copyright © 2007 Lakshmi Nandini Moorthy. Με την επιφύλαξη παντός δικαιώματος.

19. Πόσο κουρασμένο αισθάνεται το παιδί σας λόγω της ασθένειάς του;

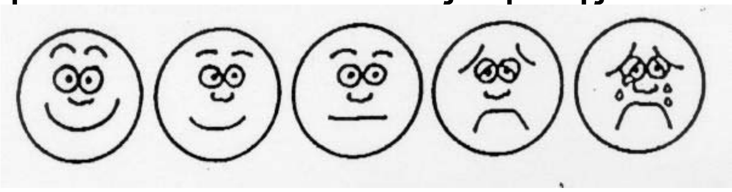

Για τις ερωτήσεις 20 Α - 26 Α, απαντήστε με "ναι" ή "όχι". Μετά, ακολουθείστε παρακαλώ τις οδηγίες.

20 Α. Οι φίλοι του παιδιού σας έχουν αλλάξει συμπεριφορά εξαιτίας της ασθένειάς του. Παρακαλώ κάντε έναν κύκλο γύρω από το "ναι" ή το "όχι". Ναι Όχι *Αν "ναι", πηγαίνετε στην ερώτηση 20 Β.*

20 Β. Το ότι το παιδί σας έχει αυτήν την ασθένεια, πώς το κάνει να αισθάνεται σχετικά με τους φίλους του επειδή έχει αυτήν την ασθένεια; Παρακαλώ**,** κάντε έναν κύκλο γύρω από το προσωπάκι που δείχνει καλύτερα το πώς αισθάνεται το παιδί σας.

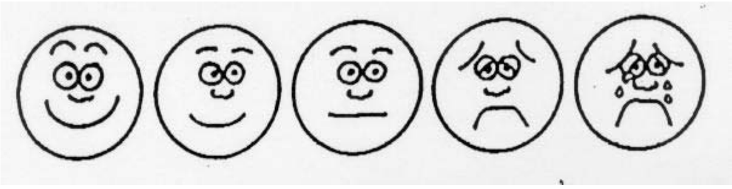

21 Α. Οι φίλοι του παιδιού σας κατανοούν το γεγονός ότι έχει μιαν ασθένεια. Ναι Όχι

Αν έχετε απαντήσει "όχι", πηγαίνετε στην ερώτηση 21 Β.

21 Β. Πώς αισθάνεται το παιδί σας που οι φίλοι του δεν κατανοούν το ότι έχει «αυτήν την ασθένεια; Παρακαλώ**,** κάντε έναν κύκλο γύρω από το προσωπάκι που δείχνει καλύτερα το πώς αισθάνεται το παιδί σας.

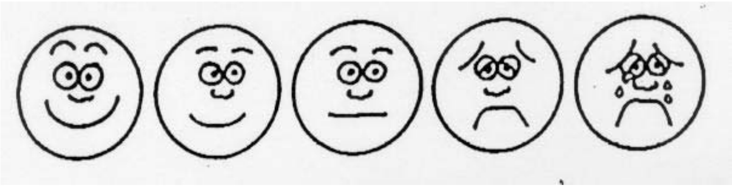

22 Α. Η ασθένειά αυτή επηρεάζει την επίδοση του παιδιού σας στο σχολείο. Ναι Όχι

Copyright © 2007 Lakshmi Nandini Moorthy. Με την επιφύλαξη παντός δικαιώματος.

Αν “ναι”, πηγαίνετε στο 2*2 B.*
22 B. Το ότι το παιδί σας έχει αυτήν την ασθένεια, πώς το κάνει να αισθάνεται σχετικά με την επίδοσή του στο σχολείο; Παρακαλώ, κάντε έναν κύκλο γύρω από το προσωπάκι που δείχνει καλύτερα το πώς αισθάνεται το παιδί σας.

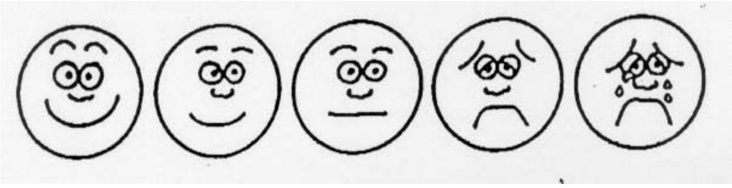

23 A. Το παιδί σας λείπει από το σχολείο (χάνει μαθήματα) εξαιτίας της ασθένειάς του.
Ναι Όχι
Αν “ναι”, πηγαίνετε στο 23 *B.*
23 B. Πώς αισθάνεται το παιδί σας που λείπει από το σχολείο εξαιτίας του αυτής της ασθένειας; Παρακαλώ, κάντε έναν κύκλο γύρω από το προσωπάκι που δείχνει καλύτερα το πώς αισθάνεται το παιδί σας.

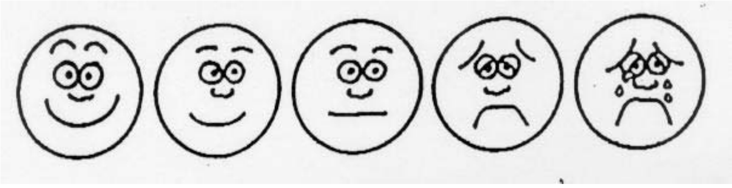

24 A. Η ασθένεια αυτή επηρεάζει τις εξωσχολικές δραστηριότητες του παιδιού σας.
Ναι Όχι

Αν απαντήσατε “ναι”, πηγαίνετε στο 24 B.
24 B. Πώς αισθάνεται το παιδί σας που ο η ασθένειά του επηρεάζει τις εξωσχολικές του δραστηριότητες; Παρακαλώ, κάντε έναν κύκλο γύρω από το προσωπάκι που δείχνει καλύτερα το πώς αισθάνεται το παιδί σας.

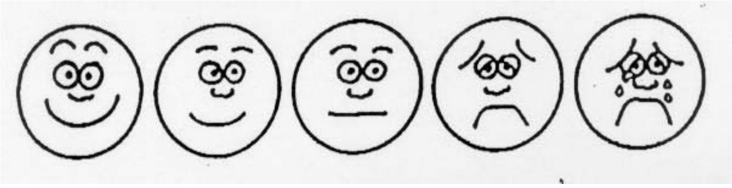

25 A. Η ασθένεια αυτή επηρεάζει το πώς διασκεδάζει το παιδί σας με τους φίλους του.

Copyright © 2007 Lakshmi Nandini Moorthy. Με την επιφύλαξη παντός δικαιώματος.

Ναι   Όχι

Αν απαντήσατε "ναι", πηγαίνετε στο 25 Β.
25 Β. Πώς αισθάνεται το παιδί σας που ο η ασθένειά του επηρεάζει το πώς διασκεδάζει με τους φίλους του; Παρακαλώ, κάντε έναν κύκλο γύρω από το προσωπάκι που δείχνει καλύτερα το πώς αισθάνεται το παιδί σας.

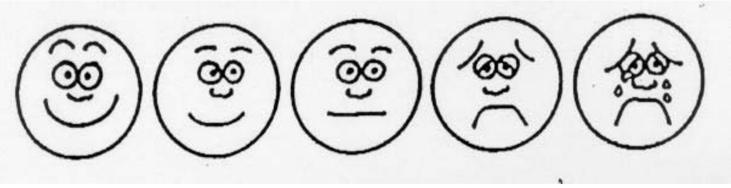

26 Α. Η ασθένεια αυτή επηρεάζει τη συμμετοχή του σε φυσικές δραστηριότητες (όπως γυμναστικές ασκήσεις στο σχολείο, τρέξιμο, περπάτημα, ένα αγαπημένο παιχνίδι ανοιχτού χώρου).   Ναι   Όχι

Αν απαντήσατε "ναι", πηγαίνετε στο 26 Β.
26 Β. Πώς αισθάνεται το παιδί σας που η ασθένεια επηρεάζει τη συμμετοχή του σε φυσικές δραστηριότητες (όπως γυμναστικές ασκήσεις στο σχολείο, τρέξιμο, περπάτημα, ένα αγαπημένο παιχνίδι ανοιχτού χώρου); Παρακαλώ, κάντε έναν κύκλο γύρω από το προσωπάκι που δείχνει καλύτερα το πώς αισθάνεται το παιδί σας.

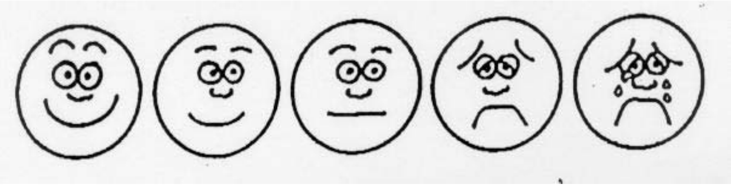

Copyright © 2007 Lakshmi Nandini Moorthy.   Με την επιφύλαξη παντός δικαιώματος.

ΑΠΟΤΙΜΗΣΗ ΜΕ ΑΠΛΟ ΤΡΟΠΟ ΤΗΣ ΕΠΙΔΡΑΣΗΣ ΤΗΣ ΑΣΘΕΝΕΙΑΣ ΣΕ ΝΕΑΡΑ ΑΤΟΜΑ (SMILY-Illness) – Αξιολόγηση του Παιδιού – Child Version

Αριθμός Μελέτης __ Ημερομηνία __/__/20__ (GREEK)

Στο ερωτηματολόγιο αυτό έχουμε χρησιμοποιήσει τα παρακάτω 5 προσωπάκια (φατσούλες) από την Κλίμακα Εκτίμησης του Πόνου με βάση την έκφραση του προσώπου των Wong-Baker (FACES Pain Rating Scale), η οποία τροποποιήθηκε με άδεια του D.L. Wong. Τα 5 αυτά προσωπάκια θα εμφανίζονται μετά από κάθε ερώτηση.

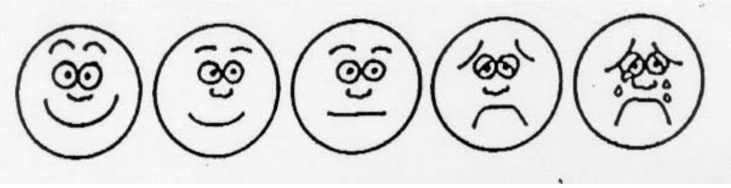

Από το Βιβλίο: Βασική Παιδιατρική Νοσηλευτική του Wong, με συγγραφείς τους Wong DL, Hockenberry-Eaton M, Wilson D, Winklestein ML και Schwartz P. 6η έκδοση: Louis, έτος 2001, εκδοτικός οίκος Mosby, σελίδα 1301. Copyright Mosby. Ανατύπωση μετά από άδεια των συγγραφέων.

Το ερωτηματολόγιο SMILY-illness είναι πνευματικά κατοχυρωμένο στην L. Nandini Moorthy, MD MS, RWJMS-UMDNJ, New Brunswick, NJ. Στη φάση αυτή της μελέτης, το ερωτηματολόγιο επιτρέπεται να χρησιμοποιηθεί μόνο από την L. Nandini Moorthy, MD, MS και τους επίσημα αναγνωρισμένους συνεργάτες της.

ΕΡΩΤΗΣΕΙΣ

Στο ερωτηματολόγιο αυτό θα σου κάνουμε πολλές ερωτήσεις σχετικά με την ασθένειά σου και το πώς αισθάνεσαι σε σχέση με αυτήν. Δεν υπάρχουν λάθος απαντήσεις. Μπορείς να μας ρωτήσεις οτιδήποτε δεν καταλαβαίνεις. Αν θέλεις να γράψεις κάποια σχόλια, παρακαλούμε να τα καταγράψεις στο πίσω μέρος των σελίδων 5 και 6.

Διάφορα πράγματα επηρεάζουν τη ζωή σου και σε κάνουν να αισθάνεσαι χαρούμενο/η ή λυπημένο/η. Υπάρχουν κάποια πράγματα που νομίζεις πως είναι σημαντικά για σένα και που μπορείς να τα κάνεις (ή που δεν μπορείς να τα κάνεις). Όλα αυτά μαζί διαμορφώνουν την ποιότητα ζωής σου.

1. **Ποιά είναι η ποιότητα ζωής σου; Παρακαλώ, κάνε έναν κύκλο γύρω από το προσωπάκι που δείχνει καλύτερα την ποιότητα ζωής σου.**

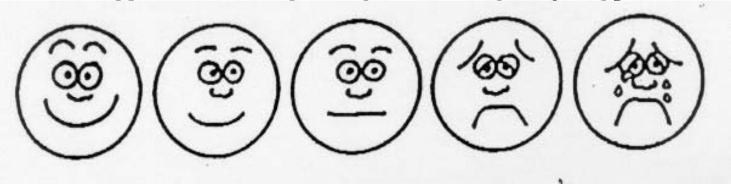

2. **Πώς είναι η ασθένειά σου αυτή τη στιγμή; Παρακαλώ, κάνε έναν κύκλο γύρω από το προσωπάκι που δείχνει καλύτερα πώς είναι η ασθένειά σου.**

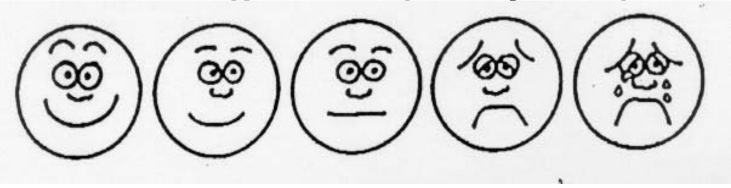

Copyright © 2007 Lakshmi Nandini Moorthy. Με την επιφύλαξη παντός δικαιώματος.

Οι ερωτήσεις 3-19 είναι σχετικές με το πώς ένοιωθες τον τελευταίο μήνα εξαιτίας της ασθένειάς σου. Παρακαλώ, κάνε έναν κύκλο γύρω από το προσωπάκι που φανερώνει καλύτερα πώς αισθάνεσαι.

3. Πώς αισθάνεσαι επειδή έχεις αυτήν την ασθένεια;

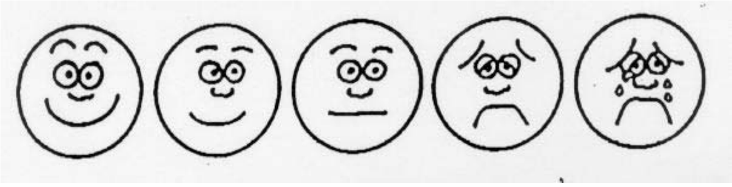

4. Το γεγονός ότι έχεις αυτήν την ασθένεια, πώς σε κάνει να αισθάνεσαι σε σχέση με τη φοίτησή σου στο σχολείο;

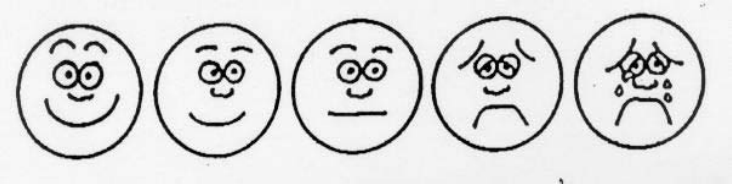

5. Το γεγονός ότι έχεις αυτήν την ασθένεια, πώς σε κάνει να αισθάνεσαι σε σχέση με το καθημερινό σου ντύσιμο;

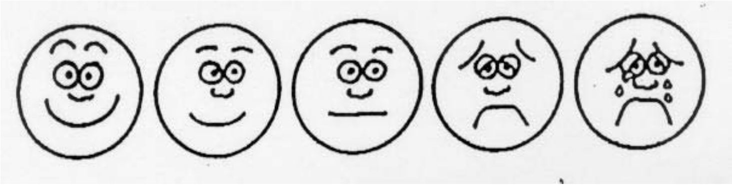

6. Το γεγονός ότι έχεις αυτήν την ασθένεια, πώς σε κάνει να αισθάνεσαι σε σχέση με την εμφάνισή σου;

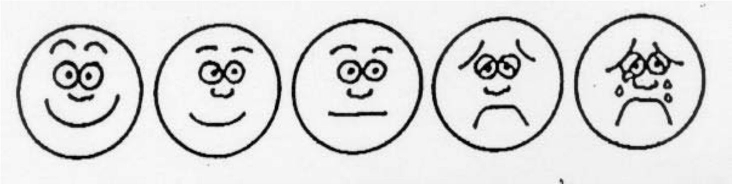

7. Το γεγονός ότι έχεις αυτήν την ασθένεια, πώς σε κάνει να αισθάνεσαι σε σχέση με τον εαυτό σου;

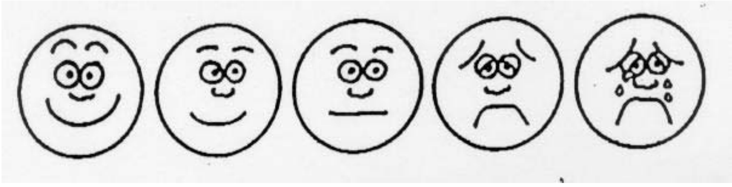

Copyright © 2007 Lakshmi Nandini Moorthy. Με την επιφύλαξη παντός δικαιώματος.

8. Πώς αισθάνεσαι όταν χρειάζεται να μιλήσεις για την ασθένειά σου;

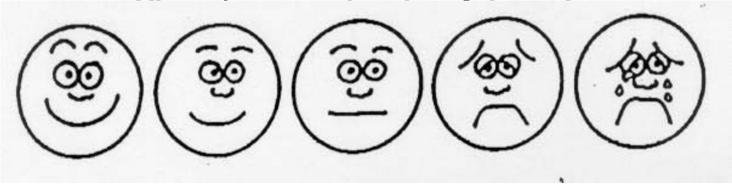

9. Πώς αισθάνεσαι με το ότι πρέπει να πηγαίνεις στο γιατρό λόγω της ασθένειάς σου;

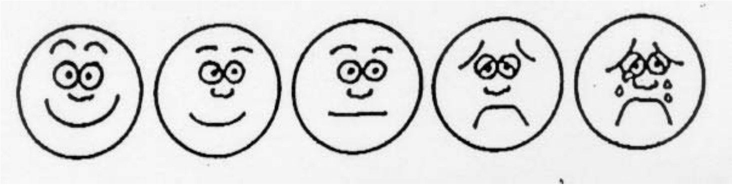

10. Πώς αισθάνεσαι με το ότι πρέπει να πηγαίνεις στο νοσοκομείο λόγω της ασθένειάς σου;

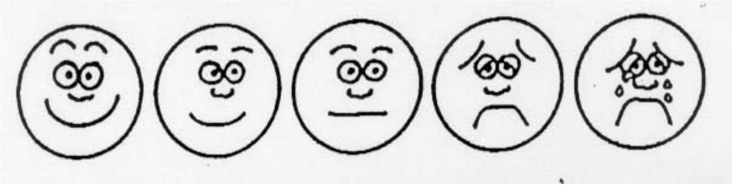

11. Πώς αισθάνεσαι λόγω του της ασθένειάς σου για το μέλλον σου;

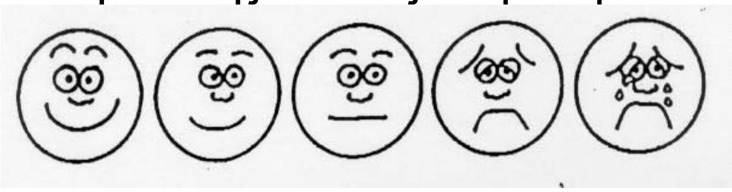

12. Πώς αισθάνεσαι σχετικά με το ότι πρέπει να παίρνεις φάρμακα για την ασθένειά σου;

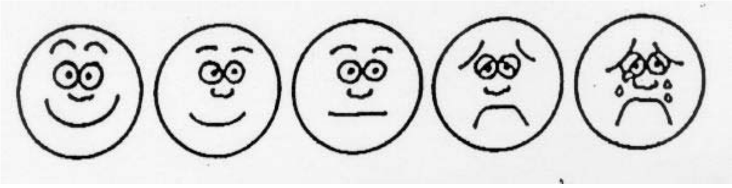

Copyright © 2007 Lakshmi Nandini Moorthy. Με την επιφύλαξη παντός δικαιώματος.

13. **Πόσο ανησυχείς για τις ανεπιθύμητες ενέργειες των φαρμάκων που παίρνεις για την ασθένειά σου;**

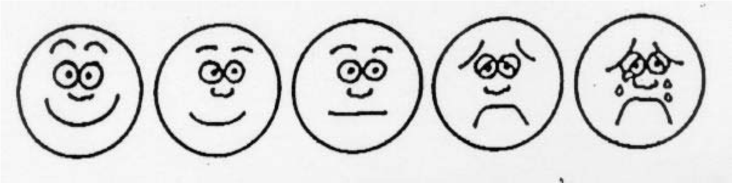

14. **Πόσο ανησυχείς για το αν θα χειροτερεύσει η ασθένειά σου;**

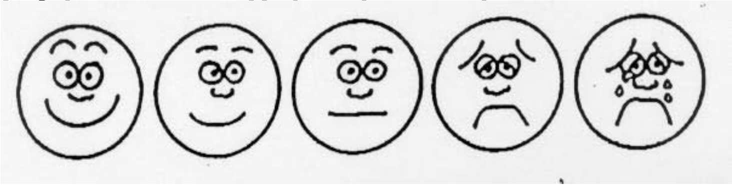

15. **Πόσο μόνος/μόνη αισθάνεσαι λόγω της ασθένειάς σου;**

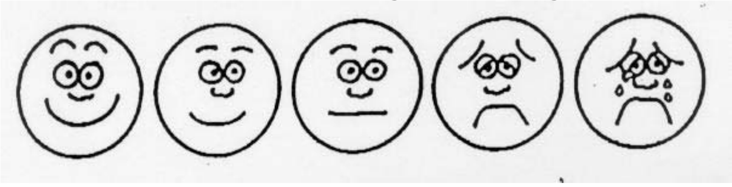

16. **Το οικογενειακό σου περιβάλλον, πώς σε κάνει να αισθάνεσαι;**

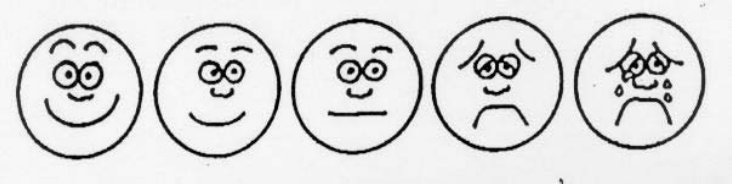

17. **Πώς αισθάνεσαι όταν πονάς εξαιτίας της ασθένειάς σου;**

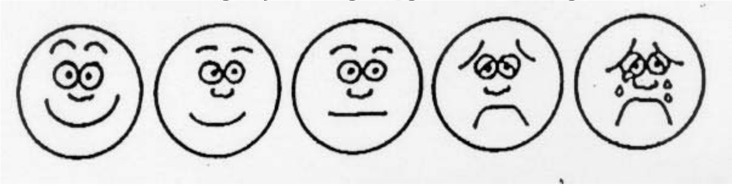

18. **Πώς αισθάνεσαι που πιθανώς δεν μπορείς να βγαίνεις στον ήλιο λόγω της ασθένειάς σου;**

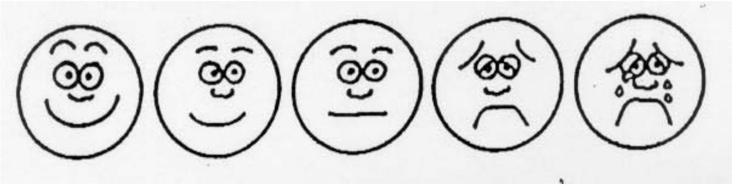

Copyright © 2007 Lakshmi Nandini Moorthy. Με την επιφύλαξη παντός δικαιώματος.

19. Πόσο κουρασμένος/κουρασμένη αισθάνεσαι λόγω της ασθένειάς σου;

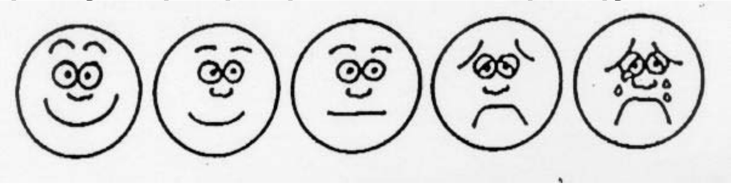

Για τις ερωτήσεις 20 Α - 26 Α, απάντησε με "ναι" ή "όχι". Μετά, ακολούθησε παρακαλώ τις οδηγίες.

20 Α. Οι φίλοι μου έχουν αλλάξει συμπεριφορά εξαιτίας της ασθένειάς μου. Παρακαλώ κάνε έναν κύκλο γύρω από το "ναι" ή το "όχι" Ναι Όχι

Αν "ναι", πηγαίνεις στην ερώτηση 20 Β.

20 Β. Το ότι έχεις αυτήν την ασθένεια, πώς σε κάνει να αισθάνεσαι σχετικά με τους φίλους σου; Παρακαλώ**,** κάνε έναν κύκλο γύρω από το προσωπάκι που δείχνει καλύτερα το πώς αισθάνεσαι.

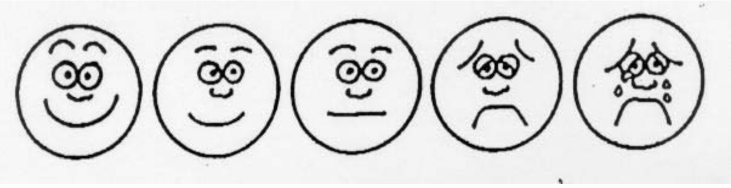

21 Α. Οι φίλοι μου κατανοούν το γεγονός ότι έχω αυτήν την ασθένεια. Ναι Όχι

Αν έχεις απαντήσει "όχι", πηγαίνεις στην ερώτηση 21 Β.

21 Β. Πώς αισθάνεσαι που οι φίλοι σου δεν κατανοούν το ότι έχεις αυτήν την ασθένεια; Παρακαλώ**,** κάνε έναν κύκλο γύρω από το προσωπάκι που δείχνει καλύτερα το πώς αισθάνεσαι.

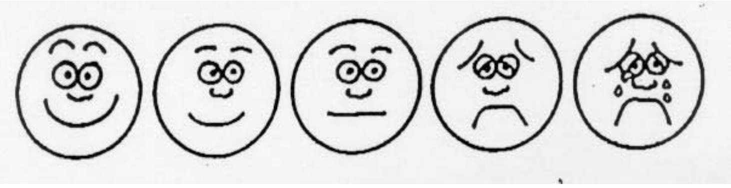

22 Α. Η ασθένειά μου επηρεάζει την επίδοσή μου στο σχολείο. Ναι Όχι

Αν "ναι", πηγαίνεις στο 2*2 Β.*

22 Β. Το ότι έχεις μιαν ασθένεια, πώς σε κάνει να αισθάνεσαι σχετικά με την επίδοσή σου στο σχολείο; Παρακαλώ**,** κάνε έναν κύκλο γύρω από το προσωπάκι που δείχνει καλύτερα το πώς αισθάνεσαι.

Copyright © 2007 Lakshmi Nandini Moorthy. Με την επιφύλαξη παντός δικαιώματος.

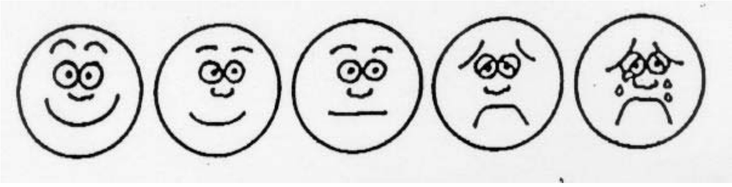

23 Α. Λείπω από το σχολείο (χάνω μαθήματα) εξαιτίας της ασθένειάς μου. Ναι Όχι

Αν "ναι", πηγαίνεις στο 23 *Β.*
23 Β. Πώς αισθάνεσαι που λείπεις από το σχολείο εξαιτίας της ασθένειάς σου; Παρακαλώ, κάνε έναν κύκλο γύρω από το προσωπάκι που δείχνει καλύτερα το πώς αισθάνεσαι.

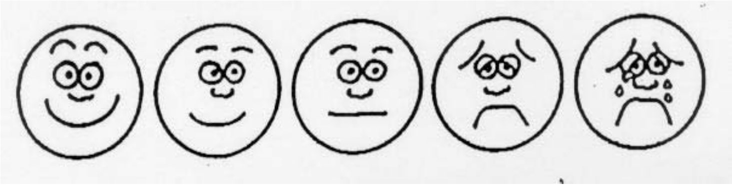

24 Α. Η ασθένειά μου επηρεάζει τις εξωσχολικές δραστηριότητές μου. Ναι Όχι

Αν απάντησες "ναι", πηγαίνεις στο 24 Β.
24 Β. Πώς αισθάνεσαι που η ασθένειά σου επηρεάζει τις εξωσχολικές σου δραστηριότητες; Παρακαλώ, κάνε έναν κύκλο γύρω από το προσωπάκι που δείχνει καλύτερα το πώς αισθάνεσαι.

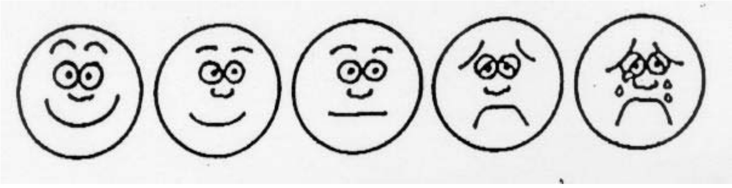

25 Α. Η ασθένειά μου επηρεάζει το πως διασκεδάζω με τους φίλους μου. Ναι Όχι

Αν απάντησες "ναι", πηγαίνεις στο 25 Β.
25 Β. Πώς αισθάνεσαι που η ασθένειά σου επηρεάζει το πως διασκεδάζεις με τους φίλους σου; Παρακαλώ, κάνε έναν κύκλο γύρω από το προσωπάκι που δείχνει καλύτερα το πώς αισθάνεσαι.

Copyright © 2007 Lakshmi Nandini Moorthy. Με την επιφύλαξη παντός δικαιώματος.

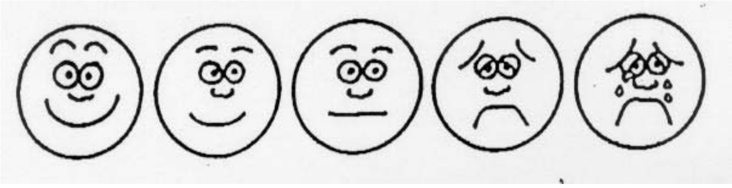

26 Α. Η ασθένειά μου επηρεάζει τη συμμετοχή μου σε φυσικές δραστηριότητες (όπως γυμναστικές ασκήσεις στο σχολείο, τρέξιμο, περπάτημα, ένα αγαπημένο παιχνίδι ανοιχτού χώρου). Ναι Όχι

Αν απάντησες "ναι", πηγαίνεις στο 26 Β.

26 Β. Πώς αισθάνεσαι που η ασθένειά σου επηρεάζει τη συμμετοχή σου σε φυσικές δραστηριότητες (όπως γυμναστικές ασκήσεις στο σχολείο, τρέξιμο, περπάτημα, ένα αγαπημένο παιχνίδι ανοιχτού χώρου); Παρακαλώ**,** κάνε έναν κύκλο γύρω από το προσωπάκι που δείχνει καλύτερα το πώς αισθάνεσαι.

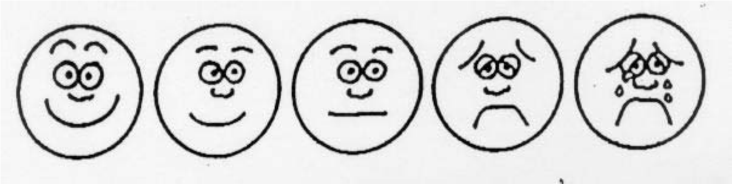

Copyright © 2007 Lakshmi Nandini Moorthy. Με την επιφύλαξη παντός δικαιώματος.

מדידה פשוטה של השפעת מחלה בצעירים
(SMILY – Illness) (סמיילי – מחלה))—דו"ח הורה

Hebrew

מחקר מס.__ תאריך___20/__/__
שנה/יום/חודש

בשאלון זה השתמשנו בחמשת הפרצופים הבאים מתוך טבלת דירוג הכאבים Wong-Baker FACES Pain Rating Scale שעובדה עם אישור מאת D.L. Wong. חמשת הפרצופים הללו יופיעו מתחת לכל שאלה.

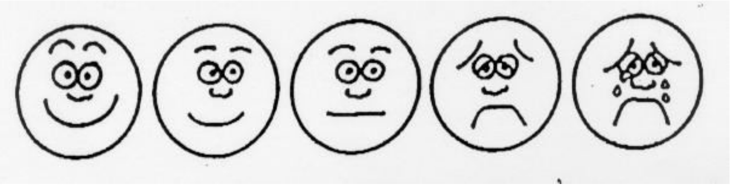

מתוך Wong DL, Hockenberry-Eaton M, Wilson D,Winklestein ML, Schwartz P.: Wong's Essentials of Pediatric Nursing, ed. 6, St. Louis, 2001, Mosby, p 1301. זכויות יוצרים של Mosby. הודפס באישור.

זכויות היוצרים של *SMILY -illness* (סמיילי-מחלה) רשומות על שם *L. Nandini Moorthy, MD MS, RWJMS-UMDNJ, New Brunswick, NJ*. שאלון זה ניתן לשימוש רק על ידי ל. נדיני מורת'י וצוות המחקר שלה.

<u>שאלות</u>

בשאלון זה נשאל אתכם שאלות רבות בנוגע למחלה של ילדכם ולרגשותיו. אין תשובות לא נכונות. אנא שאלו אותנו לגבי כל נקודה שאינכם מבינים. אם ברצונכם להוסיף הערות כלשהן, אנא עשו זאת בצידם האחורי של עמ' 5 ו-6.

ישנם דברים שונים המשפיעים על חיי ילדכם והגורמים לו להיות שמח או עצוב. ישנם דברים שילדכם חושב שהם חשובים עבורו, שהוא מסוגל לעשותם (או לא). כל הדברים השונים הללו בחיי ילדכם קובעים את איכות החיים שלו.

1. מהי איכות החיים של ילדכם? אנא הקיפו בעיגול את הפרצוף המייצג בצורה הטובה ביותר את איכות החיים של ילדכם.

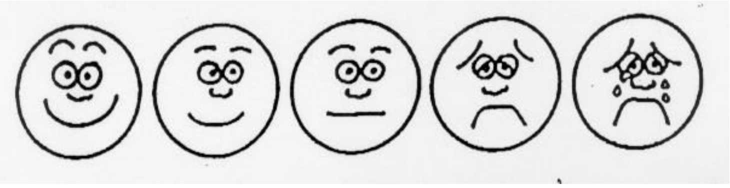

2. מה מצב המחלה של ילדכם כרגע? אנא הקיפו בעיגול את הפרצוף המייצג בצורה הטובה ביותר את מצב המחלה של ילדכם.

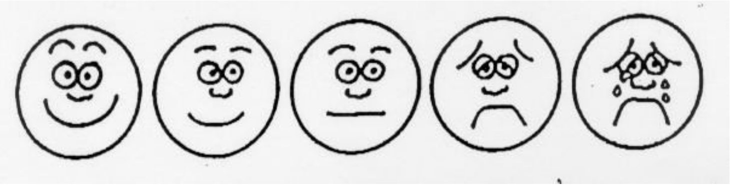

שאלות 3–19 מתייחסות להרגשתו של ילדכם בחודש האחרון עקב המחלה שלו. אנא הקיפו בעיגול את הפרצוף המייצג בצורה הטובה ביותר את תשובתכם.

זכויות יוצרים © 2007 Lakshmi Nandini Moorthy. כל הזכויות שמורות.

3. איך מרגיש ילדכם ביחס לעובדה שיש לו מחלה?

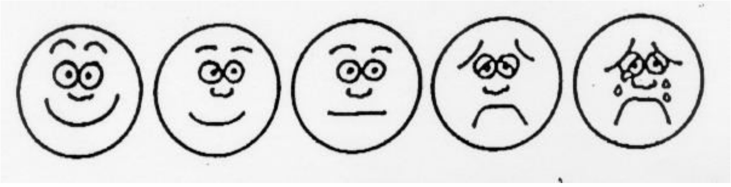

4. מה גורמת המחלה של ילדכם להרגיש בנוגע להליכה לבית הספר?

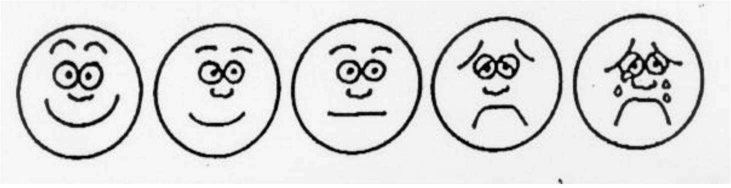

5. מה גורמת המחלה של ילדכם להרגיש בנוגע להתלבשות בכל יום?

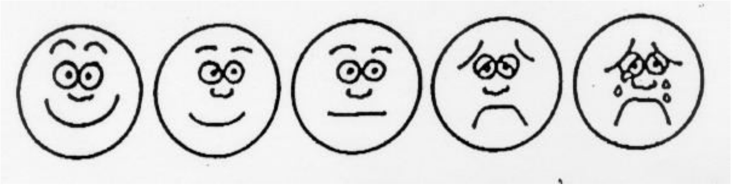

6. מה גורמת המחלה של ילדכם להרגיש בנוגע לאיך שהוא נראה?

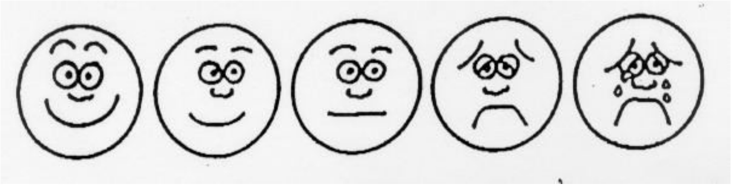

7. מה גורמת המחלה של ילדכם להרגיש ביחס לעצמו?

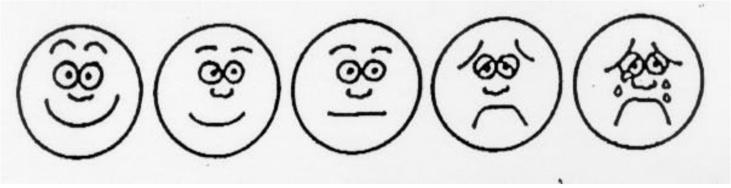

8. איך ילדך מרגיש כאשר הוא צריך לדבר על המחלה שלו?

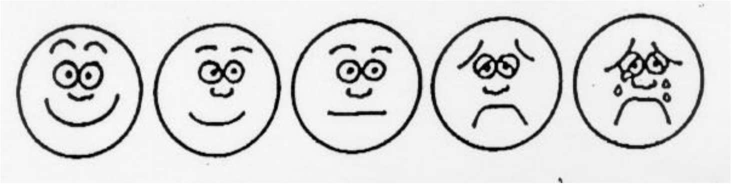

זכויות יוצרים © Lakshmi Nandini Moorthy 2007. כל הזכויות שמורות.

9. **כיצד מרגיש ילדכם ביחס להליכה לרופא עקב המחלה שלו?**

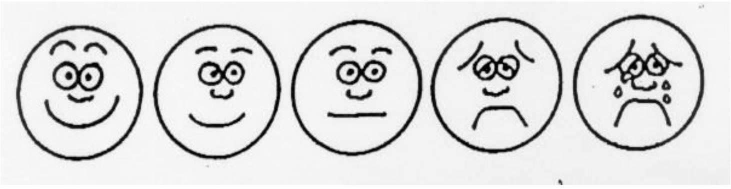

10. **כיצד מרגיש ילדכם ביחס להליכה לבית חולים עקב המחלה שלו?**

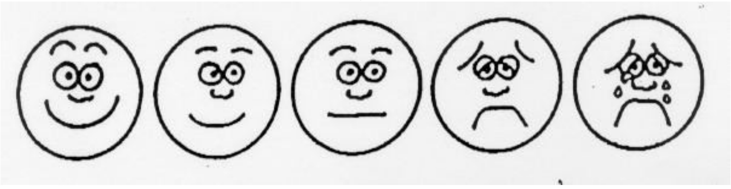

11. **מה גורמת המחלה של ילדכם להרגיש ביחס לעתידו?**

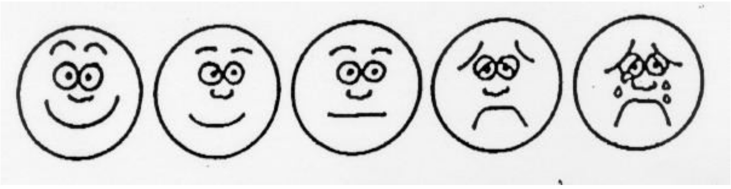

12. **מהי הרגשת ילדכם בנוגע ללקיחת תרופות לטיפול במחלה שלו?**

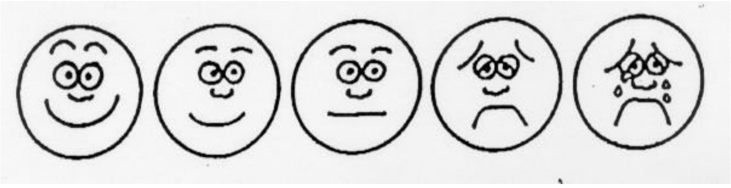

13. **עד כמה מודאג ילדכם בנוגע להשפעות הלוואי של התרופות שהוא לוקח לצורך טיפול במחלה שלו?**

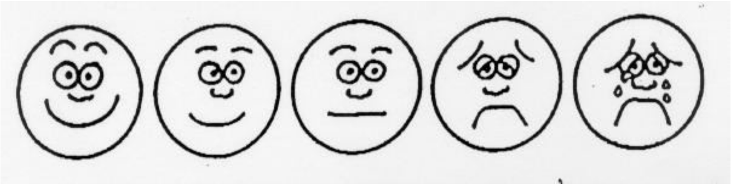

14. **עד כמה ילדכם מודאג ביחס לכך שהמחלה שלו תחמיר?**

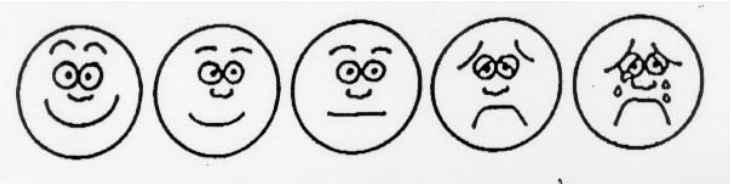

15. **עד כמה מרגיש ילדכם בודד בגלל המחלה שלו?**

זכויות יוצרים © Lakshmi Nandini Moorthy 2007. כל הזכויות שמורות.

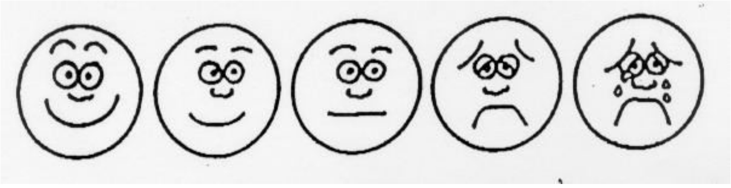

16. מה גורמת לילדכם המשפחה שלו להרגיש?

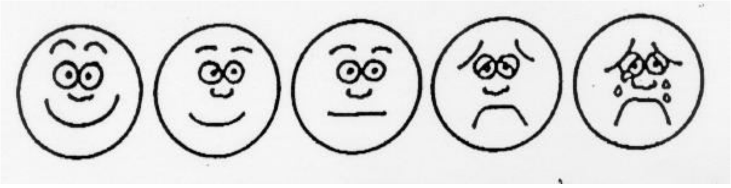

17. מה גורמים לילדכם הכאבים של המחלה שלו להרגיש?

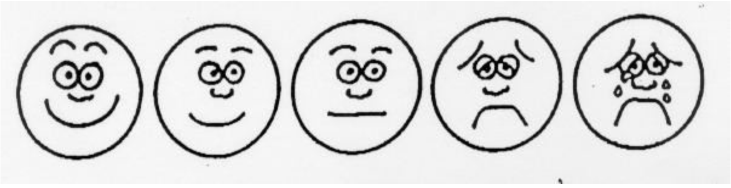

18. כיצד מרגיש ילדכם ביחס לכך שאינו מסוגל להיות בחוץ בשמש עקב המחלה שלו?

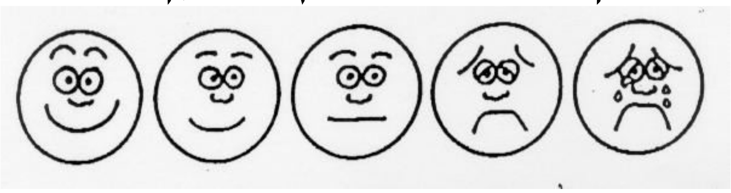

19. כמה ילדך עייף בגלל המחלה שלו?

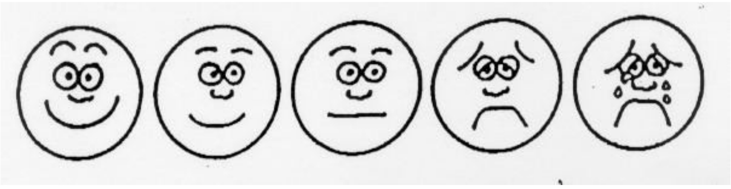

לשאלות 20א'–26א' ענו תחילה ב"כן" או "לא", ולאחר מכן המשיכו לפי ההוראות.

זכויות יוצרים © Lakshmi Nandini Moorthy 2007. כל הזכויות שמורות.

20א׳. החברים של ילדכם השתנו עקב המחלה שלו. אנא סמנו ״כן״ או ״לא״. כן לא

אם סימנתם ״כן״, עברו לשאלה 20ב׳.

20ב׳. מה גורמת המחלה של ילדכם להרגיש ביחס לחבריו?
אנא הקיפו בעיגול את הפרצוף המייצג את הרגשת ילדכם.

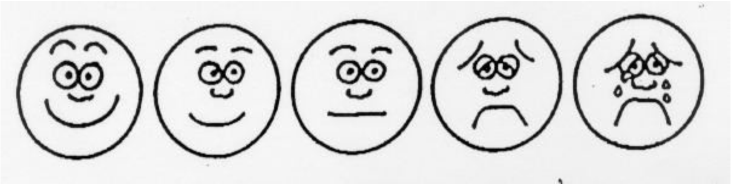

21א׳. החברים של ילדכם מבינים שיש לו מחלה. כן לא

אם סימנתם ״לא״, עברו לשאלה 21ב׳.

21ב׳. מהי הרגשת ילדכם ביחס לכך שחבריו אינם מבינים שיש לו מחלה? אנא הקיפו בעיגול את הפרצוף המייצג את הרגשת ילדכם.

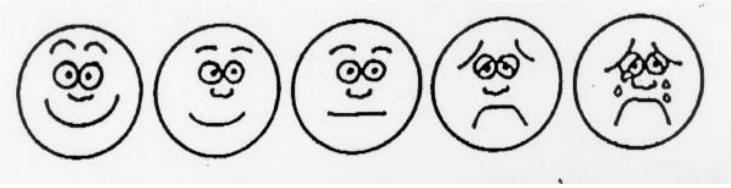

22א׳. המחלה משפיעה על הלימודים של ילדכם. כן לא

אם סימנתם ״כן״, עברו לשאלה 22ב׳.

22ב׳. מה גורמת המחלה של ילדכם להרגיש ביחס להשקעתו בלימודים?
אנא הקיפו בעיגול את הפרצוף המייצג את הרגשת ילדכם.

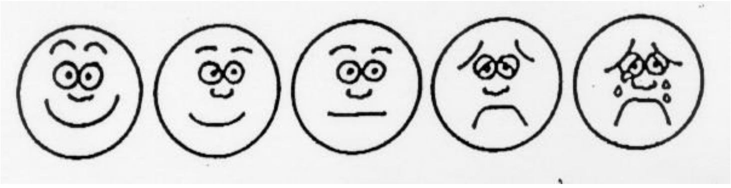

23א׳. ילדך נעדר מבית-ספר בגלל המחלה שלו. כן לא

אם סימנתם ״כן״, עברו לשאלה 23ב׳.

23ב׳. מהי הרגשת ילדכם בנוגע לכך שהוא נעדר מבית ספר בגלל המחלה שלו?
אנא הקיפו בעיגול את הפרצוף המייצג את הרגשת ילדכם.

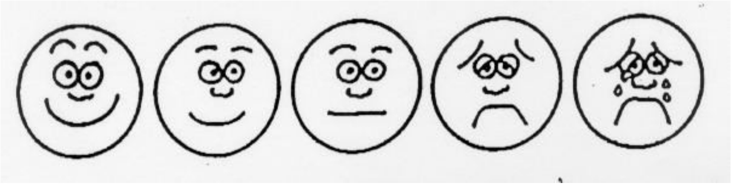

24א׳. המחלה שלו משפיעה על פעילות ילדכם מחוץ ללימודים. כן
לא

אם סימנתם ״כן״, עברו לשאלה 24ב׳.

זכויות יוצרים © Lakshmi Nandini Moorthy 2007. כל הזכויות שמורות.

24ב'. מהי הרגשת ילדכם בנוגע להשפעת המחלה שלו על פעילותו מחוץ ללימודים? אנא הקיפו בעיגול את הפרצוף המייצג את הרגשת ילדכם.

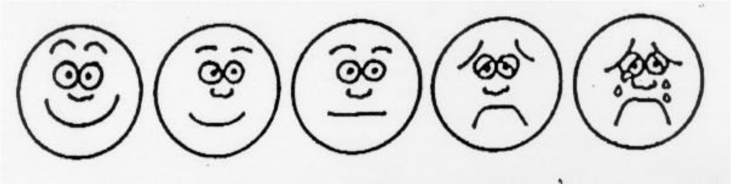

25א'. המחלה משפיעה על ההנאה שיש לילדכם עם החברים שלו. **כן** **לא**

אם סימנתם "כן", עברו לשאלה 25ב'.

25ב'. מהי הרגשת ילדכם ביחס לכך שהמחלה שלו משפיעה על ההנאה שיש לו עם החברים שלו? אנא הקיפו בעיגול את הפרצוף המייצג את הרגשת ילדכם.

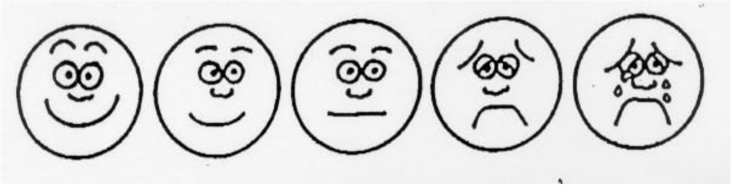

26א'. המחלה משפיעה על השתתפות ילדכם בפעילות פיזית (כגון שיעורי התעמלות בבית הספר, ריצה, הליכה, משחק אהוב מחוץ לבית).

כן **לא**

אם סימנתם "כן", עברו לשאלה 26ב'.

26ב'. מהי הרגשת ילדכם ביחס לכך שהמחלה שלו משפיעה על השתתפותו בפעילות פיזית (כגון שיעורי התעמלות בבית הספר, ריצה, הליכה, משחק אהוב מחוץ לבית)? אנא הקיפו בעיגול את הפרצוף המייצג את הרגשת ילדכם.

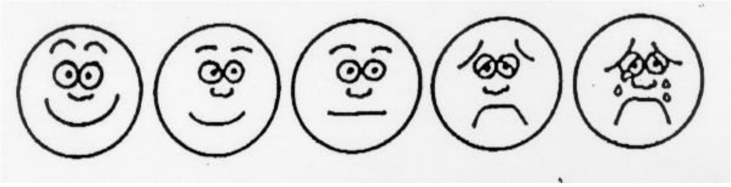

זכויות יוצרים © Lakshmi Nandini Moorthy 2007. כל הזכויות שמורות.

מדידה פשוטה של השפעת מחלה בצעירים
(SMILY – Illness (סמיילי – מחלה))—דו"ח ילד

מחקר מס.__ תאריך___20/__/__
שנה/יום/חודש

בשאלון הזה השתמשנו בחמשת הפרצופים הבאים מתוך טבלת דירוג הכאבים Wong-Baker FACES Pain Rating Scale שעובדה עם אישור מאת D.L. Wong. חמשת הפרצופים הללו יופיעו מתחת לכל שאלה.

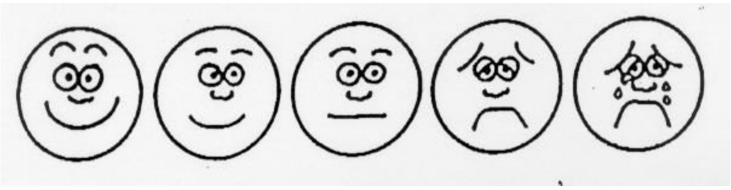

מתוך Wong DL, Hockenberry-Eaton M, Wilson D,Winklestein ML, Schwartz P.: Wong's Essentials of Pediatric Nursing, ed. 6, St. Louis, 2001, Mosby, p 1301. זכויות יוצרים של Mosby. הודפס באישור.

זכויות היוצרים של *SMILY -illness* (סמיילי-מחלה) רשומות על שם *L. Nandini Moorthy, MD MS, RWJMS-UMDNJ, New Brunswick, NJ*. שאלון זה ניתן לשימוש רק על ידי ל. ננדיני מורתי וצוות המחקר שלה.

<u>שאלות</u>

בשאלון זה נשאל שאלות רבות בנוגע למחלה שלך ולהרגשותיך. אין תשובות לא נכונות. אנא שאל אותנו לגבי כל נקודה שאינך מבין. אם ברצונך להוסיף הערות כלשהן, אנא עשה כן בצידם האחורי של עמ' 5 ו-6.

ישנם דברים שונים המשפיעים על חייך והגורמים לך להיות שמח או עצוב. ישנם דברים שאתה חושב שהם חשובים עבורך, שאתה מסוגלים לעשותם (או לא). כל הדברים השונים הללו בחייך קובעים את איכות החיים שלך.

1. מהי איכות חייך? אנא הקף בעיגול את הפרצוף המייצג בצורה הטובה ביותר את איכות החיים שלך.

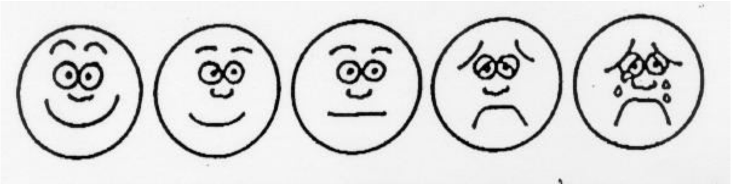

2. מה מצב המחלה שלך כרגע? אנא הקף בעיגול את הפרצוף המייצג בצורה הטובה ביותר את מצב המחלה שלך.

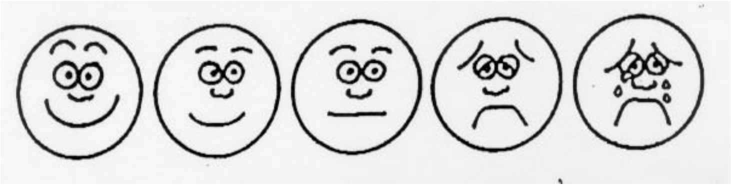

שאלות 3–19 מתייחסות להרגשתך בחודש האחרון עקב המחלה שלך. אנא הקף בעיגול את הפרצוף המייצג בצורה הטובה ביותר את הרגשתך.

זכויות יוצרים © Lakshmi Nandini Moorthy 2007. כל הזכויות שמורות.

3. מה אתה מרגיש ביחס לעובדה שיש לך מחלה?

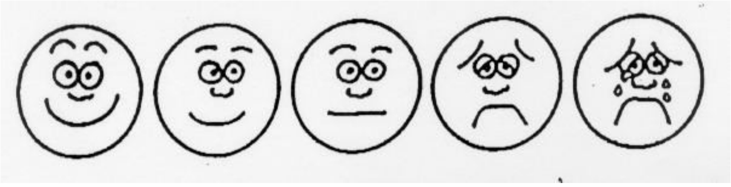

4. מה גורמת לך המחלה להרגיש בנוגע להליכה לבית הספר?

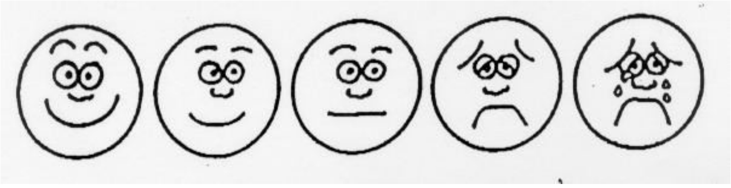

5. מה גורמת לך המחלה להרגיש בנוגע להתלבשות בכל יום?

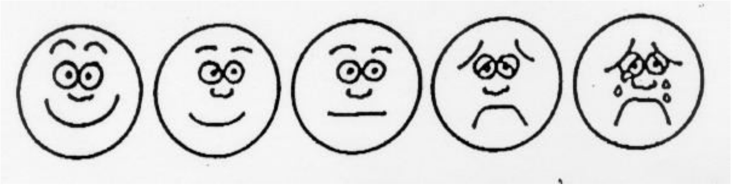

6. מה גורמת לך המחלה להרגיש בנוגע לאיך שאתה נראה?

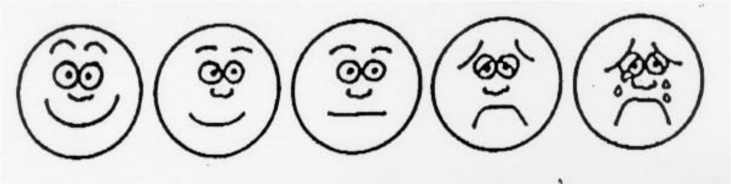

7. מה גורמת לך המחלה להרגיש ביחס לעצמך?

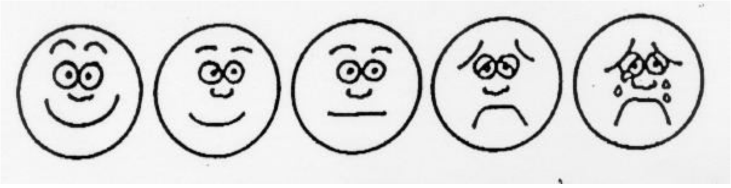

8. כיצד אתה מרגיש כשאתה צריך לדבר על המחלה שלך?

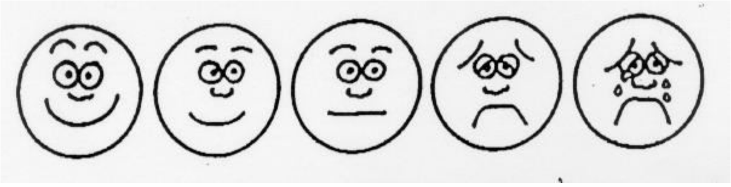

זכויות יוצרים © Lakshmi Nandini Moorthy 2007. כל הזכויות שמורות.

9. כיצד אתה מרגיש ביחס להליכה לרופא עקב המחלה?

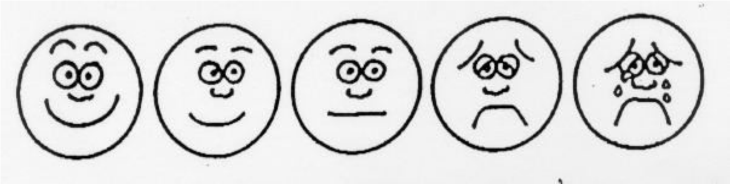

10. כיצד אתה מרגיש ביחס להליכה לבית חולים עקב המחלה?

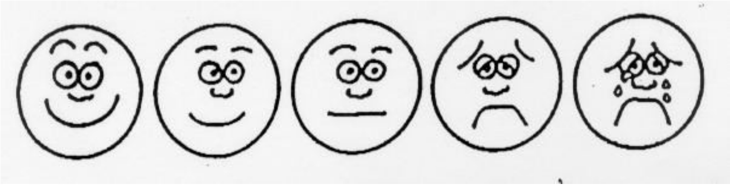

11. מה גורמת לך המחלה להרגיש ביחס לעתידך?

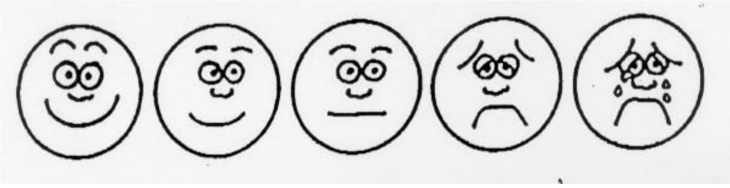

12. מה הרגשתך בנוגע ללקיחת תרופות לטיפול במחלה שלך?

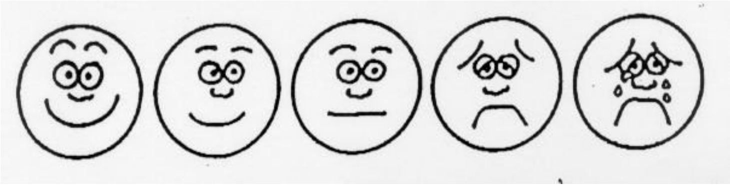

13. עד כמה אתה מודאג בנוגע להשפעות הלוואי של התרופות שאתה לוקח לצורך טיפול במחלה שלך?

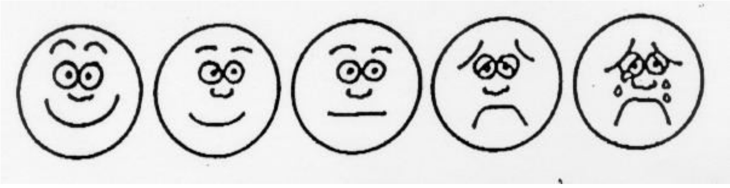

14. עד כמה אתה מודאג ביחס לכך שהמחלה שלך תחמיר?

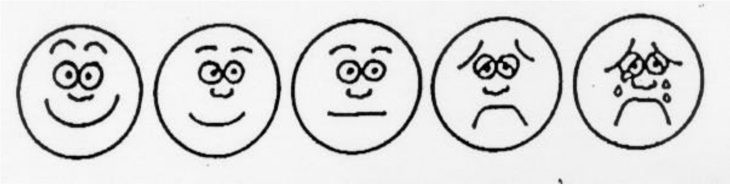

זכויות יוצרים © 2007 Lakshmi Nandini Moorthy. כל הזכויות שמורות.

.15 עד כמה אתה מרגיש בודד בגלל המחלה שלך?

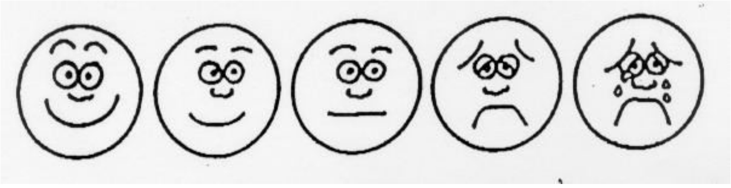

.16 מה גורמת לך המשפחה שלך להרגיש?

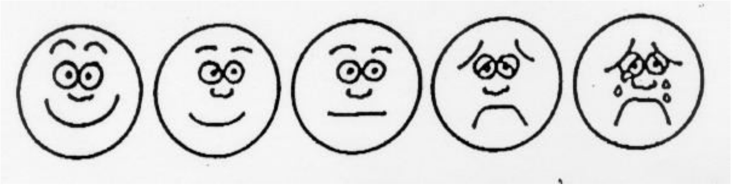

.17 מה גורמים לך הכאבים של המחלה שלך להרגיש?

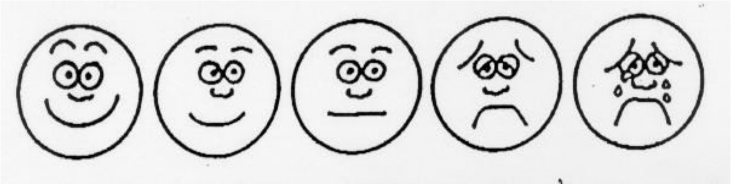

.18 כיצד אתה מרגיש ביחס לכך שאינך מסוגל להיות בחוץ בשמש עקב המחלה שלך?

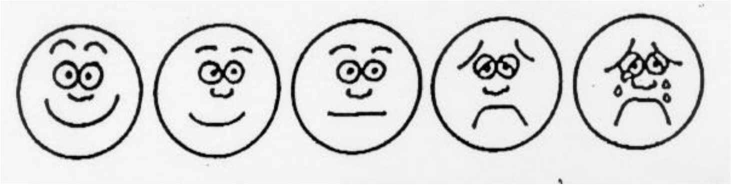

.19 כמה עייפות גורמת לך המחלה שלך להרגיש?

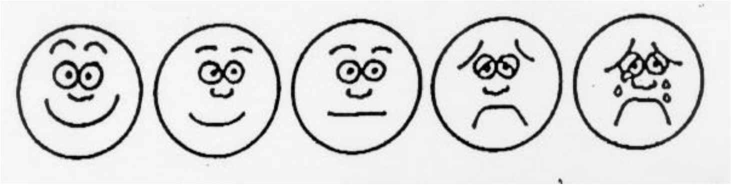

זכויות יוצרים © Lakshmi Nandini Moorthy 2007. כל הזכויות שמורות.

לשאלות 20א׳–26א׳ ענה תחילה ב״כן״ או ״לא״, ולאחר מכן המשך לפי ההוראות.

20א׳. החברים שלי השתנו עקב המחלה שלי. אנא סמן ״כן״ או ״לא״. כן לא
אם סימנת ״כן״, עבור לשאלה 20ב׳.

20ב׳. מה גורמת לך **המחלה שלך** להרגיש ביחס לחבריך?
אנא הקף בעיגול את הפרצוף המייצג את הרגשתך.

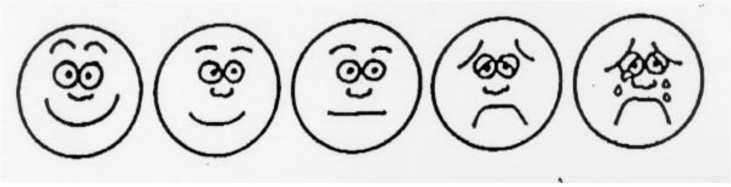

21א׳. החברים שלי מבינים שיש לי מחלה. כן לא
אם סימנת ״לא״, עבור לשאלה 21ב׳.

21ב׳. מהי הרגשתך ביחס לכך שחבריך אינם מבינים שיש לך מחלה? אנא הקף בעיגול את הפרצוף המייצג את הרגשתך.

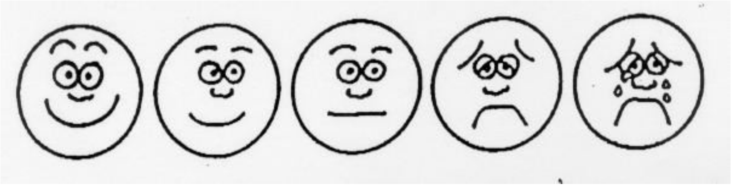

22א׳. המחלה שלי משפיעה על הלימודים שלי. כן לא
אם סימנת ״כן״, עבור לשאלה 22ב׳.

22ב׳. מה גורמת לך המחלה שלך ביחס ללימודים שלך? אנא הקף בעיגול את הפרצוף המייצג את הרגשתך.

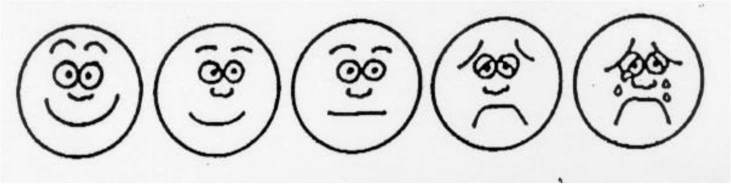

23א׳. אני נעדר מבית ספר בגלל המחלה שלי. כן לא
אם סימנת ״כן״, עבור לשאלה 23ב׳.

23ב׳. מהי הרגשתך בנוגע לכך שאתה נעדר מבית ספר בגלל המחלה שלך?
אנא הקף בעיגול את הפרצוף המייצג את הרגשתך.

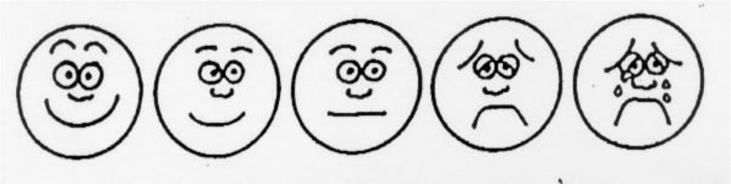

זכויות יוצרים © 2007 Lakshmi Nandini Moorthy. כל הזכויות שמורות.

24א'. המחלה שלי משפיעה על פעילותי מחוץ ללימודים. **כן** **לא**

אם סימנת "כן", עבור לשאלה 24ב'.

24ב'. מהי הרגשתך בנוגע להשפעת המחלה שלך על פעילותך מחוץ ללימודים? אנא הקף בעיגול את הפרצוף המייצג את הרגשתך.

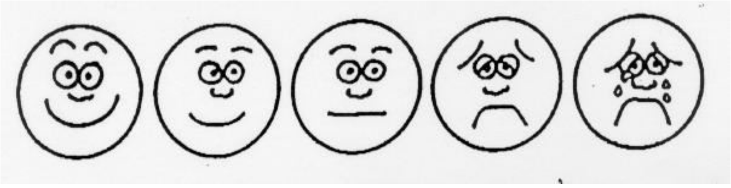

25א'. המחלה שלי משפיעה על ההנאה שיש לי עם החברים שלי. **כן** **לא**

אם סימנת "כן", עבור לשאלה 25ב'.

25ב'. מהי הרגשתך ביחס לכך שהמחלה שלך משפיעה על ההנאה שיש לך עם החברים שלך? אנא הקף בעיגול את הפרצוף המייצג את הרגשתך.

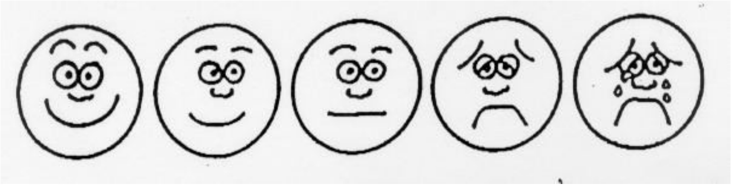

26א'. המחלה שלי משפיעה על השתתפותי בפעילויות פיזיות (כגון שיעורי התעמלות בבית הספר, ריצה, הליכה, משחק אהוב מחוץ לבית).

כן **לא**

אם סימנת "כן", עבור לשאלה 26ב'.

26ב'. מהי הרגשתך ביחס לכך שהמחלה שלך משפיעה על השתתפותך בפעילויות פיזיות (כגון שיעורי התעמלות בבית הספר, ריצה, הליכה, משחק אהוב מחוץ לבית)? אנא הקף בעיגול את הפרצוף המייצג את הרגשתך.

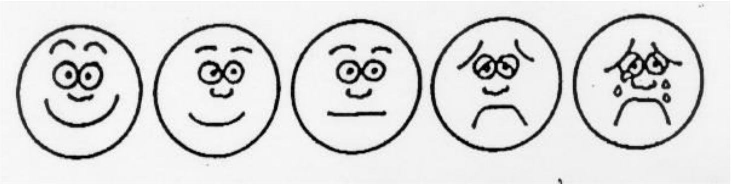

זכויות יוצרים © Lakshmi Nandini Moorthy 2007. כל הזכויות שמורות.

युवाओं में बीमारी से हुए दुष्प्रभाव का सरल उपाय
(स्माईली – बीमारी) – अभिभावक संस्करण

(Hindi)

अध्ययन संख्या .... दिनांक ..../........./20.............

इस प्रश्नावली के लिये 5 चेहरे, वॉग–बेकर के फेसिस संताप दशा स्तर से लिये गये हैं। इन चेहरों को डी0एल0 वॉग की पूर्वानुमति से रूपान्तरित किया गया हैं। ये चेहरे हर प्रश्न के नीचे दिखाई पड़ेगें।

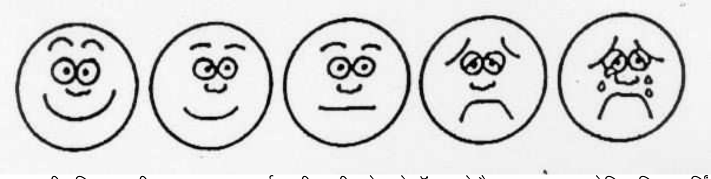

वॉग डी0एल0, हौकेनबेरी–इटन एम0, विल्सन डी, विन्कलस्टीन एम0एल0,श्वार्टस पी0 की ओर सेःवॉगस ऐसैन्शयल्स आफ पेडियाट्रिक नर्सिंग, संस्करण 6, सेंट लुइस, 2001, मौसबी, 1301 कापीराइट मौसबी। पुर्वानुमति से पुनः प्रकाशित।

दी स्माईली – बीमारी की कापीराइट एल0 नंदिनी मूर्ति एमडी, एमएस, आरडबल्यूजेएमएस– यूएनडीएनजे, – न्यू ब्रुनस्विक, न्यू जर्सी सर्वाधिकार सुरक्षित। वर्तमान काल में इस प्रश्नावली का प्रयोग केवल एल0 नंदिनी मूर्ति, एमडी एमएस और उनके द्वारा नियुक्त सहयोगियों द्वारा ही किया जाये।

सवाल

इस प्रश्नावली में हम आपसे आपके बच्चे की बीमारी एवं उसकी भावनाओं पर अनेक प्रश्न पूछेंगे। कोई गलत उत्तर नहीं हैं। कुछ न समझ आने पर, कृपया हमसे पूछें। यदि आप कोई टिप्पणी करना चाहते हैं तो पृष्ठ 5 और 6 के पीछे लिखें।

जीवन के अलग–अलग विषय आपके बच्चे के जीवन को प्रभावित करते हैं और उसे प्रसन्न अथवा उदास करते हैं। कुछ ऐसी विशेष चीजें होती है जो आपके बच्चे के लिये महत्वपूर्ण हैं जो वह कर पाता है (अथवा नहीं)। ये सारी अलग–अलग चीजें आपके बच्चे के जीवन की गुणवत्ता का स्तर बनाती हैं।

प्र0 1 आपके बच्चे के जीवन की गुणवत्ता का स्तर कैसा है? कृपया उस चेहरे पर {√} चिन्ह लगायें जो उसका जीवन स्तर दर्शाता है।

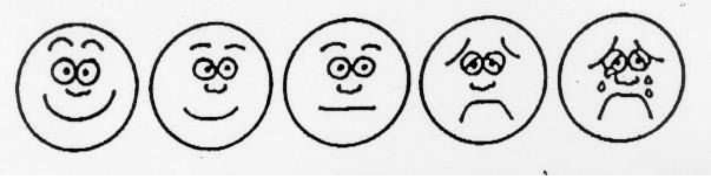

प्र0 2 आपके बच्चे की बीमारी अभी कैसी है? कृपया उस चेहरे पर {√} का चिन्ह लगायें जो यह दर्शाता हैं कि आपके बच्चे की बीमारी कैसी है।

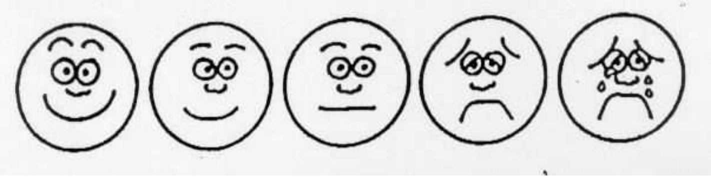

प्रश्न 3–19 इस विषय के बारे में है कि आपके बच्चे को लूपस के कारण पिछले एक महीने से कैसा महसूस हो रहा है। कृपया {√} का चिन्ह उस चेहरे पर लगायें जो आपका उत्तर दर्शाता है।

प्र0 3 बीमारी के होने से आपके बच्चे को कैसा अनुभव होता है?

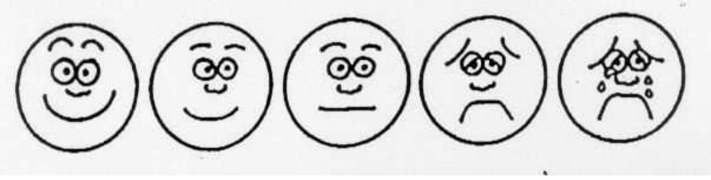

प्र0 4 बीमारी के होने से आपके बच्चे को स्कूल जाना कैसा लगता है?

कॉपीराइट 2007 लक्ष्मी नंदिनी मूर्ति. सभी अधिकार सुरक्षित.

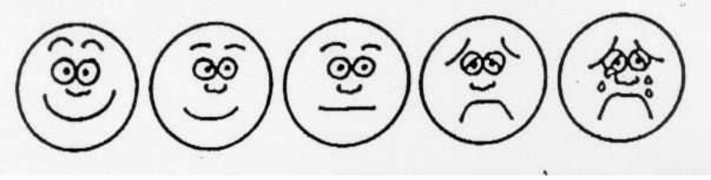

प्र0 5 बीमारी के होने से आपके बच्चे को प्रतिदिन कपडे पहनने का अनुभव कैसा लगता हैं?

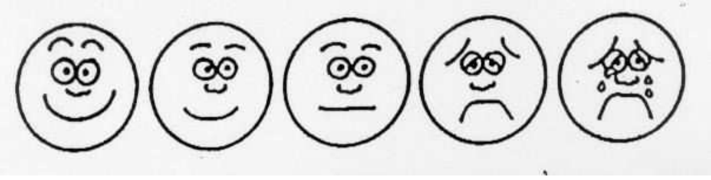

प्र0 6 बीमारी के होने से आपके बच्चे को अपने रूप के विषय में कैसा अनुभव होता है?

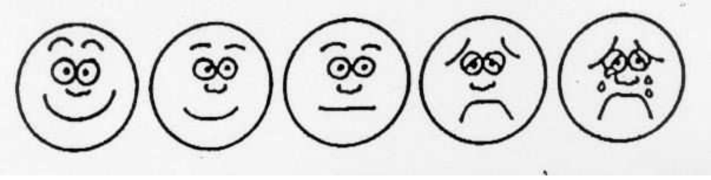

प्र0 7 आपके बच्चे को बीमारी के होने से अपने विषय में क्या अनुभव होता हैं?

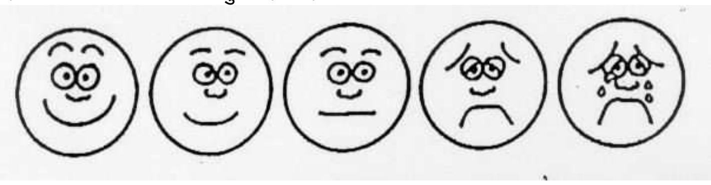

प्र0 8 आपके बच्चे को अपनी बीमारी के विषय में जब बात करनी होती है, तो उसे कैसा अनुभव होता है?

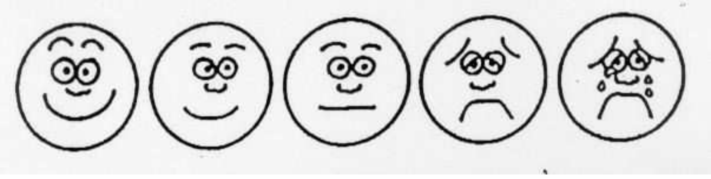

प्र0 9 आपके बच्चे को अपनी बीमारी के कारण डाक्टर के पास जाना कैसा लगता है?

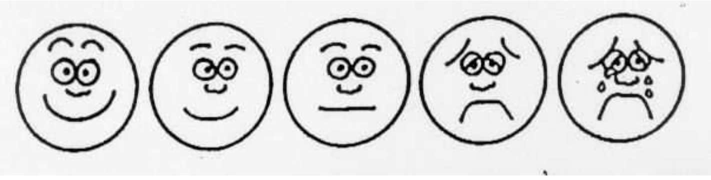

प्र0 10 आपके बच्चे को अपनी बीमारी के कारण अस्पताल जाना कैसा लगता है?

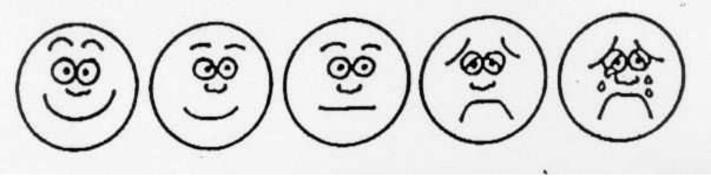

प्र0 11 आपके बच्चे को अपनी बीमारी के कारण अपने भविष्य के बारे में कैसा महसूस होता है?

कॉपीराइट 2007 लक्ष्मी नंदिनी मूर्ति. सभी अधिकार सुरक्षित.

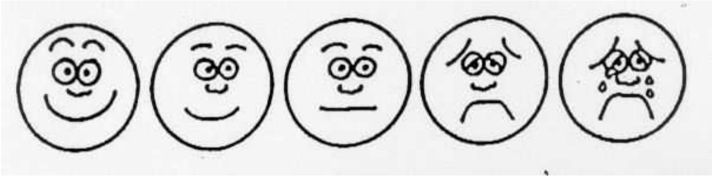

प्र0 12 आपके बच्चे को अपनी बीमारी के लिये दवाइयॉ लेना कैसा लगता है?

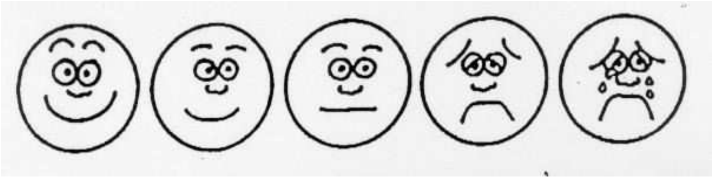

प्र0 13 बीमारी की दवाइयों के हानिकारक प्रभाव के विषय में सोचकर आपका बच्चा कितना चिंतित हैं?

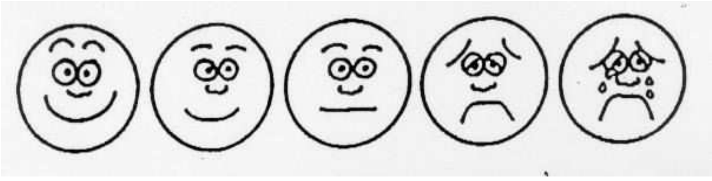

प्र0 14 आपका बच्चा अपनी बीमारी के और भी खराब हो जाने के विषय में सोंचकर कितना चिंतित है?

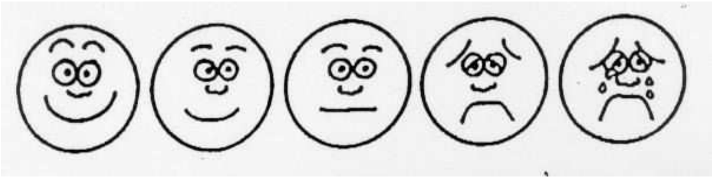

प्र0 15 अपनी बीमारी के कारण आपका बच्चा अपने आपको कितना अकेला महसूस करता हैं?

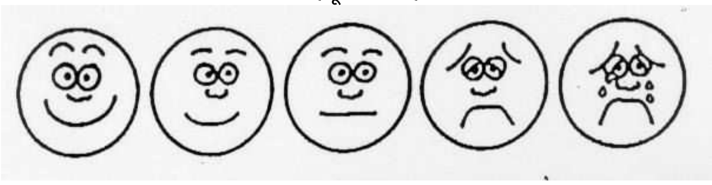

प्र0 16 आपके बच्चे का परिवार उसे कैसा महसूस करवाते हैं?

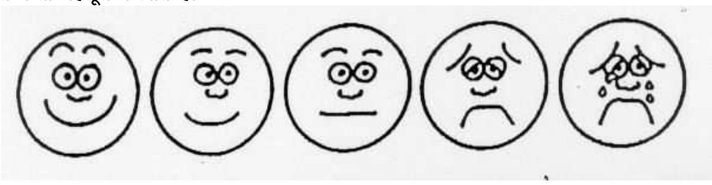

प्र0 17 आपके बच्चे पर बीमारी के दर्द का क्या असर होता हैं?

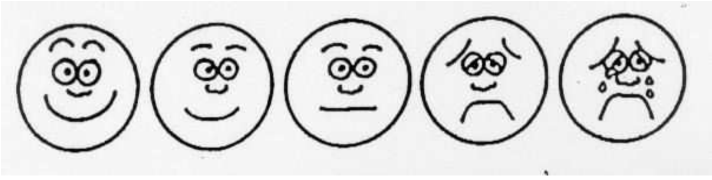

कॉपीराइट 2007 लक्ष्मी नंदिनी मूर्ति. सभी अधिकार सुरक्षित.

प्र0 18 बीमारी होने से बाहर धूप में न जा पाने के कारण आपके बच्चे को कैसा महसूस होता है?

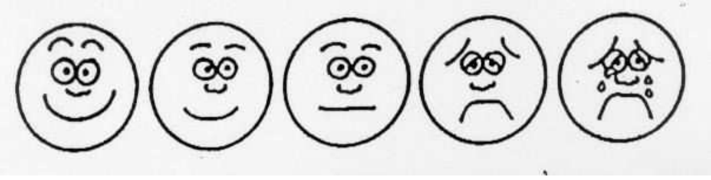

प्र0 19 बीमारी आपके बच्चे को कितना थका देता हैं?

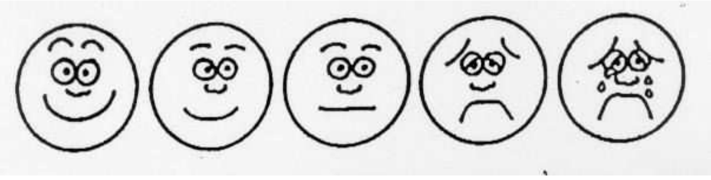

प्रश्न 20क व 26क के उत्तर 'हॉ' या 'नहीं' में दें। फिर कृपया आदेश का अनुसरण करें।

प्र020क आपके बच्चे के मित्र उसकी बीमारी हो जाने के कारण बदल गये हैं। कृपया 'हॉ' या 'नहीं' पर गोल करें। हॉ नहीं
यदि उत्तर 'हॉ' है तो प्र0 20ख का उत्तर दें।

प्र0 20ख आपके बच्चे को लूपस होने के कारण उसे अपने मित्रो के बारे में कैसा लगता हैं? कृपया उस चेहरे पर घेरा बनायें जो आपके बच्चे की भावना को दर्शाता हैं।

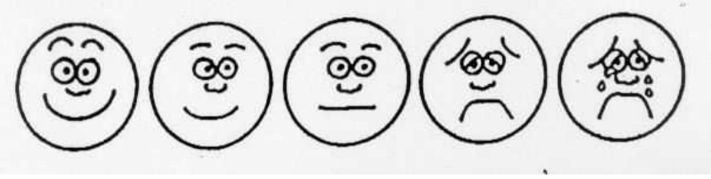

प्र021क आपके बच्चे के मित्र समझते हैं कि उसे बीमारी है। हॉ नहीं
यदि उत्तर 'नहीं' है तो प्र0 21ख पर जायें।

प्र021ख आपका बच्चा कैसा अनुभव करता है कि उसके मित्रा नहीं समझ पाते कि उसे बीमारी है? कृपया उस चेहरे पर घेरा बनायें जो आपके बच्चे की भावना को दर्शाता हैं।

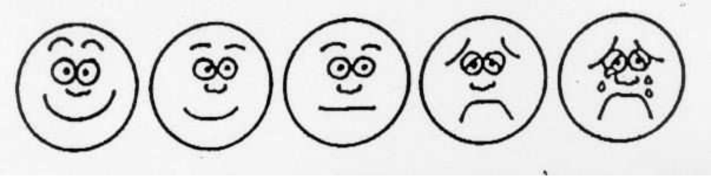

प्र022क बीमारी के कारण आपके बच्चे का स्कूल कार्य पर प्रभाव पड़ता है? हॉ नहीं
यदि उत्तर 'हॉ' है तो 22 ख को पढें।

प्र022ख बीमारी होने के कारण आपका बच्चा अपने स्कूल कार्य के विषय में कैसा महसूस करता हैं। कृपया उस चेहरे पर घेरा बनायें जो आपके बच्चे की भावना को दर्शाता है।

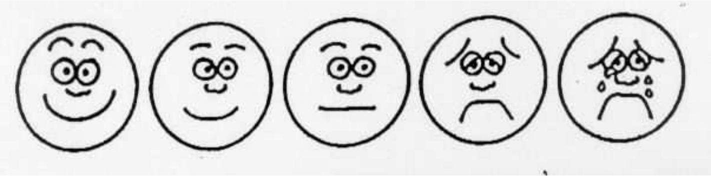

कॉपीराइट 2007 लक्ष्मी नंदिनी मूर्ति. सभी अधिकार सुरक्षित.

प्र023क आपका बच्चा अपनी बीमारी के कारण स्कूल नहीं जाता। हॉ नहीं
यदि 'हॉ' तो प्र0 23 ख पर जायें।

प्र023ख **अपनी बीमारी** के कारण आपके बच्चे को स्कूल से अनुपस्थित होना कैसा लगता है? कृपया उस चेहरे पर गोला बनायें जो आपके बच्चे की भावना को दर्शाता है।

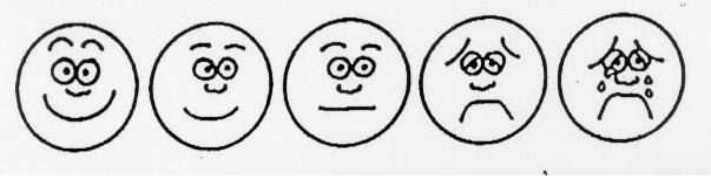

प्र024क बीमारी आपके बच्चे के स्कूल से बाहर किये गए कार्यों पर प्रभाव डालता है? हॉ नहीं
यदि आपका उत्तर 'हॉ' है तो प्र0 24 ख पर जायें

प्र024ख आपका बच्चा कैसा अनुभव करता है कि बीमारी उसके स्कूल के बाहर किये गए कार्यों को प्रभावित करता है? कृपया उस चेहरे पर घेरा बनायें जो आपकी भावना को दर्शाता है।

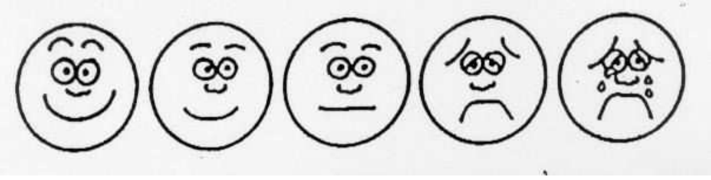

प्र025क बीमारी का प्रभाव आपके बच्चे के उस आनंद पर पड़ता है जो उसे अपने मित्रों के साथ मिलता है। हॉ नहीं
यदि आपका उत्तर 'हॉ' है तो प्र025 ख पर जायें।

प्र025ख आपके बच्चे को कैसा अनुभव होता है कि बीमारी उसे उसके मित्रों के साथ प्राप्त आनंद को प्रभावित करता है। कृपया उस चेहरे पर घेरा बनायें जो उसकी भावना को दर्शाता है।

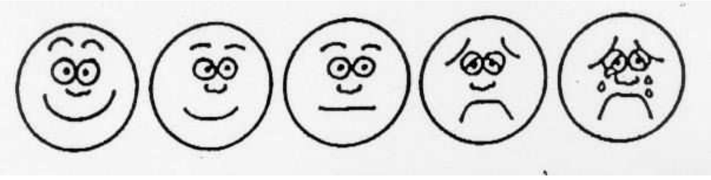

प्र026क बीमारी का प्रभाव आपके बच्चे की शारीरिक सक्रियता {जैसे कि स्कूल में जिम जाना, भागना, चलना एवं घर से बाहर पसंदीदा खेल खेलना} पर पड़ता है। हॉ नहीं
यदि आपका उत्तर 'हॉ' है तो प्र026 ख पर जायें।

प्र026ख आपका बच्चा कैसा अनुभव करता है कि बीमारी उसकी शारीरिक सक्रियता {जैसे कि स्कूल में जिम जाना, भागना, चलना एवं घर से बाहर पसंदीदा खेल खेलना} को प्रभावित करता है? कृपया उस चेहरे पर घेरा बनायें जो उसकी भावना को दर्शाता है।

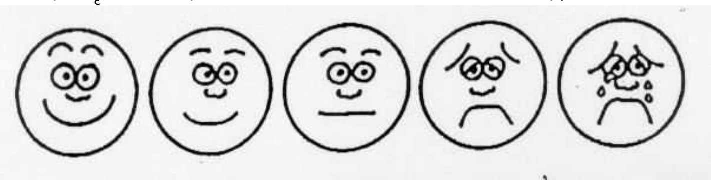

कॉपीराइट 2007 लक्ष्मी नंदिनी मूर्ति. सभी अधिकार सुरक्षित.

युवाओं में बीमारी से हुए दुष्प्रभाव का सरल उपाय
(स्माईली – बीमारी) – संतान रिपोर्ट

अध्ययन संख्या .... दिनांक ..../........./20.............

इस प्रश्नावली के लिये 5 चेहरे, वॉग–बेकर के फेसिस संताप दशा स्तर से लिये गये हैं। इन चेहरों को डी0एल0 वॉग की पूर्वानुमति से रूपान्तरित किया गया हैं। ये चेहरे हर प्रश्न के नीचे दिखाई पडेंगें।

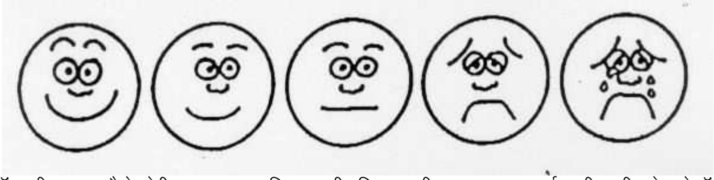

वॉग डी0एल0, हौकेनबेरी–इटन एम0, विल्सन डी, विन्कलस्टीन एम0एल0,श्वार्टस पी0 की ओर से:वॉगस ऐसैन्शयल्स आफ पेडियाट्रिक नर्सिंग, संस्करण 6, सेंट लुइस, 2001, मौसबी, 1301 कापीराइट मौसबी। पुर्वानुमति से पुनः प्रकाशित।

दी स्माईली – बीमारी की कापीराइट एल0 नंदिनी मूर्ति एमडी, एमएस, आरडबल्यूजेएमएस– यूएनडीएनजे, – न्यू ब्रुनस्विक, न्यू जर्सी सर्वाधिकार सुरक्षित। वर्तमान काल में इस प्रश्नावली का प्रयोग केवल एल0 नंदिनी मूर्ति, एमडी एमएस और उनके द्वारा नियुक्त सहयोगियों द्वारा ही किया जाये।

सवाल

इस प्रश्नावली में हम आपसे आपकी बीमारी एवं आपकी भावनाओं पर अनेक प्रश्न पूछेंगे। कोई गलत उत्तर नहीं हैं। कृपया कुछ न समझ आने पर हमसे पूछें। यदि आप कोई टिप्पणी करना चाहते हैं तो पृष्ठ 5 और 6 के पीछे लिखें।

जीवन के अलग–अलग विषय आपको प्रभावित करते हैं और आपको खुश अथवा दुखी करते हैं। कुछ ऐसी विशेष चीजें होती है जो आपके लिये महत्वपूर्ण हैं वे आप कर पाते हैं या नहीं। ये चीजें आपके जीवन में गुणवत्ता का स्तर बनाती हैं

प्र0 1 आपके जीवन में गुणवत्ता का स्तर कैसा है, कृपया जो चेहरा आपका जीवन स्तर दर्शाता है उस पर {√} का चिन्ह लगायें।

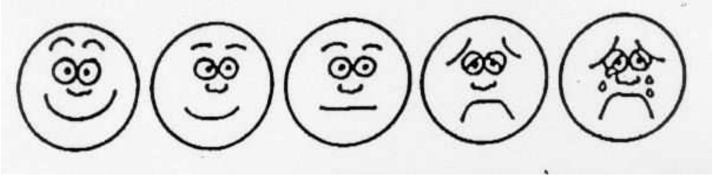

प्र0 2 अभी आपकी बीमारी कैसी है, कृपया उस चेहरे पर {√} का चिन्ह लगायें जो आपकी बीमारी की अवस्था को दर्शाता हैं।

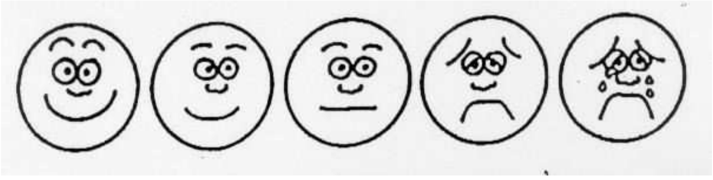

3–19 प्रश्न इस विषय के बारे में है कि आपकी बीमारी के कारण पिछले एक महीने से कैसा अनुभव कर रहे हैं। कृपया जो चेहरा आपकी भावनाओं को दर्शाता है उस पर {√} का चिन्ह लगायें।

प्र0 3 आपकी बीमारी के कारण आपको कैसा अनुभव होता है?

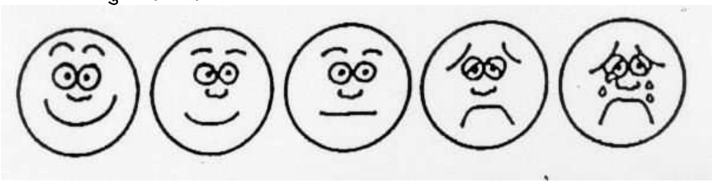

प्र0 4 आपकी बीमारी के होने से आपको स्कूल जाने के विषय में कैसा लगता है?

कॉपीराइट 2007 लक्ष्मी नंदिनी मूर्ति. सभी अधिकार सुरक्षित.

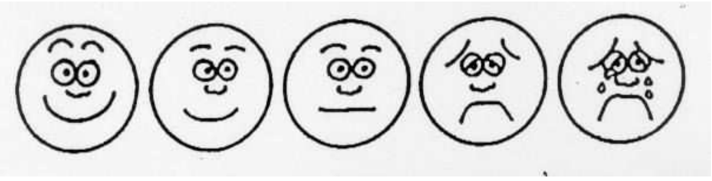

प्र0 5 आपकी बीमारी के होने से आपको प्रतिदिन अपने कपडे पहनने के विषय में कैसा लगता है?

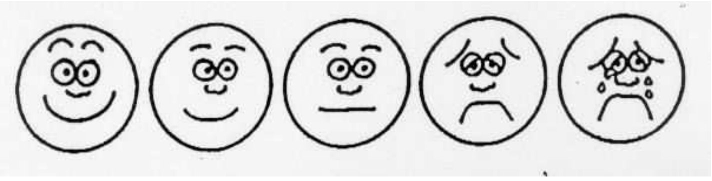

प्र0 6 बीमारी के होने से आपको इस विषय में कैसा अनुभव होता है कि आप कैसे दिखते हैं?

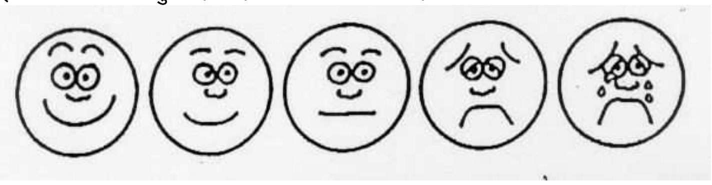

प्र0 7 बीमारी के होने से आप अपने विषय में कैसा महसूस करते हैं?

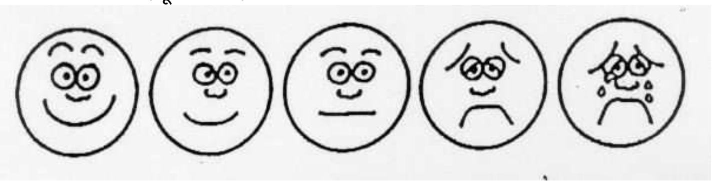

प्र0 8 आपको कैसा महसूस होता है जब आपको अपनी बीमारी के विषय में बात करनी होती है?

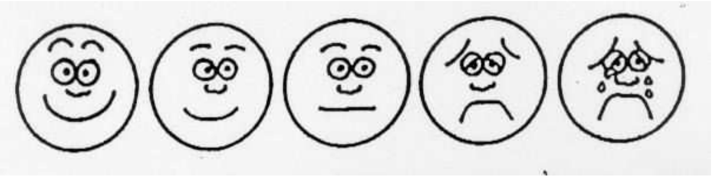

प्र0 9 अपनी बीमारी के कारण आपको डाक्टर के पास जाना कैसा लगता है?

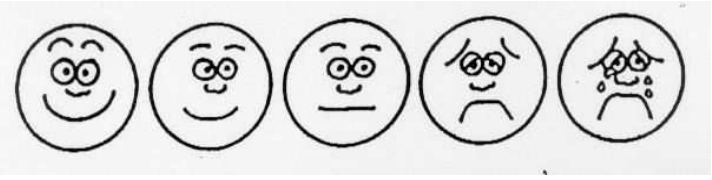

प्र0 10 अपनी बीमारी के कारण आपको अस्पताल जाना कैसा लगता है?

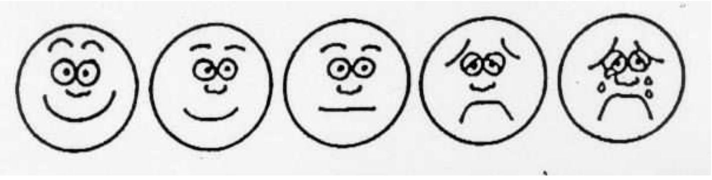

प्र0 11 अपनी बीमारी के कारण आपके अपने भविष्य के बारे में कैसा महसूस होता है?

कॉपीराइट 2007 लक्ष्मी नंदिनी मूर्ति. सभी अधिकार सुरक्षित.

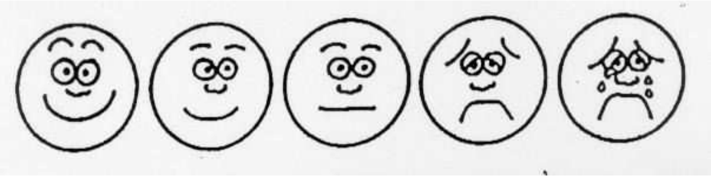

प्र0 12 आपको अपनी बीमारी के लिये दवाइयॉं लेना कैसा लगता है?

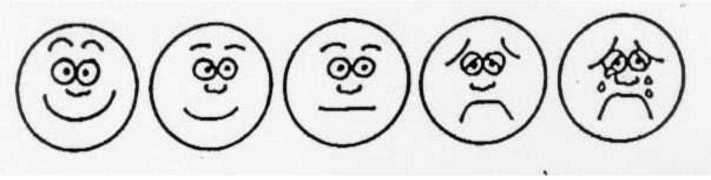

प्र0 13 अपनी बीमारी की दवाइयों के हानिकारक प्रभाव के विषय में सोच कर आप कितने चिंतित हैं?

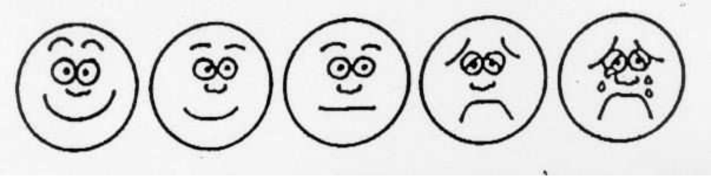

प्र0 14 बीमारी के खराब हो जाने के विषय में सोंचकर आप कितने चिंतित हैं?

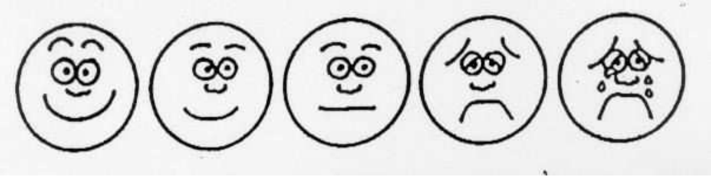

प्र0 15 अपनी बीमारी के कारण आप कितना अकेला महसूस करते हैं?

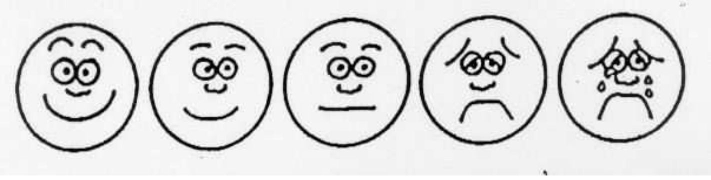

प्र0 16 आपके परिवार के साथ आपका कैसा अनुभव हैं?

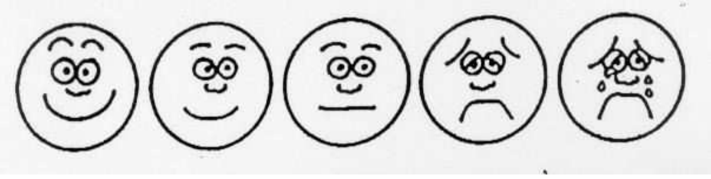

प्र0 17 बीमारी के कारण दर्द होने पर आप कैसा महसूस करते हैं?

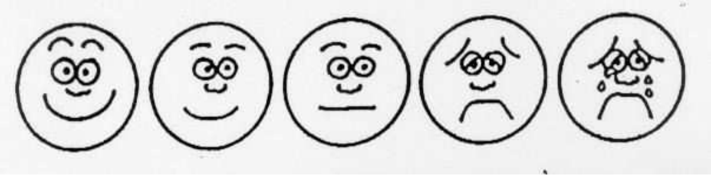

प्र0 18 बीमारी होने से आप बाहर धूप में न जा पाने के कारण कैसा अनुभव करते हैं?

कॉपीराइट 2007 लक्ष्मी नंदिनी मूर्ति. सभी अधिकार सुरक्षित.

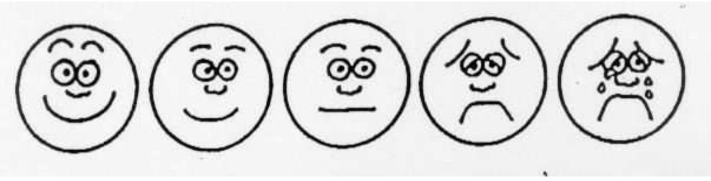

प्र0 19 अपनी बीमारी के कारण आप कितनी थकान महसूस करते हैं?

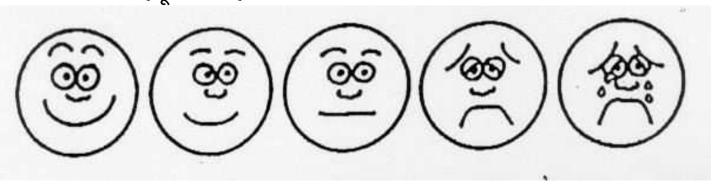

प्रश्न 20क व 26क के उत्तर हॉ या नहीं में दें फिर कृपया आदेश का अनुसरण करें।

प्र0 20क मेरे मित्र बदल गये हैं मेरी बीमारी के कारण। कृपया 'हॉ' या 'नहीं' पर गोल करें। हॉ नहीं

यदि उत्तर 'हॉ' है तो प्र0 20ख का उत्तर दें।

प्र0 20ख **बीमारी** होने के कारण आप अपने मित्रों के विषय में कैसा महसूस करते हैं? कृपया उस चेहरे पर घेरा बनायें जैसा आप अनुभव करते हैं।

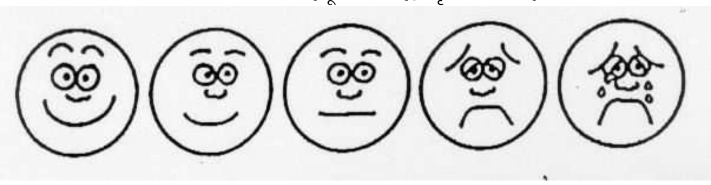

प्र021क मेरे मित्र समझते हैं कि मुझे बीमारी है हॉ नहीं

यदि उत्तर 'नही' है तो प्र0 21ख पर जायें।

प्र021ख आप कैसा अनुभव करते हैं जब आपके मित्रा नहीं समझ पाते कि आपको लूपस है। कृपया उस चेहरे पर घेरा बनायें जैसा आप अनुभव करते हैं।

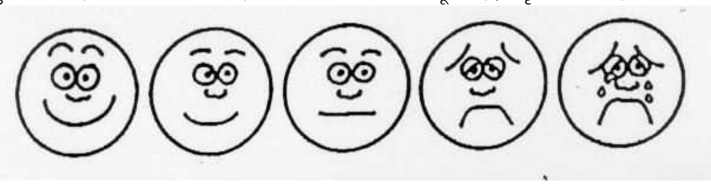

प्र022क बीमारी के कारण मेरे स्कूल कार्य पर प्रभाव पडता है। हॉ नहीं

यदि उत्तर 'हॉ' है तो 22 ख को पढे

प्र022ख आप लूपस के कारण अपने स्कूल कार्य के विषय में कैसा महसूस करते हैं। कृपया उस चेहरे पर घेरा बनायें जो आपकी भावना को दर्शाता है।

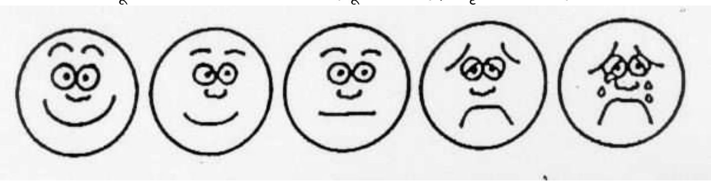

प्र023क अपनी बीमारी के कारण मैं स्कूल नहीं जाता। हॉ नहीं

यदि 'हॉ' तो प्र0 23 ख पर जायें।

प्र023ख लूपस के कारण आपको स्कूल से अनुपस्थित होना कैसा लगता है? कृपया उस चेहरे पर गोला बनायें जो आपकी भावना को दर्शाता है।

कॉपीराइट 2007 लक्ष्मी नंदिनी मूर्ति. सभी अधिकार सुरक्षित.

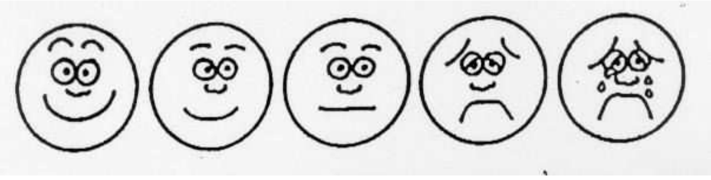

प्र024क बीमारी मेरे स्कूल से बाहर किये कार्यों को प्रभावित करता है। हॉ नहीं

यदि आपका उत्तर 'हॉ' है तो प्र0 24 ख पर जायें

प्र024ख आपको कैसा अनुभव होता है कि लूपस आपके स्कूल से बाहर किये कार्यों को प्रभावित करता है? कृपया उस चेहरे पर घेरा बनायें जो आपकी भावना को दर्शाता है।

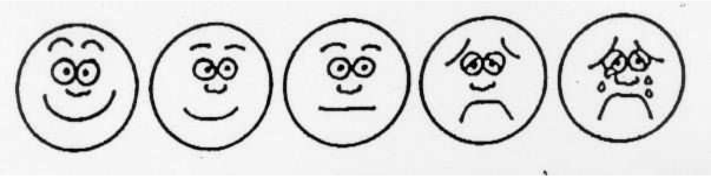

प्र025क दोस्तों के साथ प्राप्त आनंद को बीमारी प्रभावित करता है। हॉ नहीं

यदि आपका उत्तर 'हॉ' है तो प्र025 ख पर जायें।

प्र025ख आपको कैसा लगता है कि दोस्तों के साथ प्राप्त आनंद को **अपनी बीमारी** प्रभावित करता है? कृपया उस चेहरे पर घेरा बनायें जो आपकी भावना को दर्शाता है।

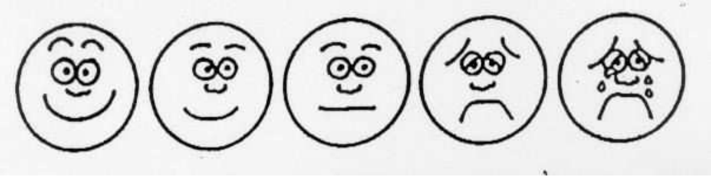

प्र026क बीमारी का प्रभाव मेरी शारीरिक सकियता {जैसे कि स्कूल में जिम जाना, भागना, चलना एवं घर से बाहर पसंदीदा खेल खेलना} पर है। हॉ नहीं

यदि आपका उत्तर 'हॉ' है तो प्र026 ख पर जायें।

प्र026ख आपको कैसा अनुभव होता है कि बीमारी का प्रभाव आपकी शारीरिक सकियता {जैसे कि स्कूल में जिम जाना, भागना, चलना एवं घर से बाहर पसंदीदा खेल खेलना} पर है? कृपया उस चेहरे पर घेरा बनायें जो दर्शाता है कि आप कैसा अनुभव करते हैं।

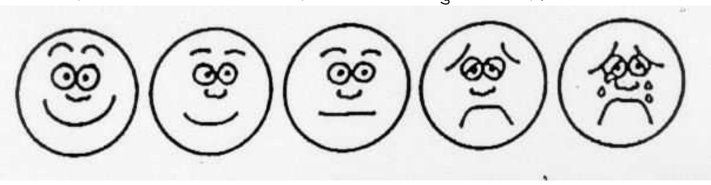

कॉपीराइट 2007 लक्ष्मी नंदिनी मूर्ति. सभी अधिकार सुरक्षित.

SIMPLE MEASURE OF IMPACT OF ILLNESS IN YOUNGSTERS (SMILY-Illness) – Szülői kérdőív

Hungarian

Vizsgálat száma __ Dátum __/__/20__

A kérdőív készítésekor az alábbi öt arcot választottuk ki a Wong-Baker fájdalomértékelő skáláról (Wong-Baker FACES Pain Rating Scale). A módosítás D.L. Wong engedélyével történt. Minden kérdés alatt ez az öt arc szerepel.

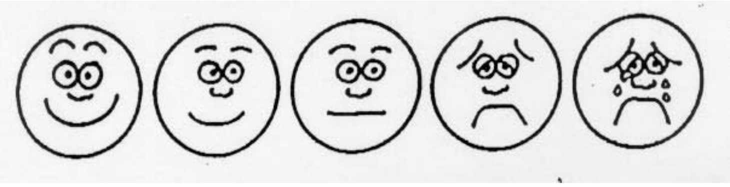

Engedéllyel átvéve: Wong DL, Hockenberry-Eaton M, Wilson D, Winklestein ML, Schwartz P.: Wong's Essentials of Pediatric Nursing, ed. 6, St. Louis, 2001, Mosby, p 1301. Copyright Mosby.

A SMILY-illness szerzői jogtulajdonosa L. Nandini Moorthy, MD MS, RWJMS-UMDNJ, New Brunswick, NJ. A kérdőívet jelenleg kizárólag L. Nandini Moorthy, MD MS és az általa kijelölt kutatók használhatják.

KÉRDÉSEK

Gyermeke betegségéről és azzal kapcsolatos érzéseiről teszünk fel kérdéseket ebben a kérdőívben. Nincsenek rossz válaszok. Kérdezzen meg bármit, amit nem ért. Amennyiben kiegészítő megjegyzéseket tenne, az 5. és a 6. oldal határa írhat.

Különféle dolgok hatnak gyermeke életére, vannak olyanok, amelyektől vidám lesz, másoktól elszomorodik. Vannak olyan dolgok, amelyek fontosak számára - ezeket vagy képes megcsinálni, vagy épp nem tudja megcsinálni őket. Gyermeke életében ezek a különféle dolgok együttesen alakítják ki gyermeke életminőségét.

1. Milyen gyermeke életminősége? Karikázza be azt az arcot, amelyik a leginkább kifejezi gyermeke életminőségét.

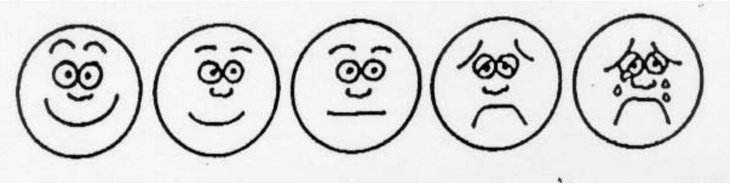

2. Milyen gyermeke betegsége ebben a pillanatban? Karikázza be azt az arcot, amelyik a leginkább kifejezi gyermeke betegségét.

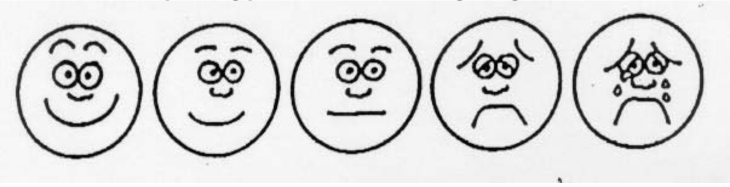

Copyright © 2007 Lakshmi Nandini Moorthy. Minden jog fenntartva.

A 3.-19. kérdés arra vonatkozik, hogy gyermeke a betegség miatt hogy érezte magát az elmúlt hónapban. Kérjük, karikázza be azt az arcot, amelyik legjobban kifejezi a válaszát.

3. Milyen érzés a gyermeke számára, hogy betegsége van?

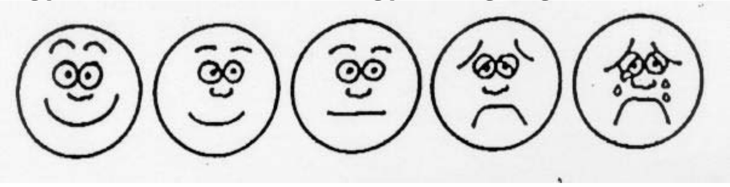

4. Milyen érzés a gyermeke számára, hogy betegként kell iskolába járnia?

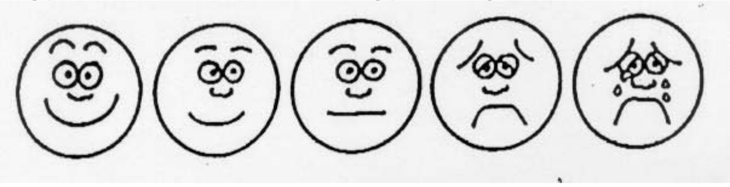

5. Milyen érzés a gyermeke számára, hogy minden nap betegként fel kell öltöznie?

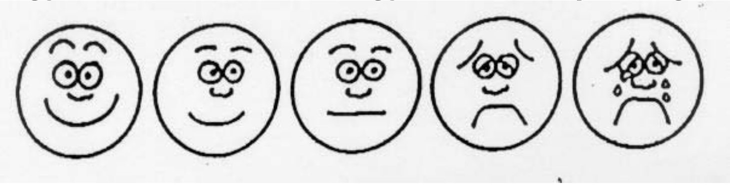

6. Mit gondol gyermeke a saját külsejéről amiatt, hogy beteg?

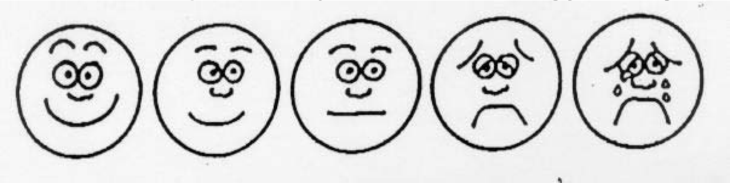

7. Hogyan befolyásolja a betegség a gyermeke önmagáról alkotott véleményét?

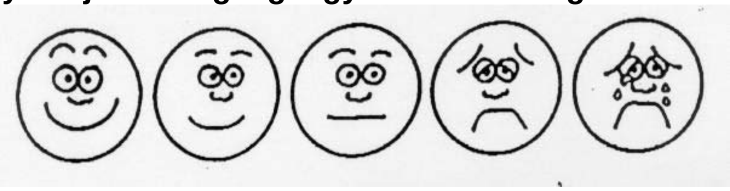

8. Hogy érzi a gyerek magát, amikor a betegségéről kell beszélnie?

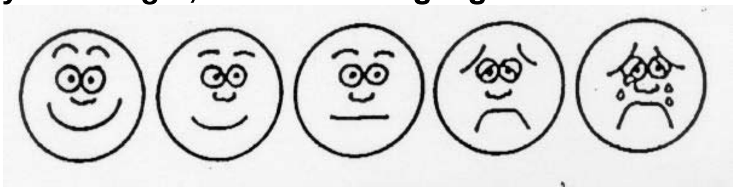

Copyright © 2007 Lakshmi Nandini Moorthy. Minden jog fenntartva.

9. Hogy érzi a gyerek magát, amikor a betegsége miatt orvoshoz kell mennie?

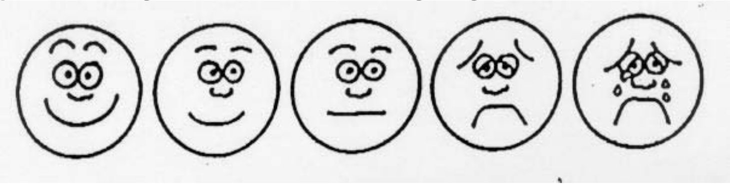

10. Hogy érzi a gyerek magát, amikor a betegsége miatt kórházba kell mennie?

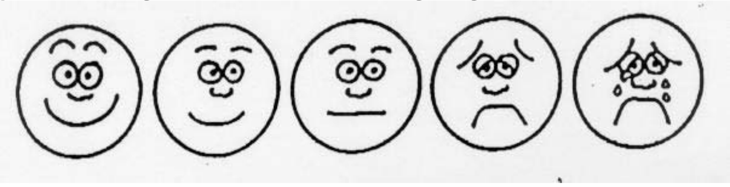

11. Hogyan hat a betegség a gyermek jövővel kapcsolatos érzéseire?

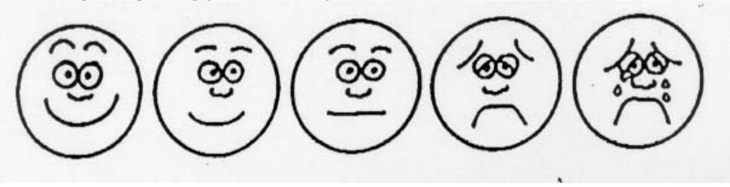

12. Hogy érzi a gyerek magát amiatt, hogy a betegségére gyógyszereket kell szednie?

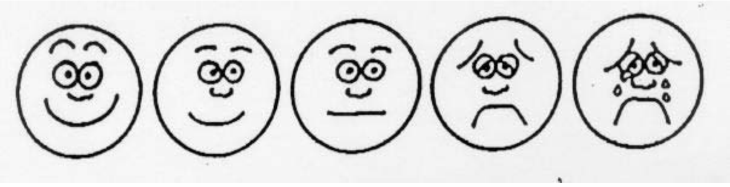

13. Mennyire aggódik azoknak a gyógyszereknek mellékhatásától gyermeke, amelyet a betegségére szed?

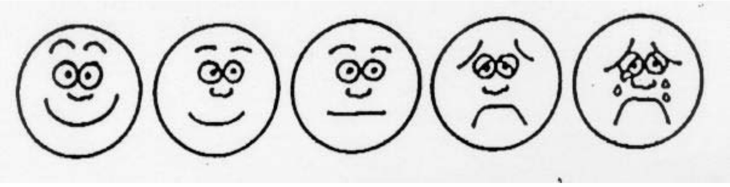

14. Mennyire aggódik gyermeke amiatt, hogy a betegsége romolhat?

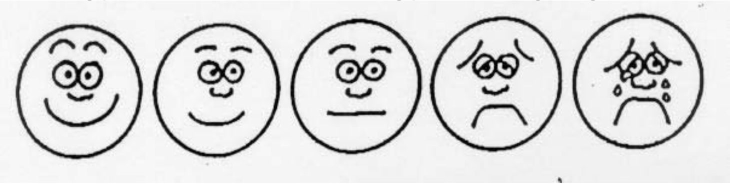

Copyright © 2007 Lakshmi Nandini Moorthy. Minden jog fenntartva.

15. **Mennyire érzi magát magányosnak gyermeke a betegsége miatt?**

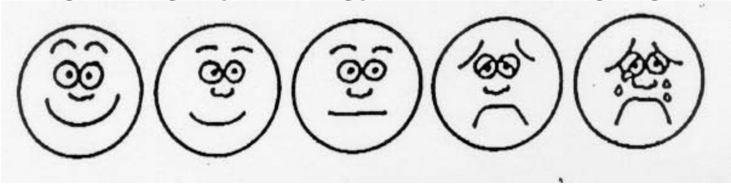

16. **Hogyan hat a családja a gyemeke kedélyére?**

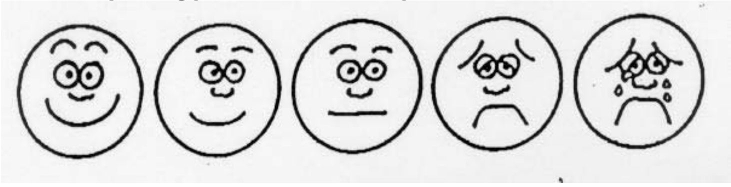

17. **Hogy érzi magát gyermeke a betegség okozta fájdalom miatt?**

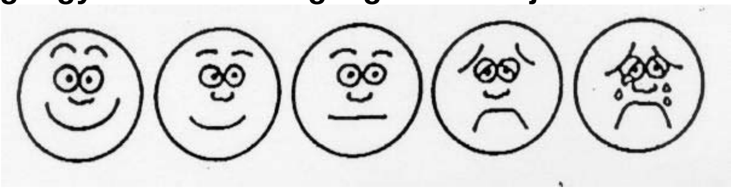

18. **Hogy érzi gyermeke magát attól, hogy a betegség miatt nem mehet ki a napra?**

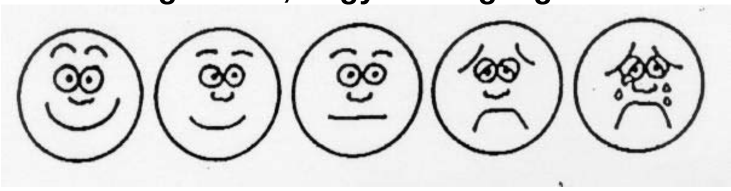

19. **Mennyire fárasztja a gyermekét a betegség?**

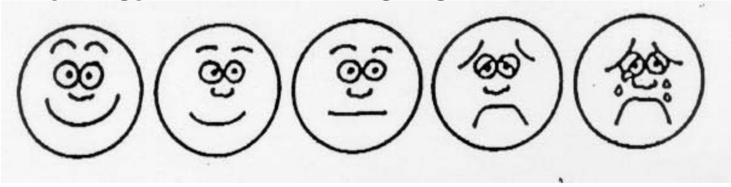

Copyright © 2007 Lakshmi Nandini Moorthy. Minden jog fenntartva.

A 20A-26A kérdésekre *igen*nel vagy *nem*mel válaszoljon, majd kövesse az utasításokat

20 A. Gyermeke barátai megváltoztak amiatt, hogy a gyermek beteg. Karikázza be az *igen*t vagy a *nem*et! Igen Nem

Ha igennel válaszolt, folytassa a 20 B-vel!

20 B. Hogyan hat a betegség a gyerek barátaihoz fűződő érzelmeire? Karikázza be azt az arcot, ahogy a gyerek érzi magát!

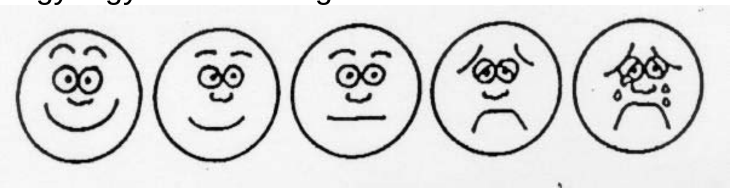

21 A. Gyermeke barátai értik, hogy a gyermek beteg. Igen Nem

Ha nemmel válaszolt, folytassa a 21 B-vel!

21 B. Mit érez gyermeke amiatt, hogy a barátai nem értik, hogy beteg? Karikázza be azt az arcot, amelyik gyermeke érzését tükrözi!

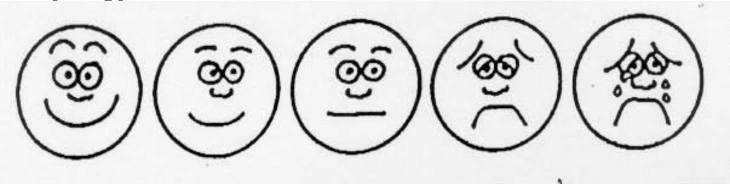

22 A. A betegség befolyásolja gyermeke iskolai munkáját. Igen Nem

Ha igennel válaszolt, folytassa a 22 B-vel!

22 B. Hogyan befolyásolja a betegség gyermeke iskolai munkájával kapcsolatos érzéseit? Karikázza be azt az arcot, amelyik gyermeke érzését tükrözi!

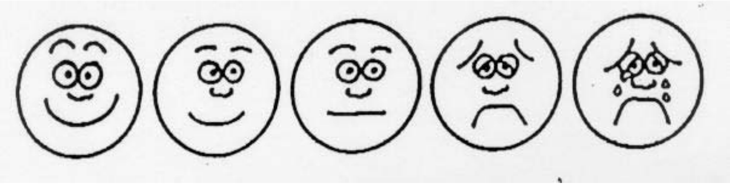

23 A. Gyermeke a betegség miatt hiányzik az iskolából. Igen Nem

Ha igennel válaszolt, folytassa a 23 B-vel!

23 B. Mit érez gyermeke amiatt, hogy a betegség miatt hiányzik az iskolából? Karikázza be azt az arcot, amelyik gyermeke érzését tükrözi!

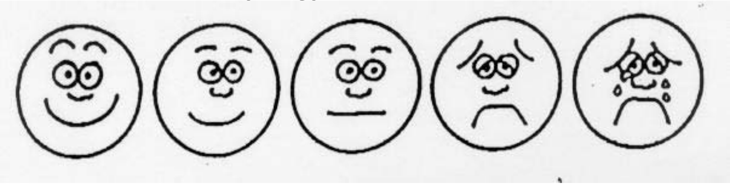

Copyright © 2007 Lakshmi Nandini Moorthy. Minden jog fenntartva.

24 A. A betegség befolyásolja gyermeke iskolán kívüli programjait. Igen Nem

Ha igennel válaszolt, folytassa a 24 B-vel!

24 B. Mit érez gyermeke amiatt, hogy a betegség befolyásolja az iskolán kívüli programjait? Karikázza be azt az arcot, amelyik gyermeke érzését tükrözi!

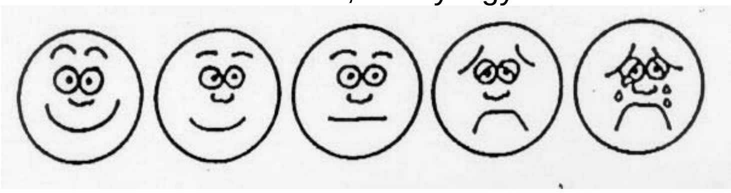

25 A. A betegség befolyásolja, hogyan szórakozik gyermeke a barátaival. Igen Nem

Ha igennel válaszolt, folytassa a 25 B-vel!

25 B. Mit érez a gyerek amiatt, hogy a betegség befolyásolja, hogyan szórakozik a barátaival? Karikázza be azt az arcot, amelyik gyermeke érzését tükrözi!

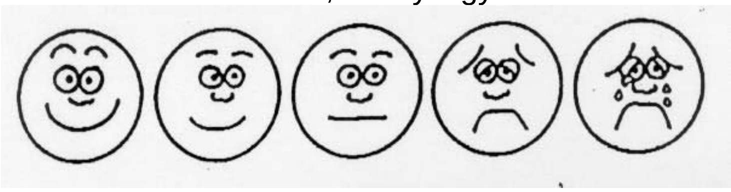

26 A. A betegség befolyásolja, hogy a gyermeke részt vesz-e testmozgással járó programokban (például iskolai sportjátékok, futás, sétálás, kedvenc szabadtéri játéka). Igen Nem

Ha igennel válaszolt, folytassa a 26 B-vel!

26 B. Mit érez a gyerek amiatt, hogy a betegség befolyásolja, részt vesz-e testmozgással járó programokban (például iskolai sportjátékok, futás, sétálás, kedvenc szabadtéri játéka)? Karikázza be azt az arcot, amelyik leginkább kifejezi, amit gyermeke érez!

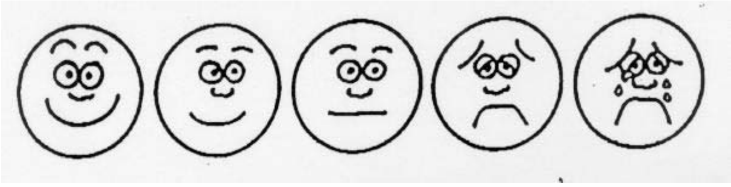

Copyright © 2007 Lakshmi Nandini Moorthy. Minden jog fenntartva.

SIMPLE MEASURE OF IMPACT OF ILLNESS IN YOUNGSTERS© (SMILY-Illness) – Gyerek kérdőív

Vizsgálat száma __ Dátum __/__/20__

A kérdőív készítésekor az alábbi öt arcot választottuk ki a Wong-Baker fájdalomértékelő skáláról (Wong-Baker FACES Pain Rating Scale). A módosítás D.L. Wong engedélyével történt. Minden kérdés alatt ez az öt arc szerepel.

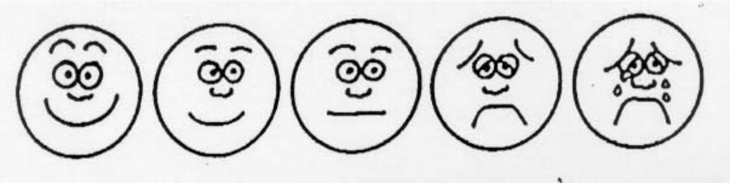

Engedéllyel átvéve: Wong DL, Hockenberry-Eaton M, Wilson D, Winklestein ML, Schwartz P.: Wong's Essentials of Pediatric Nursing, ed. 6, St. Louis, 2001, Mosby, p 1301. Copyright Mosby.

A SMILY –illness szerzői jogtulajdonosa L. Nandini Moorthy, MD MS, RWJMS-UMDNJ, New Brunswick, NJ. A kérdőívet jelenleg kizárólag L. Nandini Moorthy, MD MS és az általa kijelölt kutatók használhatják.

KÉRDÉSEK

A betegségedről és az azzal kapcsolatos érzéseidről teszünk fel kérdéseket ebben a kérdőívben. Nincsenek rossz válaszok. Kérdezz meg bármit, amit nem értesz. Amennyiben kiegészítő megjegyzéseket tennél, az 5. és a 6. oldal hátára írhatsz.

Különféle dolgok hatnak életedre, vannak olyanok, amelyektől vidám leszel, másoktól elszomorodsz. Vannak olyan dolgok, amelyek fontosak számodra - ezeket vagy képes vagy megcsinálni, vagy épp nem tudod megcsinálni őket. Életedben ezek a különféle dolgok együttesen alakítják ki életminőségedet.

1. Milyen a betegséged most? Karikázd be azt az arcot, amelyik a legjobban kifejezi a betegségedet!

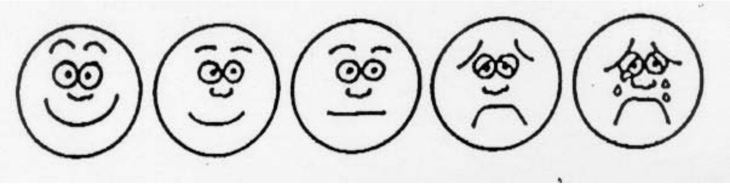

2. Milyen a betegséged ebben a pillanatban? Karikázd be azt az arcot, amelyik a legjobban kifejezi a betegséged!

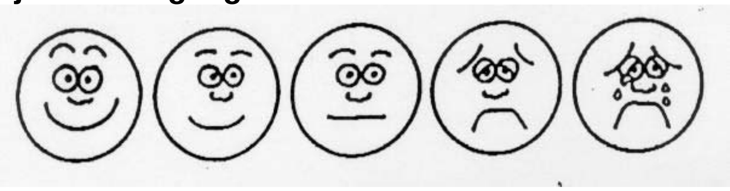

Copyright © 2007 Lakshmi Nandini Moorthy. Minden jog fenntartva.

A 3-19. kérdés arra vonatkozik, hogy az elmúlt egy hónapban hogyan érezted magad a betegséged miatt. Karikázd be azt az arcot, amelyik legjobban kifejezi, hogy érzed magad!

3. Hogy érzed magad amiatt, hogy betegséged van?

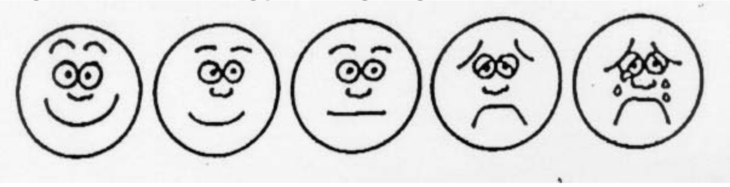

4. Milyen érzés, hogy betegként kell iskolába járnod?

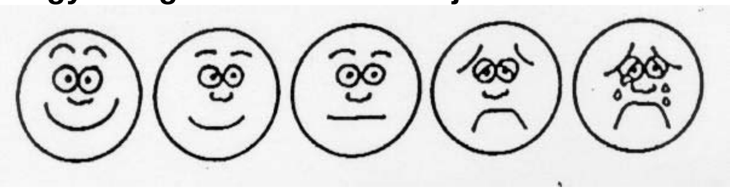

5. Milyen érzés, hogy betegként kell mindennap felöltöznöd?

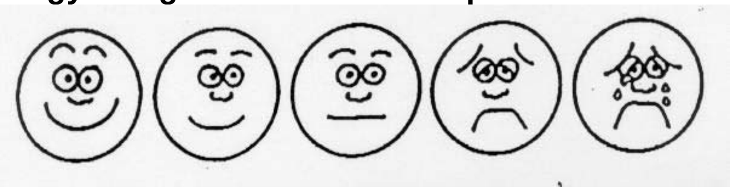

6. Mit gondolsz a saját külsődről amiatt, hogy beteg vagy?

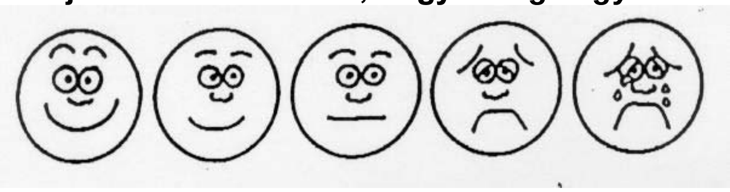

7. Hogy befolyásolja a betegség a magadról alkotott véleményed?

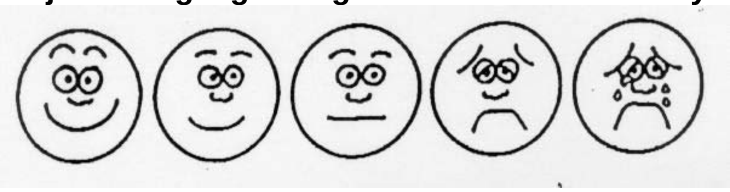

8. Hogy érzed magad, amikor a betegségedről kell beszélned?

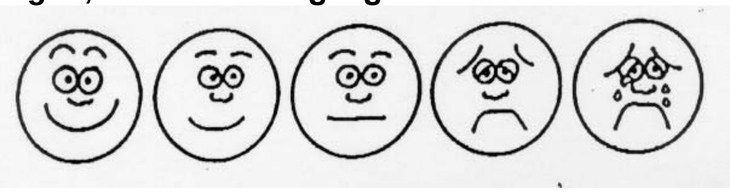

Copyright © 2007 Lakshmi Nandini Moorthy. Minden jog fenntartva.

9. Hogy érzed magad, amikor a betegség miatt orvoshoz kell menned?

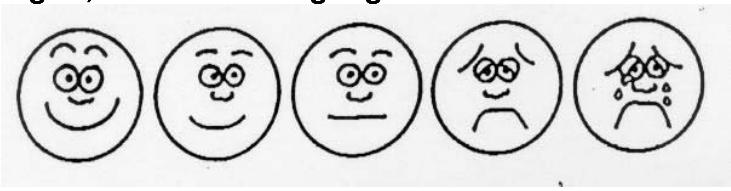

10. Hogy érzed magad, amikor a betegség miatt kórházba kell menned?

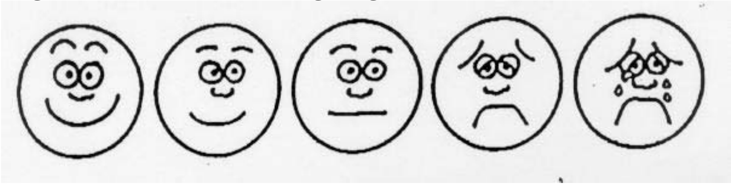

11. Hogyan hat a betegség a jövővel kapcsolatos érzéseidre?

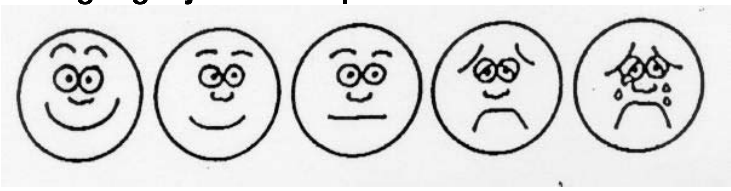

12. Hogy érzed magad amiatt, hogy a betegségedre gyógyszereket kell szedned?

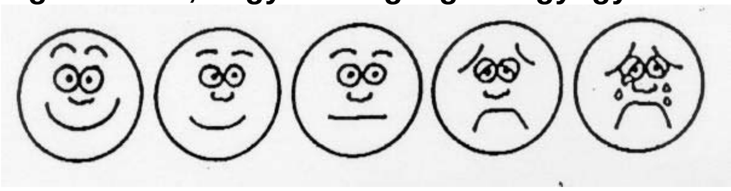

13. Mennyire aggódsz azoknak a gyógyszereknek a mellékhatásától, amelyeket a betegségedre szedsz?

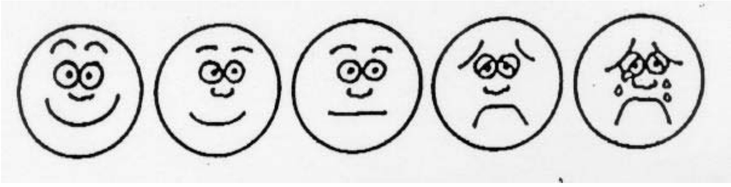

14. Mennyire aggódsz amiatt, hogy a betegséged romolhat?

Copyright © 2007 Lakshmi Nandini Moorthy. Minden jog fenntartva.

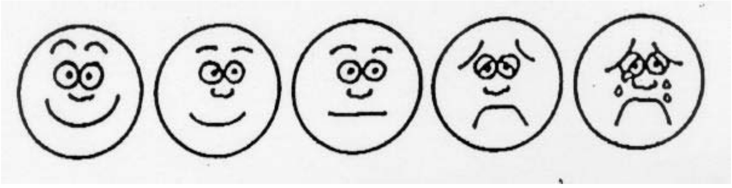

15. **Mennyire érzed magad magányosnak a betegséged miatt?**

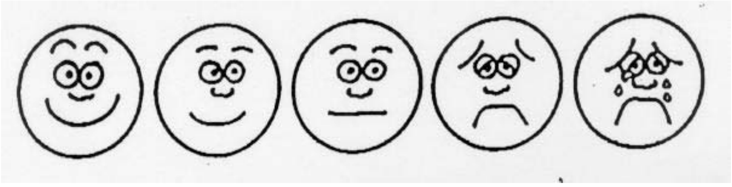

16. **A családod hogyan hat a kedélyedre?**

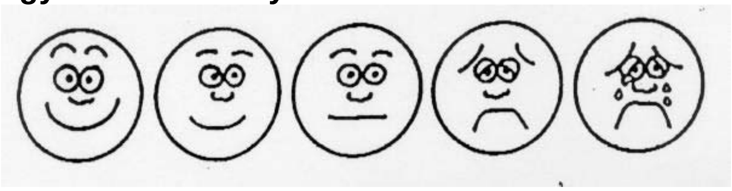

17. **Mit érzel amiatt, hogy a betegséged fájdalommal jár?**

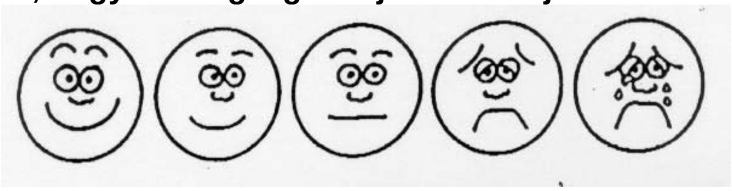

18. **Mit érzel amiatt, hogy a betegséged miatt nem mehetsz ki a napra?**

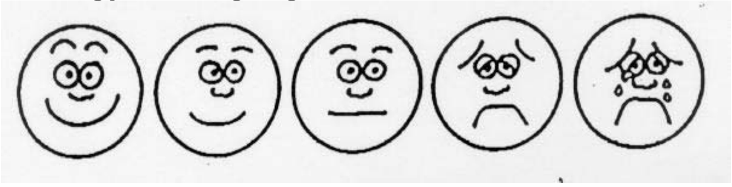

19. **Mennyire fáraszt a betegséged?**

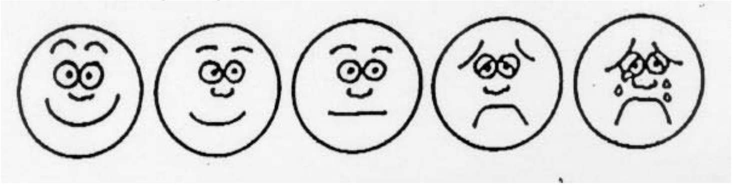

Copyright © 2007 Lakshmi Nandini Moorthy. Minden jog fenntartva.

A 20A-26A kérdésekre *igen*nel vagy *nem*mel válaszolj, majd kövesd az utasításokat!

20 A. Barátaim megváltoztak amiatt, hogy beteg vagyok. Karikázd be az *igen*t vagy a *nem*et!

Igen Nem

Ha igennel válaszoltál, folytasd a 20 B-vel!

20 B. A betegség hatására te hogy gondolsz a barátaidra? Karikázd be azt az arcot, ahogy érzed magad!

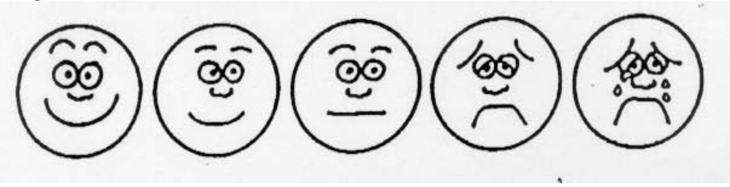

21 A. Barátaim értik, hogy beteg vagyok. Igen Nem

Ha nemmel válaszoltál, folytasd a 21 B-vel!

21 B. Milyen érzés, hogy a barátaid nem értik, hogy beteg vagy? Karikázd be azt az arcot, amelyik az érzéseid tükrözi!

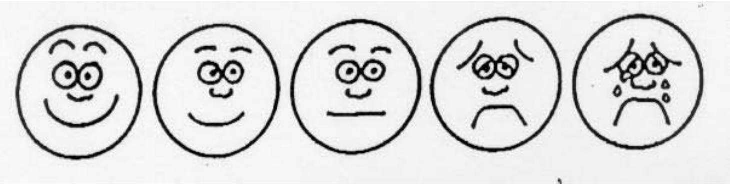

22 A. A betegségem befolyásolja iskolai munkámat. Igen Nem

Ha igennel válaszoltál, folytasd a 22 B-vel!

22 B. Hogyan befolyásolja a betegség az iskolai munkával kapcsolatos érzéseidet? Karikázd be azt az arcot, amelyik az érzéseid tükrözi!

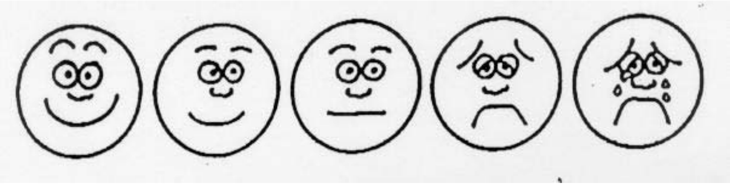

23 A. A betegségem miatt hiányzom az iskolából. Igen Nem

Ha igennel válaszoltál, folytasd a 23 B-vel!

23 B. Milyen érzés, hogy a betegség miatt hiányzol az iskolából? Karikázd be azt az arcot, amelyik az érzéseid tükrözi!

Copyright © 2007 Lakshmi Nandini Moorthy. Minden jog fenntartva.

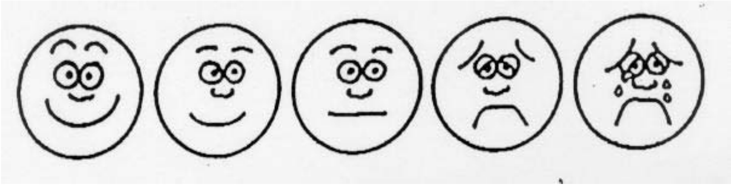

24 A. A betegségem befolyásolja az iskolán kívüli programjaimat. Igen Nem

Ha igennel válaszoltál, folytasd a 24 B-vel!

24 B. Milyen érzés, hogy a betegség befolyásolja az iskolán kívüli programjaidat? Karikázd be azt az arcot, amelyik az érzéseid tükrözi!

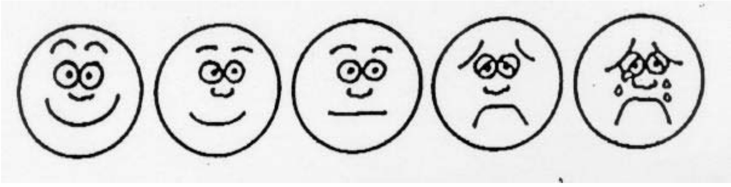

25 A. A betegségem befolyásolja, hogyan szórakozom a barátaimmal. Igen Nem

Ha igennel válaszoltál, folytasd a 25 B-vel!

25 B. Milyen érzés, hogy a betegség befolyásolja, hogyan szórakozol a barátaiddal? Karikázd be azt az arcot, amelyik az érzéseid tükrözi!

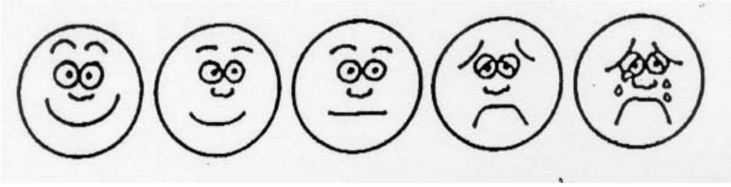

26 A. A betegségem befolyásolja, hogy részt veszek-e testmozgással járó programokban (például iskolai sportjátékok, futás, sétálás, kedvenc szabadtéri játékom).

Igen Nem

Ha igennel válaszoltál, folytasd a 26 B-vel!

26 B. Milyen érzés, hogy a betegség befolyásolja, részt veszel-e testmozgással járó programokban (például iskolai sportjátékok, futás, sétálás, kedvenc szabadtéri játékod)? Karikázd be azt az arcot, amelyik az érzéseid tükrözi!

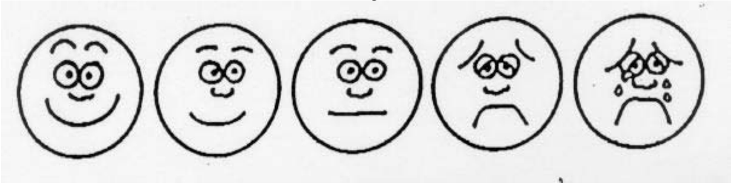

Copyright © 2007 Lakshmi Nandini Moorthy. Minden jog fenntartva.

SEMPLICE MISURA DELL'IMPATTO DELLA MALATTIA NEI BAMBINI (SMILY-Illness) – Resoconto del Bambino

Italian **Studio numero __ Data __/__/20__**

Per questo questionario, abbiamo preso le 5 facce qui sotto da quelle della scala di valutazione del dolore FACES di Wong-Baker, che sono state modificate con il permesso di D.L. Wong. Queste 5 facce compariranno sotto ogni domanda.

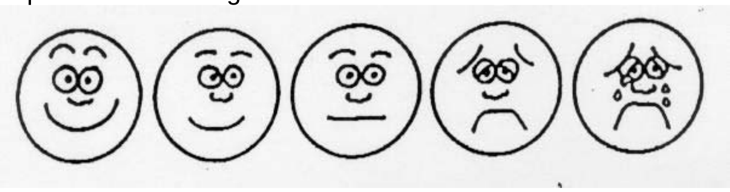

Da Wong DL, Hockenberry-Eaton M, Wilson D,Winklestein ML, Schwartz P.: *Wong's Essentials of Pediatric Nursing*, 6[a] ed., St. Louis, 2001, Mosby, p 1301. Copyright Mosby. Ristampato con autorizzazione.

Il copyright dello SMILY-Illness è di L. Nandini Moorthy, MD MS, RWJMS-UMDNJ, New Brunswick, NJ. Il questionario può essere usato solo da L. Nandini Moorthy, MD MS e dal suo personale di ricerca designato.

DOMANDE

In questo questionario, ti faremo molte domande sulla tua malattia e su quello che provi. Non esistono risposte sbagliate. Chiedici qualunque cosa tu non capisca. Se desideri scrivere qualunque commento, fallo dietro alle pagine 5 e 6.

Diverse cose influenzano la tua vita e ti rendono felice o triste. Ci sono alcune cose che per te sono importanti, che sei in grado di fare (o che non sei in grado di fare). Tutte queste diverse cose nel loro insieme costituiscono la tua qualità di vita.

1. Com'è la tua qualità di vita? Segna (X) la faccia che meglio indica la tua qualità di vita.

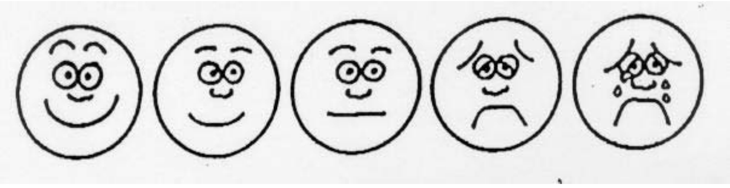

2. Com'è la malattia in questo momento? Segna (X) la faccia che meglio indica com'è la malattia.

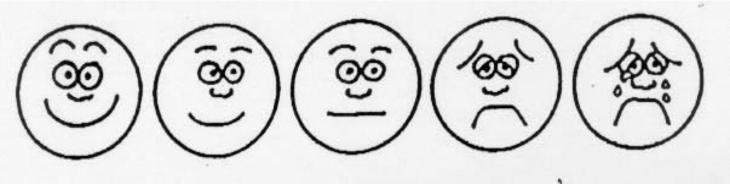

Copyright © 2007 Lakshmi Nandini Moorthy. Tutti i diritti riservati.

Le domande da 3 a 19 sono su come ti sei sentito/a nell'ultimo mese a causa della malattia. Segna (X) la faccia che indica meglio come ti sei sentito/a.

3. Come ti senti a causa della malattia?

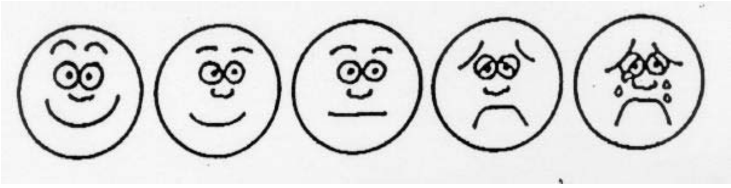

4. Come ti senti a causa della malattia nell'andare a scuola?

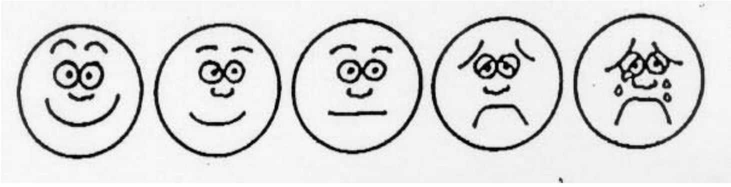

5. Come ti senti a causa della malattia nel vestirsi?

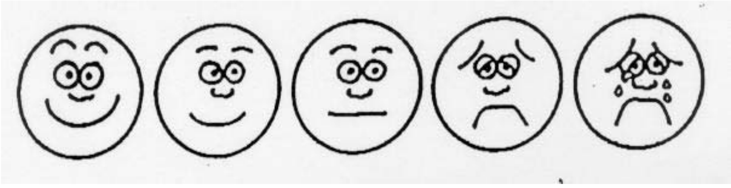

6. Come ti senti a causa della malattia riguardo al tuo aspetto fisico?

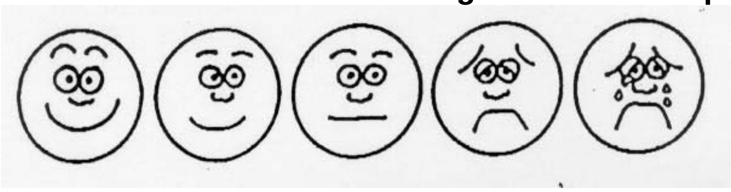

7. Come ti senti a causa della malattia riguardo a te stesso/a?

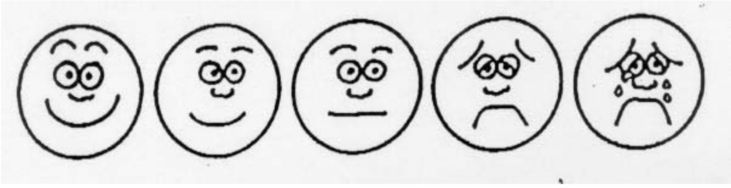

8. Come ti senti quando hai bisogno di parlare della tua malattia?

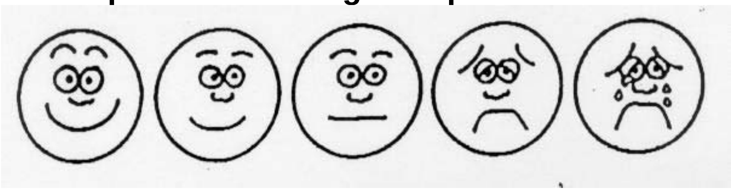

Copyright © 2007 Lakshmi Nandini Moorthy. Tutti i diritti riservati.

9. Come ti senti riguardo all'andare dal dottore a causa di una malattia?

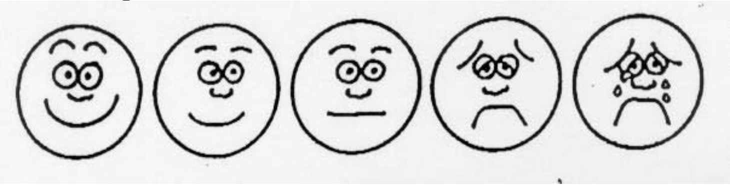

10. Come ti senti riguardo all'andare in ospedale a causa di una malattia?

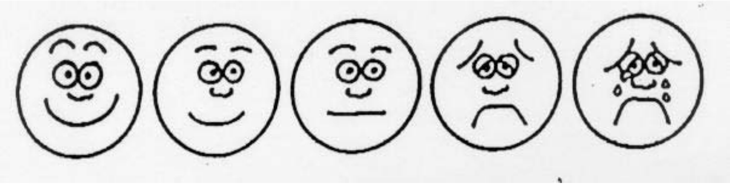

11. Come ti senti a causa della malattia riguardo al tuo futuro?

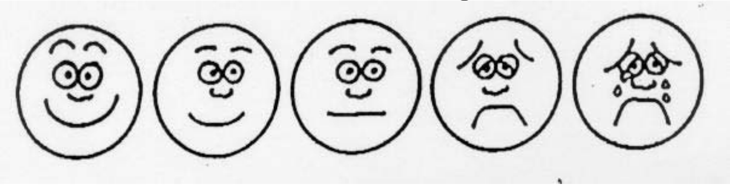

12. Come ti senti per il fatto di assumere farmaci a causa della malattia?

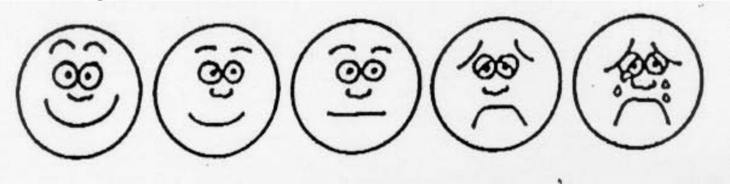

13. Quanto sei preoccupato/a degli effetti collaterali delle medicine che prendi per la malattia?

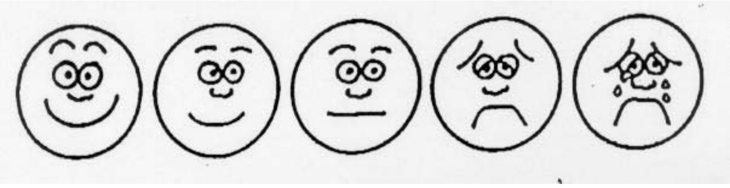

14. Quanto sei preoccupato/a che la malattia peggiori?

Copyright © 2007 Lakshmi Nandini Moorthy. Tutti i diritti riservati.

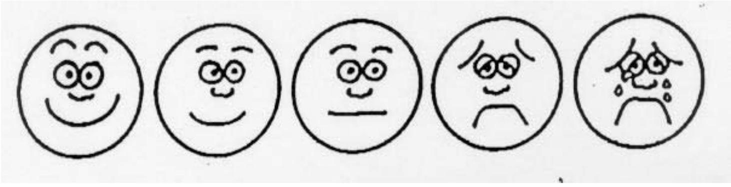

15. Quanto ti senti solo/a a causa della malattia?

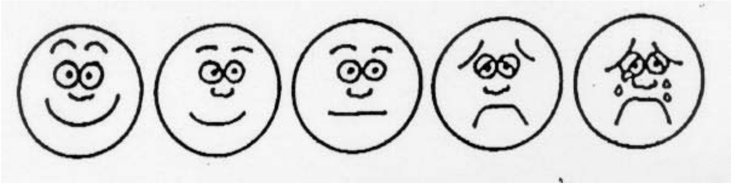

16. Come ti fa sentire la tua famiglia?

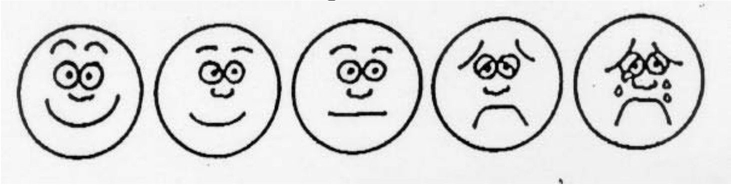

17. Come ti fa sentire il dolore a causa della malattia?

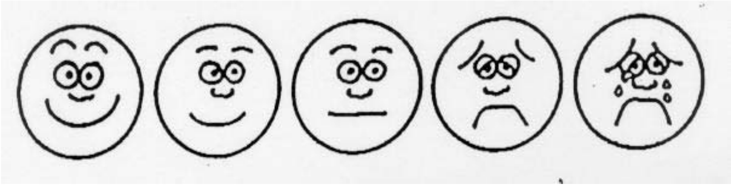

18. Come ti fa sentire il fatto di non potere uscire al sole a causa della malattia?

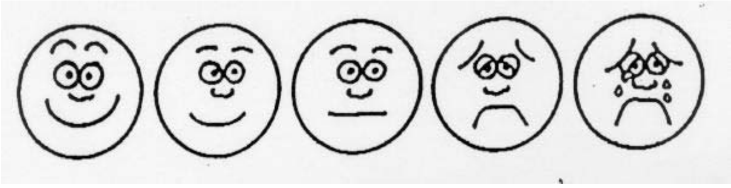

19. Quanto ti fa sentire stanco/a la malattia?

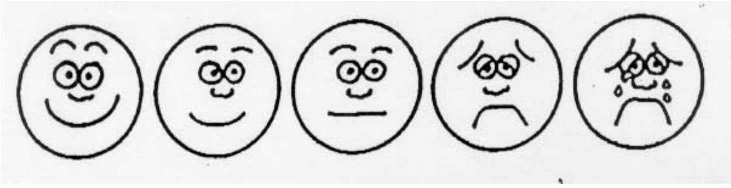

Copyright © 2007 Lakshmi Nandini Moorthy. Tutti i diritti riservati.

Per le domande da 20 A a 26 A, rispondi "sì" o "no". Poi segui le istruzioni.

20 A. I miei amici sono cambiati a causa della mia malattia. Segna "sì" o "no". Sì No

Se "sì", vai alla domanda 20 B.

20 B. Come ti fa sentire l'avere una malattia per quel che riguarda i tuoi amici?

Fai un cerchio attorno alla faccia che indica come ti senti.

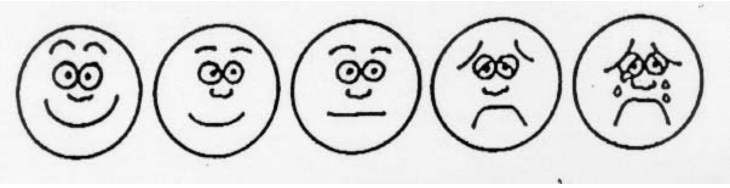

21 A. I miei amici capiscono che io ho una malattia. Sì No

Se hai risposto "no", vai alla domanda 21 B.

21 B. Come ti fa sentire il fatto che i tuoi amici non capiscono che tu hai una malattia? Fai un cerchio attorno alla faccia che indica come ti senti.

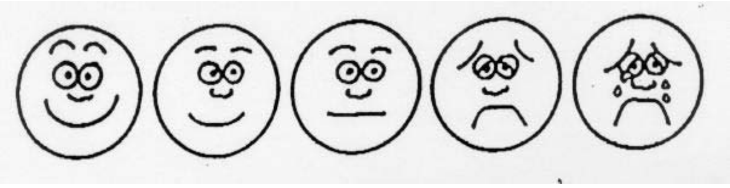

22 A. La malattia influisce sul mio lavoro scolastico. Sì No

Se "sì", vai alla domanda 22 B.

22 B. Come ti fa sentire l'avere la malattia per quel che riguarda il tuo lavoro scolastico? Fai un cerchio attorno alla faccia che indica come ti senti.

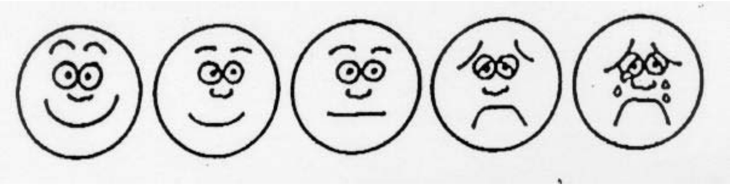

23 A. Perdo giorni di scuola a causa della malattia. Sì No

Se "sì", vai alla domanda 23 B.

23 B. Come ti senti riguardo al perdere giorni di scuola a causa della mlattia?

Fai un cerchio attorno alla faccia che indica come ti senti.

Copyright © 2007 Lakshmi Nandini Moorthy. Tutti i diritti riservati.

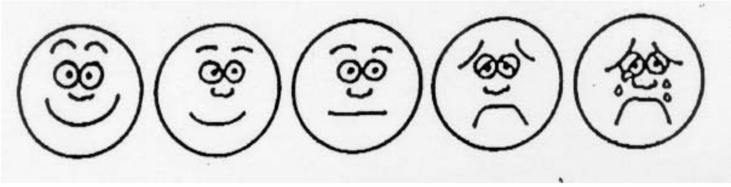

24 A. La malattia influisce sulle attività di Suo/a figlio/a al di fuori della scuola.

Sì **No**

Se hai risposto "sì", vai alla domanda 24 B.

24 B. Come ti senti per il fatto che la malattia influenza le tue attività all'infuori della scuola? Fai un cerchio attorno alla faccia che indica come ti senti.

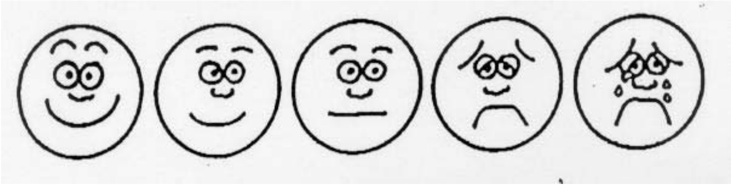

25 A. La malattia influisce su quanto mi diverto con i miei amici.

Sì **No**

Se hai risposto "sì", vai alla domanda 25 B.

25 B. Come ti fa sentire il fatto che la malattia influisce su quanto ti diverti con i tuoi amici? Fai un cerchio attorno alla faccia che indica come ti senti.

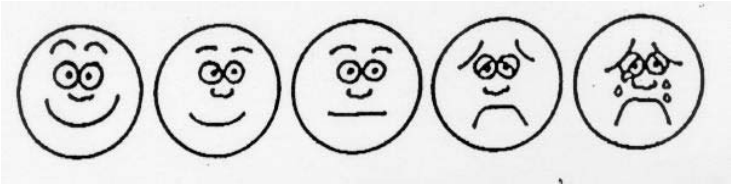

26 A. La malattia influisce sulla mia partecipazione alle attività fisiche (come la ginnastica a scuola, la corsa, il camminare, il giocare al gioco all'aperto preferito).

Sì **No**

Se hai risposto "sì", vai alla domanda 26 B.

26 B. Come ti senti per il fatto che la malattia influisce sulla tua partecipazione alle attività fisiche (come la ginnastica a scuola, la corsa, il camminare, il giocare al gioco all'aperto preferito)? Fai un cerchio attorno alla faccia che indica come ti senti.

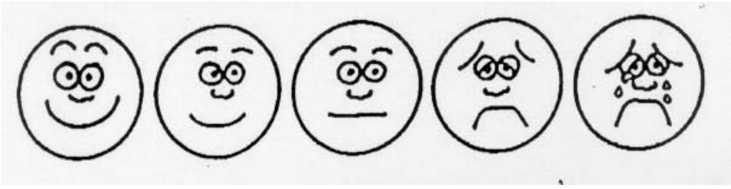

Copyright © 2007 Lakshmi Nandini Moorthy. Tutti i diritti riservati.

Copyright © 2007 Lakshmi Nandini Moorthy. Tutti i diritti riservati.

SEMPLICE MISURA DELL'IMPATTO DELLA MALATTIA NEI BAMBINI (SMILY-Illness) – Resoconto dei Genitori

Studio numero __ Data __/__/20__

Per questo questionario, abbiamo preso le 5 facce qui sotto da quelle della scala di valutazione del dolore FACES di Wong-Baker, che sono state modificate con il permesso di D.L. Wong. Queste 5 facce compariranno sotto ogni domanda.

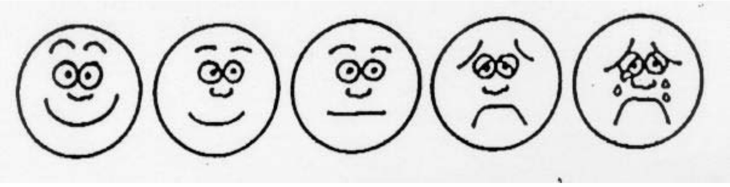

Da Wong DL, Hockenberry-Eaton M, Wilson D,Winklestein ML, Schwartz P.: *Wong's Essentials of Pediatric Nursing*, 6[a] ed., St. Louis, 2001, Mosby, p 1301. Copyright Mosby. Ristampato con autorizzazione.

Il copyright dello SMILY-Illness è di L. Nandini Moorthy, MD MS, RWJMS-UMDNJ, New Brunswick, NJ. Il questionario può essere usato solo da L. Nandini Moorthy, MD MS e dal suo personale di ricerca designato.

DOMANDE

In questo questionario, Le faremo molte domande sulla malattia di Suo/a figlio/a e su quello che lui/lei prova. Non esistono risposte sbagliate. Ci chieda qualunque cosa Lei non capisca. Se desidera scrivere qualunque commento, lo faccia dietro alle pagine 5 e 6.

Diverse cose influenzano la vita di Suo/a figlio/a e lo/a rendono felice o triste. Ci sono alcune cose che per Suo/a figlio/a sono importanti, che è in grado di fare (o che non è in grado di fare). Tutte queste diverse cose nel loro insieme costituiscono la qualità di vita di Suo/a figlio/a.

1. **Com'è la qualità di vita di Suo/a figlio/a? Segni (X) la faccia che meglio indica la qualità di vita di Suo/a figlio/a.**

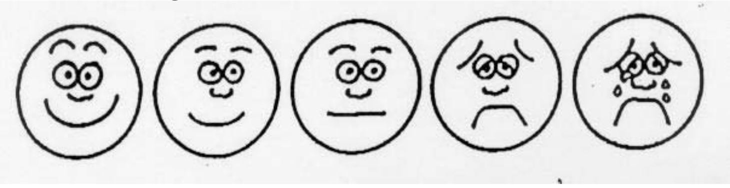

2. **Com'è la malattia di Suo/a figlio/a in questo momento? Segni (X) la faccia che meglio indica com'è la malattia di Suo/a figlio/a.**

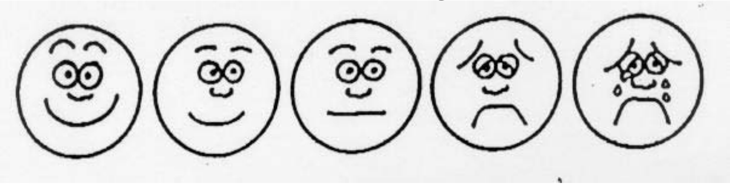

Copyright © 2007 Lakshmi Nandini Moorthy. Tutti i diritti riservati.

Le domande da 3 a 19 sono su come si è sentito Suo/a figlio/a nell'ultimo mese a causa della malattia. Segni (X) la faccia che meglio indica come si è sentito Suo/a figlio/a.

3. **Come si sente Suo/a figlio/a a causa della malattia?**

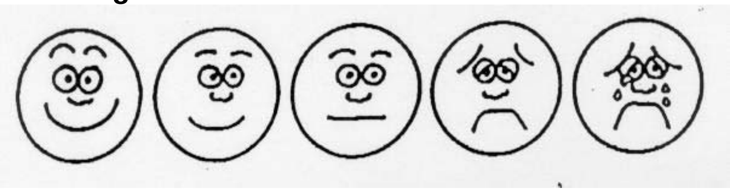

4. **Come si sente Suo/a figlio/a a causa della malattia nell'andare a scuola?**

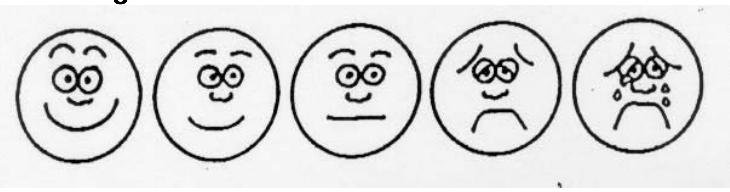

5. **Come si sente Suo/a figlio/a a causa della malattia nel vestirsi?**

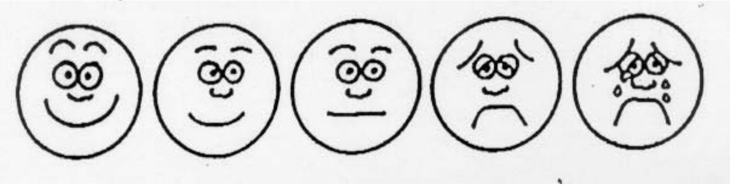

6. **Come si sente Suo/a figlio/a a causa della malattia riguardo al proprio aspetto fisico?**

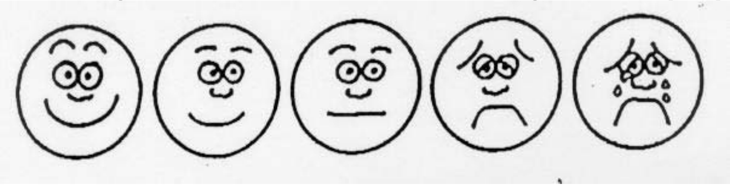

7. **Come si sente Suo/a figlio/a a causa della malattia riguardo a se stesso/a?**

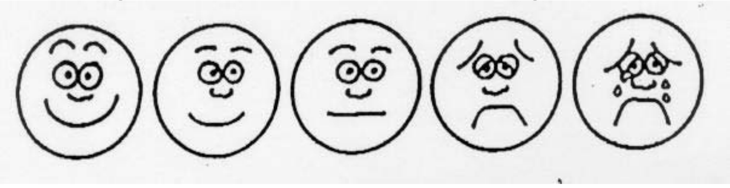

8. **Come si sente Suo/a figlio/a quando ha bisogno di parlare della propria malattia?**

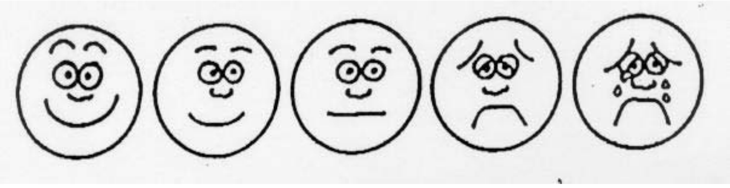

Copyright © 2007 Lakshmi Nandini Moorthy. Tutti i diritti riservati.

9. Come si sente Suo/a figlio/a riguardo all'andare dal dottore a causa della malattia?

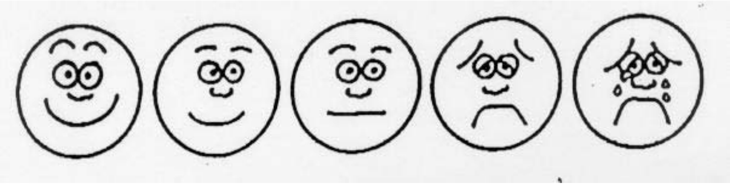

10. Come si sente Suo/a figlio/a riguardo all'andare in ospedale a causa della malattia?

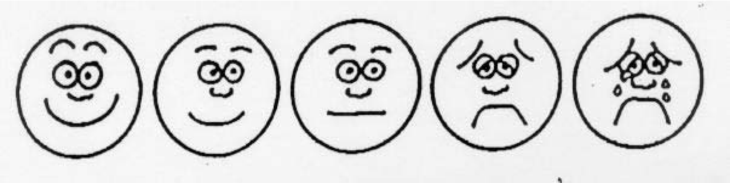

11. Come si sente Suo/a figlio/a a causa della malattia riguardo al proprio futuro?

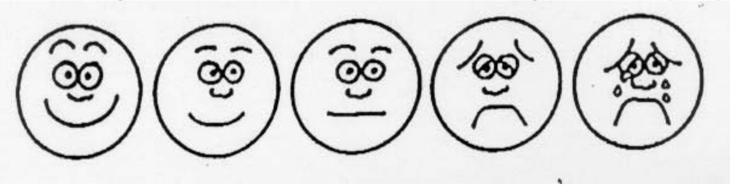

12. Come si sente Suo/a figlio/a per il fatto di assumere farmaci a causa della malattia?

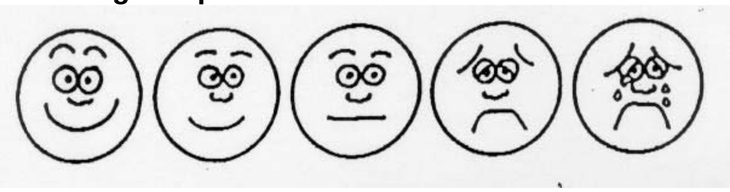

13. Quanto è preoccupato/a Suo/a figlio/a degli effetti collaterali delle medicine che prende per la malattia?

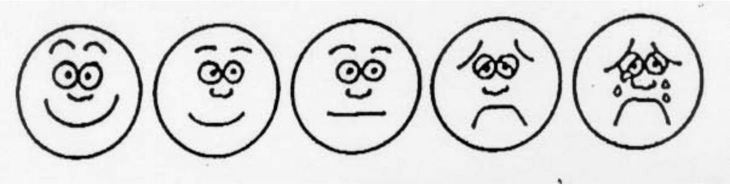

14. Quanto è preoccupato/a Suo/a figlio/a che la malattia peggiori?

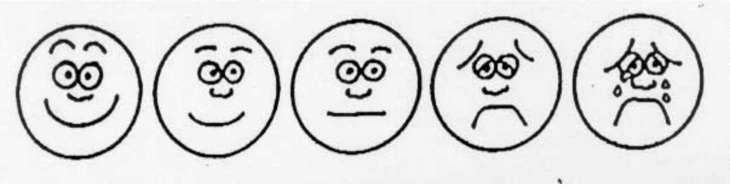

Copyright © 2007 Lakshmi Nandini Moorthy. Tutti i diritti riservati.

15. **Quanto si sente solo/a Suo/a figlio/a a causa della malattia?**

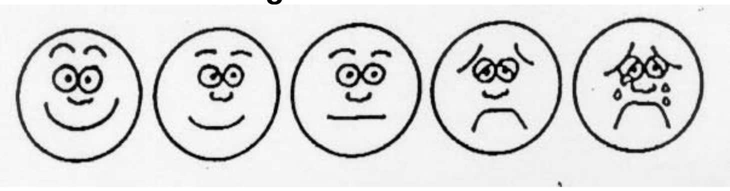

16. **La propria famiglia come fa sentire Suo/a figlio/a?**

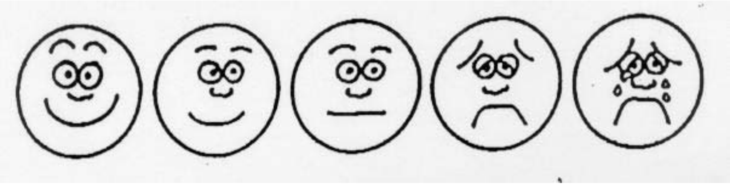

17. **Il dolore a causa della malattia come fa sentire Suo/a figlio/a?**

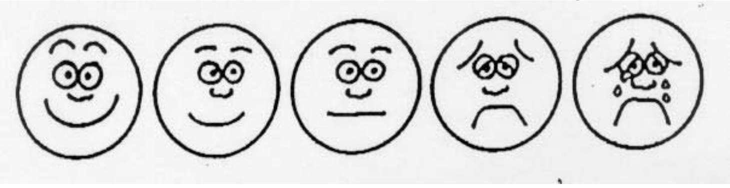

18. **Come si sente Suo/a figlio/a per il fatto di non potere uscire al sole a causa della malattia?**

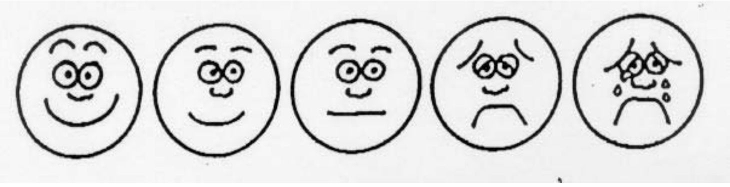

19. **Quanto la malattia fa sentire stanco/a Suo/a figlio/a?**

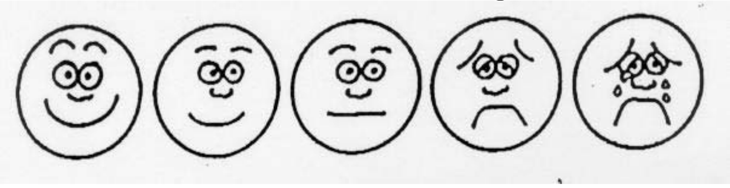

Per le domande da 20 A a 26 A, risponda “sì” o “no”. Poi segua le istruzioni.

20 A. Gli amici di Suo/a figlio/a sono cambiati a causa della sua malattia. Segnare “sì” o “no”. **Sì** **No**

Copyright © 2007 Lakshmi Nandini Moorthy. Tutti i diritti riservati.

Se "sì", vada alla domanda 20 B.

20 B. Come fa sentire Suo/a figlio/a l'avere la malattia per quel che riguarda i propri amici?

Faccia un cerchio attorno alla faccia che indica come si sente Suo/a figlio/a.

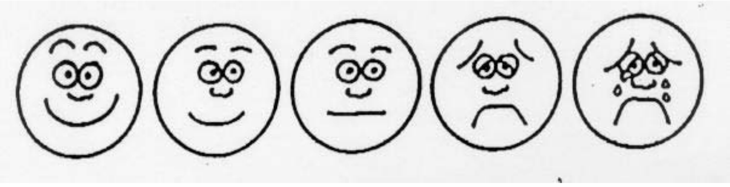

21 A. Gli amici di Suo/a figlio/a capiscono che lui/lei ha una malattia. Sì No

Se ha risposto "no", vada alla domanda 21 B.

21 B. Come fa sentire Suo/a figlio/a il fatto che i propri amici non capiscono che lui/lei ha una malattia? Faccia un cerchio attorno alla faccia che indica come si sente Suo/a figlio/a.

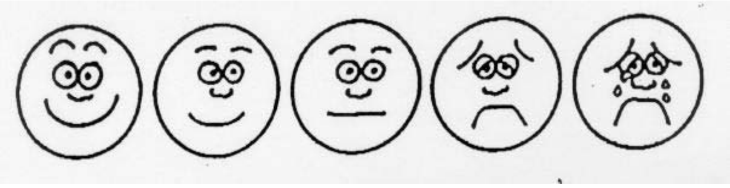

22 A. Una malattia influisce sul lavoro scolastico di Suo/a figlio/a. Sì No

Se "sì", vada alla domanda 22 B.

22 B. Come fa sentire Suo/a figlio/a l'avere la malattia per quel che riguarda il lavoro scolastico?

Faccia un cerchio attorno alla faccia che indica come si sente Suo/a figlio/a.

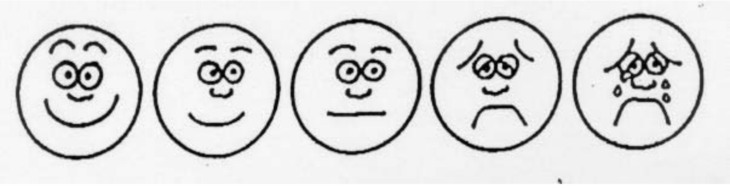

23 A. Suo/a figlio/a perde giorni di scuola a causa della malattia. Sì No

Se "sì", vada alla domanda 23 B.

23 B. Come si sente Suo/a figlio/a riguardo al perdere giorni di scuola a causa della malattia? Faccia un cerchio attorno alla faccia che indica come si sente Suo/a figlio/a.

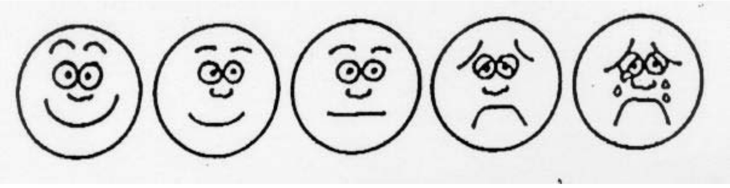

24 A. La malattia influisce sulle attività di Suo/a figlio/a al di fuori della scuola.

Sì No

Se ha risposto "sì", vada alla domanda 24 B.

Copyright © 2007 Lakshmi Nandini Moorthy. Tutti i diritti riservati.

24 B. Come si sente Suo/a figlio/a per il fatto che la malattia influenza le sue attività all'infuori della scuola? Faccia un cerchio attorno alla faccia che indica come si sente Suo/a figlio/a.

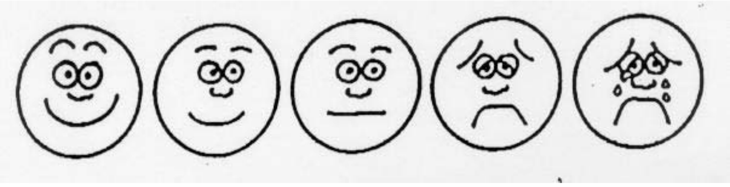

25 A. La malattia influisce su quanto Suo/a figlio/a si diverte con i propri amici.

Sì **No**

Se ha risposto "sì", vada alla domanda 25 B.

25 B. Come fa sentire Suo/a figlio/a il fatto che la malattia influisce sul divertimento che ha con i propri amici? Faccia un cerchio attorno alla faccia che indica come si sente Suo/a figlio/a.

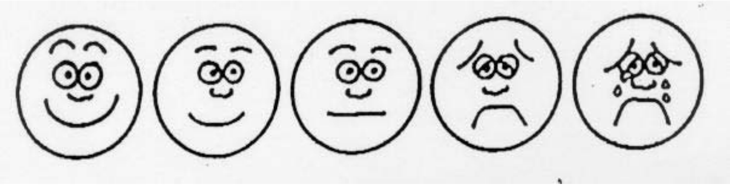

26 A. La malattia influisce sulla partecipazione di Suo/a figlio/a alle attività fisiche (come la ginnastica a scuola, la corsa, il camminare, il giocare al gioco all'aperto preferito).

Sì **No**

Se ha risposto "sì", vada alla domanda 26 B.

26 B. Come si sente Suo/a figlio/a per il fatto che la malattia influisce sulla sua partecipazione alle attività fisiche (come la ginnastica a scuola, la corsa, il camminare, il giocare al gioco all'aperto preferito)? Faccia un cerchio attorno alla faccia che indica come si sente Suo/a figlio/a.

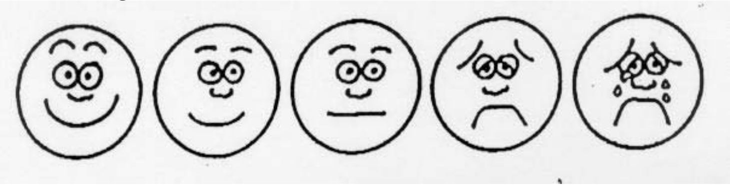

Copyright © 2007 Lakshmi Nandini Moorthy. Tutti i diritti riservati.

子供における病気の影響に関する簡易評価
(SMILY-Illness)– 保護者用調査票 – Parent Version

Japanese　　試験番号 __　日付 **20**__年__月__日

この調査では、“Wong-Baker FACES Pain Rating Scale”というフェイススケール（表情を用いた評価法）を、D.L. Wong 氏の許可のもとに改変したものを使います。これらの 5 つの顔は、どの質問にも出てきます。

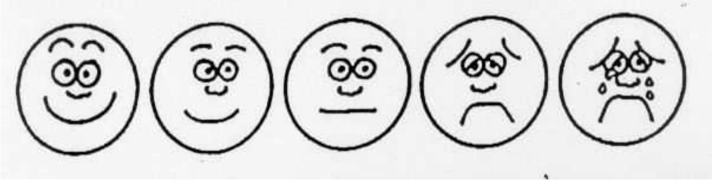

出典 Wong DL, Hockenberry-Eaton M, Wilson D, Winklestein ML, Schwartz P.著「Wong's Essentials of Pediatric Nursing」第 6 版、米国セントルイス、2001 年、Mosby 社、p 1301 著作権 Mosby 社。転載許可取得。

スマイリーの著作権は、**L. Nandini Moorthy** 医学博士および理学修士（米国ニュージャージー州ニューブランズウィック市 **RWJMS-UMDNJ** 所属）に帰属します。現時点において、**L. Nandini Moorthy** 医学博士および理学修士とその研究スタッフ以外がこのアンケートを使用することは禁じられています。

質問

この調査では, お子さんの病気や気持ちについてうかがいます。お答えは正しいとか誤っているとかというものではありません。質問がわからなければ尋ねてください。何かコメントを記述されたい場合は、**5** ページまたは **6** ページの後ろを使ってください。

いろいろなことがお子さんの生活に影響を与え、お子さんの気持ちを楽しくしたり、悲しくしたりします。お子さん自身にとって重要だなと考えていることがいくつかあると思います。お子さんがそれを出来ることもあるでしょうし、出来ないこともあるでしょう。お子さんの生活の中で起こるこうしたこと全てが、総合的にお子さんの QOL (Quality of Life: 生活の質) となっています。

Copyright © 2007 Lakshmi Nandini Moorthy. 無断複製転載禁止。

1. お子さんの **QOL (**生活の質**)** はいかがですか？お子さんの **QOL** を最もあらわしている“顔”を囲んでください。

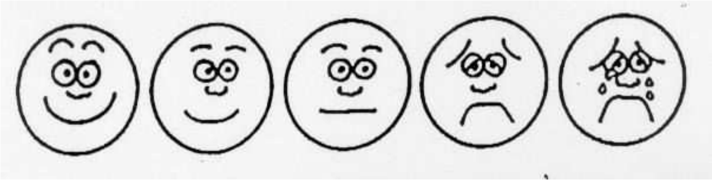

2. 現在のお子さんの病気 はいかがですか？お子さんの病気 の状態を最もよくあらわしている“顔”を囲んでください。

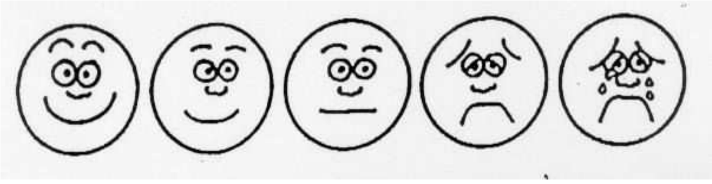

質問 **3-19** は、お子さんがこの **1** ヶ月、病気があるために、どのような気持ちで過ごしていたかに関するものです。お子さんの印象に最も合う“顔”を囲んで下さい。

3. 病気が原因で、お子さんはどのような気持ちでいますか？

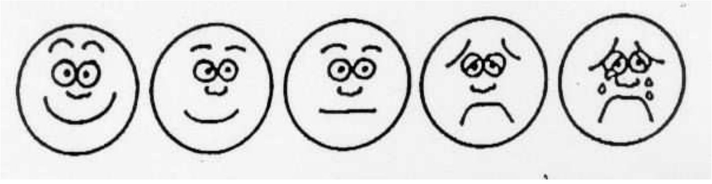

4. 病気があることで、学校に行くことについてお子さんはどのような気持ちになりますか？

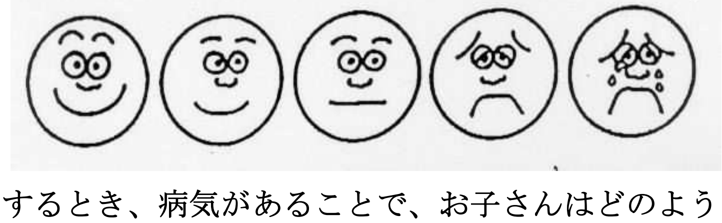

5. 毎日着替えをするとき、病気があることで、お子さんはどのような気持ちになりますか？

Copyright © 2007 Lakshmi Nandini Moorthy. 無断複製転載禁止。

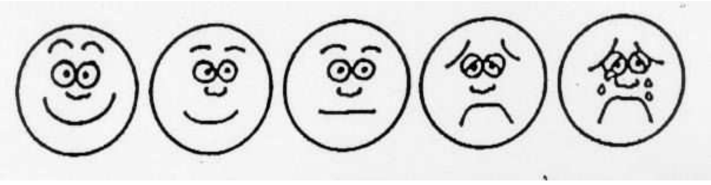

6. 病気があることで、お子さんが自分自身の外見に対してどのように感じていますか？

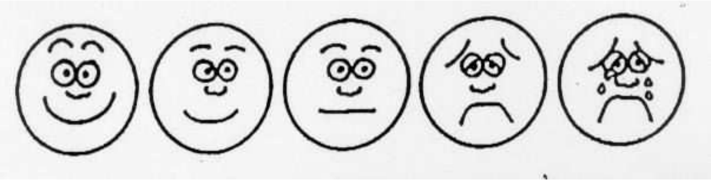

7. 病気があることで、お子さんは自分自身についてどのように感じていますか？

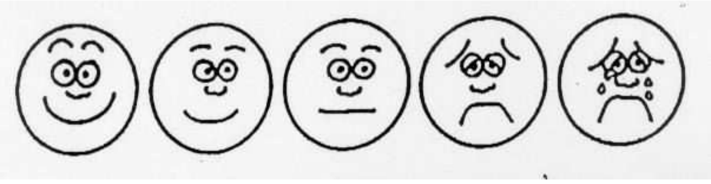

8. お子さんが、誰かに自分の病気について話さなければならないとき、お子さんはどのような気持ちになりますか？

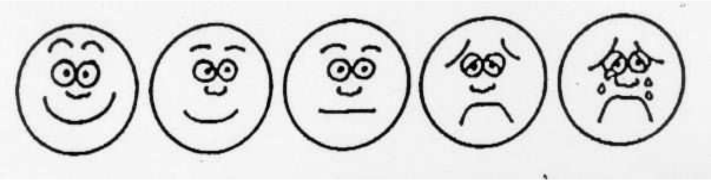

9. 病気のために医者の診察を受けることについて、お子さんはどのように感じていますか？

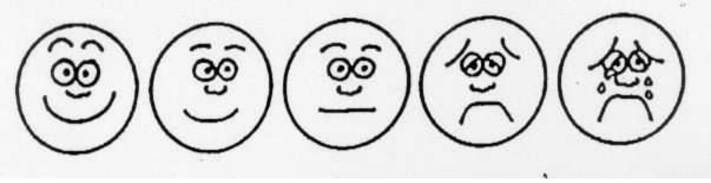

10. 病気のために病院に行くことを、お子さんはどのように感じていますか？

Copyright © 2007 Lakshmi Nandini Moorthy. 無断複製転載禁止。

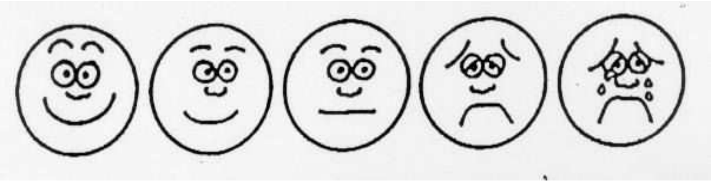

11. 病気があることで、お子さんは自分の将来についてどのように心配していますか？

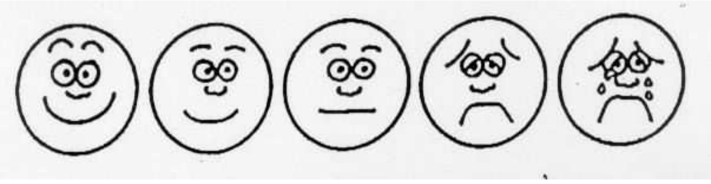

12. 病気のために薬をのむことを、お子さんはどのように感じていますか？

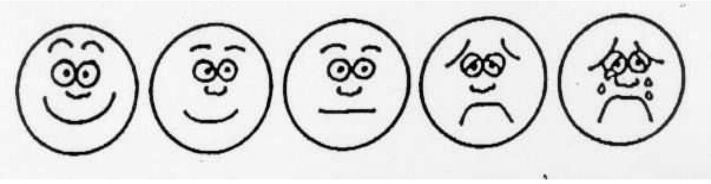

13. 病気のために飲む薬の副作用について、お子さんはどのくらい心配していますか？

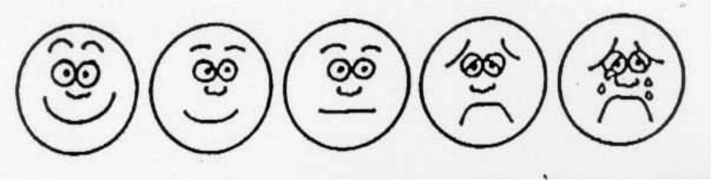

14. 病気が悪くなるかどうかについて、お子さんはどのくらい心配していますか？

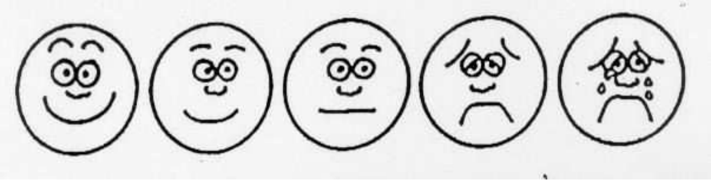

15. 病気のために、お子さんはどのくらい心細いさみしさを感じていますか？

Copyright © 2007 Lakshmi Nandini Moorthy. 無断複製転載禁止。

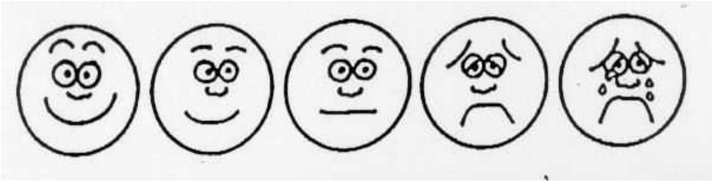

16. ご家族は、お子さんをどのような気持ちにさせますか？

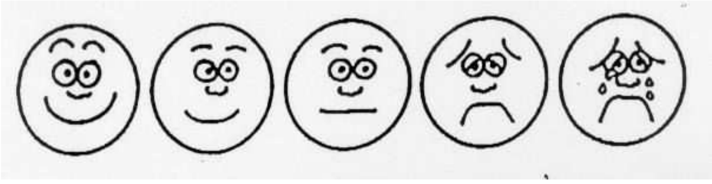

17. お子さんは、病気による痛みをどのように感じていますか？

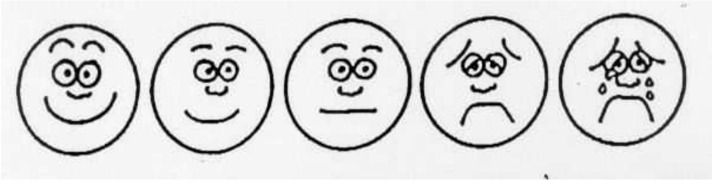

18. お子さんは、病気のために、日光が当たるところに行けないことをどのように感じていますか？

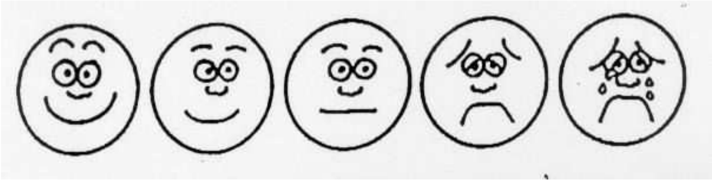

19. お子さんは、病気による疲れをどのように感じていますか**?**

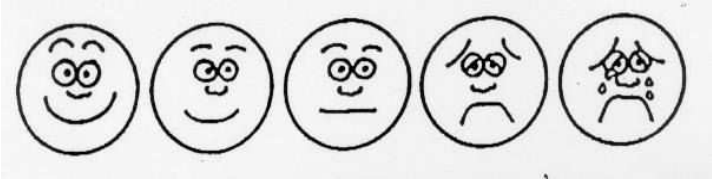

質問 **20 A- 26 A** については**,** まず**“**はい**”** か **“**いいえ**”** でお答え下さい。その答えにより、あとの指示に従ってください。

Copyright © 2007 Lakshmi Nandini Moorthy. 無断複製転載禁止。

20 A. お子さんの病気のせいで、お子さんの友人に変化がありましたか？
"はい" か "いいえ"を囲んでください。 はい　いいえ
"はい" の場合、20B の質問に答えてください。
20 B. 病気になったことで、お子さん自身の友人に対する気持ちはどうなりましたか？お子さんの気持ちを表す顔を囲んでください。

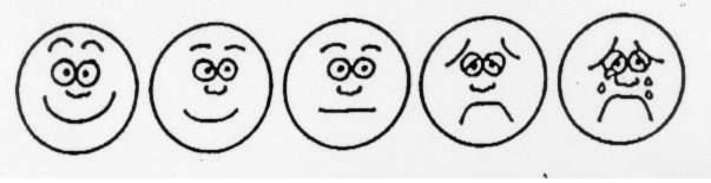

21 A. お子さんの友人は、お子さんが病気であることを理解していますか？
はい　いいえ
"いいえ" の場合、21B の質問に答えてください。
21 B. お子さんは、友人がお子さんの病気について理解していないことを、どう感じていますか。お子さんの気持ちを表す顔を囲んでください。

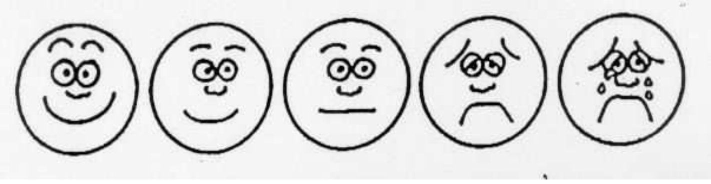

22 A. 病気はお子さんの学業に影響を与えていますか？ はい　いいえ

"はい" の場合、22B の質問に答えてください。
22B.　病気であることで、お子さんの学習に対する気持ちはそうなりましたか？ お子さんの気持ちを表す顔を囲んでください。

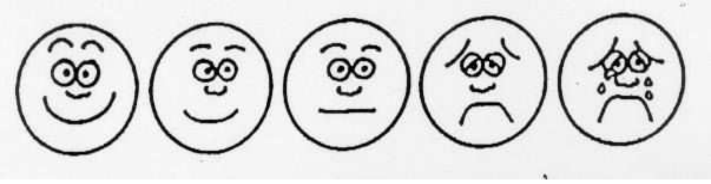

23 A. 病気のために、お子さんは学校を休みましたか？ はい　いいえ

Copyright © 2007 Lakshmi Nandini Moorthy. 無断複製転載禁止。

"はい" の場合、23B の質問に答えてください。

23 B. 病気のために学校を休むことについて、お子さんはどのように思っています か？ お子さんの気持ちを表す顔を囲んでください。

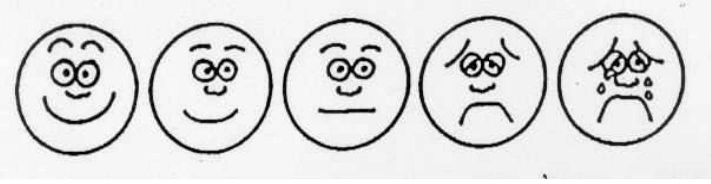

24 A. 病気のために、お子さんの学校以外の場所での活動に支障をきたしています か？

はい　いいえ

"はい" の場合、24B の質問に答えてください。

24 B.お子さんは、病気のために、学校以外の場所での活動に支障をきたすことについ てどう思っていますか？ お子さんの気持ちを表す顔を囲んでください。

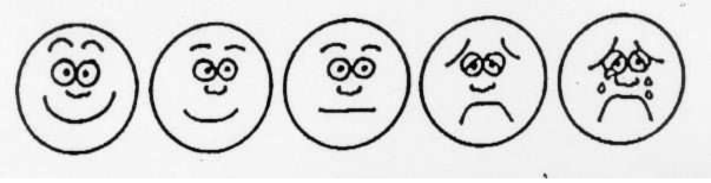

25 A. 病気のために、お子さんは友人との楽しい時間を過ごすことができなくなりま したか？

はい　いいえ

"はい" の場合、25B の質問に答えてください。

25 B. お子さんは、病気が自分と友人との交流に支障をきたしていることをどう思っ ていますか？お子さんの気持ちを表す顔を囲んでください。

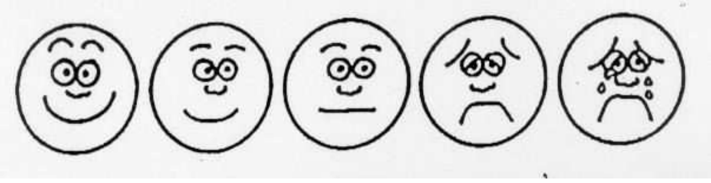

Copyright © 2007 Lakshmi Nandini Moorthy. 無断複製転載禁止。

26 A. 病気のために、お子さんの運動活動への参加（学校での体育、ランニング、ウォーキング、お気に入りの野外ゲームなど）に支障をきたしていますか？

はい　いいえ

“はい”の場合、26B の質問に答えてください。

26 B. お子さんは、病気によって、運動活動への参加（学校での体育、ランニング、ウォーキング、お気に入りの野外ゲームなど）に支障をきたしていることをどう思っていますか？お子さんの気持ちを表す顔を囲んでください。

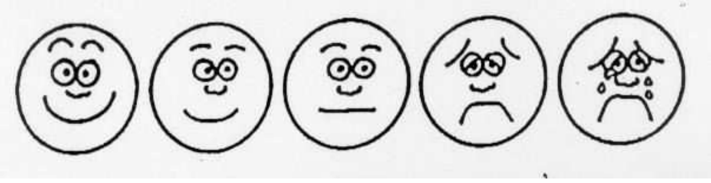

Copyright © 2007 Lakshmi Nandini Moorthy. 無断複製転載禁止。

子供における病気の影響に関する簡易評価 (SMILY-Illness)
子供用調査票 – Child Version

試験番号 __　日付 20__年__月__日

この調査では、“Wong-Baker FACES Pain Rating Scale”というフェイススケール（表情を用いた評価法）を D.L. Wong 氏の許可のもとに改変したものを使います。これらの 5 つの顔は、どの質問にも出てきます。

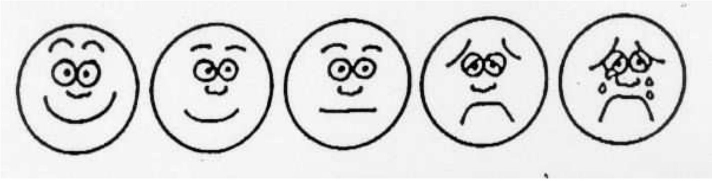

出典 Wong DL, Hockenberry-Eaton M, Wilson D, Winklestein ML, Schwartz P.著「Wong’s Essentials of Pediatric Nursing」第 6 版、米国セントルイス、2001 年、Mosby 社、p 1301
著作権 Mosby 社。転載許可取得。

スマイリー- 病気の著作権は、**L. Nandini Moorthy** 医学博士および理学修士（米国ニュージャージー州ニューブランズウィック市 **RWJMS-UMDNJ** 所属）に帰属します。現時点において、**L. Nandini Moorthy** 医学博士および理学修士とその研究スタッフ以外がこのアンケートを使用することは禁じられています。

質問

この調査では, あなたの病気や気持ちについてうかがいます。お答えは正しいとか誤っているとかというものではありません。質問がわからなければたずねてください。何かコメントを書きたい場合は、5 ページまたは 6 ページの後ろを使ってください。

いろいろなことがあなたの生活に影響を与え、あなたの気持ちを楽しくしたり、悲しくしたりします。あなたにとって重要だなと考えていることがいくつかあると思います。あなたがそれをできることもあるでしょうし、できないこともあるでしょう。あなたの生活の中

Copyright © 2007 Lakshmi Nandini Moorthy. 無断複製転載禁止。

で起こるこうしたこと全てが、総合的にあなたの QOL (Quality of Life: 生活の質)となっています。

1. あなたの **QOL (**生活の質**)** はいかがですか？あなたの **QOL** を最もよくあらわしている“顔”を囲んでください。

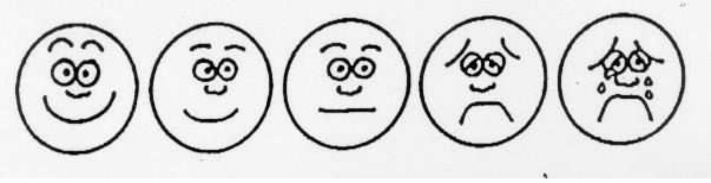

2. あなたの病気は今いかがですか？あなたの病気の状態を最もよくあらわしている“顔”を囲んでください。

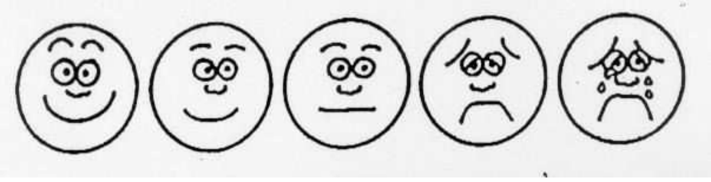

質問 **3-19** は、あなたがこの **1** ヶ月、病気があるために、どのような気持ちで過ごしていたかに関するものです。あなたの印象に最も合う“顔”を囲んで下さい。

3. 病気が原因で、あなたはどのような気持ちでいますか？

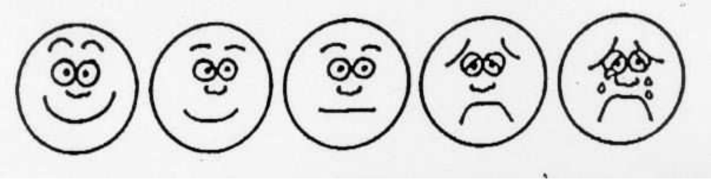

Copyright © 2007 Lakshmi Nandini Moorthy. 無断複製転載禁止。

4. 病気(びょうき)があることで、学校に行くことについてあなたはどのような気持ちになりますか？

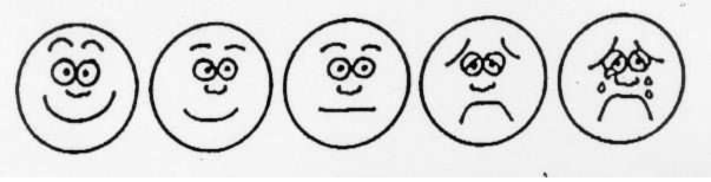

5. 毎日着替(きが)えをするとき、病気(びょうき) があることで、あなたはどのような気持ちになりますか？

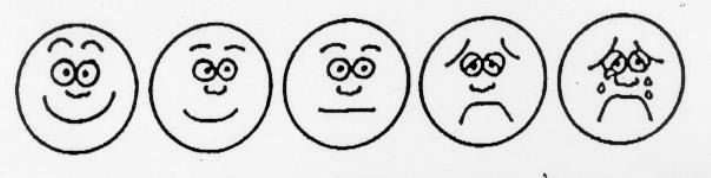

6. 病気(びょうき)があることで、自分自身(じぶんじしん)の外見(がいけん)に対してどのように感じていますか？

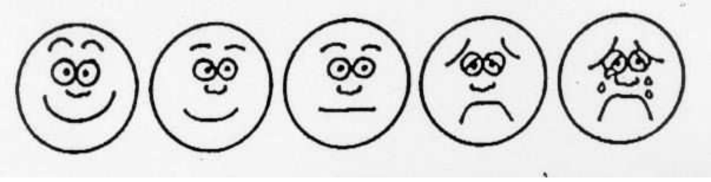

7. 病気(びょうき)があることで、あなたは自分自身(じぶんじしん)についてどのように感じていますか？

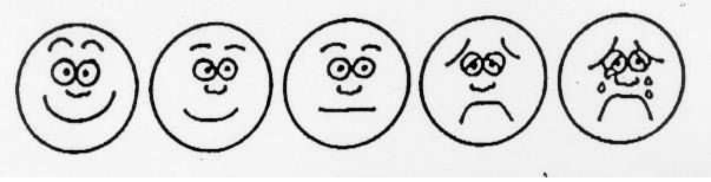

Copyright © 2007 Lakshmi Nandini Moorthy. 無断複製転載禁止。

8. あなたが、誰(だれ)かに自分(じぶん)の病気(びょうき)について話(はな)さなければならないとき、あなたはどのような気持ちになりますか？

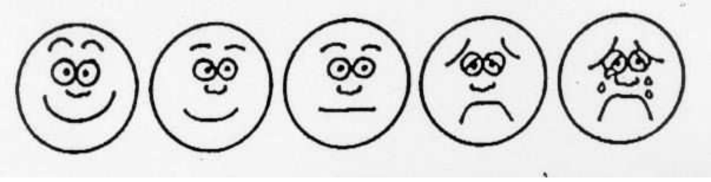

9. 病気(びょうき)のために医者(いしゃ)の診察(しんさつ)を受(う)けることについて、あなたはどのように感じていますか？

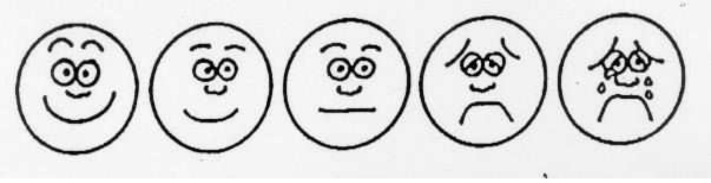

10. 病気(びょうき)のために病院(びょういん)に行くことを、あなたはどのように感じていますか？

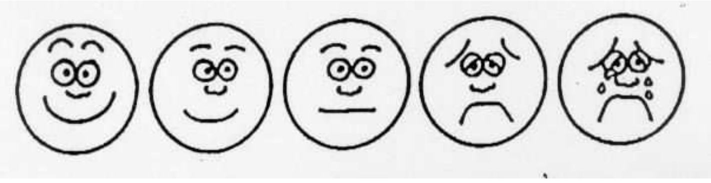

11. 病気(びょうき)があることで、あなたは自分の将来(しょうらい)についてどのくらい心配(しんぱい)していますか？

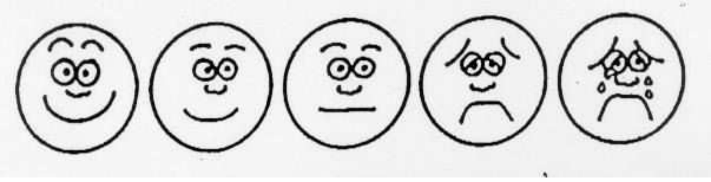

12. 病気(びょうき)のために薬(くすり)をのむことを、あなたはどのように感じていますか？

Copyright © 2007 Lakshmi Nandini Moorthy. 無断複製転載禁止。

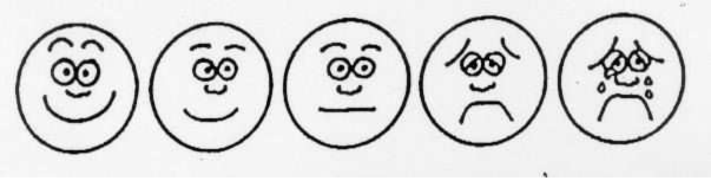

13. 病気(びょうき)のために飲む薬(くすり)の副作用(ふくさよう)について、あなたはどのくらい心配(しんぱい)していますか？

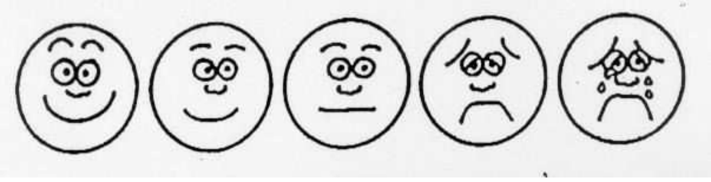

14. 病気(びょうき)が悪(わる)くなるかどうかについて、あなたはどのくらい心配(しんぱい)していますか？

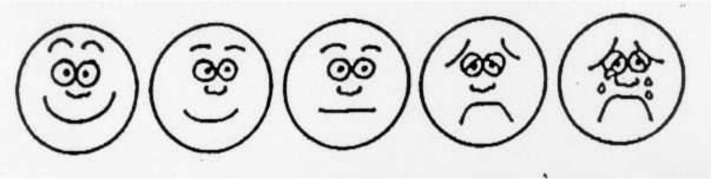

15. 病気(びょうき)のために、あなたはどのくらい心細(こころぼそ)いさみしさを感じていますか？

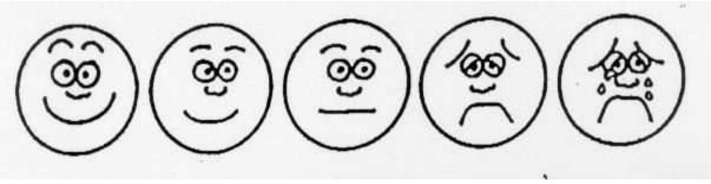

16. あなたのご家族(かぞく)は、あなたをどのような気持ちにさせますか？

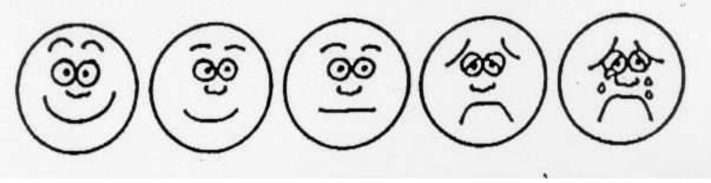

17. あなたは、病気(びょうき)による痛(いた)みをどのように感じていますか？

Copyright © 2007 Lakshmi Nandini Moorthy. 無断複製転載禁止。

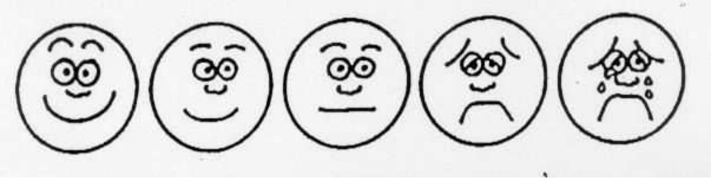

18. あなたは、病気のために、日光があたるところに行けないことをどのように感じていますか？

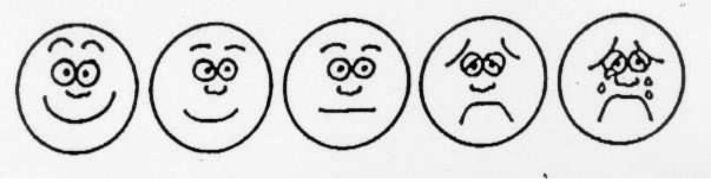

19. あなたは、病気による疲れをどのように感じていますか**?**

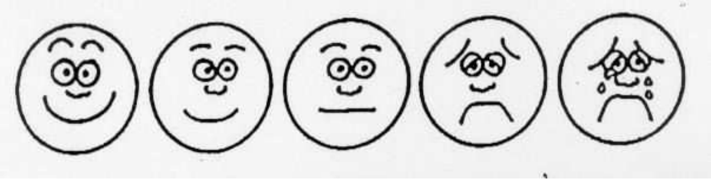

質問 **20 A- 26 A** については**,** まず**"**はい**"** か **"**いいえ**"** でお答え下さい。その答えにより、あとの指示に従ってください。

20 A. あなたの病気のせいで、友だちに変化がありましたか？

"はい**"** か **"**いいえ**"**を囲んでください。 はい いいえ

"はい" の場合、20B の質問に答えてください。

Copyright © 2007 Lakshmi Nandini Moorthy. 無断複製転載禁止。

20 B. 病気になったことで、あなた自身の友だちに対する気持ちはどうなりましたか？あなたの気持ちを表す顔を囲んでください。

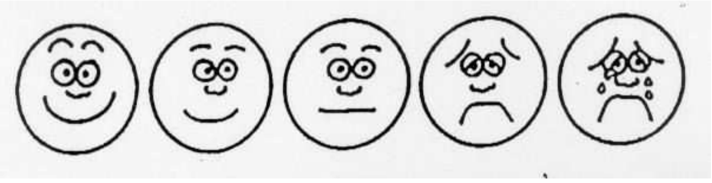

21 A. あなたの友だちは、あなたが病気であることを理解していますか？

はい　いいえ

“いいえ” の場合、21B の質問に答えてください。

21 B. あなたが病気であることについて友だちが理解していないことを、あなたはどう感じていますか。あなたの気持ちを表す顔を囲んでください。

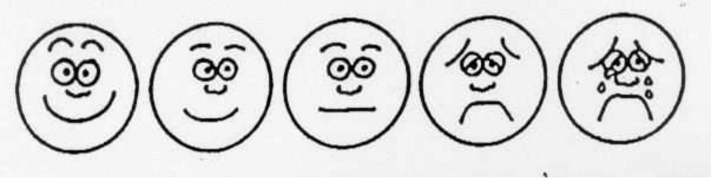

22 A. 病気はあなたの学業に影響を与えていますか？　　はい　いいえ

“はい” の場合、22B の質問に答えてください。

Copyright © 2007 Lakshmi Nandini Moorthy. 無断複製転載禁止。

22B.　病気があることで、あなたの学習に対する気持ちはどうなりましたか？ あなたの気持ちを表す顔を囲んでください。

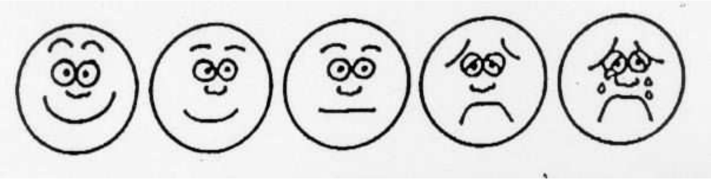

23 A. 病気のために、あなたは学校を休みましたか？

はい　いいえ

“はい” の場合、23B の質問に答えてください。

23 B. 病気のために学校を休むことについて、あなたはどのように思っていますか？ あなたの気持ちを表す顔を囲んでください。

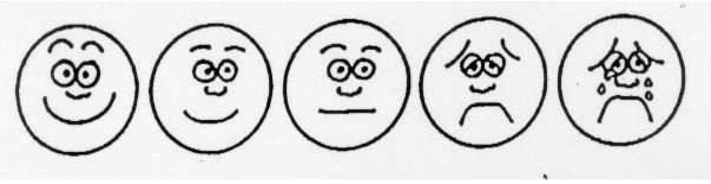

24 A. 病気のために、学校以外の場所での活動に支障をきたしていますか？

はい　いいえ

“はい” の場合、24B の質問に答えてください。

Copyright © 2007 Lakshmi Nandini Moorthy. 無断複製転載禁止。

24 B.あなたは、病気のために、学校以外の場所での活動に支障をきたすことについてどう思っていますか？ あなたの気持ちを表す顔を囲んでください。

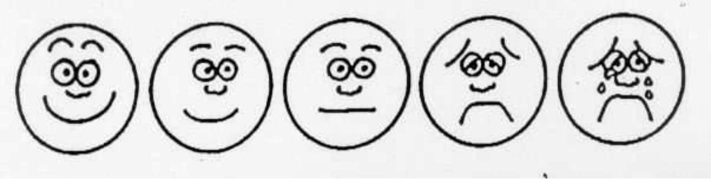

25 A. 病気のために、友だちとの楽しい時間を過ごすことができなくなりましたか？

はい　いいえ

“はい” の場合、25B の質問に答えてください。

25 B. あなたは、病気が自分と友だちとの交流に支障をきたしていることをどう思っていますか？あなたの気持ちを表す顔を囲んでください。

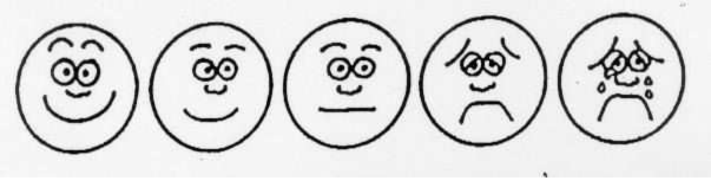

26 A. 病気のために、運動活動への参加（学校での体育、ランニング、ウォーキング、お気に入りの野外ゲームなど）に支障をきたしていますか？

はい　いいえ

“はい” の場合、26B の質問に答えてください。

Copyright © 2007 Lakshmi Nandini Moorthy. 無断複製転載禁止。

26 B. 病気によって、運動活動への参加（学校での体育、ランニング、ウォーキング、お気に入りの野外ゲームなど）に支障をきたしていることを、あなたはどう思っていますか？あなたの気持ちを表す顔を囲んでください。

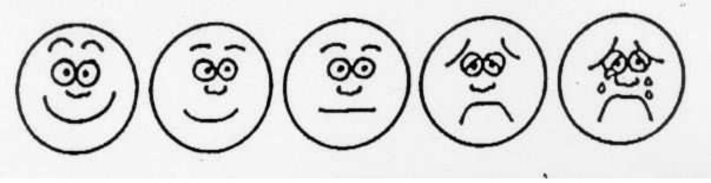

Copyright © 2007 Lakshmi Nandini Moorthy. 無断複製転載禁止。

SIMPLE MEASURE OF IMPACT OF ILLNESS IN YOUNGSTERS (SMILY-Illness) – Relatório da criança – Child Version

Número do estudo __ Data __/__/20__

Para este questionário, adotamos as 5 faces abaixo, da Escala de intensidade da dor FACES de Wong-Baker, modificadas com a permissão de D.L. Wong. Essas 5 faces aparecerão abaixo de todas as perguntas.

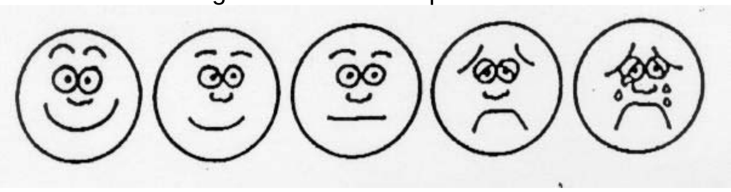

De Wong DL, Hockenberry-Eaton M, Wilson D,Winklestein ML, Schwartz P.: Wong's Essentials of Pediatric Nursing, ed. 6, St. Louis, 2001, Mosby, p 1301. Copyright Mosby. Reproduzido com permissão.

The SMILY-illness está protegido por direitos autorais para L. Nandini Moorthy, MD MS, RWJMS-UMDNJ, New Brunswick, NJ. Neste momento, o questionário só pode ser usado por L. Nandini Moorthy, MD MS e sua equipe de pesquisa designada.

PERGUNTAS

Neste questionário, faremos muitas perguntas sobre a sua doença e os seus sentimentos. Não existem respostas erradas. Pergunte-nos se não entender alguma coisa. Se desejar escrever algum comentário, faça-o no verso das páginas 5 e 6.

Coisas diferentes afetam sua vida e fazem você se sentir alegre ou triste. Existem algumas coisas que você acha importantes, que consegue fazer (ou não consegue fazer). Todas essas coisas diferentes de sua vida juntas compõem sua qualidade de vida.

1. Como está sua qualidade de vida? Assinale (√) a face que melhor represente sua qualidade de vida.

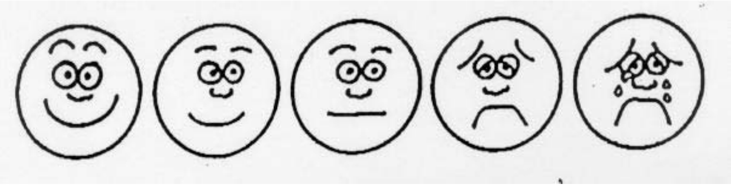

2. Como está sua doença agora? Assinale (√) a face que melhor represente como está a sua doença.

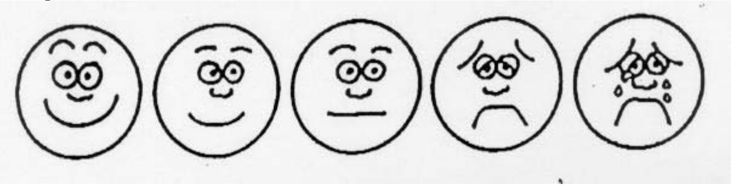

Copyright © 2007 Lakshmi Nandini Moorthy. Todos os direitos reservados.

As perguntas de 3 a 19 são sobre como você se sentiu no último mês por causa da doença. Assinale (√) a face que melhor represente como você se sente.

3. Como você se sente por ter uma doença?

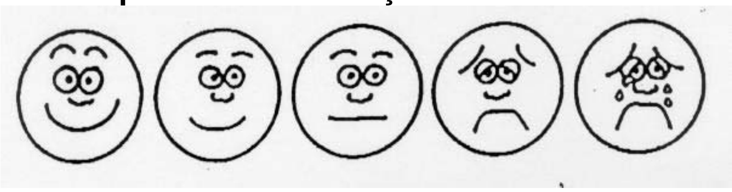

4. Como você se sente sobre ir à escola tendo uma doença?

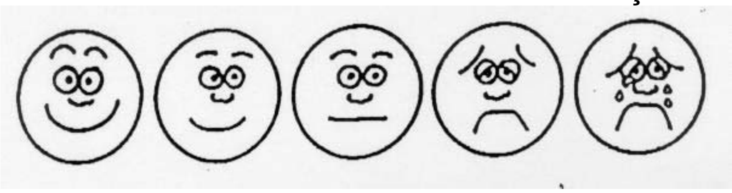

5. Como você se sente sobre ter que se vestir todos os dias tendo uma doença?

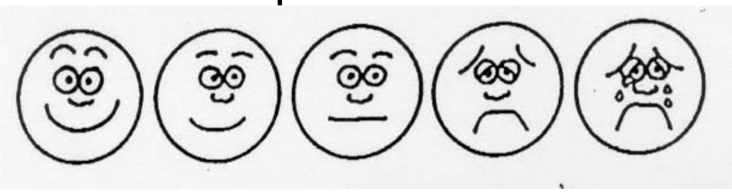

6. Como você se sente sobre sua aparência tendo uma doença?

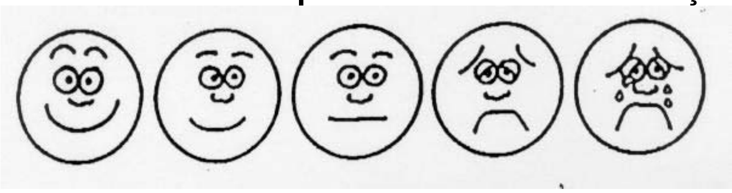

7. Como você se sente em relação a si mesmo tendo uma doença?

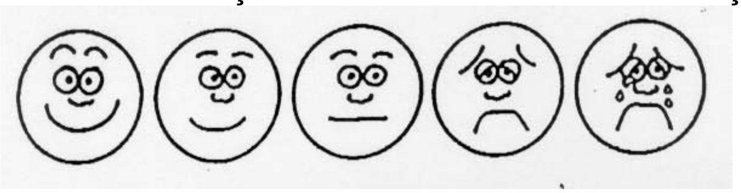

8. Como você se sente quando precisa falar sobre a sua doença?

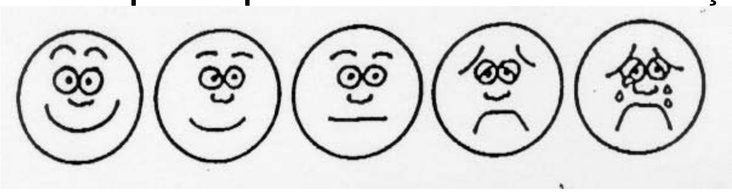

Copyright © 2007 Lakshmi Nandini Moorthy. Todos os direitos reservados.

9. Como você se sente por ter que ir ao médico por causa da doença?

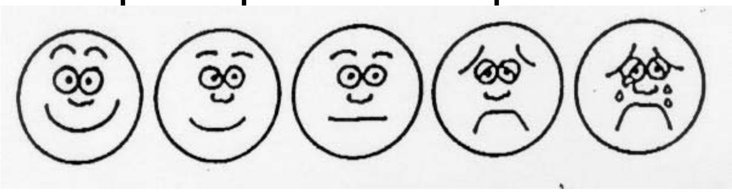

10. Como você se sente por ter que ir ao hospital por causa da doença?

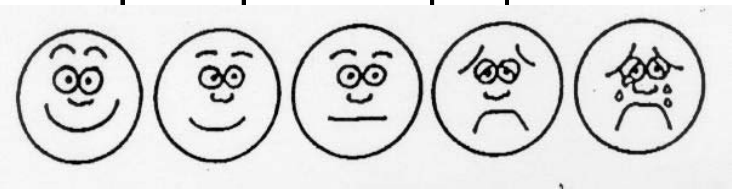

11. Como você se sente em relação ao futuro por ter uma doença?

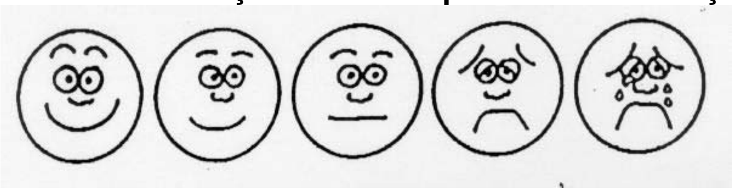

12. Como você se sente sobre tomar remédios para a doença?

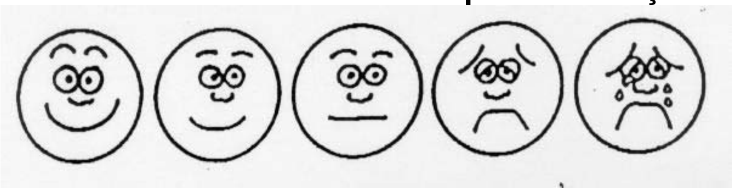

13. O quanto você está preocupado em relação aos efeitos colaterais dos medicamentos que toma para a doença?

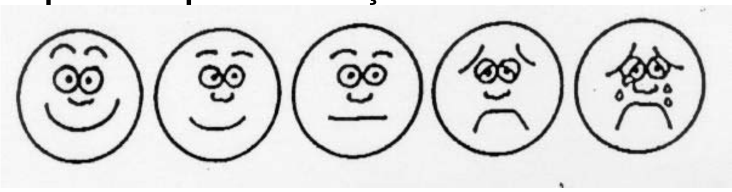

14. O quanto você está preocupado que a doença piore?

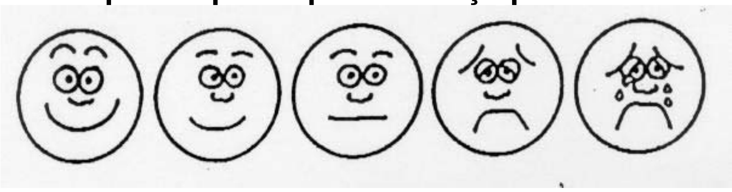

Copyright © 2007 Lakshmi Nandini Moorthy. Todos os direitos reservados.

15. O quanto você se sente sozinho por causa da doença?

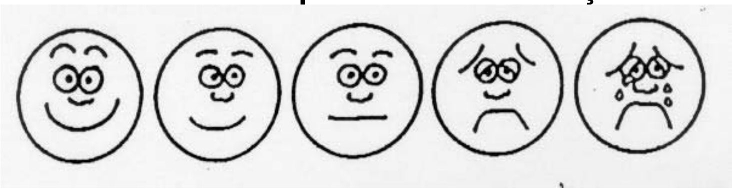

16. Como sua família faz você se sentir?

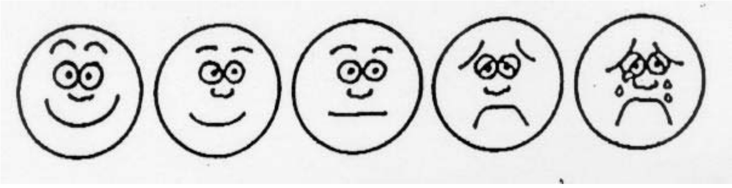

17. Como a dor da doença faz você se sentir?

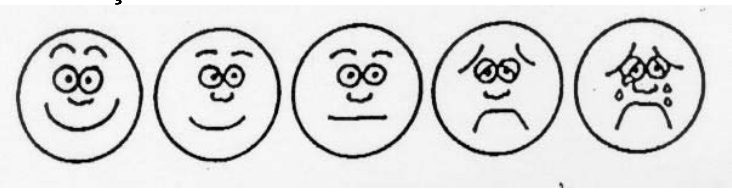

18. Como você se sente por não poder se expor ao sol por causa da doença?

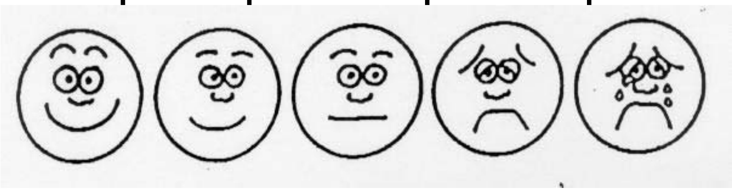

19. O quanto você se sente cansado por causa da doença?

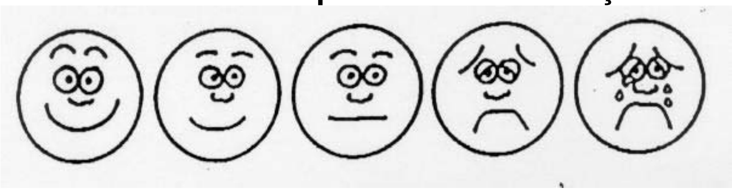

Copyright © 2007 Lakshmi Nandini Moorthy. Todos os direitos reservados.

Para as perguntas de 20 A - 26 A, responda “sim” ou “não.” Em seguida, siga as instruções.

20 A. Meus amigos mudaram por causa da minha doença. Sim Não

Se "sim", vá para 20-B.
20 B. Como você se sente em relação a seus amigos tendo uma doença?
Assinale a face que melhor represente como você se sente.

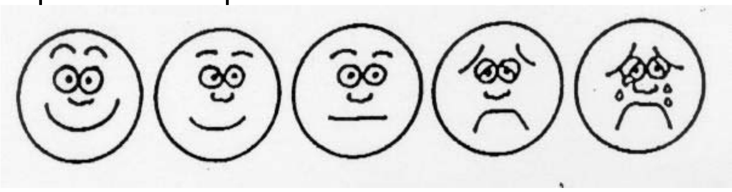

21 A. Meus amigos compreendem que eu tenho uma doença. Sim Não
Se tiver respondido "não", vá para a pergunta 21 B.
21 B. Como você se sente pelo fato dos seus amigos não compreenderem que você tem uma doença? Assinale a face que melhor represente como você se sente.

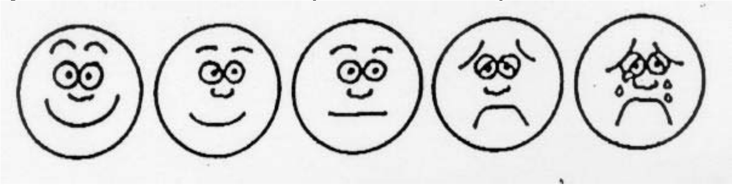

22 A. A doença afeta minhas tarefas escolares. Sim Não
Se "sim", vá para 22-B.
22 B. Como você se sente em relação às suas tarefas escolares tendo uma doença?
Assinale a face que melhor represente como você se sente.

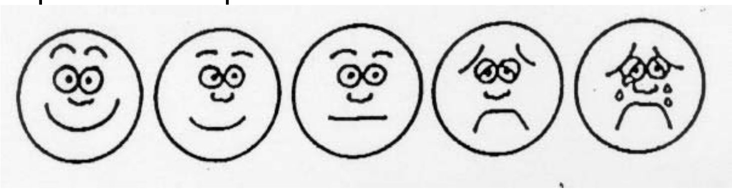

23 A. Eu perco aulas por causa da doença. Sim Não
Se "sim", vá para a pergunta 23-B.
23 B. Como você se sente por perder aula por causa da doença?
Assinale a face que melhor represente como você se sente.

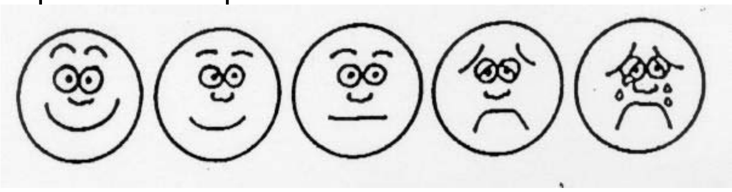

Copyright © 2007 Lakshmi Nandini Moorthy. Todos os direitos reservados.

24 A. A doença afeta minhas atividades fora da escola. Sim Não

Se tiver respondido "sim", vá para a pergunta 24 B.

24 B. Como você se sente pelo fato da doença afetar suas atividades fora da escola? Assinale a face que melhor represente como você se sente.

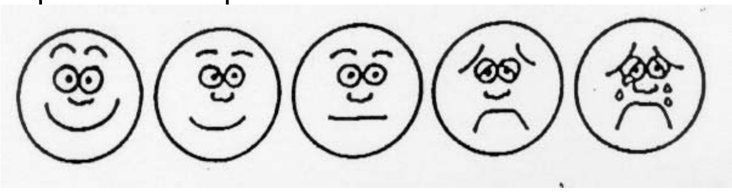

25 A. A doença afeta as diversões com meus amigos. Sim Não

Se tiver respondido "sim", vá para a pergunta 25 B.

25 B. Como você se sente sobre a doença afetar as diversões com seus amigos? Assinale a face que melhor represente como você se sente.

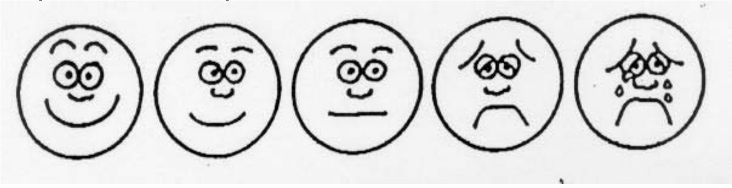

26 A. A doença afeta minha participação nas atividades físicas (como as atividades de ginástica na escola, corrida, caminhadas, um jogo favorito ao ar livre).

Sim Não

Se tiver respondido "sim", vá para a pergunta 26 B.

26 B. Como você se sente pelo fato de a doença afetar sua participação em atividades físicas (como as atividades de ginástica na escola, corrida, caminhadas, um jogo favorito ao ar livre). Assinale a face que melhor represente como você se sente.

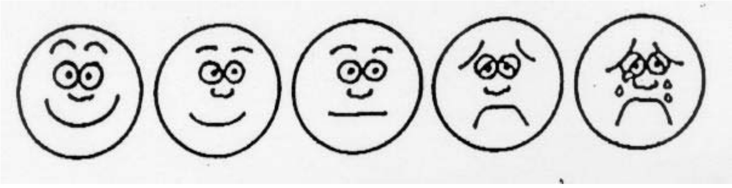

Copyright © 2007 Lakshmi Nandini Moorthy. Todos os direitos reservados.

SIMPLE MEASURE OF IMPACT OF ILLNESS IN YOUNGSTERS
(SMILY-Illness) Relatório dos pais – Parent Version

Número do estudo __ Data __/__/20_

Para este questionário, adotamos as 5 faces abaixo, da Escala de intensidade da dor FACES de Wong-Baker, modificadas com a permissão de D.L. Wong. Essas 5 faces aparecerão abaixo de todas as perguntas.

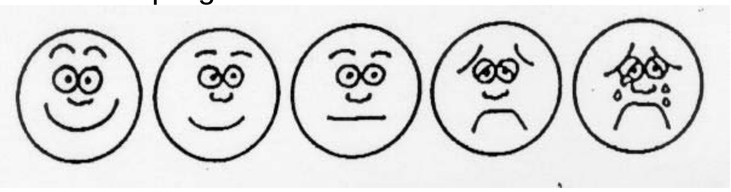

De Wong DL, Hockenberry-Eaton M, Wilson D,Winklestein ML, Schwartz P.: Wong's Essentials of Pediatric Nursing, ed. 6, St. Louis, 2001, Mosby, p 1301. Copyright Mosby. Reimpresso com permissão.

The SMILY-illness está protegido por direitos autorais para L. Nandini Moorthy, MD MS, RWJMS-UMDNJ, New Brunswick, NJ. Neste momento, o questionário só poderá ser usado por L. Nandini Moorthy, MD MS e sua equipe de pesquisa designada.

PERGUNTAS

Neste questionário, faremos muitas perguntas sobre a doença e os sentimentos do(a) seu(sua) filho(a). Não existem respostas erradas. Pergunte-nos se não entender alguma coisa. Se quiser escrever algum comentário, use o verso das páginas 5 e 6.

Coisas diferentes afetam a vida de seu(sua) filho(a) fazendo com que ele(a) se sinta alegre ou triste. Há algumas coisas que seu(sua) filho(a) pensa que são importantes para ele(a), que ele(a) tem ou não condições de fazer. Todas essas coisas diferentes na vida de seu(sua) filho(a) juntas compõem a qualidade de vida dele(a).

1. **Como é a qualidade de vida de seu(sua) filho(a)? Assinale (√) a face que melhor represente a qualidade de vida de seu(sua) filho(a).**

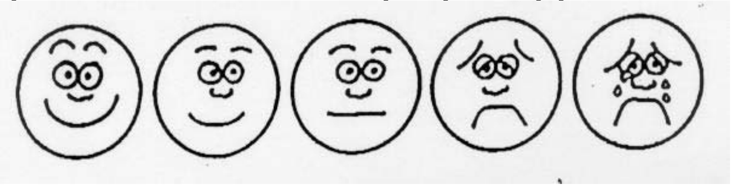

2. **Como está a doença de seu(sua) filho(a) agora? Assinale (√) a face que melhor represente como está a doença de seu(sua) filho(a).**

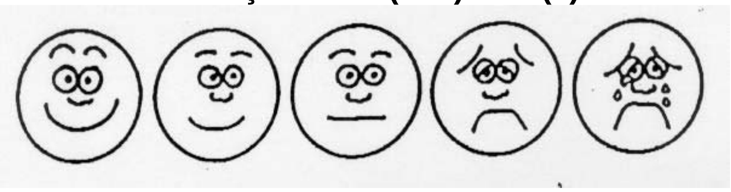

Copyright © 2007 Lakshmi Nandini Moorthy. Todos os direitos reservados.

As perguntas de 3 a 19 são sobre como seu(sua) filho(a) se sentiu no último mês por causa da doença. Assinale (√) a face que melhor represente sua resposta.

3. Como seu(sua) filho(a) se sente por ter uma doença?

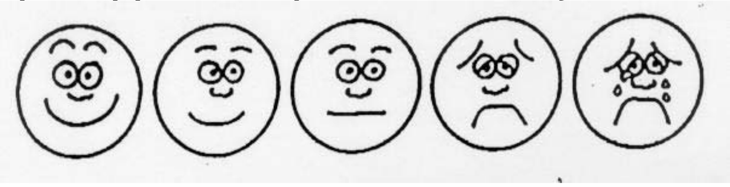

4. Como seu(sua) filho(a) se sente sobre ir à escola tendo uma doença?

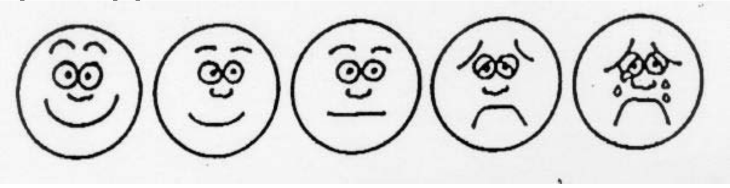

5. Como seu(sua) filho(a) se sente sobre se vestir todos os dias tendo uma doença?

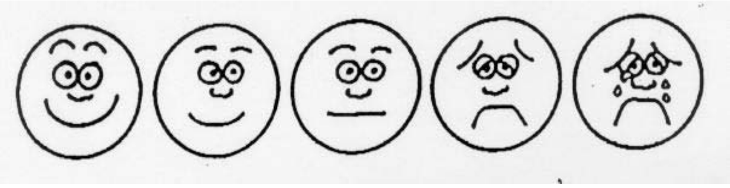

6. Como seu(sua) filho(a) se sente sobre sua aparência tendo uma doença?

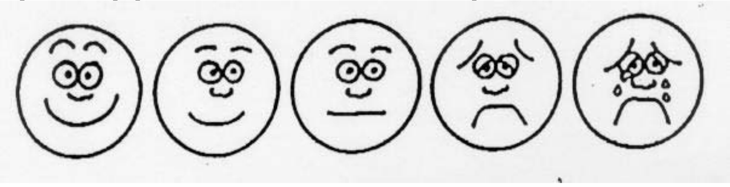

7. Como seu(sua) filho(a) se sente sobre si mesmo tendo uma doença?

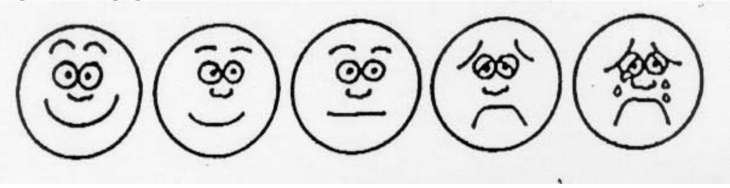

8. Como seu(sua) filho(a) se sente quando precisa falar sobre a sua doença?

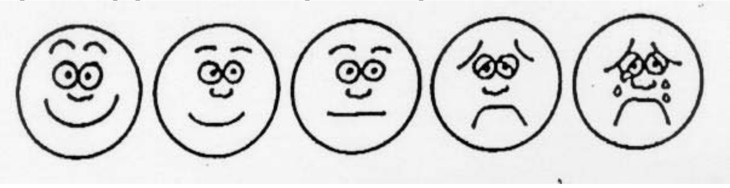

Copyright © 2007 Lakshmi Nandini Moorthy. Todos os direitos reservados.

9. Como seu(sua) filho(a) se sente por ter que ir ao médico por causa de sua doença?

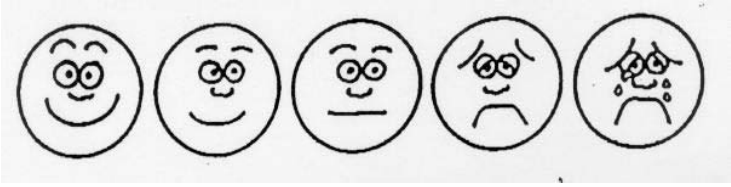

10. Como seu(sua) filho(a) se sente por ter que ir ao hospital por causa de sua doença?

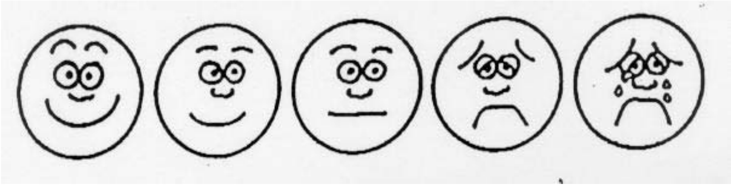

11. Como seu(sua) filho(a) se sente sobre seu futuro tendo uma doença?

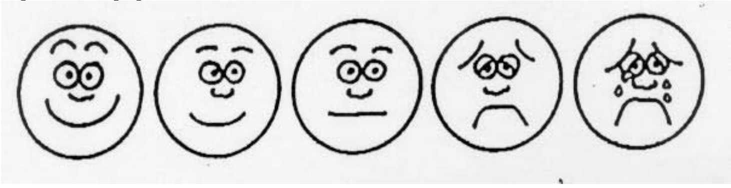

12. Como seu(sua) filho(a) se sente sobre tomar remédios para a doença?

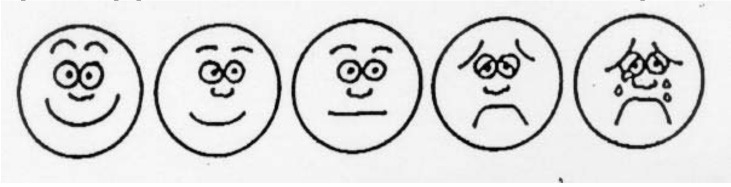

13. O quanto seu(sua) filho(a) está preocupado(a) sobre os efeitos colaterais do medicamento que ele(a) toma para a doença?

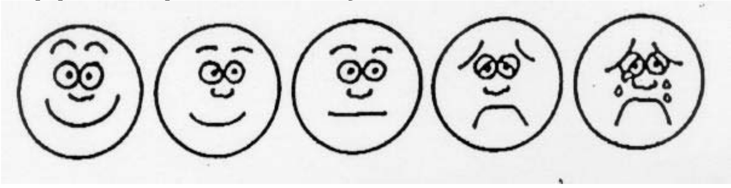

Copyright © 2007 Lakshmi Nandini Moorthy. Todos os direitos reservados.

14. O quanto seu(sua) filho(a) está preocupado(a) que a doença piore?

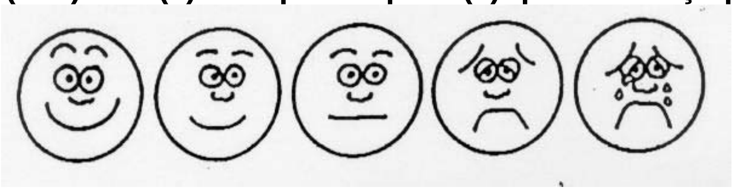

15. O quanto seu(sua) filho(a) se sente só por causa da doença?

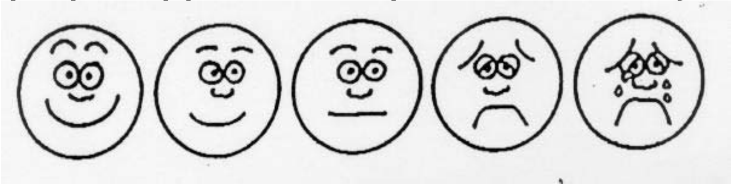

16. Como a família de seu(sua) filho(a) faz com que ele(a) se sinta?

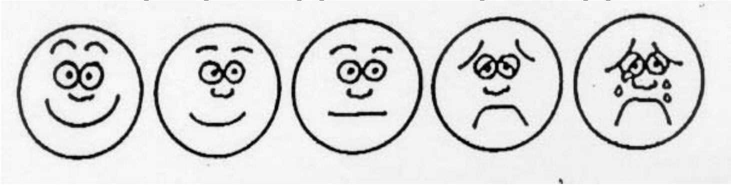

17. Como a dor da doença faz com que seu(sua) filho(a) se sinta?

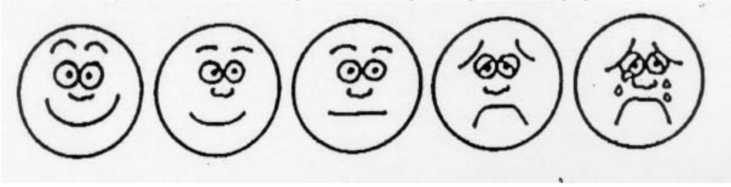

18. Como seu(sua) filho(a) se sente por não poder se expor ao sol por causa da doença?

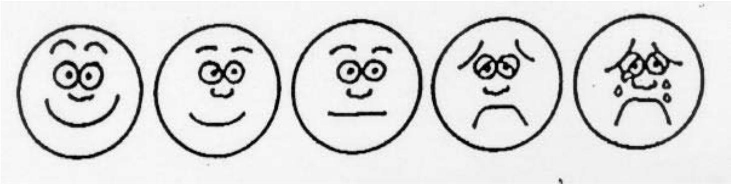

19. Quanto a doença faz seu(sua) filho(a) se sentir cansado(a)?

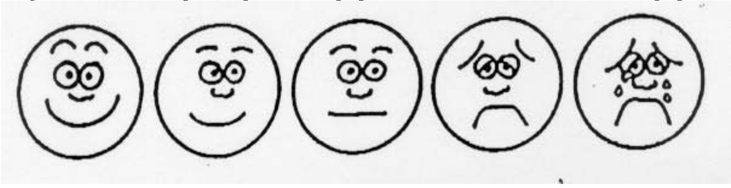

Copyright © 2007 Lakshmi Nandini Moorthy. Todos os direitos reservados.

Para as perguntas de 20 A - 26 A, responda "sim" ou "não." Em seguida, siga as instruções.

20 A Os amigos de seu(sua) filho(a) mudaram por causa de sua doença. Sim Não

Se "sim", vá para 20-B.

20 B. Como seu(sua) filho(a) se sente em relação a seus amigos tendo uma doença? Assinale a face que melhor represente como seu(sua) filho(a) se sente.

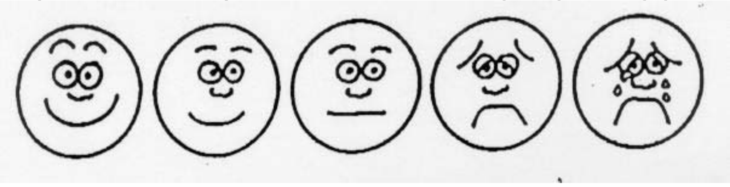

21 A. Os amigos de seu(sua) filho(a) compreendem que ele(a) tem uma doença.

Se tiver respondido "não", vá para a pergunta 21 B. **Sim Não**

21 B. Como seu(sua) filho(a) se sente porque seus amigos não compreendem que ele(a) tem uma doença? Assinale a face que melhor represente como seu(sua) filho(a) se sente.

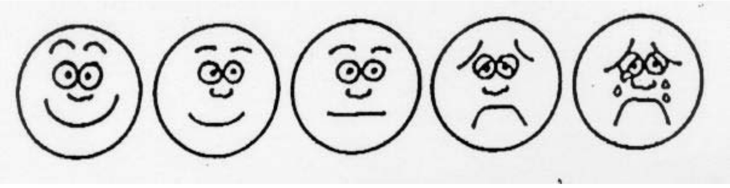

22 A. A doença afeta as atividades escolares de seu(sua) filho(a). Sim Não

Se "sim", vá para 22-B.

22 B. Como a doença faz seu(sua) filho(a) se sentir em relação às atividades escolares?

Assinale a face que melhor represente como seu(sua) filho(a) se sente.

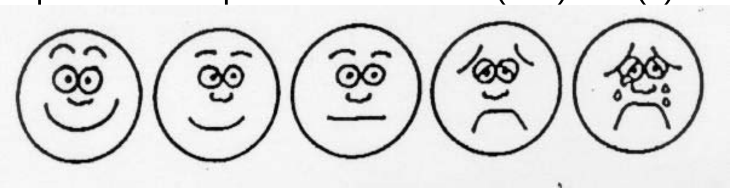

23 A. Seu(sua) filho(a) perde aulas por causa da doença. Sim Não

Se "sim", vá para a pergunta 23-B.

23 B. Como seu(sua) filho(a) se sente por perder aulas por causa da doença? Assinale a face que melhor represente como seu(sua) filho(a) se sente.

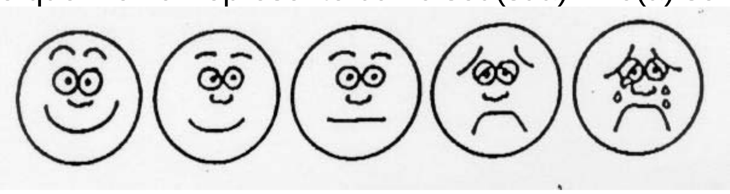

Copyright © 2007 Lakshmi Nandini Moorthy. Todos os direitos reservados.

24 A. A doença afeta as atividades de seu(sua) filho(a) fora da escola. Sim Não

Se tiver respondido "sim", vá para a pergunta 24 B.

24 B. Como seu(sua) filho(a) se sente pelo fato de a doença afetar suas atividades fora da escola? Assinale a face que melhor represente como seu(sua) filho(a) se sente.

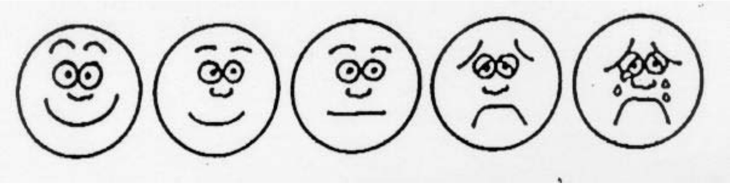

25 A. A doença afeta a diversão de seu(sua) filho(a) com seus amigos. Sim Não

Se tiver respondido "sim", vá para a pergunta 25 B.

25 B. Como seu(sua) filho(a) se sente sobre a doença afetar sua diversão com os amigos? Assinale a face que melhor represente como seu(sua) filho(a) se sente.

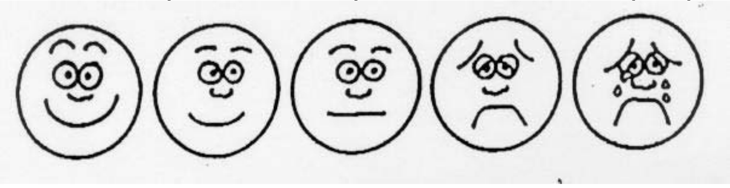

26 A. A doença afeta a participação do seu filho(a) em atividades físicas (como as atividades de ginástica na escola, corrida, caminhadas, um jogo favorito ao ar livre).

Sim Não

Se tiver respondido "sim", vá para a pergunta 26 B.

26 B. Como seu(sua) filho(a) se sente pelo fato da doença afetar sua participação em atividades físicas (como as atividades de ginástica na escola, corrida, caminhadas, um jogo favorito ao ar livre). Assinale a face que melhor represente como seu(sua) filho(a) se sente.

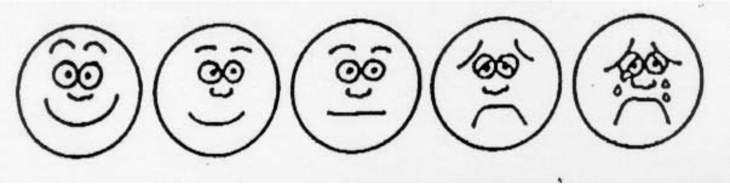

Copyright © 2007 Lakshmi Nandini Moorthy. Todos os direitos reservados.

MĂSURAREA SIMPLĂ A IMPACTULUI BOLII LA TINERI (SMILY-Boală) – Versiunea pentru părinte

Romania

Numărul studiului __ Data __/__/20_

Pentru acest chestionar, am preluat cele 5 figuri de mai jos din Scala Wong-Baker pentru Cuantificarea Durerii (FACES), pe care le-am modificat cu permisiunea lui D.L. Wong. Aceste 5 figuri vor apărea sub fiecare întrebare.

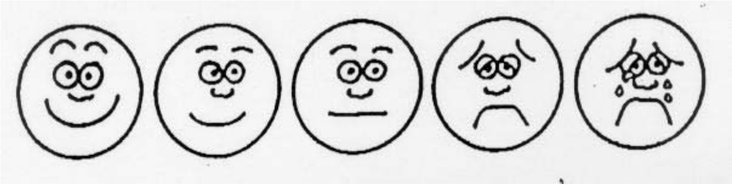

Din Wong DL, Hockenberry-Eaton M, Wilson D,Winkelstein ML, Schwartz P.: Wong's Essentials of Pediatric Nursing, ed. 6, St. Louis, 2001, Mosby, p 1301. Copyright Mosby. Reprodus cu acord.

SMILY- boală este proprietatea lui L. Nandini Moorthy, MD MS, RWJMS-UMDNJ, New Brunswick, NJ. În prezent, chestionarul poate fi utilizat doar de L. Nandini Moorthy, MD MS şi de personalul din echipa sa de cercetare.

ÎNTREBĂRI

În acest chestionar, vă vom adresa mai multe întrebări despre boala copilului dumneavoastră şi despre felul cum percepe el această boală. Nu există răspunsuri greşite. Dacă ceva este neclar vă rugăm să ne cereți explicații. Dacă doriți să scrieți unele comentarii, vă rugăm să utilizați verso paginilor 5 şi 6 .

Diferite lucruri afectează viața copilului dumneavoastră, făcându-l vesel sau trist. Există câteva lucruri pe care copilul dumneavoastră le consideră importante pentru el/ea şi pe care el/ea poate (sau nu poate) să le facă. Toate aceste lucruri diferite contribuie la calitatea vieții copilului dumneavoastră.

1. **Care este calitatea vieții copilului dumneavoastră? Vă rugăm să încercuiți figura care indică cel mai bine calitatea vieții copilului dumneavoastră.**

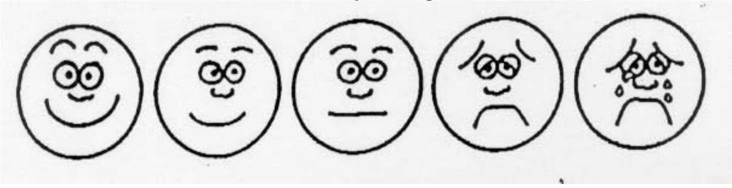

2. **Care este evoluția bolii copilului dumneavoastră în acest moment? Vă rugăm să încercuiți figura care indică cel mai bine evoluția de moment a bolii copilului dumneavoastră.**

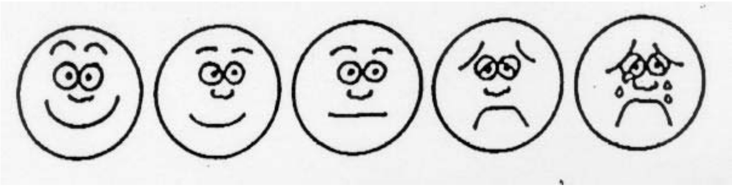

Copyright © 2007 Lakshmi Nandini Moorthy. Toate drepturile rezervate.

Întrebările 3-19 se referă la modul în care s-a simțit copilul dumneavoastră în ultima lună datorită bolii sale. Vă rugăm să încercuiți figura care indică cel mai bine răspunsul dumneavoastră.

3. Ce simte copilul dumneavoastră despre faptul că suferă de această boală?

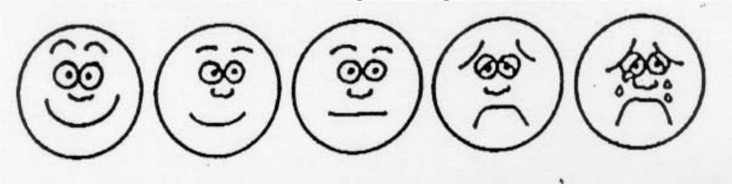

4. Cum influențează boala mersul la şcoală al copilului dumneavoastră?

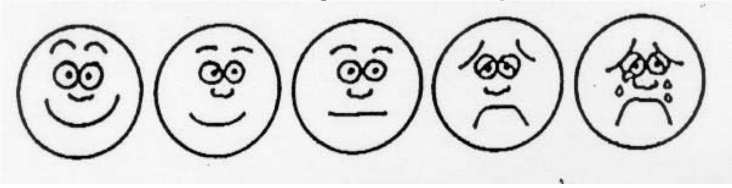

5. Cum influențează boala îmbrăcatul zilnic al copilului dumneavoastră?

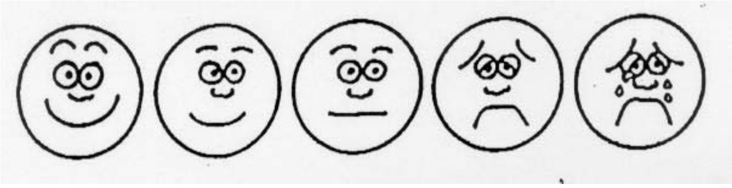

6. Cum influențează boala felul în care arată copilul dumneavoastră?

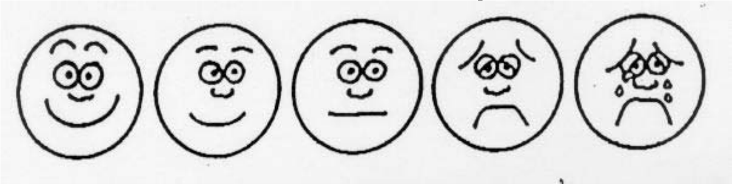

7. Cum influențează boala felul în care copilul se vede pe sine însuşi?

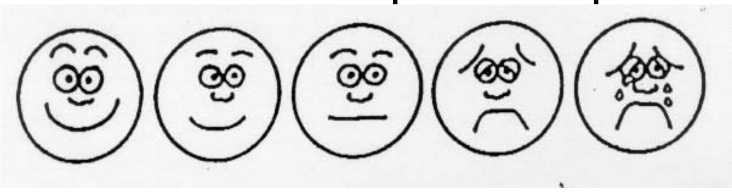

8. Cum se simte copilul dumneavoastră când trebuie să vorbească despre boala sa?

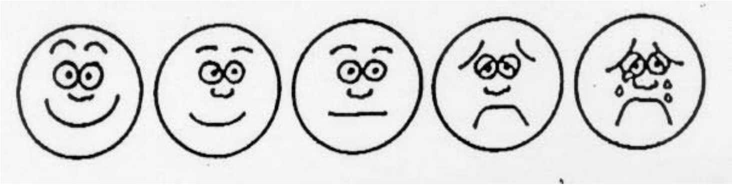

Copyright © 2007 Lakshmi Nandini Moorthy. Toate drepturile rezervate.

9. Ce simte copilul dumneavoastră când trebuie să meargă la medic din cauza bolii sale?

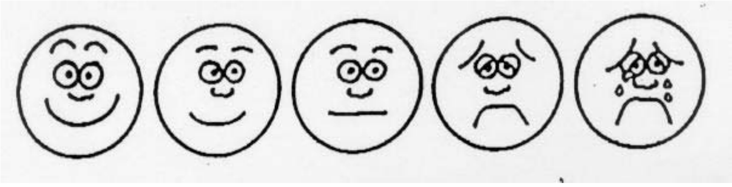

10. Ce simte copilul dumneavoastră când trebuie să meargă la spital din cauza bolii sale?

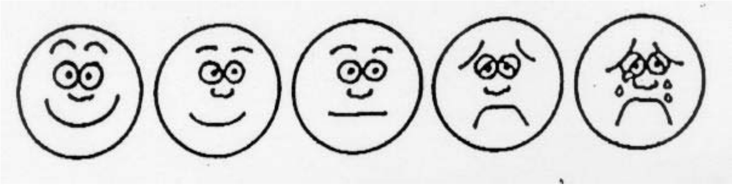

11. Cum îi influențează boala perspectiva asupra viitorului?

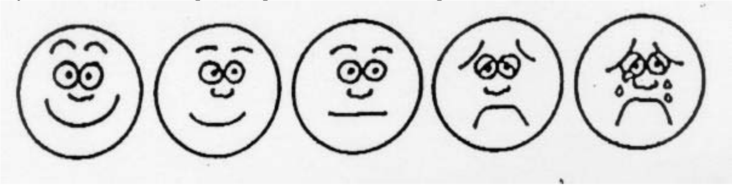

12. Ce crede copilul dumneavoastră despre faptul că trebuie să ia medicamente pentru boala sa?

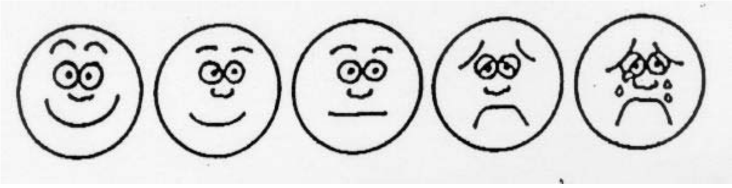

13. Cât de îngrijorat este copilul dumneavoastră despre efectele secundare ale medicației pe care o ia pentru boala sa?

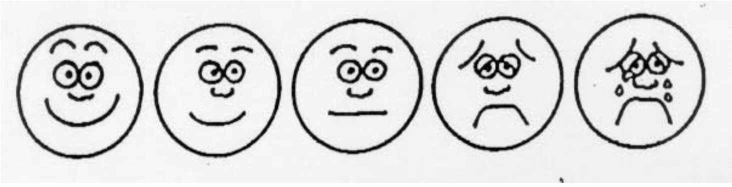

14. Cât de îngrijorat este copilul dumneavoastră despre agravarea bolii sale?

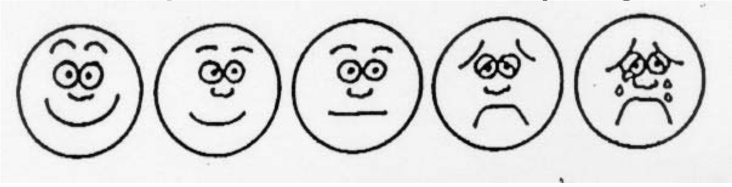

Copyright © 2007 Lakshmi Nandini Moorthy. Toate drepturile rezervate.

15. Cât de singur se simte copilul dumneavoastră datorită bolii sale?

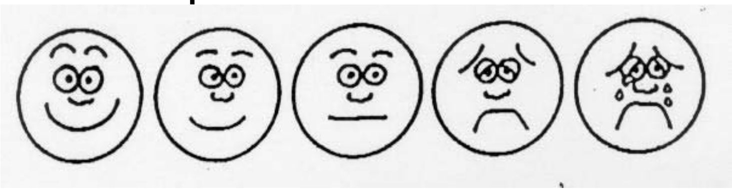

16. Cum îl face familia să se simtă pe copilul dumneavoastră?

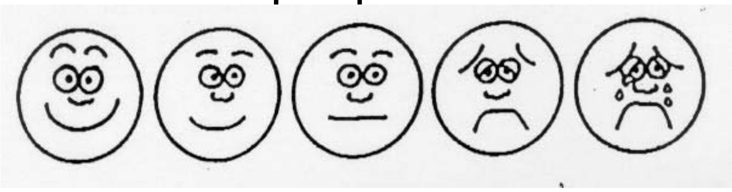

17. Cum îl afectează pe copil durerea cauzată de boala sa?

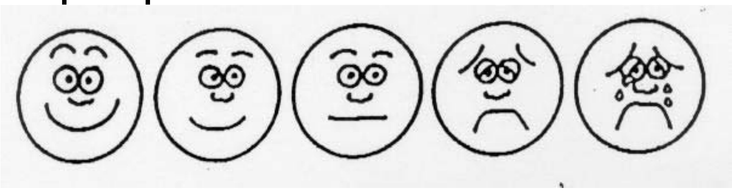

18. Cât de obosit se simte copilul dumneavoastră datorită bolii sale?

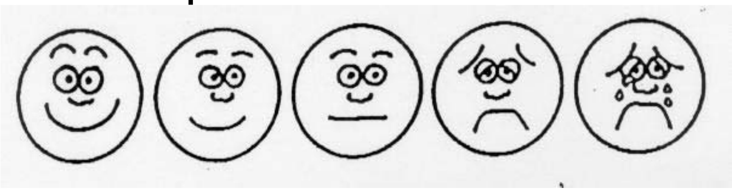

Copyright © 2007 Lakshmi Nandini Moorthy. Toate drepturile rezervate.

La întrebările 20 A- 26 A, răspundeți cu "da" sau "nu." Apoi, urmați indicațiile.

19 A. Prietenii copilului dumneavoastră s-au schimbat datorită bolii de care suferă copilul dumneavoastră ?
Vă rugăm să încercuiți "da" sau "nu".
Da Nu

Dacă ați răspuns cu "da", mergeți la întrebarea 20 B.
20 B. Cum influențează boala felul în care copilul dumneavoastră relaționează cu prietenii? Încercuiți figura care indică ce simte copilul dumneavoastră.

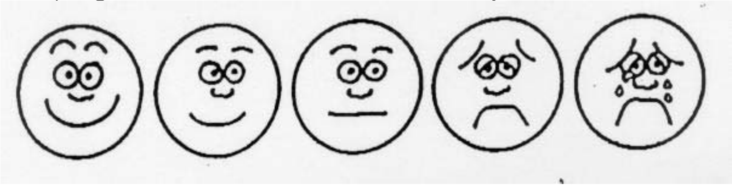

20 A. Prietenii copilului dumneavoastră înțeleg faptul că el/ea are o boală ?
Vă rugăm să încercuiți "da" sau "nu".
Da Nu

Dacă ați răspuns cu "nu", mergeți la întrebarea 21 B.
21 B. Cum se simte copilul dumneavoastră datorită faptului că prietenii săi nu înțeleg că el/ea suferă de o boală? Încercuiți figura care indică ce simte copilul dumneavoastră.

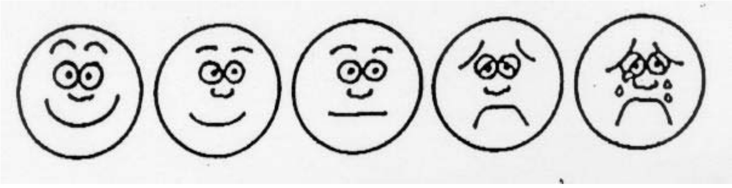

21 A. Boala afectează activitățile şcolare ale copilului dumneavoastră?
Vă rugăm să încercuiți "da" sau "nu".
Da Nu

Dacă ați răspuns cu "da", mergeți la întrebarea 22 B.
22 B. Ce crede copilul dumneavoastră despre modul în care boala îi afectează activitățile şcolare? Încercuiți figura care indică ce simte copilul dumneavoastră.

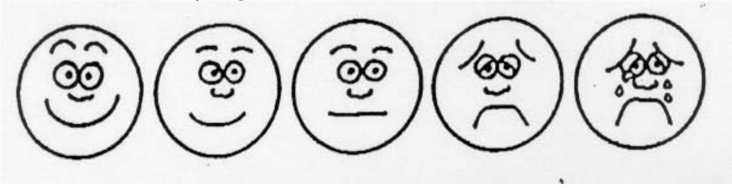

Copyright © 2007 Lakshmi Nandini Moorthy. Toate drepturile rezervate.

22 A. Copilul dumneavoastră lipseşte de la şcoală datorită bolii sale ?
Vă rugăm să încercuiţi "da" sau "nu".

Da   Nu

Dacă aţi răspuns cu "da", mergeţi la întrebarea 23 B.

23 B. Ce simte copilul dumneavoastră despre faptul că lipseşte de la şcoală datorită bolii sale? Încercuiţi figura care indică ce simte copilul dumneavoastră.

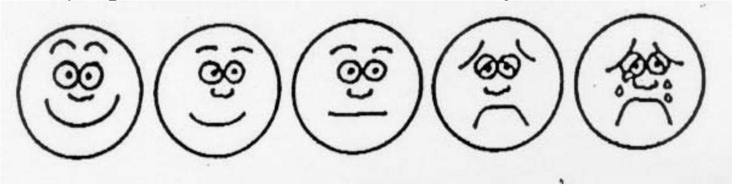

23 A. Boala afectează activităţile extra-şcolare ale copilului dumneavoastră ?
Vă rugăm să încercuiţi "da" sau "nu".

Da   Nu

Dacă aţi răspuns cu "da", mergeţi la întrebarea 24 B.

24 B. Ce crede copilul dumneavoastră despre modul în care boala sa îi afectează activităţile extra-şcolare? Încercuiţi figura care indică ce simte copilul dumneavoastră.

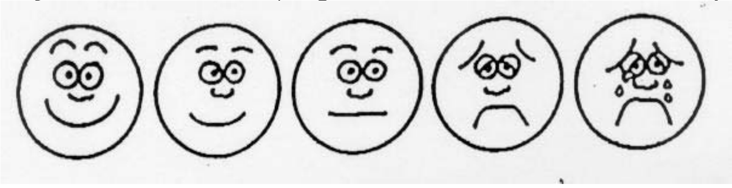

25 A. Boala afectează activităţile distractive pe care copilul dumneavoastră le desfăşoară împreună cu prietenii?
Vă rugăm să încercuiţi "da" sau "nu".

Da   Nu

Dacă aţi răspuns cu "da", mergeţi la întrebarea 25 B.

25 B. Ce crede copilul dumneavoastră despre faptul că boala sa îi afectează activităţile distractive pe care le desfăşoară împreună cu prietenii? Încercuiţi figura care indică ce simte copilul dumneavoastră.

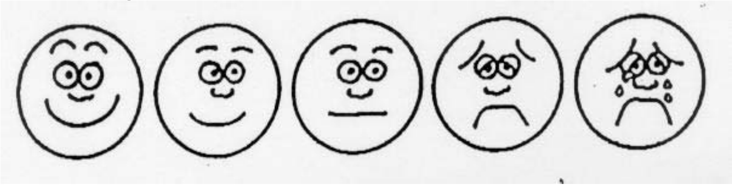

Copyright © 2007 Lakshmi Nandini Moorthy. Toate drepturile rezervate.

26 A. Boala afectează participarea copilului dumneavoastră la diferite activități fizice (ca de exemplu: orele de sport de la şcoală, alergatul, plimbarea, un joc preferat în aer liber) ?
Vă rugăm să încercuiți “da” sau “nu”.

Da   Nu

Dacă ați răspuns cu “da”, mergeți la întrebarea 26B.

26 B. Ce crede copilul dumneavoastră despre faptul că boala sa îi afectează participarea la diferite activități fizice (ca de exemplu: orele de sport de la şcoală, alergatul, plimbarea, un joc preferat în aer liber)? Încercuiți figura care indică ce simte copilul dumneavoastră.

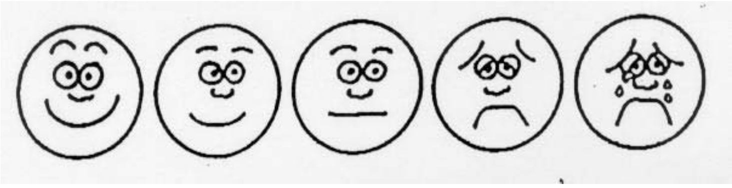

Copyright © 2007 Lakshmi Nandini Moorthy. Toate drepturile rezervate.

MĂSURAREA SIMPLĂ A IMPACTULUI BOLII LA TINERI (SMILY-Boală) – Raportul copilului

Numărul studiului __ Data __/__/200_

Pentru acest chestionar, am preluat cele 5 figuri de mai jos din Scala Wong-Baker pentru Cuantificarea Durerii (FACES), pe care le-am modificat cu permisiunea lui D.L. Wong. Aceste 5 figuri vor apărea sub fiecare întrebare.

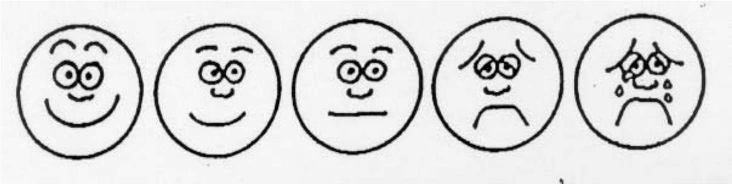

Din Wong DL, Hockenberry-Eaton M, Wilson D,Winkelstein ML, Schwartz P.: Wong's Essentials of Pediatric Nursing, ed. 6, St. Louis, 2001, Mosby, p 1301. Copyright Mosby. Reprodus cu acord.

SMILY-boală este proprietatea lui L. Nandini Moorthy, MD MS, RWJMS-UMDNJ, New Brunswick, NJ. În prezent, chestionarul poate fi utilizat doar de L. Nandini Moorthy, MD MS şi de personalul din echipa sa de cercetare.

ÎNTREBĂRI

În acest chestionar, vă vom adresa mai multe întrebări despre boala dumneavoastră şi despre felul în care vă percepeţi boala. Nu există răspunsuri greşite. Dacă ceva este neclar vă rugăm să ne cereți explicații. Dacă doriți să scrieți unele comentarii, vă rugăm să utilizați verso paginilor 5 şi 6.

Diferite lucruri vă afectează viața, făcându-vă vesel sau trist. Există câteva lucruri pe care le considerați importante pentru dumneavoastră , pe care puteți (sau nu puteți) să le faceți. Toate aceste lucruri diferite contribuie la calitatea vieții dumneavoastră.

1. Care este calitatea vieții dumneavoastră? Vă rugăm să încercuiți figura care indică cel mai bine calitatea vieții dumneavoastră.

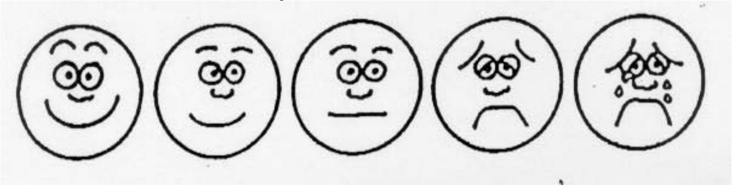

2. Care este evoluția bolii dumneavoastră în acest moment? Vă rugăm să încercuiți figura care indică cel mai bine evoluția de moment a bolii dumneavoastră.

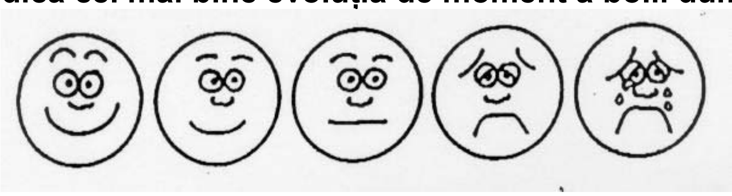

Copyright © 2007 Lakshmi Nandini Moorthy. Toate drepturile rezervate.

Întrebările 3-19 se referă la modul în care v-ați simțit în ultima lună datorită bolii dumneavoastră. Vă rugăm să încercuiți figura care indică cel mai bine răspunsul dumneavoastră.

3. Cum vă simțiți în legătură cu fapul că suferiți de această boală ?

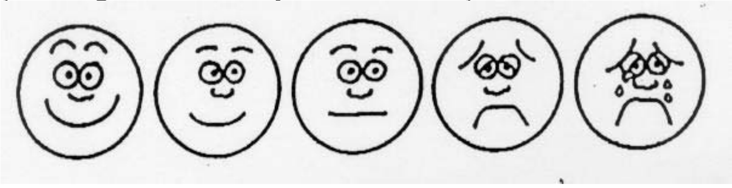

4. Cum vă influențează boala mersul la şcoală?

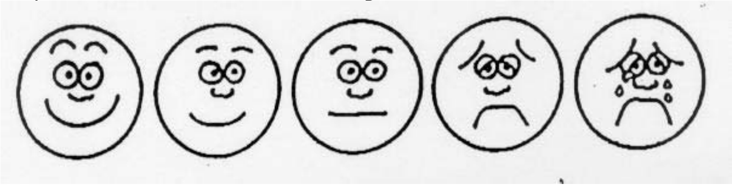

5. Cum vă influențează boala îmbrăcatul zilnic?

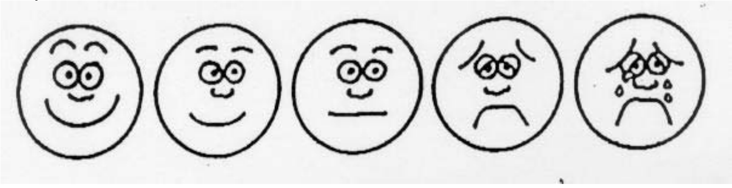

6. Cum vă influențează boala felul în care arătați?

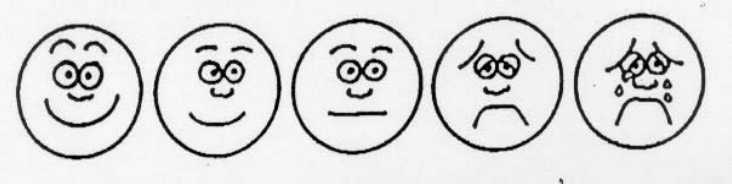

7. Cum vă influențează boala felul în care vă vedeți pe dumneavoastră înşivă?

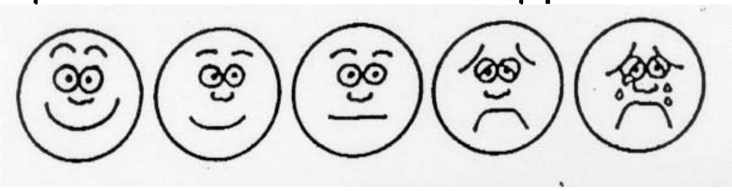

8. Cum vă simțiți când trebuie să vorbiți despre boala dumneavoastră?

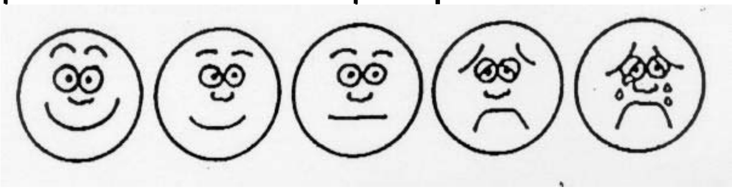

9. Ce simțiți când trebuie să mergeți la medic din cauza bolii dumneavoastră?

Copyright © 2007 Lakshmi Nandini Moorthy. Toate drepturile rezervate.

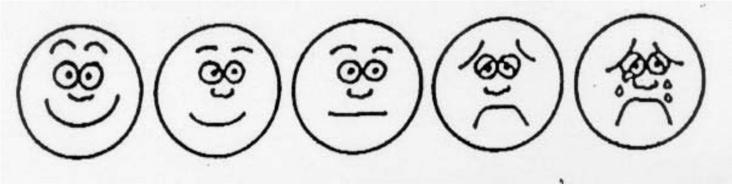

10. Ce simțiți când trebuie să mergeți la spital din cauza bolii dumneavoastră ?

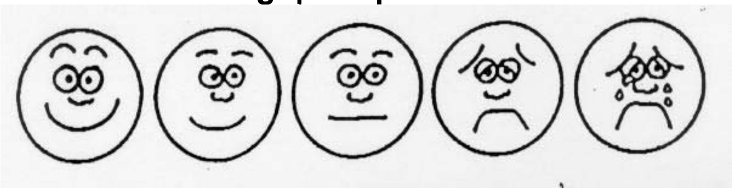

11. Cum vă influențează boala dumneavoastră perspectiva asupra viitorului?

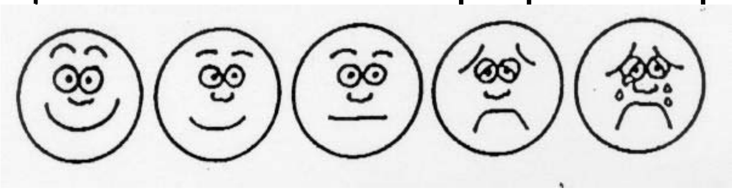

12. Ce părere aveți despre faptul că trebuie să luați medicamente pentru boala dumneavoastră?

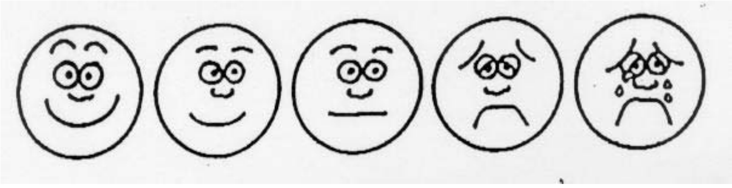

13. Cât de îngrijorat sunteți despre efectele secundare ale medicației pe care o luați pentru boala dumneavoastră?

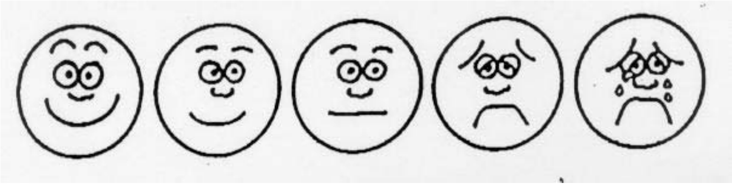

14. Cât de îngrijorat sunteți despre agravarea bolii dumneavoastră?

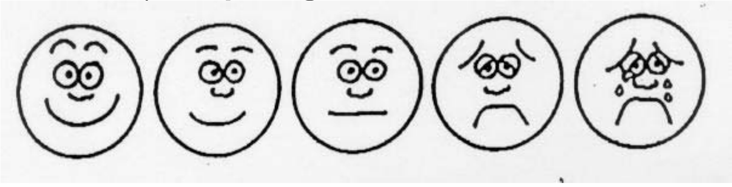

Copyright © 2007 Lakshmi Nandini Moorthy. Toate drepturile rezervate.

15. Cât de singur vă simțiți datorită bolii dumneavoastră?

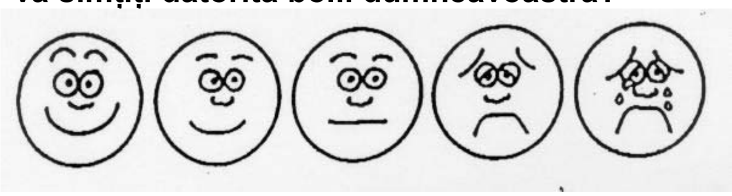

16. Cum vă face familia să vă simțiți?

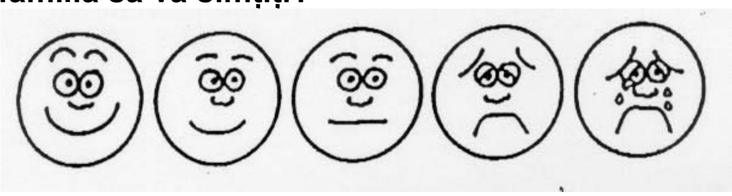

17. Cum vă afectează durerea cauzată de boala dumneavoastră?

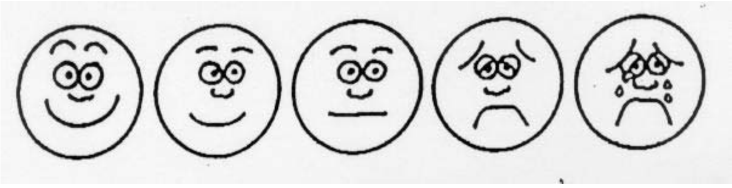

18. Cât de obosit vă simțiți datorită bolii dumneavoastră?

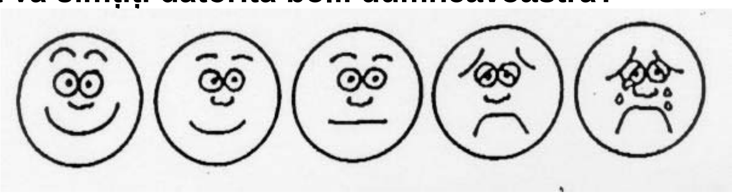

Copyright © 2007 Lakshmi Nandini Moorthy. Toate drepturile rezervate.

La întrebările 20 A- 26 A, răspundeți cu "da" sau "nu." Apoi urmați indicațiile.

19 A. Prietenii mei s-au schimbat datorită bolii mele ?
Vă rugăm încercuiți "da" sau "nu".
Da Nu

Dacă ați răspuns cu "da", mergeți la întrebarea 20 B.
20 B. Cum influențează boala dumneavoastră relația cu prietenii dumneavoastră? Încercuiți figura care indică ce simțiți.

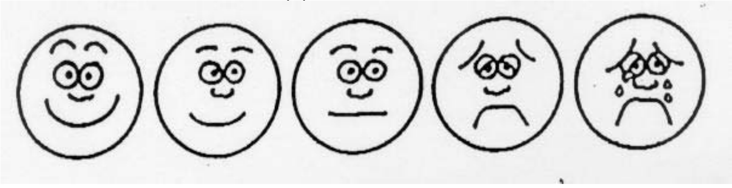

21 A. Prietenii mei înțeleg faptul că am o boală ?
Vă rugăm să încercuiți "da" sau "nu".
Da Nu

Dacă ați răspuns cu "nu", mergeți la întrebarea 21 B.
21 B. Ce credeți despre faptul că prietenii dumneavoastră nu înțeleg că suferiți de o boală? Încercuiți figura care indică ce simțiți.

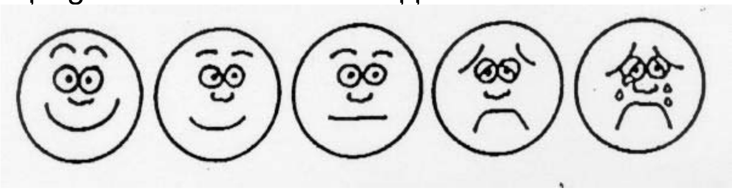

22 A. Boala mea afectează activitățile mele şcolare ?
Vă rugăm să încercuiți "da" sau "nu".
Da Nu

Dacă ați răspuns cu "da", mergeți la întrebarea 22 B.
22 B. Ce credeți despre modul în care boala dumneavoastră vă afectează activitățile şcolare? Încercuiți figura care indică ce simțiți.

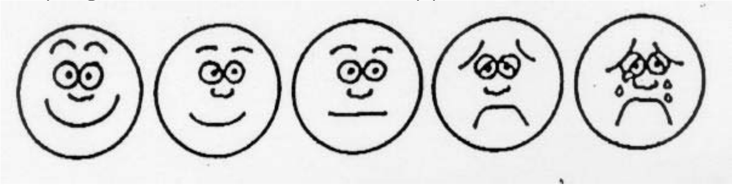

Copyright © 2007 Lakshmi Nandini Moorthy. Toate drepturile rezervate.

23 A. Lipsesc de la şcoală datorită bolii mele ?
Vă rugăm să încercuiţi “da” sau “nu”.
Da Nu

Dacă aţi răspuns cu “da”, mergeţi la întrebarea 23 B.
23 B. Ce simţiţi despre faptul că lipsiţi de la şcoală datorită bolii dumneavoastră? Încercuiţi figura care indică ce simţiţi.

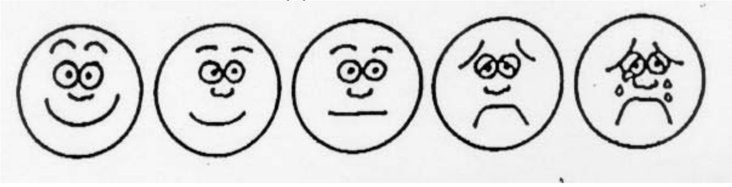

24 A. Boala mea afectează activităţile mele extra-şcolare ?
Vă rugăm să încercuiţi “da” sau “nu”.
Da Nu

Dacă aţi răspuns cu “da”, mergeţi la întrebarea 24 B.
24 B. Ce credeţi despre modul în care boala dumneavoastră vă afectează activităţile extra-şcolare? Încercuiţi figura care indică ce simţiţi.

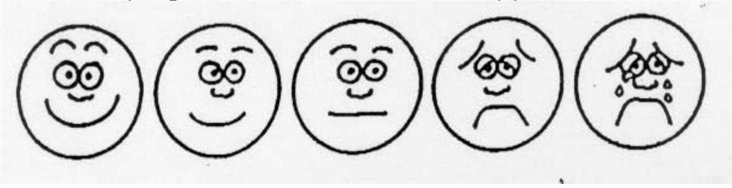

25 A. Boala mea afectează activităţile distractive pe care le desfăşor împreună cu prietenii mei ?
Vă rugăm să încercuiţi “da” sau “nu”.
Da Nu

Dacă aţi răspuns cu “da”, mergeţi la întrebarea 25 B.
25 B. Ce credeţi despre faptul că boala dumneavoastră vă afectează activităţile distractive pe care le desfăşuraţi împreună cu prietenii dumneavoastră? Încercuiţi figura care indică ce simţiţi.

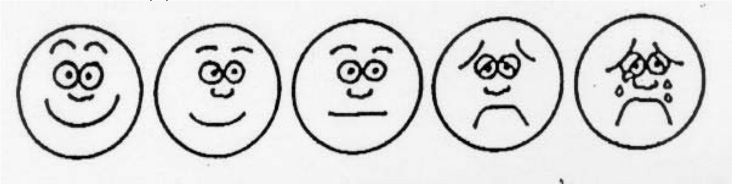

Copyright © 2007 Lakshmi Nandini Moorthy. Toate drepturile rezervate.

26 A. Boala mea afectează participarea mea la diferite activităţi fizice (ca de exemplu: orele de sport de la şcoală, alergatul, plimbarea, jocul meu preferat în aer liber) ?
Vă rugăm să încercuiţi “da” sau “nu”.

Da Nu

Dacă aţi răspuns cu “da”, mergeţi la întrebarea 26B.

26 B. Ce credeţi despre faptul că boala dumneavoastră vă afectează participarea la diferite activităţi fizice (ca de exemplu: orele de sport de la şcoală, alergatul, plimbarea, jocul meu preferat în aer liber)? Încercuiţi figura care indică ce simţiţi.

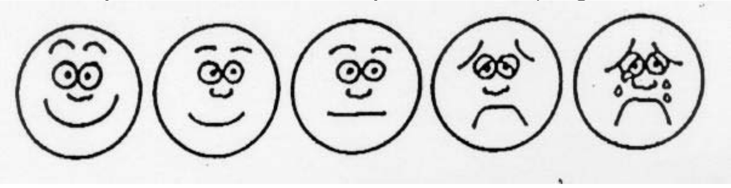

Copyright © 2007 Lakshmi Nandini Moorthy. Toate drepturile rezervate.

JEDNOSTAVNA PROCENA UTICAJA BOLESTI NA MLADE (SMILY-Illness) –
Upitnik za decu

Serbia **Broj studije __ Datum __/__/20__**

Za ovaj upitnik smo izabrali 5 izraza lica (dole prikazana) iz skale merenja bola po Wong-Baker, koja su izmenjena uz dozvolu D.L. Wong-a. Ovih 5 izraza lica će se pojavljivati ispod svakog pitanja. Izaberi jedan od izraza lica kao odgovor.

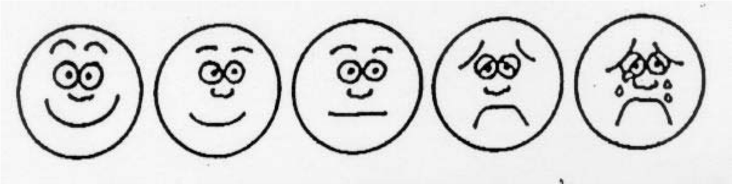

From Wong DL, Hockenberry-Eaton M, Wilson D,Winklestein ML, Schwartz P.: Wong's Essentials of Pediatric Nursing, ed. 6, St. Louis, 2001, Mosby, p 1301. Copyright Mosby. Reprinted with Permission.

SMILY-Illness je autorsko delo L. Nandini Moorthy, MD MS, RWJMS-UMDNJ, Nju Brunzvik, država Nju Džerzi. U ovom trenutku, upitnik mogu da koriste samo L. Nandini Moorthy, MD MS i njeno istraživačko osoblje.

PITANJA

U ovom upitniku postavićemo ti različita pitanja u vezi sa tvojom bolešću i o tome kako se osećaš. Nema pogrešnih odgovora. Slobodno nas pitaj ako ti nešto nije jasno. Ako želiš da napišeš bilo kakav komentar, to možeš da učiniš na poleđini 5. i 6. stranice.

Različite stvari utiču na tvoj život i čine te srećnim/srećnom ili tužnim/tužnom. Postoje određene stvari koje su bitne za tebe i koje si u stanju da uradiš (ili nisi). Sve ove stvari čine kvalitet tvog života.

1. Označi izrazom lica kakav je kvalitet tvog života.

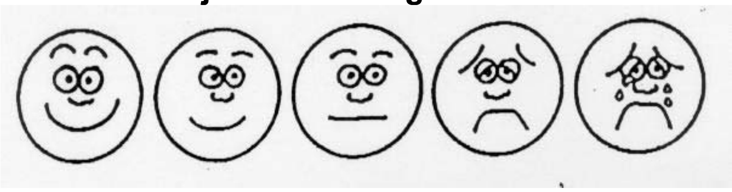

2. Označi izraz lica koji pokazuje kakva je tvoja bolest u ovom trenutku.

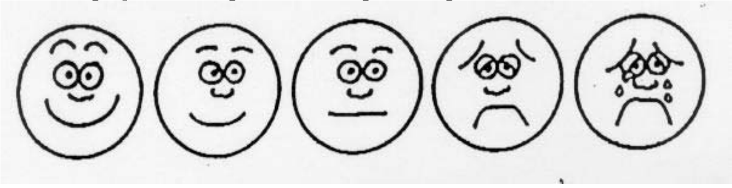

Copyright © 2007 Lakshmi Nandini Moorthy. Sva prava zadržana.

Pitanja od 3 do 19 se odnose na to kako se zbog bolesti osećaš poslednjih mesec dana.
Molimo te da zaokružiš izraz lica koji najbolje pokazuje kako se osećaš.

3. Kako se osećaš zbog bolesti?

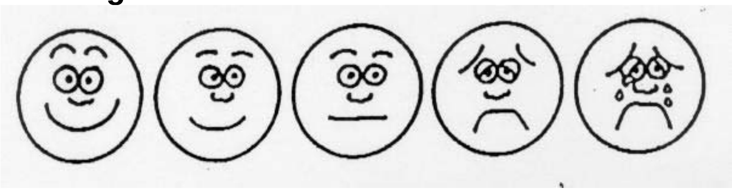

4. Kako se zbog bolesti osećaš kada treba da ideš u školu?

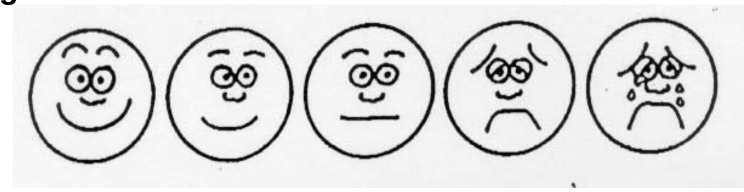

5. Kako bolest utiče na tvoje svakodnevno oblačenje?

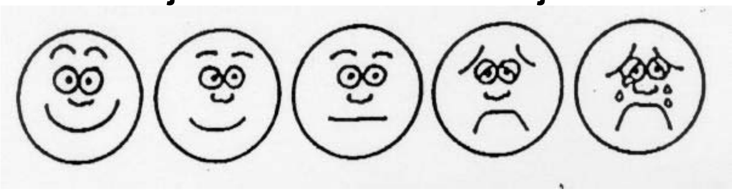

6. Kako bolest utiče na razmišljanja o tvom sopstvenom izgledu?

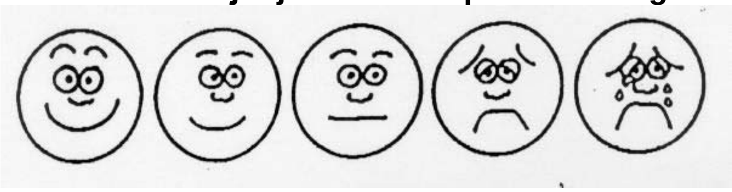

7. Kako bolest utiče na tvoja razmišljanja o sebi?

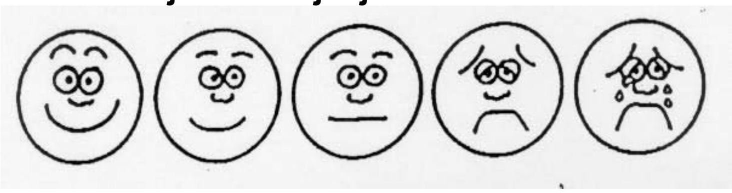

8. Kako se osećaš kada treba da pričaš o svojoj bolesti?

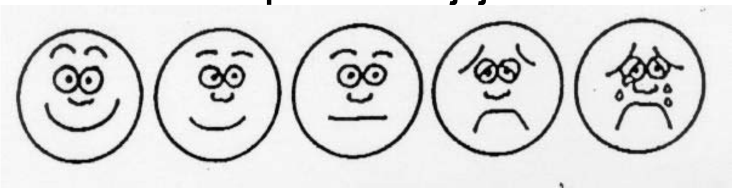

Copyright © 2007 Lakshmi Nandini Moorthy. Sva prava zadržana.

9. Kako se osećaš kada zbog bolesti treba da odeš kod lekara?

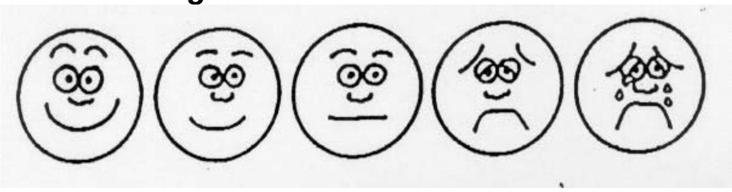

10. Kako se osećaš kada zbog bolesti treba da ideš u bolnicu?

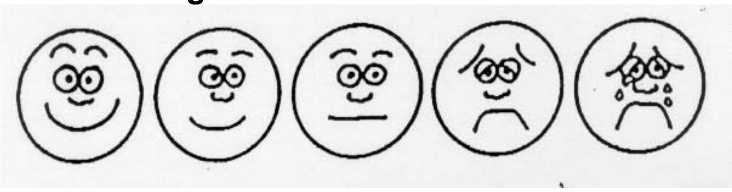

11. Kako bolest utiče na tvoja razmišljanja o budućnosti?

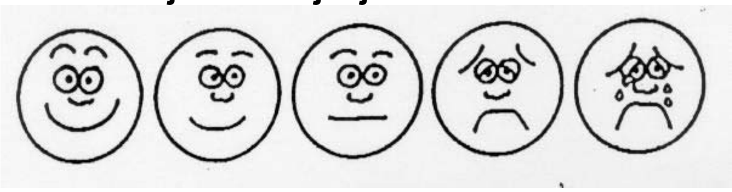

12. Kako se osećaš kada treba da piješ lekove protiv bolesti?

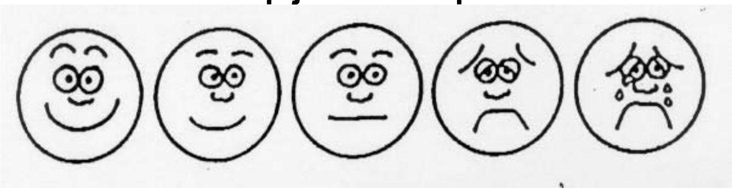

13. Koliko si zabrinut/a zbog neželjenih delovanja lekova koje uzimaš zbog bolesti?

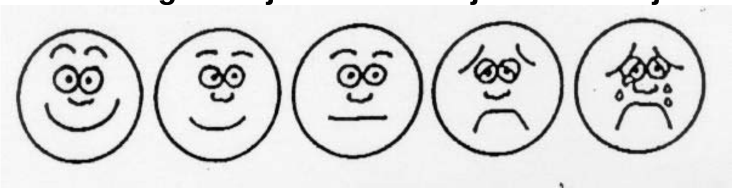

14. Koliko si zabrinut/a da ti se bolest pogoršava?

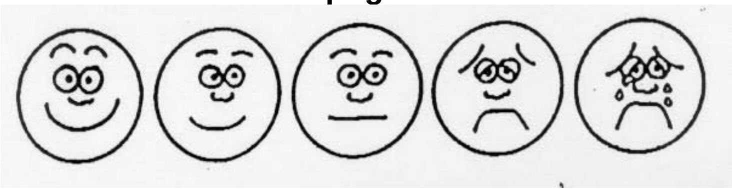

15. Označi koliko se osećaš usamljeno zbog bolesti?

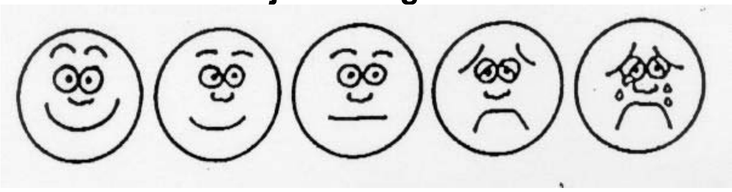

Copyright © 2007 Lakshmi Nandini Moorthy. Sva prava zadržana.

16. Kako porodica utiče na tebe?

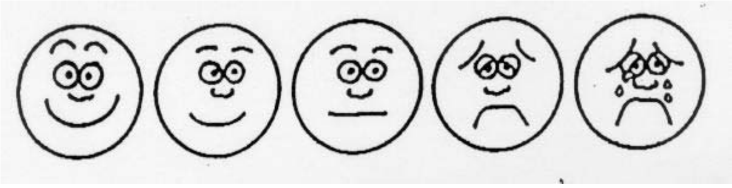

17. Kako se osećaš kada ti bolest izaziva bol?

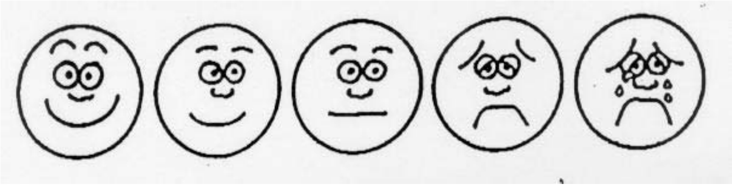

18. Kako se osećaš što zbog bolesti ne možeš da se sunčaš?

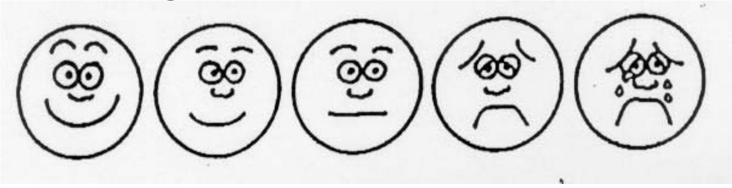

19. Koliko te bolest čini umornim/umornom?

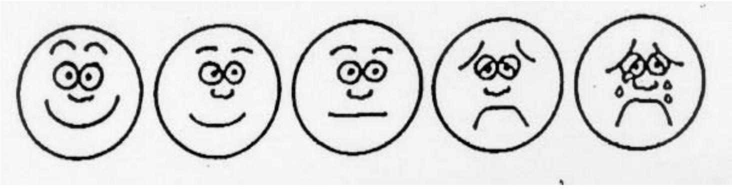

Copyright © 2007 Lakshmi Nandini Moorthy. Sva prava zadržana.

Na pitanja 20 A-26 A odgovori sa DA ili NE. Zatim te molimo da slediš uputstva.

20 A. Moji prijatelji su promenili svoj odnos prema meni od kada sam se razboleo/la. Molimo te da zaokružiš "da" ili "ne". Da Ne

Ako je odgovor "DA" pređi na pitanje 20 B.

20 B. Kako se osećaš zbog toga što su tvoji prijatelji promenili svoj odnos prema tebi? Molimo te da zaokružiš izraz lica koji najbolje pokazuje kako se osećaš.

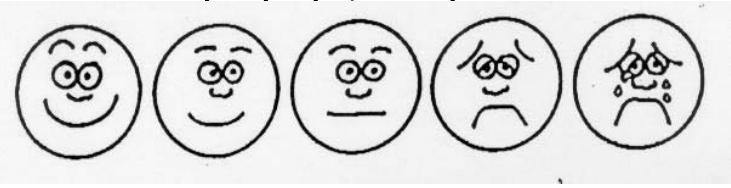

21 A. Moji drugovi shvataju da sam se razboleo/la. Da Ne

Ako je odgovor "ne" pređi na pitanje 21 B.

21 B. Kako se osećaš što tvoji prijatelji nemaju razumevanja za tvoju bolest? Molimo te da zaokružiš izraz lica koji najbolje pokazuje kako se osećaš.

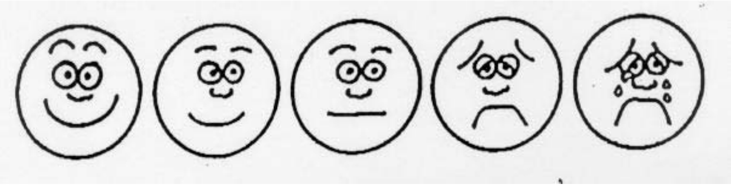

22 A. Bolest utiče na moje školske aktivnosti. Da Ne

Ako je odgovor "da" pređi na pitanje 22 B.

22 B. Kako se osećaš zbog toga što bolest utiče na tvoje školske aktivnosti? Molimo te da zaokružiš izraz lica koji najbolje pokazuje kako se osećaš.

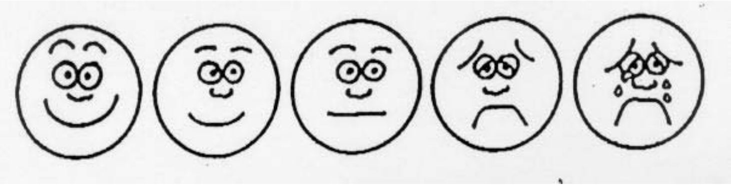

Copyright © 2007 Lakshmi Nandini Moorthy. Sva prava zadržana.

23 A. Izostajem iz škole zbog bolesti. **Da** **Ne**

Ako je odgovor "da" pređi na pitanje 23 B.

23 B. Kako se osećaš što zbog bolesti izostaješ iz škole? Molimo te da zaokružiš izraz lica koji najbolje pokazuje kako se osećaš.

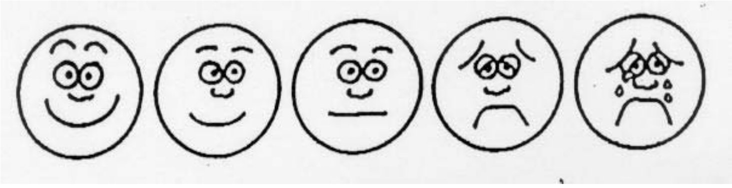

24 A. Bolest utiče na moje vanškolske aktivnosti. **Da** **Ne**

Ako je odgovor "da" pređi na pitanje 24 B.

24 B. Kako se osećaš što zbog bolesti ne možeš da se baviš vanškolskim aktivnostima? Molimo te da zaokružiš izraz lica koji najbolje pokazuje kako se osećaš.

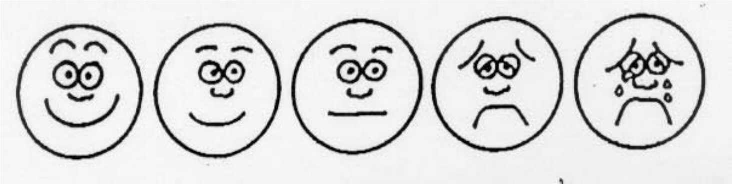

25 A. Bolest me sprečava da se družim sa prijateljima. **Da** **Ne**

Ako je odgovor "da" pređi na pitanje 25 B.

25 B. Kako se osećaš što te bolest sprečava da se družiš sa prijateljima? Molimo te da zaokružiš izraz lica koji najbolje pokazuje kako se osećaš.

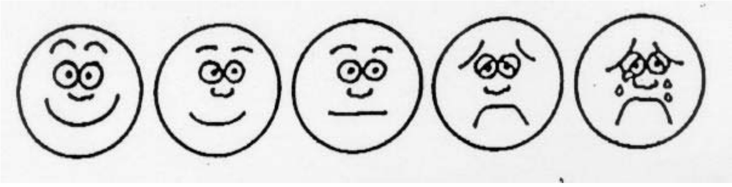

Copyright © 2007 Lakshmi Nandini Moorthy. Sva prava zadržana.

26 A. Bolest me sprečava da se bavim fizičkim aktivnostima kao što su gimnastika, trčanje ili bavljenje omiljenim sportom itd.

Da **Ne**

Ako je odgovor “da”, pređi na pitanje 26 B.

26 B. Kako se osećaš što ne možeš da se baviš fizičkim aktivnostima kao što su gimnastika, trčanje ili bavljenje omiljenim sportom? Molimo te da zaokružiš izraz lica koji najbolje pokazuje kako se osećaš.

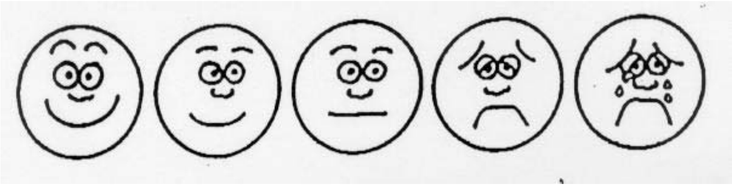

Copyright © 2007 Lakshmi Nandini Moorthy. Sva prava zadržana.

JEDNOSTAVNA PROCENA UTICAJA BOLESTI NA MLADE - (SMILY-Illness) - Upitnik za roditelje

Broj studije __ Datum __/__/20__

Za ovaj upitnik smo izabrali 5 izraza lica (dole prikazana) iz skale merenja bola po Wong-Baker, koja su izmenjena uz dozvolu D.L. Wong-a. Ovih 5 izraza lica će se pojavljivati ispod svakog pitanja. Izaberite jedan od izraza lica kao odgovor.

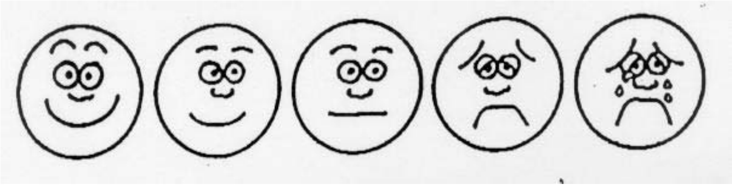

From Wong DL, Hockenberry-Eaton M, Wilson D,Winklestein ML, Schwartz P.: Wong's Essentials of Pediatric Nursing, ed. 6, St. Louis, 2001, Mosby, p 1301. Copyright Mosby. Reprinted with Permission.

(SMILY-Illness) je autorsko delo L. Nandini Moorthy, MD MS, RWJMS-UMDNJ, Nju Brunzvik, država Nju Džerzi. U ovom trenutku, upitnik mogu da koriste samo L. Nandini Moorthy, MD MS i njeno istraživačko osoblje.

PITANJA

U ovom upitniku postavićemo Vam različita pitanja u vezi sa bolešću Vašeg deteta i o tome kako se ono oseća. Nema pogrešnih odgovora. Slobodno nas pitajte ako Vam nešto nije jasno. Ako želite da napišete bilo kakav komentar, to možete da učinite na poleđini upitnika.

Različite stvari utiču na život Vašeg deteta i čine ga srećnim ili tužnim. Postoje određene stvari koje su bitne za Vaše dete i koje je dete u stanju da uradi (ili nije). Sve ove stvari čine kvalitet života Vašeg deteta.

1. Označite izrazom lica kakav je kvalitet života Vašeg deteta.

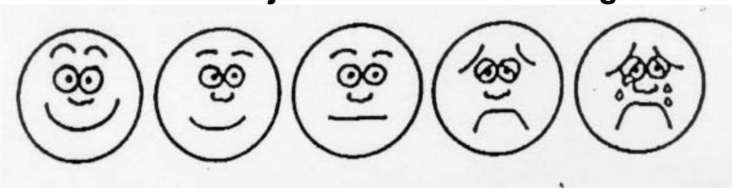

2. Označite izraz lica koji pokazuje kakva je bolest Vašeg deteta u ovom trenutku.

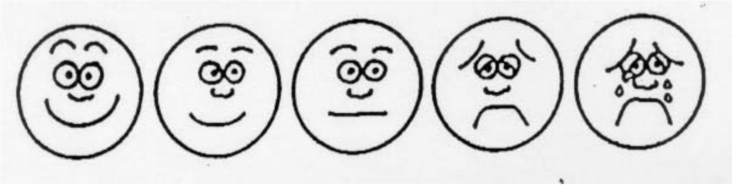

Copyright © 2007 Lakshmi Nandini Moorthy. Sva prava zadržana.

Pitanja od 3 do 19 se odnose na to kako se Vaše dete osećalo poslednjih mesec dana u vezi sa svojom bolešću. Molimo Vas da zaokružite izraz lica koji po Vama najbolje pokazuje kako se Vaše dete osećalo.

3. Kako se Vaše dete oseća zbog bolesti?

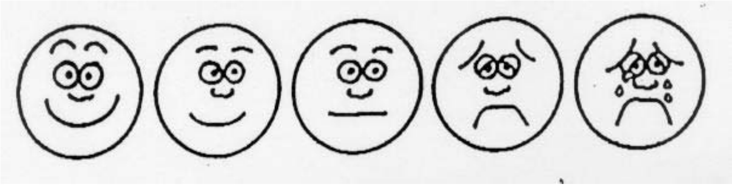

4. Kako se Vaše dete oseća zbog bolesti kada treba da ide u školu?

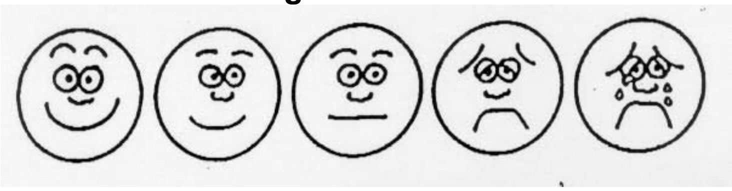

5. Kako bolest utiče na svakodnevno oblačenje Vašeg deteta?

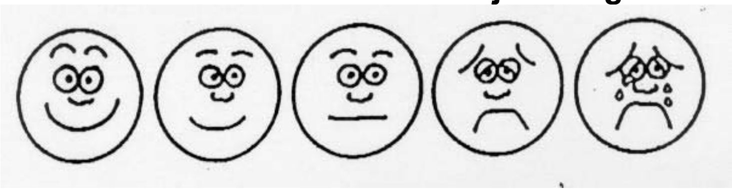

6. Kako bolest utiče na razmišljanja Vašeg deteta o sopstvenom izgledu?

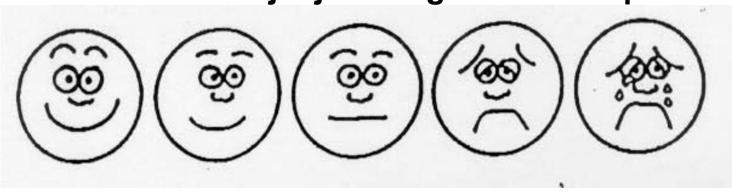

7. Kako bolest utiče na razmišljanja Vašeg deteta o svojoj ličnosti?

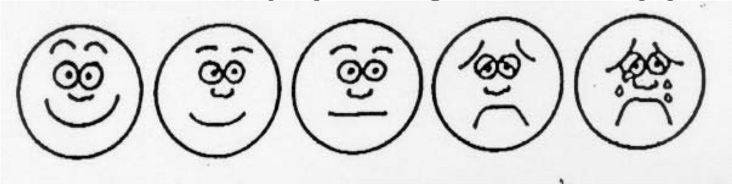

Copyright © 2007 Lakshmi Nandini Moorthy. Sva prava zadržana.

8. **Kako se Vaše dete oseća kada treba da priča o svojoj bolesti?**

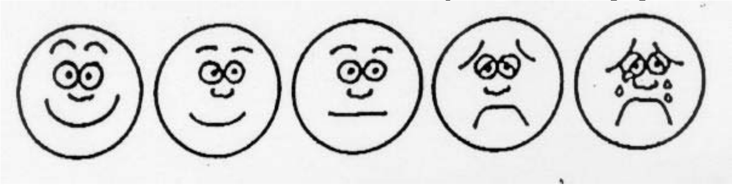

9. **Kako se Vaše dete oseća kada zbog bolesti treba da ode kod lekara?**

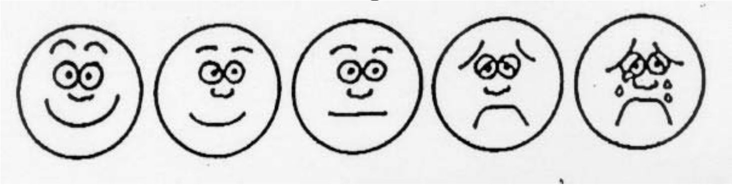

10. **Kako se Vaše dete oseća kada zbog bolesti treba da ide u bolnicu?**

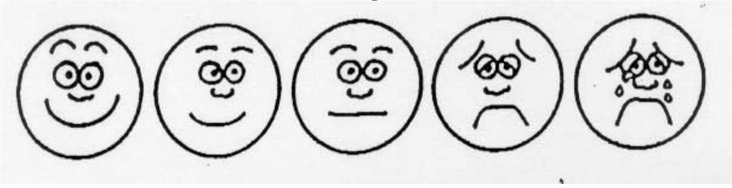

11. **Kako bolest utiče na razmišljanja Vašeg deteta o njegovoj/njenoj budućnosti?**

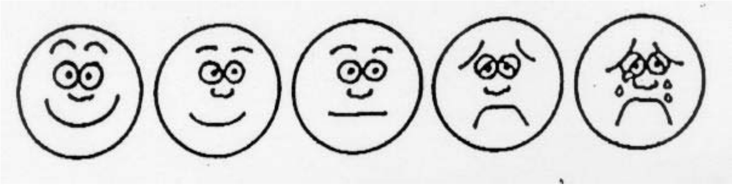

12. **Kako se Vaše dete oseća kada treba da pije lekove protiv bolesti?**

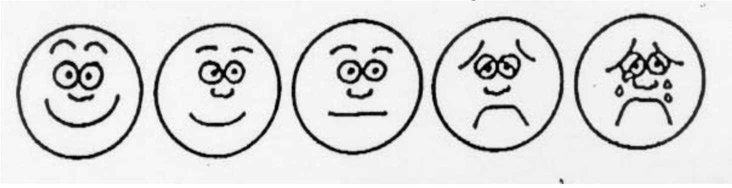

13. **Koliko je Vaše dete zabrinuto zbog neželjenih efekata lekova koje pije zbog bolesti?**

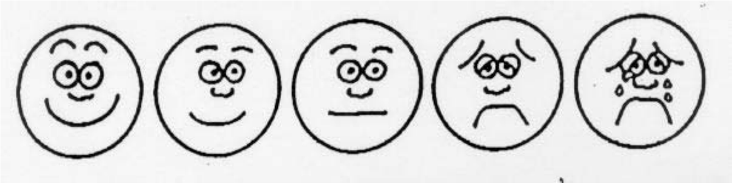

Copyright © 2007 Lakshmi Nandini Moorthy. Sva prava zadržana.

14. Koliko je Vaše dete zabrinuto zbog toga što mu se bolest pogoršava?

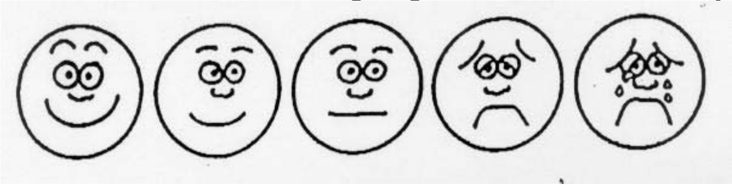

15. Koliko se Vaše dete oseća usamljeno zbog bolesti?

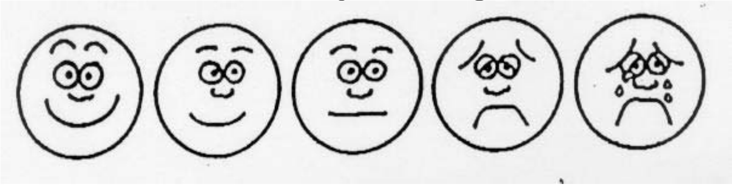

16. Kako Vi i porodica utičete na Vaše dete?

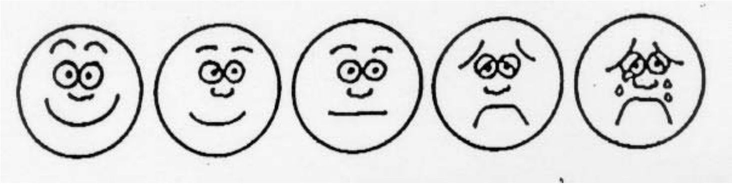

17. Kako se Vaše dete oseća kada mu bolest izaziva bol?

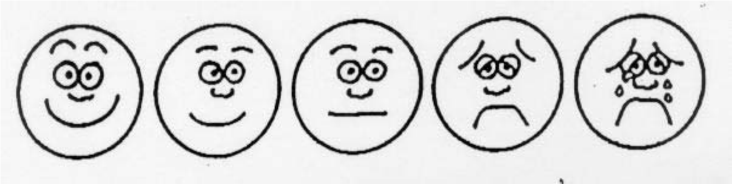

18. Kako se Vaše dete oseća što zbog bolesti ne može da se sunča?

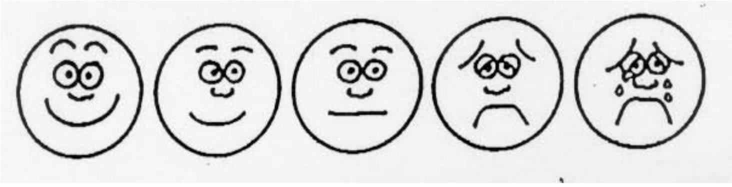

19. Koliko bolest Vaše dete čini umornim/umornom?

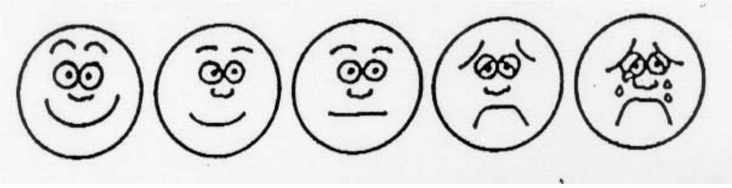

Copyright © 2007 Lakshmi Nandini Moorthy. Sva prava zadržana.

Na pitanja 20A-26A odgovorite sa DA ili NE. Zatim Vas molimo da sledite uputstva.

20 A. Drugovi Vašeg deteta su promenili svoj odnos prema njemu od kada se razboleo/la.

Molimo Vas da zaokružite "da" ili "ne". Da Ne

Ako je odgovor "DA" pređite na pitanje 20 B .

20 B. Kako se Vaše dete oseća zbog toga što su njeni /njegovi drugovi promenili svoj odnos prema njemu od kada se razbolela/o? Molimo Vas da zaokružite izraz lica koji po Vama najbolje pokazuje kako se Vaše dete oseća.

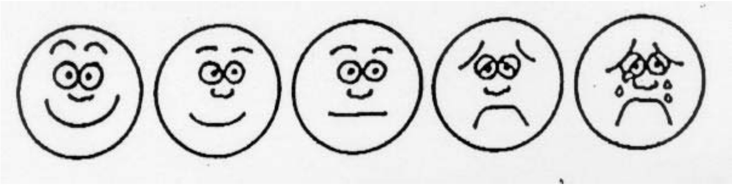

21 A. Drugovi Vašeg deteta shvataju da boluje. Da Ne

Ako je odgovor "ne" pređite na pitanje 21 B.

21 B. Kako se Vaše dete oseća zbog toga što njeni/njegovi drugovi ne razumeju da je oboleo/la? Molimo Vas da zaokružite izraz lica koji po Vama najbolje pokazuje kako se Vaše dete oseća.

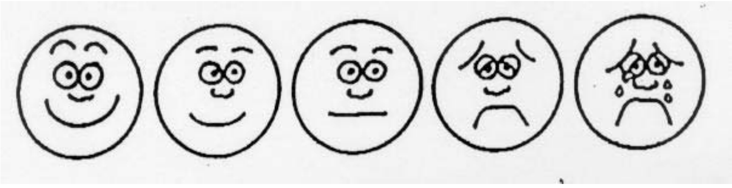

22 A. Bolest utiče na školske aktivnosti Vašeg deteta. Da Ne

Ako je odgovor "da" pređite na pitanje 22 B

22 B. Kako se Vaše dete oseća zbog toga što bolest utiče na njene/njegove školske aktivnosti? Molimo Vas da zaokružite izraz lica koji po Vama najbolje pokazuje kako se Vaše dete oseća.

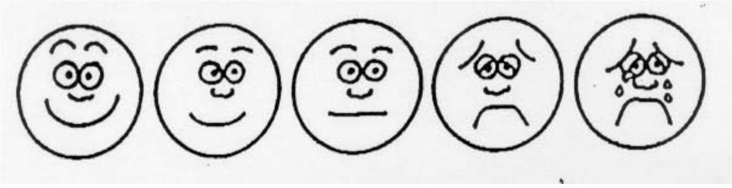

23 A. Vaše dete često izostaje iz škole zbog bolesti. Da Ne

Ako je odgovor "da" pređite na pitanje 23 B.

23 B. Kako se Vaše dete oseća što izostaje iz škole zbog bolesti? Molimo Vas da zaokružite izraz lica koji po Vama najbolje pokazuje kako se Vaše dete oseća.

Copyright © 2007 Lakshmi Nandini Moorthy. Sva prava zadržana.

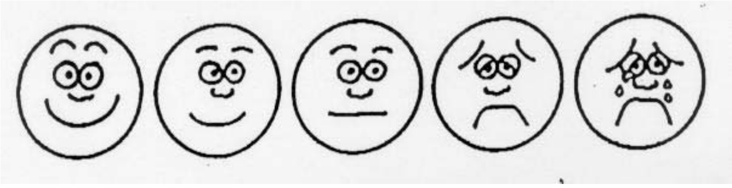

24 A. Bolest utiče na vanškolske aktivnosti Vašeg deteta. Da Ne

Ako je odgovor "da" pređite na pitanje 24 B.

24 B. Kako se Vaše dete oseća što zbog bolesti ne može da se bavi vanškolskim aktivnostima? Molimo Vas da zaokružite izraz lica koji po Vama najbolje pokazuje kako se Vaše dete oseća.

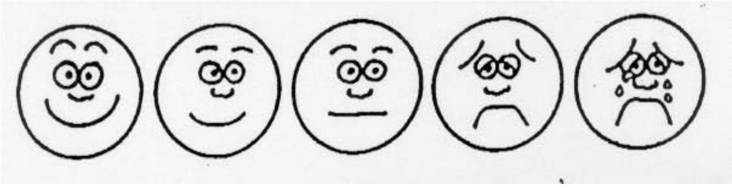

25 A. Bolest sprečava da se Vaše dete druži sa svojim prijateljima. Da Ne

Ako je odgovor "da" pređite na pitanje 25 B.

25 B. Kako se Vaše dete oseća što ga bolest sprečava da se druži sa prijateljima? Molimo Vas da zaokružite izraz lica koji po Vama najbolje pokazuje kako se Vaše dete oseća.

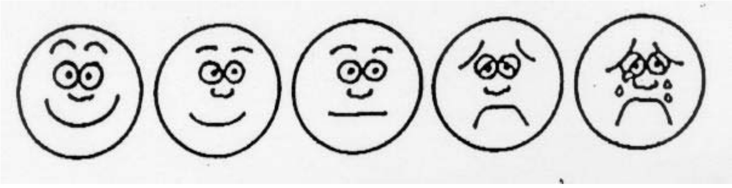

26 A. Bolest sprečava Vaše dete da se bavim fizičkim aktivnostima kao što su gimnastika, trčanje ili bavljenje omiljenim sportom itd. Da Ne

Ako je odgovor "da" pređite na pitanje 26B.

26 B. Kako se Vaše dete oseća što ne može da se bavi fizičkim aktivnostima kao što su gimnastika, trčanje ili bavljenje omiljenim sportom? Molimo Vas da zaokružite izraz lica koji po Vama najbolje pokazuje kako se Vaše dete oseća.

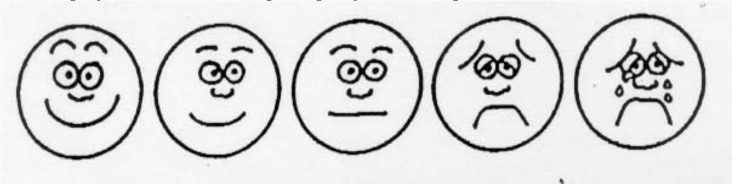

Copyright © 2007 Lakshmi Nandini Moorthy. Sva prava zadržana.

Slovenia

ENOSTAVNO MERILO ZA SPREMLJANJE VPLIVA BOLEZNI NA OTROKE IN MLADOSTNIKE (SMILY-bolezen) – verzija za otroke

Številka raziskave__ Datum__/__/20_

Pri izdelavi tega vprašalnika smo uporabili 5 obrazov iz Wong-Baker-jeve obrazne bolečinske lestvice, ki smo jo priredili z dovoljenjem D.L. Wong. Teh 5 obrazov boš zagledal pod vsakim vprašanjem.

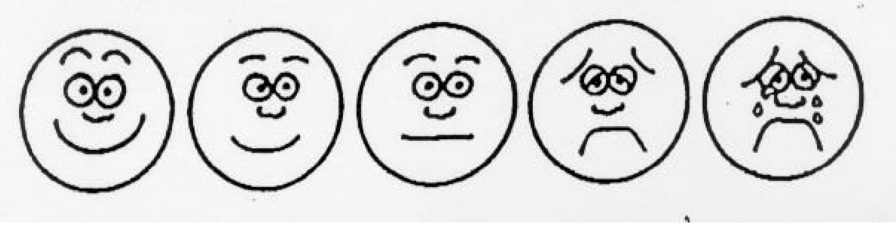

Prirejeno po Wong DL, Hockenberry-Eaton M, Wilson D, Winklestein ML, Schwartz P.: Wong's Essentials of Pediatric Nursing, ed. 6, St. Louis, 2001, Mosby, p 1301. Copyright Mosby. Natisnjeno z dovoljenjem.

Avtorske pravice za vprašalnik SMILY-bolezen ima L. Nandini Moorthy, MD, MS, RWJMS-UMDNJ, New Brunswick, NJ. Trenutno lahko ta vprašalnik uporablja samo L. Nandini Moorthy, MD, MS in raziskovalci, ki z njo sodelujejo.

VPRAŠANJA

S tem vprašalnikom te bomo vprašali številna vprašanja v zvezi s tvojo boleznijo in tvojih občutkih. Napačnih odgovorov ni. Prosim vprašaj, če česa ne boš razumel. Če želiš dopisati komentar, ga prosim napiši na hrbtni strani 5. in 6. lista.

Na tvoje življenje vplivajo različne stvari, ki te delajo srečnega ali žalostnega. Nekatere od teh stvari so zate bolj pomembne, nekatere si sposoben opraviti, medtem ko nekaterih drugih ne zmoreš. Vse te stvari skupaj predstavljajo kvaliteto tvojega življenja.

1. Kakšna je kvaliteta tvojega življenja? Prosim označi (√) obraz, ki najbolje predstavlja kvaliteto tvojega življenja.

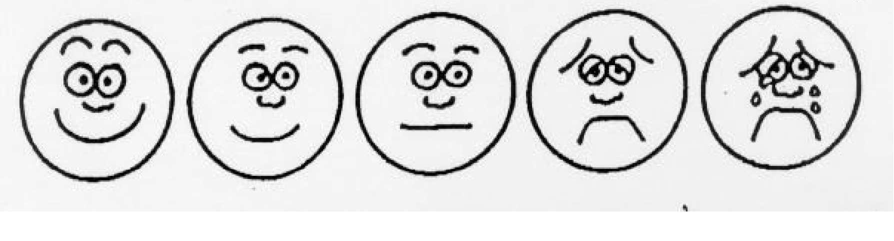

2. V kakšnem stanju je trenutno tvoja bolezen? Prosim označi (√) obraz, ki najbolje predstavlja trenutno stanje tvoje bolezni.

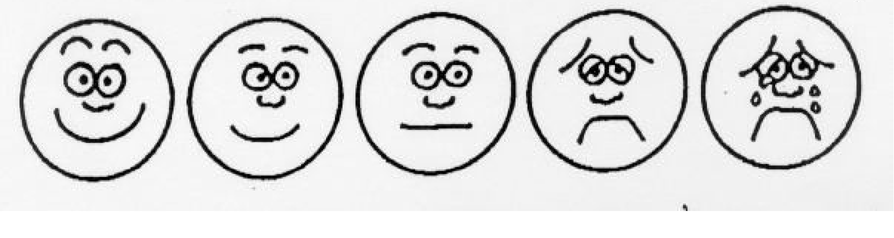

Avtorske parvice © 2007 Lakshmi Nandini Moorthy. Vse pravice pridržane

Vprašanja 3-19 so v zvezi s tem, kako si se zadnji mesec počutil zaradi bolezni. Prosim označi (√) obraz, ki najbolje predstavlja tvoj odgovor.

3. Kako se počutiš zaradi bolezni?

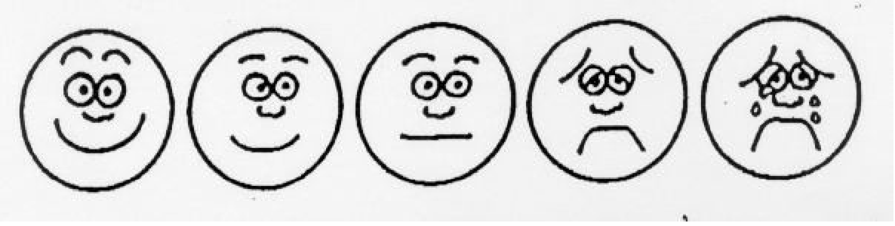

4. Kako se počutiš zaradi bolezni, kadar moraš iti v šolo?

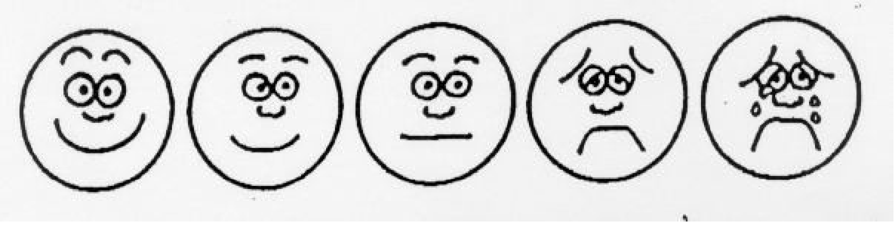

5. Kako se počutiš zaradi bolezni med vsakodnevnim oblačenjem?

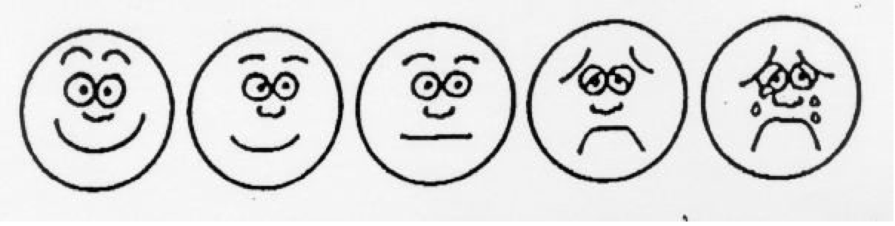

6. Kako se počutiš zaradi bolezni glede svojega izgleda?

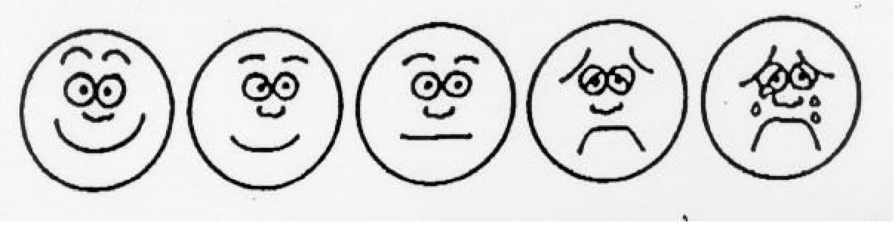

7. Kakšne občutke imaš do samega sebe zaradi bolezni?

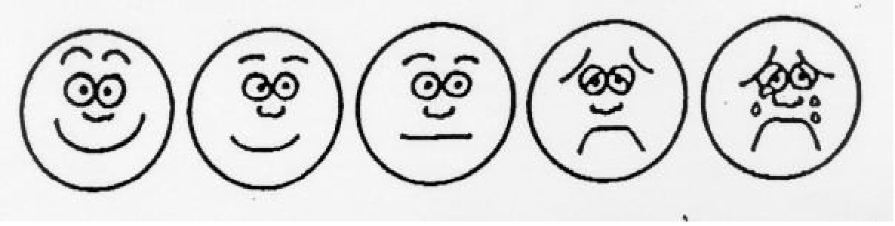

8. Kako se počutiš, ko moraš z nekom govoriti o svoji bolezni?

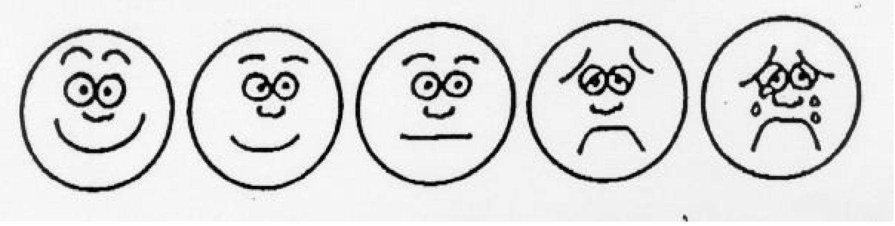

Avtorske parvice © 2007 Lakshmi Nandini Moorthy. Vse pravice pridržane

9. **Kako se počutiš zaradi bolezni kadar moraš k zdravniku?**

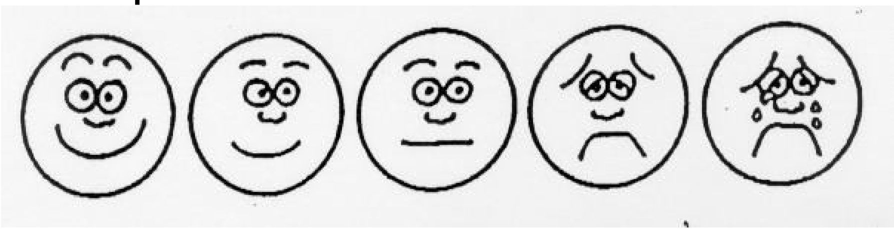

10. **Kako se počutiš zaradi bolezni kadar moraš v bolnišnico?**

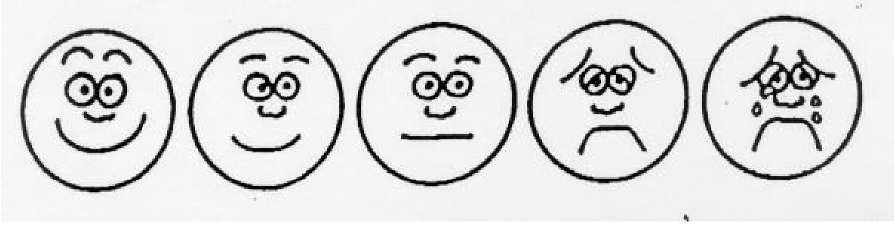

11. **Kako se počutiš zaradi bolezni glede svoje prihodnosti?**

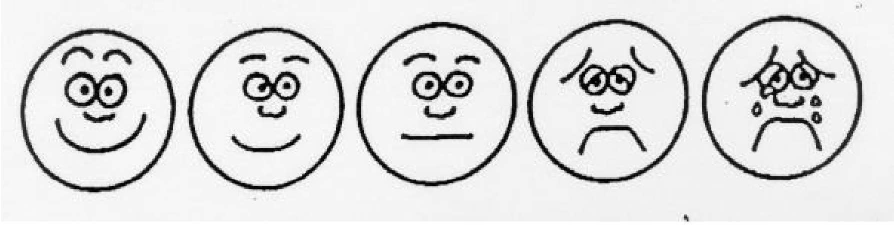

12. **Kako se počutiš, ker moraš zaradi bolezni jemati zdravila?**

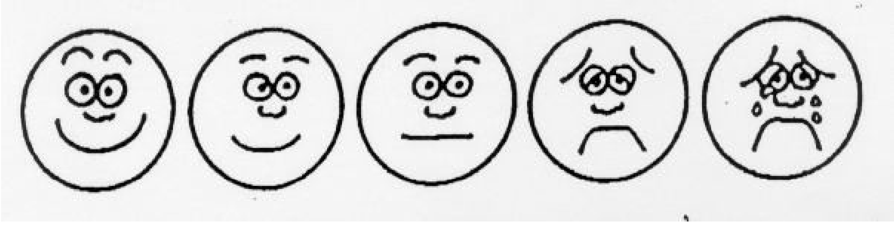

13. **Kako zaskrbljen si zaradi stranskih učinkov zdravil, ki jih moraš jemati zaradi bolezni?**

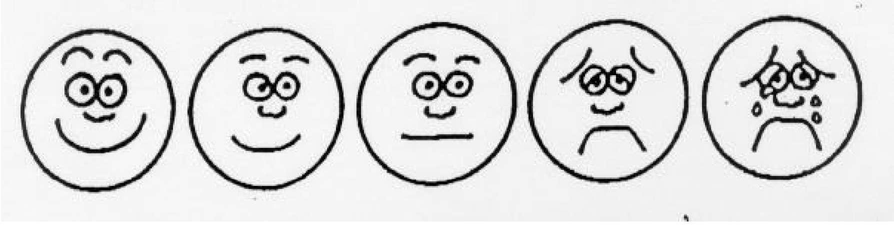

14. **Kako zaskrbljen si, da se bo tvoja bolezen poslabšala?**

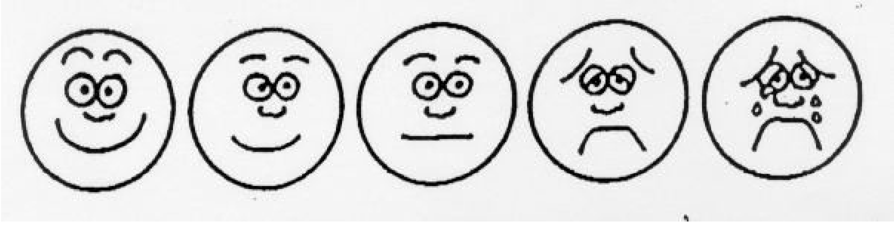

Avtorske parvice © 2007 Lakshmi Nandini Moorthy. Vse pravice pridržane

15. **Kako osamljenega so počutiš zaradi bolezni?**

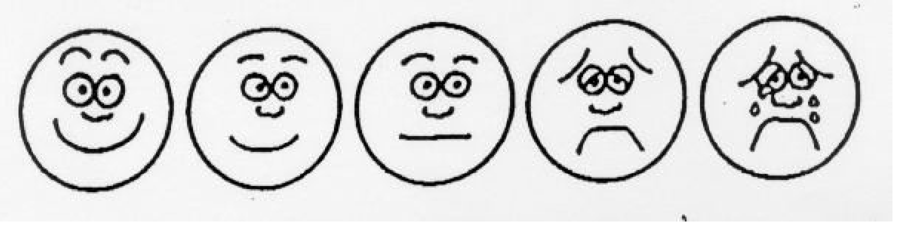

16. **Kako na tvoje počutje vpliva družina?**

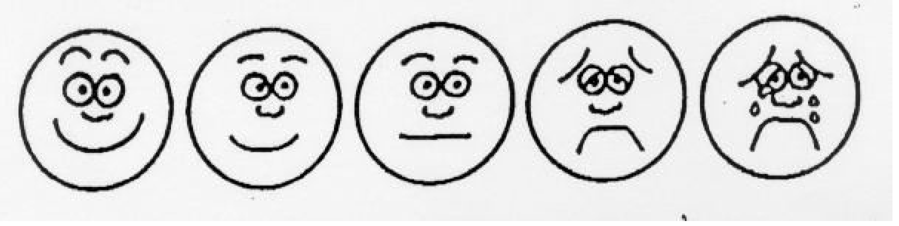

17. **Kako na tvoje počutje vpliva bolečina zaradi bolezni?**

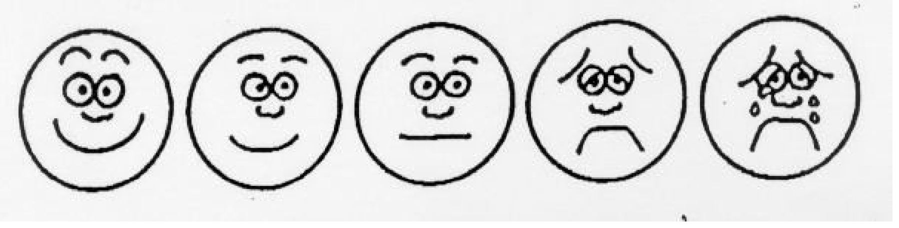

18. **Kako se počutiš, ker zaradi bolezni ne moreš ven in se izpostaviti sončnim žarkom?**

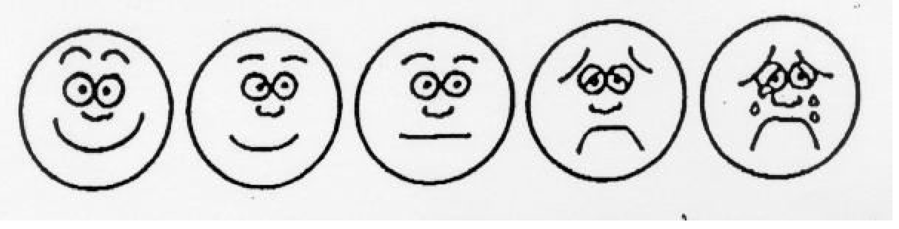

19. **Kako utrujenega se počutiš zaradi bolezni?**

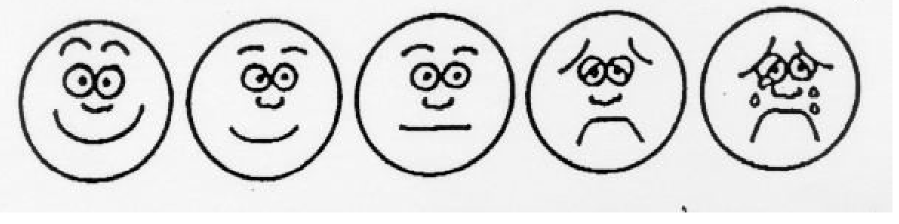

Avtorske parvice © 2007 Lakshmi Nandini Moorthy. Vse pravice pridržane

Pri vprašanjih 20A - 26A odgovori z "da" ali "ne". Nato prosim upoštevaj navodila.

20 A. Moji prijatelji so se spremenili zaradi moje bolezni. Prosim označi "da" ali "ne". Da Ne

Če si označil "da", potem nadaljuj z vprašanjem 20 B.

20 B. Kako se počutiš zaradi bolezni glede tvojih prijateljev? Prosim označi obraz, ki najbolje izraža tvoje občutke.

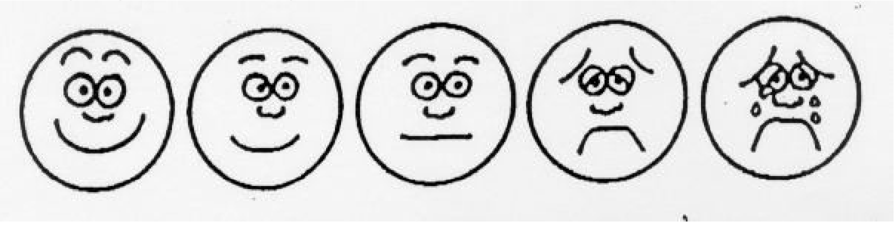

21 A. Moji prijatelji razumejo, da imam bolezen. Da Ne

Če si označili "ne", potem nadaljuj z vprašanjem 21 B.

21 B. Kako se počutiš, ker prijatelji ne razumejo tvoje bolezni? Prosim označi obraz, ki najbolje izraža tvoje občutke.

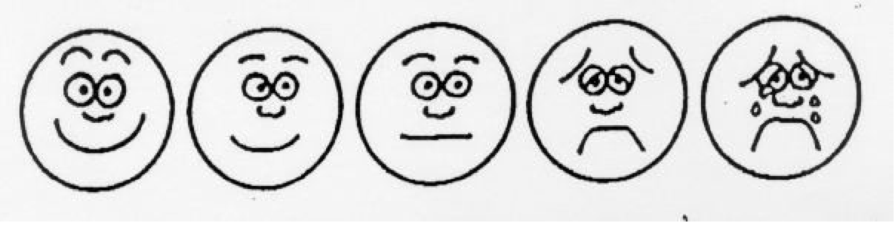

22 A. Bolezen vpliva na moj šolski uspeh. Da Ne

Če si označil "da", potem nadaljuj z vprašanjem 22 B.

22 B. Kako se počutiš, ker bolezen vpliva na tvoj šolski uspeh? Prosim označi obraz, ki najbolje izraža tvoje občutke.

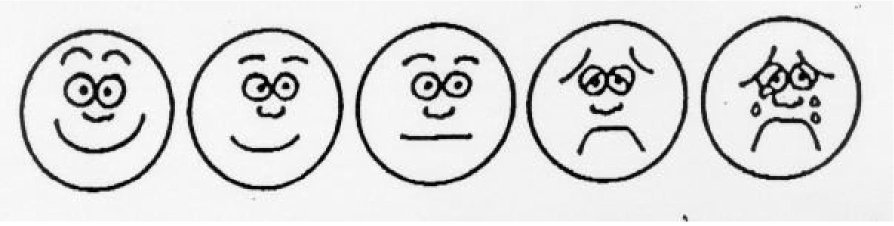

23 A. Zaradi bolezni manjkam v šoli. Da Ne

Če si označil "da", potem nadaljuj z vprašanjem 23 B.

23 B. Kako se počuti, ker zaradi bolezni manjkaš v šoli? Prosim označi obraz, ki najbolje izraža tvoje občutke.

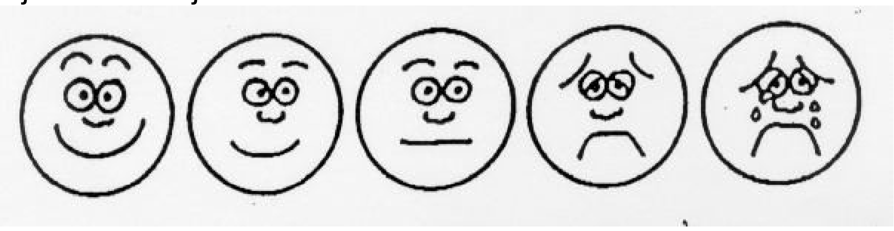

Avtorske parvice © 2007 Lakshmi Nandini Moorthy. Vse pravice pridržane

24 A. Bolezen vpliva na moje zunajšolske aktivnosti. **Da** **Ne**

Če si označil “da”, potem nadaljuj z vprašanjem 24 B.

24 B. Kako se počutiš, ker bolezen vpliva na tvoje zunajšolske aktivnosti? Prosim označi obraz, ki najbolje izraža tvoje občutke.

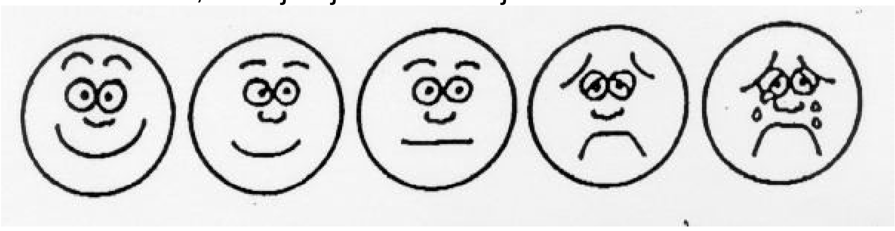

25 A. Bolezen vpliva na prijetne trenutke, ki jih preživim skupaj s prijatelji. **Da** **Ne**

Če si označil “da”, potem nadaljuj z vprašanjem 25 B.

25 B. Kako se počutiš, ker bolezen vpliva na prijetne trenutke, ki jih preživiš skupaj s prijatelji? Prosim označi obraz, ki najbolje izraža tvoje občutke.

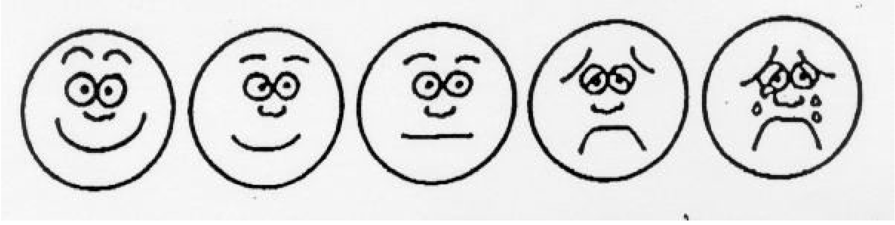

26 A. Bolezen vpliva na moje sodelovanje pri fizične aktivnosti (kot npr. šolska telovadba, tek, hoja, igranje priljubljene igre na prostem) **Da** **Ne**

Če si označil “da”, potem nadaljuj z vprašanjem 26 B.

26 B. Kako se počutiš, ker bolezen vpliva na tvoje sodelovanje pri fizičnih aktivnostih (kot npr. šolska telovadba, tek, hoja, igranje priljubljene igre na prostem)? Prosim označi obraz, ki najbolje izraža tvoje občutke.

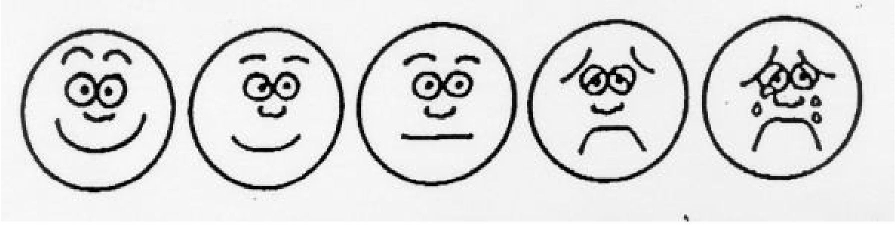

Avtorske parvice © 2007 Lakshmi Nandini Moorthy. Vse pravice pridržane

ENOSTAVNO MERILO ZA SPREMLJANJE VPLIVA BOLEZNI NA OTROKE IN MLADOSTNIKE (SMILY-bolezen) – verzija za starše

Številka raziskave__ Datum__/__/20_

Pri izdelavi tega vprašalnika smo uporabili 5 obrazov iz Wong-Baker-jeve obrazne bolečinske lestvice, ki smo jo priredili z dovoljenjem D.L. Wong. Teh 5 obrazov boste zagledali pod vsakim vprašanjem.

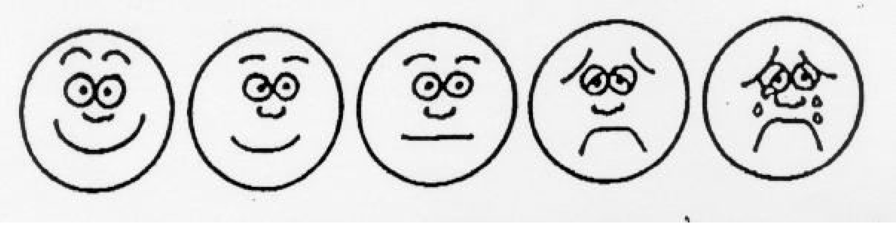

Prirejeno po Wong DL, Hockenberry-Eaton M, Wilson D, Winklestein ML, Schwartz P.: Wong's Essentials of Pediatric Nursing, ed. 6, St. Louis, 2001, Mosby, p 1301. Copyright Mosby. Natisnjeno z dovoljenjem.

Avtorske pravice za vprašalnik SMILY-bolezen ima L. Nandini Moorthy, MD, MS, RWJMS-UMDNJ, New Brunswick, NJ. Trenutno lahko ta vprašalnik uporablja samo L. Nandini Moorthy, MD, MS in raziskovalci, ki z njo sodelujejo.

VPRAŠANJA

S tem vprašalnikom Vas bomo vprašali številna vprašanja v zvezi z boleznijo Vašega otroka in Vaših občutkih. Napačnih odgovorov ni. Prosim vprašajte, če česa ne boste razumeli. Če želite dopisati komentar prosim storite to na hrbtni strani 5. in 6. lista.

Na življenje Vašega otroka vplivajo različne stvari, ki ga delajo srečnega ali žalostnega. Za nekatere od teh stvari Vaš otrok misli, da so bolj pomembne, nekatere stvari je sposoben opraviti, medtem ko nekaterih drugih ne more. Vse te stvari skupaj predstavljajo kvaliteto življenja Vašega otroka.

1. **Kakšna je kvaliteta življenja Vašega otroka? Prosim označite (√) obraz, ki najbolje predstavlja kvaliteto otrokovega življenja.**

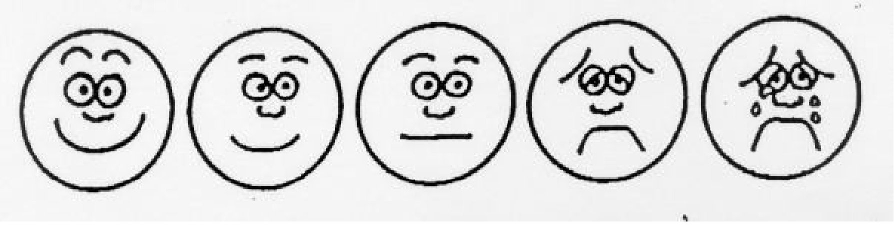

2. **V kakšnem stanju je trenutno bolezen Vašega otroka? Prosim označite (√) obraz, ki najbolje predstavlja trenutno stanje otrokove bolezni.**

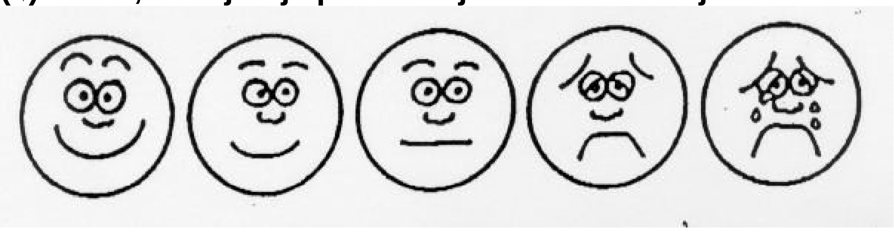

Avtorske parvice © 2007 Lakshmi Nandini Moorthy. Vse pravice pridržane.

Vprašanja 3-19 so v zvezi s tem, kako se je Vaš otrok zadnji mesec počutil zaradi bolezni. Prosim označite (√) obraz, ki najbolje predstavlja Vaš odgovor.

3. Kako se Vaš otrok počuti zaradi bolezni?

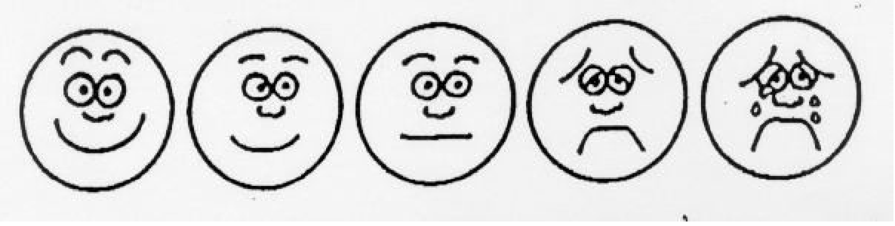

4. Kako se Vaš otrok počuti zaradi bolezni, kadar mora iti v šolo?

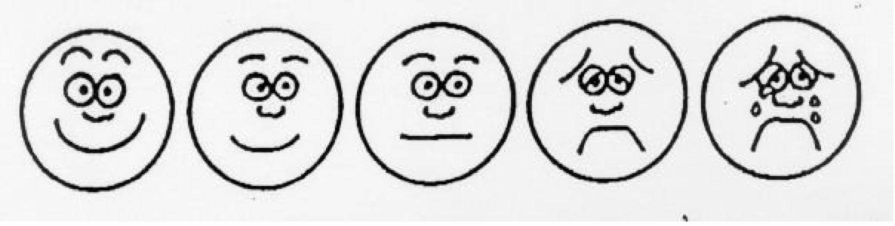

5. Kako se Vaš otrok počuti zaradi bolezni med vsakodnevnim oblačenjem?

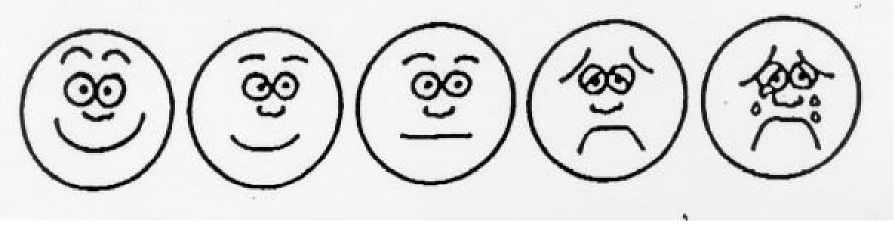

6. Kako se Vaš otrok počuti zaradi bolezni glede svojega izgleda?

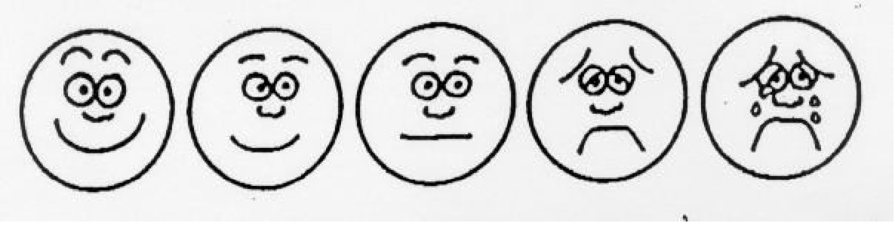

7. Kakšne občutke ima Vaš otrok do samega sebe zaradi bolezni?

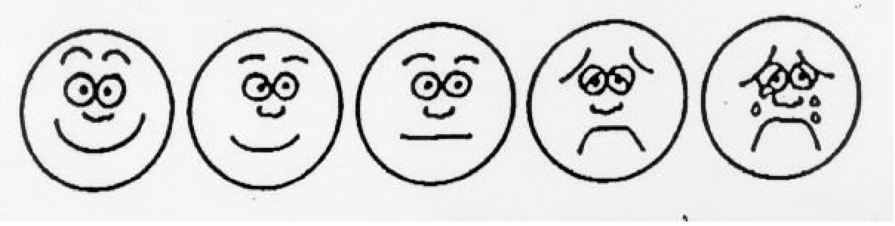

8. Kako se Vaš otrok počuti, ko mora z nekom govoriti o svoji bolezni?

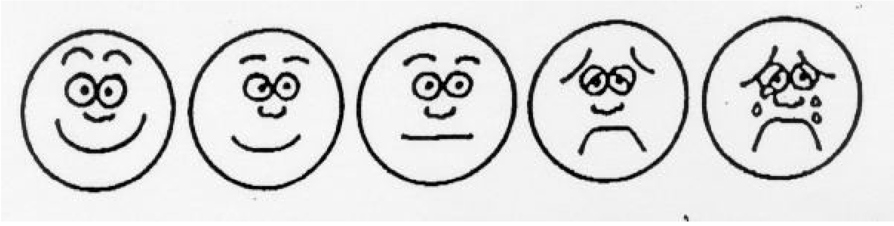

9. Kako se Vaš otrok počuti zaradi bolezni kadar mora k zdravniku?

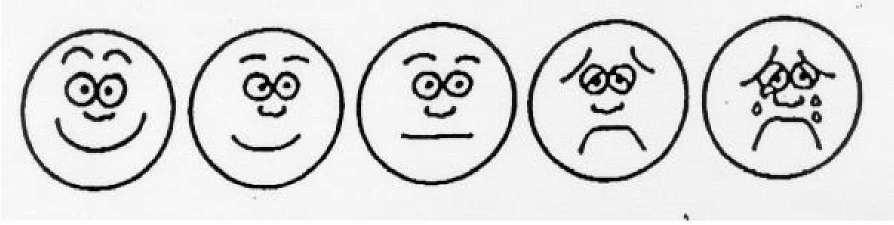

10. Kako se Vaš otrok počuti zaradi bolezni, kadar mora v bolnišnico?

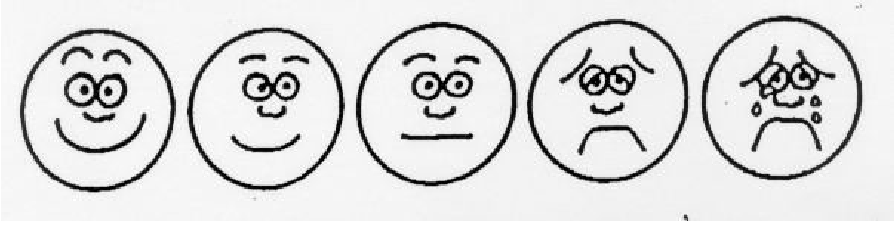

11. Kako se Vaš otrok počuti zaradi bolezni glede svoje prihodnosti?

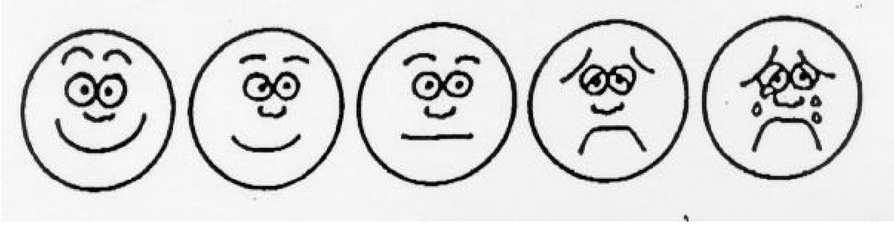

12. Kako se Vaš otrok počuti, ker mora zaradi bolezni jemati zdravila?

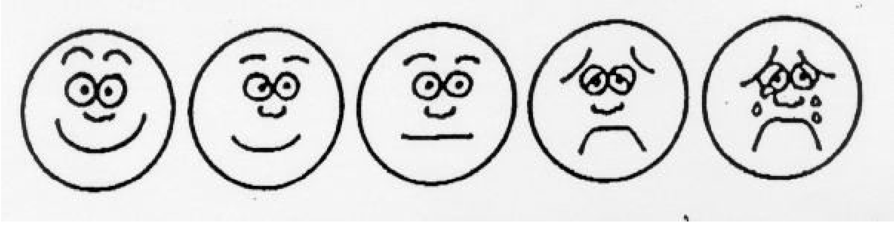

13. Kako zaskrbljen je Vaš otrok zaradi stranskih učinkov zdravil, ki jih mora jemati zaradi bolezni?

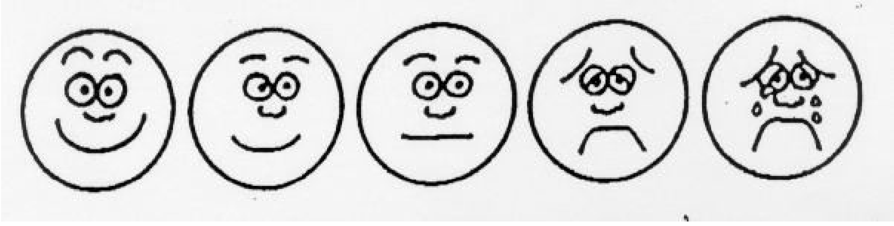

14. Kako zaskrbljen je Vaš otrok, da se bo njegova bolezen poslabšala?

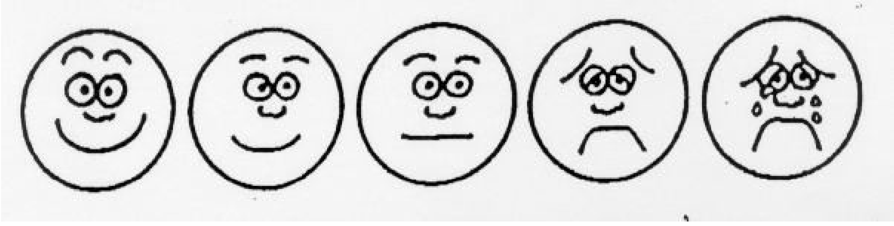

15. Ali se zaradi bolezni Vaš otrok počuti bolj osamljenega?

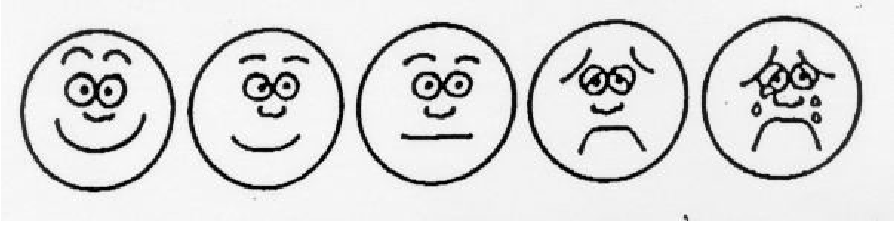

16. Kako na počutje otroka vpliva Vaša družina?

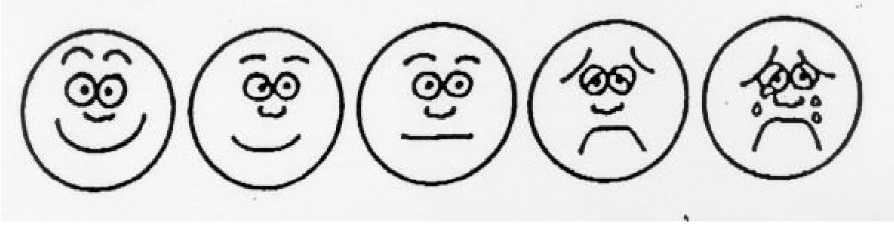

17. Kako na počutje Vašega otroka vpliva bolečina zaradi bolezni?

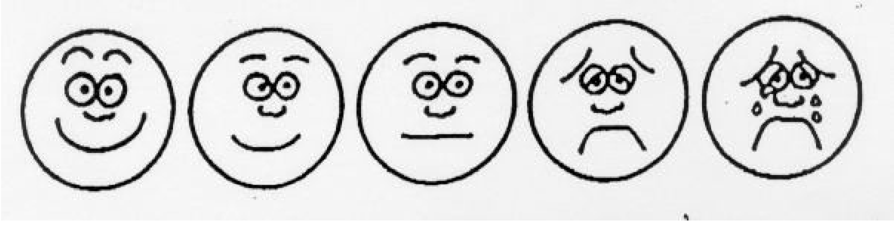

18. Kako se Vaš otrok počuti, ker zaradi bolezni ne more ven in se izpostaviti sončnim žarkom?

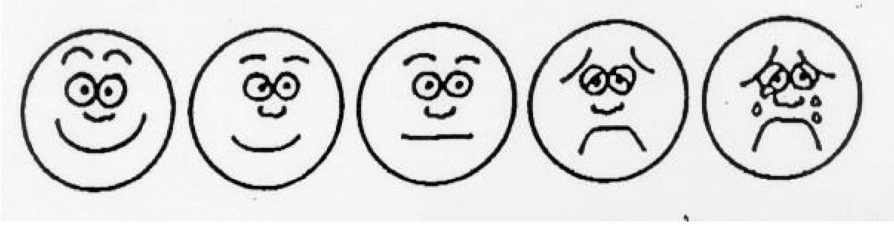

19. Kako utrujenega se Vaš otrok počuti zaradi bolezni?

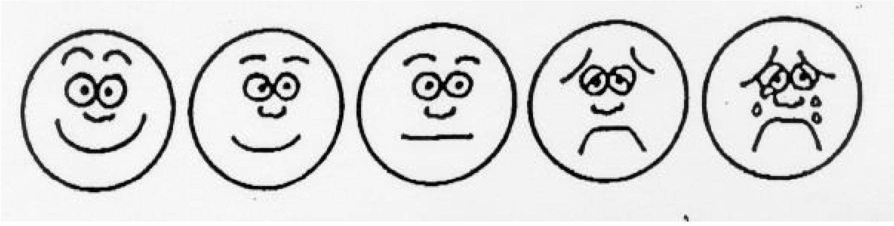

Pri vprašanjih 20A - 26A odgovorite z “da” ali “ne”. Nato prosim sledite navodilom.

20 A. Prijatelji Vašega otroka so se spremenili zaradi njegove bolezni. Prosim označite “da” ali “ne”. Da Ne

Če ste označili “da”, potem nadaljujete z vprašanjem 20 B.

20 B. Kako se Vaš otrok počuti zaradi bolezni glede svojih prijateljev? Prosim označite obraz, ki najbolje izraža občutke Vašega otroka.

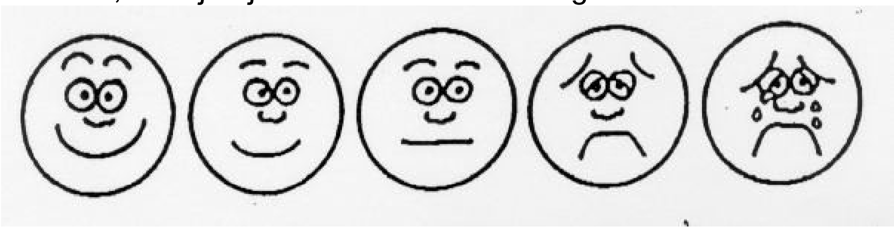

21 A. Prijatelji Vašega otroka razumejo, da ima Vaš otrok bolezen. Da Ne

Če ste označili “ne”, potem nadaljujete z vprašanjem 21 B.

21 B. Kako se Vaš otrok počuti, ker njegovi prijatelji ne razumejo njegove bolezni? Prosim označite obraz, ki najbolje izraža občutke Vašega otroka.

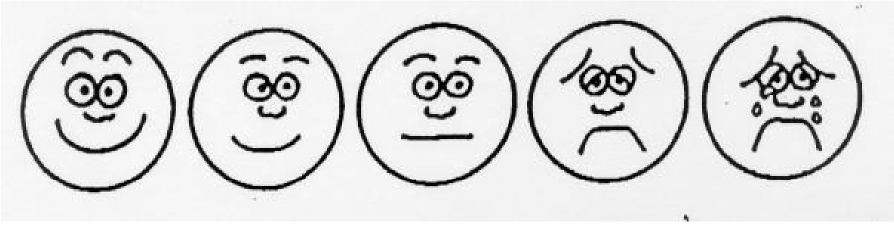

22 A. Bolezen vpliva na šolski uspeh Vašega otroka. Da Ne

Če ste označili “da”, potem nadaljujete z vprašanjem 22 B.

22 B. Kako se Vaš otrok počuti, ker bolezen vpliva na njegov šolski uspeh? Prosim označite obraz, ki najbolje izraža občutke Vašega otroka.

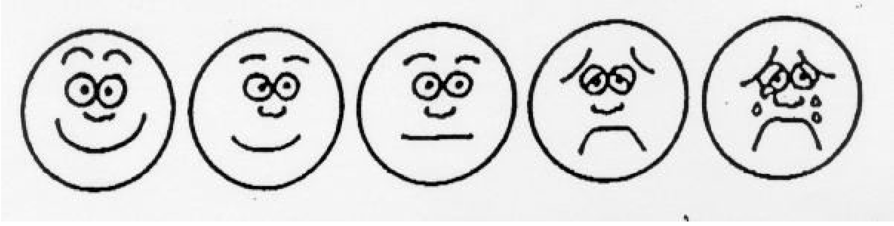

23 A. Vaš otrok zaradi bolezni manjka v šoli. Da Ne

Če ste označili “da”, potem nadaljujete z vprašanjem 23 B.

23 B. Kako se Vaš otrok počuti, ker zaradi bolezni manjka v šoli? Prosim označite obraz, ki najbolje izraža občutke Vašega otroka.

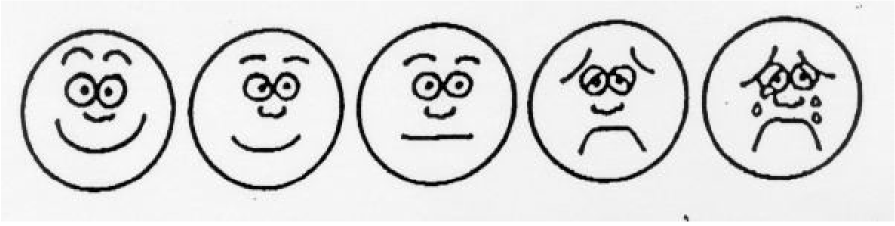

24 A. Bolezen vpliva na zunajšolske aktivnosti Vašega otroka. Da Ne

Če ste označili "da", potem nadaljujete z vprašanjem 24 B.
24 B. Kako se Vaš otrok počuti, ker bolezen vpliva na njegove zunajšolske aktivnosti? Prosim označite obraz, ki najbolje izraža občutke Vašega otroka.

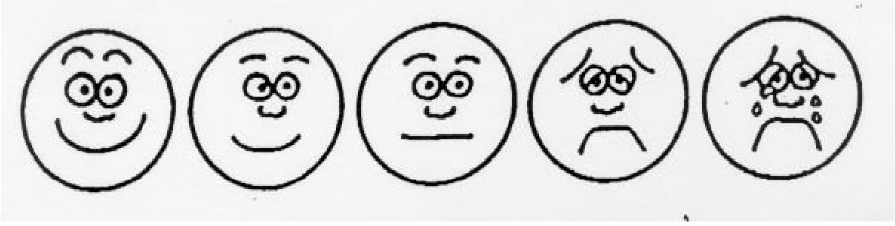

25 A. Bolezen vpliva na prijetne trenutke, ki jih Vaš otrok preživi skupaj s prijatelji. Da Ne

Če ste označili "da", potem nadaljujete z vprašanjem 25 B.
25 B. Kako se Vaš otrok počuti, ker bolezen vpliva na prijetne trenutke, ki jih preživi skupaj s prijatelji? Prosim označite obraz, ki najbolje izraža občutke Vašega otroka.

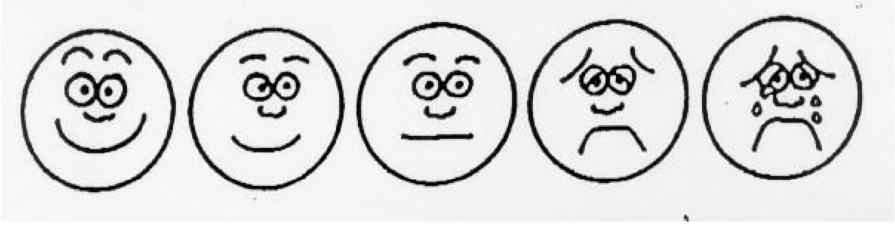

26 A. Bolezen vpliva na fizične aktivnosti Vašega otroka (kot npr. šolska telovadba, tek, hoja, igranje priljubljene igre na prostem) Da Ne

Če ste označili "da", potem nadaljujete z vprašanjem 26 B.
26 B. Kako se Vaš otrok počuti, ker bolezen vpliva na njegove fizične aktivnosti (kot npr. šolska telovadba, tek, hoja, igranje priljubljene igre na prostem)? Prosim označite obraz, ki najbolje izraža občutke Vašega otroka.

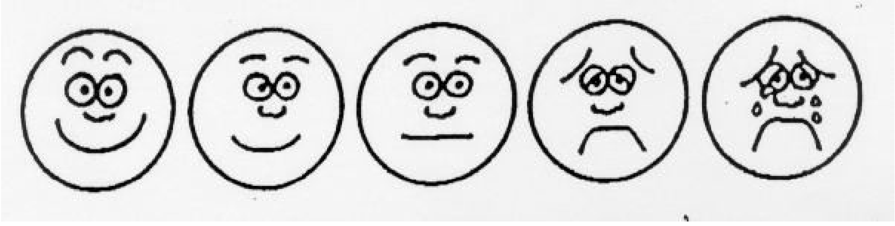

MEDIDA SIMPLE DEL IMPACTO DE LA ENFERMEDAD EN LOS NIÑOS
(SMILY-Illness) Versión para niños

Spanish argentina

Número de estudio __ Fecha __/__/20__

Para este cuestionario hemos seleccionado las 5 caras que siguen de la escala de puntuación de dolor Wong-Baker FACES, que hemos modificado con permiso de D.L. Wong. Las cinco caras aparecerán luego de cada pregunta.

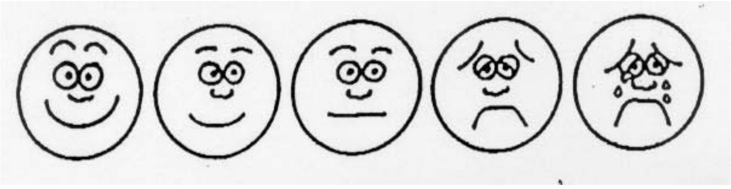

En Wong DL, Hockenberry-Eaton M, Wilson D,Winklestein ML, Schwartz P.: Wong's Essentials of Pediatric Nursing, ed. 6, St. Louis, 2001, Mosby, p 1301. Copyright Mosby. Reimpreso con permiso.

Los derechos de autor del SMILY-Illness pertenecen a L. Nandini Moorthy, MD MS,RWJM-UMDNJ, New Brunswick, NJ. Actualmente, el cuestionario solo puede ser usado por L. Nandini Moorthy, MD MS y personal de investigación designado.

PREGUNTAS

En este cuestionario, te vamos a hacer muchas preguntas sobre tu enfermedad y sobre cómo te sientes. No hay respuestas incorrectas. Por favor, pregúntanos lo que no entiendas. Si quieres escribir algún comentario, hazlo al final, después de las páginas 5 y 6.

Hay varias cosas que afectan la vida personal y que hacen que uno se sienta contento o triste. Hay cosas que son importantes para ti, que tú puedes hacer (o que no puedes hacer). Todas estas cosas forman parte de la calidad de vida de una persona.

1. ¿Cómo es tu calidad de vida? Por favor, marca con un círculo la cara que mejor indique cómo es tu calidad de vida.

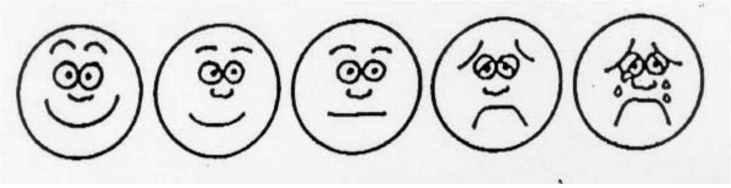

2. ¿Cómo está tu enfermedad en estos momentos? Por favor, marca con un círculo la cara que mejor indique cómo está tu enfermedad.

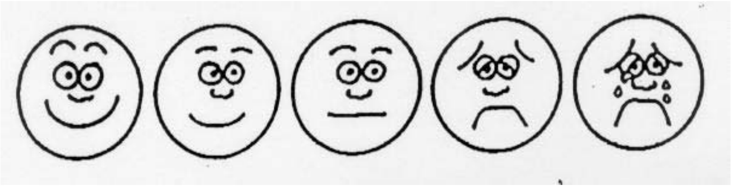

Copyright © 2007 Lakshmi Nandini Moorthy. Todos los derechos reservados.

Las preguntas 3-19 son acerca de cómo te has sentido en este último mes por causa de tu enfermedad. Por favor, marca con un círculo la cara que mejor indique cómo te sientes.

3. ¿Cómo te sientes por tener una enfermedad?

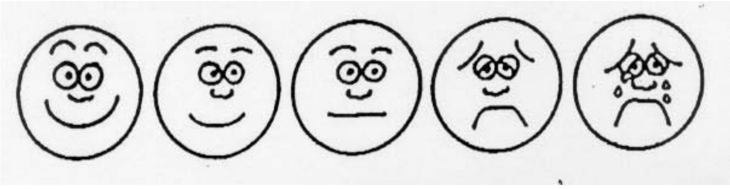

4. ¿Cómo hace una enfermedad que te sientas al ir a la escuela?

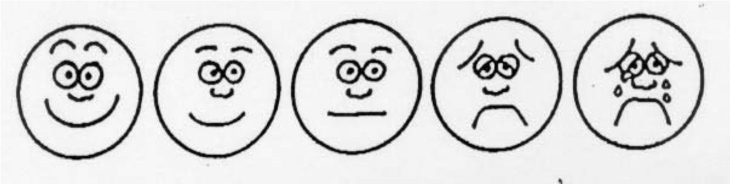

5. ¿Cómo hace una enfermedad que te sientas cuando te vistes cada día?

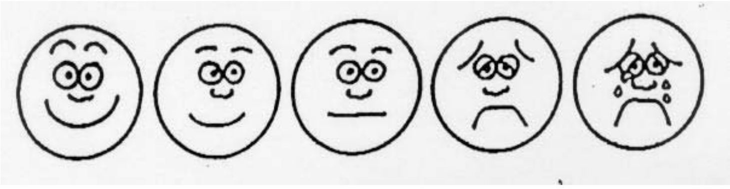

6. ¿Cómo te sientes acerca de como te ves por una enfermedad?

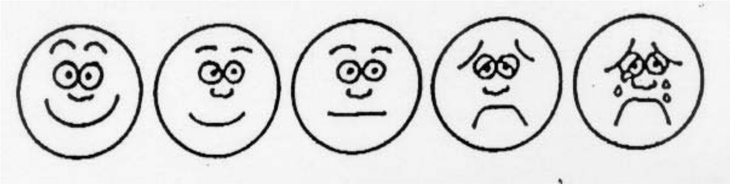

7. ¿Qué piensas de ti mismo por tener una enfermedad?

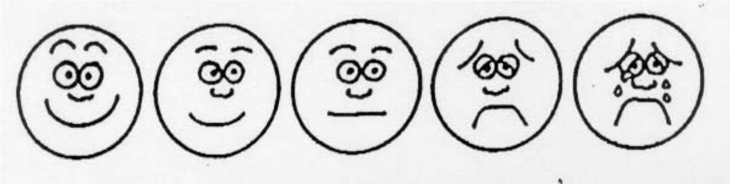

8. ¿Cómo te sientes cuando tienes que hablar de tu enfermedad?

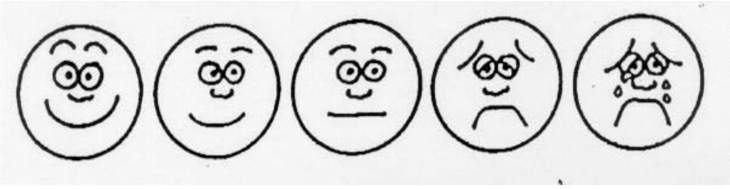

Copyright © 2007 Lakshmi Nandini Moorthy. Todos los derechos reservados.

9. ¿Cómo te sientes por tener que ir al médico por la enfermedad?

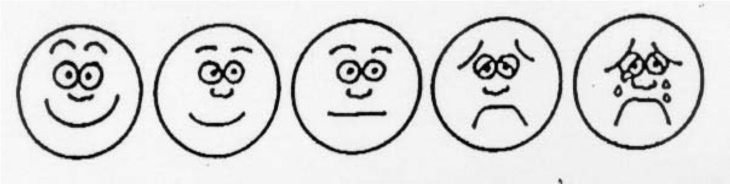

10. ¿Cómo te sientes por tener que ir al hospital por la enfermedad?

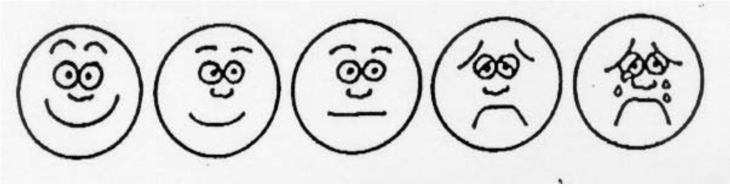

11. ¿Cómo hace la enfermedad que te sientas al pensar en tu futuro?

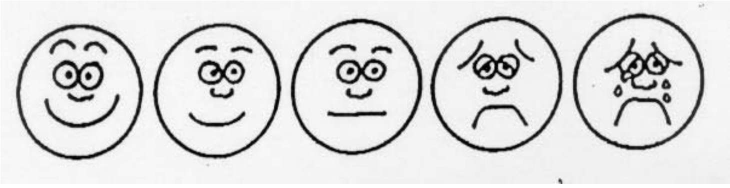

12. ¿Cómo te sientes al tomar medicinas por tu enfermedad?

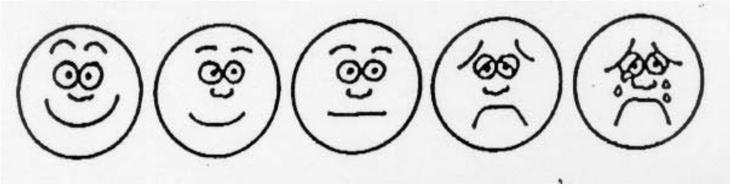

13. ¿Cuán preocupado/a estás por los efectos secundarios que puedan tener las medicinas que tomas para tu enfermedad?

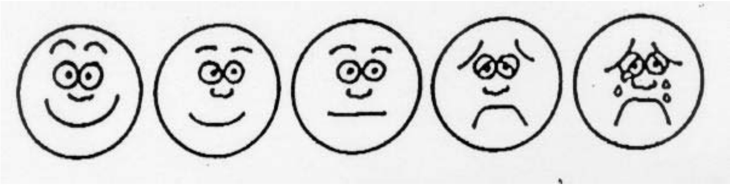

14. ¿Cuán preocupado/a estás de que tu enfermedad empeore?

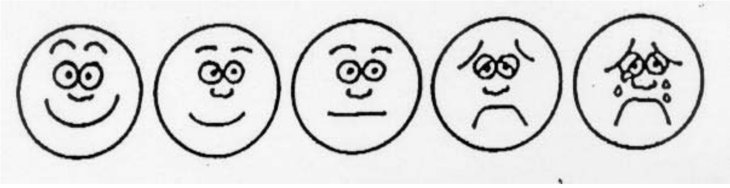

Copyright © 2007 Lakshmi Nandini Moorthy. Todos los derechos reservados.

15. ¿Cuán solo/a te sientes por tu enfermedad?

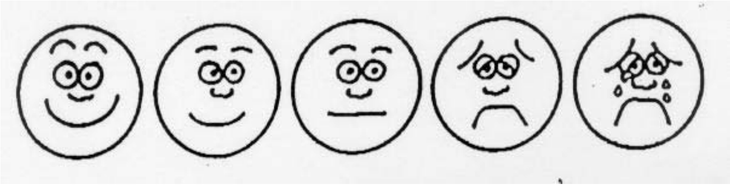

16. ¿Cómo te hace sentir tu familia?

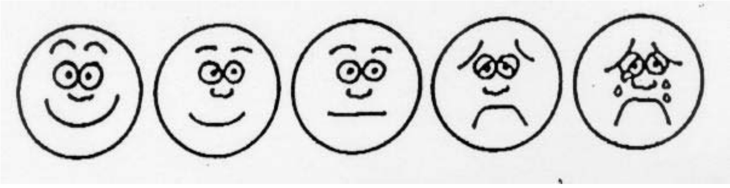

17. ¿Cómo te sientes por el dolor de tu enfermedad?

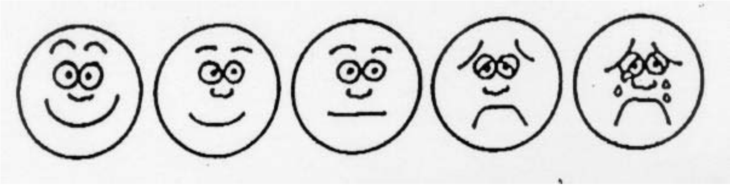

18. ¿Cómo te sientes por no poder salir al sol por tu enfermedad?

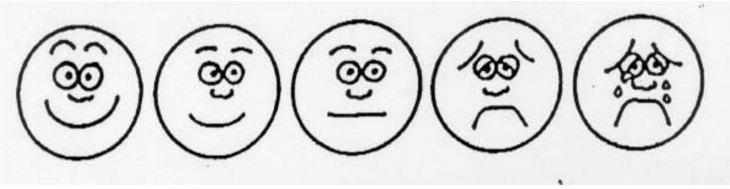

19. ¿Cuán cansado/a te sientes por tu enfermedad?

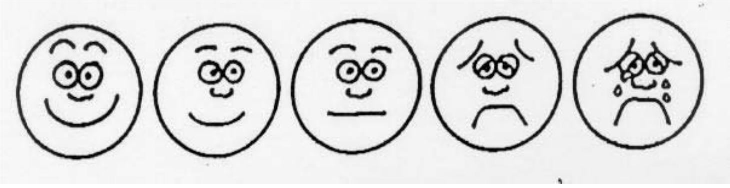

Copyright © 2007 Lakshmi Nandini Moorthy. Todos los derechos reservados.

Contesta "sí" o "no" a las preguntas 20 A-26 A, y luego sigue las instrucciones.

20 A. Mis amigos han cambiado porque tengo una enfermedad. Sí No

Si contestaste que "sí", pasa a la pregunta 20 B.

20 B. ¿Cómo te sientes acerca de tus amigos por tener la enfermedad? Por favor, marca con un círculo la cara que mejor indique cómo te sientes.

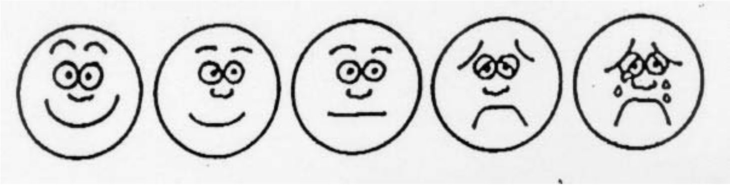

21 A. Mis amigos entienden que tengo una enfermedad Sí No

Si contestaste que "no", pasa a la pregunta 21 B.

21 B. ¿Cómo te sientes porque tus amigos no entienden que tienes una enfermedad? Por favor, marca con un círculo la cara que mejor indique cómo te sientes.

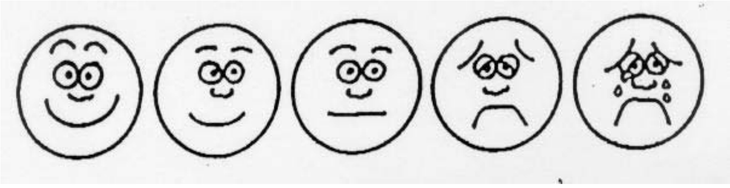

22 A. Mi enfermedad afecta mi trabajo en la escuela. Sí No

Si contestaste que "sí", pasa a la pregunta 22 B

22 B. ¿Cómo hace la enfermedad que te sientas con el trabajo de la escuela? Por favor, marca con un círculo la cara que mejor indique cómo te sientes.

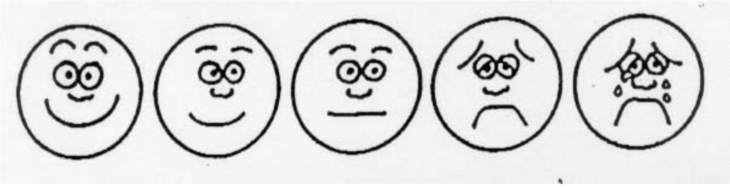

23 A. Falto a la escuela por mi enfermedad. Sí No

Si contestaste que "sí", pasa a la pregunta 23 B.

23 B. ¿Cómo hace que te sientas al faltar a la escuela por tener tu enfermedad? Por favor, marca con un círculo la cara que mejor indique cómo te sientes.

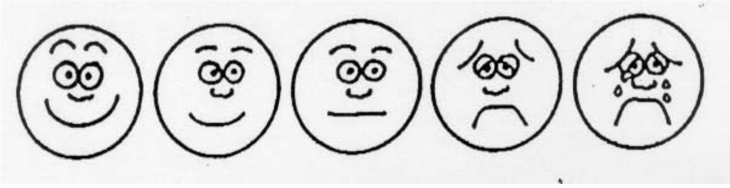

Copyright © 2007 Lakshmi Nandini Moorthy. Todos los derechos reservados.

24 A. Mi enfermedad afecta mis actividades fuera de la escuela. Sí No

Si contestaste que "sí", pasa a la pregunta 24 B.

24 B. ¿Cómo hace que te sientes el hecho de que tu enfermedad afecta tus actividades fuera de la escuela? Por favor, marca con un círculo la cara que mejor indique cómo te sientes.

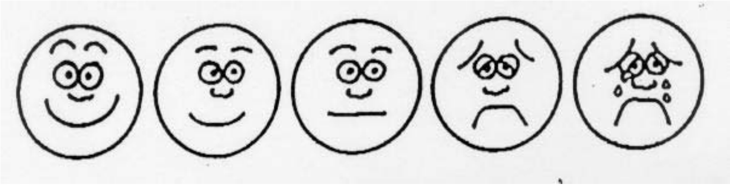

25 A. Mi enfermedad afecta como me divierto con mis amigos. Sí No

Si contestaste que "sí", pasa a la pregunta 25 B.

25 B. ¿Cómo hace que te sientas que tu enfermedad afecte como te diviertes con tus amigos? Por favor, marca con un círculo la cara que mejor indique cómo te sientes.

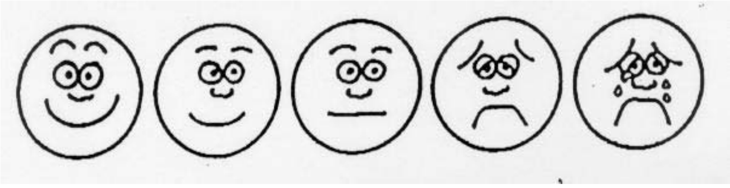

26 A. Mi enfermedad afecta mi participación en actividades físicas (como por ejemplo, hacer ejercicio en la escuela, correr, caminar, participar en mis juegos favoritos al aire libre).

Sí No

Si contestaste que "sí", pasa a la pregunta 26B.

26 B. ¿Cómo hace que te sientas el hecho de que tu enfermedad afecte tu participación en actividades físicas (como por ejemplo, hacer ejercicio en la escuela, correr, caminar, participar en mis juegos favoritos al aire libre)? Por favor, marca con un círculo la cara que mejor indique cómo te sientes.

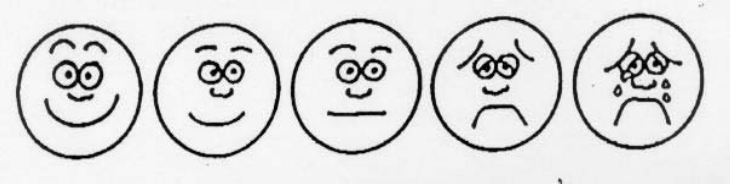

Copyright © 2007 Lakshmi Nandini Moorthy. Todos los derechos reservados.

MEDIDA SIMPLE DEL IMPACTO DE LA ENFERMEDAD EN LOS NIÑOS
(SMILY-Illness) – Versión para los padres

Número de estudio __ Fecha __/__/20_

Para este cuestionario hemos seleccionado las 5 caras que siguen de la escala de puntuación de dolor Wong-Baker FACES, que hemos modificado con permiso de D.L. Wong. Las cinco caras aparecerán luego de cada pregunta.

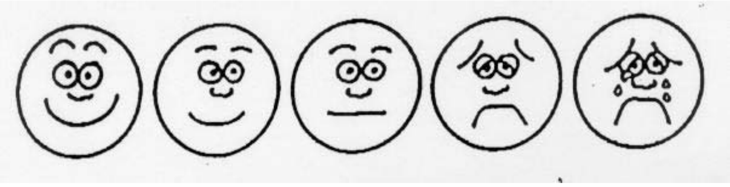

En Wong DL, Hockenberry-Eaton M, Wilson D,Winklestein ML, Schwartz P.: Wong's Essentials of Pediatric Nursing, ed. 6, St. Louis, 2001, Mosby, p 1301. Copyright Mosby. Reimpreso con permiso.

Los derechos de autor del SMILY-illness pertenecen a L. Nandini Moorthy, MD MS,RWJM-UMDNJ, New Brunswick, NJ. Actualmente, el cuestionario solo puede ser usado por L. Nandini Moorthy, MD MS y personal de investigación designado.

PREGUNTAS

En este cuestionario, le vamos a hacer muchas preguntas sobre la enfermedad y sobre cómo se siente su hijo/a. No hay respuestas incorrectas. Por favor, pregúntenos lo que no entienda. Si quiere escribir algún comentario, hágalo al final, después de las páginas 5 y 6.

Hay varias cosas que afectan la vida de su hijo y que hacen que se sienta contento/a o triste. Hay cosas que son importantes para su hijo/a, que él o ella puede hacer (o que no puede hacer). Todas estas cosas forman parte de la calidad de vida del niño.

1. ¿Cómo es la calidad de vida de su hijo/a? Por favor, marque con un círculo la cara que mejor indique cómo es su calidad de vida.

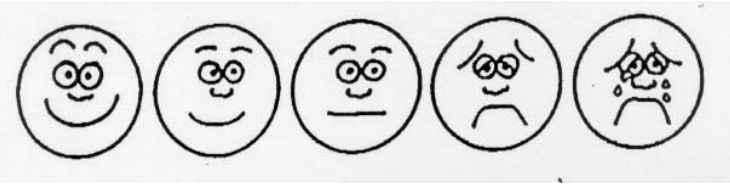

2. ¿Cómo va la enfermedad de su hijo/a en estos momentos? Por favor, marque con un círculo la cara que mejor indique cómo va la enfermedad de su hijo/a.

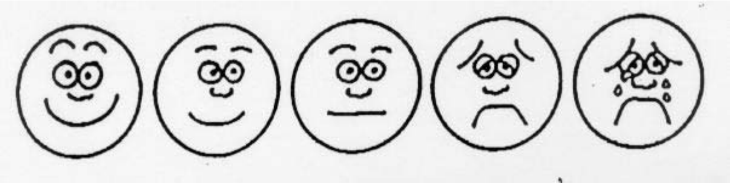

Copyright © 2007 Lakshmi Nandini Moorthy. Todos los derechos reservados.

Las preguntas 3-19 son acerca de cómo se ha sentido en este último mes por causa de una enfermedad de su hijo/a. Por favor, marque con un círculo la cara que mejor indique cómo se siente.

3. ¿Cómo se siente su hijo/a por tener una enfermedad?

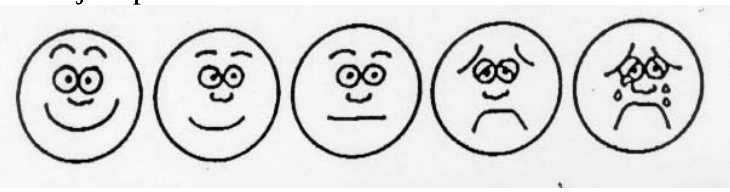

4. ¿Cómo hace la enfermedad que se sienta su hijo/a al ir a la escuela?

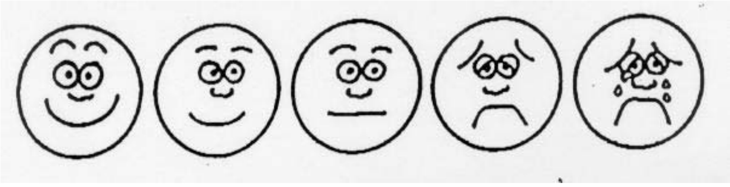

5. ¿Cómo hace la enfermedad que se sienta su hijo al tenerse que vestir cada día?

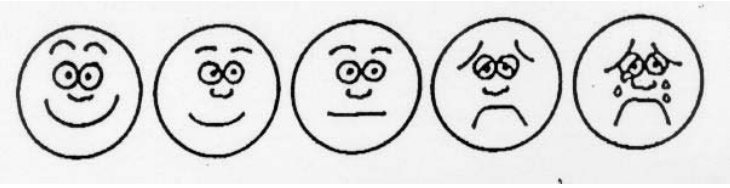

6. ¿Cómo se siente su hijo/a por el aspecto que tiene por su enfermedad?

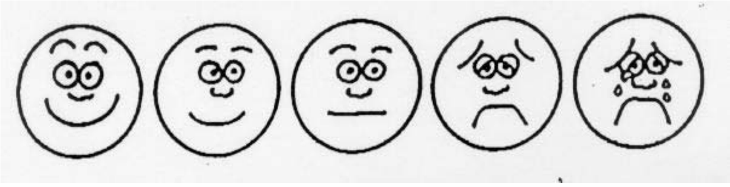

7. ¿Qué piensa su hijo/a de sí mismo/a por tener una enfermedad?

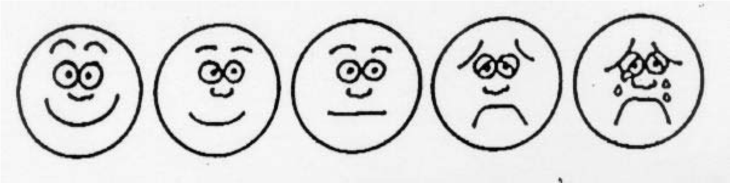

8. ¿Cómo se siente su hijo/a cuando tiene que hablar de su enfermedad?

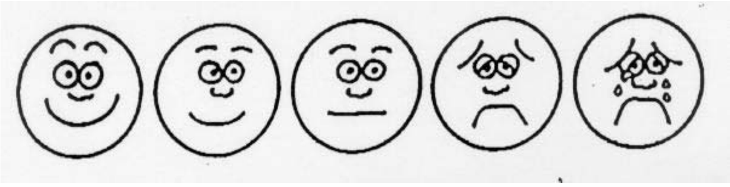

Copyright © 2007 Lakshmi Nandini Moorthy. Todos los derechos reservados.

9. ¿Cómo se siente su hijo/a por tener que ir al médico por su enfermedad?

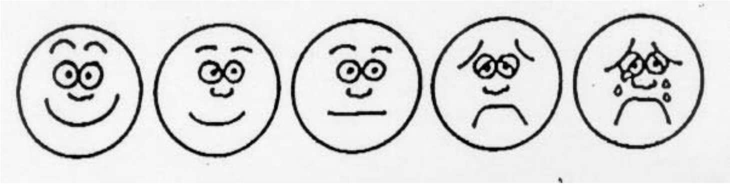

10. ¿Cómo se siente su hijo/a por tener que ir al hospital por su enfermedad?

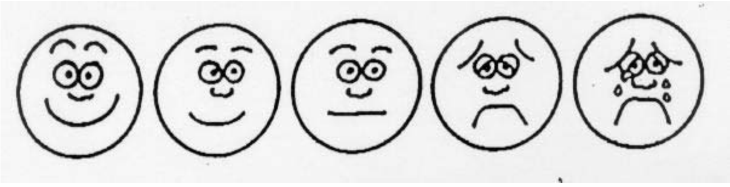

11. ¿Cómo hace la enfermedad que se sienta su hijo/a al pensar en el futuro?

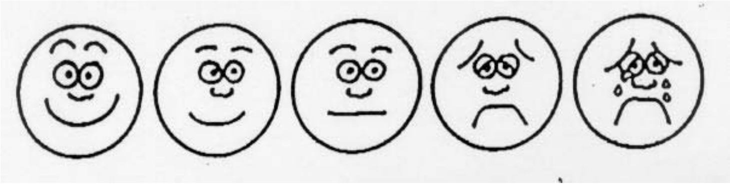

12. ¿Cómo se siente su hijo/a al tomar medicinas por su enfermedad?

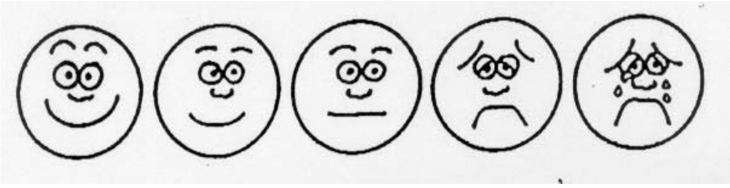

13. ¿Cuán preocupado/a está su hijo/a por los efectos secundarios que puedan tener las medicinas que toma para su enfermedad?

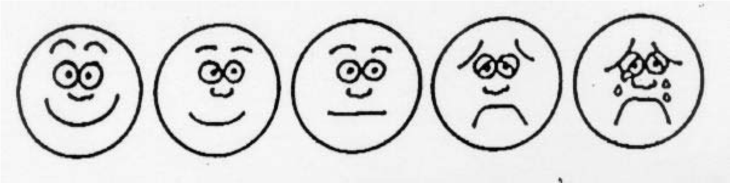

14. ¿Cuán preocupado/a está su hijo/a de que su enfermedad empeore?

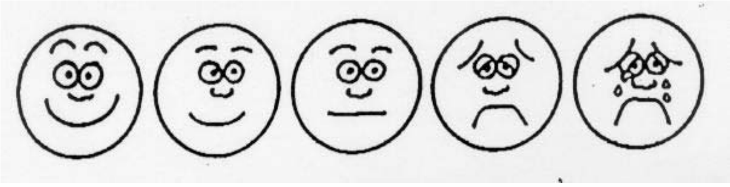

Copyright © 2007 Lakshmi Nandini Moorthy. Todos los derechos reservados.

15. ¿Cuán solo/a se siente su hijo/a por su enfermedad?

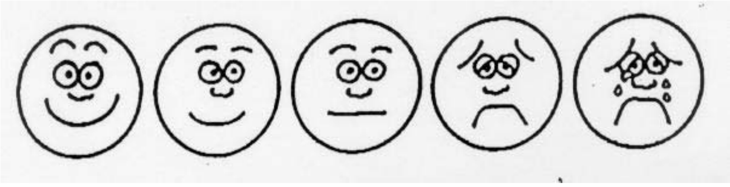

16. ¿Cómo hace sentir su familia a su hijo/a?

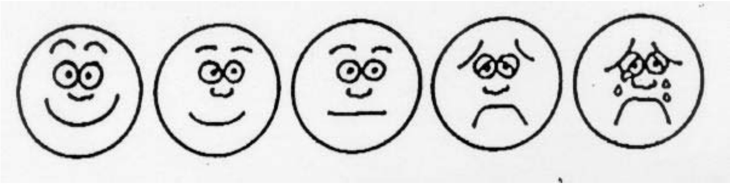

17. ¿Cómo se siente su hijo/a por el dolor de su enfermedad?

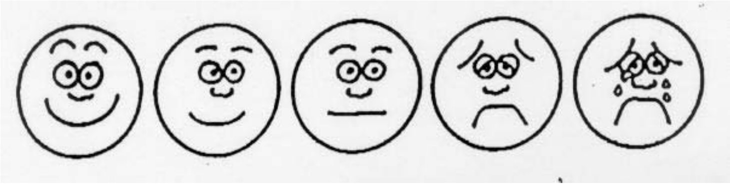

18. ¿Cómo se siente su hijo/a por el hecho de no poder salir al sol por su enfermedad?

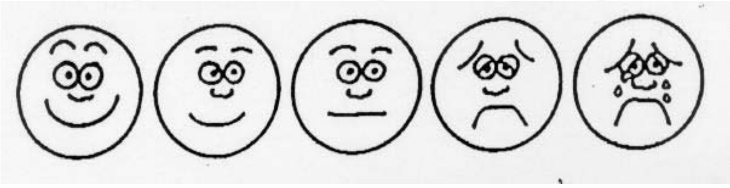

19. ¿Cuán cansado/a se siente su hijo/a por su enfermedad?

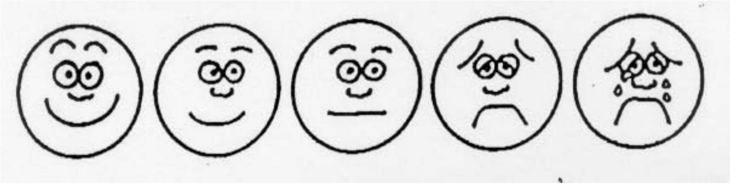

Conteste "sí" o "no" a las preguntas 20 A-26 A, y luego siga las instrucciones.

Copyright © 2007 Lakshmi Nandini Moorthy. Todos los derechos reservados.

20 A. Los amigos de su hijo/a han cambiado desde que tiene una enfermedad. Sí No

Si contestó que "sí", pase a la pregunta 20 B.

20 B. ¿Cómo hace que se sienta su hijo con sus amigos/as al tener una enfermedad? Por favor, marque con un círculo la cara que mejor indique cómo se siente su hijo/a.

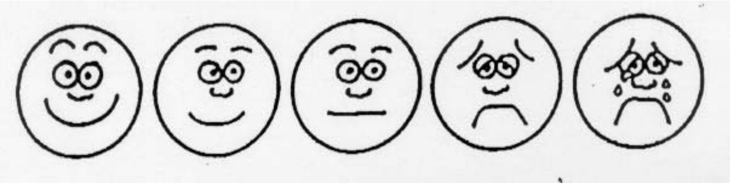

21 A. Los amigos/as de su hijo/a entienden que tiene una enfermedad. Sí No

Si contestó que "no", pase a la pregunta 21 B.

21 B. ¿Cómo hace que se sienta su hijo/a que sus amigos/as no entiendan que tiene una enfermedad? Por favor, marque con un círculo la cara que mejor indique cómo se siente su hijo/a.

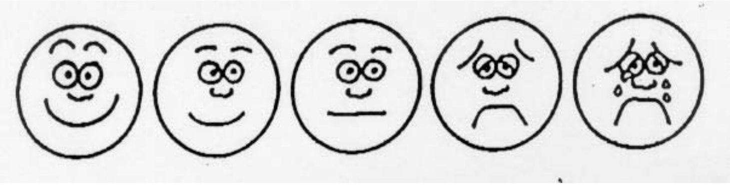

22 A. Una enfermedad afecta el trabajo de su hijo/a en la escuela. Sí No

Si contestó que "sí", pase a la pregunta 22 B

22 B. ¿Cómo hace una enfermedad que se sienta su hijo/a con el trabajo de la escuela? Por favor, marque con un círculo la cara que mejor indique cómo se siente su hijo/a.

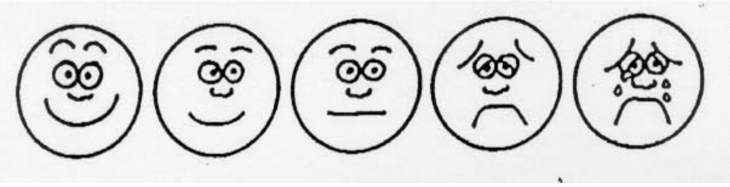

23 A. Su hijo/a falta a la escuela por su enfermedad. Sí No

Si contestó que "sí", pase a la pregunta 23 B.

23 B. ¿Cómo se siente su hijo/a al faltar a la escuela por su enfermedad? Por favor, marque con un círculo la cara que mejor indique cómo se siente su hijo/a.

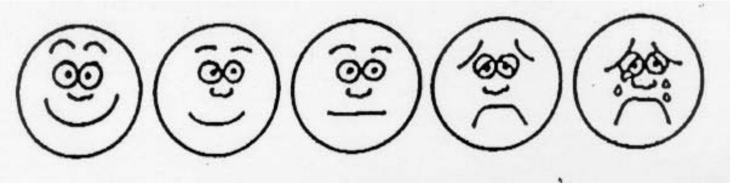

24 A. La enfermedad afecta las actividades de su hijo/a fuera de la escuela. Sí No

Si contestó que "sí", pase a la pregunta 24 B.

Copyright © 2007 Lakshmi Nandini Moorthy. Todos los derechos reservados.

24 B. ¿Cómo se siente su hijo/a por el hecho de que su enfermedad afecte sus actividades fuera de la escuela? Por favor, marque con un círculo la cara que mejor indique cómo se siente su hijo/a.

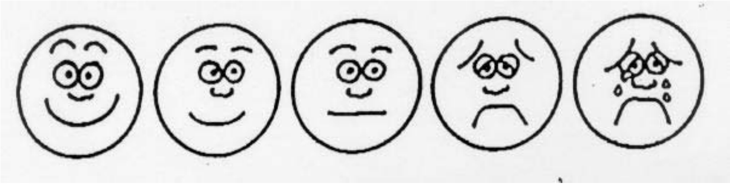

25 A. La enfermedad afecta como se divierte su hijo/a con sus amigos. Sí No

Si contestó que "sí", pase a la pregunta 25 B.

25 B. ¿Cómo se siente su hijo/a de que su enfermedad afecte como se divierte con sus amigos/as? Por favor, marque con un círculo la cara que mejor indique cómo se siente su hijo/a.

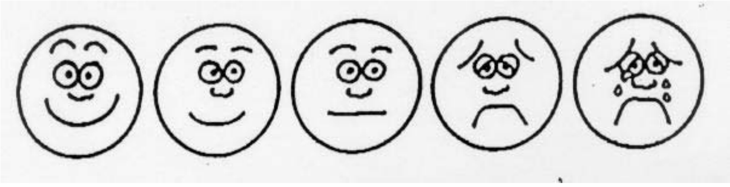

26 A. La enfermedad afecta la participación de su hijo/a en actividades físicas (como por ejemplo, hacer ejercicio en la escuela, correr, caminar, participar en sus juegos favoritos al aire libre).

Sí No

Si contestó que "sí", pase a la pregunta 26B.

26 B. ¿Cómo se siente su hijo/a de que su enfermedad afecte su participación en sus juegos favoritos al aire libre)? Por favor, marque con un círculo la cara que mejor indique cómo se siente su hijo/a.

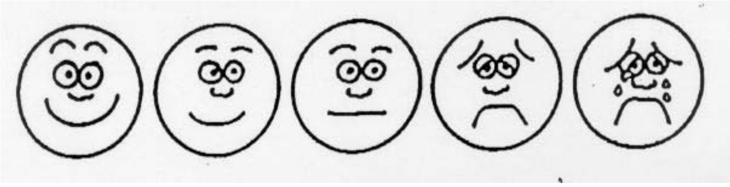

Copyright © 2007 Lakshmi Nandini Moorthy. Todos los derechos reservados.

MEDIDA SIMPLE DEL IMPACTO DE LA ENFERMEDAD EN LOS NIÑOS
(SMILY-Illness) – Versión para niños

Spanish mexi

Número de estudio __ Fecha __/__/20__

Para este cuestionario hemos seleccionado las 5 caras que de la escala de puntuación de dolor Wong-Baker FACES modificada con permiso de D.L. Wong. Después de cada pregunta, aparecen las cinco caras.

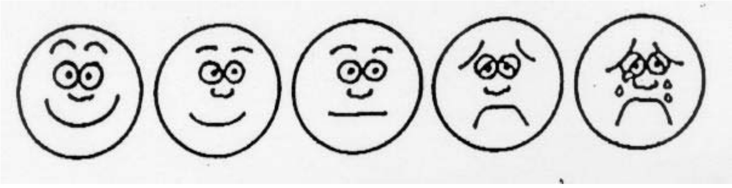

En Wong DL, Hockenberry-Eaton M, Wilson D,Winklestein ML, Schwartz P.: Wong's Essentials of Pediatric Nursing, ed. 6, St. Louis, 2001, Mosby, p 1301. Copyright Mosby. Reimpreso con permiso.

Los derechos de autor del SMILY-illness pertenecen a L. Nandini Moorthy, MD MS, RWJMS-UMDNJ, New Brunswick, NJ. El cuestionario, actualmente, solo puede ser usado por L. Nandini Moorthy, MD MS y el personal de investigación designado.

PREGUNTAS

En este cuestionario, te vamos a hacer muchas preguntas acerca de tu enfermedad y cómo te sientes. No hay respuestas incorrectas. Por favor, pregúntanos lo que no entiendas. Si quieres escribir algún comentario, hazlo al final, después de las páginas 5 y 6.

Hay varias cosas que afectan la vida de cada persona y que hacen que uno se sienta contento o triste. Hay cosas que son importantes para ti, que tú puedes hacer (o que no puedes hacer). Todas estas cosas forman parte de la calidad de vida de una persona.

1. ¿Cómo es tu calidad de vida? Por favor, marca con un círculo la cara que mejor indique cómo es tu calidad de vida.

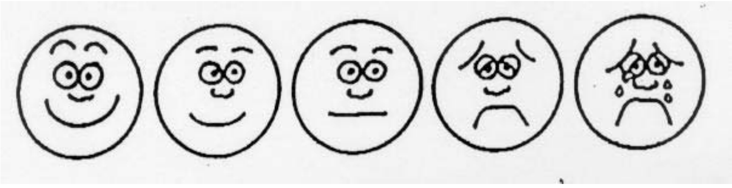

2. ¿Cómo estás por la enfermedad en estos momentos? Por favor, marca con un círculo la cara que mejor indique cómo estás por la enfermedad.

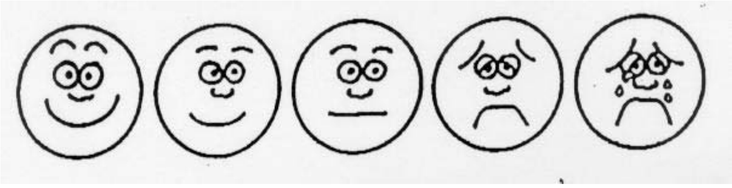

Derechos reservados © 2007 Lakshmi Nandini Moorthy. Todos los derechos reservados.

Las preguntas 3-19 son acerca de cómo te has sentido en este último mes por causa de la enfermedad. Por favor, marca con un círculo la cara que mejor indique cómo te sientes.

3. ¿Cómo te sientes por tener la enfermedad?

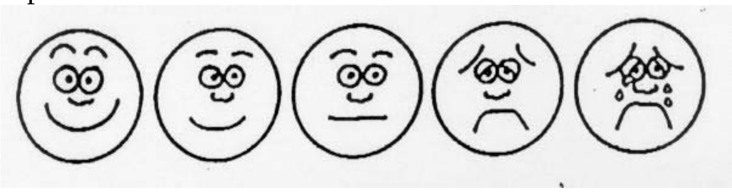

4. ¿Cómo te hace sentir la enfermedad al ir a la escuela?

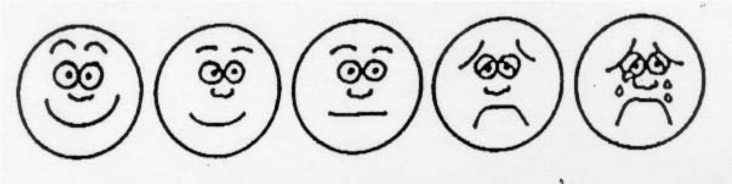

5. ¿Cómo te hace sentir la enfermedad al vestirte cada día?

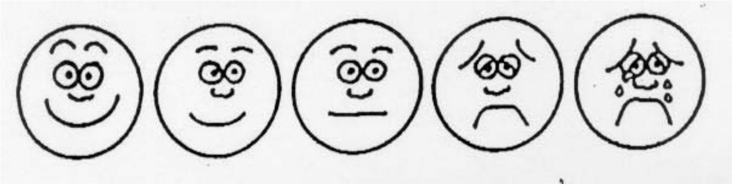

6. ¿Cómo te sientes acerca de como te ves por la enfermedad?

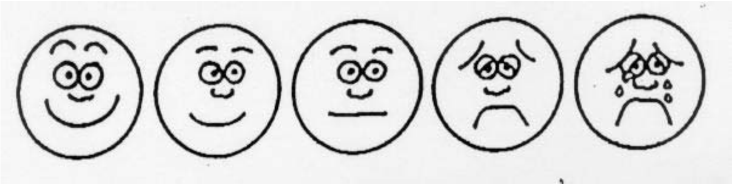

7. ¿Qué piensas de ti mismo por tener la enfermedad?

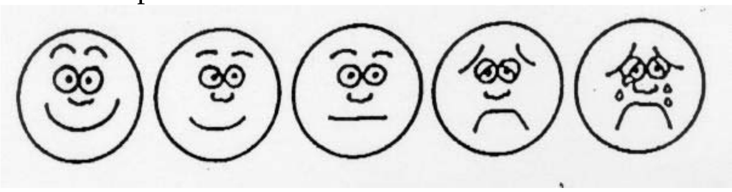

8. ¿Cómo te sientes cuando tienes que hablar de la enfermedad?

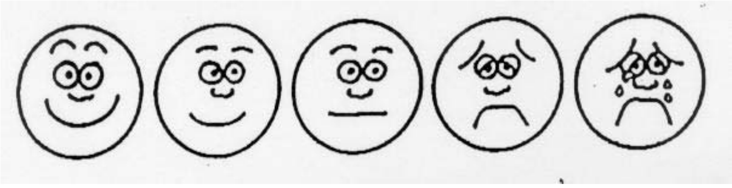

9. ¿Cómo te sientes por tener que ir al médico por la enfermedad?

Derechos reservados © 2007 Lakshmi Nandini Moorthy. Todos los derechos reservados.

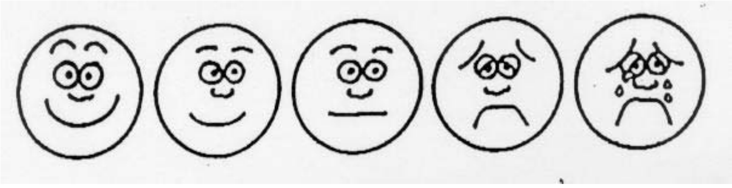

10. ¿Cómo te sientes por tener que ir al hospital por la enfermedad?

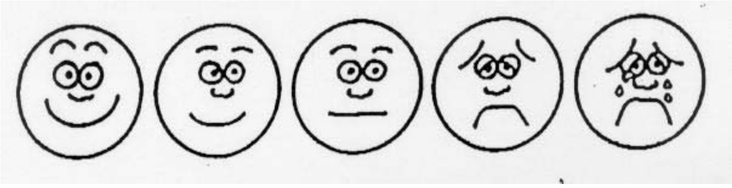

11. ¿ Cómo te hace sentir la enfermedad al pensar en tu futuro?

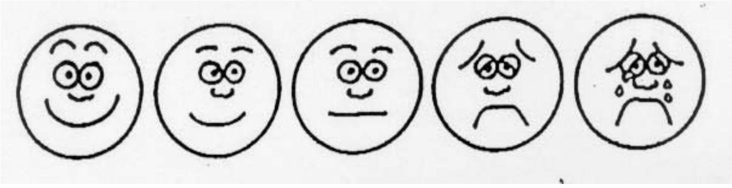

12. ¿Cómo te sientes al tomar medicinas por la enfermedad?

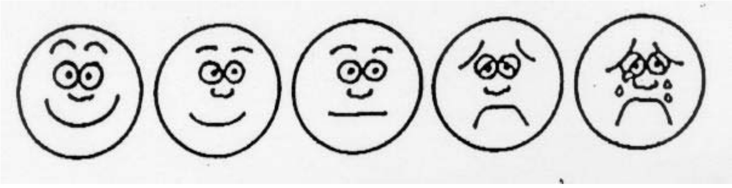

13. ¿Qué tan preocupado (a) estás por los efectos secundarios que pudieran tener las medicinas que tomas para la enfermedad?

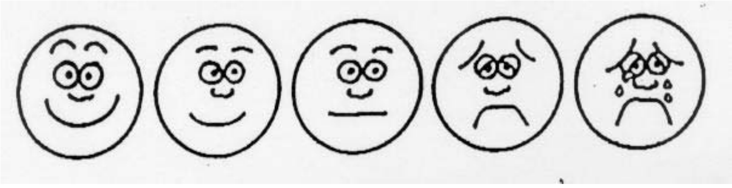

14. ¿Qué tan preocupado (a) estás de empeorar por la enfermedad?

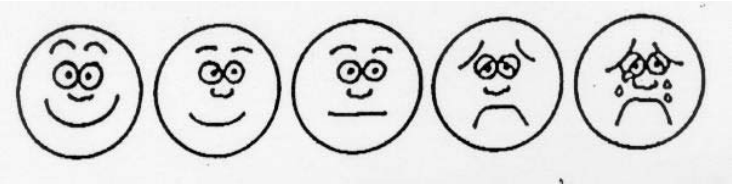

Derechos reservados © 2007 Lakshmi Nandini Moorthy. Todos los derechos reservados.

15. ¿Qué tan solo (a) te sientes por la enfermedad?

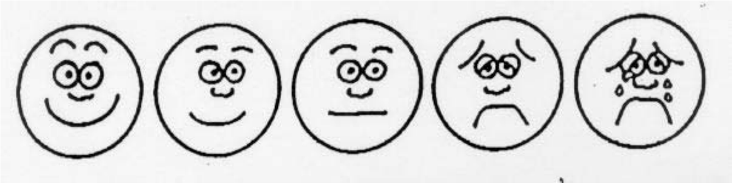

16. ¿Cómo te hace sentir tu familia?

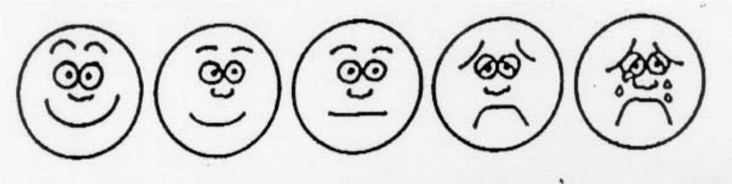

17. ¿Cómo te sientes por el dolor de la enfermedad?

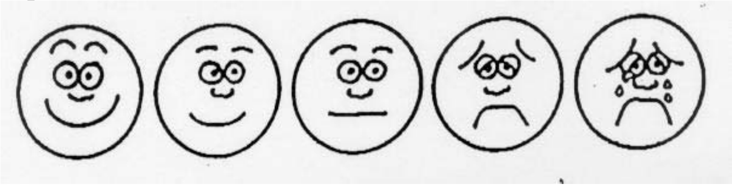

18. ¿Cómo te sientes por no poder salir al sol por la enfermedad?

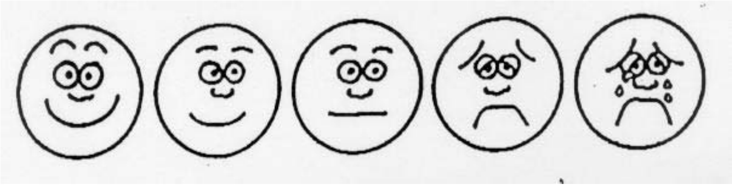

19. ¿Qué tan cansado (a) te sientes por la enfermedad?

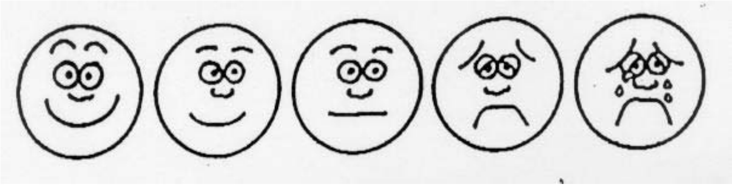

Derechos reservados © 2007 Lakshmi Nandini Moorthy. Todos los derechos reservados.

Contesta "sí" o "no" a las preguntas 20 A-26 A, y luego sigue las instrucciones.

20 A. Mis amigos han cambiado porque estoy enfermo. Sí No

Si contestaste que "sí", pasa a la pregunta 20 B.

20 B. ¿Cómo te sientes porque tus amigos cambiaron contigo al saber que tienes la enfermedad? Por favor, marca con un círculo la cara que mejor indique cómo te sientes.

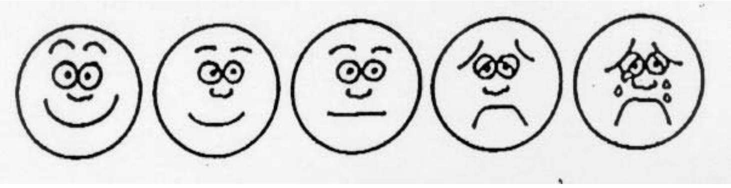

21 A. Mis amigos entienden que tengo una enfermedad Sí No

Si contestaste que "no", pasa a la pregunta 21 B.

21 B. ¿Cómo te sientes porque tus amigos no entienden que tienes una enfermedad? Por favor, marca con un círculo la cara que mejor indique cómo te sientes.

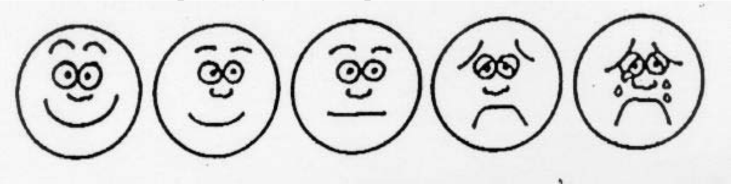

22 A. La enfermedad afecta mi trabajo en la escuela. Sí No

Si contestaste que "sí", pasa a la pregunta 22 B

22 B. ¿Cómo te hace sentir la enfermedad con tus tareas de la escuela? Por favor, marca con un círculo la cara que mejor indique cómo te sientes.

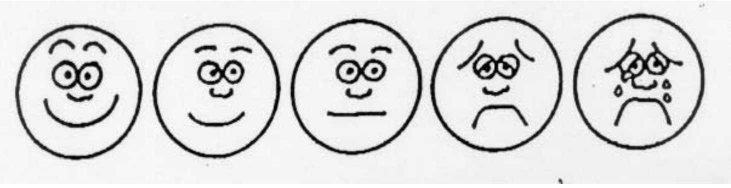

23 A. Falto a la escuela por la enfermedad. Sí No

Si contestaste que "sí", pasa a la pregunta 23 B.

23 B. ¿Cómo te hace sentir la enfermedad por faltar a la escuela? Por favor, marca con un círculo la cara que mejor indique cómo te sientes.

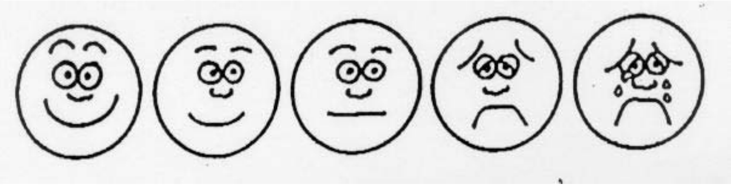

Derechos reservados © 2007 Lakshmi Nandini Moorthy. Todos los derechos reservados.

24 A. La enfermedad afecta mis actividades fuera de la escuela. Sí No

Si contestaste que "sí", pasa a la pregunta 24 B.

24 B. ¿Cómo te hace sentir la enfermedad por afectar tus actividades fuera de la escuela? Por favor, marca con un círculo la cara que mejor indique cómo te sientes

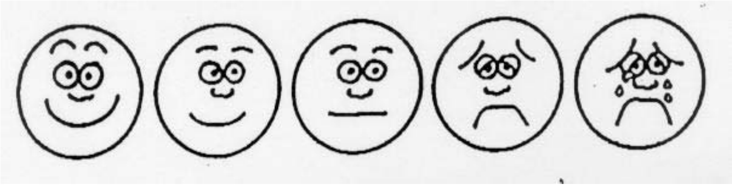

25 A. La enfermedad afecta como me divierto con mis amigos. Sí No

Si contestaste que "sí", pasa a la pregunta 25 B.

25 B. ¿Cómo te hace sentir la enfermedad por afectar como te diviertes con tus amigos? Por favor, marca con un círculo la cara que mejor indique cómo te sientes.

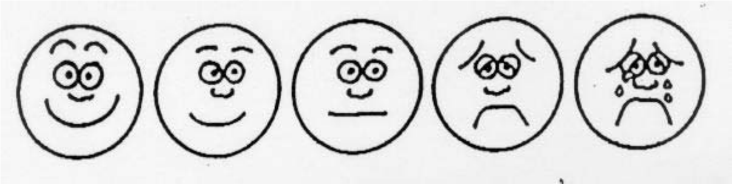

26 A. La enfermedad afecta mi participación en actividades físicas (como por ejemplo, hacer ejercicio en la escuela, correr, caminar, participar en mis juegos favoritos al aire libre).

Sí No

Si contestaste que "sí", pasa a la pregunta 26B.

26 B. ¿Cómo te hace sentir la enfermedad por afectar tu participación en actividades físicas (como por ejemplo, hacer ejercicio en la escuela, correr, caminar, participar en tus juegos favoritos al aire libre)? Por favor, marca con un círculo la cara que mejor indique cómo te sientes.

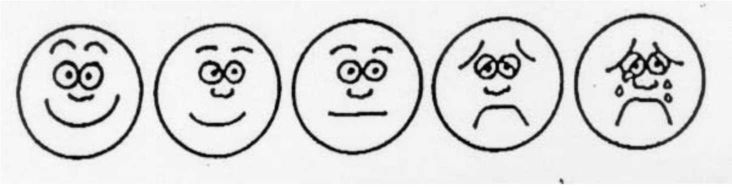

Derechos reservados © 2007 Lakshmi Nandini Moorthy. Todos los derechos reservados.

MEDIDA SIMPLE DEL IMPACTO DE LA ENFERMEDAD EN LOS NIÑOS
(SMILY-Illness) – Versión para los padres

Número de estudio __ Fecha __/__/20__

Para este cuestionario hemos seleccionado las 5 caras que siguen de la escala de puntuación de dolor Wong-Baker FACES, modificada con permiso de D.L. Wong. Después de cada pregunta aparecen las cinco caras.

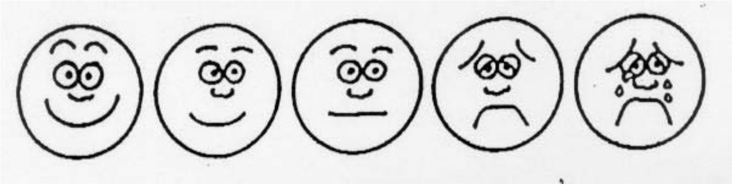

En Wong DL, Hockenberry-Eaton M, Wilson D,Winklestein ML, Schwartz P.: Wong's Essentials of Pediatric Nursing, ed. 6, St. Louis, 2001, Mosby, p 1301. Copyright Mosby. Reimpreso con permiso.

Los derechos de autor del SMILY-illness pertenecen a L. Nandini Moorthy, MD MS, RWJMS-UMDNJ, New Brunswick, NJ. El cuestionario, actualmente, solo puede ser usado por L. Nandini Moorthy, MD MS y el personal de investigación designado.

PREGUNTAS

En este cuestionario, le vamos a hacer muchas preguntas acerca de la enfermedad y sobre como se siente su hijo/a. No hay respuestas incorrectas. Por favor, pregúntenos lo que no entienda. Si quiere escribir algún comentario, hágalo al final, después de las páginas 5 y 6.

Hay varias cosas que afectan la vida de su hijo (a) y que hacen que se sienta contento (a) o triste. Hay cosas que son importantes para su hijo (a), que él o ella puede hacer (o que no puede hacer). Todas estas cosas forman parte de la calidad de vida del niño (a).

1. ¿Cómo es la calidad de vida de su hijo/a? Por favor, marque con un círculo la cara que mejor indique cómo es su calidad de vida.

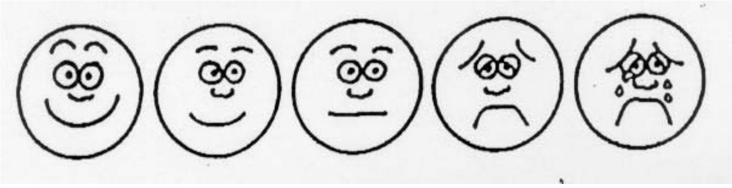

2. ¿Cómo ***está*** su hijo (a) por la enfermedad en estos momentos? Por favor marque con un círculo la cara que mejor indique como está su hijo (a) por la enfermedad

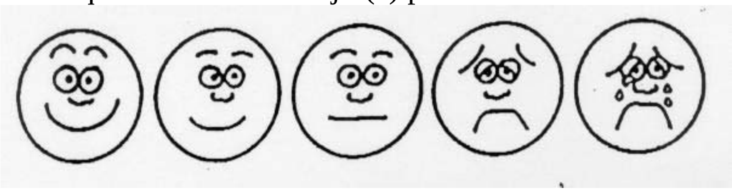

i

Derechos reservados © 2007 Lakshmi Nandini Moorthy. Todos los derechos reservados.

Las preguntas 3-19 se refieren al cómo se ha sentido su hijo (a) en este último mes a causa de la enfermedad. Por favor, marque con un círculo la cara que mejor indique cómo se siente.

3. ¿Cómo se siente su hijo (a) por tener la enfermedad?

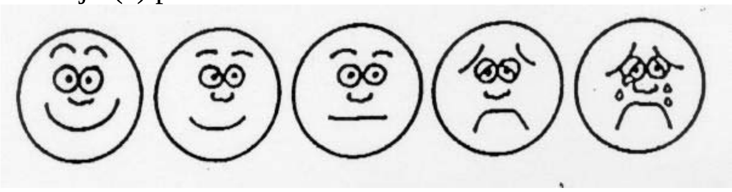

4. ¿Cómo se siente su hijo (a) por la enfermedad al ir a la escuela?

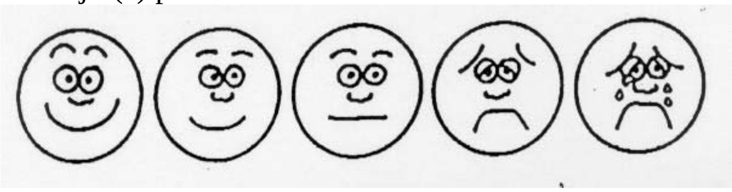

5. ¿Cómo se siente su hijo (a) por la enfermedad al vestirse cada día?

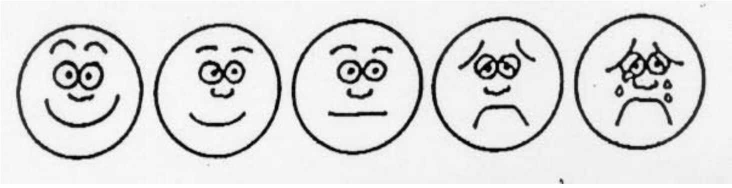

6. ¿ Cómo se siente su hijo (a)**,** por como se ve por la enfermedad?

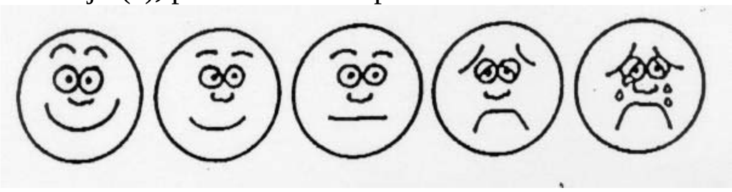

7. ¿Qué piensa su hijo (a) de sí mismo (a) por tener la enfermedad?

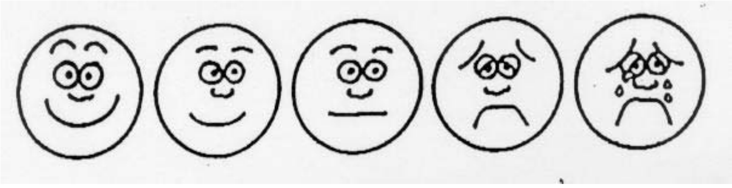

8. ¿Cómo se siente su hijo (a) cuando tiene que hablar de la enfermedad?

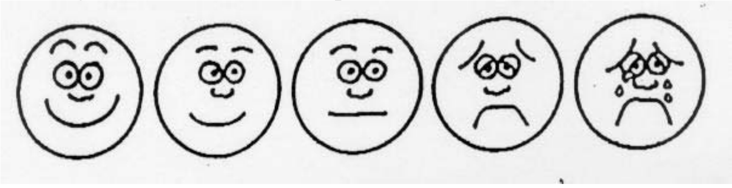

Derechos reservados © 2007 Lakshmi Nandini Moorthy. Todos los derechos reservados.

9. ¿Cómo se siente su hijo (a) por tener que ir al médico por la enfermedad?

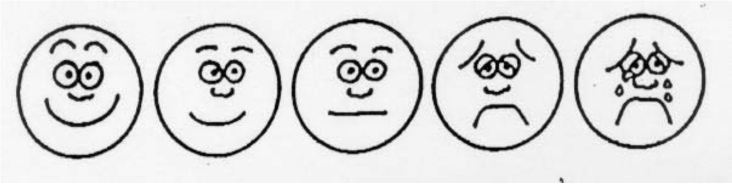

10. ¿Cómo se siente su hijo (a) por tener que ir al hospital por la enfermedad?

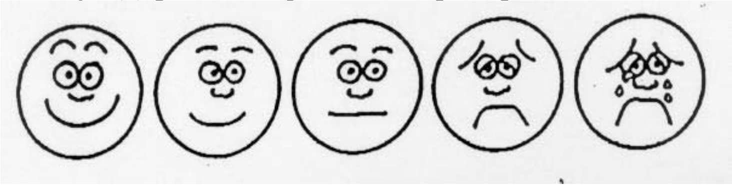

11. ¿Cómo se siente su hijo (a) al pensar en el futuro por la enfermedad?

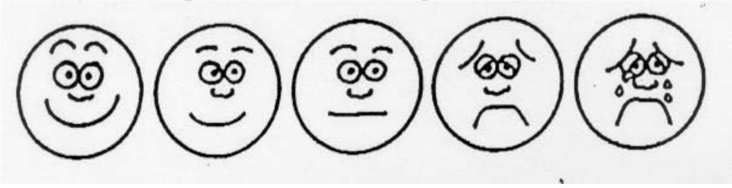

12. ¿Cómo se siente su hijo (a) al tomar medicinas por la enfermedad?

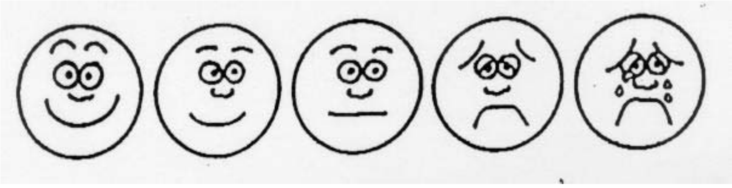

13. ¿Qué tan preocupado (a) está su hijo (a) por los efectos secundarios que pudieran tener las medicinas que toma para la enfermedad?

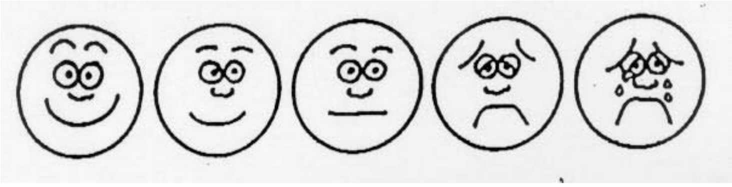

14. ¿Qué tan preocupado (a) está su hijo (a) de empeorar por la enfermedad?

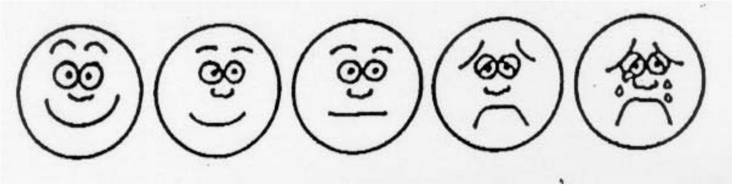

Derechos reservados © 2007 Lakshmi Nandini Moorthy. Todos los derechos reservados.

15. ¿Qué tan solo (a) se siente su hijo (a) por la enfermedad?

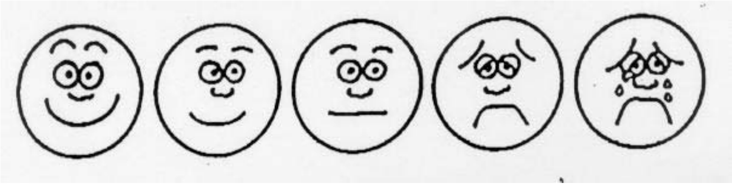

16. ¿ Cómo hace sentir su familia a su hijo (a)?

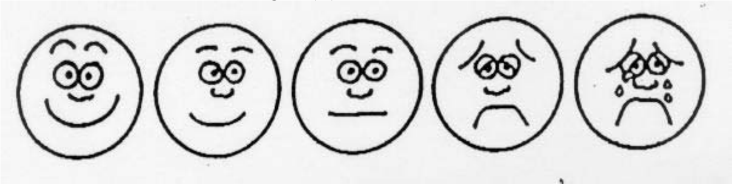

17. ¿Cómo se siente su hijo (a) por el dolor que le provoca la enfermedad?

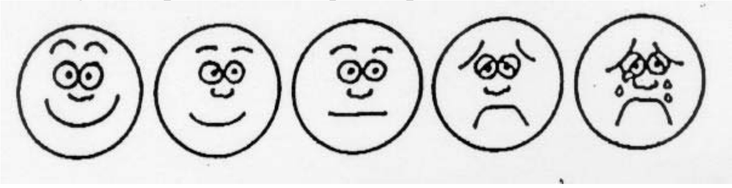

18. ¿Cómo se siente su hijo (a) por el hecho de no poder salir al sol por la enfermedad?

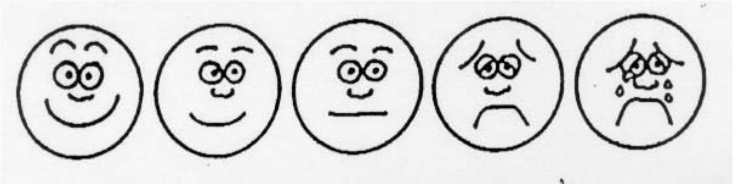

19. ¿Qué tan cansado (a) se siente su hijo (a) por la enfermedad?

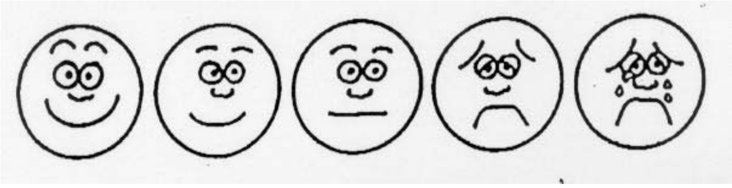

Derechos reservados © 2007 Lakshmi Nandini Moorthy. Todos los derechos reservados.

Conteste "sí" o "no" a las preguntas 20 A-26 A, y luego siga las instrucciones.

20 A. ¿Los amigos(as) de su hijo (a) han cambiado desde que está enfermo? Sí No

Si contestó que "sí", pase a la pregunta 20 B.

20 B. ¿Cómo se siente su hijo (a) por el cambio de sus amigos (as) al saber que tiene la enfermedad? Por favor, marque con un círculo la cara que mejor indique cómo se siente su hijo (a)

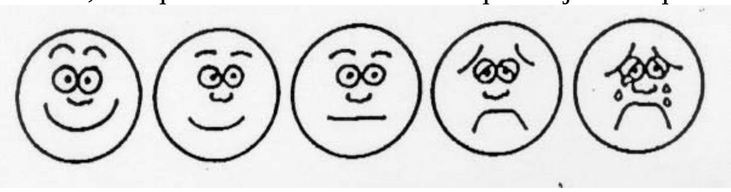

21 A. ¿Los amigos (as) de su hijo (a) entienden que tiene una enfermedad? Sí No

Si contestó que "no", pase a la pregunta 21 B.

21 B. ¿Cómo se siente su hijo(a) por que sus amigos (as) no entiendan que tiene la enfermedad? Por favor, marque con un círculo la cara que mejor indique cómo se siente su hijo (a)

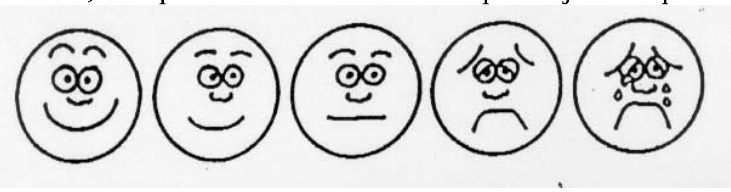

22 A. ¿La enfermedad afecta el trabajo de su hijo (a) en la escuela? Sí No

Si contestó que "sí", pase a la pregunta 22 B

22 B. ¿Cómo se siente su hijo (a) con las tareas de la escuela por la enfermedad? Por favor, marque con un círculo la cara que mejor indique cómo se siente su hijo (a)

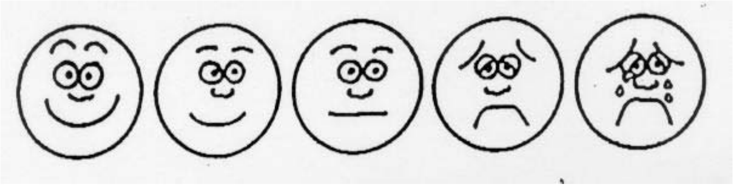

23 A. ¿Su hijo (a) falta a la escuela por la enfermedad? Sí No

Si contestó que "sí", pase a la pregunta 23 B.

23 B. ¿Cómo se siente su hijo/a al faltar a la escuela por la enfermedad?. Por favor, marque con un círculo la cara que mejor indique cómo se siente su hijo (a)

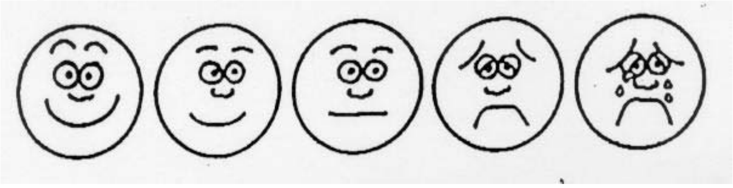

24 A. ¿La enfermedad afecta las actividades de su hijo (a) fuera de la escuela? Sí No

Derechos reservados © 2007 Lakshmi Nandini Moorthy. Todos los derechos reservados.

Si contestó que "sí", pase a la pregunta 24 B.

24 B. ¿Cómo se siente su hijo (a) por qué la enfermedad afecta sus actividades fuera de la escuela?. Por favor, marque con un círculo la cara que mejor indique como se siente su hijo (a)

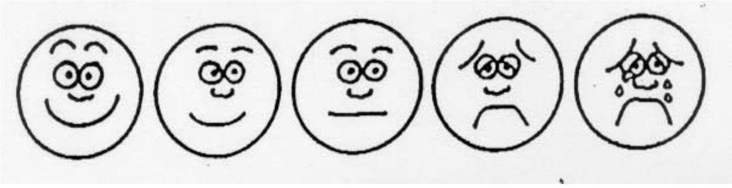

25 A. ¿La enfermedad afecta el como se divierte su hijo (a) con sus amigos? Sí No

Si contestó que "sí", pase a la pregunta 25 B.

25 B. ¿Cómo se siente su hijo (a) porque la enfermedad afecta como se divierte con sus amigos?. Por favor, marque con un círculo la cara que mejor indique cómo se siente su hijo (a)

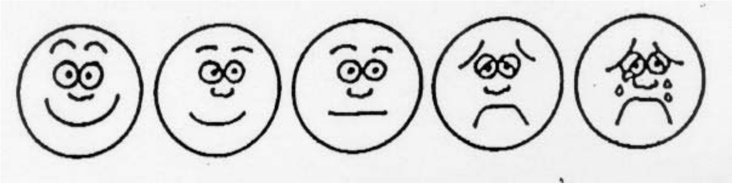

26 A. ¿La enfermedad afecta la participación de su hijo (a) en actividades físicas (por ejemplo, hacer ejercicio en la escuela, correr, caminar, participar en sus juegos favoritos al aire libre)?
Sí No

Si contestó que "sí", pase a la pregunta 26B.

26 B. ¿Cómo se siente su hijo (a) por que la enfermedad afecta su participación en sus juegos favoritos al aire libre?. Por favor, marque con un círculo la cara que mejor indique cómo se siente su hijo (a).

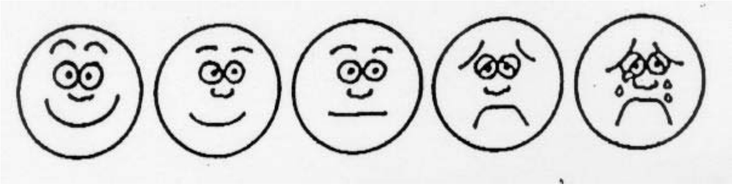

---
i

Derechos reservados © 2007 Lakshmi Nandini Moorthy. Todos los derechos reservados.

MEDICIÓN RAPIDA DEL IMPACTO DE LA ENFERMEDAD EN LOS NIÑOS
(SMILY-Illness) – Versión para niños Spanish-Spain

Número de estudio __ Fecha __/__/20_

Para este cuestionario hemos seleccionado las 5 caras que siguen de la escala de puntuación del dolor Wong-Baker FACES, que hemos modificado con permiso de D.L. Wong. Las cinco caras aparecerán a continuación de cada pregunta.

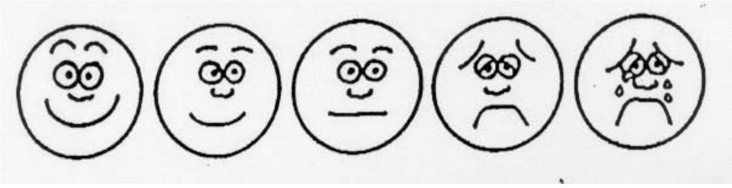

En Wong DL, Hockenberry-Eaton M, Wilson D,Winklestein ML, Schwartz P.: Wong's Essentials of Pediatric Nursing, ed. 6, St. Louis, 2001, Mosby, p 1301. Copyright Mosby. Reimpreso con permiso.

Los derechos de autor del SMILY-Illness pertenecen a L. Nandini Moorthy, MD MS, RWJM-UMDNJ, New Brunswick, NJ. En este punto, el cuestionario sólo puede ser usado por L. Nandini Moorthy, MD MS y el personal de investigación que designe.

PREGUNTAS

En este cuestionario, te vamos a hacer muchas preguntas sobre tu enfermedad y sobre cómo te sientes. No hay malas respuestas. Por favor, pregúntanos lo que no entiendas. Si quieres escribir algún comentario, hazlo al final, después de las páginas 5 y 6.

Hay varias cosas que afectan la vida personal y que hacen que uno se sienta contento o triste. Hay cosas que son importantes para ti, que tú puedes hacer (o que no puedes hacer). Todas estas cosas forman parte de la calidad de vida de una persona.

1. ¿Cómo es tu calidad de vida? Por favor, marca con un círculo la cara que mejor indique cómo es tu calidad de vida.

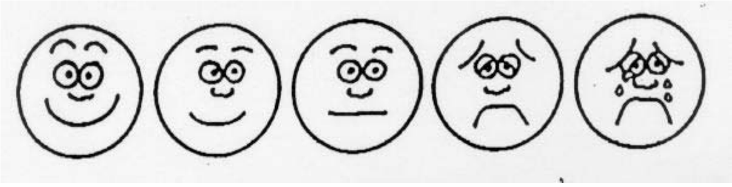

2. ¿Cómo va tu enfermedad en estos momentos? Por favor, marca con un círculo la cara que mejor indique cómo va tu enfermedad.

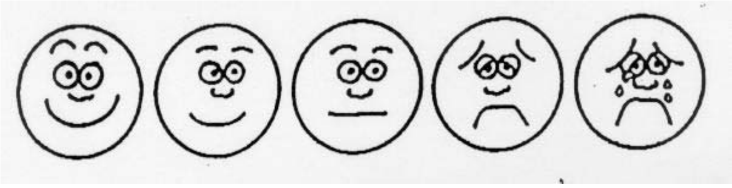

Copyright © 2007 Lakshmi Nandini Moorthy. Todos los derechos reservados.

Las preguntas 3-19 son acerca de cómo te has sentido en este último mes por a causa de la enfermedad. Por favor, marca con un círculo la cara que mejor indique cómo te sientes.

3. ¿Cómo te sientes por tener una enfermedad?

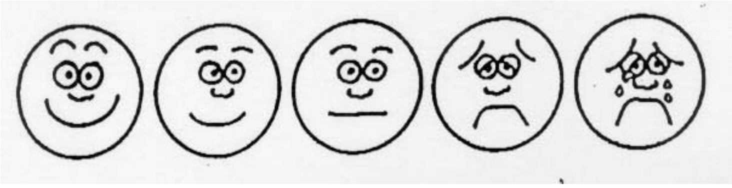

4. ¿Cómo hace la enfermedad que te sientas al ir a la escuela?

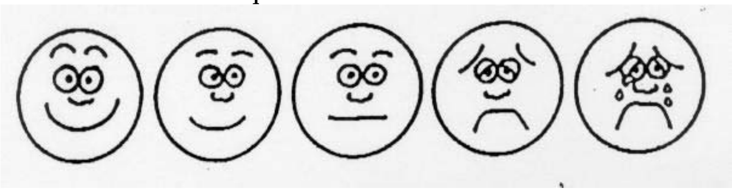

5. ¿Cómo hace la enfermedad que te sientas cuando te vistes cada día?

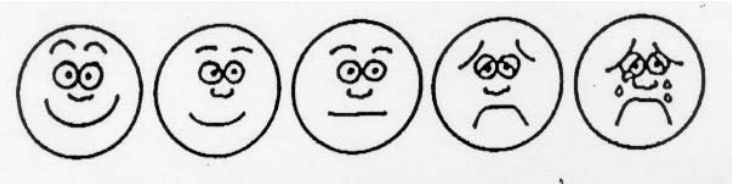

6. ¿Cómo te sientes con la enfermedad por el aspecto que tienes?

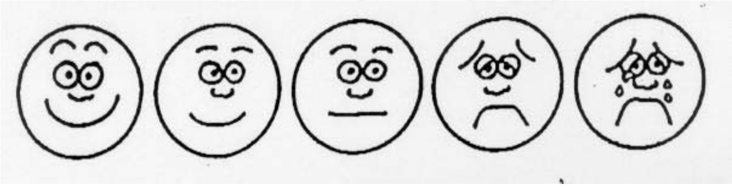

7. ¿Qué piensas de ti mismo por tener la enfermedad?

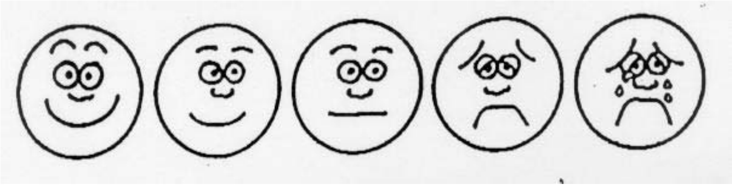

Copyright © 2007 Lakshmi Nandini Moorthy. Todos los derechos reservados.

8. ¿Cómo te sientes cuando tienes que hablar de la enfermedad?

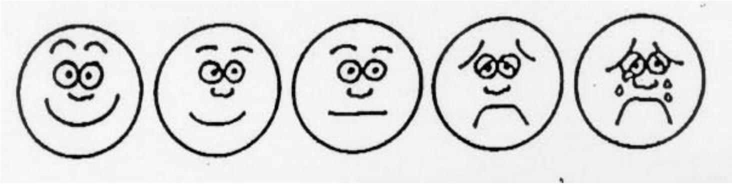

9. ¿Cómo te sientes por tener que ir al médico por la enfermedad?

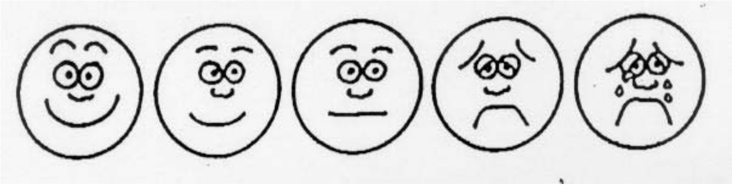

10. ¿Cómo te sientes por tener que ir al hospital por la enfermedad?

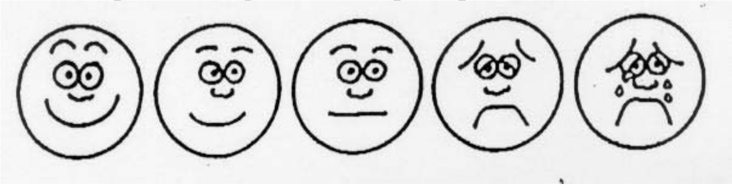

11. ¿Cómo hace la enfermedad que te sientas al pensar en tu futuro?

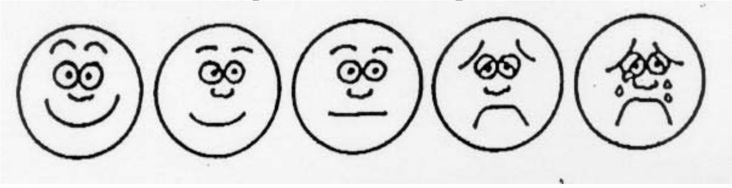

12. ¿Cómo te sientes al tomar medicamentos por la enfermedad?

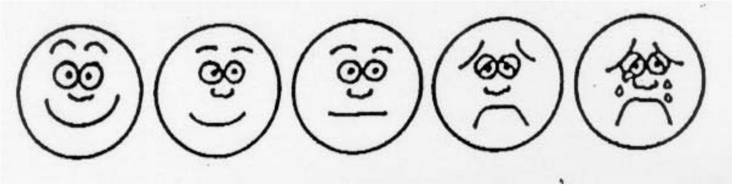

Copyright © 2007 Lakshmi Nandini Moorthy. Todos los derechos reservados.

13. ¿Como de preocupado estás por los efectos secundarios que puedan tener las medicamentos que tomas para la enfermedad?

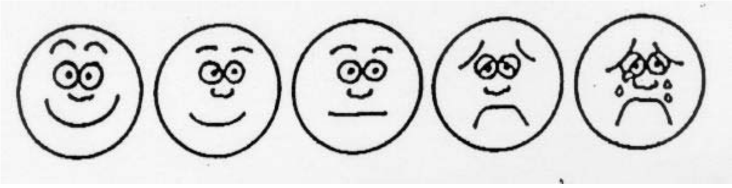

14. ¿Cómo de preocupado estás por si tu enfermedad empeora?

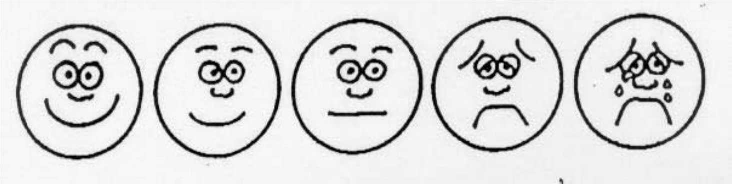

15. ¿Come de solo te sientes por la enfermedad?

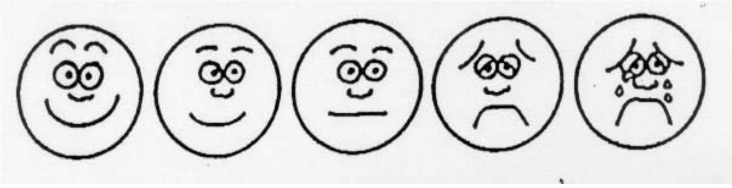

16. ¿Cómo te hace sentir tu familia?

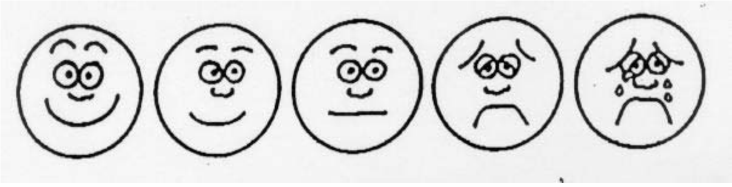

17. ¿Cómo hace que te sientas el dolor de la enfermedad?

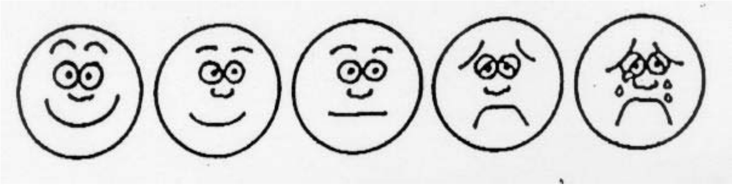

18. ¿Cómo te sientes por el hecho de no poder estar el sol por la enfermedad?

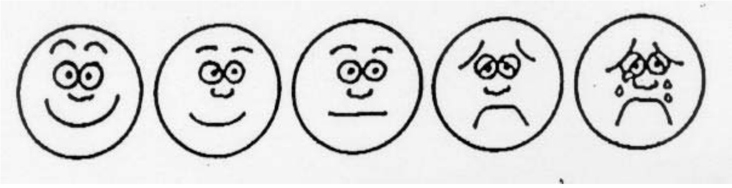

Copyright © 2007 Lakshmi Nandini Moorthy. Todos los derechos reservados.

19. ¿Cuán cansado/a te sientes por la enfermedad?

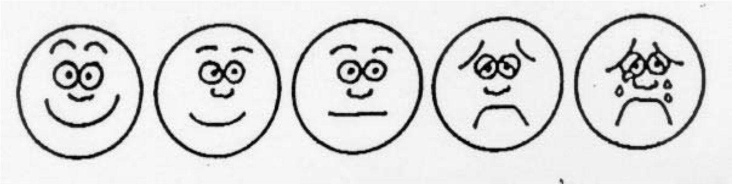

Contesta "sí" o "no" a las preguntas 20 A-26 A, y luego sigue las instrucciones.

20 A. Mis amigos han cambiado por la enfermedad. Sí No

Si contestaste que "sí", pasa a la pregunta 20 B.

20 B. ¿Cómo te sientes con tus amigos por tener la enfermedad? Por favor, marca con un círculo la cara que mejor indique cómo te sientes.

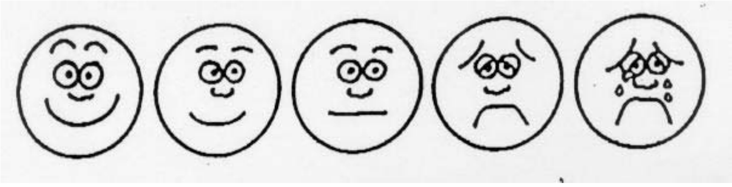

21 A. Mis amigos entienden que tengo la enfermedad Sí No

Si contestaste que "no", pasa a la pregunta 21 B.

21 B. ¿Cómo hace que te sientas que tus amigos no entiendan que tienes la enfermedad? Por favor, marca con un círculo la cara que mejor indique cómo te sientes.

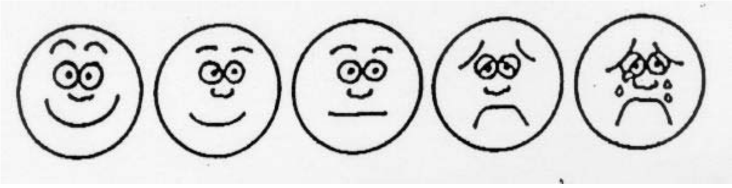

22 A. La enfermedad afecta mi trabajo en la escuela. Sí No

Si contestaste que "sí", pasa a la pregunta 22 B

22 B. ¿Cómo hace la enfermedad que te sientas con el trabajo de la escuela? Por favor, marca con un círculo la cara que mejor indique cómo te sientes.

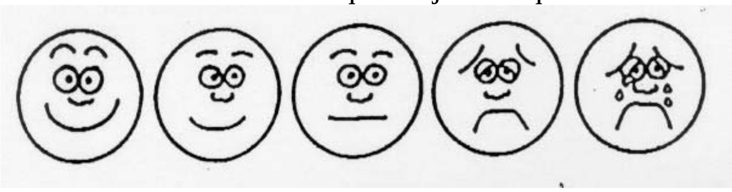

Copyright © 2007 Lakshmi Nandini Moorthy. Todos los derechos reservados.

23 A. Falto a la escuela por la enfermedad. Sí

No

Si contestaste que "sí", pasa a la pregunta 23 B.

23 B. ¿Cómo hace que te sientas el faltar a la escuela por la enfermedad? Por favor, marca con un círculo la cara que mejor indique cómo te sientes.

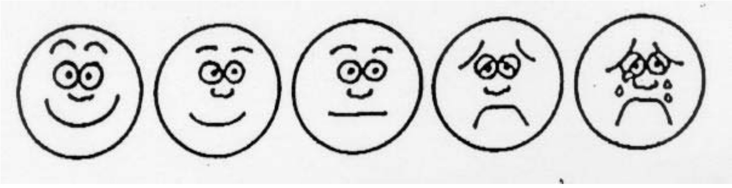

24 A. La enfermedad afecta mis actividades de fuera de la escuela. Sí

No

Si contestaste que "sí", pasa a la pregunta 24 B.

24 B. ¿Cómo hace que te sientas que la enfermedad afecte tus actividades de fuera de la escuela? Por favor, marca con un círculo la cara que mejor indique cómo te sientes.

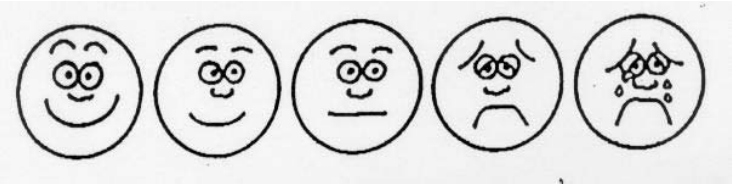

25 A. La enfermedad afecta la diversión que tengo con mis amigos. Sí

No

Si contestaste que "sí", pasa a la pregunta 25 B.

25 B. ¿Cómo hace que te sientas que la enfermedad afecte la diversión que tienes con tus amigos? Por favor, marca con un círculo la cara que mejor indique cómo te sientes.

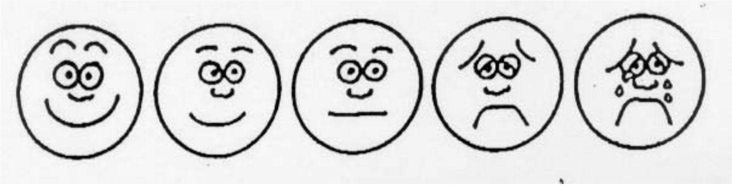

Copyright © 2007 Lakshmi Nandini Moorthy. Todos los derechos reservados.

26 A. La enfermedad afecta mi participación en actividades físicas (como por ejemplo, hacer ejercicio en la escuela, correr, caminar, jugar a mis juegos favoritos al aire libre). Sí No

Si contestaste que "sí", pasa a la pregunta 26B.

26 B. ¿Cómo hace que te sientas el hecho de que la enfermedad afecte tu participación en actividades físicas (como por ejemplo, hacer ejercicio en la escuela, correr, caminar, jugar a tus juegos favoritos de aire libre)? Por favor, marca con un círculo la cara que mejor indique cómo te sientes.

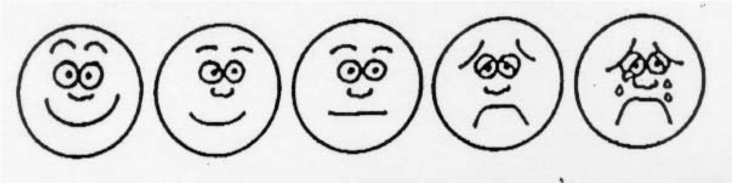

Copyright © 2007 Lakshmi Nandini Moorthy. Todos los derechos reservados.

MEDICIÓN RAPIDA DEL IMPACTO DE LA ENFERMEDAD EN LOS NIÑOS (SMILY-Illness) – Versión para los padres

Número de estudio __ Fecha __/__/20__

Para este cuestionario hemos seleccionado las 5 caras que siguen de la escala de puntuación del dolor Wong-Baker FACES, que hemos modificado con permiso de D.L. Wong. Las cinco caras aparecerán a continuación de cada pregunta.

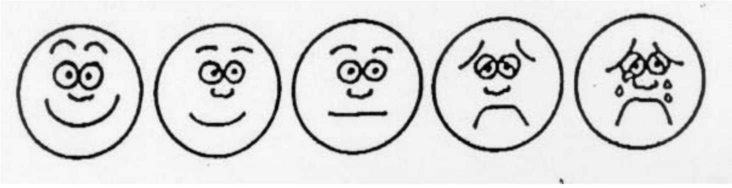

En Wong DL, Hockenberry-Eaton M, Wilson D,Winklestein ML, Schwartz P.: Wong's Essentials of Pediatric Nursing, ed. 6, St. Louis, 2001, Mosby, p 1301. Copyright Mosby. Reimpreso con permiso.

Los derechos de autor del SMILY- illness pertenecen a L. Nandini Moorthy, MD MS, RWJM-UMDNJ, New Brunswick, NJ. En este punto, el cuestionario sólo puede ser usado por L. Nandini Moorthy, MD MS y el personal de investigación que designe.

PREGUNTAS

En este cuestionario, le vamos a hacer muchas preguntas sobre la enfermedad y sobre lo que piensa su hijo/a. No hay malas respuestas. Por favor, pregúntenos lo que no entienda. Si quiere escribir algún comentario, hágalo al final, después de las páginas 5 y 6.

Hay varias cosas que afectan la vida de su hijo y que hacen que se sienta contento/a o triste. Hay cosas que son importantes para su hijo/a, que él o ella puede hacer (o que no puede hacer). Todas estas cosas forman parte de la calidad de vida del niño.

1. ¿Cómo es la calidad de vida de su hijo/a? Por favor, marque con un círculo la cara que mejor indique cómo es su calidad de vida.

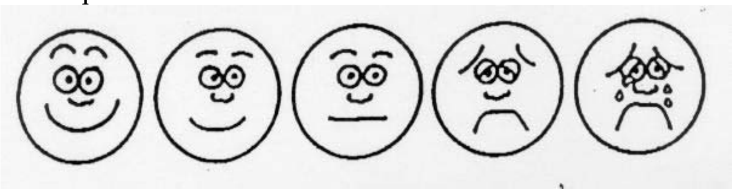

2. ¿Cómo va la enfermedad de su hijo/a en estos momentos? Por favor, marque con un círculo la cara que mejor indique cómo va la enfermedad de su hijo/a.

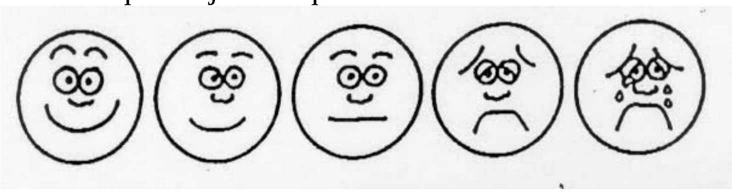

Copyright © 2007 Lakshmi Nandini Moorthy. Todos los derechos reservados.

Las preguntas 3-19 son sobre cómo se ha sentido su hijo/a en este último mes por a causa de la enfermedad. Por favor, marque con un círculo la cara que mejor indique cómo se siente.

3. ¿Cómo se siente su hijo/a por tener la enfermedad?

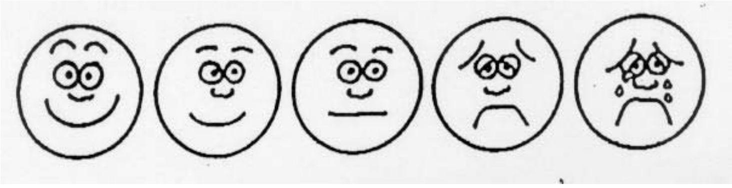

4. ¿Cómo hace la enfermedad que se sienta su hijo/a al ir a la escuela?

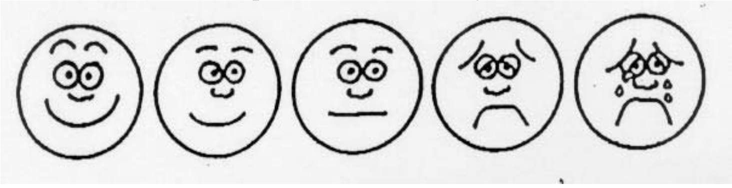

5. ¿Cómo hace la enfermedad que se sienta su hijo al vestirse cada día?

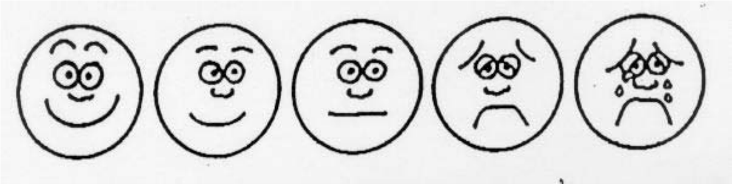

6. ¿Cómo se siente su hijo/a por el aspecto que tiene con la enfermedad?

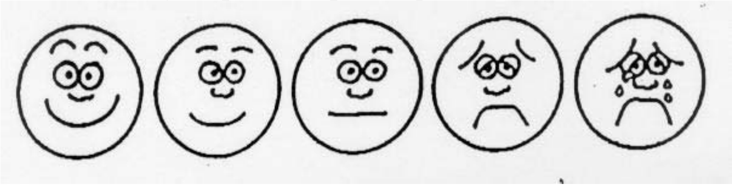

7. ¿Qué piensa su hijo/a de sí mismo/a por tener la enfermedad?

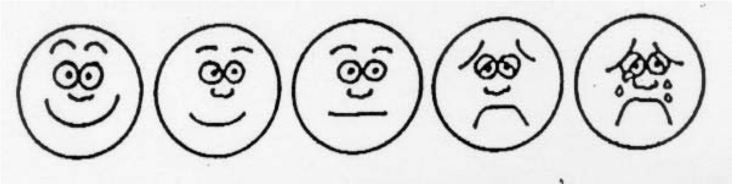

Copyright © 2007 Lakshmi Nandini Moorthy. Todos los derechos reservados.

8. ¿Cómo se siente su hijo/a cuando tiene que hablar de la enfermedad?

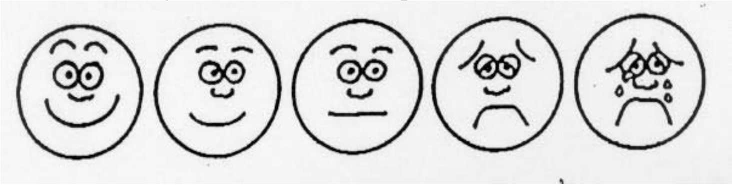

9. ¿Cómo se siente su hijo/a por tener que ir al médico por la enfermedad?

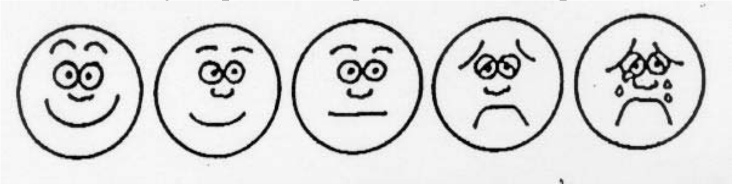

10. ¿Cómo se siente su hijo/a por tener que ir al hospital por la enfermedad?

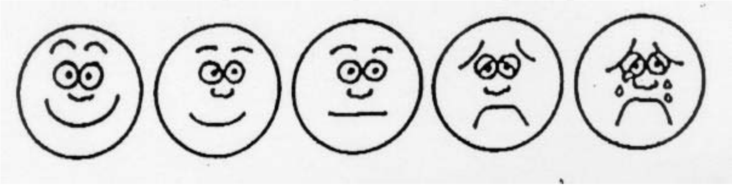

11. ¿Cómo hace la enfermedad que se sienta su hijo/a al pensar en el futuro?

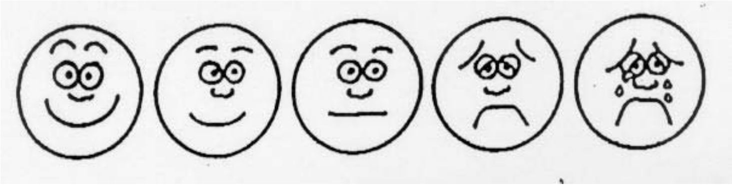

12. ¿Cómo se siente su hijo/a al tomar medicamentos por la enfermedad?

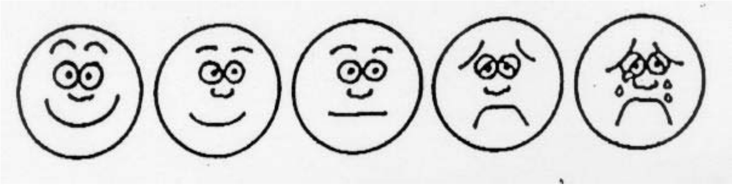

13. ¿Cuán preocupado/a está su hijo/a de los efectos secundarios que puedan tener las medicamentos que toma para la enfermedad?

Copyright © 2007 Lakshmi Nandini Moorthy. Todos los derechos reservados.

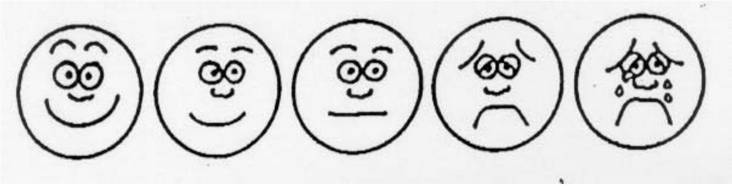

14. ¿Cuán preocupado/a está su hijo/a de que la enfermedad empeore?

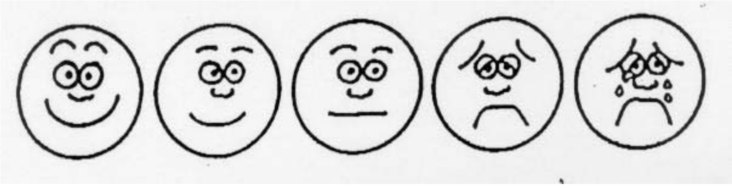

15. ¿Cuán solitario/a se siente su hijo/a por la enfermedad?

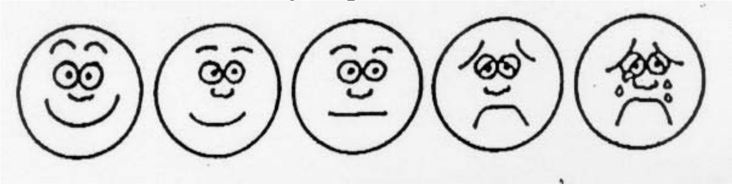

16. ¿Cómo hace sentir su familia a su hijo/a?

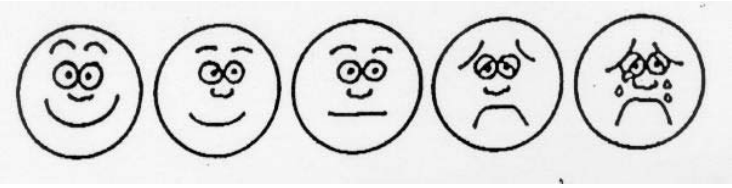

17. ¿Cómo se siente su hijo/a por el dolor de la enfermedad?

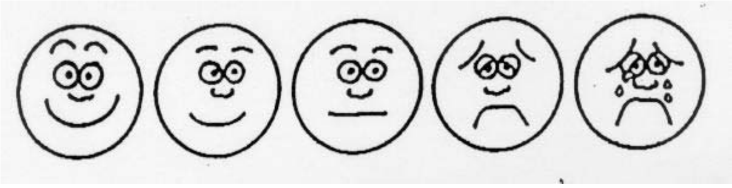

18. ¿Cómo se siente su hijo/a por el hecho de no poder estar al sol por la enfermedad?

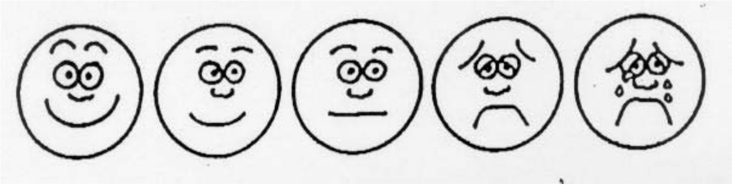

19. ¿Cuán cansado/a se siente su hijo/a por la enfermedad?

Copyright © 2007 Lakshmi Nandini Moorthy. Todos los derechos reservados.

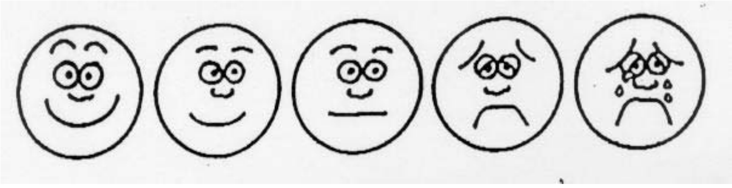

Conteste "sí" o "no" a las preguntas 20 A-26 A, y luego siga las instrucciones.

20 A. Los amigos de su hijo/a han cambiado desde que tiene la enfermedad. Sí
No

Si contestó que "sí", pase a la pregunta 20 B.

20 B. ¿Cómo hace que se sienta su hijo con sus amigos/as al tener la enfermedad? Por favor, marque con un círculo la cara que mejor indique cómo se siente su hijo/a.

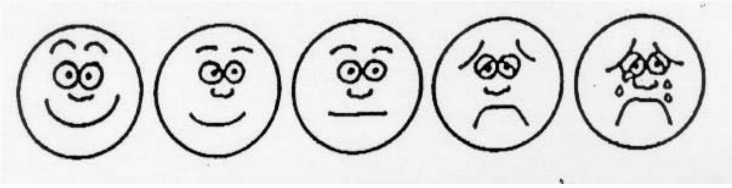

21 A. Los amigos/as de su hijo/a entienden que tiene la enfermedad. Sí
No

Si contestó que "no", pase a la pregunta 21 B.

21 B. ¿Cómo hace que se sienta su hijo/a que sus amigos/as no entiendan que tiene la enfermedad? Por favor, marque con un círculo la cara que mejor indique cómo se siente su hijo/a.

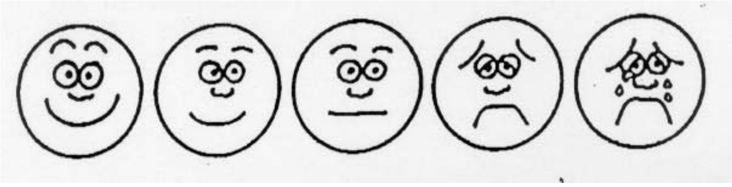

22 A. La enfermedad afecta el trabajo de su hijo/a en la escuela. Sí
No

Si contestó que "sí", pase a la pregunta 22 B

22 B. ¿Cómo hace la enfermedad que se sienta su hijo/a con el trabajo de la escuela? Por favor, marque con un círculo la cara que mejor indique cómo se siente su hijo/a.

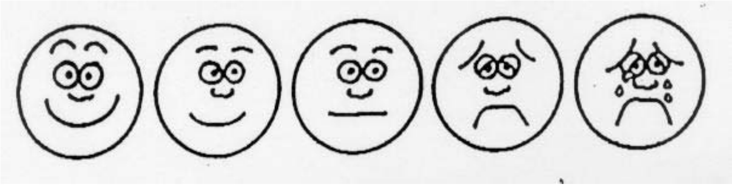

Copyright © 2007 Lakshmi Nandini Moorthy. Todos los derechos reservados.

23 A. Su hijo/a falta a la escuela por la enfermedad. Sí No

Si contestó que "sí", pase a la pregunta 23 B.

23 B. ¿Cómo se siente su hijo/a al faltar a la escuela por la enfermedad? Por favor, marque con un círculo la cara que mejor indique cómo se siente su hijo/a.

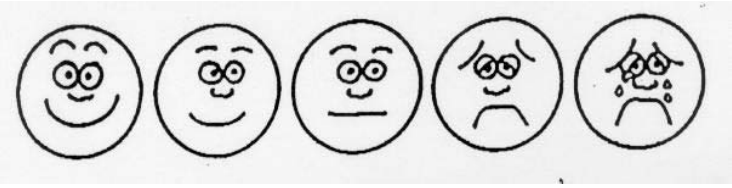

24 A. La enfermedad afecta las actividades de su hijo/a de fuera de la escuela. Sí No

Si contestó que "sí", pase a la pregunta 24 B.

24 B. ¿Cómo se siente su hijo/a de que la enfermedad afecte sus actividades de fuera de la escuela? Por favor, marque con un círculo la cara que mejor indique cómo se siente su hijo/a.

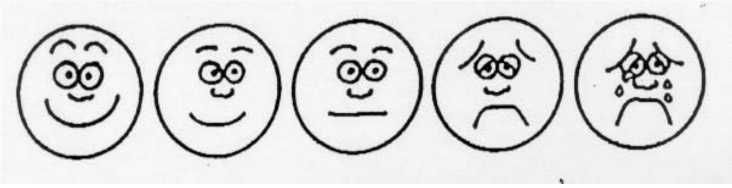

25 A. La enfermedad afecta la diversión que su hijo/a tiene con sus amigos. Sí No

Si contestó que "sí", pase a la pregunta 25 B.

25 B. ¿Cómo se siente su hijo/a de que la enfermedad afecte la diversión que tiene con sus amigos/as? Por favor, marque con un círculo la cara que mejor indique cómo se siente su hijo/a.

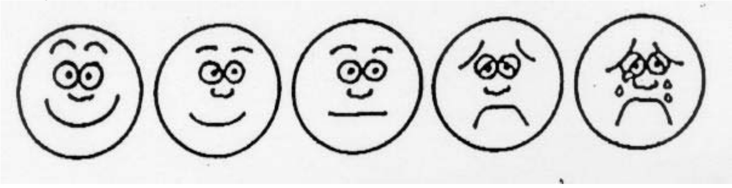

26 A. La enfermedad afecta la participación de su hijo/a en actividades físicas (como por ejemplo, hacer ejercicio en la escuela, correr, caminar, jugar a sus juegos favoritos al aire libre). Sí No

Si contestó que "sí", pase a la pregunta 26B.

26 B. ¿Cómo se siente su hijo/a de que la enfermedad afecte su participación en actividades físicas (como por ejemplo, hacer ejercicio en la escuela, correr, caminar, jugar a sus juegos favoritos de aire libre)? Por favor, marque con un círculo la cara que mejor indique cómo se siente su hijo/a.

Copyright © 2007 Lakshmi Nandini Moorthy. Todos los derechos reservados.

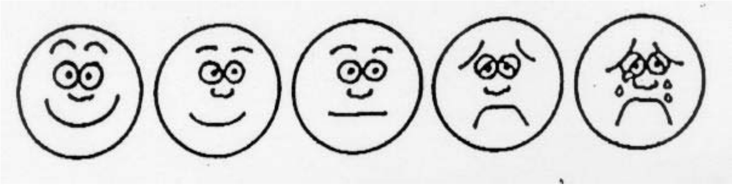

Copyright © 2007 Lakshmi Nandini Moorthy. Todos los derechos reservados.

MEDIDA SIMPLE DEL IMPACTO DEL LUPUS ERITEMATOSO EN LOS NIÑOS (SMILEY©) Versión para niños

Spanish-US&PR

Número de estudio __ Fecha __/__/200__

Para este cuestionario hemos seleccionado las 5 caras que siguen de la escala de puntuación de dolor Wong-Baker FACES, que hemos modificado con permiso de D.L. Wong. Las cinco caras aparecerán luego de cada pregunta.

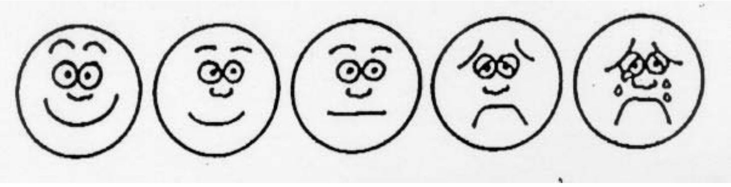

En Wong DL, Hockenberry-Eaton M, Wilson D,Winklestein ML, Schwartz P.: Wong's Essentials of Pediatric Nursing, ed. 6, St. Louis, 2001, Mosby, p 1301. Copyright Mosby. Reimpreso con permiso.

© Los derechos de autor del SMILEY pertenecen a L.Nandini Moorthy,MD,MS,RWJM-UMDNJ, NewBrunswick, NJ. Actualmente, el questionario solo puede ser usado por L.Nandini Morrthy, MD,MS y personal de investigación designado.

PREGUNTAS

En este cuestionario, te vamos a hacer muchas preguntas sobre tu lupus y sobre cómo te sientes. No hay respuestas incorrectas. Por favor, pregúntanos lo que no entiendas. Si quieres escribir algún comentario, hazlo al final, después de las páginas 5 y 6.

1. Hay varias cosas que afectan la vida personal y que hacen que uno se sienta contento o triste. Hay cosas que son importantes para ti, que tú puedes hacer (o que no puedes hacer). Todas estas cosas forman parte de la calidad de vida de una persona.

   ¿Cómo es tu calidad de vida? Por favor, marca con un círculo la cara que mejor indique cómo es tu calidad de vida.

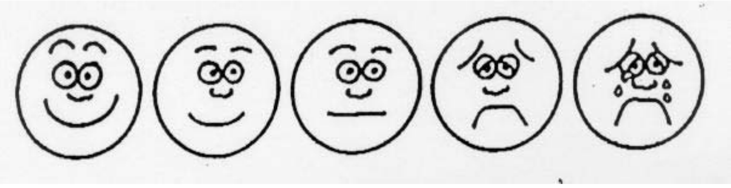

2. ¿Cómo va tu lupus en estos momentos? Por favor, marca con un círculo la cara que mejor indique cómo va tu lupus.

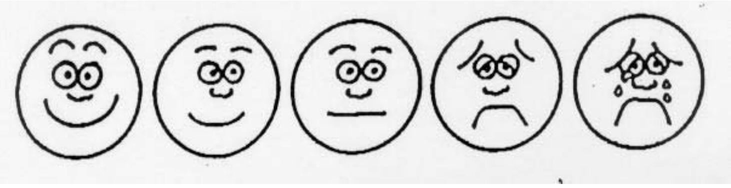

versión: 05/24/2007

Copyright © 2003 L. Nandini Moorthy, MD MS. All rights reserved.

Las preguntas 3-19 son acerca de cómo te has sentido en este último mes por causa del lupus. Por favor, marca con un círculo la cara que mejor indique cómo te sientes.

3. ¿Cómo te sientes por tener lupus?

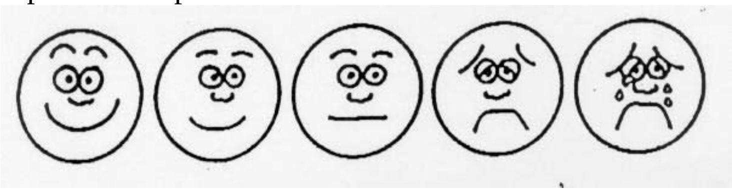

4. ¿Cómo hace el lupus que te sientas al ir a la escuela?

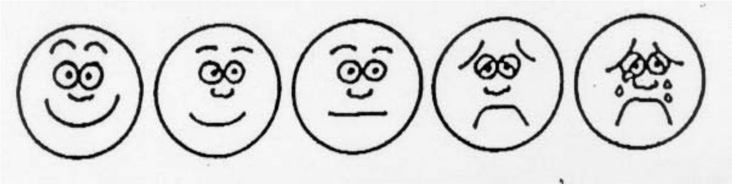

5. ¿Cómo hace el lupus que te sientas cuando te vistes cada día?

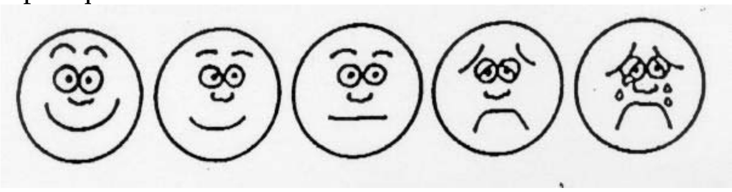

6. ¿Cómo te sientes acerca de como te ves por el lupus ?

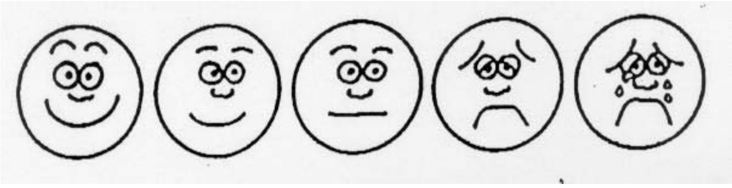

7. ¿Qué piensas de ti mismo por tener lupus?

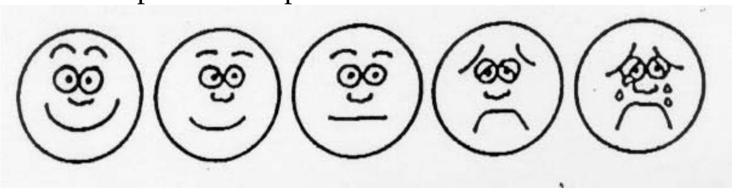

8. ¿Cómo te sientes cuando tienes que hablar del lupus?

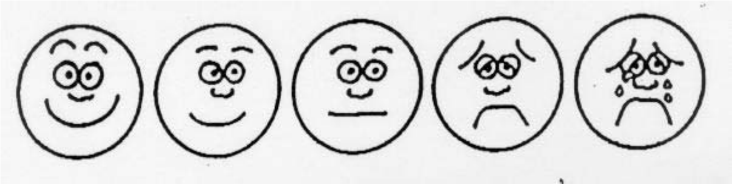

Copyright © 2003 L. Nandini Moorthy, MD MS. All rights reserved.

9. ¿Cómo te sientes por tener que ir al médico por el lupus?

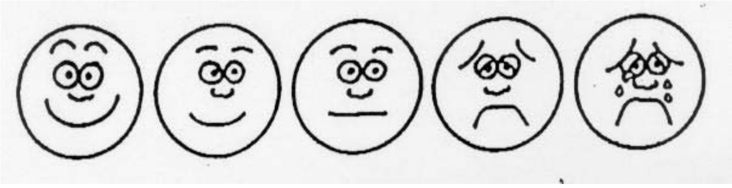

10. ¿Cómo te sientes por tener que ir al hospital por el lupus?

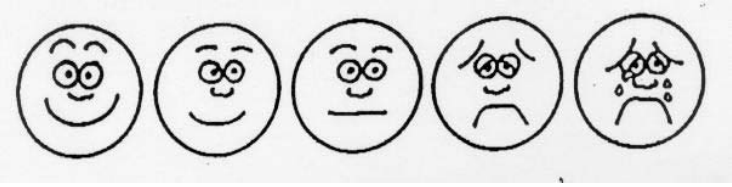

11. ¿Cómo hace el lupus que te sientas al pensar en tu futuro?

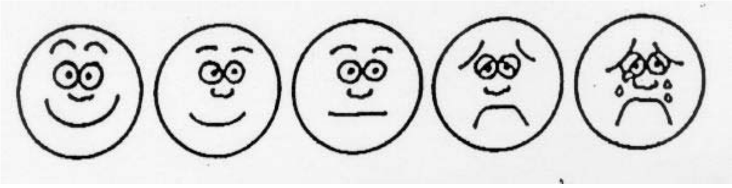

12. ¿Cómo te sientes al tomar medicinas por el lupus?

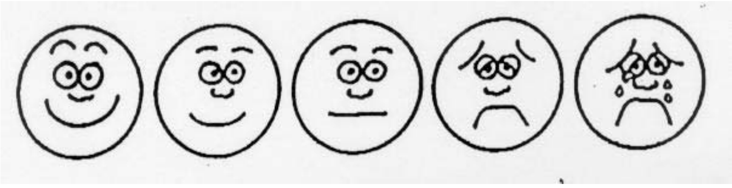

13. ¿Cuán preocupado/a estás por los efectos secundarios que puedan tener las medicinas que tomas para el lupus?

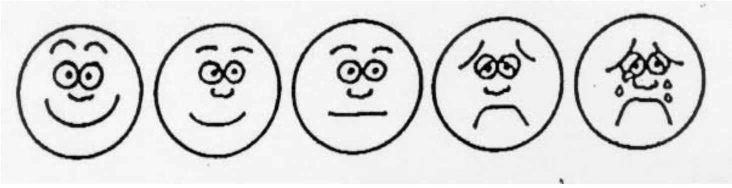

14. ¿Cuán preocupado/a estás de que tu lupus empeore?

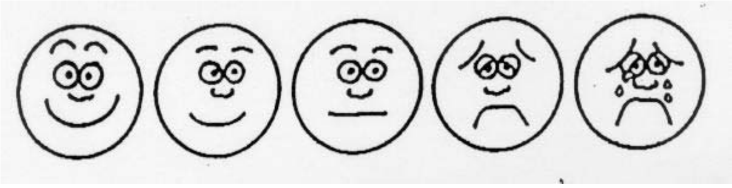

Copyright © 2003 L. Nandini Moorthy, MD MS. All rights reserved.

15. ¿Cuán solo/a te sientes por el lupus?

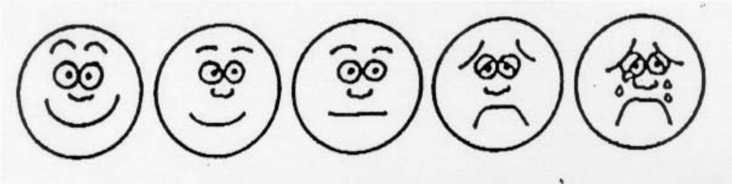

16. ¿Cómo te hace sentir tu familia?

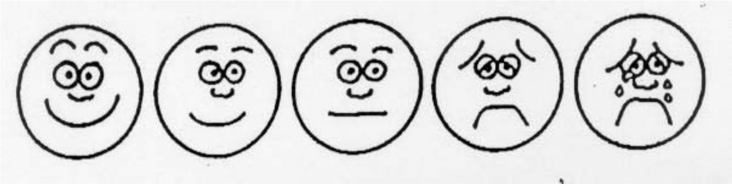

17. ¿Cómo te sientes por el dolor del lupus?

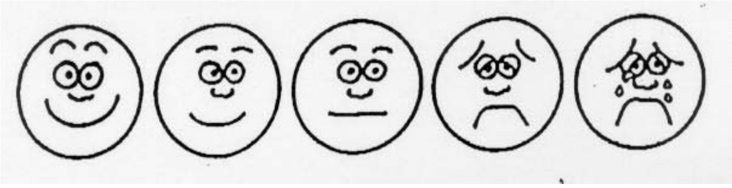

18. ¿ Cómo te sientes por no poder salir al sol por el lupus?

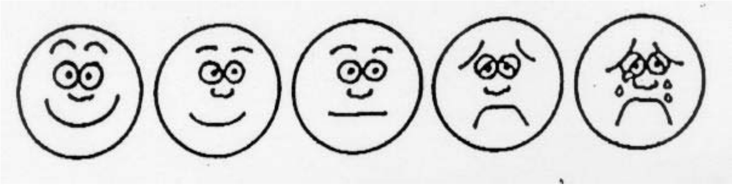

19. ¿Cuán cansado/a te sientes por el lupus?

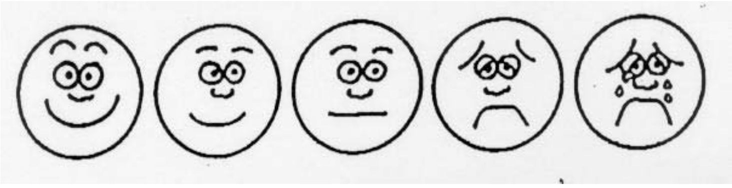

versión: 05/24/2007
Copyright © 2003 L. Nandini Moorthy, MD MS. All rights reserved.

Contesta "sí" o "no" a las preguntas 20 A-26 A, y luego sigue las instrucciones.

20 A. Mis amigos han cambiado porque tengo lupus. Sí No

Si contestaste que "sí", pasa a la pregunta 20 B.

20 B. ¿ Cómo te sientes acerca de tus amigos por tener lupus? Por favor, marca con un círculo la cara que mejor indique cómo te sientes.

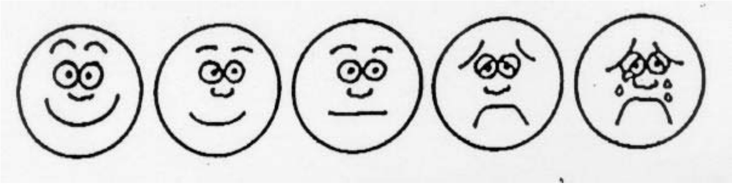

21 A. Mis amigos entienden que tengo lupus Sí No

Si contestaste que "no", pasa a la pregunta 21 B.

21 B. ¿ Cómo te sientes porque tus amigos no entienden que tienes lupus? Por favor, marca con un círculo la cara que mejor indique cómo te sientes.

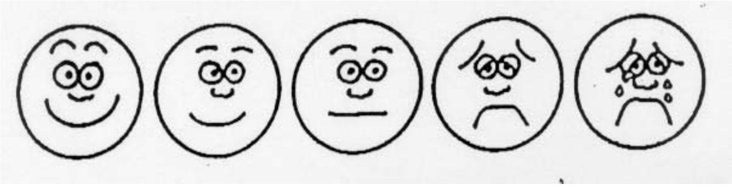

22 A. El lupus afecta mi trabajo en la escuela. Sí No

Si contestaste que "sí", pasa a la pregunta 22 B

22 B. ¿Cómo hace el lupus que te sientas con el trabajo de la escuela? Por favor, marca con un círculo la cara que mejor indique cómo te sientes.

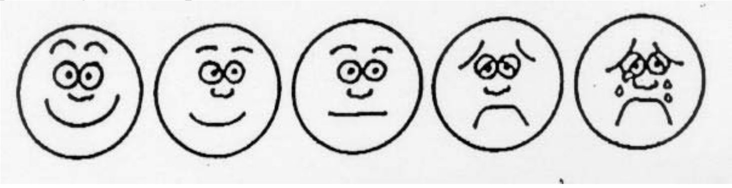

23 A. Falto a la escuela por el lupus. Sí No

Si contestaste que "sí", pasa a la pregunta 23 B.

23 B. ¿Cómo hace que te sientas faltar a la escuela por tener lupus? Por favor, marca con un círculo la cara que mejor indique cómo te sientes.

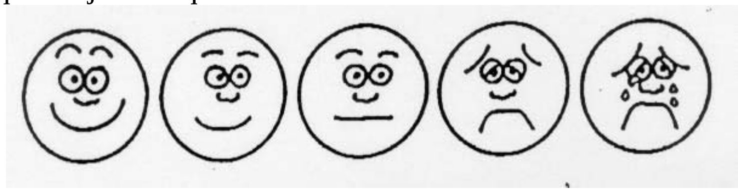

versión: 05/24/2007
Copyright © 2003 L. Nandini Moorthy, MD MS. All rights reserved.

24 A. El lupus afecta mis actividades fuera de la escuela. Sí No

Si contestaste que "sí", pasa a la pregunta 24 B.

24 B. ¿ Cómo hace que te sientes el hecho de que el lupus afecta tus actividades fuera de la escuela? Por favor, marca con un círculo la cara que mejor indique cómo te sientes.

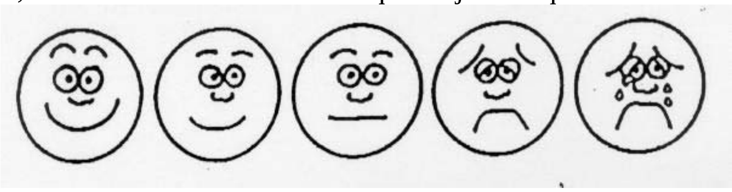

25 A. El lupus afecta como me divierto con mis amigos. Sí No

Si contestaste que "sí", pasa a la pregunta 25 B.

25 B. ¿Cómo hace que te sientas que el lupus afecte como te diviertes con tus amigos? Por favor, marca con un círculo la cara que mejor indique cómo te sientes.

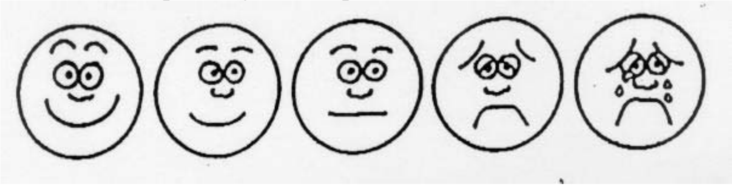

26 A. El lupus afecta mi participación en actividades físicas (como por ejemplo, hacer ejercicio en la escuela, correr, caminar, participar en mis juegos favoritos al aire libre).

Sí No

Si contestaste que "sí", pasa a la pregunta 26B.

26 B. ¿Cómo hace que te sientas el hecho de que el lupus afecte tu participación en actividades físicas (como por ejemplo, hacer ejercicio en la escuela, correr, caminar, participar en mis juegos favoritos al aire libre)? Por favor, marca con un círculo la cara que mejor indique cómo te sientes.

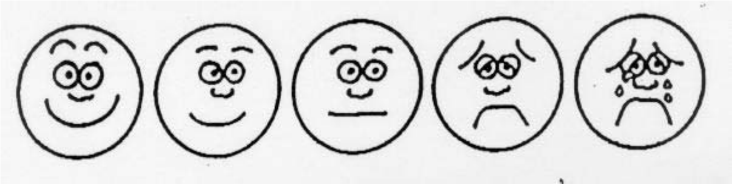

versión: 05/24/2007

Copyright © 2003 L. Nandini Moorthy, MD MS. All rights reserved.

MEDIDA SIMPLE DEL IMPACTO DEL LUPUS ERITEMATOSO EN LOS NIÑOS (SMILEY©) – Versión para los padres

Número de estudio __ Fecha __/__/200_

Para este cuestionario hemos seleccionado las 5 caras que siguen de la escala de puntuación de dolor Wong-Baker FACES, que hemos modificado con permiso de D.L. Wong. Las cinco caras aparecerán luego de cada pregunta.

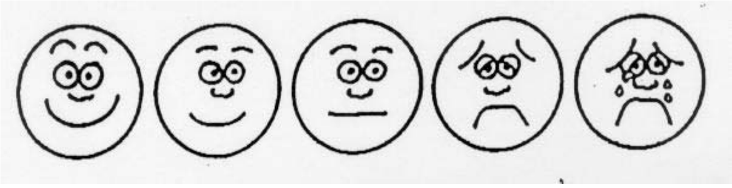

En Wong DL, Hockenberry-Eaton M, Wilson D,Winklestein ML, Schwartz P.: Wong's Essentials of Pediatric Nursing, ed. 6, St. Louis, 2001, Mosby, p 1301. Copyright Mosby. Reimpreso con permiso.

© ***Los derechos de autor del SMILEY pertenecen a L.Nandini Moorthy,MD,MS,RWJM-UMDNJ, NewBrunswick, NJ. Actualmente, el questionario solo puede ser usado por L.Nandini Morrthy, MD,MS y personal de investigación designado.***

PREGUNTAS

En este cuestionario, le vamos a hacer muchas preguntas sobre el lupus y sobre como se siente su hijo/a. No hay respuestas incorrectas. Por favor, pregúntenos lo que no entienda. Si quiere escribir algún comentario, hágalo al final, después de las páginas 5 y 6.

1. Hay varias cosas que afectan la vida de su hijo y que hacen que se sienta contento/a o triste. Hay cosas que son importantes para su hijo/a, que él o ella puede hacer (o que no puede hacer). Todas estas cosas forman parte de la calidad de vida del niño.

   ¿Cómo es la calidad de vida de su hijo/a? Por favor, marque con un círculo la cara que mejor indique cómo es su calidad de vida.

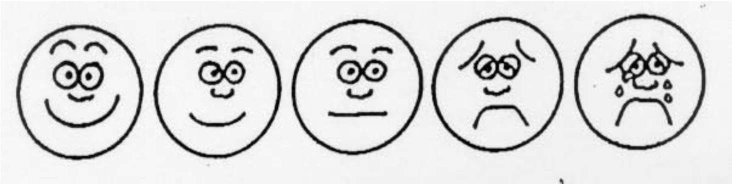

2. ¿Cómo va el lupus de su hijo/a en estos momentos? Por favor, marque con un círculo la cara que mejor indique cómo va el lupus de su hijo/a.

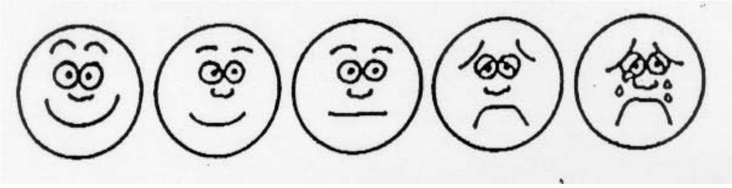

Copyright © 2003 L. Nandini Moorthy, MD MS. All rights reserved.

Las preguntas 3-19 son acerca de cómo se ha sentido en este último mes por causa del lupus de su hijo/a. Por favor, marque con un círculo la cara que mejor indique cómo se siente.

3. ¿Cómo se siente su hijo/a por tener lupus?

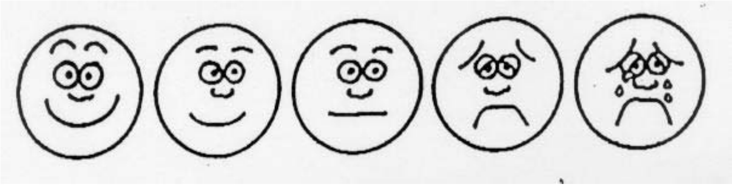

4. ¿Cómo hace el lupus que se sienta su hijo/a al ir a la escuela?

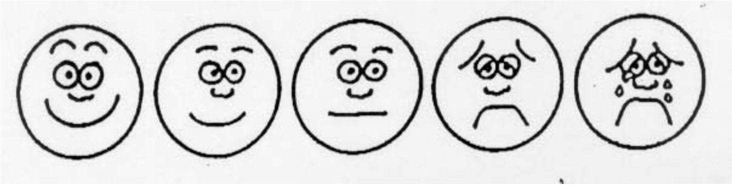

5. ¿Cómo hace el lupus que se sienta su hijo al tenerse que vestir cada día?

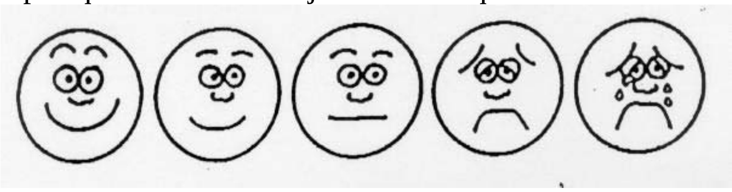

6. ¿Cómo se siente su hijo/a por el aspecto que tiene por el lupus?

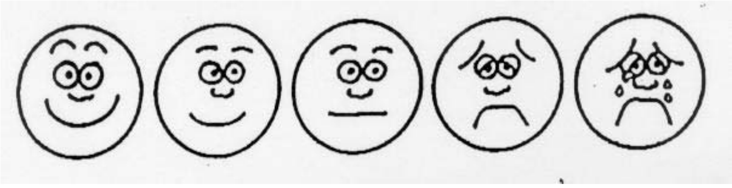

7. ¿Qué piensa su hijo/a de sí mismo/a por tener lupus?

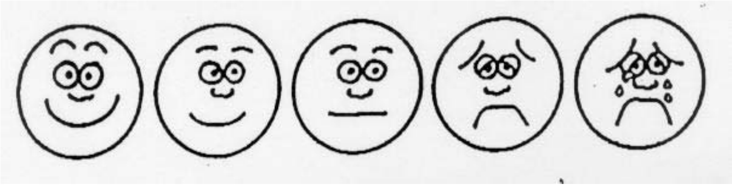

8. ¿Cómo se siente su hijo/a cuando tiene que hablar del lupus?

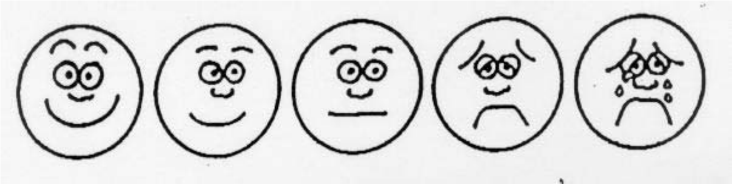

versión: 05/24/2007
Copyright © 2003 L. Nandini Moorthy, MD MS. All rights reserved.

9. ¿Cómo se siente su hijo/a por tener que ir al médico por el lupus?

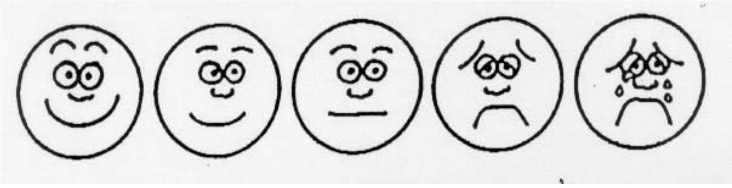

10. ¿Cómo se siente su hijo/a por tener que ir al hospital por el lupus?

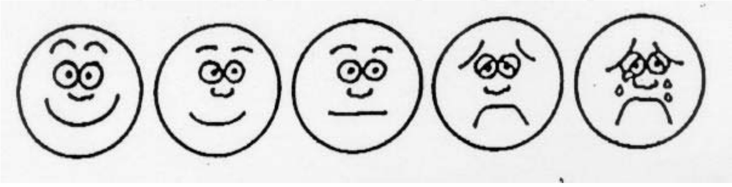

11. ¿Cómo hace el lupus que se sienta su hijo/a al pensar en el futuro?

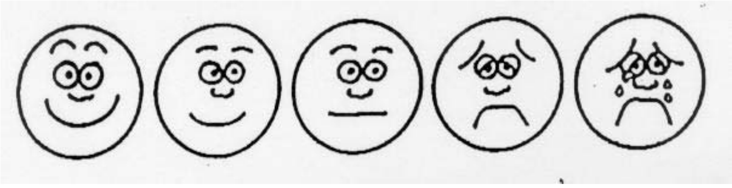

12. ¿Cómo se siente su hijo/a al tomar medicinas por el lupus?

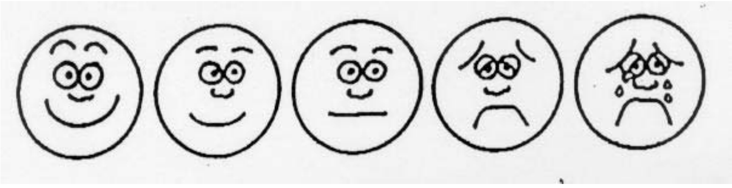

13. ¿Cuán preocupado/a está su hijo/a por los efectos secundarios que puedan tener las medicinas que toma para el lupus?

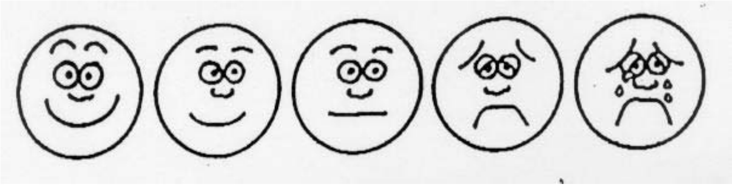

14. ¿Cuán preocupado/a está su hijo/a de que el lupus empeore?

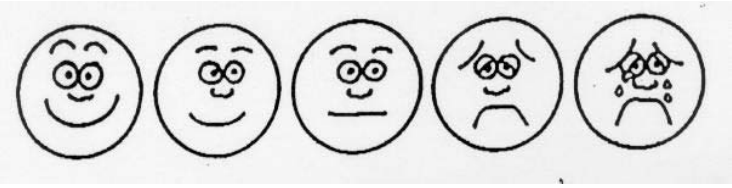

Copyright © 2003 L. Nandini Moorthy, MD MS. All rights reserved.

15. ¿Cuán solo/a se siente su hijo/a por el lupus?

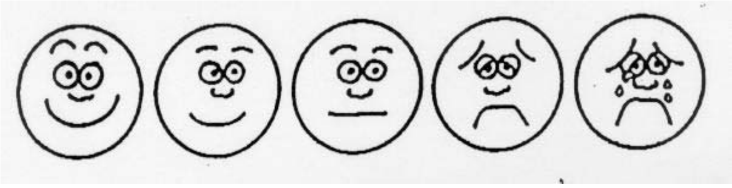

16. ¿Cómo hace sentir su familia a su hijo/a?

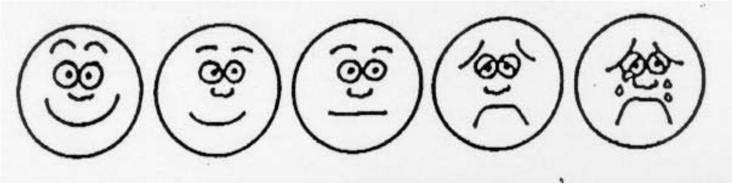

17. ¿Cómo se siente su hijo/a por el dolor del lupus?

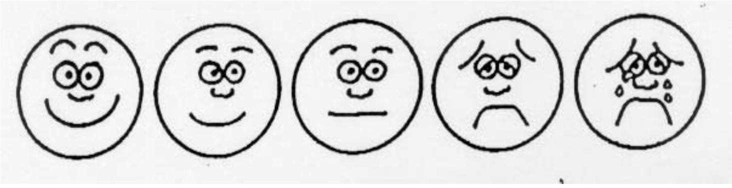

18. ¿Cómo se siente su hijo/a por el hecho de no poder salir al sol por el lupus?

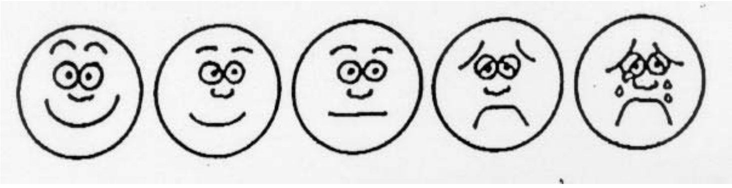

19. ¿Cuán cansado/a se siente su hijo/a por el lupus?

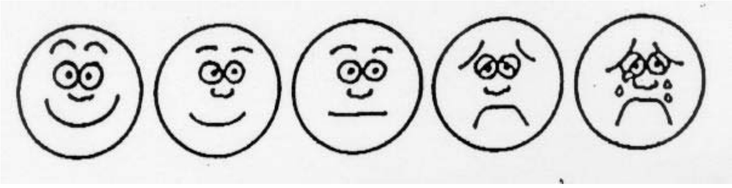

Copyright © 2003 L. Nandini Moorthy, MD MS. All rights reserved.

Conteste "sí" o "no" a las preguntas 20 A-26 A, y luego siga las instrucciones.

20 A. Los amigos de su hijo/a han cambiado desde que tiene lupus. Sí No

Si contestó que "sí", pase a la pregunta 20 B.

20 B. ¿Cómo hace que se sienta su hijo con sus amigos/as al tener lupus? Por favor, marque con un círculo la cara que mejor indique cómo se siente su hijo/a.

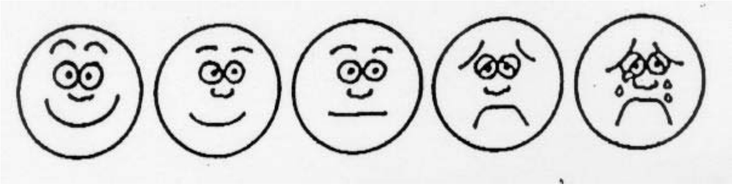

21 A. Los amigos/as de su hijo/a entienden que tiene lupus. Sí No

Si contestó que "no", pase a la pregunta 21 B.

21 B. ¿Cómo hace que se sienta su hijo/a que sus amigos/as no entiendan que tiene lupus? Por favor, marque con un círculo la cara que mejor indique cómo se siente su hijo/a.

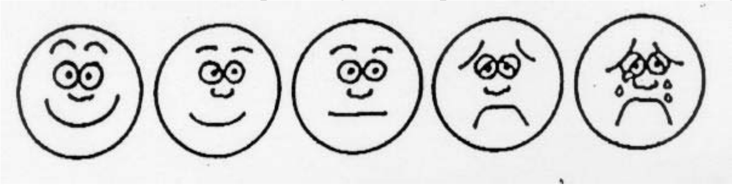

22 A. El lupus afecta el trabajo de su hijo/a en la escuela. Sí No

Si contestó que "sí", pase a la pregunta 22 B

22 B. ¿Cómo hace el lupus que se sienta su hijo/a con el trabajo de la escuela? Por favor, marque con un círculo la cara que mejor indique cómo se siente su hijo/a.

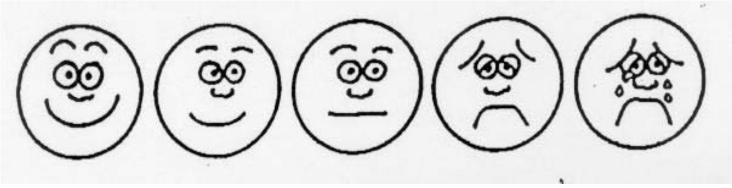

23 A. Su hijo/a falta a la escuela por el lupus. Sí No

Si contestó que "sí", pase a la pregunta 23 B.

23 B. ¿Cómo se siente su hijo/a al faltar a la escuela por el lupus? Por favor, marque con un círculo la cara que mejor indique cómo se siente su hijo/a.

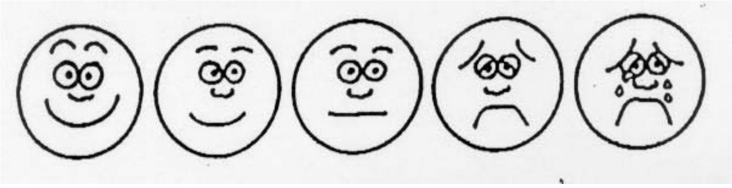

Copyright © 2003 L. Nandini Moorthy, MD MS. All rights reserved.

24 A. El lupus afecta las actividades de su hijo/a fuera de la escuela. Sí No

Si contestó que "sí", pase a la pregunta 24 B.

24 B. ¿ Cómo se siente su hijo/a el hecho de que el lupus afecte sus actividades fuera de la escuela? Por favor, marque con un círculo la cara que mejor indique cómo se siente su hijo/a.

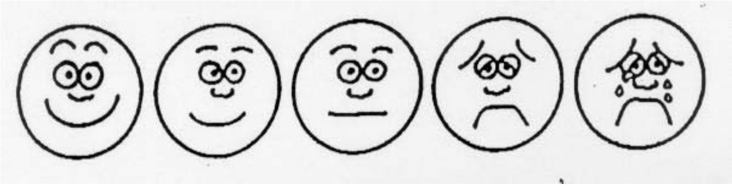

25 A. El lupus afecta como se divierte su hijo/a con sus amigos.

Sí No

Si contestó que "sí", pase a la pregunta 25 B.

25 B. ¿Cómo se siente su hijo/a de que el lupus afecte como se divierte con sus amigos/as? Por favor, marque con un círculo la cara que mejor indique cómo se siente su hijo/a.

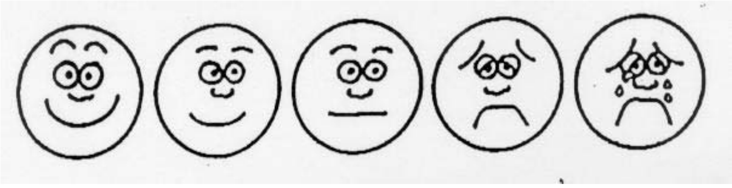

26 A. El lupus afecta la participación de su hijo/a en actividades físicas (como por ejemplo, hacer ejercicio en la escuela, correr, caminar, participar en sus juegos favoritos al aire libre).

Sí No

Si contestó que "sí", pase a la pregunta 26B.

26 B. ¿Cómo se siente su hijo/a de que el lupus afecte su participación en participar en mis juegos favoritos al aire libre)? Por favor, marque con un círculo la cara que mejor indique cómo se siente su hijo/a.

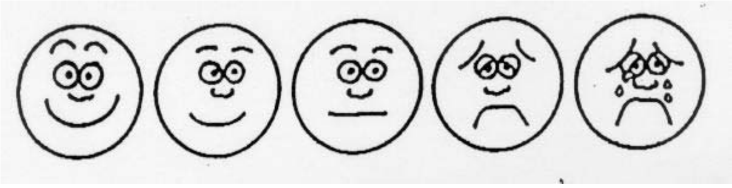

Copyright © 2003 L. Nandini Moorthy, MD MS. All rights reserved.

MEDIDA SIMPLE DEL IMPACTO DE LA ENFERMEDAD EN LOS NIÑOS
(SMILY-Illness) Versión para niños

Número de estudio __ Fecha __/__/20__(Spanish for Venezuela)

Para este cuestionario, hemos seleccionado las 5 caras que siguen de la escala de puntuación de dolor Wong-Baker FACES, que hemos modificado con permiso de D.L. Wong. Las cinco caras aparecerán luego de cada pregunta de este cuestionario.

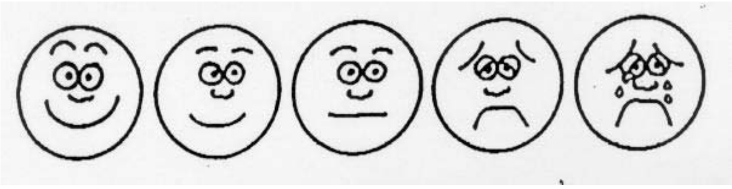

En Wong DL, Hockenberry-Eaton M, Wilson D, Winklestein ML, Schwartz P.: Wong's Essentials of Pediatric Nursing, ed. 6, St. Louis, 2001, Mosby, pág. 1301. Copyright Mosby. Reimpreso con permiso.

Los derechos de autor del SMILY-illness pertenecen a L. Nandini Moorthy, MD MS, RWJM-UMDNJ, New Brunswick, NJ. Actualmente, el cuestionario solo puede ser usado por L. Nandini Moorthy, MD MS y el personal de investigación designado.

PREGUNTAS

En este cuestionario, te vamos a hacer muchas preguntas acerca de tu enfermedad y de cómo te sientes. No hay respuestas incorrectas. Por favor, pregúntanos lo que no entiendas. Si quieres escribir algún comentario, hazlo al final, después de las páginas 5 y 6.

Hay varias cosas que afectan la vida personal y que hacen que uno se sienta contento o triste. Hay cosas que son importantes para ti, que tú puedes hacer (o que no puedes hacer). Todas estas cosas forman parte de la calidad de vida de una persona. Explicar lo que es calidad de vida

1. ¿Cómo es tu calidad de vida? Por favor, marca con un círculo la cara que mejor indique cómo es tu calidad de vida. El niño no va a entender calidad de vida sino se lo explican bien

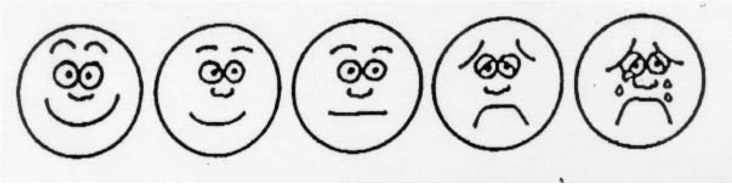

2. ¿Cómo te va con tu enfermedad en estos momentos? Por favor, marca con un círculo la cara que mejor indique cómo está tu enfermedad.

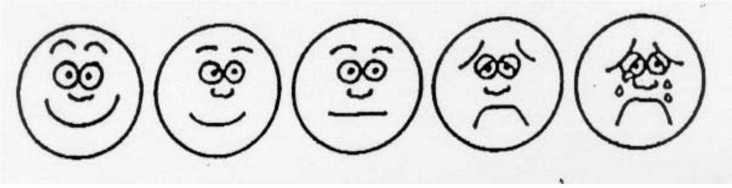

Copyright © 2007 Lakshmi Nandini Moorthy. Todos los derechos reservados.

Las preguntas 3-19 son acerca de cómo te has sentido en este último mes por causa de tu enfermedad. Por favor, marca con un círculo la cara que mejor indique cómo te sientes.

3. ¿Cómo te sientes por tener una enfermedad?

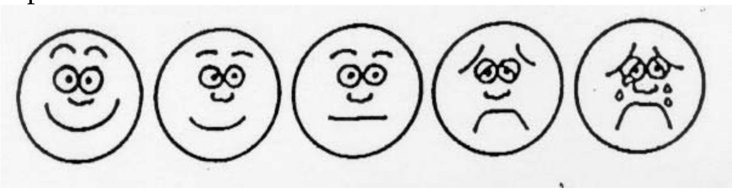

4. ¿Como te hace sentir esta enfermedad para ir a la escuela?

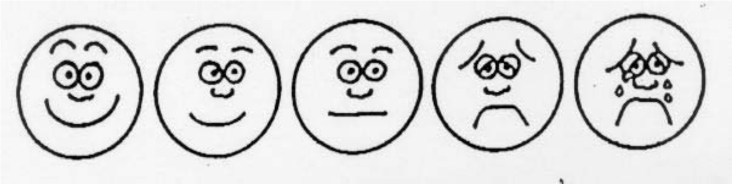

5. ¿Cómo te hace sentir esta enfermedad con respecto a vestirte cada día?

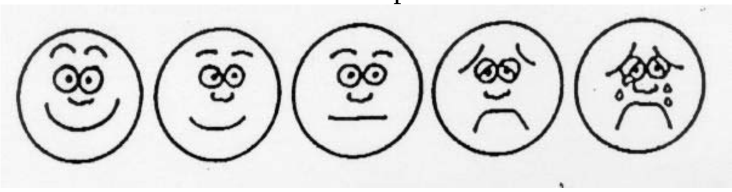

6. ¿Como te sientes con ESTA enfermedad respecto a como te ves? A tu aspecto físico?

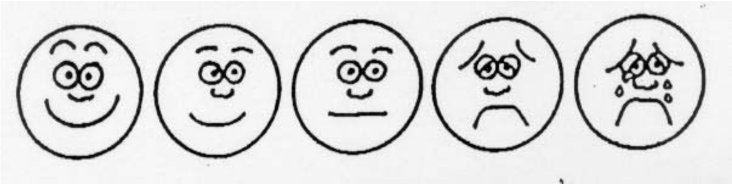

7. ¿Qué piensas de ti mismo por tener esta enfermedad?

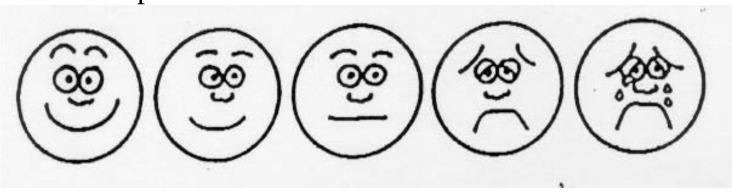

8. ¿Cómo te sientes cuando tienes que hablar acerca de tu enfermedad?

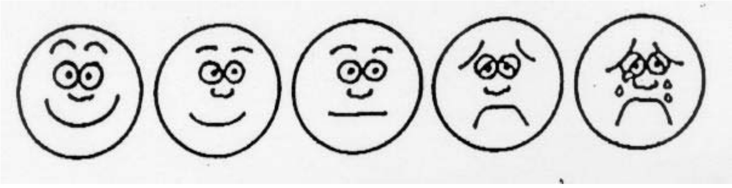

9. ¿Cómo te sientes por tener que ir al médico por esta enfermedad?

Copyright © 2007 Lakshmi Nandini Moorthy. Todos los derechos reservados.

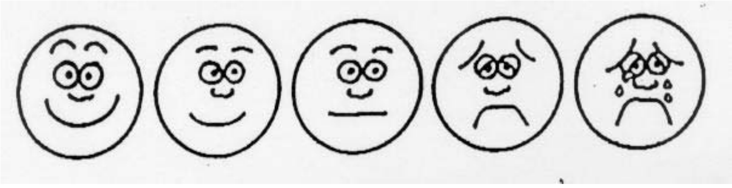

10. ¿Cómo te sientes por tener que ir al hospital por esta enfermedad?

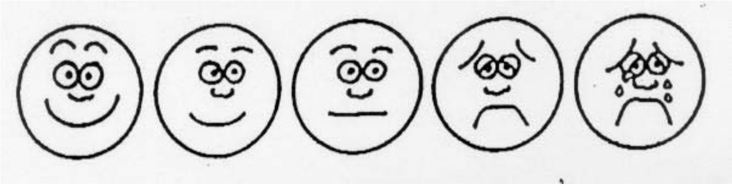

11. ¿Como te sientes acerca de tu futuro por esta enfermedad?

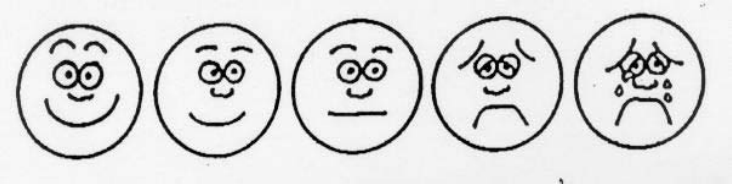

12. ¿Cómo te sientes al tomar medicinas por tu enfermedad?

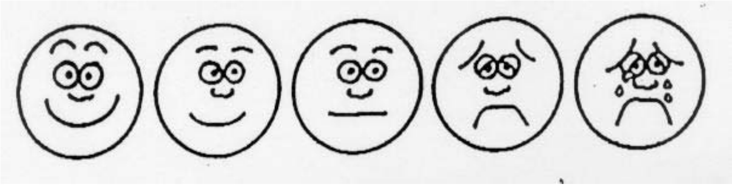

13. ¿Cuán preocupado/a estás por los efectos que puedan tener las medicinas que tomas para tu enfermedad?

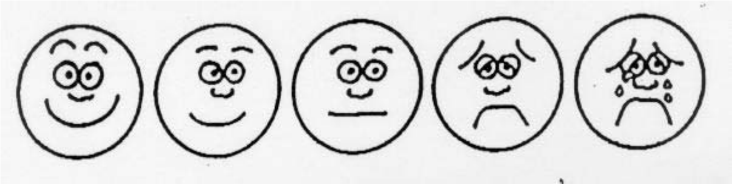

14. ¿Cuán preocupado/a estás de que tu enfermedad empeore?

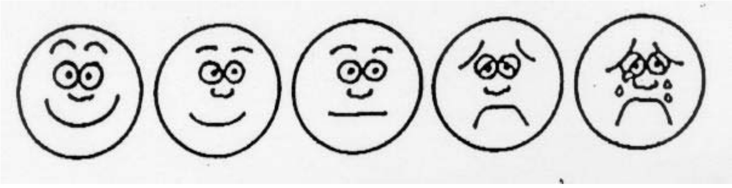

Copyright © 2007 Lakshmi Nandini Moorthy. Todos los derechos reservados.

15. ¿Cuán solo/a te sientes por tu enfermedad?

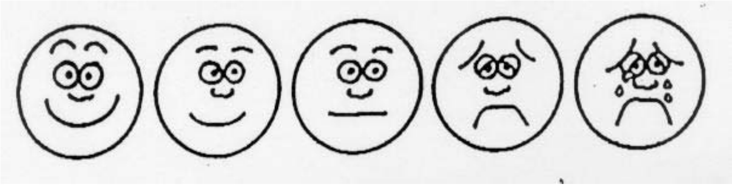

16. ¿Cómo te hace sentir tu familia?

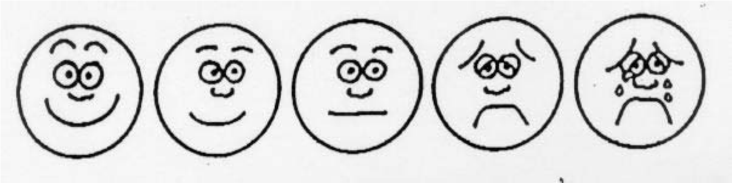

17. ¿Cómo te sientes por el dolor que te produce tu enfermedad?

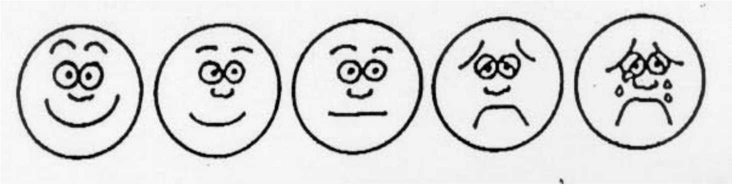

18. ¿Cómo te sientes por no poder salir al sol por tu enfermedad?

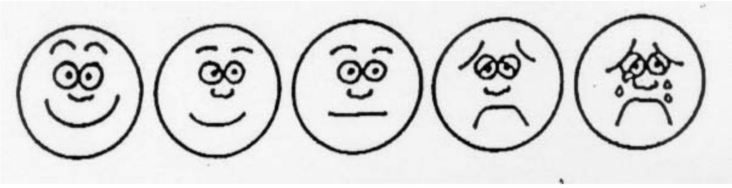

19. ¿Cuán cansado/a te sientes por tu enfermedad?

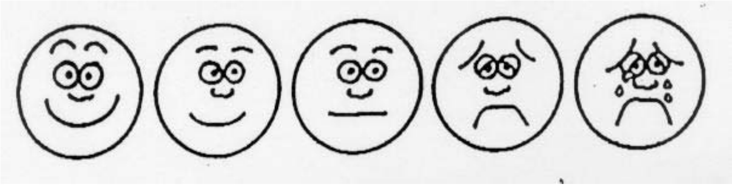

Contesta "sí" o "no" a las preguntas 20 A-26 A, y luego sigue las instrucciones.

Copyright © 2007 Lakshmi Nandini Moorthy. Todos los derechos reservados.

20 A. Mis amigos han cambiado porque tengo una enfermedad. Sí No

Si contestaste que "sí", pasa a la pregunta 20 B.

20 B. ¿Cómo te sientes con respecto a tus amigos por tener esta enfermedad? Por favor, marca con un círculo la cara que mejor indique cómo te sientes.

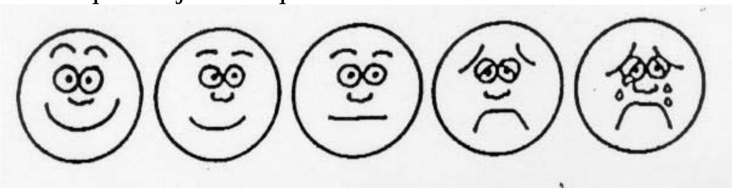

21 A. Mis amigos entienden que tengo una enfermedad Sí No

Si contestaste que "no", pasa a la pregunta 21 B.

21 B. ¿Cómo te sientes porque tus amigos no entienden que tienes una enfermedad? Por favor, marca con un círculo la cara que mejor indique cómo te sientes.

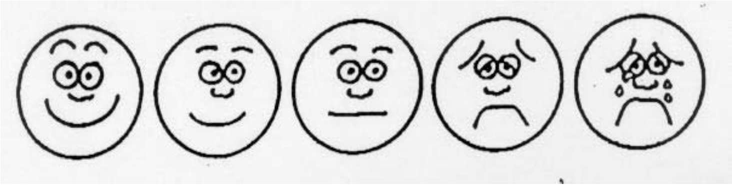

22 A. Mi enfermedad afecta mi trabajo en la escuela. Sí No

Si contestaste que "sí", pasa a la pregunta 22 B

22 B. ¿Cómo te hace sentir esta enfermedad con respecto al trabajo de la escuela? Por favor, marca con un círculo la cara que mejor indique cómo te sientes.

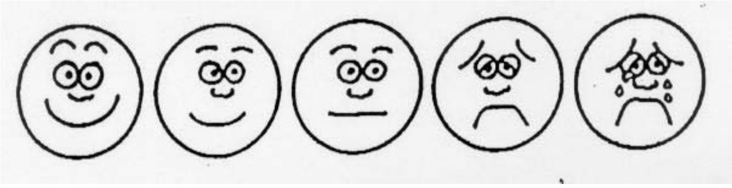

23 A. Falto a la escuela por mi enfermedad. Sí No

Si contestaste que "sí", pasa a la pregunta 23 B.

23 B. ¿Cómo te hace sentir el faltar a la escuela por tu enfermedad? Por favor, marca con un círculo la cara que mejor indique cómo te sientes.

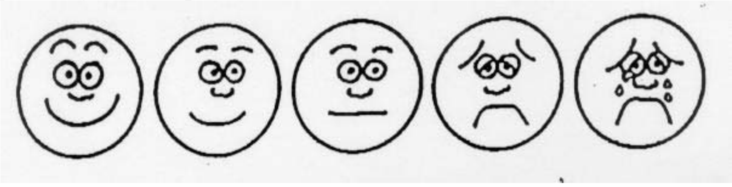

24 A. Mi enfermedad afecta mis actividades fuera de la escuela. Sí No

Si contestaste que "sí", pasa a la pregunta 24 B.

Copyright © 2007 Lakshmi Nandini Moorthy. Todos los derechos reservados.

24 B. ¿Cómo te hace sentir el hecho de que tu enfermedad afecte tus actividades fuera de la escuela? Por favor, marca con un círculo la cara que mejor indique cómo te sientes.

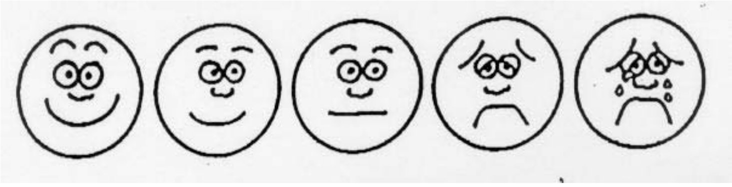

25 A. Mi enfermedad afecta la manera en que me divierto con mis amigos. Sí No

Si contestaste que "sí", pasa a la pregunta 25 B.

25 B. ¿Cómo te hace sentir que tu enfermedad afecte la manera en que te diviertes con tus amigos? Por favor, marca con un círculo la cara que mejor indique cómo te sientes.

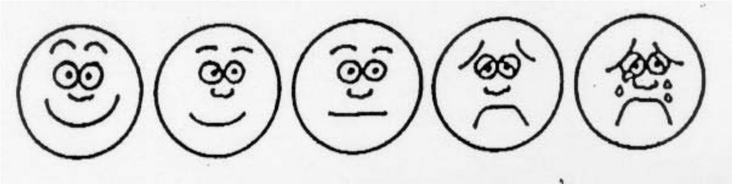

26 A. Mi enfermedad afecta mi participación en actividades físicas (como por ejemplo: hacer ejercicio en la escuela, correr, caminar, participar en mis juegos favoritos al aire libre).

Sí No

Si contestaste que "sí", pasa a la pregunta 26B.

26 B. ¿Cómo te hace sentir el hecho de que tu enfermedad afecte tu participación en actividades físicas (como por ejemplo: hacer ejercicio en la escuela, correr, caminar, participar en mis juegos favoritos al aire libre)? Por favor, marca con un círculo la cara que mejor indique cómo te sientes.

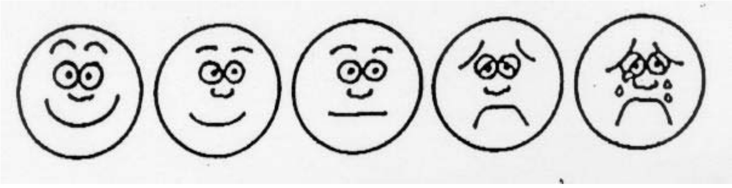

Copyright © 2007 Lakshmi Nandini Moorthy. Todos los derechos reservados.

MEDIDA SIMPLE DEL IMPACTO DE LA ENFERMEDAD EN LOS NIÑOS
(SMILY-Illness) – Versión para los padres

Número de estudio __ Fecha __/__/20_ (Spanish for Venezuela)

Para este cuestionario, hemos seleccionado las 5 caras que siguen de la escala de puntuación de dolor Wong-Baker FACES, que hemos modificado con permiso de D.L. Wong. Las cinco caras aparecerán luego de cada pregunta.

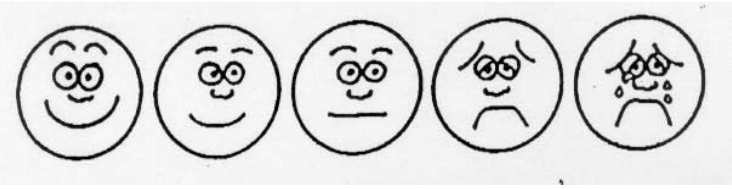

En Wong DL, Hockenberry-Eaton M, Wilson D, Winklestein ML, Schwartz P.: Wong's Essentials of Pediatric Nursing, ed. 6, St. Louis, 2001, Mosby, pág. 1301. Copyright Mosby. Reimpreso con permiso.

Los derechos de autor del SMILY-illness pertenecen a L. Nandini Moorthy, MD MS, RWJM-UMDNJ, New Brunswick, NJ. Actualmente, el cuestionario solo puede ser usado por L. Nandini Moorthy, MD MS y el personal de investigación designado.

PREGUNTAS

En este cuestionario, le vamos a hacer muchas preguntas sobre la enfermedad y sobre cómo se siente su hijo/a. No hay respuestas incorrectas. Por favor, pregúntenos lo que no entienda. Si quiere escribir algún comentario, hágalo al final, después de las páginas 5 y 6.

Hay varias cosas que afectan la vida de su hijo/a y que hacen que se sienta contento/a o triste. Hay cosas que son importantes para su hijo/a, que él o ella puede hacer (o que no puede hacer). Todas estas cosas forman parte de la calidad de vida del niño. EXPLICAR LO QUE ES CLAIDAD DE VIDA

1. ¿Cómo es la calidad de vida de su hijo/a? Por favor, marque con un círculo la cara que mejor indique cómo es su calidad de vida.

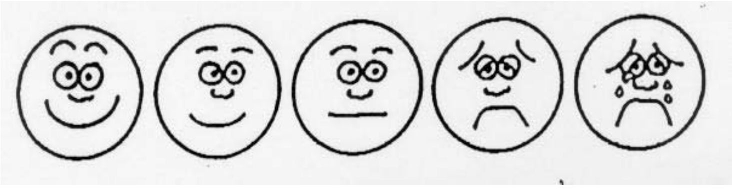

2. ¿Cómo se siente su hijo respecto a su enfermedad en estos momentos? Por favor, marque con un círculo la cara que mejor indique cómo está la enfermedad de su hijo/a.

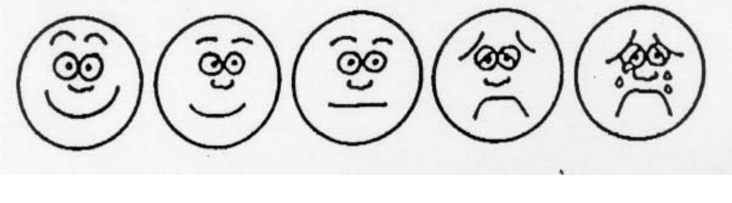

Copyright © 2007 Lakshmi Nandini Moorthy. Todos los derechos reservados.

Las preguntas 3-19 son acerca de cómo se ha sentido en este último mes por causa de la enfermedad de su hijo/a. Por favor, marque con un círculo la cara que mejor indique cómo se siente.

3. ¿Cómo se siente su hijo/a por tener esta enfermedad?

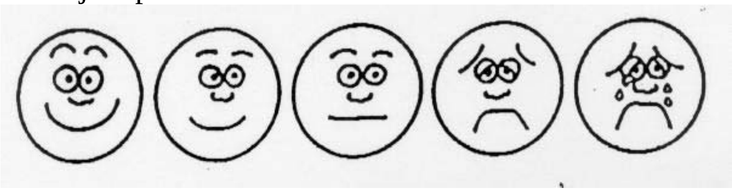

4. ¿ Como se siente su hijo con esta enfermedad con respecto a ir a la escuela?

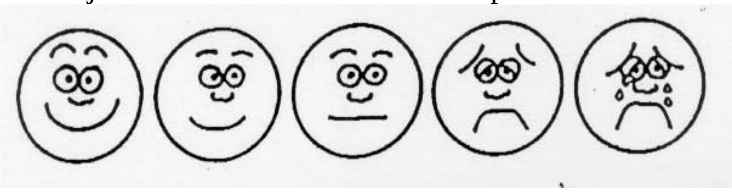

5. ¿Cómo se siente su hijo con esta enfermedad respecto a tener que vestirse cada dia?

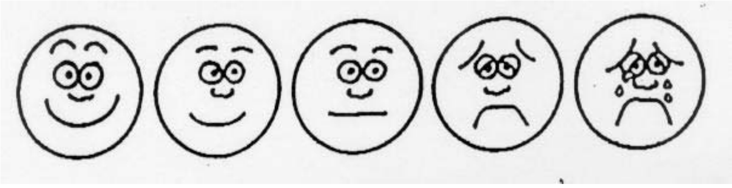

6. ¿Cómo se siente su hijo/a por el aspecto que tiene a causa de esta enfermedad?

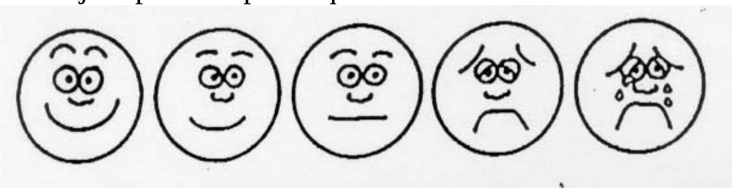

7. ¿ Como se siente su hijo con esta enfermedad respecto a si mismo?

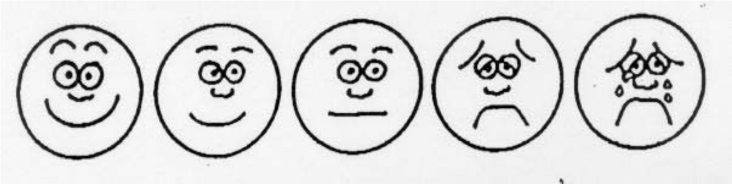

8. ¿Cómo se siente su hijo/a cuando tiene que hablar acerca de su enfermedad?

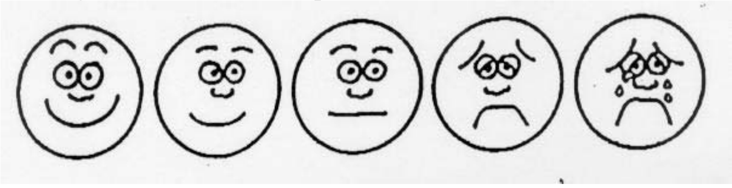

Copyright © 2007 Lakshmi Nandini Moorthy. Todos los derechos reservados.

9. ¿Cómo se siente su hijo/a por tener que ir al médico por su enfermedad?

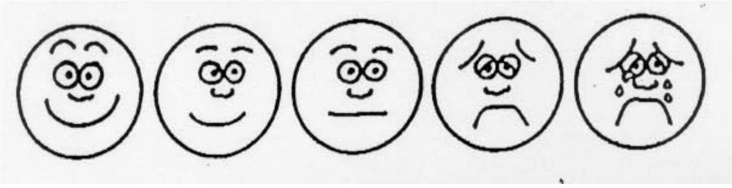

10. ¿Cómo se siente su hijo/a por tener que ir al hospital por su enfermedad?

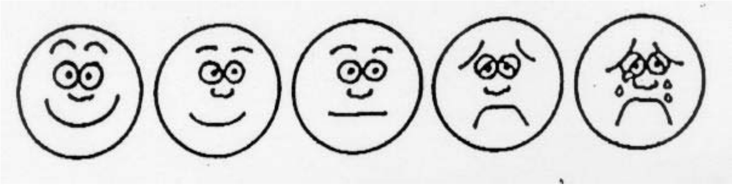

11. ¿Cómo hace esta enfermedad sentir a su hijo/a al pensar en el futuro?

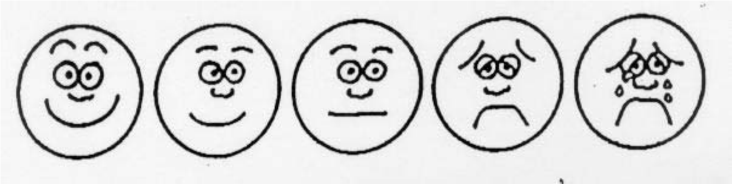

12. ¿Cómo se siente su hijo/a al tomar medicinas por su enfermedad?

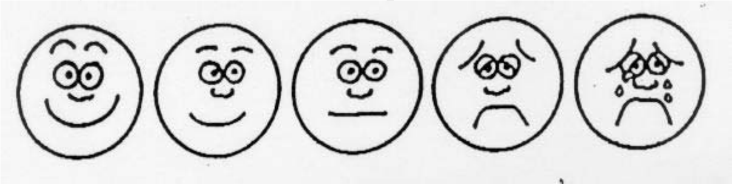

13. ¿Cuán preocupado/a está su hijo/a por los efectos que puedan tener las medicinas que toma para su enfermedad?

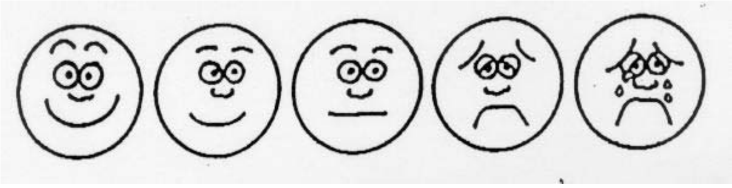

14. ¿Su hijo siente preocupación o esta preocupado porque su enfermedad empeore?

Copyright © 2007 Lakshmi Nandini Moorthy. Todos los derechos reservados.

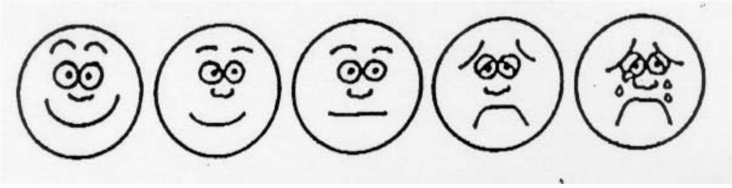

15. ¿Su hijo se siente solo por su enfermedad?

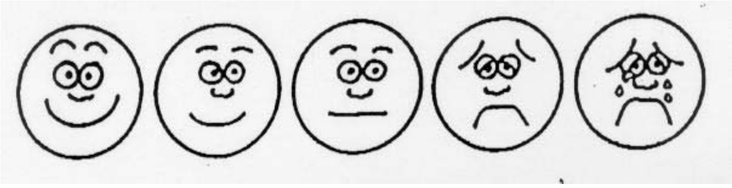

16. ¿Cómo hace sentir su familia a su hijo/a?

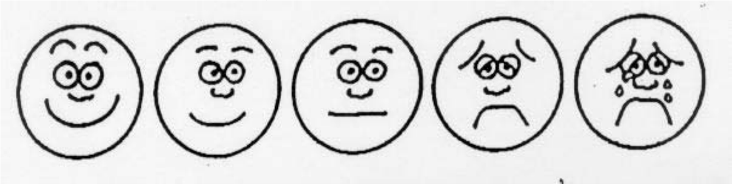

17. ¿Cómo se siente su hijo/a por el dolor que le produce su enfermedad?

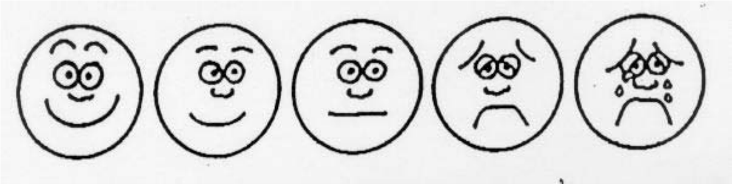

18. ¿Cómo se siente su hijo/a por el hecho de no poder salir al sol por su enfermedad?

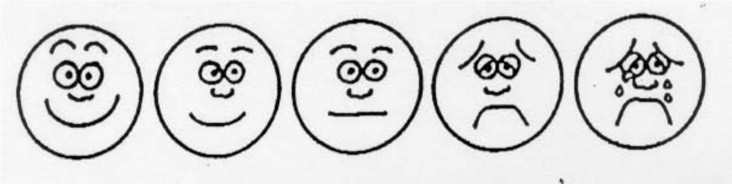

19. ¿Cuán cansado/a se siente su hijo/a por su enfermedad?

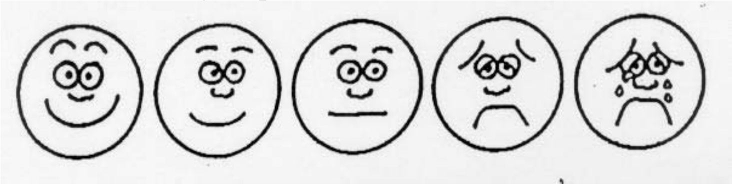

Copyright © 2007 Lakshmi Nandini Moorthy. Todos los derechos reservados.

Conteste "sí" o "no" a las preguntas 20 A-26 A, y luego siga las instrucciones.

20 A. Los amigos de su hijo/a han cambiado desde que tiene una enfermedad. Sí No

Si contestó que "sí", pase a la pregunta 20 B.

20 B. ¿Cómo se siente su hijo/a con respecto a sus amigos por tener esta enfermedad? Por favor, marque con un círculo la cara que mejor indique cómo se siente su hijo/a.

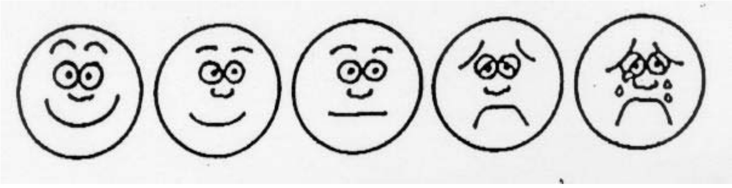

21 A. Los amigos de su hijo/a entienden que tiene una enfermedad. Sí No

Si contestó que "no", pase a la pregunta 21 B.

21 B. ¿Cómo hace que se sienta su hijo/a el hecho de que sus amigos no entiendan que tiene esta enfermedad? Por favor, marque con un círculo la cara que mejor indique cómo se siente su hijo/a.

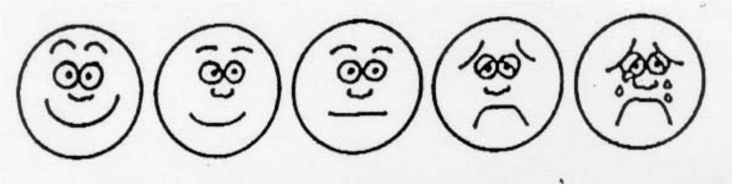

22 A. Una enfermedad afecta el trabajo de su hijo/a en la escuela. Sí No

Si contestó que "sí", pase a la pregunta 22 B

22 B. ¿Cómo hace esta enfermedad sentir a su hijo/a con respecto al trabajo de la escuela? Por favor, marque con un círculo la cara que mejor indique cómo se siente su hijo/a.

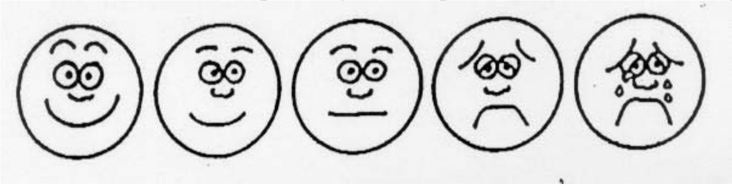

23 A. Su hijo/a falta a la escuela por su enfermedad. Sí No

Si contestó que "sí", pase a la pregunta 23 B.

23 B. ¿Cómo se siente su hijo/a al faltar a la escuela por su enfermedad? Por favor, marque con un círculo la cara que mejor indique cómo se siente su hijo/a.

Copyright © 2007 Lakshmi Nandini Moorthy. Todos los derechos reservados.

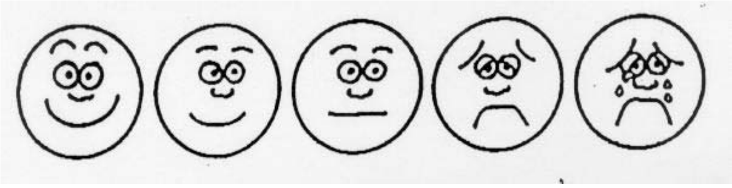

24 A. La enfermedad afecta las actividades de su hijo/a fuera de la escuela. Sí No

Si contestó que "sí", pase a la pregunta 24 B.

24 B. ¿Cómo hace sentir a su hijo/a el hecho de que su enfermedad afecte sus actividades fuera de la escuela? Por favor, marque con un círculo la cara que mejor indique cómo se siente su hijo/a.

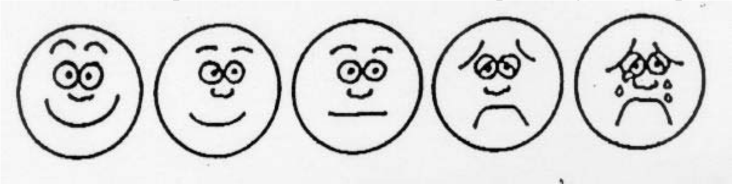

25 A. La enfermedad afecta la manera en que su hijo/a se divierte con sus amigos.

Sí No

Si contestó que "sí", pase a la pregunta 25 B.

25 B. ¿Cómo hace sentir a su hijo/a el hecho de que su enfermedad afecte la manera en que se divierte con sus amigos? Por favor, marque con un círculo la cara que mejor indique cómo se siente su hijo/a.

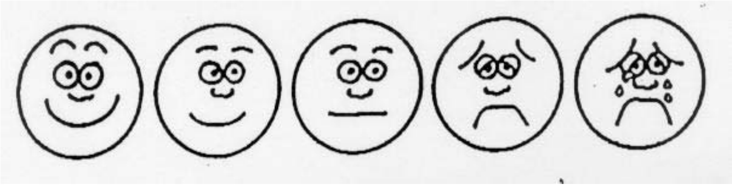

26 A. La enfermedad afecta la participación de su hijo/a en actividades físicas (como por ejemplo: hacer ejercicio en la escuela, correr, caminar, participar en sus juegos favoritos al aire libre).

Sí No

Si contestó que "sí", pase a la pregunta 26B.

26 B. ¿Cómo hace sentir a su hijo/a el hecho de que su enfermedad afecte su participación en actividades físicas (como por ejemplo: hacer ejercicio en la escuela, correr, caminar, participar en sus juegos favoritos al aire libre)? Por favor, marque con un círculo la cara que mejor indique cómo se siente su hijo/a.

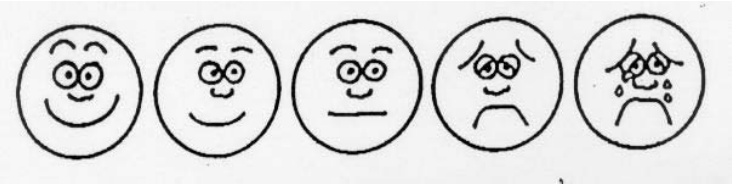

Copyright © 2007 Lakshmi Nandini Moorthy. Todos los derechos reservados.

GENÇLERDE HASTALIĞIN ETKİLERİNİN BASİT ÖLÇÜMÜ (SMILY-Hastalık)

ÇOCUK RAPORU

Çalışma numarası:
Çalışma tarihi: _/_/20

Bu anket için Wong-Baker Ağrı Ölçek Skalasında kullanılan 5 yüz ifadesi D.L.Wong'un izniyle ufak değişikler yapılarak kullanılmıştır. Her sorunun ardından bu yüz ifadeleri cevaplamada kullanılacaktır.

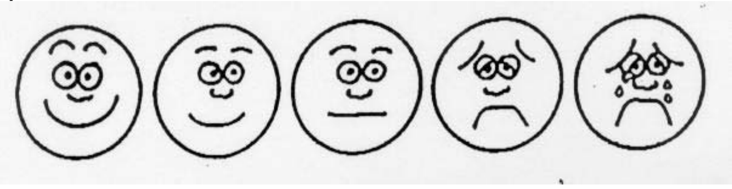

Wong DL, Hockenberry-Eaton M, Wilson D,Winklestein ML, Schwartz P.: Wong's Essentials of Pediatric Nursing, ed. 6.baskı. Louis, 2001, Mosby, p 1301. Mosby'den telif edildi.İzni alınarak yeniden basılmıştır.

SMILY-illness telif hakkı L. Nandini Moorthy, MD MS, RWJMS-UMDNJ, New Brunswick, NJ'ye aittir.Bu nedenle sadece L. Nandini Moorthy, MD MS ve yetki verdiği araştırma görevlisi tarafından kullanılabilir.

SORULAR

Bu ankette size, hastalığınız ve size hissettirdikleri hakkında birçok soru sorulacak. Soruların yanlış cevabı yoktur. Lütfen anlamadığınız herhangi birşey olursa bize danışın. Eğer önerileriniz olursa bunları 5. ve 6. sayfaların arkasına yazabilirsiniz.

Farklı şeyler yaşamınızı etkiler ve sizin mutlu ya da üzgün olmanıza neden olur. Hayatta sizin için önemli olduğunu düşündüğünüz, yapabileceğiniz ya da yapamayacağınız bazı şeyler vardır. Hayatınızdaki tüm bu farklılıklar sizin yaşam kalitenizi oluşturur.

1-Yaşam kalitenizi nasıl değerlendiriyorsunuz? Lütfen yaşam kalitenizi en iyi yansıttığını düşündüğünüz yüz ifadesini işaretleyiniz.

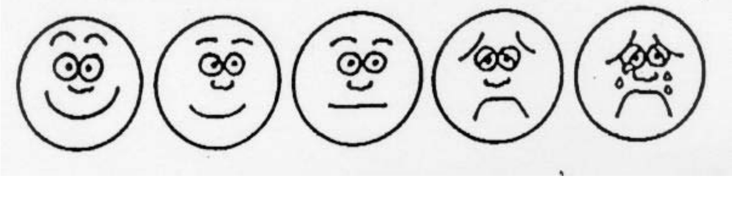

2-Şu anda hastalığınız ne durumda? Lütfen en iyi yansıtan yüz ifadesini işaretleyiniz.

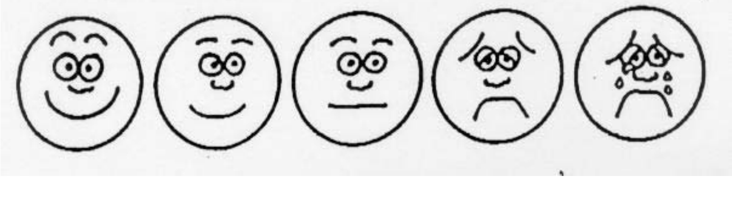

Telif Hakkı © 2007 Lakshmi Nandini Moorthy. Tüm hakları saklıdır.

3-19 arasındaki sorular hastalık nedeniyle son bir ay içerisinde nasıl hissettiğinizi sorgulamaktadır. Lütfen cevabınıza en uygun yüz ifadesini işaretleyiniz.

3-Hasta olmak nasıl hissetmenize sebep oluyor?

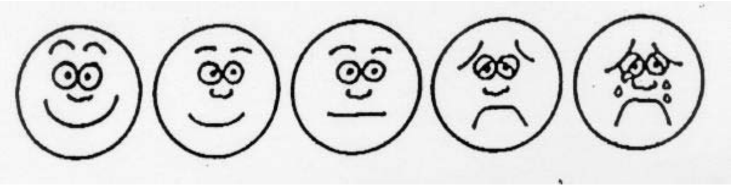

4-Hastalık okula gitme isteğinizi nasıl etkiliyor?

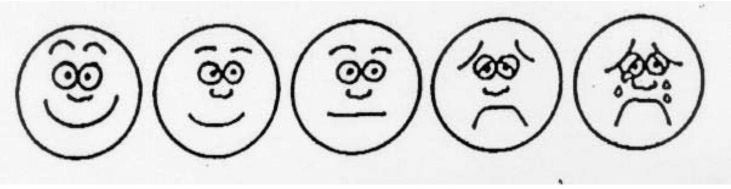

5-Hasta olmak hergün giyinmeniz noktasında duygularınızı nasıl etkiliyor?

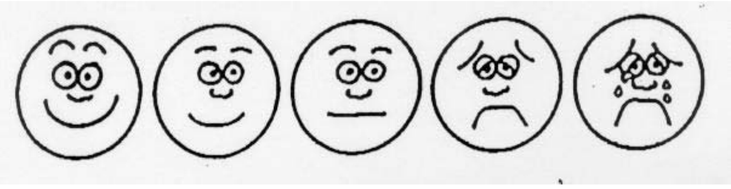

6-Hasta olmak dış görünüşünüz noktasında nasıl hissetmenize neden oluyor?

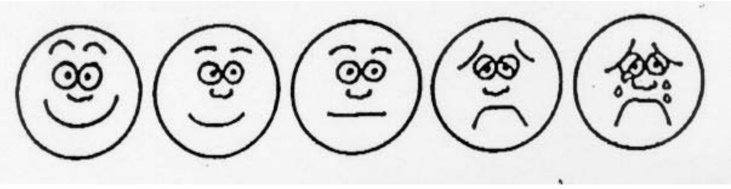

7-Hastalık kendinize bakış açınızı nasıl etkiliyor?

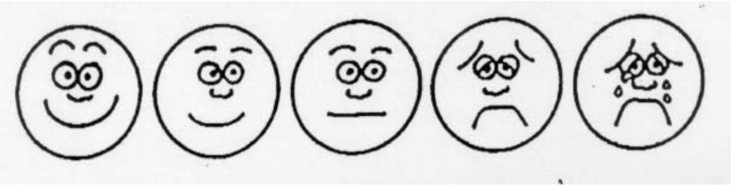

Telif Hakkı © 2007 Lakshmi Nandini Moorthy. Tüm hakları saklıdır.

8-Hastalıkla ilgili konuşmanız gerektiğinde nasıl hissediyorsunuz?

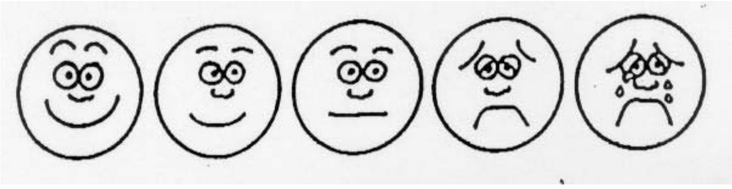

9-Hastalık nedeniyle doktora gitmek nasıl hissetmenize neden oluyor?

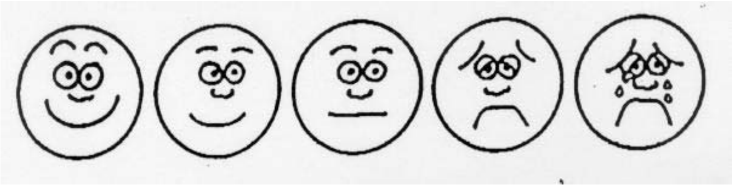

10-Hastalık nedeniyle hastaneye gitmek nasıl hissetmenize neden oluyor?

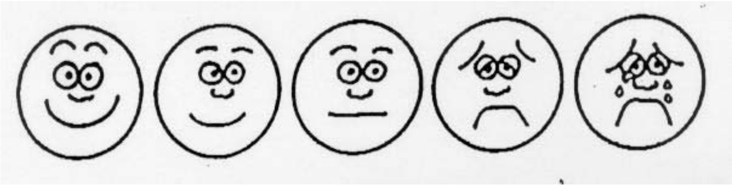

11-Hastalık geleceğinizle ilgili nasıl hissetmenize neden oluyor?

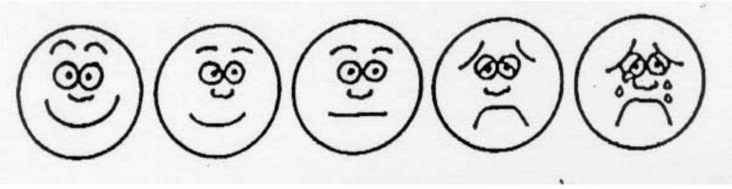

12-Hastalık için ilaç tedavisi almak zorunda olmanız sizi nasıl etkiliyor?

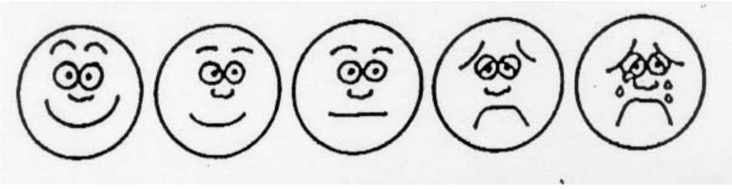

13-Hastalık için aldığınız tedavinin yan etkileri konusunda ne ölçüde endişe duyuyorsunuz?

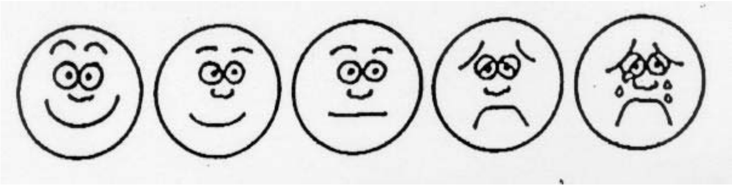

Telif Hakkı © 2007 Lakshmi Nandini Moorthy. Tüm hakları saklıdır.

14-Hastalığınızın ilerleyip daha kötü olması konusunda ne kadar endişelisiniz?

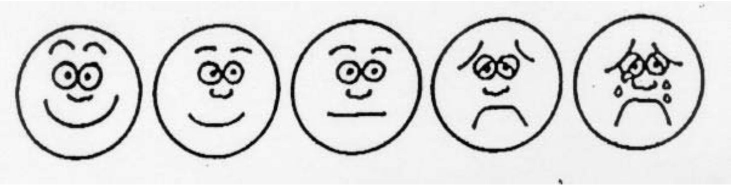

15-Hastalıktan dolayı kendinizi ne kadar yalnız hissediyorsunuz?

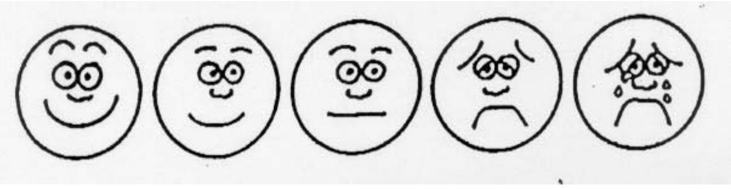

16-Aileniz bu süreçte size kendinizi nasıl hissettiriyor?

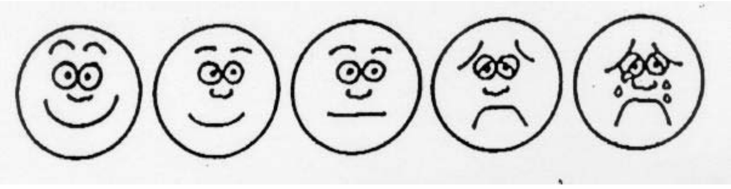

17-Hastalıktan kaynaklanan ağrılar nasıl hissetmenize neden oluyor?

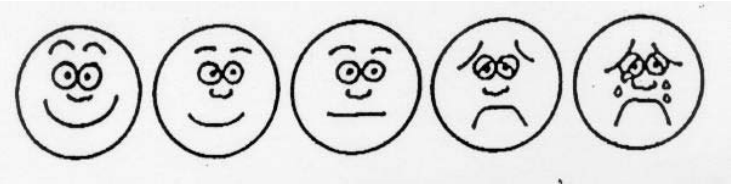

18-Hastalık nedeniyle güneş ışığında dışarı çıkamamak sizi nasıl etkiliyor?

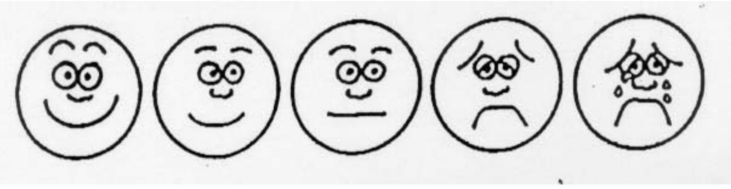

19-Hastalık kendinizi ne kadar yorgun hissetmenize sebep oluyor?

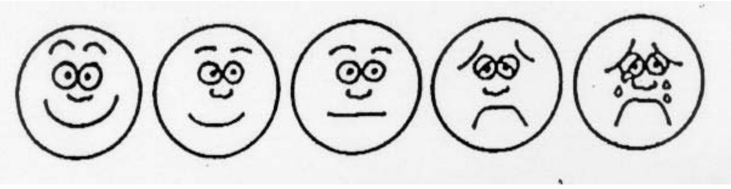

Telif Hakkı © 2007 Lakshmi Nandini Moorthy. Tüm hakları saklıdır.

20A'dan 26A'ya kadar olan sorular evet veya hayır olarak cevaplandırılacaktır. Lütfen direktifleri izleyerek cevaplandırınız.

.

20A-Arkadaşlarım hasta olmam nedeniyle bana karşı değişti. Lütfen eveti ya da hayırı işaretleyiniz. Evet Hayır

*Cevabınız evetse lütfen 20B'yi cevaplayınız.

20B-Hastalık arkadaşlarınız hakkında nasıl hissetmenize neden oluyor? Lütfen hislerinize en uygun yüz ifadesini işaretleyiniz.

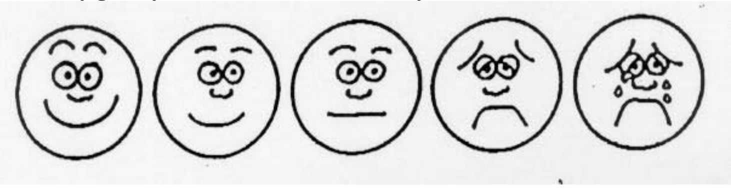

21A-Arkadaşlarım hasta olduğumu anlıyorlar. Evet Hayır

*Cevabınız hayırsa lütfen 20Byi cevaplayınız.

21B-Arkadaşlarınızın hasta olduğunuzu anlamaları nasıl hissetmenize neden oluyor? Lütfen hislerinize en uygun yüz ifadesini işaretleyiniz.

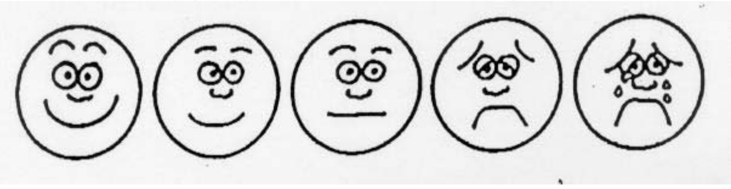

22A-Hastalık okul hayatımı etkiliyor Evet Hayır

*cevabınız evetse lütfen 22Byi cevaplayınız.

22B-Hastalık okul hayatınız hakkında nasıl hissetmenize neden oluyor? Lütfen hislerinize en uygun yüz ifadesini işaretleyiniz.

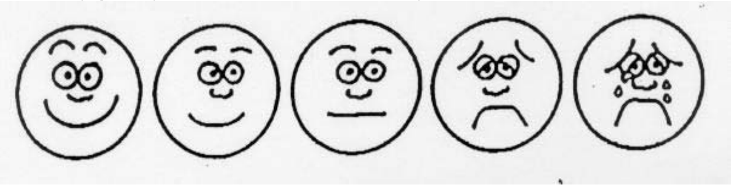

23A-Hastalık nedeniyle derslerimi kaçırıyorum. Evet Hayır.

*Cevabınız evetse lütfen 23Byi cevaplayınız

23B-Hastalık nedeniyle derslerinizi kaçırmak nasıl hissetmenize neden oluyor? Lütfen hislerinize en uygun yüz ifadesini işaretleyiniz.

Telif Hakkı © 2007 Lakshmi Nandini Moorthy. Tüm hakları saklıdır.

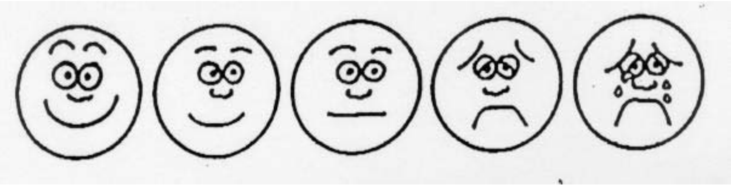

24A-Hastalık okuldışı aktivitelerimi etkiliyor. Evet Hayır

*Cevabınız evetse lütfen 24Byi cevaplayınız

24B-Okul dışı aktivitelerinizin hastalık nedeniyle etkilenmesinden dolayı nasıl hissediyorsunuz? Lütfen hislerinize en uygun yüz ifadesini işaretleyiniz.

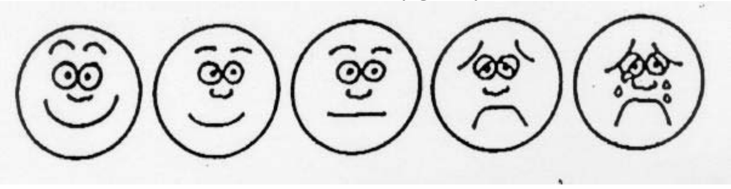

25A-Hastalık arkadaşlarımla eğlenmemi kötü etkiliyor. Evet Hayır

*Cevabınız evetse lütfen 25Byi cevaplayınız

25B-Hastalığınızın arkadaşlarınızla eğlenmenizi etkilemesi nasıl hissetmenize neden oluyor? Lütfen hislerinize en uygun yüz ifadesini işaretleyiniz.

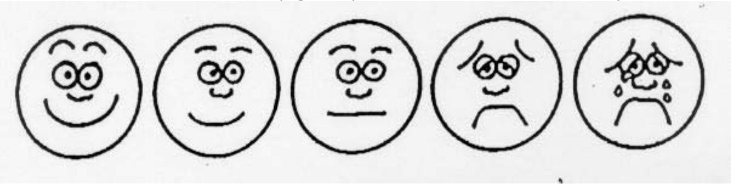

26A-Hastalık fiziksel aktivitelere katılmamı etkiliyor (okuldaki beden eğitimi, koşmak, yürümek ya da en sevdiğim sokak oyunu vb. Evet Hayır

*Cevabınız evetse lütfen 26Byi cevaplayınız.

26B- Hastalığınızın fiziksel aktivitelerinizi etkilemesi nasıl hissetmenize neden oluyor? Lütfen hislerinize en uygun yüz ifadesini işaretleyiniz.

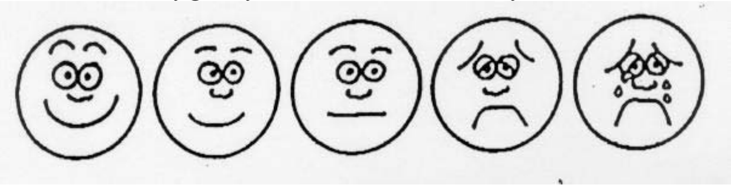

Telif Hakkı © 2007 Lakshmi Nandini Moorthy. Tüm hakları saklıdır.

GENÇLERDE HASTALIĞIN ETKİLERİNİN BASİT ÖLÇÜMÜ (SMILY-Illness)

EBEVEYN TESTİ

Çalışma numarası:
Çalışma tarihi: _/_/20

Bu anket için Wong-Baker Ağrı Ölçek Skalasında kullanılan 5 yüz ifadesi D.L.Wong'un izniyle ufak değişikler yapılarak kullanılmıştır. Her sorunun ardından bu yüz ifadeleri cevaplamada kullanılacaktır.

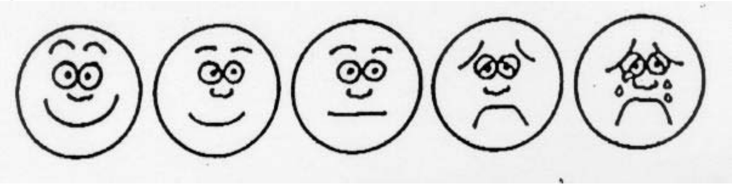

Wong DL, Hockenberry-Eaton M, Wilson D,Winklestein ML, Schwartz P.: Wong's Essentials of Pediatric Nursing, ed. 6.baskı. Louis, 2001, Mosby, p 1301. Mosby'den telif edildi. İzni alınarak yeniden basılmıştır.

SMILY-illness telif hakkı L. Nandini Moorthy, MD MS, RWJMS-UMDNJ, New Brunswick, NJ'ye aittir. Bu nedenle sadece L.Nandini Moorthy,MD MS ve yetki verilen araştırma görevlisi tarafından kullanılabilir.

SORULAR

Bu anket çocuğunuzun hastalığı ve hastalığın ondaki etkileri hakkında birçok soru içermektedir. Soruların yanlış cevabı yoktur. Lütfen anlamadığınız herhangi bir şey olduğunda bize sorun. Eğer herhangi bir yorum yapmak isterseniz 5 ya da 6 numaralı sayfaların arkasını kullanınız.

Farklı şeyler çocuğunuzun yaşamını etkiler ve onun mutlu ya da üzgün olmasına neden olur. Hayatta çocuğunuzun kendisi için önemli olduğunu düşündüğü, yapabileceği ya da yapamayacağı bazı şeyler vardır. Onun hayatındaki tüm bu farklılıklar yaşam kalitesini oluşturur.

1-Çocuğunuzun hayat kalitesini nasıl değerlendiriyorsunuz? Lütfen çocuğunuzun hayat kalitesini en iyi yansıttığını düşündüğünüz yüz ifadesini işaretleyiniz.

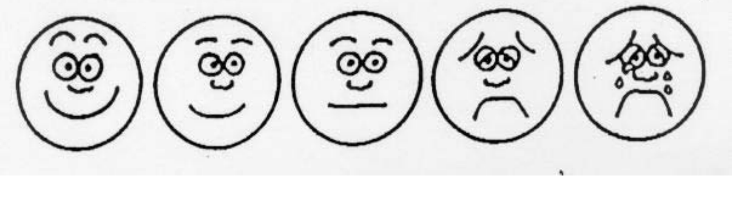

2-Çocuğunuzun hastalığının şu andaki seyri nasıl? Lütfen çocuğunuzun hastalığını en iyi yansıtan yüz ifadesini işaretleyiniz.

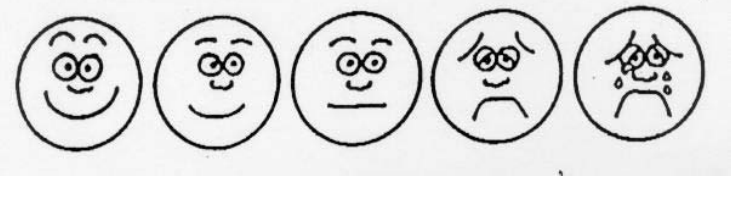

Telif Hakkı © 2007 Lakshmi Nandini Moorthy. Tüm hakları saklıdır.

3-19 arasındaki sorular çocuğunuzun hastalık nedeniyle son bir ay içerisinde nasıl hissettiğini sorgulamaktadır. Lütfen cevabınıza en uygun yüz ifadesini işaretleyiniz.

3- Hasta olması, çocuğunuzun nasıl hissetmesine neden oluyor?

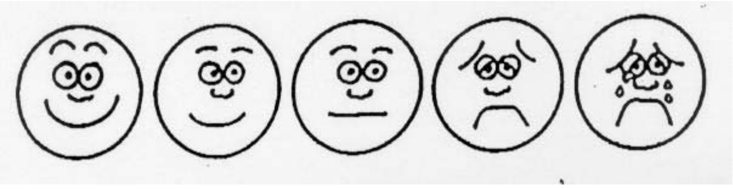

4-Hastalık çocuğunuzun okula gitme isteğini nasıl etkiliyor?

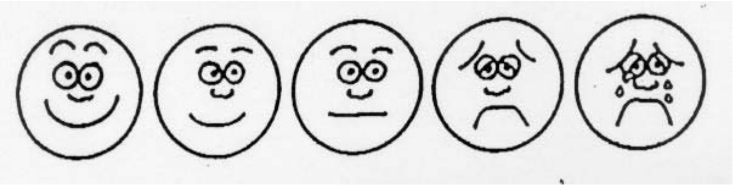

5-Hasta olmak her gün giyinmek noktasında çocuğunuzun duygularını nasıl etkiliyor?

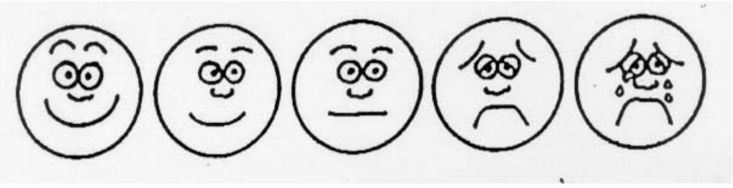

6-Hastalık çocuğunuzun dış görünüşü ile ilgili olarak nasıl hissetmesine neden oluyor?

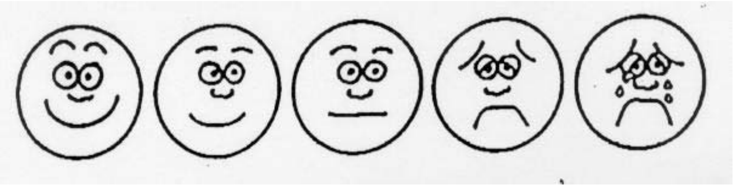

7-Hasta olmak çocuğunuzun kendisine bakış açısını nasıl etkiliyor?

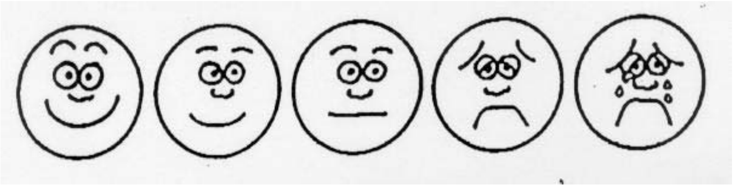

Telif Hakkı © 2007 Lakshmi Nandini Moorthy. Tüm hakları saklıdır.

8-Çocuğunuz hastalığı hakkında konuşması gerektiğinde nasıl hissediyor?

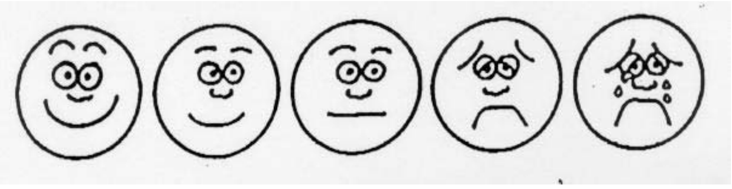

9-Çocuğunuz hastalığı sebebiyle doktora gitmesi gerektiğinde nasıl hissediyor?

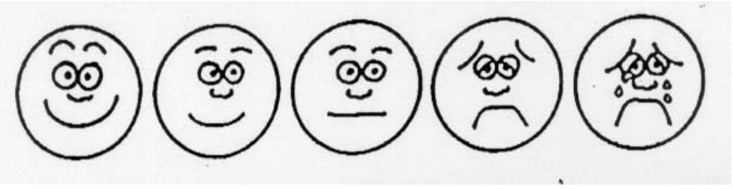

10-Hastalığı nedeniyle hastaneye gittiğinde çocuğunuz nasıl hissediyor?

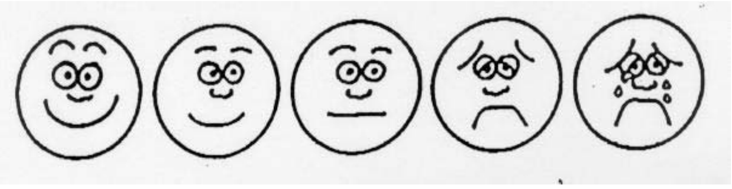

11-Hastalık çocuğunuzun geleceği ile ilgili nasıl hissetmesine neden oluyor?

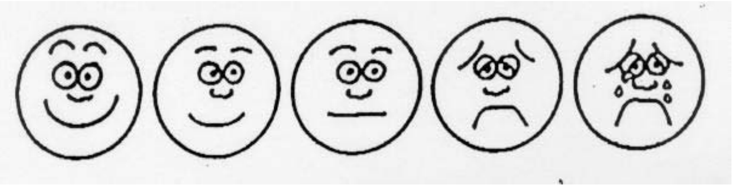

12-Hastalık için ilaç almak zorunda olmak çocuğunuzun duygularını nasıl etkiliyor?

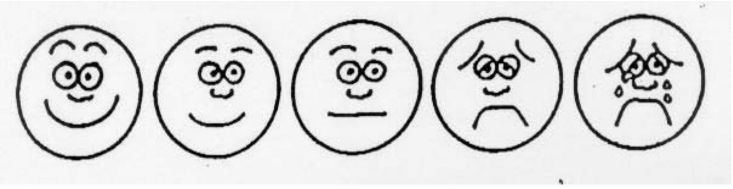

13-Hastalık için aldığı tedavinin yan etkileri konusunda çocuğunuz ne kadar endişe duyuyor?

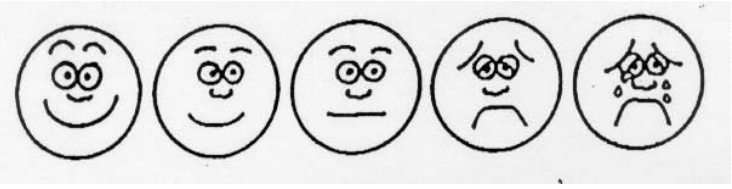

Telif Hakkı © 2007 Lakshmi Nandini Moorthy. Tüm hakları saklıdır.

14-Hastalığın ilerlemesi çocuğunuzu ne ölçüde endişelendiriyor?

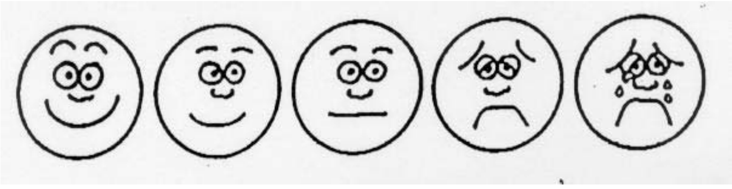

15-Hastalığından dolayı çocuğunuz kendini ne kadar yalnız hissediyor?

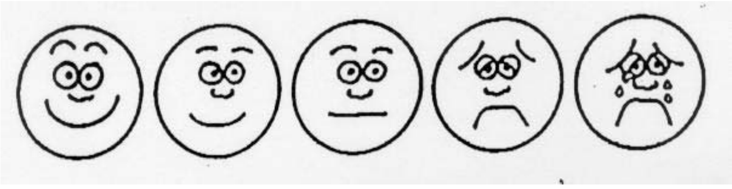

16-Ailesi çocuğunuzun kendisini nasıl hissetmesine neden oluyor?

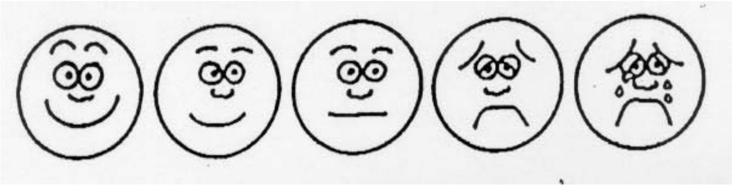

17-Hastalıktan kaynaklanan ağrılarının olması çocuğunuzun nasıl hissetmesine neden oluyor?

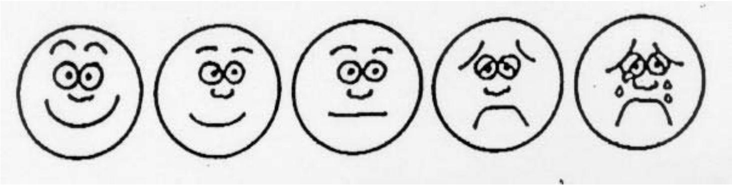

18-Çocuğunuz hastalık nedeniyle güneş ışığında dışarı çıkamamasından dolayı nasıl hissediyor?

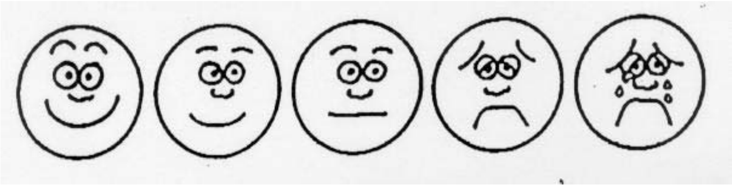

19-Hastalık çocuğunuzun ne kadar yorgun hissetmesine neden oluyor?

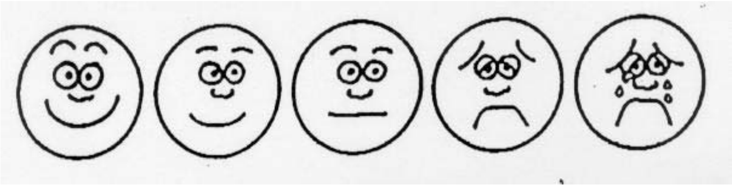

Telif Hakkı © 2007 Lakshmi Nandini Moorthy. Tüm hakları saklıdır.

20-26 arası sorular evet ya da hayır olarak cevaplandırılacaktır. Lütfen direktifleri izleyiniz

20-Çocuğunuzun arkadaşları hatsa olması nedeniyle ona karşı değiştiler. Lütfen evet ya da hayırı işaretleyiniz Evet Hayır
*Cevabınız evetse lütfen 20B numaralı soruyu cevaplayınız.

20B-Hasta olmak çocuğunuzun arkadaşlarına karşı nasıl hissetmesine neden oluyor? Lütfen çocuğunuzun hissettiklerine en uygun olan yüz ifadesini seçiniz.

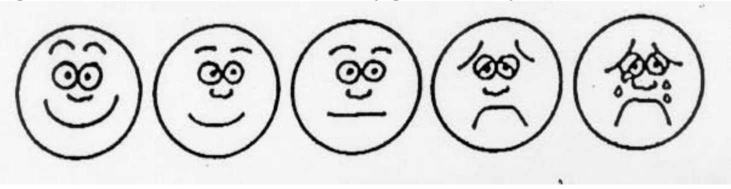

21-Arkadaşları çocuğunuzun hastalığı olduğunu anlıyorlar. Evet Hayır
*Cevabınız hayırsa lütfen 21B numaralı soruyu cevaplandırınız.

21B-Arkadaşlarının onun hasta olduğunu anlamaları çocuğunuza nasıl hissettiriyor? Lütfen çocuğunuzun hissettiklerine en uygun olan yüz ifadesini seçin

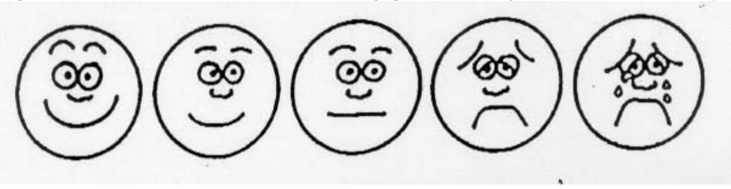

22-Hastalık çocuğunuzun okul hayatını etkiliyor. Evet
Hayır
*Cevabınız evetse lütfen 22B numaralı soruyu cevaplandırınız.

22B-Hastalık çocuğunuzun okul hayatı hakkında nasıl hissetmesine neden oluyor? Lütfen çocuğunuzun hissettiklerine en uygun olan yüz ifadesini seçiniz.

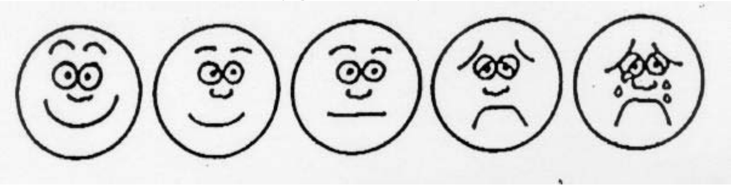

23-Hastalık nedeniyle çocuğunuz okul hayatından geri kalıyor. Evet Hayır
*Cevabınız evetse lütfen 23Byi cevaplandırınız.

23B-Hastalık nedeniyle okul hayatından geri kalması çocuğunuzun nasıl hissetmesine neden oluyor? Lütfen çocuğunuzun hissettiklerine en uygun olan yüz ifadesini seçiniz.

Telif Hakkı © 2007 Lakshmi Nandini Moorthy. Tüm hakları saklıdır.

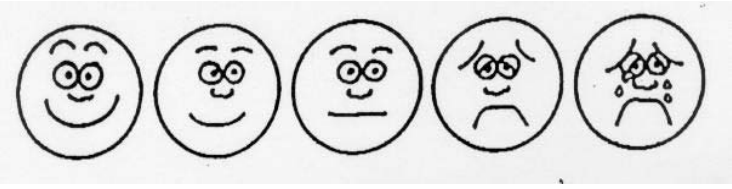

24-Hastalık çocuğunuzun okul dışı aktivitelerini etkiliyor. Evet Hayır
*Cevabınız evetse lütfen 24Byi cevaplandırınız.

24-BÇocuğunuz, okul dışı aktivitelerinin hastalık nedeniyle etkilenmesinden dolayı nasıl hissediyor? Lütfen çocuğunuzun hissettiklerine en uygun olan yüz ifadesini seçiniz.

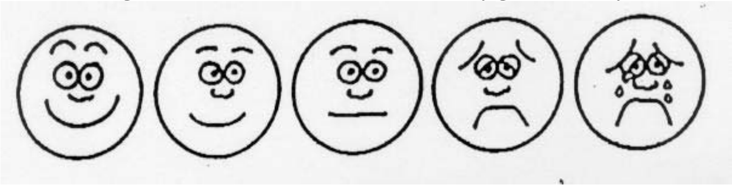

25-Hastalık çocuğunuzun arkadaşlarıyla eğlenmesini kötü etkiliyor. Evet Hayır
*Cevabınız evetse lütfen 25Byi cevaplandırınız.

25B-Çocuğunuzun hastalığı nedeniyle arkadaşlarıyla eğlenmesinin olumsuz etkilenmesi onun nasıl hissetmesine neden oluyor? Lütfen çocuğunuzun hissettiklerine en uygun olan yüz ifadesini seçiniz

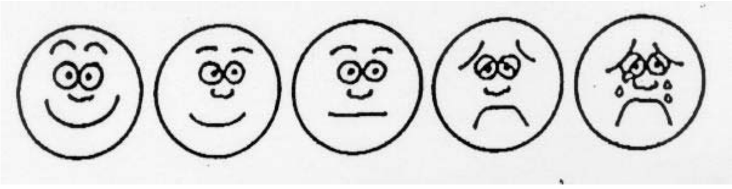

26-Hastalık çocuğunuzun fiziksel aktivitelere katılımını etkiliyor (okuldaki beden eğitimi, koşma, yürüme, en sevdiği sokak oyunu vb.) Evet Hayır
*Cevabınız evetse lütfen 26B numaralı soruyu cevaplandırınız.

26B-Fiziksel aktivitelere katılımının(okuldaki beden eğitimi, koşma, yürüme, en sevdiği sokak oyunu vb.) olumsuz etkilenmesi, çocuğunuzun nasıl hissetmesine neden oluyor? Lütfen çocuğunuzun hissettiklerine en uygun olan yüz ifadesini seçiniz

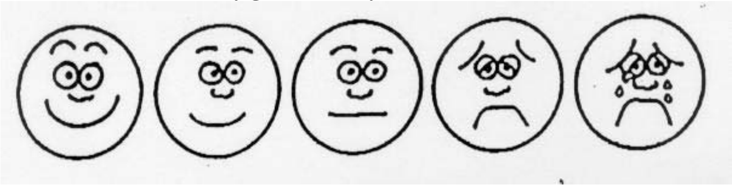

Telif Hakkı © 2007 Lakshmi Nandini Moorthy. Tüm hakları saklıdır.

UMLINGANISELO OLULA WEMPEMBELELO YESIGULO KUBANTU ABATSHA (SMILY-Illness)- Ingxelo yomntwana (XHOSA)

INomboloyophando….Umhla _/_/20

Kweli phepha lemibuzo, sithathe le mifanekiso yobuso mi-5 ingezantsi kwiSikali sokulinganisa Iintlungu NGOBUSO sikaWong-Baker FACES; esithe salungiswa ngemvume ka-D.L. Wong. Le mifanekiso yobuso mi-5 izakuvela phantsi kombuzo ngamnye.

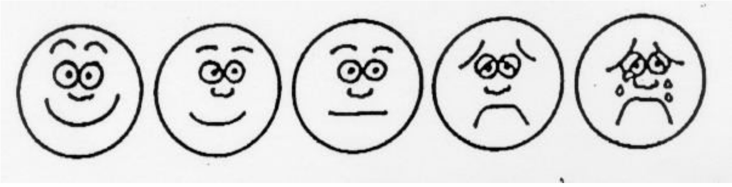

Ivela kuWong DL, Hockenberry-Eaton M, Wilson D,Winklestein ML, Schwartz P.: I-Essentials of Pediatric NursingkaWong, ed. 6, eSt.Louis, 2001, Mosby, iphepha 1301.Ilungelo lobunikazi lelika Mosby.Iphindwe yashicilelwa ngemvume.

Ilungelo lobunikazi elithi SMILY-illness ngu-L. Nandini Moorthy, MD MS, RWJMS-UMDNJ, New Brunswick, NJ. Okwangoku, iphepha lemibuzo lingasetyenziswa kuphela ngu-L. Nandini Moorthy, MD MS kunye nabasebenzi bakhe abamiselwe olu phando.

IMIBUZO

Kweli phepha lemibuzo, siza kubuza imibuzo emininzi ngesigulo sakho kunye neemvakalelo zakho. Akukho mpendulo engeyiyo.Nceda usibuze nantonina ongayiqondiyo. Ukuba unqwenela ukubhala naziphi na izimvo, nceda ukwenze oku, emva kwephepha lesi-5 kunye nele-6.

Izinto ezahlukeneyo zithi zichaphazele ubomi bakho kwaye oko kwenza ukuba wonwabe okanye udakumbe.Kukho izinto ocinga ukuba zibalulekile kuwe, okwaziyo ukuzenza(okanye ongakwaziyo ukuzenza).? Zonke ezi zinto zohlukeneyo kubomi bakho zithi zonke zenze umgangatho wobomi bakho.

1. **Unjani umgangatho wobomi bakho? Nceda wenze isangqa kubuso obuthi bubonise ngcono umgangatho wobomi bakho.**

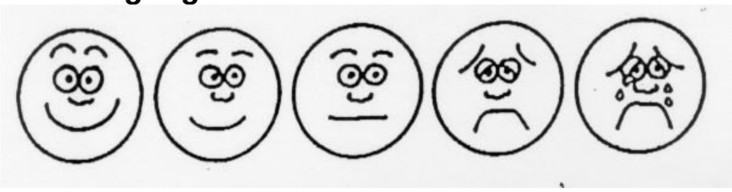

2. **Sinjani isigulo sakho ngoku? Nceda wenze isangqa kubuso obuthi bubonise imeko yesigulo sakho.**

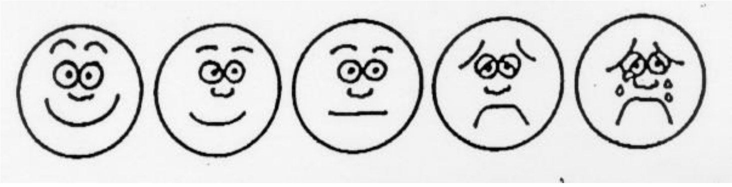

Copyright © 2007 Lakshmi Nandini Moorthy. All rights reserved.

Imibuzo 3-19
Yimibuzo emalunga nokuba ubuziva njani kule nyanga iphelileyo ngenxa yesigulo sakho. Nceda wenze isangqa kubuso obuthi bubonise impendulo yakho.

3. Ukuba nesigulo kukwenza uzive njani?

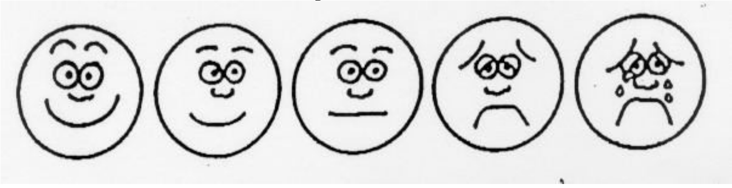

4. Ukuba nesigulo kukwenza uzive njani ngokuya esikolweni?

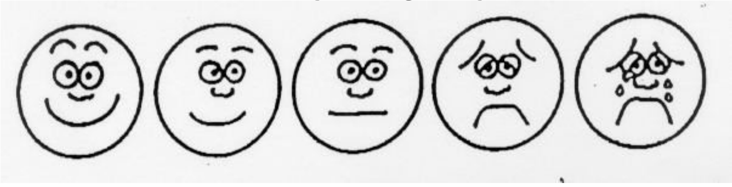

5. Ukuba nesigulo kukwenza uzive njani ngokunxiba yonke imihla?

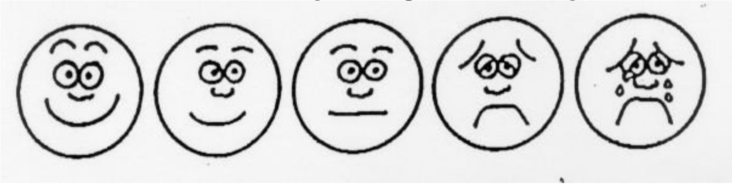

6. Ukuba nesigulo kukwenza uzive njani ngendlela ojongeka ngayo?

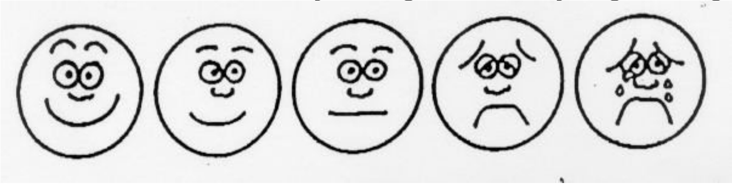

7. Ukuba nesigulo kukwenza uzive njani ngesiqu sakho?

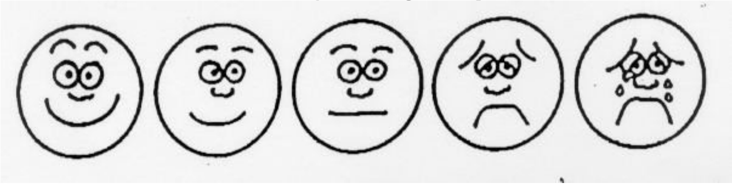

8. Uziva njani xa ufuna ukuthetha ngesigulo sakho?

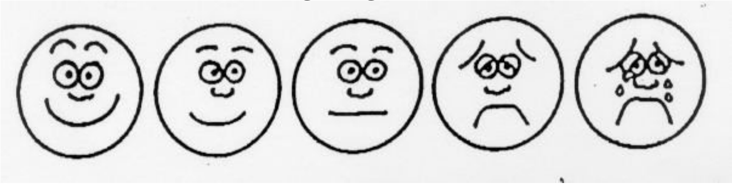

Copyright © 2007 Lakshmi Nandini Moorthy. All rights reserved.

9. Uziva njani ngokuya kwagqirha ngenxa yesigulo sakho?

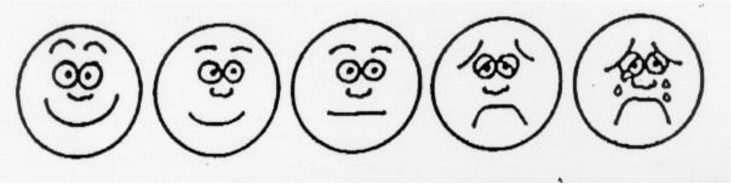

10. Uziva njani ngokuya esibhedlele ngenxa yesigulo sakho?

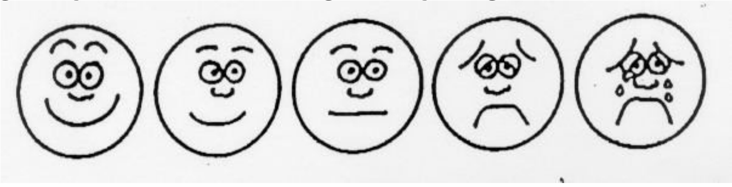

11. Ukuba nesigulo kukwenza uzive njani ngekamva lakho?

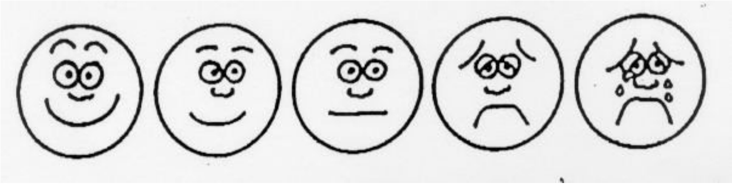

12. Uziva njani ngokusebenzisa amayeza esigulo sakho?

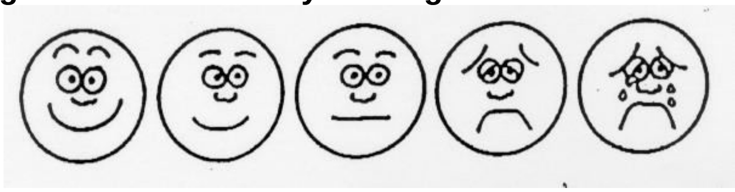

13. Ukhathazeke kangakanani ngezinye zeziphumo ezibangelwa kukusebenzisa amayeza esigulo sakho?

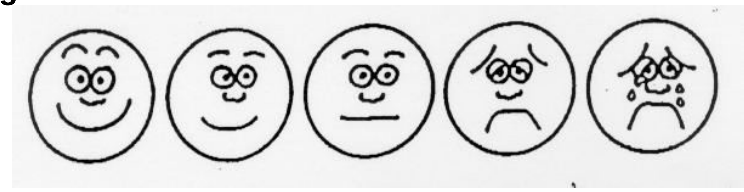

14. Ukhathazeke kangakanani ngokuqatsela kwesigulo sakho?

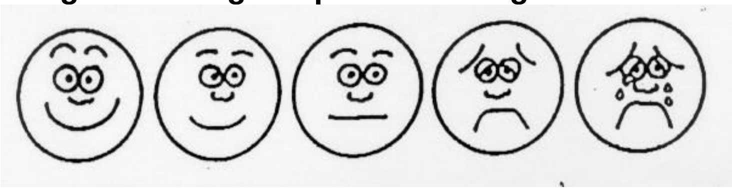

Copyright © 2007 Lakshmi Nandini Moorthy. All rights reserved.

15. Uziva ulikheswa kangakanani ngenxa yesigulo sakho?

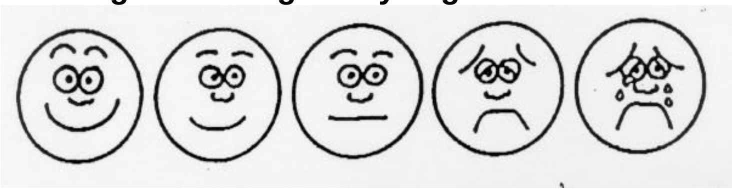

16. Intsapho yakho ikwenza uzive njani?

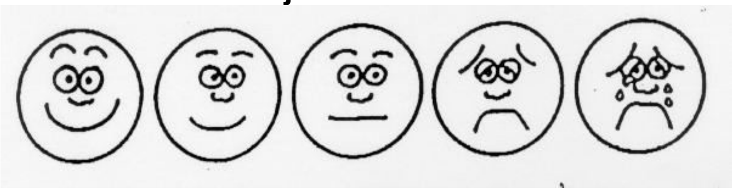

17. Intlungu yesigulo sakho ikwenza uzive njani?

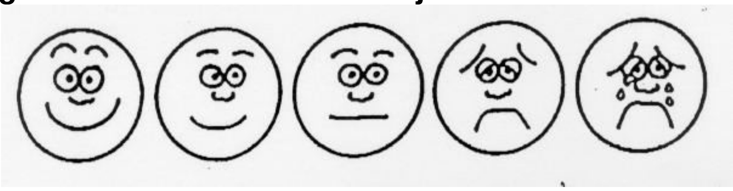

18. Uziva njani malunga nokungakwazi ukuphuma phandle elangeni ngenxa yesigulo sakho?

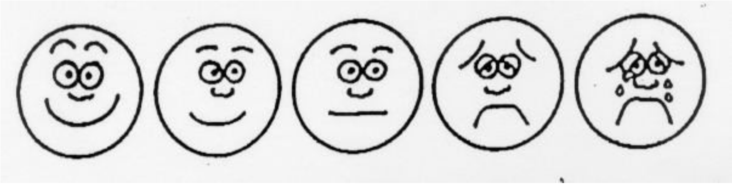

19. Isigulo sakho sikwenza uzive udinwe kangakanani?

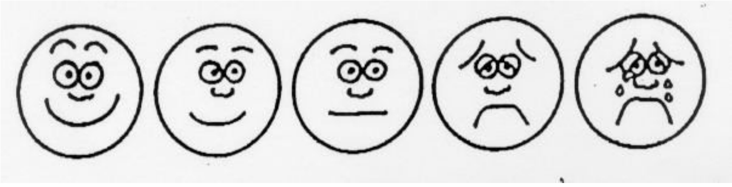

Copyright © 2007 Lakshmi Nandini Moorthy. All rights reserved.

Kwimibuzo 20A-26A, phendula uthi " ewe"okanye "hayi." Emva koko, nceda ulandele imiyalelo.

20 A. Abahlobo bam batshintshile ngenxa yesigulo sam. Nceda wenze isangqa ku-"ewe" okanye ku-"hayi." **Ewe** **Hayi**

Ukuba impendulo ngu-"ewe", yiya ku-20B.

20 B. Isigulo sakho sikwenza uzive njani ngabahlobo bakho?
Nceda wenze isangqa kubuso obubonisa ukuba uziva njani?

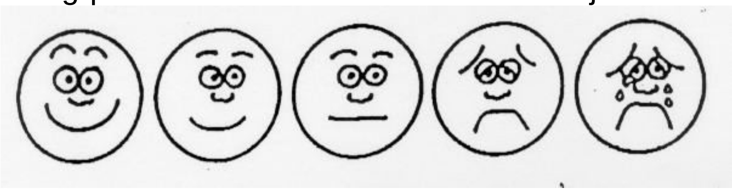

21 A. Abahlobo bam bayayiqonda into yokuba ndinesigulo. **Ewe** **Hayi**

Ukuba impendulo ngu-"hayi", yiya kumbuzo 21B.

21 B. Uzivanjani ngabahlobo bakho abangaqondiyo ukuba unesigulo? Nceda wenze isangqa kubuso obubonisa ukuba uzivanjani.

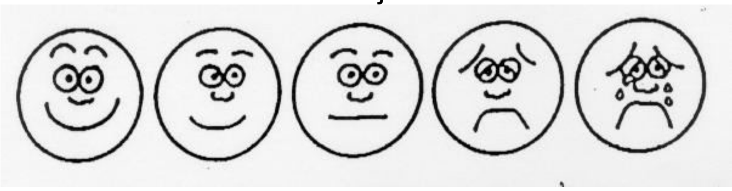

22 A. Isigulo sam sichaphazela umsebenzi wam wesikolo. **Ewe** **Hayi**

Ukuba impendulongu-"ewe"yiya ku-22 B

22 B. Ukuba nesigulo kukwenza uzive njani ngomsebenzi wakho wesikolo? Nceda wenze isangqa kubuso obubonisa ukuba uzivanjani.

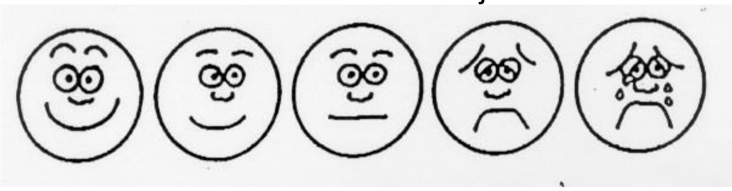

23 A. Ndiphoswa sisikolo ngenxa yesigulo sam. **Ewe** **Hayi**

Ukuba impendulo ngu-"ewe", yiya kumbuzo 23 B

23 B. Uziva njani ngokuphoswa sisikolo ngenxa yesigulo sakho?
Nceda wenze isangqa kubuso obubonisa ukuba uziva njani.

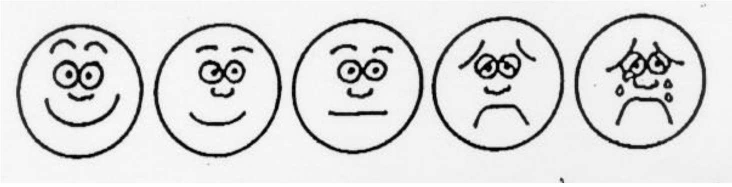

Copyright © 2007 Lakshmi Nandini Moorthy. All rights reserved.

24 A. Isigulo sam sichaphazela inxaxheba yam ngaphandle kwesikolo. Ewe Hayi

Ukuba impendulo ngu-"ewe" yiya kumbuzo 24 B

24 B. Uziva njani ngokuchaphazeleka kwenxaxheba yakho ngaphandle kwesikolo ngenxa yesigulo? Nceda wenze isangqa kubuso obubonisa ukuba uziva njani.

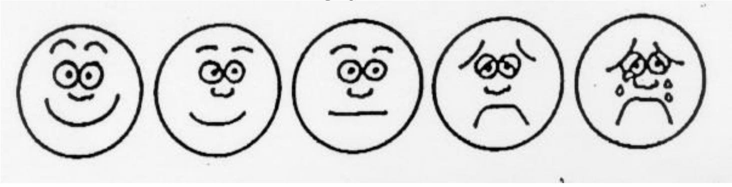

25 A. Isigulo sam sichaphazela ulonwabo endiba nalo nabahlobo bam. Ewe Hayi

Ukuba impendulo ngu-"ewe"yiya kumbuzo 25 B.

25 B. Uziva njani ngendlela isigulo sakho esichaphazela ngayo ulonwabo lwakho kunye nabahlobo bakho? Nceda wenze isangqa kubuso obubonisa ukuba uziva njani.

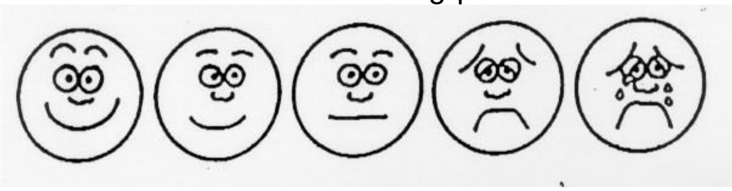

26 A. Isigulo sam sichaphazela inxaxheba yam kwimithambo yomzimba (njengokuzilolonga esikolweni, ukubaleka, ukuhamba, ukudlala imidlalo endiyithandayo ngaphandle). Ewe Hayi

Ukuba impendulo ngu-"ewe"yiya kumbuzo 26 B.

26 B. Uziva njani ngesigulo sakho esichaphazela ukuthatha kwakho inxaxheba kwimithambo yomzimba (njengokuzilolonga esikolweni, ukubaleka, ukuhamba, ukudlala imidlalo oyithandayo ngaphandle)? Nceda wenze isangqa kubuso obubonisa ukuba uzivanjani.

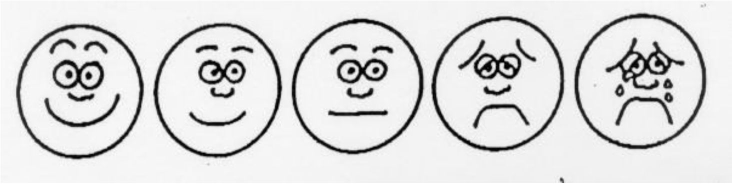

Copyright © 2007 Lakshmi Nandini Moorthy. All rights reserved.

UMLINGANISELO OLULA WEMPEMBELELO YESIGULO KUBANTU ABATSHA (SMILY-Illness) – Eyomzali (XHOSA)

INombolo Yophando….Umhla _/_/20

Kweli phepha lemibuzo, sithathe le mifanekiso yobuso mi-5 ingezantsi kwiSikali sokulinganisa Iintlungu NGOBUSO sikaWong-Baker FACES; esithe salungiswa ngemvume ka-D.L. Wong. Le mifanekiso yobuso mi-5 izakuvela phantsi kombuzo ngamnye.

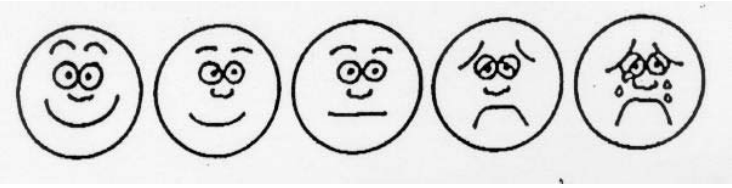

Ivela kuWong DL, Hockenberry-Eaton M, Wilson D,Winklestein ML, Schwartz P.: I-Essentials of Pediatric NursingkaWong, ed. 6, eSt.Louis, 2001, Mosby, iphepha 1301.Ilungelo lobunikazi lelika Mosby.Iphindwe yashicilelwa ngemvume.

Ilungelo lobunikazi elithi SMILY ngu-L. Nandini Moorthy, MD MS, RWJMS-UMDNJ, New Brunswick, NJ. Okwangoku, iphepha lemibuzo lingasetyenziswa kuphela ngu-L. Nandini Moorthy, MD MS kunye nabasebenzi bakhe abamiselwe olu phando.

IMIBUZO

Kweli phepha lemibuzo, siza kubuza imibuzo emininzi ngesigulo somntwana wakho kunye neemvakalelo zakho. Akukho mpendulo engeyiyo nceda usibuze nantonina ongayiqondiyo. Ukuba unqwenela ukubhala naziphi na izimvo, nceda ukwenze oku, Emva kwephepha lesi-5 kunye nele-6.

Izinto ezahlukeneyo zithi zichaphazele ubomi bakho kwaye oko kwenza ukuba wonwabe okanye udakumbe.Kukho izinto ocinga ukuba zibalulekile kuwe, okwaziyo ukuzenza (okanye ongakwaziyo ukuzenza)? Zonke ezi zinto zohlukeneyo kubomi bakho zithi zonke zenze umgangatho wobomi bakho.

1. **Unjani umgangatho wobomi bakho?**
   Nceda wenze isangqa kubuso obuthi bubonise ngcono umgangatho wobomi bakho.

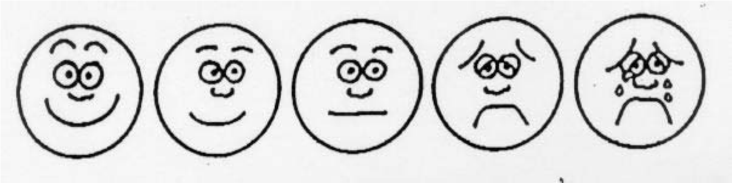

2. **Sinjani isigulo somntwana wakho ngoku?**
   Nceda wenze isangqa kubuso obuthi bubonise imeko yesigulo somntwana wakho.

Copyright © 2007 Lakshmi Nandini Moorthy. All rights reserved.

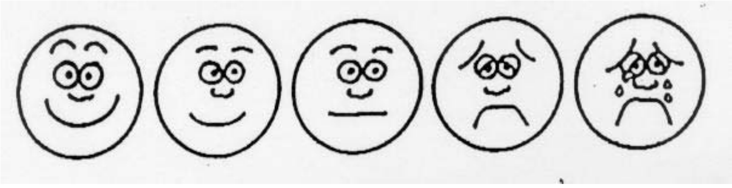

Imibuzo 3-19
Yimibuzo emalunga nokuba ubuziva njani kulenyanga iphelileyo ngenxa yesigulo sakho. Nceda wenze isangqa kubuso obuthi bubonise impendulo yakho.

3. Ukuba nesigulo kwenza umntwana wakho azive njani?

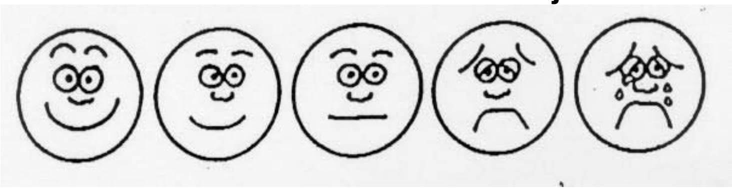

4. Ukuba nesigulo komntwana wakho kumenza azive njani ngokuya esikolweni?

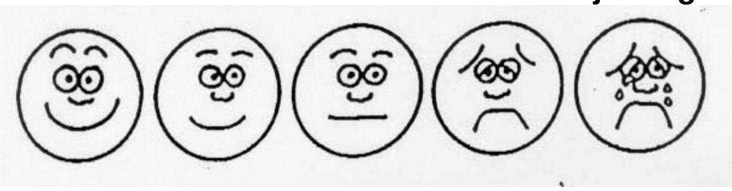

5. Ukuba nesigulo komntwana wakho kumenza azive njani ngokunxiba yonke imihla?

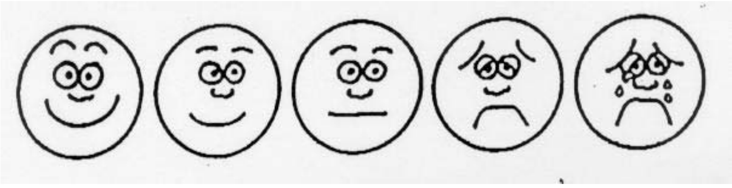

6. Ukuba nesigulo komntwana wakho kumenza azive njani ngendlela ajongeka ngayo?

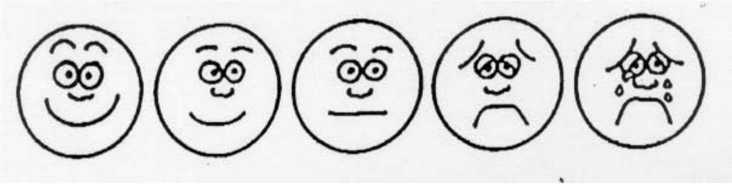

Copyright © 2007 Lakshmi Nandini Moorthy. All rights reserved.

7. Ukuba nesigulo komntwana wakho kumenza azive njani ngesiqu sakhe?

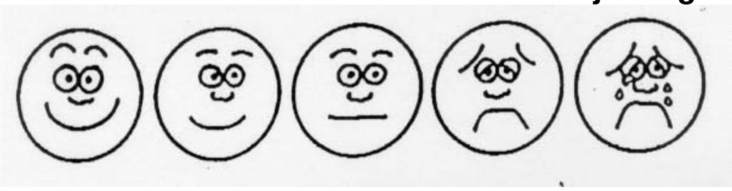

8. Uziva njani umntwana wakho xa efuna ukuthetha ngesigulo sakhe?

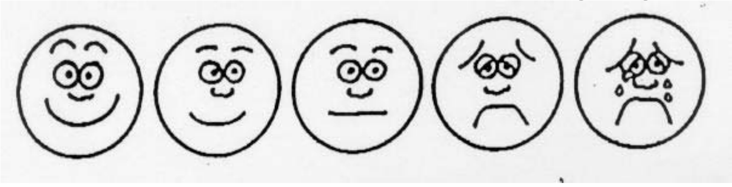

9. Uziva njani umntwana wakho ngokuya kwagqirha ngenxa yesigulo sakhe?

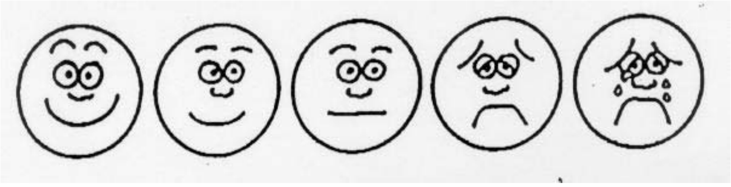

10. Uziva njani umntwana wakho ngokuya esibhedlele ngenxa yesigulo sakhe?

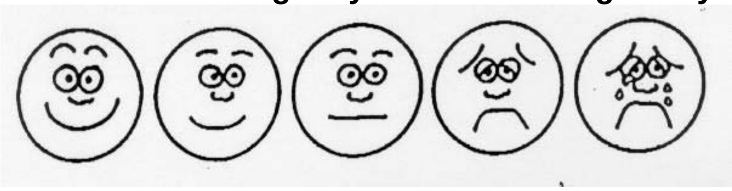

11. Ukuba nesigulo komntwana wakho kumenza azive njani ngekamva lakhe?

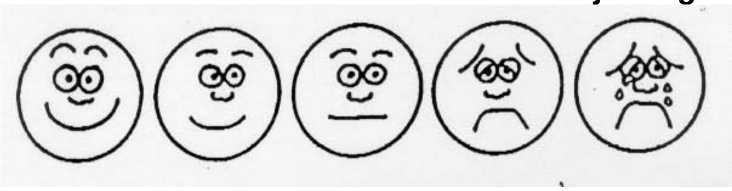

12. Uziva njani umntwana wakho ngokusebenzisa amayeza esigulo sakhe?

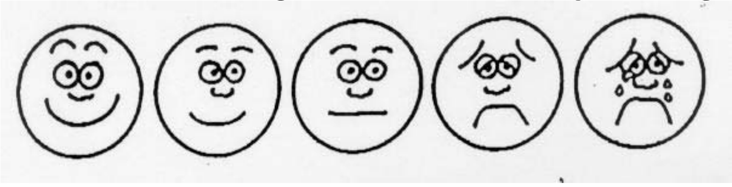

13. Ukhathazeke kangakanani umntwana wakho ngezinye zeziphumo ezibangelwa

Copyright © 2007 Lakshmi Nandini Moorthy. All rights reserved.

kukusebenzisa amayeza esigulo sakhe?

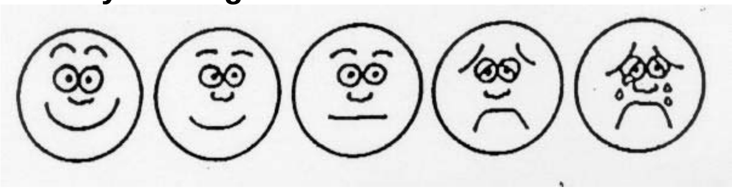

14. Ukhathazeke kangakanani umntwana wakho ngokuqatsela kwesigulo sakhe?

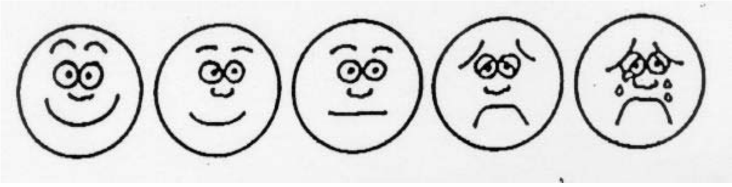

15. Uziva elikheswa kangakanani umntwana wakho ngenxa yesigulo sakhe?

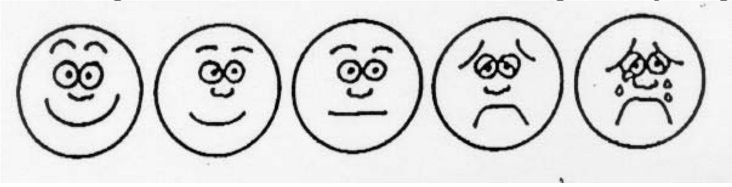

16. Intsapho yomntwana wakho imenza azive njani?

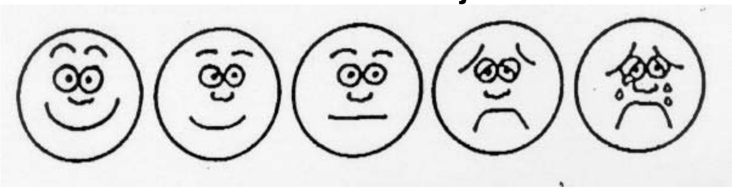

17. Intlungu yesigulo sakhe imenza azive njani umntwana wakho?

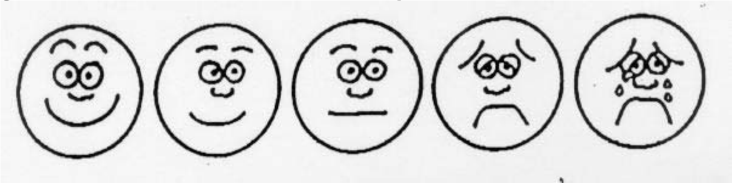

18. Uziva njani umntwana wakho ngokungakwazi ukuphuma phandle elangeni ngenxa yesigulo sakhe?

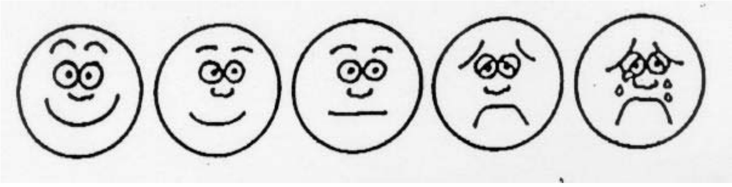

Copyright © 2007 Lakshmi Nandini Moorthy. All rights reserved.

19. Isigulo somntwana wakho simenza azive edinwe kangakanani?

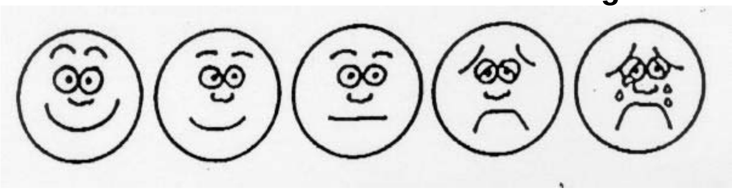

Kwimibuzo 20A-26A, phendula uthi " ewe"okanye "hayi." Ngoko, nceda ulandele imiyalelo.

20 A. Abahlobo bomntwana wakho batshintshile ngenxa yesigulo sakhe. Nceda wenze isangqa ku-"ewe" okanye ku-"hayi."

Ewe Hayi

Ukuba impendulo ngu-"ewe", yiya ku-20B.
20 B. Ukuba nesigulo kumenza azive njani umntwana wakho ngabahlobo bakhe? Nceda wenze isangqa kubuso obubonisa iimvakalelo zomntwana wakho.

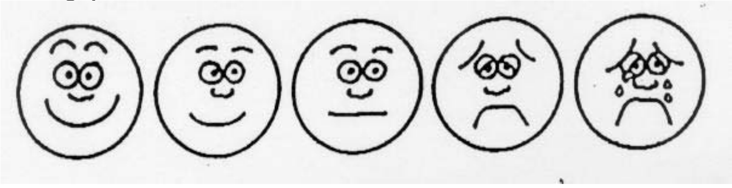

21 A. Abahlobo bomntwana wakho bayayiqonda into yokuba unesigulo. Ewe Hayi

Ukuba impendulo ngu-"hayi", yiya kumbuzo 21B.
21 B. Umntwana wakho uziva njani ngabahlobo bakhe abangaqondiyo ukuba unesigulo? Nceda wenze isangqa kubuso obubonisa ukuba umntwana wakho uziva njani.

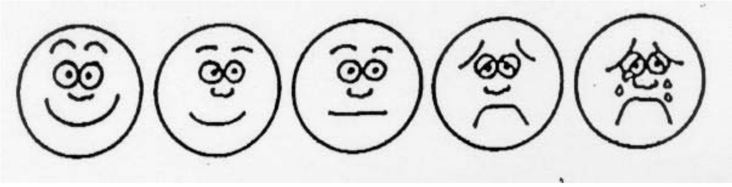

22 A. Isigulo sichaphazela umsebenzi womntwana wam wesikolo. Ewe Hayi

Ukuba impendulongu-"ewe"yiya ku-22 B
22 B. Ukuba nesigulo kwenza umntwana wakho azive njani ngomsebenzi wakhe wesikolo?
Nceda wenze isangqa kubuso obubonisa ukuba uzivanjani.

Copyright © 2007 Lakshmi Nandini Moorthy. All rights reserved.

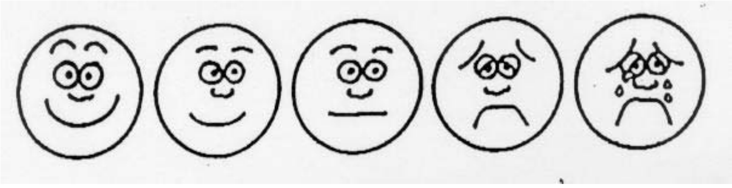

23 A. Umntwana wakho uphoswa sisikolo ngenxa yesigulo sakhe. Ewe Hayi

Ukuba impendulo ngu-"ewe", yiya kumbuzo 23 B

23 B. Uziva njani ngokuphoswa sisikolo ngenxa yesigulo sakho?

Nceda wenze isangqa kubuso obubonisa ukuba uziva njani.

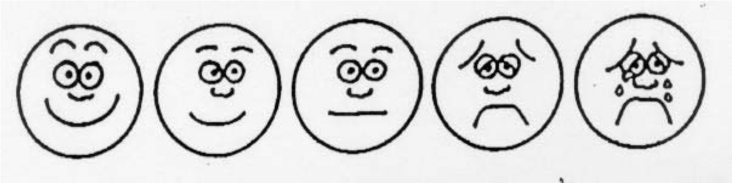

24 A. Esi sigulo sichaphazela inxaxheba yomntwana wam ngaphandle kwesikolo.
Ewe Hayi

Ukuba impendulo ngu-"ewe"yiya kumbuzo 24 B

24 B. Uziva njani ngokuchaphazeleka kwenxaxheba yomntwana wakho ngaphandle kwesikolo? Nceda wenze isangqa kubuso obubonisa ukuba umntwana wakho uziva njani.

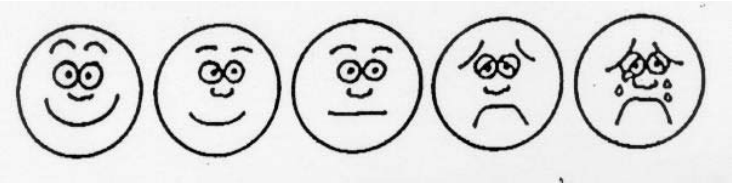

25 A. Esi sigulo sichaphazela ulonwabo aba nalo umntwana wam nabahlobo bakhe.
Ewe Hayi

Ukuba impendulo ngu-"ewe"yiya kumbuzo 25 B.

25 B. Umntwana wakho uziva njani ngendlela isigulo sakhe esichaphazela ngayo ulonwabo lwakhe kunye nabahlobo bakhe? Nceda wenze isangqa kubuso obubonisa ukuba uzivanjani.

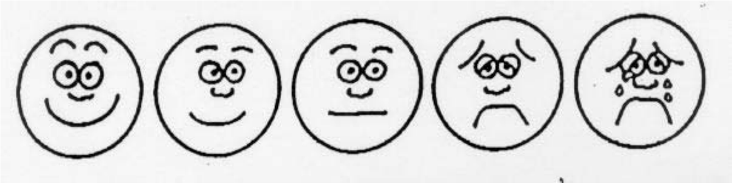

26 A. Esi sigulo sichaphazela inxaxheba yomntwana wam kwimithambo yomzimba

Copyright © 2007 Lakshmi Nandini Moorthy. All rights reserved.

(njengengokuzilolonga esikolweni, ukubaleka, ukuhamba, ukudlala imidlalo ayithandayo ngaphandle). Ewe Hayi

Ukuba impendulo ngu-“ewe”yiya kumbuzo 26 B.

26 B. Umntwana wakho uziva njani ngesigulo sakhe esichaphazela ukuthatha kwakhe inxaxheba kwimithambo yomzimba(njengokuzilolonga esikolweni, ukubaleka, ukuhamba, ukudlala imidlalo ayithandayo ngaphandle)? Nceda wenze isangqa kubuso obubonisa ukuba uzivanjani.

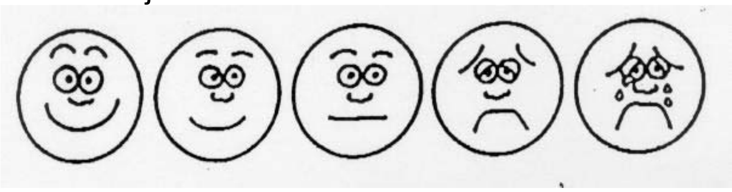

Copyright © 2007 Lakshmi Nandini Moorthy. All rights reserved.
